# Catalytic synthesis of phenols with nitrous oxide

**DOI:** 10.1038/s41586-022-04516-4

**Published:** 2022-04-27

**Authors:** Franck Le Vaillant, Ana Mateos Calbet, Silvia González-Pelayo, Edward J. Reijerse, Shengyang Ni, Julia Busch, Josep Cornella

**Affiliations:** 1 Max-Planck-Institut für Kohlenforschung, Mülheim an der Ruhr, Germany; 2 Max-Planck-Institut für Chemische Energiekonversion, Mülheim an der Ruhr, Germany

**Keywords:** Homogeneous catalysis, Synthetic chemistry methodology

## Abstract

The development of catalytic chemical processes that enable the revalorization of nitrous oxide (N_2_O) is an attractive strategy to alleviate the environmental threat posed by its emissions^1–6^. Traditionally, N_2_O has been considered an inert molecule, intractable for organic chemists as an oxidant or O-atom transfer reagent, owing to the harsh conditions required for its activation (>150 °C, 50‒200 bar)^7–11^. Here we report an insertion of N_2_O into a Ni‒C bond under mild conditions (room temperature, 1.5–2 bar N_2_O), thus delivering valuable phenols and releasing benign N_2_. This fundamentally distinct organometallic C‒O bond-forming step differs from the current strategies based on reductive elimination and enables an alternative catalytic approach for the conversion of aryl halides to phenols. The process was rendered catalytic by means of a bipyridine-based ligands for the Ni centre. The method is robust, mild and highly selective, able to accommodate base-sensitive functionalities as well as permitting phenol synthesis from densely functionalized aryl halides. Although this protocol does not provide a solution to the mitigation of N_2_O emissions, it represents a reactivity blueprint for the mild revalorization of abundant N_2_O as an O source.

## Abstract (web summary)

A study demonstrates that nitrous oxide can act as the source of O in a catalytic conversion of aryl halides to phenols, releasing N_2_ as by-product.

## Main

**Fig. 1:**
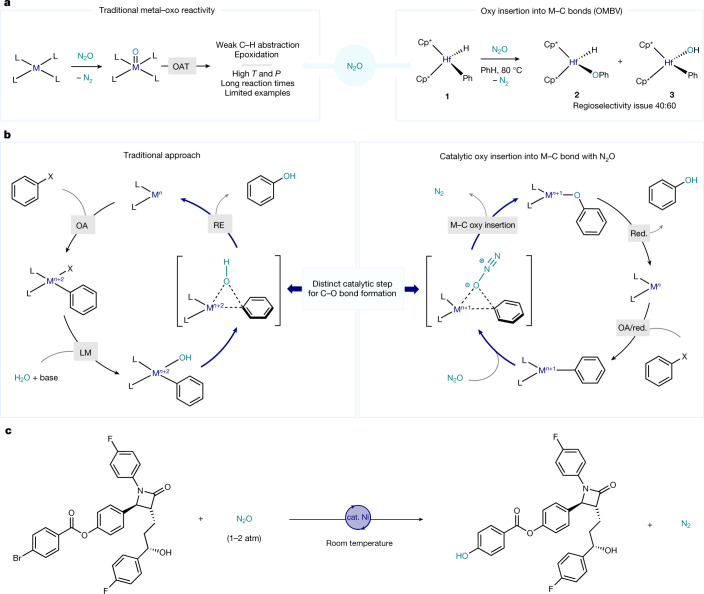
Relevance of N_2_O as a green oxidant in the synthesis of phenol derivatives. **a**, Reactivity of N_2_O and transition metals in the formation of C–O bonds. The right panel is based on the work described in ref. ^17^; OAT, oxygen atom transfer; Cp*, pentamethylcyclopentadienyl anion **b**, Comparison between traditional C(*sp*^2^)–O bond formation via reductive elimination (left panel) and through O insertion (right panel; this work) for the synthesis of phenols. Red., reduction.; OA, oxidative addition; RE, reductive elimination; LM, ligand metathesis;  **c**, Activation of N_2_O in a Ni reductive catalytic cross-electrophile phenol synthesis, (this work).

The increasing emission of greenhouse gases represents a global environmental threat, and strategies to address this issue have been the focus of intense research in recent times^1,2^. From the sustainability point of view, the development of chemical processes that extend beyond the traditional degradations and repurpose such gaseous by-products as useful synthons to produce valuable chemical feedstocks is highly desirable. Whereas the revalorization of CO_2_ or CH_4_ as carbon sources for organic synthesis through catalytic strategies has received a great deal of attention^12,13^, much less interest has been focused on the chemical transformation of another major contributor to the global warming: N_2_O. Governmental reports and recent scientific evidence both warn of the consequences that result from the increasing presence of this undervalued gas in the atmosphere^3–5^. N_2_O exhibits a global warming potential >300 times that of CO_2_, with a decomposition half-time in the atmosphere of >100 years^6^. Human activities have accelerated the emissions, with an estimated rate of increase for N_2_O of 2% per decade. Yet, a detailed analysis through the lens of sustainable synthesis presents a unique opportunity for N_2_O revalorization, as it represents an excellent O-atom source: it is readily available, non-toxic (laughing gas) and releases benign N_2_ as a by-product on O removal. Conversely, N_2_O is an inert gas, whose activation requires high temperatures (140–350 °C) and pressures (50–200 bar), resulting in limited applications as an oxidant for organic synthesis^7,8,14^. Yet, the structure of N_2_O has captivated chemists, who studied in detail coordination modes and the reactivity thereof in a plethora of metal complexes^9^. However, few reports focused on its activation towards the formation of C‒O bonds^10,15^ (arguably among the most valuable bonds in organic synthesis), as it would permit access to highly coveted alcohols, ethers, epoxides and so on. Still, these few examples rely on traditional metal–oxo reactivity, which requires high temperatures (100–200 °C) and pressures (10 bar)^11^ or long reaction times (1 ton per week)^16^ (Fig. 1a). In a groundbreaking report^17^, a fundamentally different outcome was observed: on exposure to a N_2_O atmosphere, the O atom could be inserted into a Hf‒Ph bond of complex **1** forging the desired Hf‒O‒Ph (**2**) with extrusion of N_2_. However, regioselectivity issues arose, as the O atom was competitively transferred to the hydride ligand, thus also producing a Hf‒O‒H complex (**3**). Mechanistic studies on the O insertion step into various M‒C(*sp*^2^) bonds using N_2_O, peroxides or oxygen suggest that an organometallic Baeyer–Villiger (OMBV)-type mechanism is operating, whereby the anionic carbon migrates to the coordinated O atom, forging the M‒O‒C bond^18–23^. On the basis of this reactivity, we aimed at unlocking the potential of N_2_O as an O-atom source in a fundamentally different catalytic synthesis of phenols. In the canonical transition-metal-catalysed phenol synthesis from aryl halides^24–28^, th‑e C(*sp*^2^)‒O bond-forming step proceeds through the well-established ligand exchange with a nucleophilic O source. On reductive elimination with the aryl group, the desired C(*sp*^2^)‒O bond is formed while the metal center is twofold-reduced (Fig. 1b, left). The source of O in these cases is usually H_2_O or a protic O-based nucleophile  in combination with a base that lead to the corresponding phenol^29^. In the alternative catalytic cycle proposed herein, the fundamental step for C(*sp*^2^)‒O bond formation capitalizes on the OMBV-type mechanism: on N_2_O coordination to the metal centre, the electrophilic O is eventually inserted into the M‒C bond, with concomitant formation of N_2_ (Fig. 1b, right). In contrast to the traditional synthesis of phenols, after C‒O bond formation the oxidation state of the metal centre remains intact, thus requiring an external reductant to close the cycle. To orchestrate this reductive process, we focused our attention on Ni and its demonstrated ability to manoeuvre between different oxidation states through single-electron transfer^30–32^. Here we demonstrate that a mechanistically guided approach for the activation of N_2_O with organometallic complexes results in the development of a mild and selective catalytic synthesis of high-value phenols from aryl halides using N_2_O as an electrophilic O source^33^. The mild conditions (25 °C and 1.5–2 atm) allow the accommodation of a variety of functional groups, including base-sensitive moieties, thus providing an orthogonal strategy to the current technologies (Fig. 1c).

**Fig. 2:**
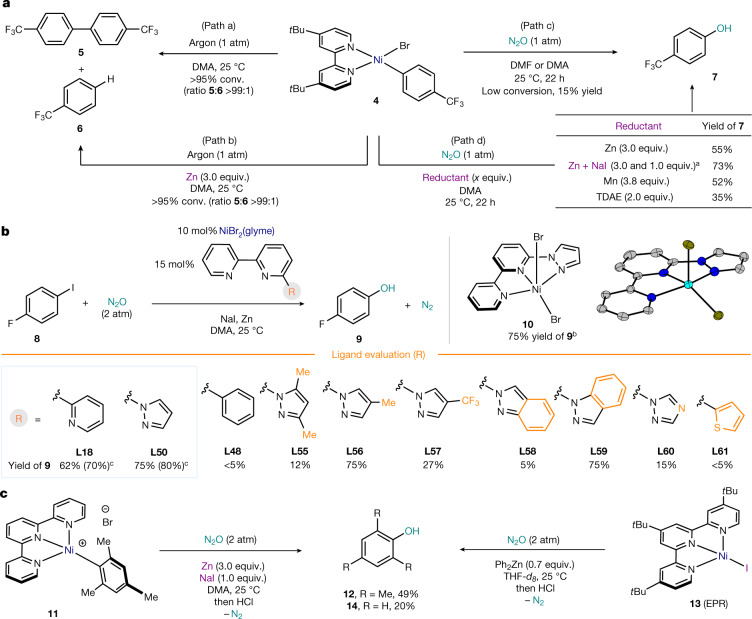
Initial discovery, ligand optimization and potential intermediates. **a**, Stoichiometric reactivity using bipyridine-supported Ni(II) oxidative addition complexes. conv., conversion; equiv., equivalents. **b**, Key electronic and structural features of the ligand in the catalytic synthesis of phenols from aryl halides and N_2_O. Details on the complete optimization of the reaction conditions can be found in the Supplementary Information. **c**, Reactivity studies of tridentate terpyridine-supported Ni(II) and Ni(I) complexes with N_2_O, pointing to the involvement of Ni(I)–C(*sp*^2^) species. Electron paramagnetic resonance (EPR) details and discussion for **13** can be found in the Supplementary Information. ^a^Using 2 atm of N_2_O_._^b^With 5 mol% extra **L50**. ^c^Yields using NiBr_2_(diglyme).

To investigate the feasibility of the M‒C(*sp*^2^) oxidation, we drew inspiration from previous work^17^, in which N_2_O was demonstrated to react with certain phosphine–Ni(II) complexes^34,35^. To this end, we synthesized the product of oxidative addition **4**, and studied its reactivity with N_2_O (Fig. 2a). As expected, **4** rapidly decomposes mainly towards homocoupling (**5**) when dissolved in DMA under argon, with only traces of protodemetallation (**6**) detected (path a). This reactivity is exacerbated by the presence of reducing agents such as Zn (path b). Yet, when the argon atmosphere is replaced by N_2_O, the bright red colour of the solution of **4** remains, thus pointing towards a slower decomposition rate. After acidic workup, a 15% yield of phenol **7** is observed. However, when the same reaction is performed in the presence of a reducing agent, substantially higher yields of **7** were observed, with a 73% yield obtained when a combination of Zn and NaI was used (path d). These results point to the feasibility of developing a reductive catalytic protocol based on Ni catalysis using aryl halide precursors. From our extensive ligand survey (see Supplementary Information), it was evident that tridentate nitrogenated ligands with the general pattern of 2-substituted bipyridine were crucial to obtain catalytic activity, with terpyridine (**L18**) and 6-pyrazolyl-2,2'-bipyridine (**L50**) affording the highest yields of **9** (Fig. 2b and Supplementary Information). Analysis of the R group revealed three key features of the ligand for catalytic activity: replacing the N atom with C‒H or S prevents catalytic activity (**L48** and **L61**); steric encumbrance next to the N of the pyrazole unit inhibits productive catalysis (**L55** and **L58**); and electron-deficient substituents on the pyrazole markedly reduce the yield of phenol. To confirm Ni–ligand ligation in the reaction mixture, complex **10** was prepared and structurally characterized. A 75% yield of **9** was obtained using **10** as a catalyst, thus confirming that the pre-ligated complex is catalytically competent. **L50** represents a new ligand platform in Ni catalysis, with virtually no examples. As Fig. 2a, b suggests that formal Ni(I)‒C(*sp*^2^) species might be involved, we prepared terpyridine–Ni derivatives such as **11** (Fig. 2c), as greater stability of the corresponding (terpy)Ni(I)‒Ar has been noted^36–38^. As for **4**, reaction of **11** in the absence of reducing agent led to no phenol formation. Yet, despite the presence of two Me groups in *ortho*, reaction performed under N_2_O in the presence of Zn and NaI afforded the desired mesitol (**12**) in 49% yield on acidic workup. To further confirm the involvement of a formal Ni(I)‒C(*sp*^2^) in the tridentate system, the (*t*Bu-terpy)Ni(I)‒I (**13**) was reacted with Ph_2_Zn under N_2_O. Despite the reported instability of (terpy)Ni(I)‒Ph (ref. ^37^), a 20% yield of phenol (**14**) was obtained. These findings suggest that reduction of Ni(II) species to formal Ni(I) and the presence of iodide salts in the system are of importance to forge the desired C(*sp*^2^)‒O bond. Mechanistic investigations on M‒Ar oxy insertions for late transition metals (Pd, Ni and Fe) reveal that subtle changes in the ligand environment also lead to differences between the metal‒oxo/oxyl or concerted Baeyer-Villiger pathways for Ar‒O bond formation^18–23^. In this case, we suggest that in the continuum between the two extreme possibilities offered in the OMBV reaction, the oxy insertion of N_2_O in a *d*^9^ complex lies towards the formation of the M‒O bond and N_2_, before Ar migration^39^.

**Fig. 3:**
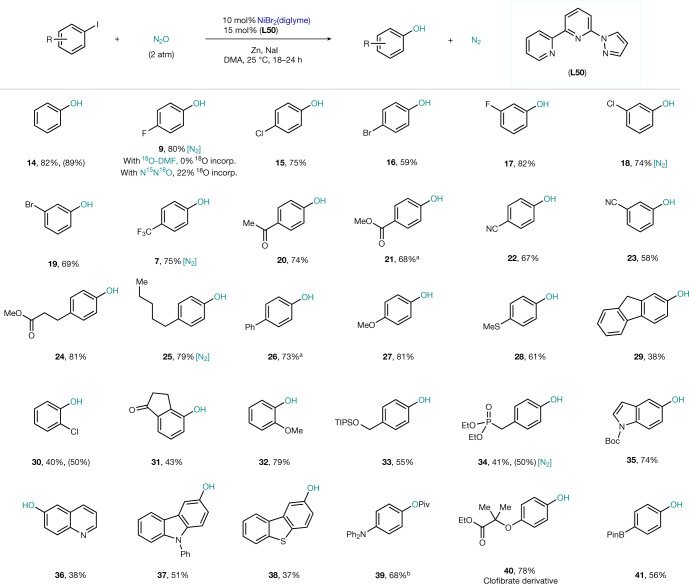
Revalorization of N_2_O as O source in the catalytic synthesis of phenols. Scope of aryl iodides. [N_2_], N_2_ detected by a gas chromatography–thermal conductivity detector at the end of the reaction. All yields are of isolated pure material. Yield in brackets: ^1^H NMR yield calculated using dibromomethane as an internal standard. incorp., incorporated. See the Supplementary Information for details of the procedures. ^a^Use of **L18** as the ligand instead of **L50**. ^b^Owing to the rapid oxidation of the free alcohol, **39** was obtained after quenching with Piv_2_O.

With the optimized catalytic system in hand, a preliminary scope of the aryl halide counterpart was interrogated. As shown in Fig. 3, aryl iodides bearing other halogens in both *para* (**9**, 15 and **16**) and *meta* (**17–19**) positions smoothly afforded the corresponding phenol in excellent yields. The presence of electron-withdrawing groups such as CF_3_ (**7**), ketone (**20**), ester (**21** and **24**) or nitrile (**22–23**) posed no difficulty for the C‒O bond formation. Electron-donating substituents such as alkyl (**25**), aryl (**26**), or even methoxy and thiomethyl (**27** and **28**) delivered phenol in good yields. Moreover, a fluorene derivative (**29**) featuring benzylic C‒H bonds was also amenable for phenol synthesis, albeit in 38% yield. A classical feature of reductive couplings is that steric hindrance at the *ortho* position can impede reactivity. Indeed, C‒O bond formation from indanone (**31**) and 1-chloro-2-iodobenzene (**30**) derivatives afforded slightly diminished yields. In contrast to **30**, **32** was obtained in 79% yield, illustrating a possible beneficial chelating effect of the *ortho* OMe and the Ni centre. A silylated benzylic alcohol (**33**) or a diethylphosphonate (**34**) were also tolerated in this protocol. Heterocycles such as indole (**35**), quinoline (**36**), carbazole (**37**) or dibenzothiophene (**38**) also afforded the corresponding phenol in good yields. Substrates prone to rapid oxidation after C‒O bond formation could be further functionalized in situ, as exemplified by the 68% yield obtained for the pivaloyl derivative **39**. An iodide derivative of the biologically active agent clofibrate could be converted to phenol **40** in 78% yield despite the presence of a tertiary *α*-oxy ester. This protocol does not require the use of nucleophilic alkoxy surrogates, and hence base-sensitive functionalities such as esters or sensitive amides, can be tolerated. An example of this chemoselectivity is observed in the derivatization of a substrate containing pinacol boronate. In this case, phenol **41** was still obtained in 56% yield, thus providing an orthogonal tool to classical oxidation. The observation of 7% yield of sulfoxide in the reaction of **28** (ref. ^40^), and the low yield obtained for fluorenol **29**, suggest that the oxy-insertion step lies towards the oxo/oxyl–pathway in the continuum postulated for OMBV-type reactions. N_2_ was detected using a gas chromatography–thermal conductivity detector in the headspace after the reaction had finished for **7**, **9**, **18**, **25** and **34** (Fig. 3). When the oxygen on the solvent was labelled ([^18^O]DMF, 25% ^18^O), no ^18^O was incorporated in **9**. On the other hand, when N^15^N^18^O was used (ca. 23% ^18^O), 22% ± 1 of the O in **9** was labelled (Supplementary Information). Together, these data point to N_2_O as the source of O.

**Fig. 4:**
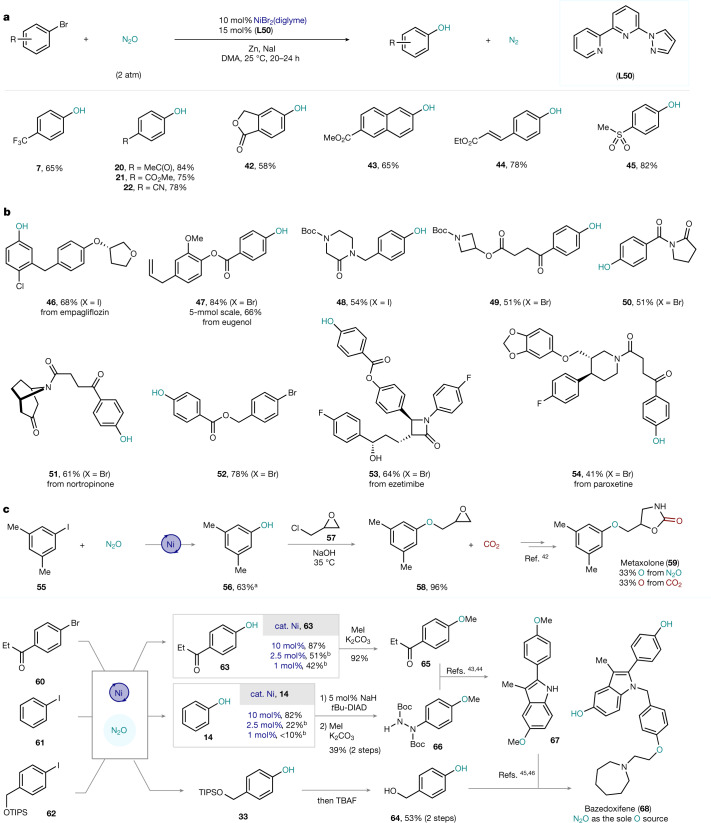
Scope and applications of the catalytic protocol. **a**, Catalytic synthesis of phenols from aryl bromides using N_2_O. **b**, Exploring the insertion of O atoms into densely functionalized aryl halides (starting from Ar–X). **c**, Using greenhouse gases as building blocks for the synthesis of biologically relevant molecules. All yields are of isolated pure material. See Supplementary Information for experimental details. ^a^Use of **L18** as the ligand instead of **L50**. ^b^Yields determined by ^1^H NMR using 1,3,5-trimethoxybenzene as an internal standard.

The same optimized reaction conditions for aryl iodides permitted C‒O bond formation of more accessible and commercially available aryl bromides. Yet, electron-withdrawing substituents were required to allow C(*sp*^2^)‒Br cleavage to occur. In this sense, phenols bearing CF_3_ (**7**), Ac (**20**) and CN (**22**) in the *para* position, as well as paraben (**21**), could be obtained in high yields (Fig. 4a). Medicinally relevant phthalide was also smoothly converted to the phenol (**42**), thus providing a method to synthesize this building block with three fewer steps compared with the reported method^41^. Phenols derived from π-extended or conjugated systems such as naphthoate **43** or cinnamate **44** were also obtained in 65% and 78% yields, respectively. In contrast to current light-mediated processes, no isomerization of the double bond in **44** was observed^30^. Finally, another base-sensitive group such as the aryl methyl sulfone could be tolerated and the corresponding phenol **45** was obtained in 82% yield. Heterocyclic bromides are not compatible with the current protocol.

Complex aryl halides functionalized with sensitive moieties were then tested. For example, an empagliflozin derivative, which contains a plethora of weak C‒H bonds prone to HAT, was smoothly converted to the corresponding phenol (**46**) in excellent yield (Fig. 4b). An ester derivative of the natural product eugenol afforded the desired phenol (**47**) in an 84% yield, highlighting the high chemoselectivity of this process over alternative oxidation through metal–oxo pathways. Despite the triphasic nature of the protocol, synthesis of **47** could be scaled up to 5 mmol with only a slight reduction in the yield (66%). Substrates containing saturated N-heterocycles such as piperazinone **48**, azetidine **49**, pyrrolidinone (aniracetam intermediate) **50** and nortropinone derivative **51** are well tolerated. The requirement of an electron-withdrawing group to activate the aryl bromide can be turned into a synthetic advantage, thus permitting regioselective control on the activated aryl bromide (**52**, 78%). Finally, a derivative ezetimibe, the drug used to treat high blood cholesterol, could be smoothly converted into the corresponding phenol (**53**), without altering the chiral and unprotected secondary alcohol, the ester and the strained β-lactam. Similar chemoselectivity can be observed in the conversion of paroxetine derivative **54**. Finally, Fig. 4c illustrates a proof-of-concept of the potential for the revalorization of greenhouse gases for organic synthesis. It is now possible to combine N_2_O and CO_2_ revalorization strategies and obtain metaxolone (**59**), in which 66% of the oxygen atoms originate from waste gaseous feedstock^42^. A more striking example is illustrated in the synthesis of bazedoxifene (**68**), a drug candidate against breast and pancreatic cancer. The three phenolic building blocks could be rapidly obtained from the parent halides in good yields (**64**–**66**). Subsequent Fischer-indole synthesis allows access to indole **67**, enabling the synthesis of bazedoxifene (**68**) with all O atoms originating from N_2_O (refs. ^43–46^). Whereas a 42% yield could be obtained for precursor **63** with 1 mol% catalyst loading, a <10% yield of **14** was observed with the same catalyst loading, with substantial protodehalogenation of the parent iodide **61**, which highlights the subtle differences between aryl iodides and aryl bromides in this system.

## Conclusions

Through a distinct fundamental organometallic step, a catalytic protocol for the revalorization of N_2_O as a green, mild and chemoselective O-atom insertion reagent for organic synthesis has been unlocked. Mechanistically guided insights into the reactivity of N_2_O with Ni complexes point to formally low-valent Ni(I)‒aryl permitting the O insertion in an efficient manner. The inert N_2_O molecule succumbs to activation under mild conditions for the selective synthesis of phenols from aryl halides. The catalytic system features an electronically asymmetric tridentate bipyridine-based ligand (**L50**) for the Ni centre, which enables selective C‒O bond formation. The reported conditions are simple and robust, allowing phenol formation in densely functionalized molecules. Whereas other catalytic protocols capitalize on nucleophilic HO^‒^ counterparts, this method represents a unique example of catalytic C‒O bond formation with an electrophilic O-atom source, which in turn can accommodate base-sensitive functionalities. Furthermore, this protocol demonstrates the feasibility of accessing relevant drugs for which N_2_O is the sole source of oxygen atoms.

## Online content

Any methods, additional references, Nature Research reporting summaries, source data, extended data, supplementary information, acknowledgements, peer review information; details of author contributions and competing interests; and statements of data and code availability are available at 10.1038/s41586-022-04516-4.

## Supplementary information

Supplementary Information This file contains general methods, chemical compounds, figures and references.

## Data Availability

Details on the procedures, optimization, characterization and mechanisms, including spectra of new compounds and compounds made using the reported method, are available in the Supplementary Information. Crystallographic data for compound **10** can be obtained free of charge from www.ccdc.cam.ac.uk under reference number 2114695.
