# Author Correction: Catalytic synthesis of phenols with nitrous oxide

**DOI:** 10.1038/s41586-022-05064-7

**Published:** 2022-07-21

**Authors:** Franck Le Vaillant, Ana Mateos Calbet, Silvia González-Pelayo, Edward J. Reijerse, Shengyang Ni, Julia Busch, Josep Cornella

**Affiliations:** 1 Max-Planck-Institut für Kohlenforschung, Mülheim an der Ruhr, Germany; 2 Max-Planck-Institut für Chemische Energiekonversion, Mülheim an der Ruhr, Germany

**Keywords:** Homogeneous catalysis, Synthetic chemistry methodology

Correction to: *Nature* 10.1038/s41586-022-04516-4 Published online 27 April 2022

In the version of this article initially published online, there was a typographical error in the description of the labelling experiments. The employed in situ-formed N^15^N^18^O is expected to be approximately 23% labelled, instead of the originally written 45%. This value remains correctly described in the Supplementary Information.

