# Supplementary material for: Catalytic synthesis of phenols with nitrous oxide
Source: Nature. 2022 Apr 27;604(7907):677–83. doi: 10.1038/s41586-022-04516-4 (PMC9046086; doi:10.1038/s41586-022-04516-4)
Supplement: Supplementary file 1 — This file contains general methods, chemical compounds, figures and references. [file 41586_2022_4516_MOESM1_ESM.pdf]

nature portfolio

https://doi.org/10.1038/s41586-022-04516-4

Supplementary information

Catalytic synthesis of phenols with nitrous oxide

In the format provided by the
authors and unedited

Supplementary information

Catalytic synthesis of phenols with nitrous oxide

Franck Le Vaillant, Ana Mateos Calbet†, Silvia González-Pelayo†, Edward J. Reijerse, Shengyang Ni, Julia Busch, Josep Cornella*

†These authors contributed equally to this work.
*Corresponding author. Email: cornella@kofo.mpg.de

Max-Planck-Institut für Kohlenforschung, Kaiser-Wilhelm-Platz 1,

Max-Planck-Institut für Chemische Energiekonversion, Stiftstrasse 34–36

Mülheim an der Ruhr, 45470, Germany

Table of Contents

1. General Methods ... 3

2. Synthesis of ligands ... 4

2.1. Preparation of ligand bipy-pyr (**L50**) ... 6

2.2. Preparation of new ligands ... 7

3. Synthesis of starting materials ... 14

4. Stoichiometric reactivity ... 23

4.1. Reaction discovery and control experiments ... 23

4.2. Color evolution ... 28

4.3 Optimization and screening of other reducing agents ... 29

4.4. Reactions with tridentate [LNi(II)–Ar]X complexes ... 35

5. Optimization of catalytic oxidation of aryl iodides using N_2O ... 48

5.1 Optimization of the reaction ... 48

5.2. Comparison of product distribution with several ligands ... 59

5.3. Fine-tuning the conditions ... 60

5.4. Comparison of terpy and bipy-pyr ligands ... 62

5.5. Synthesis, characterization and reactivity of (bipy-pyr)$NiBr_2$ (**10**) ... 63

5.6. Control experiments ... 66

5.7. Origin of O-atom ... 66

6. Scope of the catalytic oxidation of aryl halides using N_2O ... 68

6.1. Scope of aryl iodides ... 69

6.2. Scope of aryl bromides ... 84

6.3. Scope of complex molecules ... 86

6.4. Current limitations of the catalytic synthesis of phenols using N_2O and aryl halides ... 94

7. Applications ... 96

7.1. Synthesis of metaxalone intermediate ... 96

7.2. Synthesis of phenol precursors towards bazedoxifene ... 97

8. Labelling experiment ... 102

8.1. Synthesis of ^{18}O-dimethylformamide ... 102

8.2. Reaction using ^{18}O-dimethylformamide as solvent ... 103

8.3. Reaction using in situ formed ^{18}O-N_2O ... 105

9. Headspace analysis ... 108

10. X-ray crystal structure analysis of **10** ... 115

11. Spectra of new compounds ... 124

12. References ... 194

1. General Methods

Instruments

GC-MS (FID): GC-MS-QP2010 equipped (Shimadzu Europe Analytical Instruments). ESI-MS: ESQ 3000 (Bruker). Accurate mass determinations: Bruker APEX III FT-MS (7 T magnet) or MAT 95 (Finnigan). GC-TCD measurements were performed on Agilent Technologies GC 7890B with a 30 m HP-Plot 5 Å Molsieves column. Melting points were measured with an EZ-Melt Automated Melting Point Apparatus from Standford Research Systems. NMR spectra were recorded using a Bruker AVIIIHD 300 MHz, Bruker AVIIIHD 400 MHZ, Bruker AVIII 500 MHz or Bruker AVneo 600 MHz NMR spectrometer. The chemical shifts (δ) are given in ppm and were measured relative to solvent residual peak as an internal standard. For ^{1}H NMR: $CDCl_3$, δ 7.26; CD_3CN, δ 1.94; $CD_3(SO)$, δ 2.50; CD_2Cl_2, δ 5.32; THF-*d*$_8$, δ 1.72. For ^{13}C NMR: $CDCl_3$, δ 77.16; CD_3CN, δ 1.32; $CD_3(SO)$, δ 39.52; CD_2Cl_2, δ 53.84; THF-*d*$_8$, δ 67.21. The data is being reported as (s = singlet, d = doublet, t = triplet, q = quartet, quint = quintet, m = multiplet or unresolved br s = broad signal, coupling constant(s) in Hz, integration, interpretation). X-Band EPR spectra were recorded on a Bruker Elexsys E500 CW X-band spectrometer. The sample tubes of 5-mm outer diameter were placed in a Bruker ER4116DM dual mode resonator. Low-temperature measurements were obtained using an Oxford ESR 900 helium flow cryostat (3–300 K) and an Oxford Mercury Temperature controller. Q-band experiments were performed on a Bruker ELEXYS E580 pulse EPR spectrometer with a SuperX-FT microwave bridge in combination with a home-built pulse Q-band accessory and a home-built Q-band resonator described earlier.[47] Cryogenic temperatures were obtained using an Oxford CF935 Helium flow cryostat. Q-band EPR spectra were recorded in pulsed echo or Free Induction Decay (FID) amplitude detection mode. By applying a pseudomodulation transformation on the absorption mode spectra, a first derivative spectrum is obtained compatible with CW-EPR.[48]

Chemicals

Unless otherwise stated, all manipulations were performed using Schlenk techniques under dry argon in heatgun-dried glassware. Anhydrous solvents were distilled from appropriate drying agents and were transferred under Argon: THF, Et_2O (Mg/anthracene), CH_2Cl_2 (CaH_2), CH_3CN (CaH_2), hexane, toluene (Na/K), benzene-*d*$_6$ (MS), CD_2Cl_2 (MS), CD_3CN (MS). DMF-*d*$_7$, THF-*d*$_8$ were purchased from Eurisotop, degassed by repeated freeze-pump-thaw cycles, distilled from the proper drying agents, and stored over 4 Å molecular sieves. 4 Å molecular sieves were activated at 200 °C under high vacuum ($1 \times 10{-}4$ bar) for 3 days. Unless otherwise noted, all reagents were obtained from commercial suppliers and used without further purification. N_2O was provided by Air Liquide (Distickstoffmonoxid UHP 5.0) containing less than 5 ppm N_2, 1 ppm H_2O, and 1 ppm air and O_2. 90%-^{18}O- and 98%-^{15}N-Labelled $NaNO_2$, anhydrous DMA (250 mL, 99.8%) and NaI (anhydrous, free-flowing, Redi-Dri™, ReagentPlus®, ≥99%) were purchased from Sigma-Aldrich, stored directly in the glovebox, and use as received. Zinc powder (-140+325 mesh, 99.9% (metal basis)) was purchased from Alfa Aesar, stored directly in the glovebox, and used as received. Ni complexes are prepared according to literature procedure: $Ni(COD)_2$,[49] [(*t*Bu-bipy)Ni(*p*-CF_3-Ph)]Br,[50] *trans*-[$(PPh_3)_2$Ni(Mes)Br],[51] [(terpy)Ni(Mes)Br],[52] (terpy)Ni–Br,[53] (*t*Bu-terpy)Ni–I.[54]

2. Synthesis of ligands

Below is a list of 70 ligands tested in catalysis.

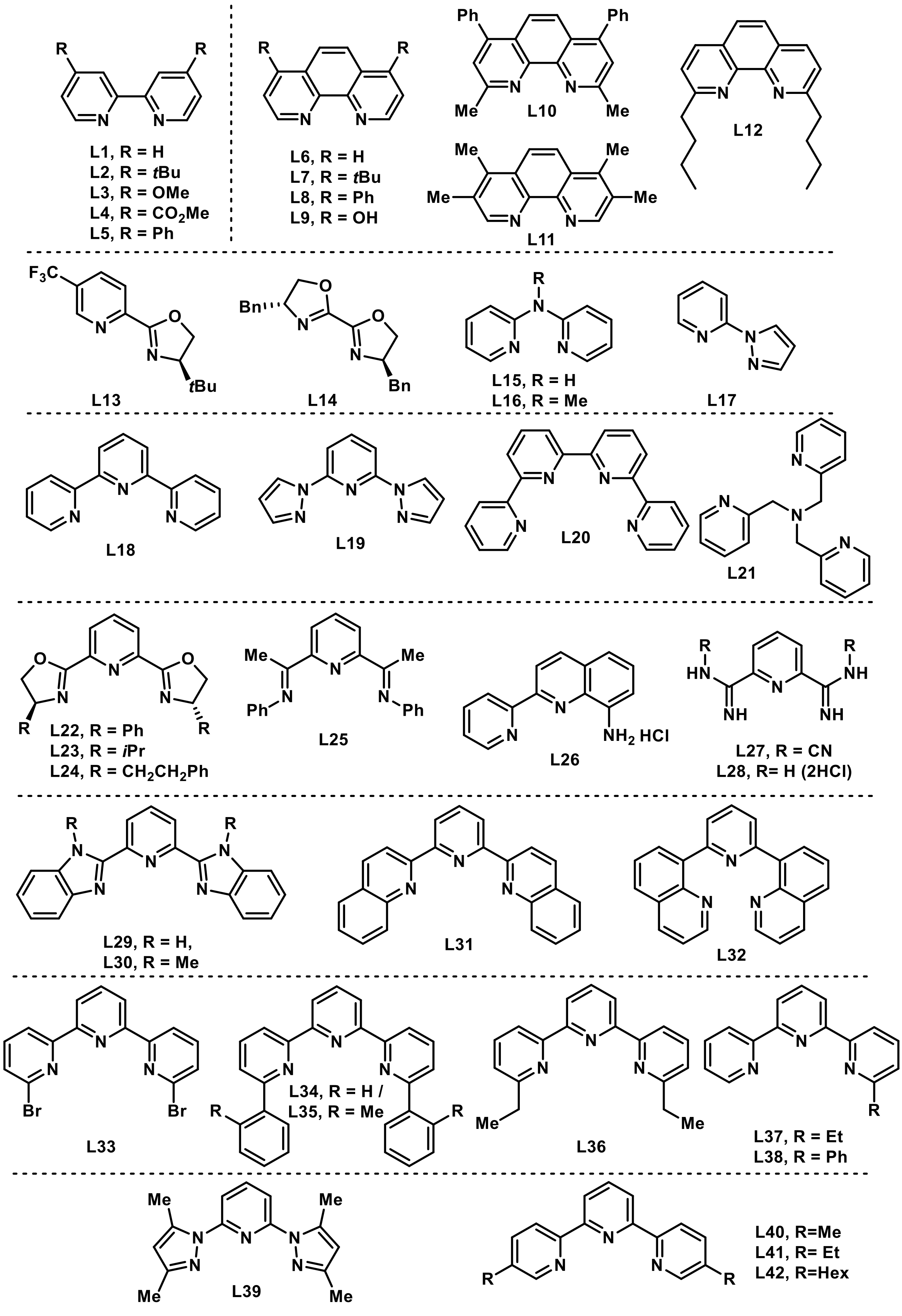

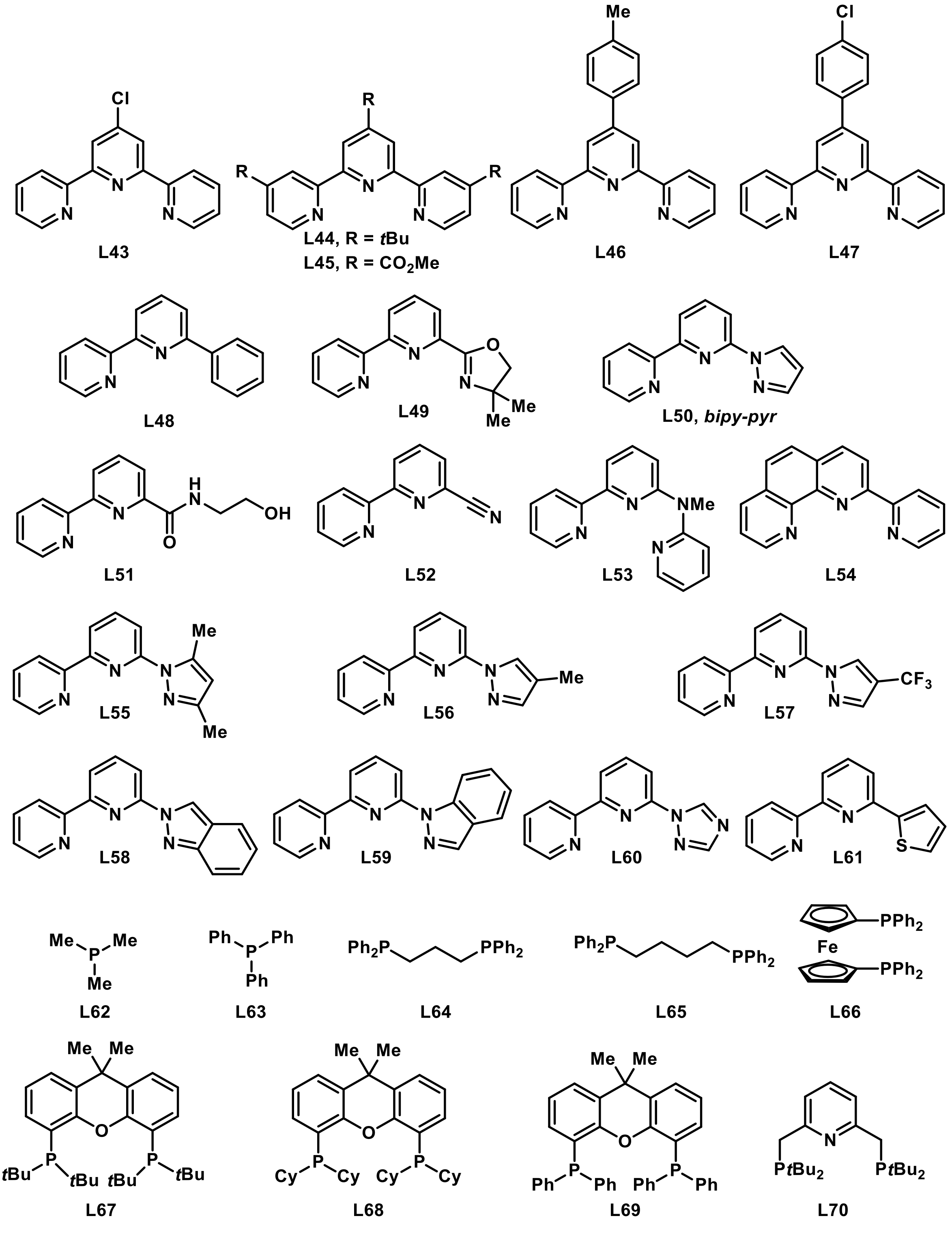

Fig. S1. List of ligands tested in the optimization of the catalytic synthesis of phenols with nitrous oxide and aryl iodides.

The following ligands are commercially available and used as received: **L1 – L14, L18, L21 – L24, L26-28, L33, L43-47, L52, L62-70.** The following ligands are known and they have been prepared in house following literature procedures. **L12, L19, L20, L25, L29 – L32, L34 – L35,**

L38 – L41, L49, L53 – L56, L60 – L61. The synthesis of the following new ligands (**L36, L37, L42, L49, L51, L57 – L59**) will be described after the synthesis of bipy-pyr (**L50**).

2.1. Preparation of ligand bipy-pyr (L50)

The synthesis of bipy-pyr (**L50**) is already described in the literature;[55] however, we report here a modified procedure with cheaper starting materials and reliable reaction conditions to access high-purity ligand.

6-(1*H*-Pyrazol-1-yl)-2,2'-bipyridine (bipy-pyr, L50)

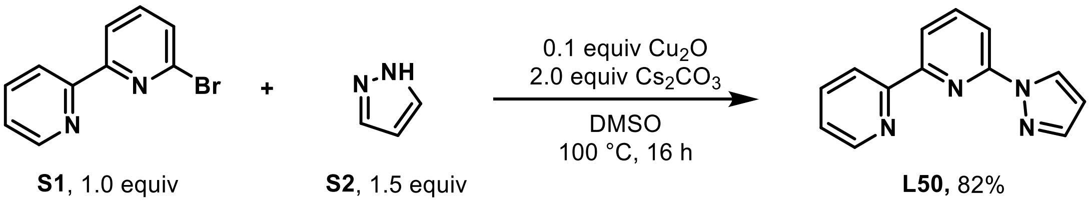

Following a slightly modified procedure,[56] 6-bromo-2,2'-bipyridine (**S1**, 626 mg, 2.66 mmol, 1.0 equiv), pyrazole (**S2**, 272 mg, 3.99 mmol, 1.5 equiv) copper(I) oxide (38 mg, 0.27 mmol, 0.1 equiv), cesium carbonate (1.74 g, 5.33 mmol, 2.0 equiv), and DMSO (3 mL) were added to a heatgun-dried pressure Schlenk flask, and heated at 100 °C for 16 h. Upon cooling to room temperature, DCM (5 mL) was added, and the mixture was filtrated through a celite-pad. The recovered filtrate was evaporated under reduced pressure. Column chromatography over silica gel afforded 485 mg (2.18 mmol, 82%) of **L50** as a white solid.

^{1}H NMR (400 MHz, DMSO-*d_6*) δ 8.91 (d, *J* = 2.6 Hz, 1H, ArH), 8.72 (dd, *J* = 3.4, 1.2 Hz, 1H, ArH), 8.58 (d, *J* = 7.9 Hz, 1H, ArH), 8.32 (d, *J* = 7.6 Hz, 1H, ArH), 8.13 (t, *J* = 7.9 Hz, 1H, ArH), 8.03 – 7.95 (m, 2H, ArH), 7.86 (d, *J* = 1.7 Hz, 1H, ArH), 7.50 (ddd, *J* = 7.5, 4.7, 1.2 Hz, 1H, ArH), 6.63 (app dd, 1H, ArH).

^{13}C NMR (101 MHz, DMSO-*d_6*) δ 154.2, 154.1, 150.5, 149.4, 142.4, 140.7, 137.5, 127.4, 124.6, 121.0, 118.2, 112.1, 108.3.

The values of the ^{1}H NMR spectrum are in accordance with reported literature data.[55]

For convenience, we also report the data in $CDCl_3$:

^{1}H NMR (300 MHz, $CDCl_3$) δ 8.74 (dd, *J* = 2.6, 0.8 Hz, 1H, Ar*H*), 8.70 (ddd, *J* = 4.8, 1.8, 1.0 Hz, 1H, Ar*H*), 8.47 (dt, *J* = 8.0, 1.1 Hz, 1H, Ar*H*), 8.33 (dd, *J* = 7.4, 1.2 Hz, 1H, Ar*H*), 8.05 – 7.91 (m, 2H, Ar*H*), 7.85 (ddd, *J* = 7.9, 7.5, 1.8 Hz, 1H, Ar*H*), 7.77 (dd, *J* = 1.7, 0.7 Hz, 1H, Ar*H*), 7.34 (ddd, *J* = 7.5, 4.8, 1.2 Hz, 1H, Ar*H*), 6.50 (dd, *J* = 2.6, 1.7 Hz, 1H, Ar*H*).

^{13}C NMR (101 MHz, $CDCl_3$) δ 154.2, 154.1, 150.5, 149.4, 142.4, 140.7, 137.5, 127.4, 124.6, 121.0, 118.2, 112.2, 108.3.

2.2. Preparation of new ligands

The following ligands are new and their synthesis is detailed below.

6,6''-Diethyl-2,2':6',2''-terpyridine (L36)

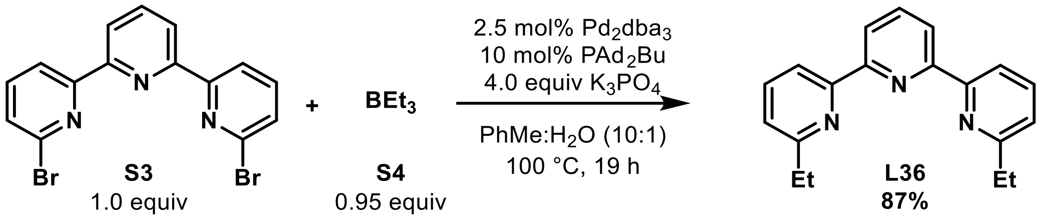

Following a modified reported procedure,[57] to a flask charged with 6,6''-terpydibromide (**S3**, 125 mg, 0.320 mmol, 1.0 equiv), triethylborane in THF (**S4**, 4.4 mL, 0.30 mmol, 0.95 equiv), $Pd_2(dba)_3$.DCM (7.3 mg, 0.0080 mmol, 0.025 equiv), *n*BuAd$_2$P (11.5 mg, 0.032 mmol, 0.10 equiv), K_3PO_4 (271 mg, 1.28 mmol, 4.0 equiv) and toluene (10 volume, 4.0 mL) and degassed water (1 volume, 0.4 mL) under argon, the reaction mixture were heated to 100 °C under argon and stirred for 19 h. The reaction mixture was cooled to room temperature and added water (5 mL) and extracted with EtOAc (10 mL), cut the layer and back extract the aqueous layer once with EtOAc (10 mL). The combined organic layers were dried over $MgSO_4$, concentrate. Purification via column chromatography over silica gel (EtOAc/Hex 2:98 to 1:9) yielded 80 mg (0.28 mmol, 87%) of 6,6''-diethyl-2,2':6',2''-terpyridine (**L36**) as an off-white solid.

M.p.: 91.9 – 94.2 °C.

^{1}H NMR (400 MHz, $CDCl_3$) δ 8.50 (d, *J* = 7.8 Hz, 2H, Ar*H*), 8.43 (d, *J* = 7.7 Hz, 2H, Ar*H*), 7.93 (t, *J* = 7.8 Hz, 1H, Ar*H*), 7.76 (t, *J* = 7.7 Hz, 2H, Ar*H*), 7.19 (d, *J* = 7.6 Hz, 2H, Ar*H*), 2.92 (q, *J* = 7.6 Hz, 4H, C*H*$_2$CH$_3$), 1.40 (t, *J* = 7.6 Hz, 6H, CH$_2$C*H*$_3$).

^{13}C NMR (101 MHz, $CDCl_3$) δ 163.0, 155.8, 155.7, 137.8, 137.2, 122.2, 121.0, 118.5, 31.6, 14.0.

HRMS (EI): calcd for $C_{19}H_{19}N_3^+$ $[M]^+$ 289.15735; found 289.15749.

6-Ethyl-2,2':6',2''-terpyridine (L37)

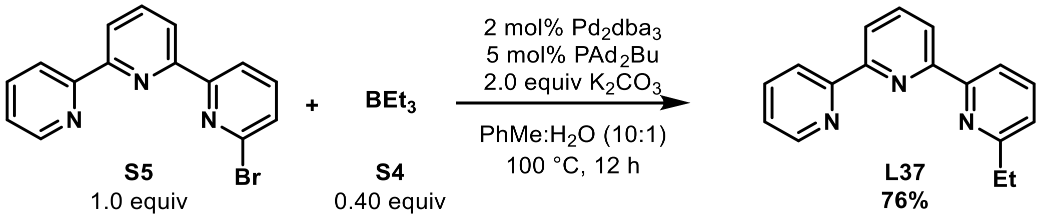

Following a reported procedure,[57] 6-bromo-2,2':6',2"-terpyridine (**S5**, 100 mg, 320 µmol, 1.0 equiv), tris(dibenzylideneacetone)dipalladium(0) (5 mg, 5 µmol, 2 mol%), di(1-adamantyl)-*n*-butylphosphine (7.0 mg, 20 µmol, 5 mol%), and potassium carbonate (106 mg, 765 µmol, 2.0 equiv) were suspended in a mixture of toluene (3.0 mL) and degassed water (0.3 mL). Triethylborane (**S4**, 1 M in THF, 0.15 mL, 0.15 mmol, 0.40 equiv) was added and the reaction mixture was heated at 100 °C for 12 h. Upon cooling to room temperature, water was added, and the mixture was extracted with EtOAc (2 × 50 mL). The combined organic layers were dried over Na_2SO_4, filtrated, and evaporated. Purification via column chromatography over silica gel (EtOAc/Hex 2:98 to 1:9) yielded 62 mg (0.24 mmol, 74%) of 6-ethyl-2,2':6',2"-terpyridine (**L37**) as an off-white solid.

M.p.: 44 °C.

^{1}H NMR (400 MHz, CDCl$_3$) δ 8.70 (ddd, *J* = 4.8, 1.8, 0.9 Hz, 1H, Ar*H*), 8.63 (dt, *J* = 8.0, 1.1 Hz, 1H, Ar*H*), 8.53 (dd, *J* = 7.8, 1.1 Hz, 1H, Ar*H*), 8.43 (m, 2H, Ar*H*), 7.95 (t, *J* = 7.8 Hz, 1H, Ar*H*), 7.86 (td, *J* = 7.7, 1.8 Hz, 1H, Ar*H*), 7.76 (t, *J* = 7.7 Hz, 1H, Ar*H*), 7.33 (ddd, *J* = 7.5, 4.8, 1.2 Hz, 1H, Ar*H*), 7.20 (dd, *J* = 7.6, 1.0 Hz, 1H, Ar*H*), 2.93 (q, *J* = 7.6 Hz, 2H, CH_2CH_3), 1.40 (t, *J* = 7.6 Hz, 3H, CH_2CH_3).

^{13}C NMR (101 MHz, CDCl$_3$) δ 163.0, 156.5, 155.9, 155.6, 155.3, 149.2, 137.9, 137.2, 137.0, 123.8, 122.3, 121.4, 121.3, 120.9, 118.4, 31.6, 14.0.

HRMS (EI): calcd for $C_{17}H_{15}N_3^+$ $[M]^+$ 261.12605; found 261.12630.

2-Methyleneoctanal (S6)

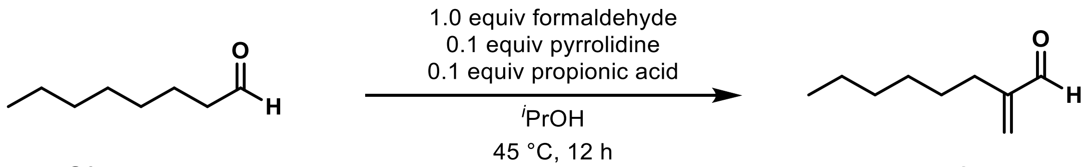

S6, 1.0 equiv **S7**, 43%

Following a reported procedure,[58] pyrrolidine (84 µL, 1.0 mmol, 0.1 equiv) and propionic acid (75 µL, 1.0 mmol, 0.1 equiv) were added to a mixture of aqueous formaldehyde solution (37%, 745 µL, 10.0 mmol, 1.0 equiv) and octanal (**S6**, 1.56 mL, 10.0 mmol, 1.0 equiv) in isopropyl alcohol (1 mL). The reaction mixture was heated at 45 °C for 12 h. Upon cooling to room temperature, $NaHCO_3$ was added. After extraction with DCM (3 × 50 mL), the combined organic layers were washed with brine. They were dried over Na_2SO_4, filtrated, and evaporated under reduced pressure. Filtration through a silica pad with Et_2O and evaporation under reduced pressure gave 602 mg (4.29 mmol, 43%) of **S7** as a colorless oil.

^{1}H NMR (400 MHz, CDCl$_3$) δ 9.53 (s, 1H, C*H*O), 6.23 (t, *J* = 1.2 Hz, 1H, C=C*H$_2$*), 5.97 (s, 1H, C=C*H$_2$*), 2.23 (t, *J* = 7.7 Hz, 2H, C*H$_2$C*(=CH$_2$)CHO), 1.5 – 1.2 (br m, 8H, (C*H$_2$*)$_4$CH$_3$), 0.90 – 0.84 (m, 3H, C*H$_3$*).

^{13}C NMR (101 MHz, CDCl$_3$) δ 194.9, 150.7, 134.0, 31.7, 29.1, 27.9, 27.9, 22.7, 14.2.

The values of the NMR spectra are in accordance with reported literature data.[58]

5,5’’-Dihexyl-2,2’:6’,2’’-terpyridine (L42)

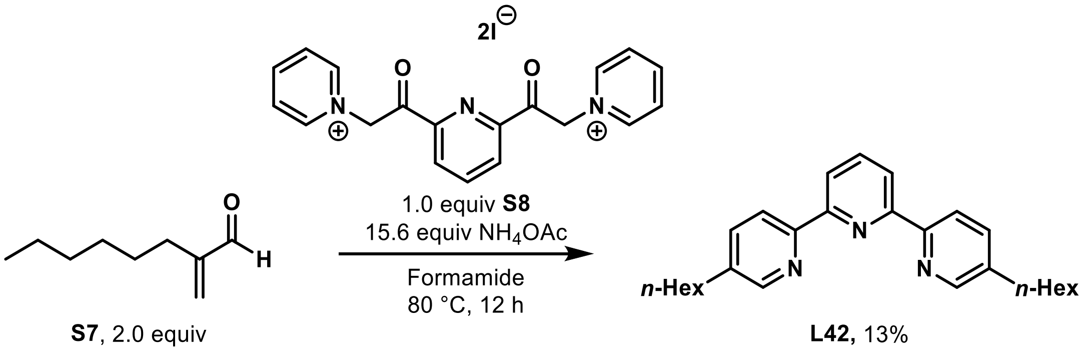

Following a slightly modified procedure,[59] ammonium acetate (1.20 g, 15.6 mmol, 15.6 equiv) and 2-methyleneoctanal (**S7**, 363 μL, 2.00 mmol, 2.0 equiv) were added to a solution 2,6-bis(pyridinioacetyl)pyridine diiodide (**S8**), 573 mg, 1.00 mmol, 1.0 equiv) in formamide (6 mL). The reaction mixture was heated at 80 °C for 12 h, and upon cooling to room temperature, water was added. After extraction with DCM (3 × 50 mL), the combined organic layers were dried over Na_2SO_4, filtrated, and evaporated under reduced pressure. Purification *via* column chromatography over silica gel (EtOAc/Hex 2:98 to 1:9) yielded 56 mg (0.13 mmol, 13%) of **L42** as a white solid.

Rf (EtOAc/Hex (1:9)) 0.17.

M.p.: 78 °C.

^{1}H NMR (400 MHz, CDCl$_3$) δ 8.55 – 8.48 (m, 4H, Ar*H*), 8.39 (d, *J* = 7.8 Hz, 2H, Ar*H*), 7.92 (t, *J* = 7.8 Hz, 1H, Ar*H*), 7.66 (dd, *J* = 8.2, 2.2 Hz, 2H, Ar*H*), 2.68 (t, *J* = 7.6 Hz, 4H, ArC*H$_2$*), 1.72 – 1.62 (m, 4H, ArCH$_2$C*H$_2$*), 1.42 – 1.27 (m, 12H, (C*H$_2$*)$_3$CH$_3$), 0.92 – 0.86 (m, 6H, C*H$_3$*).

^{13}C NMR (101 MHz, CDCl$_3$) δ 155.6, 154.2, 149.4, 138.4, 137.9, 136.9, 120.9, 120.5, 33.1, 31.8, 31.3, 29.0, 22.7, 14.2.

HRMS (ESI$^+$): calcd for $C_{27}H_{36}N_3^+$ [M+H]$^+$ 402.29037; found 402.29036.

2-([2,2'-Bipyridin]-6-yl)-4,4-dimethyl-4,5-dihydrooxazole (L49)

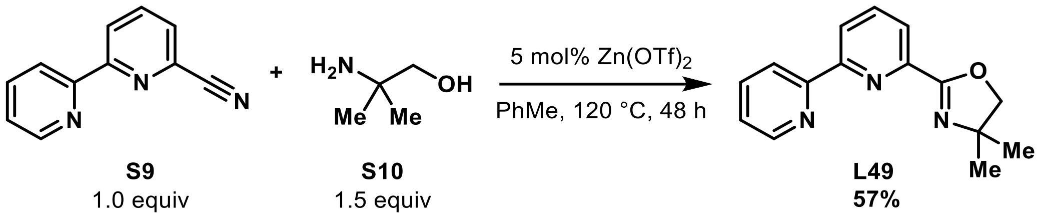

Following a slightly modified procedure,[60] an oven-dried 50 mL two-necked flask fitted with a reflux condenser was charged with 2,2’- bipyridyl-6-carbonitrile (**S9**) (300 mg, 1.64 mmol, 1.0 equiv) and zinc triflate (30 mg, 0.083 mmol, 0.05 equiv). The system was purged with argon and anhydrous toluene (5 mL) was added. The solution was stirred during 5 min and a solution of 2-amino-2-methyl-1-propanol (**S10**) (221 mg, 2.48 mmol, 1.5 equiv) in anhydrous toluene (10 mL) was added. The resulting mixture was set to reflux for 48 hours. At the end of the reaction, it was cooled down to room temperature, diluted with ethyl acetate (20 mL), and then washed with saturated aqueous sodium hydrogen carbonate (3 x 10 mL) and water (20 mL). The organic layer was dried over Na_2SO_4, filtered, and the solvent was removed under reduced pressure. The crude mixture was purified by flash column chromatography over silica gel using hexanes/EtOAc (10:1) as eluent to give 2-([2,2'-bipyridin]-6-yl)-4,4-dimethyl-4,5-dihydrooxazole **L49** (0.24 g, 0.96 mmol, 58%) as a colorless amorphous solid.

M.p.: 143.2 – 145.9 °C.

^{1}H NMR (400 MHz, $CDCl_3$) δ 8.67 (ddd, *J* = 4.8, 1.8, 0.9 Hz, 1H, Ar*H*), 8.55 (dt, *J* = 8.3, 1.2 Hz, 1H, Ar*H*), 8.53 (dd, *J* = 7.9, 1.1 Hz, 1H, Ar*H*), 8.11 (dd, *J* = 7.8, 1.1 Hz, 1H, Ar*H*), 7.90 (t, *J* = 7.8 Hz, 1H, Ar*H*), 7.82 (td, *J* = 7.7, 1.8 Hz, 1H, Ar*H*), 7.32 (ddd, *J* = 7.5, 4.8, 1.2 Hz, 1H, Ar*H*), 4.26 (s, 2H, CH_2), 1.45 (s, 6H, C$(Me)_2$).

^{13}C NMR (101 MHz, $CDCl_3$) δ 161.8, 156.3, 155.5, 149.2, 146.5, 137.7, 137.2, 124.3, 124.2, 123.2, 122.0, 79.9, 68.0, 28.5.

HRMS (EI): calcd for $C_{15}H_{15}N_3O_1^+$ $[M]^+$ 253.12096; found 253.12099.

***N*-(2-Hydroxyethyl)-[2,2'-bipyridine]-6-carboxamide (L51)**

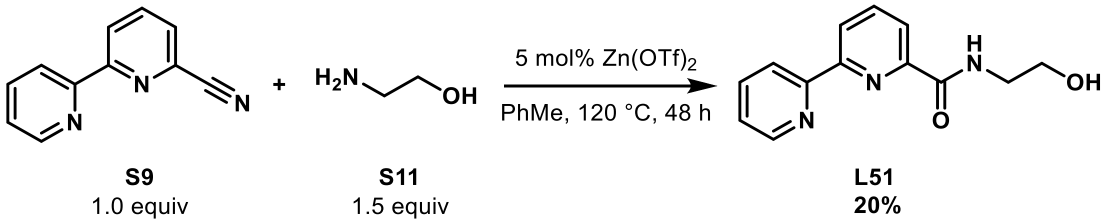

Following a slightly modified procedure,[60] an oven-dried 50 mL two-necked flask fitted with a reflux condenser was charged with 2,2'- bipyridyl-6-carbonitrile (**S9**) (205 mg, 1.13 mmol, 1.0 equiv) and zinc triflate (41 mg, 0.11 mmol, 0.1 equiv). The system was purged with argon and anhydrous toluene (5 mL) was added. The solution was stirred during 5 min and a solution of ethanolamine (**S11**) (0.11 mL, 1.8 mmol, 1.6 equiv) in anhydrous toluene (5 mL) was added. The resulting mixture was set to reflux for 48 hours. At the end of the reaction, it was cooled down to room temperature, diluted with ethyl acetate (20 mL), and then washed with saturated aqueous sodium hydrogen carbonate (3 x 10 mL) and water (20 mL). The organic layer was dried over Na_2SO_4, filtered, and the solvent was removed under reduced pressure. The crude mixture was purified by flash column chromatography over silica gel using hexanes/EtOAc (1:1 to EtOAc:MeOH (5:1)) as eluent to give *N*-(2-hydroxyethyl)-[2,2'-bipyridine]-6-carboxamide **L51** (55 mg, 0.23 mmol, 20%) as a colorless amorphous solid.

M.p.: 107.6 – 110.1 °C.

^{1}H NMR (400 MHz, CDCl$_3$) δ 8.82 – 8.73 (m, 1H, Ar*H*), 8.70 (s, 1H, N*H*), 8.50 (dd, *J* = 7.9, 1.1 Hz, 1H, Ar*H*), 8.36 (dt, *J* = 8.0, 1.1 Hz, 1H, Ar*H*), 8.24 (dd, *J* = 7.7, 1.1 Hz, 1H, Ar*H*), 8.00 (t, *J* = 7.8 Hz, 1H, Ar*H*), 7.91 (t, *J* = 7.9 Hz, 1H, Ar*H*), 7.41 (t, *J* = 6.3 Hz, 1H, Ar*H*), 3.93 – 3.89 (m, 2H, OC*H$_2$*), 3.72 (td, *J* = 5.8, 4.3 Hz, 2H, NC*H$_2$*), 3.14 (s, 1H, OH).

^{13}C NMR (101 MHz, CDCl$_3$) δ 165.5, 154.9 (br s), 149.4 (br s), 149.3, 138.7, 137.6 (br s), 131.8, 124.4, 123.9, 122.7, 121.3, 62.7, 42.8.

HRMS (ESI$^+$): calcd for $C_{13}H_{14}N_3O_2^+$ $[M+H]^+$ 244.10805; found 244.10821.

6-(4-(Trifluoromethyl)-1*H*-pyrazol-1-yl)-2,2'-bipyridine (L57)

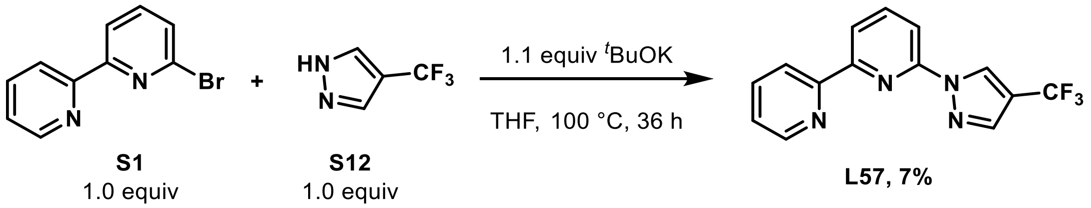

Following a slightly modified procedure,[61] a solution of 4-(trifluoromethyl)-1*H*-pyrazole (**S12**, 100 mg, 735 μmol, 1.0 equiv) in dry THF (1.0 mL) was added portion-wise to a sodium *tert*-butoxide (78 mg, 0.81 mmol, 1.1 equiv.) solution in dry THF (2.5 mL) in a heatgun-dried pressure Schlenk flask. After stirring at room temperature for 5 min, 6-bromo-2,2'-bipyridine (**S1**, 0.17 g, 0.74 mmol, 1.0 equiv) was added and the reaction mixture heated at 100 °C for 36 h. Upon cooling to room temperature, water and DCM were added, and the aqueous phase was extracted with DCM (3 × 50 mL). The combined organic layers were dried over Na_2SO_4, filtrated, and evaporated. Column chromatography over silica gel (EtOAc/Hex 5:95 to 1:9) and

further preparative TLC (EtOAc/Hex 1:9) yielded 14 mg (51 μmol, 7%) of 6-(4-(trifluoromethyl)-1*H*-pyrazol-1-yl)-2,2'-bipyridine (**L51**) as a yellowish solid.

^{1}H NMR (400 MHz, CDCl$_3$) δ 9.01 (p, *J* = 1.1 Hz, 1H, Ar*H*), 8.72 (ddd, *J* = 4.8, 1.8, 0.9 Hz, 1H, Ar*H*), 8.46 (dt, *J* = 8.0, 1.1 Hz, 1H, Ar*H*), 8.43 (dd, *J* = 7.0, 1.7 Hz, 1H, Ar*H*), 8.06 – 7.97 (m, 2H, Ar*H*), 7.94 (t, *J* = 0.7 Hz, 1H, Ar*H*), 7.89 (td, *J* = 7.7, 1.8 Hz, 1H, Ar*H*), 7.38 (ddd, *J* = 7.5, 4.8, 1.2 Hz, 1H, Ar*H*).

^{13}C NMR (101 MHz, CDCl$_3$) δ 154.9, 150.2, 149.4, 140.2, 139.0 (app q, *J* = 2.6 Hz), 137.3, 126.5 (app q, *J* = 3.5 Hz), 124.5, 121.3, 119.9, 112.8.

N.B.: Due to low amount of materials and C-F couplings, some signals are not resolved. Listed are the signals that could be observed, but 3 more signals are expected for the ^{13}C NMR spectrum of this compound.

HRMS (EI): calcd for $C_{14}H_9N_4F_3$ $[M]^+$ 290.07738; found 290.07806.

Synthesis of 1-([2,2'-bipyridin]-6-yl)-1*H*-indazole (L58) and 2-([2,2'-bipyridin]-6-yl)-2*H*-indazole (L59)

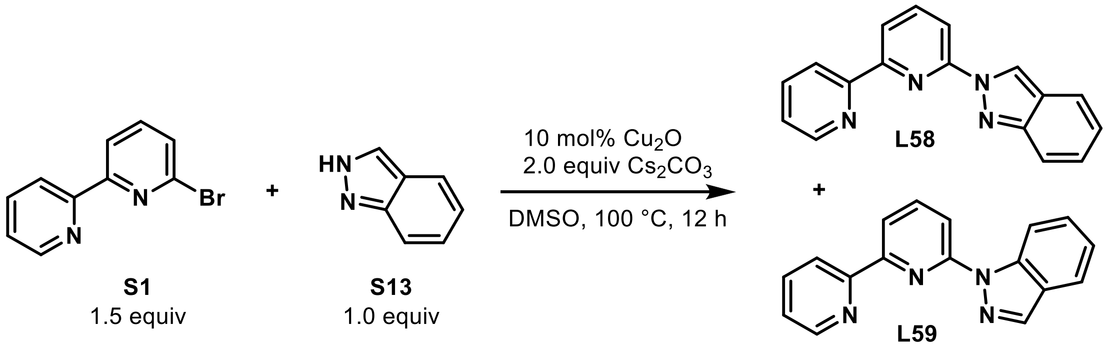

Following a slightly modified procedure,[56] cesium carbonate (456 mg, 1.40 mmol, 2.0 equiv), copper(I) oxide (10 mg, 70 μmol, 10 mol%), indazole (**S13**, 83 mg, 0.70 mmol, 1.0 equiv), and 6-bromo-2,2'-bipyridine (**S1**, 247 mg, 1.05 mmol, 1.5 equiv) were suspended in dry DMSO (1.3 mL) in a heatgun-dried Schlenk flask. The resulting mixture was heated for 12 h at 100 °C. Upon cooling to room temperature, DCM was added, and the precipitate filtrated through a Celite pad. The filtrate was then evaporated and the residue purified via column chromatography over silica gel (EtOAc/Hex 2:98 to 1:9), giving 75 mg (0.27 mmol, 39%) of **L58** and 32 mg (0.12 mmol, 17%) of **L59**.

1-([2,2'-Bipyridin]-6-yl)-1*H*-indazole (L58)

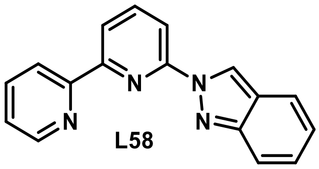

M.p.: 138 °C.

Rf (Hexane/EtOAc (85:15)) 0.23.

^{1}H NMR (600 MHz, CDCl$_3$) δ 9.31 (s, 1H, Ar*H*), 8.77 (d, *J* = 4.6 Hz, 1H, Ar*H*), 8.57 (dt, *J* = 7.9, 1.0 Hz, 1H, Ar*H*), 8.48 (d, *J* = 7.7 Hz, 1H, Ar*H*), 8.34 (dd, *J* = 8.1, 0.9 Hz, 1H, Ar*H*), 8.06 (t, *J* = 7.9 Hz, 1H, Ar*H*), 7.94 (t, *J* = 7.8 Hz, 1H, Ar*H*), 7.80 – 7.75 (m, 2H, Ar*H*), 7.42 (t, *J* = 5.6 Hz, 1H, Ar*H*), 7.35 (ddd, *J* = 8.8, 6.5, 1.0 Hz, 1H, Ar*H*), 7.12 (ddd, *J* = 8.7, 6.5, 0.8 Hz, 1H, Ar*H*).

^{13}C NMR (151 MHz, CDCl$_3$) δ 154.6, 151.4 (2C), 150.4, 148.9, 140.0, 137.6, 127.6, 124.3, 122.8, 122.4, 121.4, 121.2, 120.6, 120.1, 118.1, 114.3.

HRMS (EI): calcd for $C_{17}H_{12}N_4$ $[M]^+$ 272.10564; found 272.10584.

2-([2,2'-Bipyridin]-6-yl)-2*H*-indazole (L59)

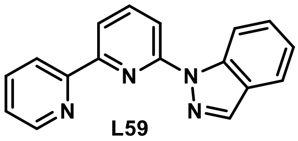

M.p.: 117 °C.

Rf (Hexane/EtOAc (85:15)) 0.34.

^{1}H NMR (600 MHz, CDCl$_3$) δ 8.93 (dq, *J* = 8.6, 0.9 Hz, 1H, Ar*H*), 8.75 (ddd, *J* = 4.8, 1.9, 0.9 Hz, 1H, Ar*H*), 8.53 (dt, *J* = 8.0, 1.1 Hz, 1H, Ar*H*), 8.32 (dd, *J* = 7.6, 1.0 Hz, 1H, Ar*H*), 8.24 (d, *J* = 0.9 Hz, 1H, Ar*H*), 8.11 (dd, *J* = 8.2, 0.9 Hz, 1H, Ar*H*), 8.02 – 7.91 (m, 2H, Ar*H*), 7.82 (dt, *J* = 8.0, 1.0 Hz, 1H, Ar*H*), 7.59 (ddd, *J* = 8.4, 7.0, 1.2 Hz, 1H, Ar*H*), 7.39 (ddd, *J* = 7.5, 4.8, 1.2 Hz, 1H, Ar*H*), 7.32 (ddd, *J* = 7.9, 7.0, 0.9 Hz, 1H, Ar*H*).

^{13}C NMR (151 MHz, CDCl$_3$) δ 155.6, 153.8 (2C), 149.0, 139.5, 138.7, 137.5, 136.9, 128.0, 126.1, 124.0, 122.5, 121.4, 121.0, 117.6, 114.9, 114.1.

HRMS (EI): calcd for $C_{17}H_{12}N_4$ $[M]^+$ 272.10564; found 272.10584.

3. Synthesis of starting materials

Methyl 3-(4-iodophenyl)propanoate (S15)

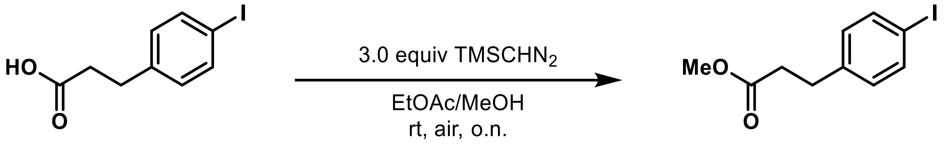

S14, 1.0 equiv **S15**, quant.

Following a slightly modified procedure,[62] a 12 mL vial equipped with a Teflon-coated stir bar was charged with 3-(4-iodophenyl)propanoic acid (**S14**, 112 mg, 0.406 mmol, 1.0 equiv). Then, under normal atmosphere, EtOAc and MeOH (5.0 mL and 2.0 mL) respectively were added and the resulting mixture was stirred for 2 min at rt to get a clear solution. At this point, (trimethylsilyl)-diazomethane (0.30 mL, 2 M in Et_2O, 0.61 mmol, 1.5 equiv) was added by syringe, and the yellow solution is stirred for 3 h. An aliquot indicated 55% conversion, so another 1.5 equiv. of (trimethylsilyl)-diazomethane was added to the solution and let it stirred overnight at rt. The obtained yellow suspension is then carefully evaporated under reduced pressure. The crude was finally purified by column chromatography over silica gel (10:1 then 8:2 pentane / Et_2O as eluent) affording the expected product methyl 3-(4-iodophenyl)propanoate **S15** in quantitative yield as a white solid (178 mg, 0.406 mmol).

Rf (Pentane / Et_2O = 8:2) 0.25.

^{1}H NMR (400 MHz, $CDCl_3$, 25 °C) δ 7.63 – 7.58 (dt, *J* = 8.4 Hz, 1.9 Hz, 2H, Ar*H*), 6.98 – 6.93 (dt, *J* = 8.4 Hz, 1.9 Hz, 2H, Ar*H*), 3.66 (s, 3H, *Me*), 2.89 (t, *J* = 7.7 Hz, 2H, ArCH_2), 2.60 (dd, *J* = 8.1, 7.2 Hz, 2H, $ArCH_2CH_2$).

^{13}C NMR (101 MHz, $CDCl_3$, 25 °C) δ 173.1, 140.3, 137.7, 130.5, 91.6, 51.8, 35.5, 30.5.

The values of the NMR spectra are in accordance with reported literature data.[63]

((4-Iodobenzyl)oxy)triisopropylsilane (S17)

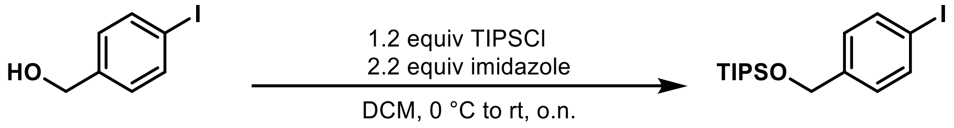

S16, 1.0 equiv **S17**, *quant.*

Following a slightly modified procedure,[64] an oven-dried 25 mL equipped with a Teflon-coated stir bar was charged with 4-iodobenzyl alcohol (**S16**, 520 mg, 2.22 mmol, 1.0 equiv) and 1*H*-imidazole (333 mg, 4.89 mmol, 2.2 equiv) in DCM (11 mL) at 0 °C. To this solution was added

dropwise TIPSCl (0.57 mL, 2.67 mmol, 1.2 equiv), and the resulting mixture was let warming-up to rt and stirred overnight. The solution was quenched with water and the DCM layer separated. The organic layer was further washed with 0.5 N HCl and $NaHCO_3$(sat). The combined organic layers were dried over $MgSO_4$, filtered, and evaporated under reduced pressure. The crude yellow oil was already of good purity, and was quickly filter through a silica pad to obtained (4-iodobenzyloxy)triisopropylsilane (**S17**) as a light yellow oil (858 mg, quant.).

^{1}H NMR (400 MHz, $CDCl_3$) δ 7.67 – 7.63 (m, 2H, Ar*H*), 7.13 – 7.08 (m, 2H, Ar*H*), 4.77 (s, 1H, ArC*H_2*), 1.20 – 1.12 (m, 3H, $(Me)_2$C*H*), 1.10 – 1.06 (m, 18H, $(Me)_2$CH).

^{13}C NMR (101 MHz, $CDCl_3$) δ 141.5, 137.3, 127.9, 92.0, 64.6, 18.2, 12.1.

HRMS (ESI$^+$): calcd for $C_{16}H_{27}O_1I_1Si_1Na_1$ $[M+Na]^+$ 413.07681; found 413.07729.

Diethyl (4-iodobenzyl)phosphonate (S19)

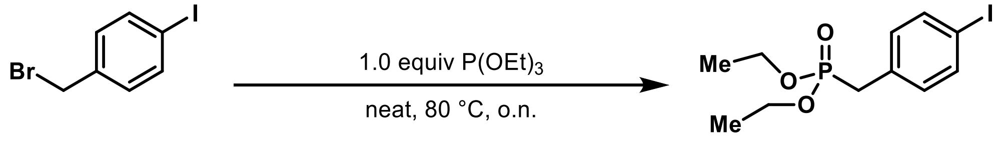

S18, 1.3 equiv　　　　**S19**, 85%

Following a slightly modified procedure,[65] 4-iodobenzyl bromide **S18** (386 mg, 1.30 mmol, 1.3 equiv.) and triethylphosphite (172 µL, 1.00 mmol, 1.0 equiv.) were given into a round bottom flask. The mixture was refluxed at 80 °C overnight. Upon cooling to room temperature, the crude was purified via column chromatography (hexanes:EtOAc 9:1) to give diethyl (4-iodobenzyl)phosphonate (**S19**) as a colorless oil (0.3 g, 85% yield).

The values of the ^{1}H NMR spectrum are in accordance with reported literature data.[65]

^{1}H NMR (400 MHz, $CDCl_3$) δ 7.63 (dd, *J* = 8.4, 1.1 Hz, 2H, Ar*H*), 7.04 (dd, *J* = 8.4, 2.5 Hz, 2H, Ar*H*), 4.07 – 3.95 (m, 4H, OC*H_2*CH_3), 3.07 (d, *J* = 21.7 Hz, 2H, ArC*H_2*), 1.25 (t, *J* = 7.1 Hz, 6H, OCH_2C*H_3*).

^{13}C NMR (101 MHz, $CDCl_3$) δ 137.7 (d, *J* = 3.1 Hz), 131.9 (d, *J* = 6.2 Hz), 131.6 (d, *J* = 9.2 Hz), 92.5 (d, *J* = 4.6 Hz), 62.4 (d, *J* = 6.8 Hz), 33.5 (d, *J* = 138.2 Hz), 16.5 (d, *J* = 6.1 Hz).

Ethyl 2-(4-iodophenoxy)-2-methylpropanoate (S22)

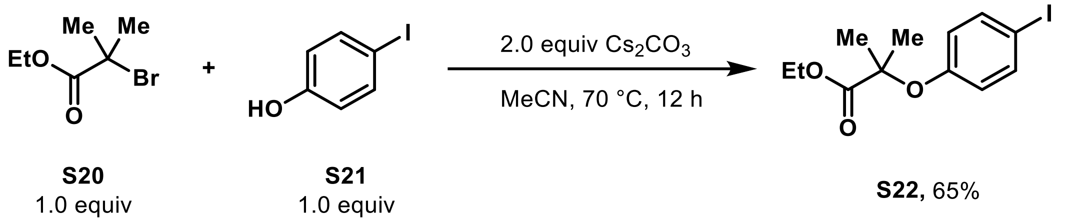

Following a reported procedure,[66] a flame-dried 100-mL round-bottom flask, equipped with a stir bar, was charged with ethyl 2-bromo-2-methylpropanoate (**S20**, 745 mg, 3.82 mmol, 1.0 equiv), 4-iodophenol (**S21**, 0.840 g, 3,82 mmol, 1.0 equiv), caesium carbonate (2.49 g, 7,64 mmol, 2.0 equiv), and MeCN (8.4 mL). The mixture was allowed to stir for 12 h at 70 °C. The mixture was then allowed to cool to room temperature and diluted with water (10 mL). The aqueous solution was then washed with ethyl acetate (10 mL x 3). The combined organic layers were dried over sodium sulfate and filtered. The solvent was removed by rotary evaporation and the residue was purified by flash silica gel chromatography (Eluent: 100:1 to 10:1 hexanes:ethyl acetate). The product **S22** was isolated as a white solid (0.70 g, 2.5 mmol, 65% yield).

^{1}H NMR (300 MHz, $CDCl_3$) δ 7.57 – 7.45 (m, 2H, Ar*H*), 6.77 – 6.51 (m, 2H, Ar*H*), 4.22 (q, *J* = 7.1 Hz, 2H, OCH_2CH_3), 1.58 (s, 6H, *gem-Me*), 1.24 (t, *J* = 7.1 Hz, 3H, OCH_2CH_3).

^{13}C NMR (75 MHz, $CDCl_3$) δ 174.1, 155.6, 138.2, 121.4, 84.9, 79.5, 61.7, 25.4, 14.2.

HRMS (ESI$^+$): calcd for $C_{12}H_{15}O_3I_1Na_1$ $[M+Na]^+$ 356.99581; found 356.99582.

4-Allyl-2-methoxyphenyl 4-bromobenzoate (S25)

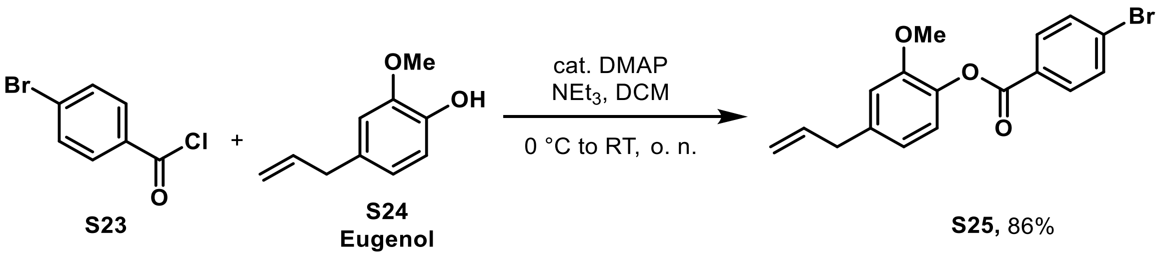

To a solution of eugenol (**S24**, 0.31 mL, 2.0 mmol, 1.0 equiv) and triethylamine (0.84 mL, 6.0 mmol, 3.0 equiv) in dry DCM (30 mL) at 0 ºC, was added *p*-bromobenzoyl choride (**S23**, 0.44 g, 2.0 mmol, 1.0 equiv) and DMAP (12 mg, 0.10 mmol, 0.05 equiv). After 1 h, the reaction mixture was allowed to warm up to room temperature and stirred for 16 h. The resulting solution was washed with water (20 mL) and the organic layer was dried over Na_2SO_4 and concentrated. The crude residue was purified by column chromatography over silica gel using Hexane/EtOAc

(20:1 to 10:1) as eluent gradient to afford the final product **S25** as a white solid (0.60 g, 1.7 mmol, 86% yield).

Rf (Hexane:EtOAc (9:1)) 0.5.

M.p.: 88.1 – 89.3 °C.

^{1}H NMR (400 MHz, $CDCl_3$) δ 8.09 – 8.05 (m, 2H, Ar*H*), 7.67 – 7.62 (m, 2H, Ar*H*), 7.06 (d, *J* = 7.9 Hz, 1H, Ar*H*), 6.85 – 6.79 (m, 2H, Ar*H*), 5.99 (ddt, *J* = 16.8, 10.1, 6.7 Hz, 1H, C=C*H*), 5.16 – 5.09 (m, 2H, C=CH_2), 3.80 (s, 3H, O*Me*), 3.41 (d, *J* = 6.7 Hz, 2H, ArCH_2).

^{13}C NMR (101 MHz, $CDCl_3$) δ 164.3, 151.1, 139.4, 138.1, 137.2, 132.0, 131.9, 128.8, 128.6, 122.7, 120.9, 116.4, 113.0, 56.0, 40.3.

HRMS (ESI$^+$): calcd for $C_{17}H_{16}O_3Br_1$ $[M+H]^+$ 347.02775; found 347.02802.

***tert*-Butyl 4-(4-iodobenzyl)-3-oxopiperazine-1-carboxylate (S27)**

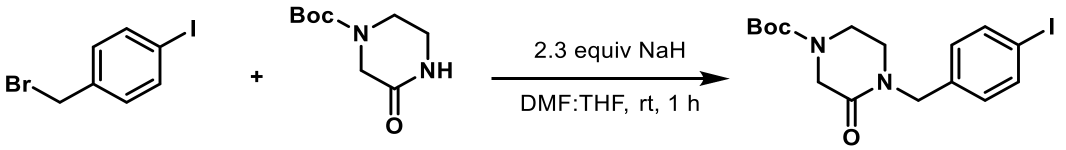

Following a reported procedure,[67] sodium hydride (112 mg, 4.66 mmol, 2.3 equiv) was added to a slurry of 3-oxo-piperazine-l- carboxylic acid *tert*-butyl ester (**S26**, 0.40 g, 2.0 mmol, 1.0 equiv) in dry N,N-dimethylformamide (25 mL) and 90 mL dry tetrahydrofuran under argon atmosphere and cooled to 0 °C. The mixture was stirred at room temperature until a solution was obtained after 30 minutes. 4-Iodobenzyl bromide (**S18**, 0.59 g, 2.0 mmol, 1 equiv), dissolved in 20 mL tetrahydrofuran, was added dropwise. The reaction was stirred for 1 hour to give a white slurry. Ethyl acetate and water was added. The organic phase was washed three times with water, once with brine, dried sodium sulfate and evaporated. The crude was purified on a silica column using dichloromethane/methanol (97:3) as eluent to give the product **S27** in 60% yield (0.50 g, 1.2 mmol).

^{1}H NMR (300 MHz, $CDCl_3$) δ 7.70 – 7.63 (m, 2H, Ar*H*), 7.06 – 6.98 (m, 2H, Ar*H*), 4.55 (s, 2H, ArCH_2), 4.15 (s, 2H, NCH_2C(O)N), 3.68 – 3.48 (m, 2H, BocNCH_2CH_2N), 3.29 – 3.13 (m, 2H, BocNCH_2CH_2N), 1.46 (s, 9H, *tBu*).

The ^{1}H NMR data are in accordance with the reported data.[67]

4-Bromobenzyl 4-bromobenzoate (S29)

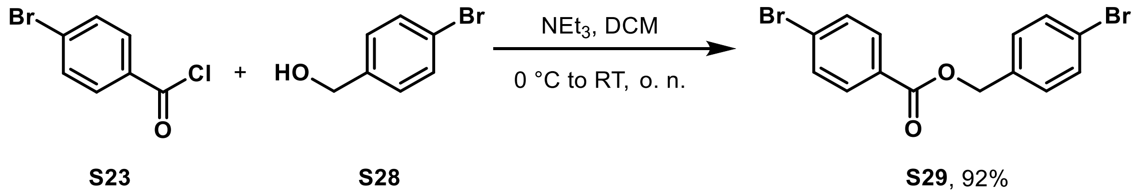

To a solution of *p*-bromobenzylalcohol (**S28**, 0.56 g, 3.0 mmol, 1.0 equiv) and triethylamine (0.56 mL, 4.0 mmol, 1.33 equiv) in dry DCM (20 mL), was added *p*-bromobenzoyl choride (**S23**, 0.66 g, 3.0 mmol, 1.0 equiv) at 0 ºC. After 1 h, the reaction mixture was allowed to warm up to room temperature and stirred for 12 h. The resulting solution was washed with water (20 mL) and the organic layer was dried over Na_2SO_4 and concentrated. The crude residue was purified by column chromatography over silica gel using (1:1 to 3:1) EtOAc/hexanes as eluent gradient to afford the final product **S29** as a white solid (1.02 g, 2.76 mmol, 92% yield).

^{1}H NMR (300 MHz, $CDCl_3$) δ 8.00 – 7.83 (m, 2H, Ar*H*), 7.63 – 7.55 (m, 2H, Ar*H*), 7.55 – 7.49 (m, 2H, Ar*H*), 7.34 – 7.28 (m, 2H, Ar*H*), 5.30 (s, 2H, C*H*$_2$).

^{13}C NMR (75 MHz, $CDCl_3$) δ 165.7, 134.9, 132.0, 131.9, 131.3, 130.1, 129.0, 128.5, 122.6, 66.3.

These NMR data are in accordance with the literature data.[68]

***tert*-Butyl 3-((4-(4-bromophenyl)-4-oxobutanoyl)oxy)azetidine-1-carboxylate (S32)**

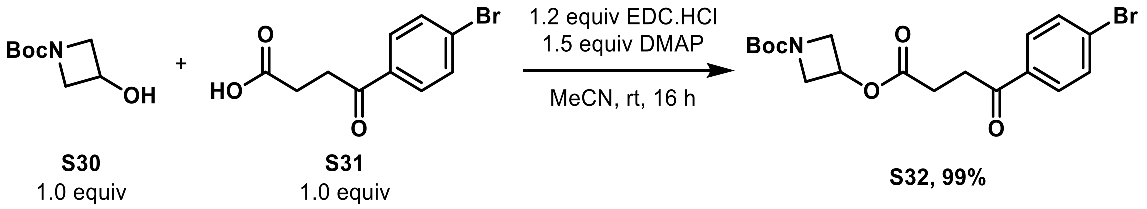

To a screw-cap vial equipped with a Teflon-coated stir bar were added *tert*-butyl 3-hydroxyazetidine-1-carboxylate (**S30**, 0.087 g, 0.50 mmol, 1.0 equiv), 4-(4-bromophenyl)-4-oxobutanoic acid (**S31**, 0.13 g, 0.50 mmol, 1.0 equiv), EDC.HCl (0.12 g, 0.60 mmol, 1.2 equiv), and DMAP (0.092 g, 0.75 mmol, 1.5 equiv). At 0 °C, 4.0 mL of dry MeCN is added and the reaction mixture is stirred for 5 min at this temperature. Then, the reaction is stirred at room temperature for 16 hours. The reaction is partitioned between water and EtOAc. The aqueous layer is extracted with EtOAc (3 x 10 mL). The combined organic layers are dried over $MgSO_4$, concentrated to dryness and purified by column chromatography over silica gel using hexanes/EtOAc (5:1 then 4:1) as eluent, affording *tert*-butyl 3-((4-(4-bromophenyl)-4-

oxobutanoyl)oxy)azetidine-1-carboxylate (**S32**) as a white solid (206 mg, 0.500 mmol, 99% yield).

M.p.: 52.6 – 57.1 °C.

Rf (Hexane:EtOAc (1:1)) 0.45.

^{1}H NMR (400 MHz, CDCl$_3$) δ 7.86 – 7.82 (m, 2H, Ar*H*), 7.64 – 7.59 (m, 2H, Ar*H*), 5.15 (tt, *J* = 6.7, 4.3 Hz, 1H, OC*H*), 4.23 (ddd, *J* = 10.1, 6.7, 1.2 Hz, 2H, NC*H$_2$*), 3.91 (ddd, *J* = 10.0, 4.3, 1.2 Hz, 2H, NC*H$_2$*), 3.28 (dd, *J* = 6.9, 6.0 Hz, 2H, ArC(O)C*H$_2$*), 2.79 (t, *J* = 6.4 Hz, 2H, OC(O)C*H$_2$*), 1.44 (s, 9H, *tBu*).

^{13}C NMR (101 MHz, CDCl$_3$) δ 196.9, 172.4, 156.2, 135.2, 132.2, 129.7, 128.7, 80.0, 63.8, 56.3 (br s), 33.3, 28.5, 28.1.

HRMS (ESI$^+$): calcd for $C_{18}H_{22}N_1O_5Br_1Na_1$ [M+Na]$^+$ 434.05737; found 434.05787.

1-(4-Bromobenzoyl)pyrrolidin-2-one (S34)

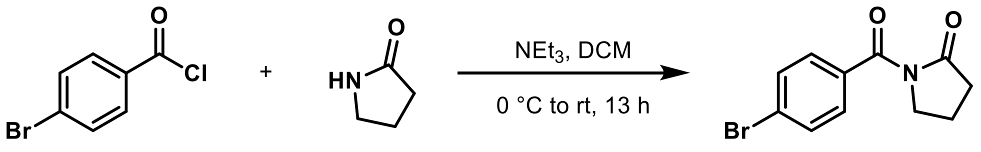

S23
1.0 equiv

S33
1.0 equiv

S34, 33%

To a solution of lactam **S33** (0.25 g, 3.0 mmol, 1.0 equiv) and triethylamine (0.56 mL, 4.0 mmol, 1.33 equiv) in dry DCM (20 mL), was added *p*-bromobenzoyl choride **S23** (0.66 g, 3.0 mmol, 1.0 equiv) at 0 ºC. After 1 h, the reaction mixture was allowed to warm up to room temperature and stirred for 12 h. The resulting solution was washed with water (20 mL) and the organic layer was dried over Na_2SO_4 and concentrated. The crude residue was purified by column chromatography over silica gel using (50-75%) EtOAc/hexanes as eluent gradient to afford the final product **S34** as a white solid (0.27 g, 1.0 mmol, 33% yield).

M.p.: 128.4 – 130.5 °C.

^{1}H NMR (300 MHz, CDCl$_3$) δ 7.58 – 7.52 (m, 2H, Ar*H*), 7.50 – 7.44 (m, 2H, Ar*H*), 4.00 – 3.81 (m, 2H, NC*H$_2$*), 2.61 (t, *J* = 8.0 Hz, 2H, NC(O)C*H$_2$*)), 2.15 (p, *J* = 7.6 Hz, 2H, NCH$_2$C*H$_2$*).

^{13}C NMR (75 MHz, CDCl$_3$) δ 176.0, 174.7, 133.2, 131.2, 130.7, 126.9, 46.7, 33.4, 17.8.

HRMS (ESI$^+$): calcd for $C_{11}H_{10}N_1O_2Br_1Na_1$ [M+Na]$^+$ 289.97872; found 289.97894.

1-(4-Bromophenyl)-4-(3-oxo-8-azabicyclo[3.2.1]octan-8-yl)butane-1,4-dione (S36)

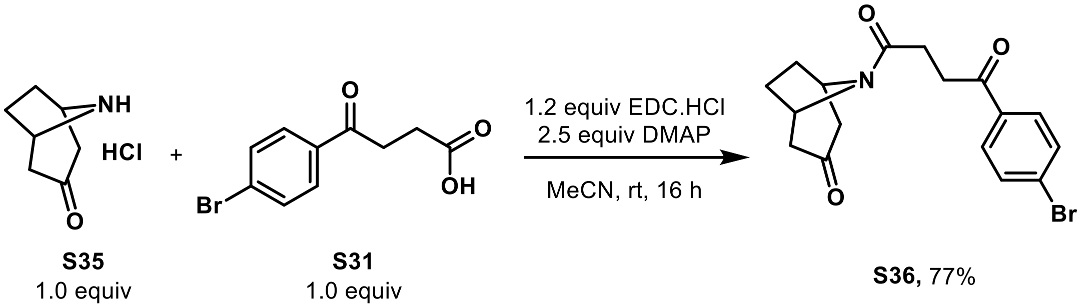

To a screw-cap vial equipped with a Teflon-coated stir bar were added nortropinone hydrochloride (**S35**, 81 mg, 0.50 mmol, 1.0 equiv), 4-(4-bromophenyl)-4-oxobutanoic acid (**S31**, 0.13 g, 0.50 mmol, 1.0 equiv), EDC.HCl (0.12 g, 0.60 mmol, 1.2 equiv), and DMAP (153 mg, 1.25 mmol, 2.5 equiv). At 0 °C, 4.0 mL of dry MeCN was added and the reaction mixture was stirred for 5 min at this temperature. Then, the reaction was stirred at rt for 16 hours. The reaction is partitioned between water and EtOAc. The aqueous layer was extracted with EtOAc (3 x 10 mL). The combined organic layers were dried over $MgSO_4$, concentrated to dryness and purified by column chromatography over silica gel using hexanes/EtOAc (1:2 then 1:3) as eluent, affording 1-(4-Bromophenyl)-4-(3-oxo-8-azabicyclo[3.2.1]octan-8-yl)butane-1,4-dione (**S36**) as a white solid (154 mg, 0.384 mmol, 77% yield).

M.p.: 121.6 – 123.8 °C.

Rf (Hexanes:EtOAc (1:3)) 0.2.

^{1}H NMR (400 MHz, $CDCl_3$) δ 7.88 – 7.84 (m, 2H, Ar*H*), 7.63 – 7.59 (m, 2H, Ar*H*), 4.91 (br s, 1H, NC*H*), 4.61 (br s, 1H, NC*H*), 3.46 (m, 1H, ArC(O)CH_2), 3.36 – 3.23 (m, 1H, ArC(O)CH_2), 2.90 – 2.83 (br d, J = 16.0 Hz, 1H, , CH_2C(O)CH_2), 2.83 (td, *J* = 6.3, 2.4 Hz, 3H, NCHCH_2CH_2), 2.73 (d, *J* = 16.0 Hz, 1H, CH_2C(O)CH_2), 2.48 (d, *J* = 15.9 Hz, 1H, CH_2C(O)CH_2), 2.38 (d, *J* = 15.9 Hz, 1H, CH_2C(O)CH_2), 2.21 (m, 1H, NC(O)CH_2), 2.06 (m, 1H, NC(O)CH_2), 1.89 – 1.76 (m, 1H, NCHCH_2CH_2), 1.76 – 1.65 (m, 1H, NCHCH_2CH_2).

^{13}C NMR (101 MHz, $CDCl_3$) δ 207.6, 198.1, 168.5, 135.5, 132.1, 129.8, 128.6, 53.9, 51.4, 49.7, 49.0, 33.5, 30.1, 28.0, 27.6.

HRMS (ESI$^+$): calcd for $C_{17}H_{19}N_1O_3Br_1$ $[M+H]^+$ 364.05429; found 364.05423.

4-((2S,3R)-1-(4-Fluorophenyl)-3-((S)-3-(4-fluorophenyl)-3-hydroxypropyl)-4-oxoazetidin-2-yl)phenyl 4-bromobenzoate (S38)

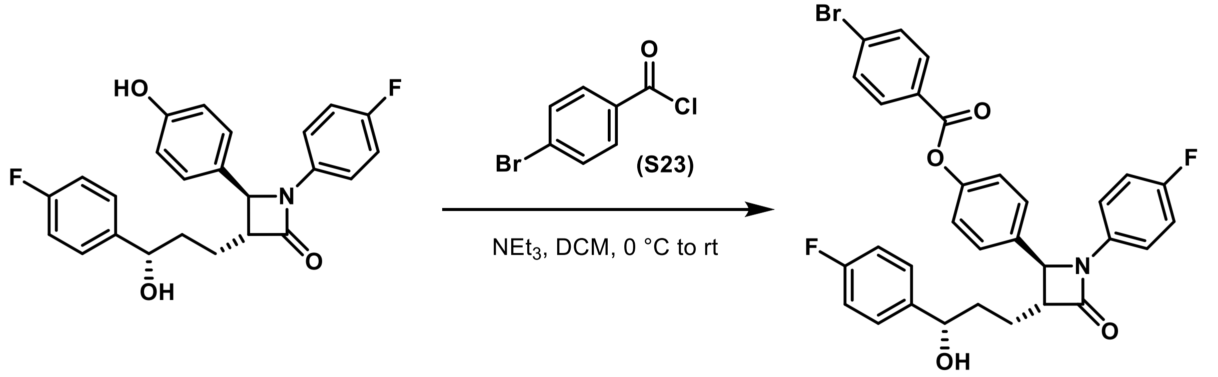

Ezetimibe (S37) S38, 73%

In an oven dried-Schlenk under argon, Ezetimibe (**S37**, 48.5 mg, 0.118 mmol, 1.0 equiv) was weighted in and dissolved in DCM (1.8 mL). Triethylamine (0.10 mL, 0,65 mmol, 5.5 equiv) was added and cooled down to 0 °C. *p*-Bromobenzoylchloride (**S23**, 26.0 mg, 0,118 mmol, 1.0 equiv) was added and stirred for 15 minutes at 0 °C, and after that for 5 h at RT. The reaction mixture was extractied with water and ethylacetate, dried over sodium sulfate, filtered and evaporated under reduced pressure. The crude residue was purified by column chromatography over silica gel using EtOAc/hexanes mixture as eluent gradient to afford the final product **S38** as a white solid (51.3 mg, 0.0866 mmol, 73% yield).

M.p.: 68.4 °C.

^{1}H NMR (300 MHz, $CDCl_3$) δ 8.08 – 8.01 (m, 2H, Ar*H*), 7.69 – 7.63 (m, 2H, Ar*H*), 7.43 – 7.36 (m, 2H, Ar*H*), 7.34 – 7.27 (m, 2H, Ar*H*), 7.23 (dd, *J* = 6.6, 2.0 Hz, 4H, Ar*H*), 7.03 (t, *J* = 8.7 Hz, 2H, Ar*H*), 6.99 – 6.90 (m, 2H, Ar*H*), 4.74 (t, *J* = 6.0 Hz, 1H, ArC*H*OH), 4.66 (d, *J* = 2.3 Hz, 1H, ArC*H*N), 3.27 – 2.96 (m, 1H, NC(O)C*H*), 2.04 – 1.86 (m, 4H, CH_2CH_2).

1-((3S,4R)-3-((Benzo[d][1,3]dioxol-5-yloxy)methyl)-4-(4-fluorophenyl)piperidin-1-yl)-4-(4-bromophenyl)butane-1,4-dione (S40)

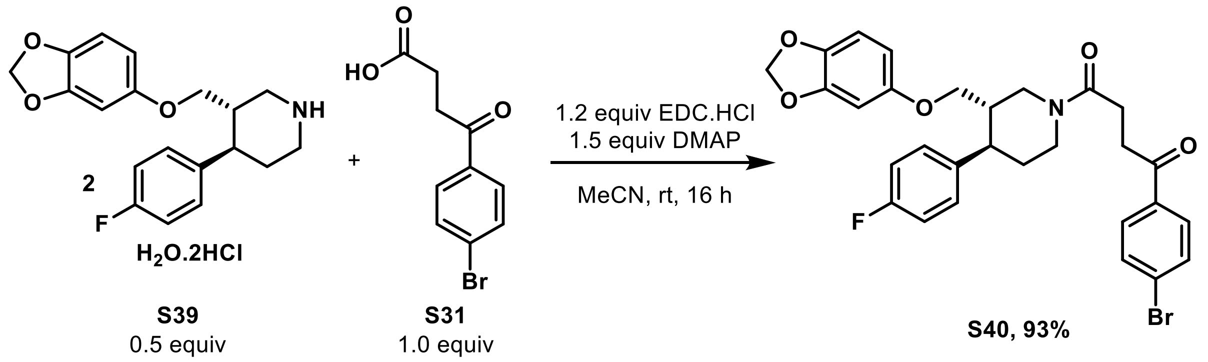

To a screw-cap vial equipped with a Teflon-coated stir bar were added paroxetine hemihydrate hydrochloride (**S39**, 0.22 g, 0.29 mmol, 0.5 equiv), 4-(4-bromophenyl)-4-oxobutanoic acid (**S31**, 151 mg, 0.587 mmol, 1.0 equiv), EDC.HCl (0.135 g, 0.704 mmol, 1.2 equiv), and DMAP (158 mg, 1.29 mmol, 2.2 equiv). At 0 °C, 5.0 mL of dry MeCN was added and the reaction mixture was stirred for 5 min at this temperature. Then, the reaction was stirred at rt for 16 hours. The reaction was partitioned between water and EtOAc. The aqueous layer was extracted with EtOAc (3 x 10 mL). The combined organic layers were dried over $MgSO_4$, concentrated to dryness and purified by column chromatography over silica gel using hexanes/EtOAc (3:1 then 2:1) as eluent, affording 1-((3S,4R)-3-((Benzo[d][1,3]dioxol-5-yloxy)methyl)-4-(4-fluorophenyl)piperidin-1-yl)-4-(4-bromophenyl)butane-1,4-dione (**S40**) as a white solid (310 mg, 0.545 mmol, 93% yield).

Rf (Hexanes:EtOAc (1:1)) 0.33.

M.p.: 107.1 – 109.7 °C.

^{1}H NMR (400 MHz, $CDCl_3$) δ 7.93 – 7.87 (m, 2H, Ar*H*), 7.64 – 7.59 (m, 2H, Ar*H*), 7.18 – 7.12 (m, 2H, Ar*H*), 7.03 – 6.95 (m, 2H, Ar*H*), 6.63 (d, *J* = 8.5 Hz, 1H, Ar*H*), 6.36 (d, *J* = 2.5 Hz, 1H, Ar*H*), 6.15 (dd, *J* = 8.5, 2.5 Hz, 1H, Ar*H*), 5.88 (s, 2H, OCH_2O), 4.76 (br s, 1H, CH_2N), 4.35 (br s, 1H, CH_2N), 3.63 (dd, *J* = 9.5, 2.8 Hz, 1H, CH_2OAr), 3.48 (dd, *J* = 9.5, 6.5 Hz, 1H, CH_2OAr), 3.34 (br t, *J* = 6.4 Hz, 2H, $ArC(O)CH_2$), 3.13 (br s, 1H, Ar_FCH), 2.98 – 2.66 (m, 4H, CH_2CH_2N+$OC(O)CH_2$), 2.04 (br s, 1H, $CHCH_2N$), 1.85 (br s, 2H, CH_2CH_2N).

^{13}C NMR (101 MHz, $CDCl_3$) δ 198.5, 170.1, 163.0, 160.6, 154.3 (br s), 148.3, 141.9, 138.8, 138.7, 135.7, 132.0, 129.8, 129.0, 128.9, 128.3, 115.9, 115.7, 108.0, 105.7, 101.3, 98.1, 68.8, 49.0 (br s), 46.1 (br s), 44.2 (br s), 42.7 (br s), 34.3 (br s), 33.7, 27.38.

HRMS (ESI$^+$): calcd for $C_{29}H_{28}N_1O_5Br_1F_1$ $[M+H]^+$ 568.112952; found 568.11361.

4. Stoichiometric reactivity

4.1. Reaction discovery and control experiments

As a starting point of our study, a typical reaction was performed by stirring oxidative complex **4** (26 mg, 47 μmol, 1.0 equiv) in 2.0 mL of DMA, at room temperature, in a pressure Schlenk under 1 atmosphere of N_2O (Table, S1, entry 1). After 22 h, the reaction was quenched with a saturated solution of NH_4Cl, extracted 3 times with Et_2O and analyzed by NMR using 4-nitro-1-fluorobenzene as internal standard. The reaction was still very orange, meaning incomplete conversion of the starting material. Interestingly, 15% of phenol **7** could be detected, and only a minor amount (< 5%) of dimer **5** was observed (Table S1, entry 1). As the reaction is very slow, it may be due to disproportionation yielding a low-valent Ni–Ar intermediate. Slow and incomplete conversion may be also problematic for the development of a catalytic system, therefore we wondered if we could enhance the reactivity by using a more reactive, low-valent Ni(I)–Ar complex. Such intermediate could be easily generated in situ under reductive conditions using a reducing agent such as Zn or Mn dust.

The addition of 7 equivalents of Zn dust (22 mg, 0.33 mmol) led to a greater amount of phenol formation (53-55%), while keeping dimer formation to a minimal level (Table S1, entry 2). Surprisingly, an amine side product **S41** is also formed in 13 – 15% yield (See Fig. S2-4), which may derive from the activation of the amide bond of DMA at a low valent nickel center (Ni(0) or Ni(I)). Performing the reaction with Zn dust under air also gave 50% yield of phenol, but with less dimer and no amine side product (Table S1, entry 3). The reaction was also much faster, as it turned light green within 2 h. This is an interesting result, as the reaction of [dtbbpyNi(II)Ar]Br complex (**4**) with air is usually not clean and gives C-Br reductive elimination, homocoupling but generally no phenol. A control experiment under argon in presence of Zn was also undertaken, and no phenol was detected and the reaction turned black quickly, corresponding to Ni black formation and substantial amount of homocoupling (Table S1, entry 4). This result is consistent with the fact that either N_2O or O_2 (from air) can stabilize and react with the unstable Ni(I) intermediate. It also points to the fact that an oxidizing/electrophilic O-source is required for the C-O bond formation. Due to higher activation barrier, N_2O reacts in a slower manner that O_2, thus allowing time for partial decomposition of Ni(I) with DMA. Interestingly, homogeneous electrophilic O-source such as camphorsulfonyloxaziridine **S42**, (Z)-1,2-diphenyldiazene 1-oxide **S43** or pyridine N-oxide **S44** are not suitable for forging the C-O bond in good yield (5%, 0% and 0% respectively, Table S1, entries 5-7). These results highlights that the compatibility of the oxidant with zinc (or

reducing agent) is a potential major hurdle for developing catalytic variants of these stoichiometric reactions. Finally, a quick direct work-up only led to very low amount of phenol, therefore confirming that the work-up is not responsible for large amount of phenol formation (Table S1, entry 8).

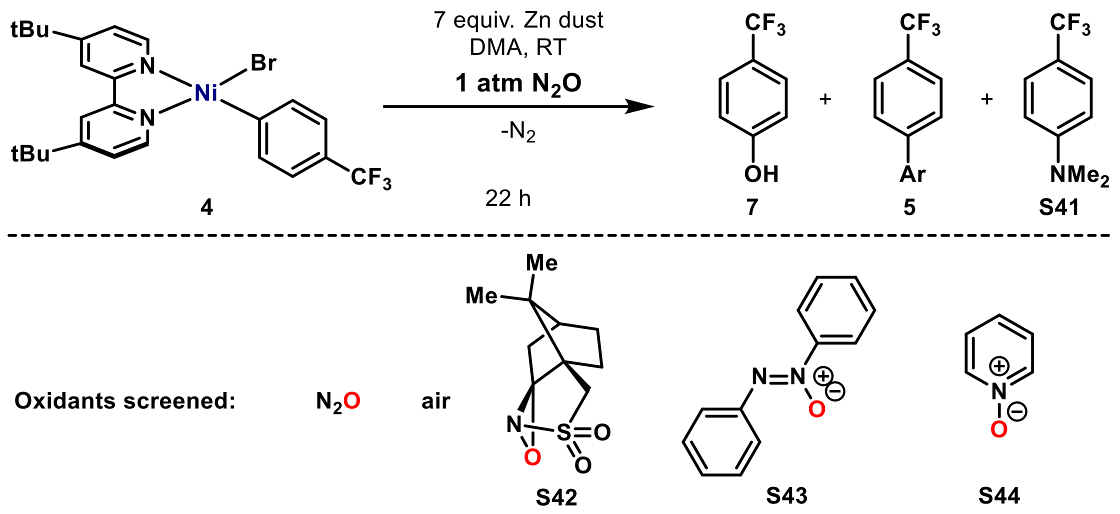

Entry	Change	Phenol (%)	Dimer (%)	Amine (%)	Remarks
1	No Zn	15	5	-	Very Slow
2	None	53-55	< 5	13-15	Slow but full conversion
3	Under air	47-50	Traces	traces	Turned Light green in 2 h
4	Under Argon	-	Major	N.D.	Turned black in 30 min
5	3 equiv. Zn<br>1.1 equiv **S42**<br>**no N_2O**	5%	40%	-	Oxaziridine not compatible with Zn
6	3 equiv. Zn<br>1.1 equiv **S43**<br>**no N_2O**	-	N.D.	N.D.	Not oxidizing enough
7	3 equiv. Zn<br>2 equiv. **S44**<br>**no N_2O**	<5%	Major	-	No phenol observed (with and without NaI)
8	Only work-up	< 5	Major	-	Dimer/Protodemetallation

Table S1. Control experiments in the stoichiometric reactivity. N.D.: not determined. ^{1}H NMR yields are given.

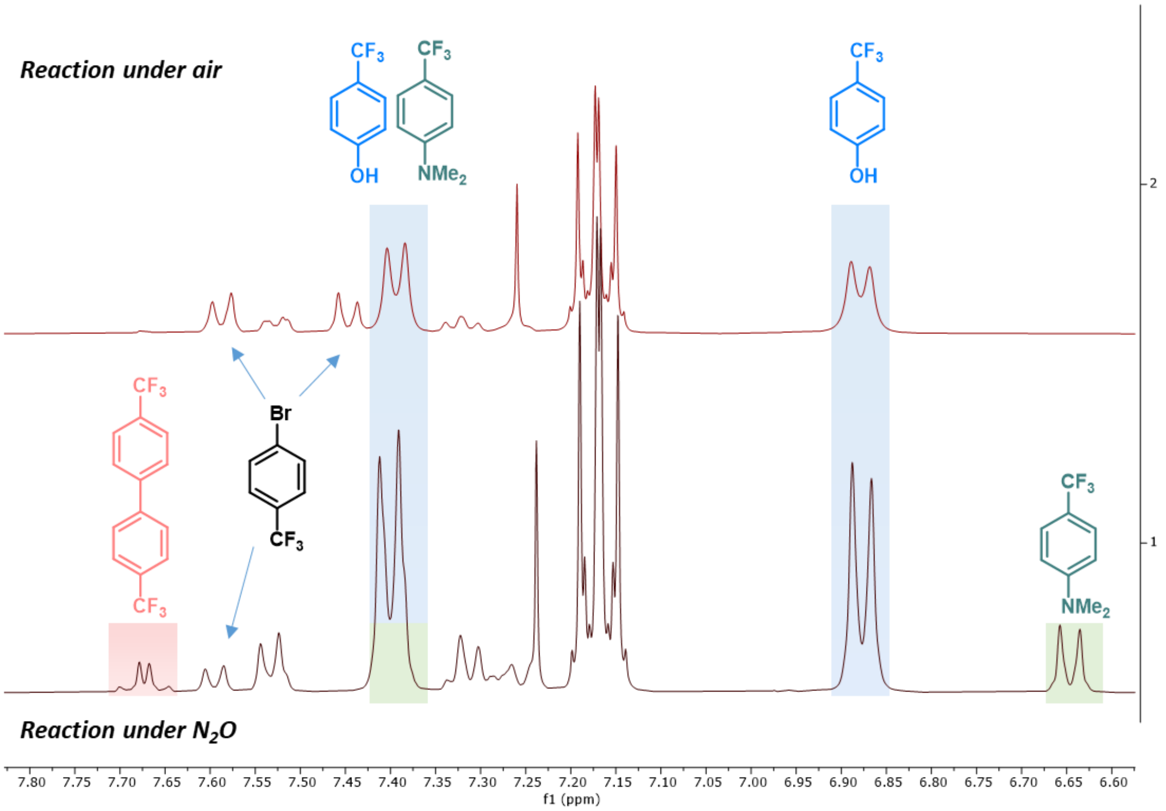

Fig. S2. Comparison of product distribution for the reaction under air (*top*), or under N_2O (*bottom*) after workup.

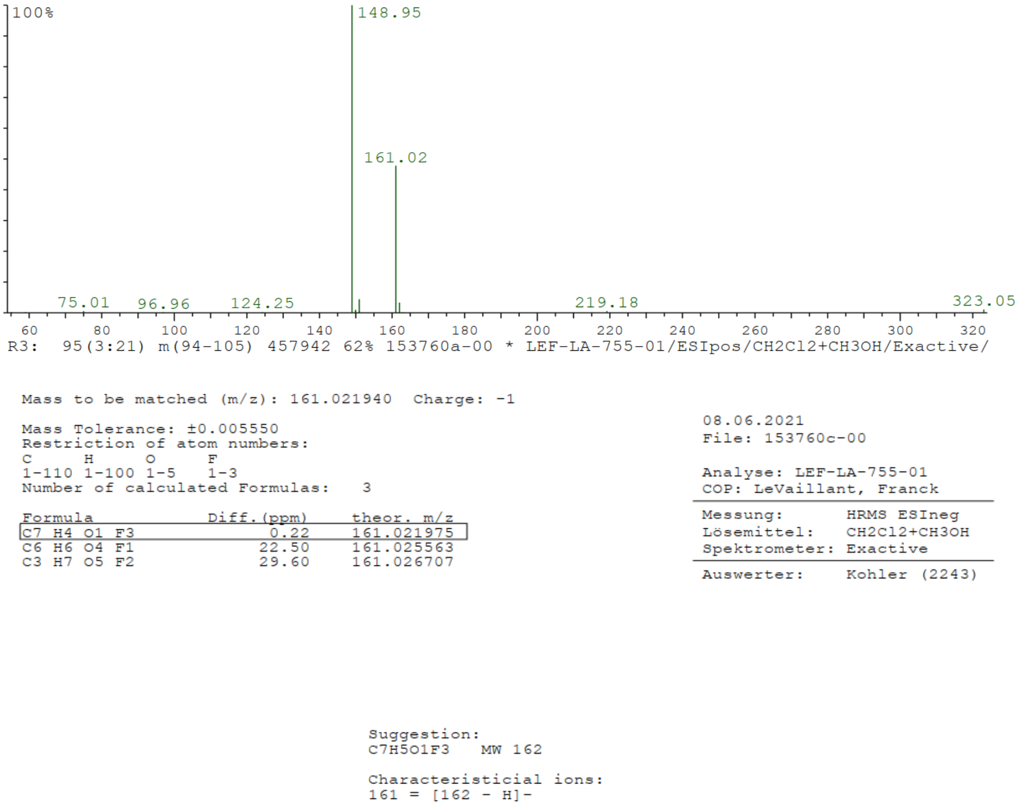

Fig. S3. Detection of the phenol **7** product in the crude by HRMS.

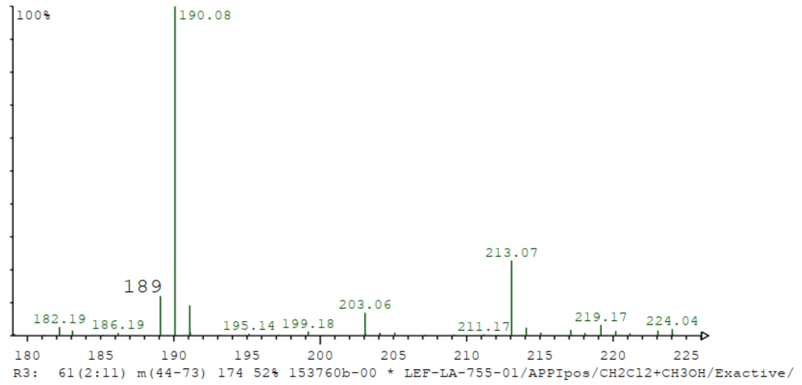

Mass to be matched (m/z): 190.083850 Charge: -1

Mass Tolerance: ±0.005550
Restriction of atom numbers:
C H N F
1-110 1-100 1-3 1-3
Number of calculated Formulas: 1

Formula	Diff.(ppm)	theor. m/z
C9 H11 N1 F3	5.57	190.084909

08.06.2021
File: 153760c-00

Analyse: LEF-LA-755-01
COP: LeVaillant, Franck

Messung:	HRMS APPIpos
Lösemittel:	CH2Cl2+CH3OH
Spektrometer:	Exactive
Auswerter:	Kohler (2243)

Suggestion:
C9H10F3N1 MW 189

Characteristicial ions:
190 = [189 + H]+

Fig. S4. Detection of the amine side **S41** product in the crude by HRMS.

From a reaction in a J-Young tube charged with **4** (7.5 mg, 13.6 μmol), in 0.6 mL DMF-*d*$_7$ at room temperature under about 1.5 bar of N_2O, a kinetic profile was run over >30 h (Fig. S5). Setting the concentration at 100% for the integration of complex **4** at t = 5 min, we obtained the graphic depicted in figure S6. This kinetic profile highlights that there is still some starting material **4** present in the reaction mixture after >30 h. This kinetic profile indicates also that decomposition of the nickel complexes occurs, and therefore the phenol formation (approx. 20% after 30 h) is much lower than the conversion of the starting material (approx.70% after 30 h). The final yield of the reaction after standing for more than a month in the J-Young tube was found to be approximatively 13%. (Addition of 6.9 mg of dibromomethane as internal standard, shaking, then transferring in another NMR tube for measurement at the 300 MHz NMR.)

Unfortunately, with zinc powder it is not possible to run such experiment due to the very heterogeneous mixture preventing both reproducible reactivity and analysis.

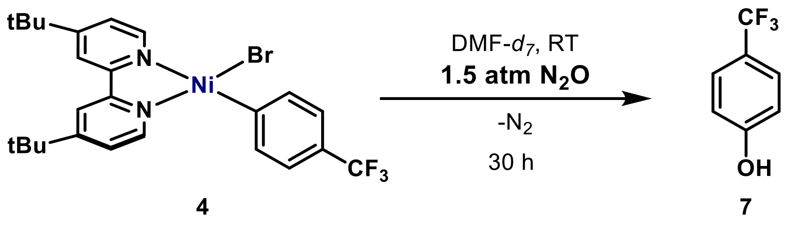

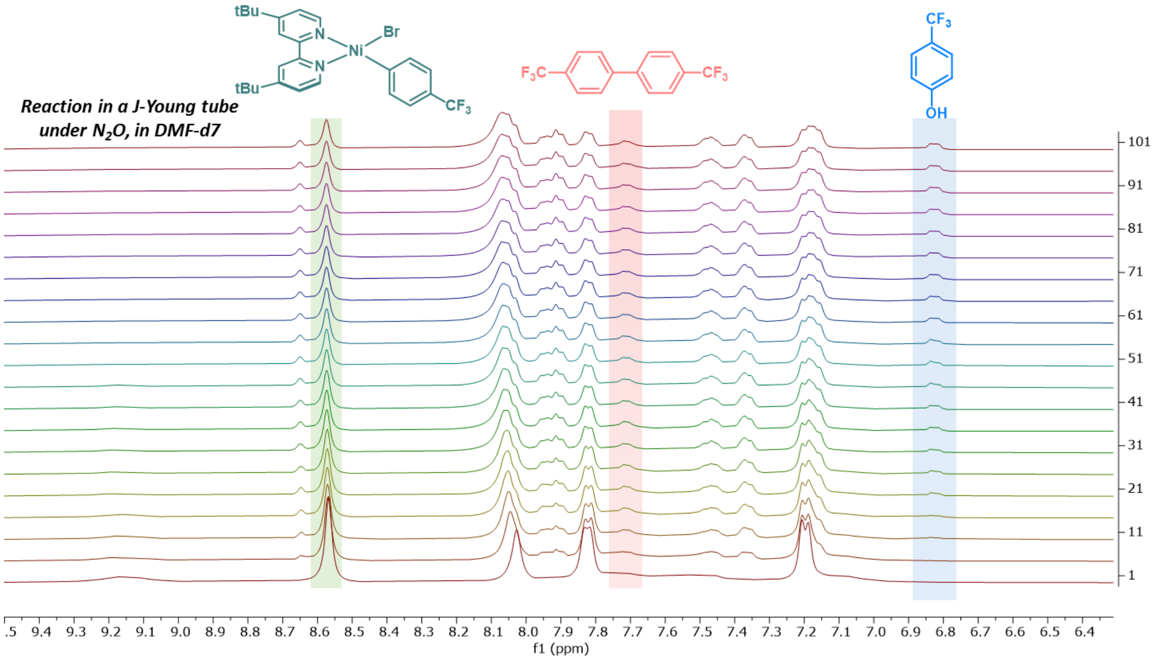

Fig. S5. Kinetic profile revealing slow production of phenol compared to the conversion of the starting material, during the reaction under N_2O in DMF-d_7, without reducing agent.

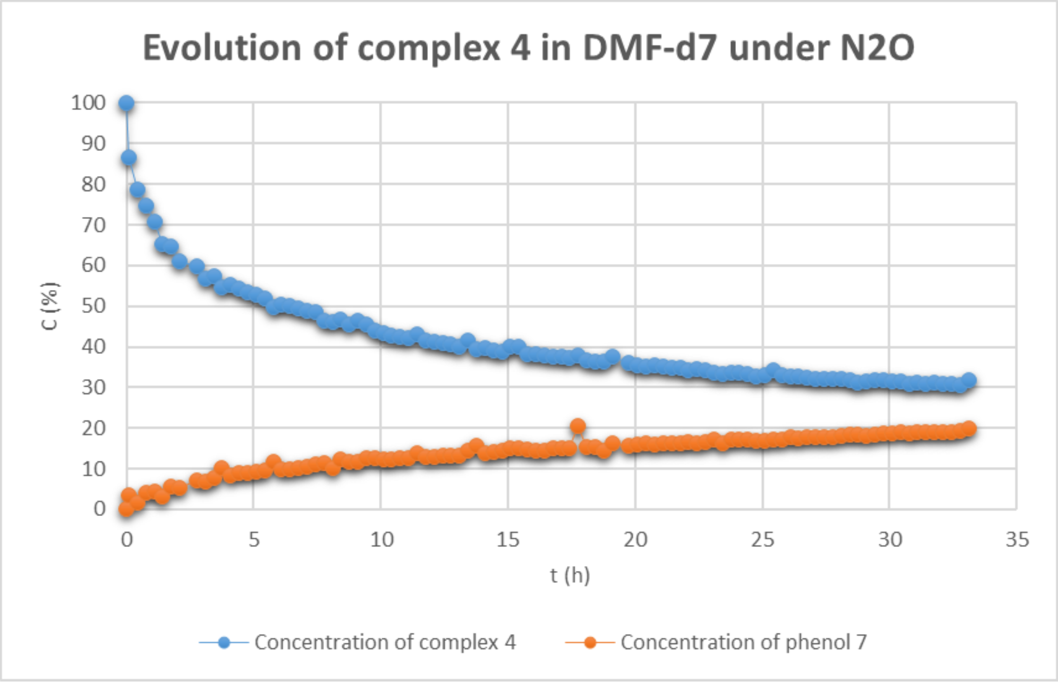

Fig. S6. Kinetic ^{1}H NMR revealing slow production of phenol during the reaction of **4** under N_2O in DMF-d_7, without reducing agent.

4.2. Color evolution

In a typical experiment with excess of Zn in DMA, the strong orange color of oxidative addition Ni(II) complex **4** diminished over time, yielding a light green solution after 20 h (Fig. S7).

Same reaction under argon atmosphere instead of N_2O turned black in 30 min.

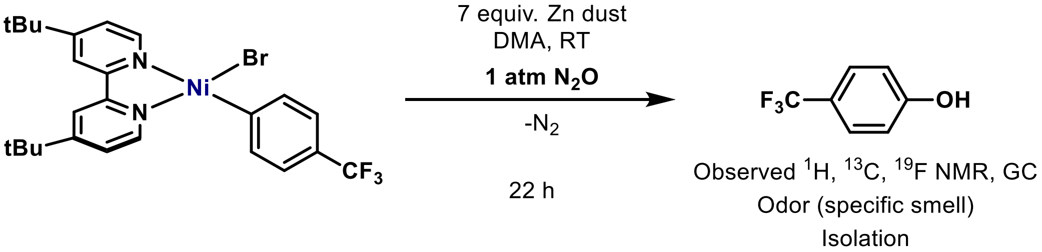

4 **[strong orange]** **7** **[light green solution]**

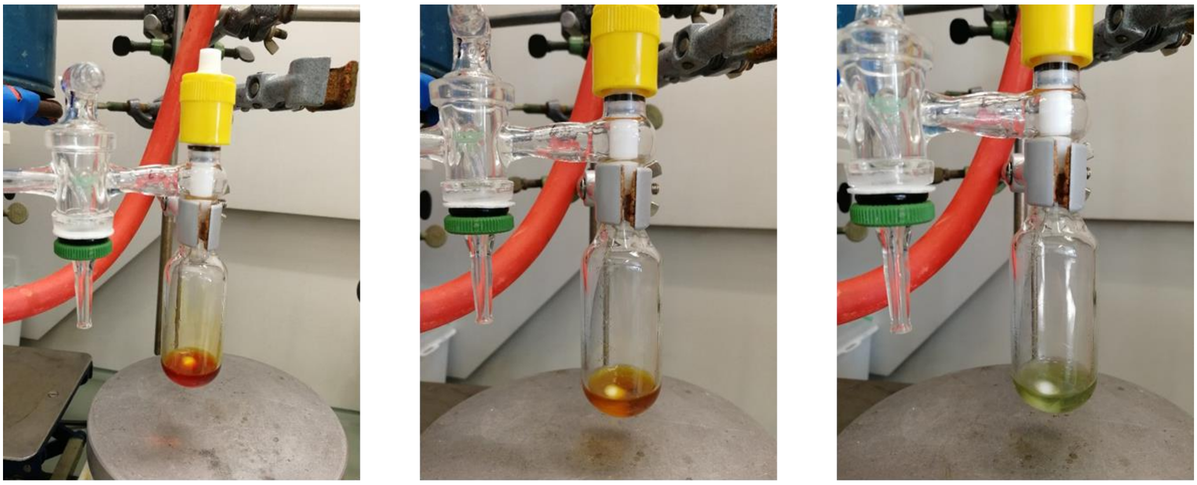

Start
5:00 pm day before

After 16h30
9:30 am

After 20h
1:00 pm

Fig. S7. Color evolution of the stoichiometric reductive phenol synthesis.

4.3 Optimization and screening of other reducing agents

Procedure used for stoichiometric reactions using bipy supported Ni(II) complex

The optimization of the stoichiometric reaction with complex **4** was developed according to the following procedure. Oven- and heatgun-dried pressure Schlenks with Teflon screw-cap and equipped with Teflon-coated stir bar were used. Two sizes were used (12 mL and 25 mL of headspace available), without any major differences noticeable. These Schlenks were brought to an argon-filled glovebox, along with a heatgun dried vial. Dry DMA (or appropriate dry and degassed solvent) was introduced to the vial. Ni complex **4** (26 mg, 47 μmol, 1.0 equiv), reducing agent and additive were introduced in the pressure Schlenk. Then, outside the glovebox, using a T-connection, the Schlenk was evacuated and refilled with N_2O. This procedure was repeated three times. Then, under N_2O flow, the Schlenk was opened and DMA (or other solvent) was added using a syringe *while stirring*. *(Stirring is important to avoid formation of agglomerates.)* The Schlenk is then closed, and the pressure of N_2O is adjusted to the indicated pressure. The reaction mixture was kept stirring at 1000 rpm, for 22 h, at RT.

After the reaction, the Schlenk is carefully opened, and the reaction was first diluted using 1-2 mL of Et_2O. The mixture is quenched first with water (the quenching can be exothermic) followed by HCl (1 M), and extracted 3 times with Et_2O (12 to 15 mL). The combined organic layers were washed with brine, dried over $MgSO_4$, filtered and concentrated under reduced pressure. The crude is analyzed by ^1H NMR and ^{19}F NMR using 4-fluoronitrobenzene as internal standard.

Comments

Yields varied depending on the reducing agents under N_2O atmosphere (Table S2). Interestingly, heterogeneous metallic reductants gave better yield toward C-O formation (entries 1-4), while homogeneous TDAE gave higher yield under air (entries 6-8). Interestingly, the use of Lewis acids also afforded phenol (entries 9-11). This is attributed to the assistance in the formation of the Ni(I) via a cleaner decomposition pathway via halogen abstraction. However, we can not discard that it exists a beneficial effect in the N_2O activation assisted by LA-coordination.[69] In an experiment with 20 min of bubbling N_2O at the very beginning of the reaction allowed a 10% increase in phenol yield (entry 14). This result indicated that saturation of the solvent with N_2O is important, and therefore we tested to use higher pressure. Indeed, further improvement was obtained in presence of 1 equiv NaI under 1.9 atm of N_2O, affording phenol in 73% yield (entry 15).

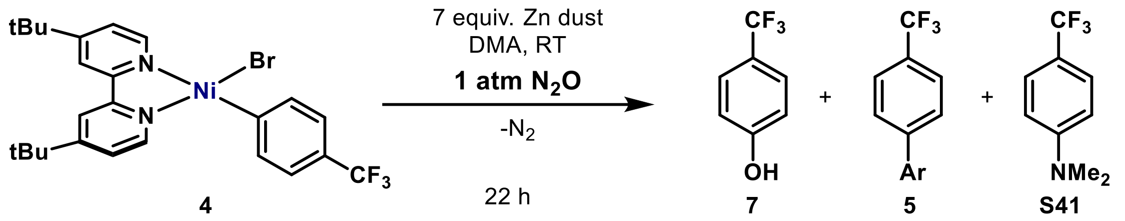

Entry	Change	DMA	Phenol (%)	Dimer (%)	Amine (%)
1	None	0.023 M	53-55	< 5	13-15
2	Zn (3)	0.047 M	56-59	traces	8
3	Zn (3)	0.094 M	55-60	traces	4
4	Mn (3.8)	0.047 M	52-55	traces	5
5	Cp_2Co (1.1)	0.047 M	8	30	Traces
6	TDAE (2)	0.047 M	32-34	traces	traces
7	TDAE (2) / air	0.047 M	66-70	traces	traces
8	TDAE (2) / air	THF 0.047 M	61-70	traces	traces
9	$ZnBr_2$ (1.1)	0.047 M	35-40	40	8
10	$ZnOTf_2$ (1.1)	0.047 M	10	10-20	15
11	$ZnOTf_2$ (0.3)	0.047 M	30-35	10-20	15
12	$CoCp^*_2$ (1.1)	0.047 M	15	45	2
13	$CoCp^*_2$ (1.1) + $ZnBr_2$ (1.1)	0.047 M	30-35	30	3
14	Zn (3) + N_2O bubbling 20 min	0.047 M	62-68	30	3
15	Zn (3) + NaI (1)	0.047 M	73	traces	7

Table S2. For reducing agent, Lewis Acid or NaI, in bracket is the number of equivalents used. ^{1}H NMR yields are given. *N.B.: Yield of dimer is calculated out of 100%.*

To explain the observed reactivity, we suggested that in a polar solvent such as DMA, complex **4** is unstable and susceptible to undergo ligand exchange (Fig. S8). Such exchange will generate stable $LNiBr_2$ and unstable $LNiAr_2$ (**A**). The latter can then undergo reductive elimination to make the biaryl side product (**5**) and a Ni(0) species. The low valent Ni(0) can now comproportionate, either with $LNiBr_2$ to make LNiBr or $[LNiBr]_2$; or with LNiArBr (**4**), generating a potentially reactive Ni(I)-Ar species (**B**) and LNiBr. Protodemetallation, N_2O reaction or DMA activation may result from this metalloradical intermediate LNi(I)–Ar (**B**). The formation of the dimethyl amino-side product **S41** is unclear at the moment; however, it should be due to DMA activation by low valent Ni complexes, or hydrolysis through a Ni-oxo type of intermediate.

It is possible that LNiBr catalyzed the decomposition of the starting material, thus regenerating LNi(I)-Ar (**B**) without "high" formation of the biaryl dimer **5**. The presence of a reducing agent accelerates the overall process by direct formation of the Ni(I)–Ar (**B**), therefore affording cleaner reactivity pattern.

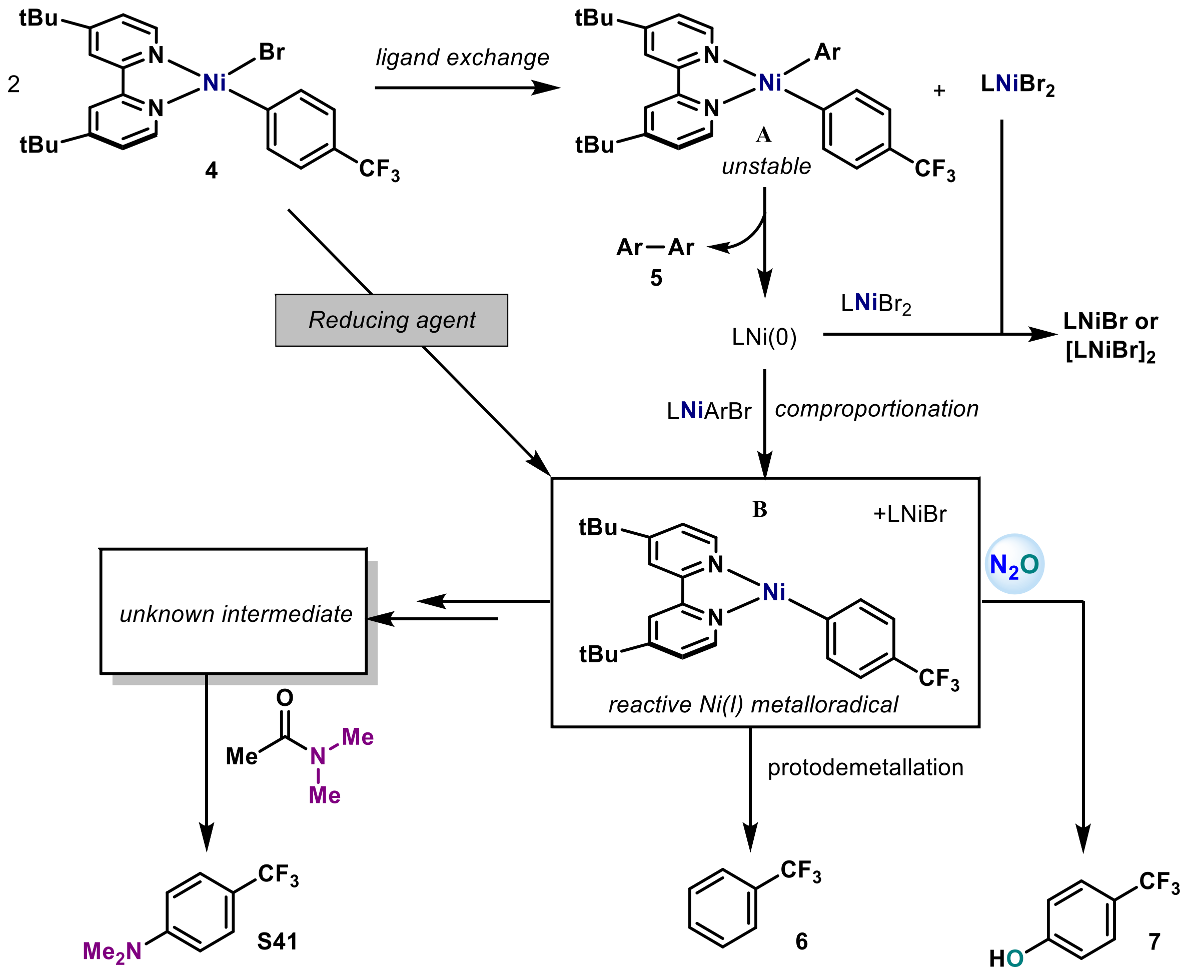

Fig. S8. Suggested mechanism for the reaction of **4** under N_2O in DMA, with/without reducing agent.

Further discussion about Cp*$_2$Co and the compatibility of the different partners

As low yields are obtained using strong reducing agent Cp*$_2$Co (Table S2, entries 12-13), we considered that potential side reactivity could occur with N_2O.

In the following publication, Mankad and coworkers stated that Cp_2Co does not directly react with N_2O.[70] This could be indeed attributed to the relatively low reduction potential of Cp_2Co (E^0 = –0.57 V in DMF vs SHE).[71] On the other hand, Cp*$_2$Co is a much stronger reductant (E^0 = –1.16 V in DMF vs SHE),[71] and therefore 1-electron reduction of N_2O may be within reach

($E^0(N_2O/N_2)$ = +1.77 V vs SHE, 2-electron reduction).[72] Although direct comparison of the 1-electron vs 2-electron reduction potentials should suggest that no reaction occurs, experimental evidence points otherwise. In addition to these observations, in the literature, Cp^*_2Co has also been used as electron source for the reduction of N_2O in combination with Lewis acid.[69] Altogether these data suggest that care must be taken in drawing conclusions from the 2-electron redox potential of N_2O.

Based on these precedents, a control experiment was performed.

Inside a glovebox, Cp^*_2Co (13 mg) was introduced in a dry pressure Schlenk equipped with a Teflon coated stir bar. Then, outside the glovebox, under N_2O atmosphere, dry and degassed solvent (1 mL) was added to the Schlenk and the reaction mixture was stirred overnight. This experiment was performed both using THF and DMA as solvent. (*Light was turned off in both the glovebox and fumehood, and the Schlenk were protected from light using aluminum foil.*)

Interestingly the color of both solutions changed quickly: from brown to orange to deep reddish purple in DMA (Fig. S10); and from brown to yellow to green in THF (Fig. S11). On the other hand, it was controlled that Cp^*_2Co under argon is stable upon time: the brown color of pure material was kept intact while stirring (in DMA or THF) at room temperature under argon for 24 h (Fig. S12).

The headspace analysis of a reaction starting with more material was performed. A dried vial was charge with Cp^*_2Co (33 mg, 0.1 mmol) in 1.0 mL of THF or DMA, and placed under 1.5 bar of N_2O. After stirring for 16 h at room temperature, the headspace was analyzed by GC-TCD (*see section 9 for more details on the method*). Such measurement revealed the formation of significant amount of N_2 (30% of the total integration in DMA (Fig. 9), 40% in THF).

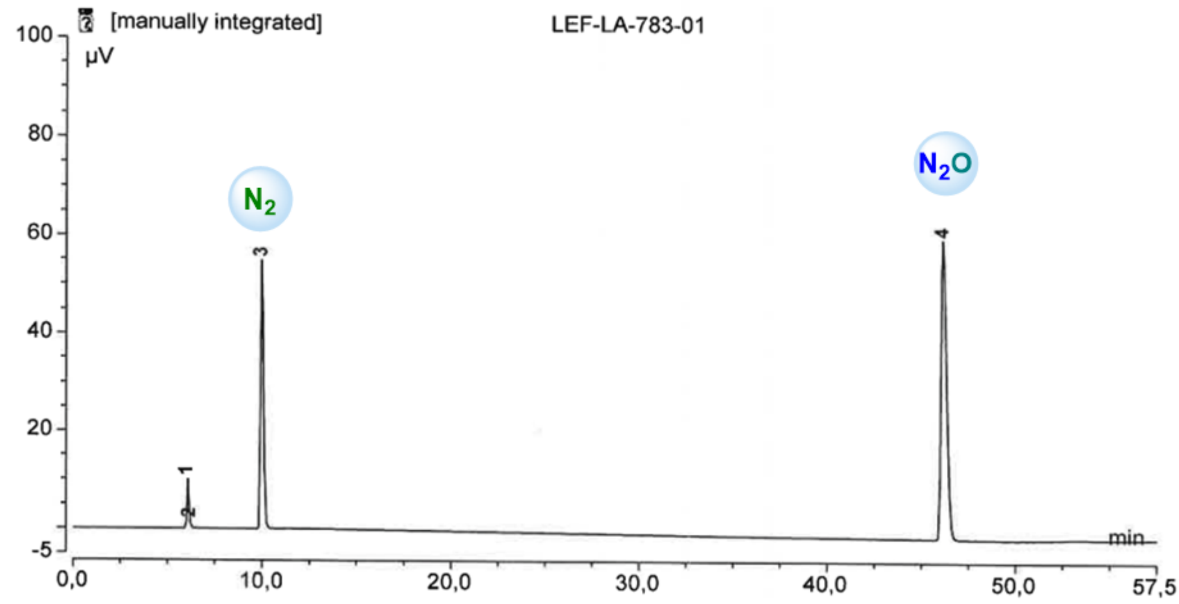

Sample:	**LEF-LA-783-01**	Instrument:	**GC_113**
Sequenz:	**8335 LEF-LA VD**	Measured:	**16.07.21 15:13**
Sequenz date:	**16.07.21**	Processing M.:	**LEF-LA**
		Report-File:	**783-01**

Zuordnung siehe PAY-PA-642-01 20/7477 und LEF-LA-442-01 20/7680

No.	Ret.Time min	area-% %	Peak Name
1	6,07	3,27	Argon (keine Basislinientrennung)
2	6,18	0,31	O2 (keine Basislinientrennung)
3	9,98	30,52	N2
4	46,13	65,90	N2O

Fig. S9. Headspace analysis by GC-TCD of a solution of $Cp*_2Co$ in DMA under N_2O.

Overall, these control experiments highlight the fact that a judicious choice of reducing agent is crucial in order to get good reactivity, and that excellent compatibility of precursor for low-valent species, reductant and oxidant is not trivial. The current system with Ni/Zn/NaI/N_2O is therefore promising for achieving catalytic organometallic Baeyer-Villiger (OMBV).

The full analysis of the product distribution is outside the range of this manuscript; however, other (decamethyl)metalocenes are known to react with N_2O under mild conditions, such as $Cp*_2V$ and $Cp*_2Ti$.[73]

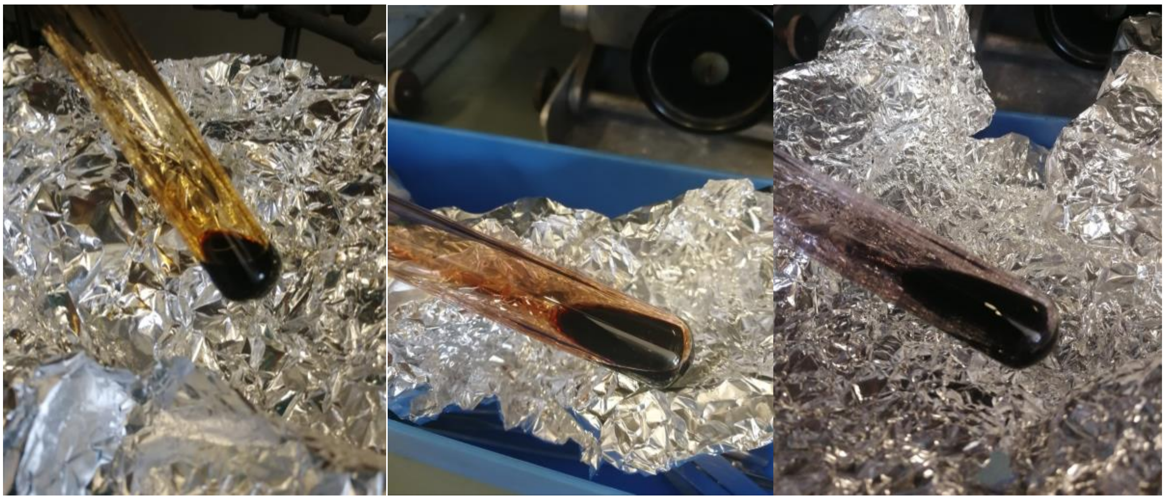

Fig. S10. Solution of Cp^*_2CO in DMA under N_2O. (*left*, t = 2 min; *middle*, t = 10 min; *right*, t = 90 min).

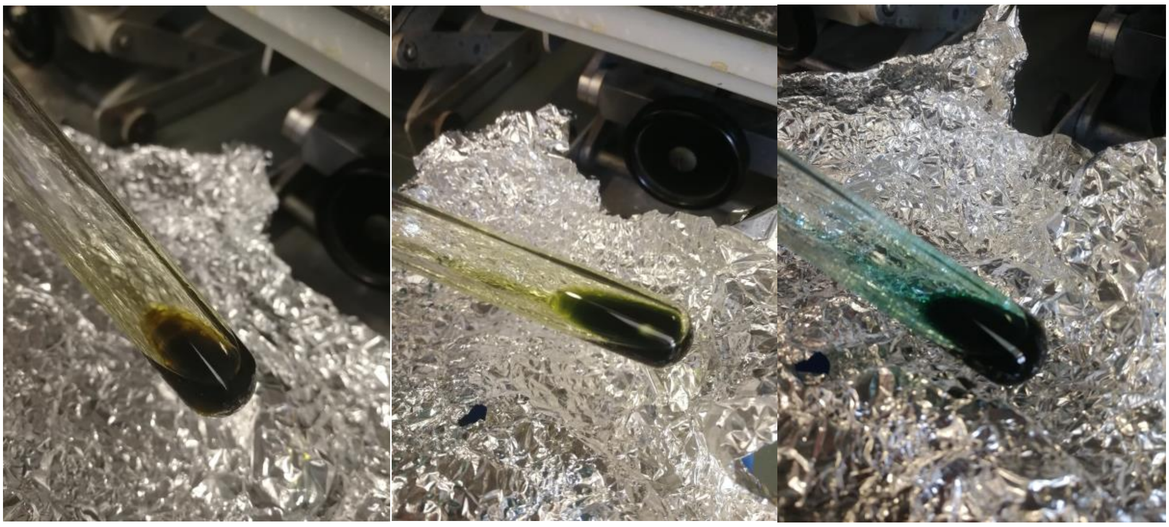

Fig. S11. Solution of Cp^*_2CO in THF under N_2O. (*left*, t = 2 min; *middle*, t = 10 min; *right*, t = 90 min).

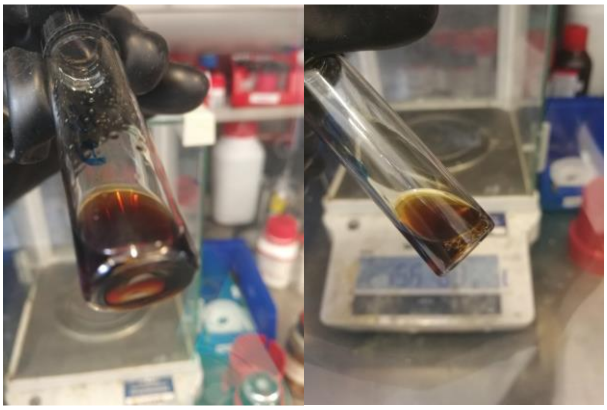

Fig. S12. For comparison, the color of solution of pure Cp^*_2Co in DMA (*left*) and THF (*right*) under argon (inside a glovebox).

4.4. Reactions with tridentate [LNi(II)–Ar]X complexes

4.4.1. Procedure used for stoichiometric reactions using terpy supported Ni(II) complex

The stoichiometric reactions with complex **11** were developed according to the following procedure. Oven- and heatgun-dried pressure Schlenks with Teflon screw-cap and equipped with Teflon-coated stir bar were used. These Schlenks were brought to argon filled glovebox, along with a heatgun dried vial. Dry DMA (or appropriate dry and degassed solvent) was introduced to the vial. Ni complex **11** (12.3 mg, 25.0 μmol, 1.0 equiv), zinc (4.9 mg, 75 μmol 3.0 equiv) and NaI (3.7 mg, 25 μmol, 1.0 equiv) were introduced in the pressure Schlenk. Then, outside the glovebox, using a T-connection, the Schlenk was evacuated and refilled with N_2O. This procedure was repeated three times. Then, under N_2O flow, the Schlenk was opened and 0.5 mL of DMA was added using a syringe *while stirring. (Stirring is important to avoid formation of agglomerates.)* The Schlenk is then closed, and the pressure of N_2O is adjusted to the indicated pressure. The reaction mixture was kept stirring at 1000 rpm, for 22 h, at RT.
After the reaction, the Schlenk is carefully opened, and the reaction was first diluted using 1-2 mL of Et_2O. The mixture is quenched first with water (the quenching can be exothermic) followed by HCl (1 M), and extracted 3 times with Et_2O (5 – 6 mL). The combined organic layers were washed with brine, dried over $MgSO_4$, filtered and concentrated under reduced pressure. The crude is analyzed by ^1H NMR and dibromomethane (8.6 mg, 49.5 μmol, 1.98 equiv) as internal standard.

For this reactivity screening, we choose only the best conditions from Table S2 in presence of Zn. Complex **11** is known to be air-stable in the solid state, and is also a more robust complex in solution compared to complex **4** supported by a bidentate ligand. In presence of Zn and NaI, 49% of mesitol (**12**) was observed by ^1H NMR (Table S3, entry 1), and GC-MS confirmed the formation of **12** (Fig. S13-14). The other results (entries 2-5) revealed that for this system (tridentate ligand, bulky aryl group) low reactivity is obtained in the absence of NaI. Such additives (like LiCl and other halogenated sources) are usually crucial in catalytic reactions under reductive conditions with heterogeneous reductants.[71,74] Importantly, in this case, the use of Lewis-acid does not allow the formation of mesitol (**12**) as confirmed by GC-MS (Fig. S15). This is in line with the low reactivity of Ni(II) complex versus N_2O, and the need for a specific reducing agent to promote the reaction.

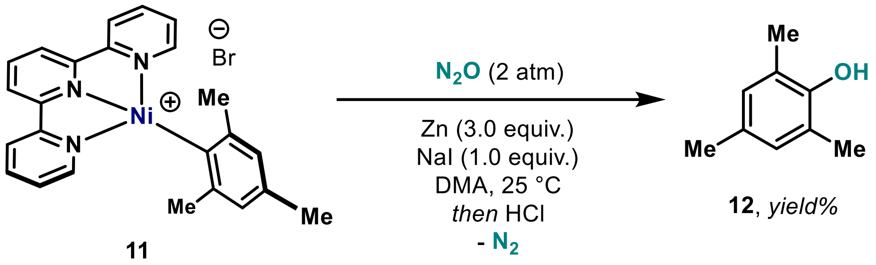

Entry	Change	Phenol (NMR yield, %)
1	None	49
2	Without NaI, under 1 atm N_2O	0
3	Without NaI, using air instead of N_2O	5
4	$ZnBr_2$ (1.1), no Zn, no NaI	0
5	$ZnOTf_2$ (0.3), no Zn, no NaI	0

Table S3. Stoichiometric reactivity of **11** for the C-O bond formation.

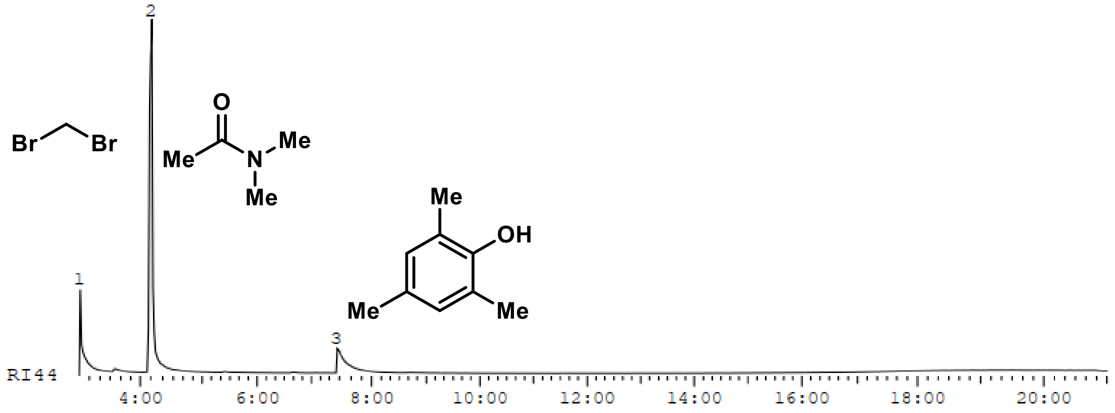

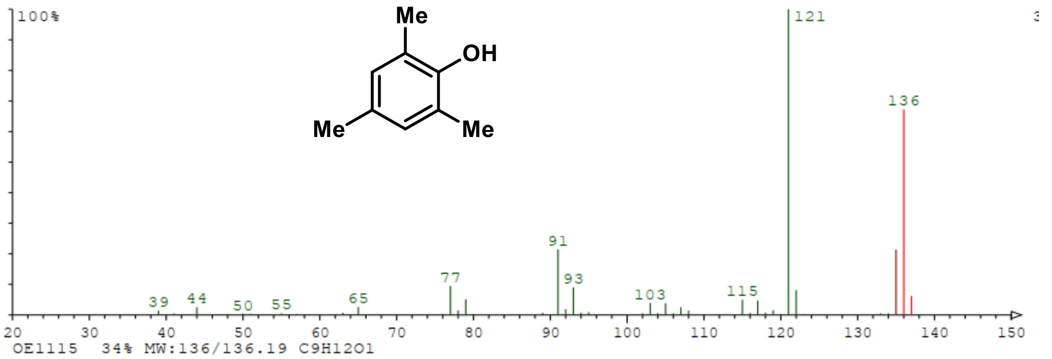

34	0.02	60	0.01	93	9.00	121	100.00
35	0.01	61	0.04	94	0.80	122	8.15
36	0.01	62	0.24	95	0.92	123	0.33
37	0.03	63	0.72	96	0.03	128	0.02
38	0.06	64	0.05	98	0.02	130	0.03
39	1.43	65	2.64	102	0.56	131	0.22
40	0.10	66	0.09	103	3.95	133	0.55
41	0.48	67	0.26	104	0.57	134	0.52
43	0.14	73	0.06	105	3.83	135	21.36
44	2.57	74	0.30	106	0.55	136	67.25
45	0.25	75	0.23	107	2.53	137	6.22
46	0.04	76	0.39	108	1.44	138	0.22
50	0.48	77	9.51	109	0.28		
51	0.43	78	1.52	113	0.01		
52	0.07	79	5.11	114	0.02		
53	0.16	80	0.35	115	4.99		
54	0.05	81	0.15	116	0.63		
55	0.54	89	0.70	117	4.71		
56	0.10	90	0.19	118	0.77		
57	0.39	91	21.45	119	1.59		
59	0.02	92	1.93	120	0.33		

OE1115 34% MW:136/136.19 C9H12O1

Fig. S13. GC-MS allowed the detection of mesitol (**12**) in the crude mixture.

```
Mass to be matched (m/z): 136.088480   Charge: 1

Mass Tolerance: ±0.050000
Restriction of atom numbers:
C      H      O
1-110  1-100  1-1
Number of calculated Formulas:    1

Formula            Diff.(ppm)    theor. m/z
C9 H12 O1              -1.58     136.088265
```

```
19.01.2021
File: 151259a-00.raw
Analyse: LEF-LA-566-01
COP: LeVaillant, Franck
Messung:        GC-MS
Ionisierung:    GC EI
Spektrometer:   QExactiveGC
Säule:          MS 50 RTX1+VS
Länge:          30+7
Temp.:          35-10-285-5
GC-Nr.:         -
MS-Nr.:         29199
Auswerter:      Margold (2242)
```

Fig. S14. HRMS confirmation of the mesitol (**12**) in the crude mixture.

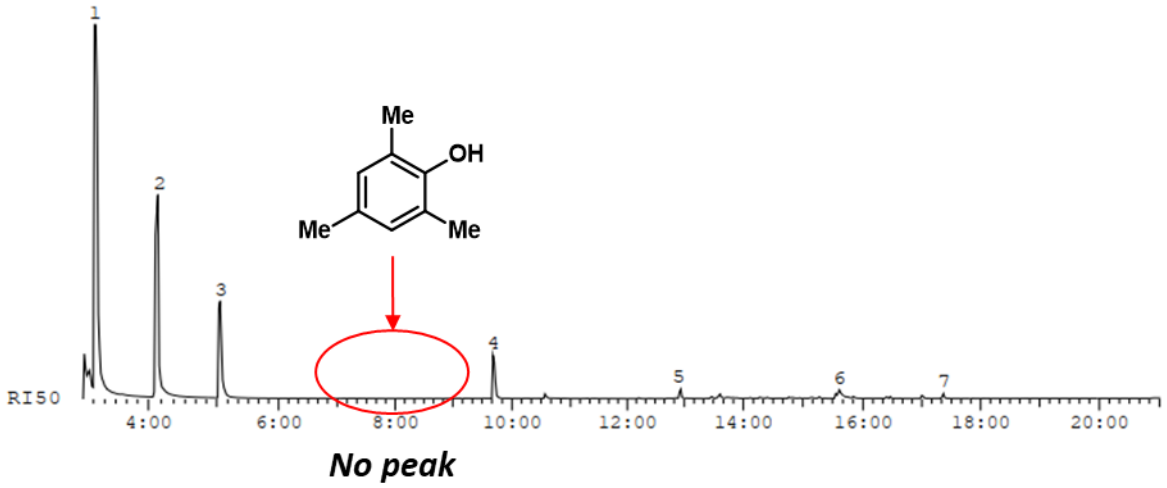

Fig. S15. GC-MS spectra showed no mesitol (**12**) in the reaction using $ZnBr_2$ instead of Zn/NaI system.

The reaction of [terpyNi–Mes]Br **11** in presence of Zn (2 equiv), NaI (2 equiv) in DMF-d_7 under N_2O was analyzed directly by transferring the reaction mixture in a J-Young tube. Two sets of paramagnetic peaks were observed, meaning that several paramagnetic Ni complexes were present (Fig. S16). One set of peaks is very similar to the known terpyNi–Br (**S45**) (with also a very broad peak found at +130 ppm in both spectra).[53] However, in presence of NaI, it might be terpyNi–I instead, or a mixture of both. The second set of peaks is unknown at this stage.

The formation of a Ni(I)–X species at the end of the transformation is consistent with a O–insertion into a Ni(I)–C bond followed by salt metathesis with NaI or NaBr. Such salt metathesis were also proposed in the mechanism of carboxylation reaction.[71]

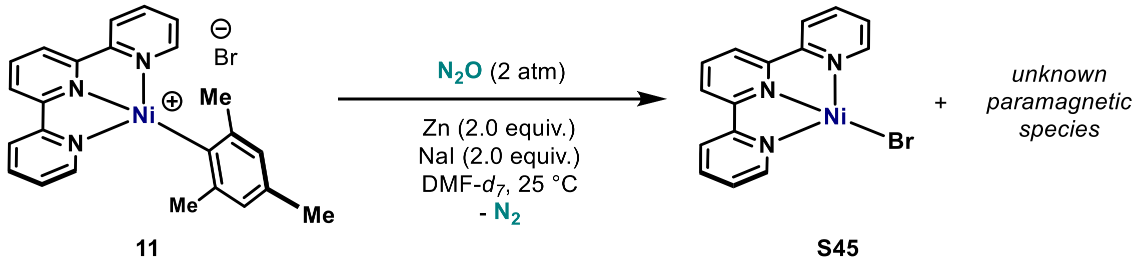

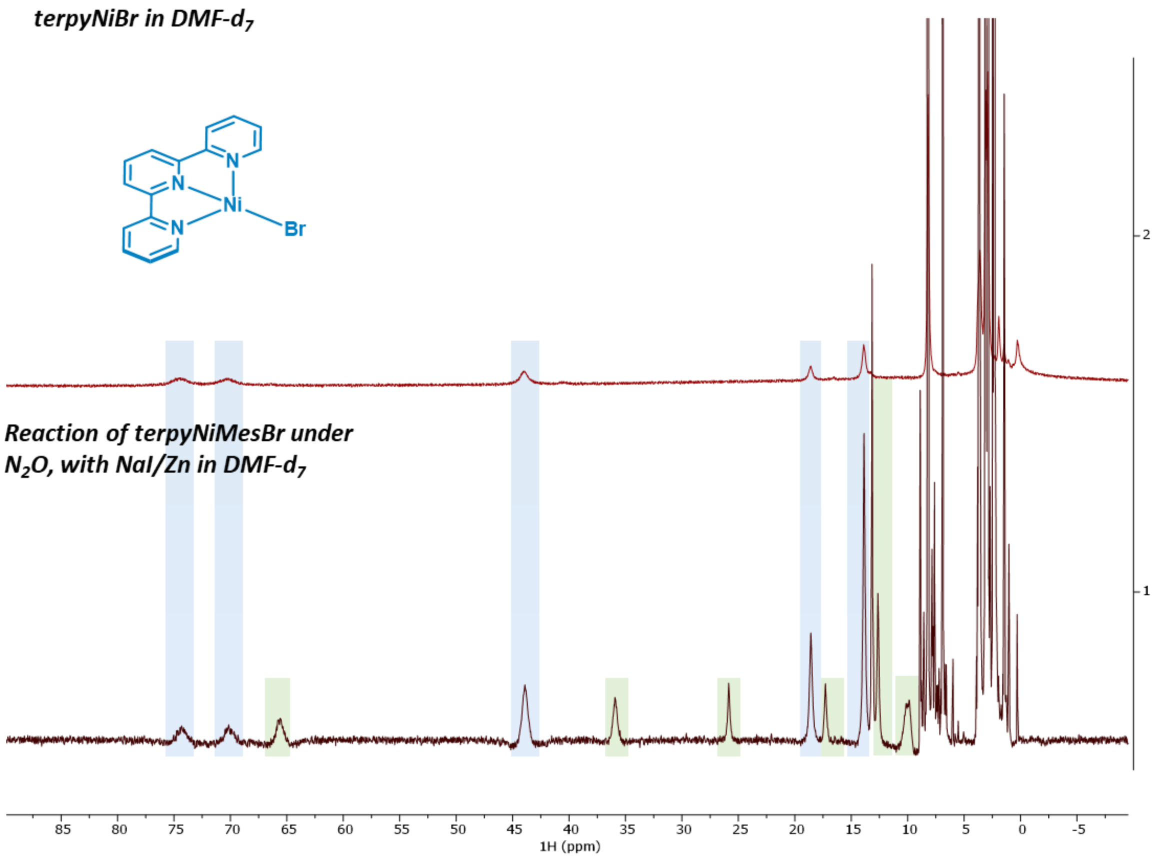

Fig. S16. Comparison of the ^{1}H NMR spectrum of terpyNi–Br (**S45**) (*top*) with the paramagnetic ^{1}H NMR spectrum of the reaction of **11** in DMF-d_7 under N_2O using NaI/Zn system (*bottom*).

4.4.2. Formation of terpyNi(I)–Mes (S46) by chemical reduction and EPR analysis

Zinc powder associated with NaI in DMA or DMF formed in-situ extra small zinc particles that could not be removed upon filtration over celite and glass fiber. Such Zn particles prevent good analysis by EPR measurement due to a very strong signal of S = 1/2 species. Without NaI, the reduction of this complex is very slow, and therefore not suitable for EPR analysis of in-situ generated intermediates.

In order to get insight into the chemical formation of terpyNi(I)–Mes from the parent Ni(II) complex, we turned our attention to the use of the strongly reducing $Co(Cp^{*})_2$. The EPR analysis of the reaction mixture is reported after the procedure.

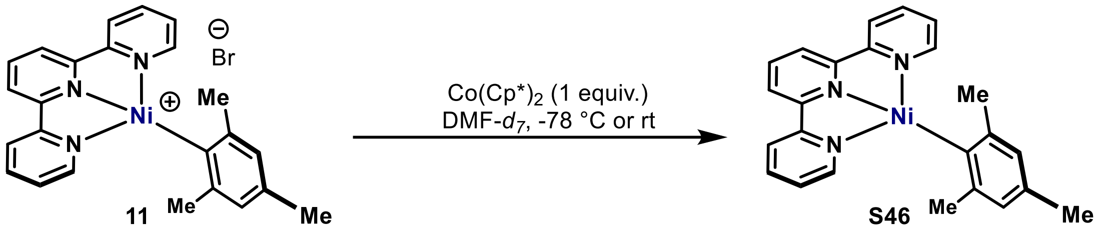

Procedure

In a glovebox, Ni complex **11** (3.0 mg, 6.1 μmol, 1.0 equiv.) and $Co(Cp^{*})_2$ (2.0 mg, 6.1 μmol 1.0 equiv) was introduced in an oven- and heatgun-dried vial equipped with a Teflon coated stir bar. Dry and degassed DMF-d_7 (0.6 mL) was added at RT and vigorously stirred for 3 to 5 min. The color changed to deep brown. Then, 150 μL was introduced to the 5 mm EPR quartz tube for X-band measurement (35 μL for 2.8 mm tubes for Q-band analysis), and the tubes were capped with adapted rubber septum, brought quickly outside of the glovebox and frozen in liquid N_2. The EPR signals are shown in figure S17 (Q-band) and figure S18 (X-band).

N.B. Repeating the same reaction in a Schlenk at –78 °C outside the glovebox gave very similar reaction mixture and EPR signal.

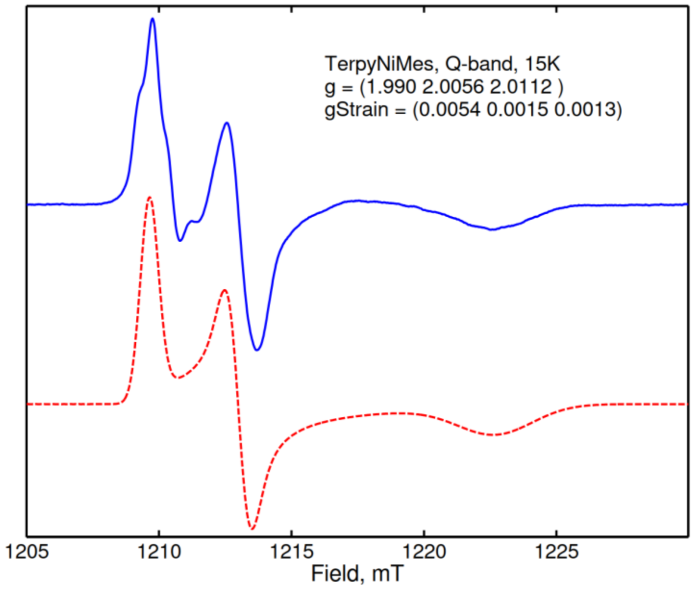

Fig. S17. TerpyNi–Mes Q-band pulse EPR (34.0513 GHz) 15 K. The spectrum was recorded in FID amplitude detection mode (π/2 pulse = 500 ns) and subsequently transformed to first derivative representation using a pseudo-modulation transformation.[48] The g-parameters were fitted using the “esfit” function in Easyspin.[75]

The g-parameters are consistent with a ligand centered paramagnetic center. The shoulders observed at the g = 2.0112 feature point to additional hyperfine interactions with ^{14}N and/or ^{1}H nuclei of the terpy ligand.

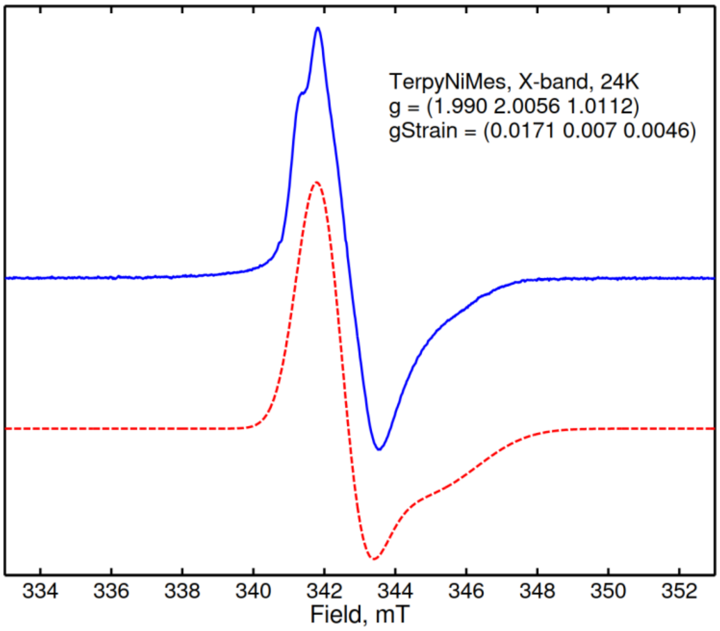

Fig. S18. TerpyNi–Mes X-band EPR (9.6207 GHz) 24 K, 2 µW. The spectrum is simulated (red curve) using the g-parameters obtained at Q-band (Fig. S17) and varying the gStrain parameters. Since the gStrain parameters are different from those used at Q-band, we have to assume that the line width and shape is strongly affected by unresolved hyperfine interactions.

Overall, these two spectra are consistent with the reported spectra of terpyNi–Mes **S46** generated upon dual electro-spectroscopy analysis.[38]

4.4.3. Formation of Ni(I)–Ar by transmetalation and reactivity with N_2O

A second approach in which a Ni(I) –Ar is prepared *in-situ* upon transmetalation was undertaken.

A characterization by EPR of the paramagnetic Ni complex (*t*Bu-terpy)Ni–I (**13**) was performed (Fig. S19). The measurement was obtained by dissolving 1 mg of **13** in 0.5 mL of dry and degassed THF-d_8.

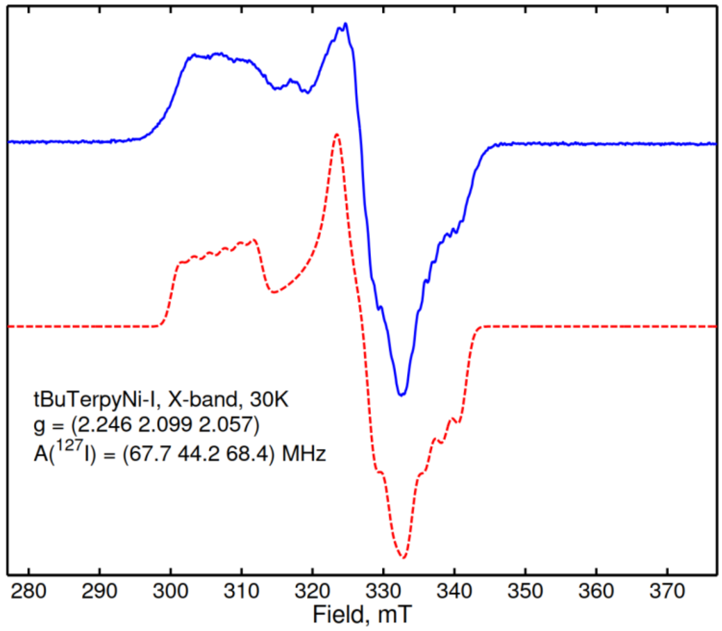

Fig. S19. (*t*Bu-terpy)Ni–I (**13**) at X-band EPR (9.6388 GHz) 30K, 0.0633 mW. Spectrum was simulated (red curve) using the "esfit" function in Easyspin[75] and assuming a dominant ^{127}I Hyperfine (I = 5/2) interaction. The strongly anisotropic g-parameters are consistent with a Ni(I) centered paramagnetic species.

Procedure

A pressure Schlenk was charged with solids (*t*Bu-terpy)Ni–I (**13**) (11.7 mg, 20 µmol, 1.0 equiv) and Ph_2Zn (3.1 mg, 14 µmol, 0.7 equiv). Then, under N_2O, the addition of THF-d_8 resulted in a quick change of color to deep yellow, which over time turned brown and less soluble. After 24 h, several drops of HCl were added, followed by addition of $MgSO_4$ and internal standard CH_2Br_2 (7.4 mg, 43 µmol, 2.14 equiv). The filtration using HPLC filter directly in a NMR tube allowed the detection of 17-20% phenol by ^{1}H NMR (Fig. S20-21). Low amount of phenyl iodide (<5%) is also observed by NMR. This mixture of phenol (**14**) and phenyliodide (**61**) is confirmed by GC-MS (Fig. S22).

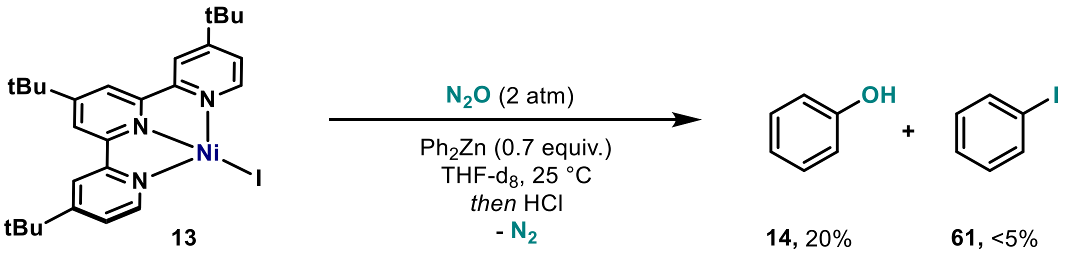

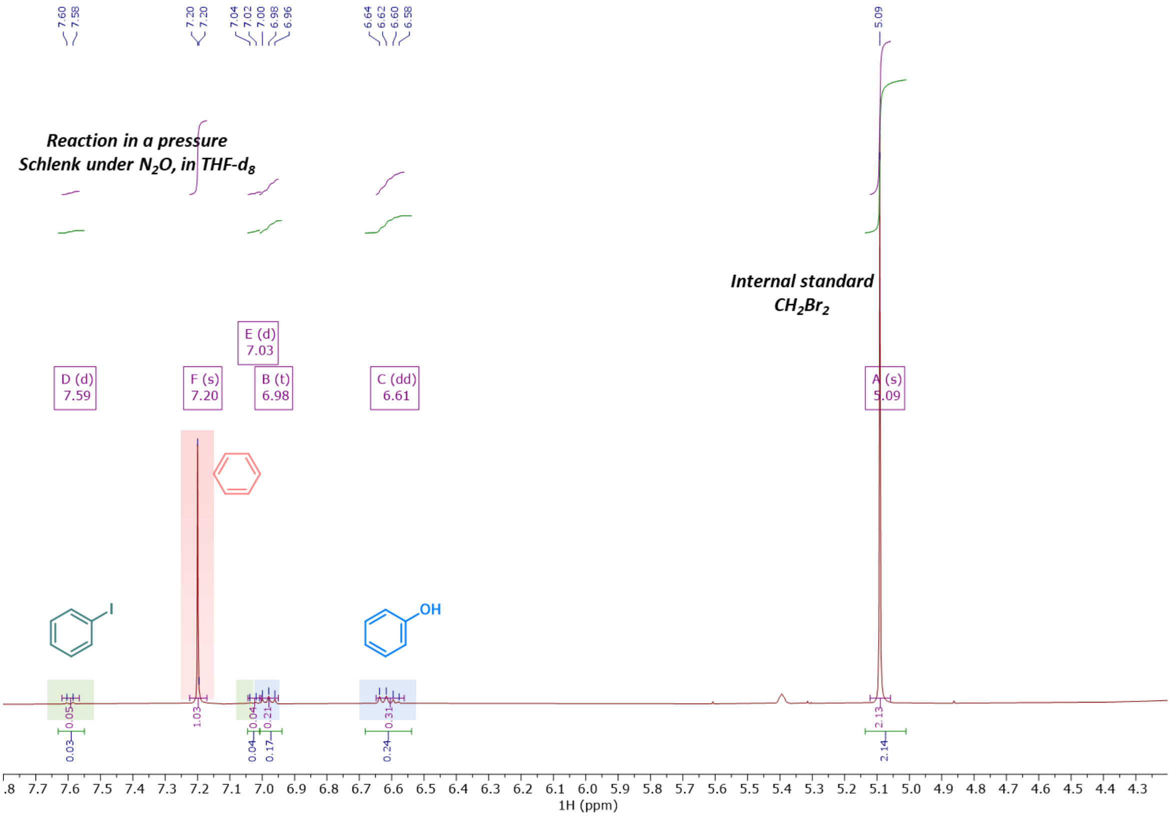

Fig. S20. NMR spectra of the crude reaction, using CH_2Br_2 as internal standard.

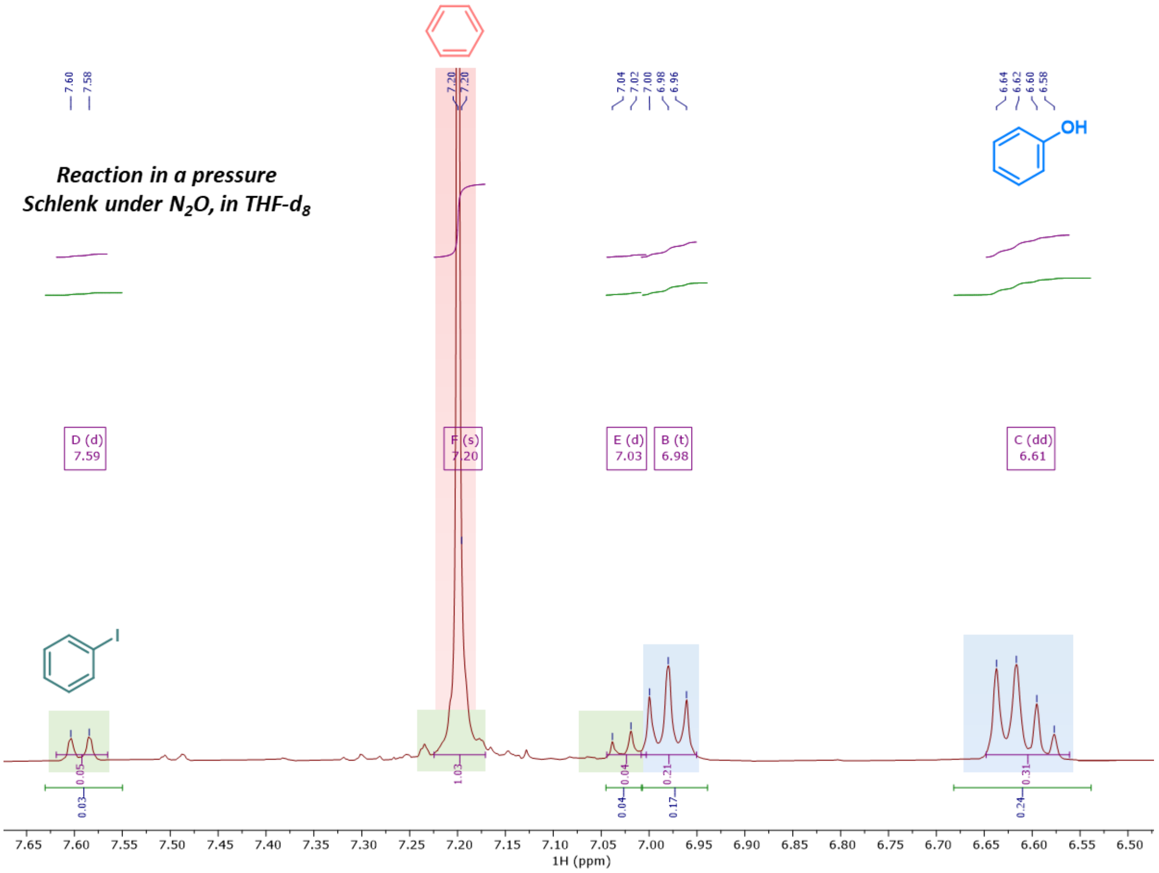

Fig. S21. Zoom on the aromatic region to observe the product distribution.

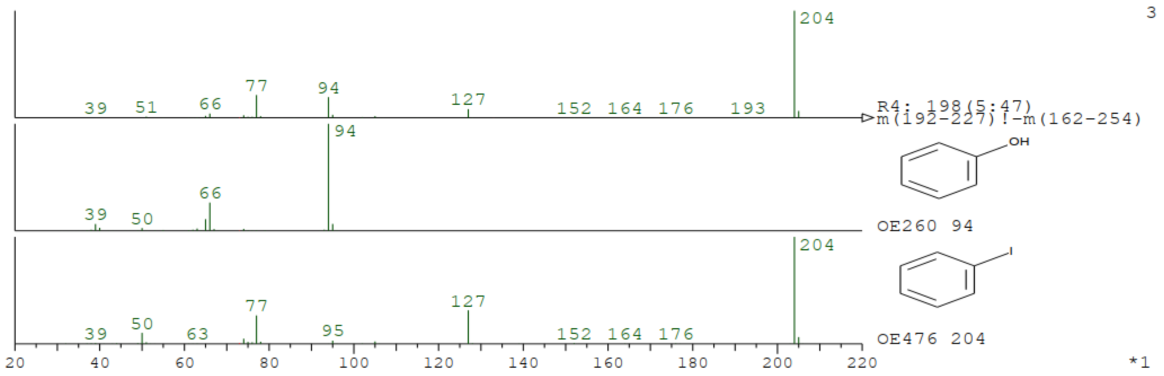

Fig. S22. GC-MS analysis of the transmetallation reaction under N_2O showing mixture of PhOH and PhI after workup.

Comments

Phenol originates most likely from the transmetallation product (*t*Bu-terpy)Ni–Ph (**A**), followed by OAT from N_2O (Fig. S23). On the other hand, phenyl iodide (**61**) could be formed upon direct attack of Ph_2Zn to the Ni–I, although it is unlikely. Another alternative is the formation of [(*t*Bu-terpy)NiPh]I complex **S47**, that could reductively eliminate upon work-up with non-degassed HCl. In fact, performing the reaction from –78 °C to RT in a J-Young tube prevented the N–O cleavage and allowed the detection of the Ni(II) intermediate after standing overnight at room temperature (see Fig. S24). The formation of [(*t*Bu-terpy)NiPh]I (**S47**) is suggested to happen by the oxidation (single electron transfer to N_2O) of either Ni(I)–I (**13**) or Ni(I)–Ph (**A**) to the corresponding Ni(II), and then Ph or I transmetallation respectively. N_2O has been shown to act as electron receptor in the literature.[76] The HRMS of this sample allowed the confirmation of the presence of (*t*Bu-terpy)$NiPh^+$ and I^-, consistent with the Ni(II) complex **S47** (Fig. S25-26). Interestingly, traces of (*t*Bu-terpy)$NiOPh^+$ were detected, which would be consistent with the intermediacy of the (*t*Bu-terpy)Ni(I)–OPh after O-insertion.

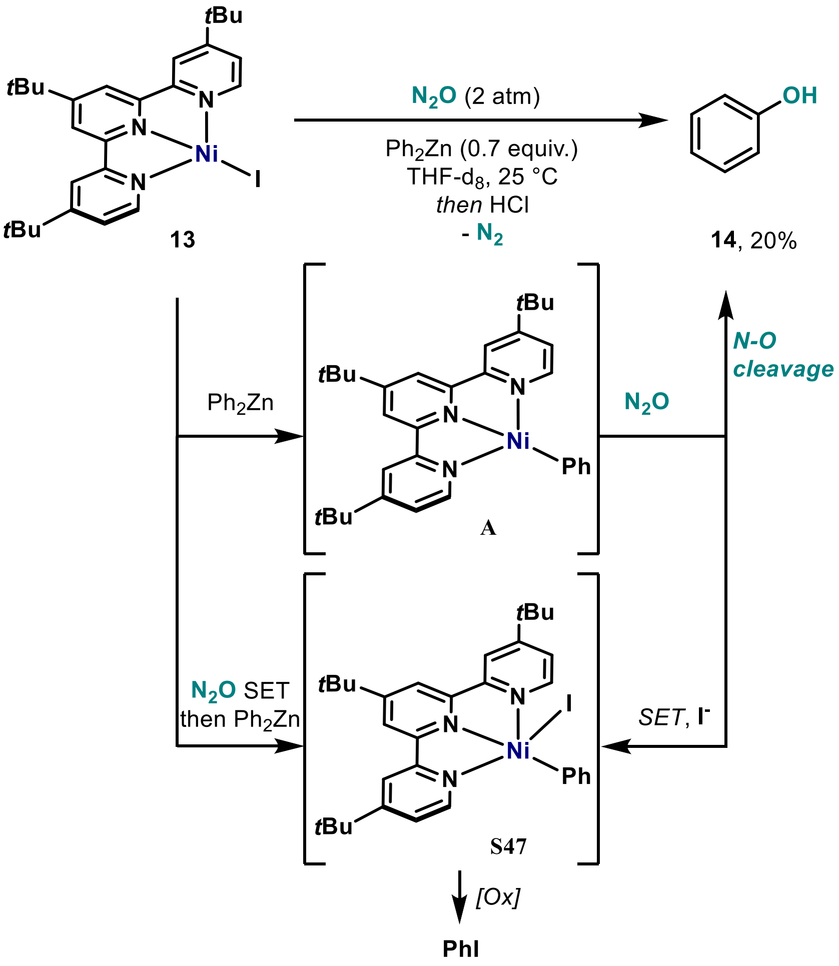

Fig. S23. Potential mechanism for the formation of PhOH and PhI.

NMR data supports the following structure:

User Report
LEF-LA-737-03

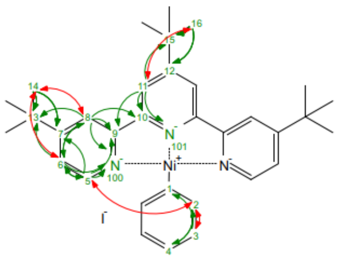

NOESY/REOSY
HMBC

Remarks:
- Sample contains approx 1.1 eq unbound COD
- The sample is diamagnetic as no paramagnetic signals are observed, therefore the Ni(II) species was proposed. The nature of the anion was only proposed based on the SM and with the result from ESI-MS.
- The signal of C1 is not visible in the 1D 13C and was extracted from the 2D-HMBC (cross peak H3-C3). The signal might be broad in the 1D spectrum due to the direct connections of the C-Ni-bond. A folding of the signal can be excluded as a different HMBC with different SW gave the crosspeak at the same position. The shift (160.3 ppm) is in a range where other Aryl-Nickel complexes have been reported (e.g. *Organometallics* 1994, 13, 2, 468–477)

P-ID: ML00xxx
Measured on: 11/05/2021
CHIFFRE: LEF-LA-737-03
ELNA #: 6010
Client: Franck Le Vaillant
Group: Cornella
Spectroscopist: Leutzsch
Analysed on: 12/05/2021
Analysed by: Leutzsch
Amount: 4.0 mg
Solvent: THF
Reference: 1H+13C on solvent,other nuclei w/ xiref
Temperature: 298 K
Spectrometer: av600neo
Probe: cryoBBO
Experiments: 1H-zg30, 13C-zgpg30, [13C, 1H]-hsqcedetgpsisp2.3, [13C, 1H]-hmbcetgpl3nd, [1H, 1H]-cosygpppqf, [1H, 1H]-noesygpph, [1H, 1H]-roesyadjsph, [15N, 1H]-hmbcgpndqf

Atom	δ (ppm)	J	COSY	HSQC	HMBC	NOESY
1 C	160.275				3	
2 C	136.869			2	2, 4	
H	7.790	7.00(3)	3, 4	2	2, 4	3, 5
3 C	127.939			3	3	
H	7.120	7.00(2), 7.30(4)	2, 4	3	1, 3	2
4 C	124.782			4	2	
H	7.008	7.30(3)	2, 3	4	2	
5 C	154.143			5	6	
H	7.440	6.00(6)	6	5	6, 7, 9	2
6 C	124.675			6	5, 8	
H	7.351	1.70(8), 6.00(5)	5, 8	6	5, 8, 13	14
7 C	167.145				5, 14	
8 C	123.266			8	6	
H	9.040	1.70(6)	6	8	6, 9, 10, 13	14
9 C	158.275				5, 8, 11	
10 C	152.739				8, 11	
11 C	122.057			11	11	
H	9.087			11	9, 10, 11, 15	16
12 C	169.277				16	
13 C	36.982				6, 8, 14	
14 C	30.653			14	14	
H3	1.451			14	7, 13, 14	6, 8
15 C	38.431				11, 16	
16 C	31.475			16	16	
H3	1.641			16	12, 15, 16	11
100 N	-164.316					
101 N	-139.848					

Fig. S24. NMR data summary of [(*t*Bu-terpy)NiPh]I (**S47**).

The different spectra are detailled in the NMR spectra section.

*Note: No NMR yield was recorded for this compound. Some remaining COD is found, and originates from the synthesis of (tBu-terpy)Ni–I (**13**).*

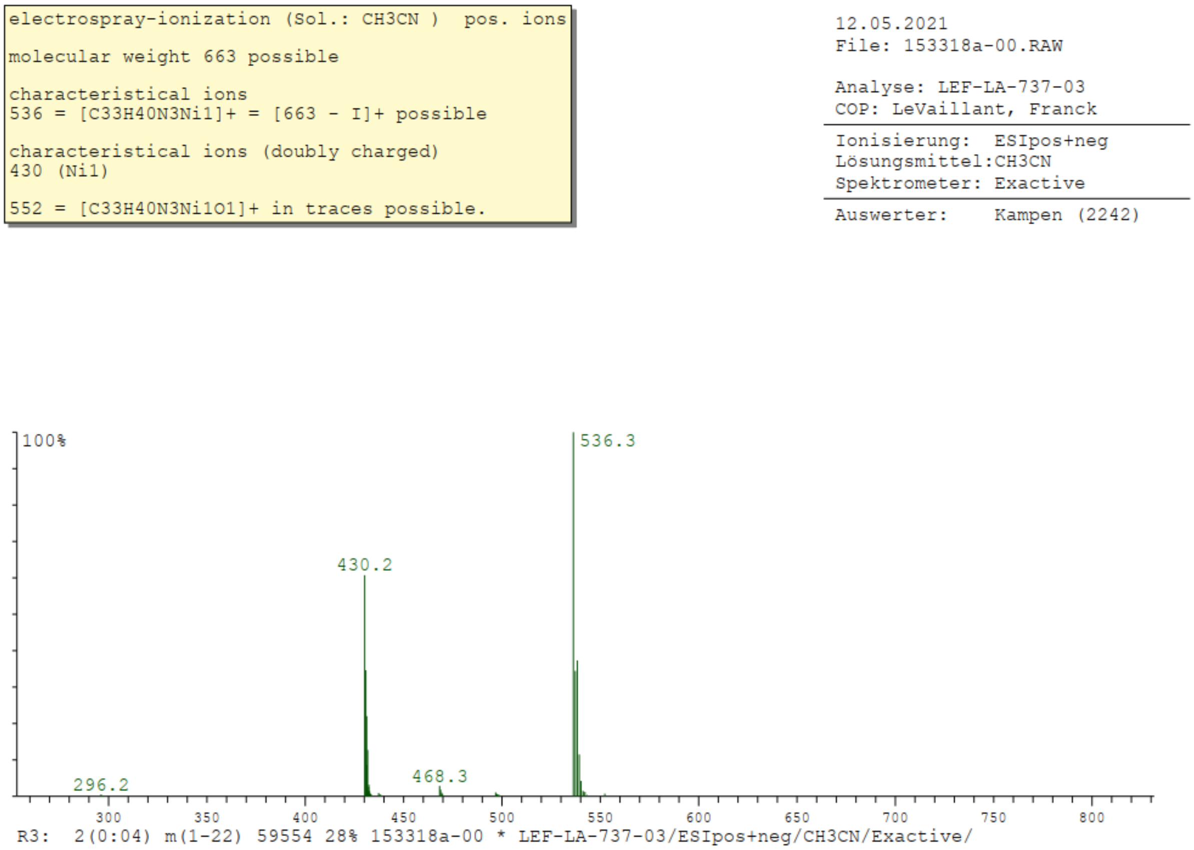

Fig. S25. MS of crude reaction mixture, when exposed to N_2O at –78 °C, using ESI^+.

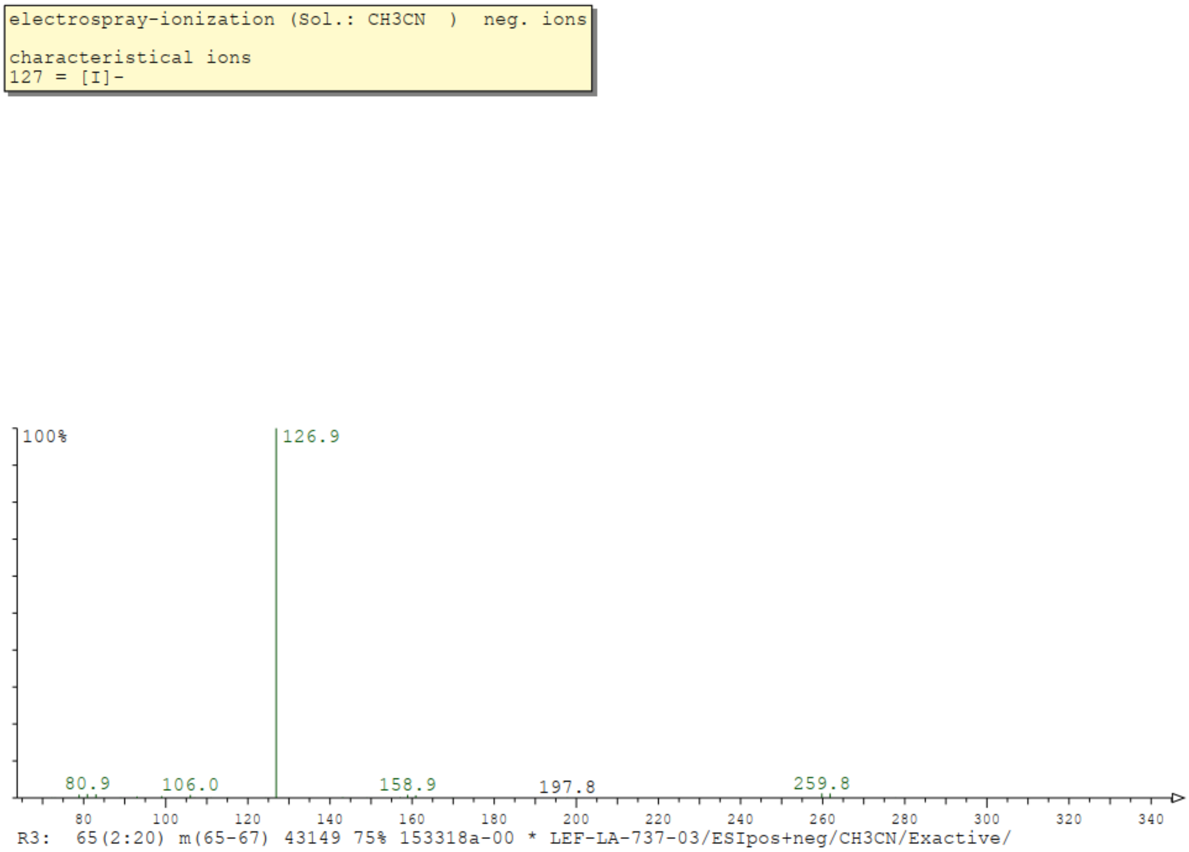

Fig. S26. MS of crude reaction mixture, when exposed to N_2O at –78 °C, using ESI^-.

4.4.4. Control experiments

The same reaction was repeated in absence of Ni complex (only Ph_2Zn in THF-d_8), and no phenol was detected, neither by NMR nor by GC-MS. Only benzene is formed upon quenching the solution. These results rule out a direct O insertion from N_2O to Zn–C bond, and are consistent with a transmetallation step to a Ni-center to explain the observed reactivity described in this section.

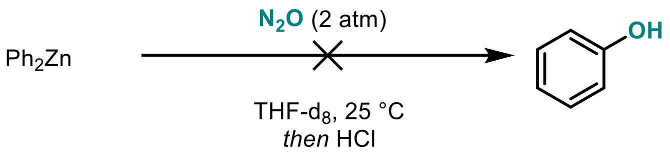

14

5. Optimization of catalytic oxidation of aryl iodides using N_2O

*Note: Side product **S41** (or corresponding ArNMe$_2$) was not found in any of the optimization reactions.*

Note 2: For all catalytic reactions, "finger pressure Schlenks" of 12 mL capacity were used unless otherwise noted. Cleaning of the Schlenks and stirring bars with mixture of sulfuric acid and hydrogen peroxide was undertaken after each reaction to avoid any metal traces.

5.1 Optimization of the reaction

5.1.1. Initial screening

As the stoichiometric experiments were successful with oxidative complex **4** derived from 4-CF_3-PhBr (**S48**), we started our investigations with this starting material (Table S4). Our goal being to use the lowest pressure of N_2O possible, we started the screening with 1 atm of N_2O only. Interestingly, phenol formation (**7**, 10%) was observed using 10 mol% of $NiBr_2$·DME and 11 mol% of terpy **L18**, in presence of 5 equivalents of Zn under N_2O atmosphere. Dimer **5** was also produced in 30% yield. The reaction was uncompleted with about 30% remaining starting material. Among the other ligands screened at this stage, bipy derivatives gave < 5% of phenol, while the phenantroline scaffold did not provide any phenol. Interestingly, increasing terpy loading (22 mol%) completely suppressed the reactivity, thus indicating that ligand saturation is detrimental and Ni(terpy)$_2$ is not an active species.

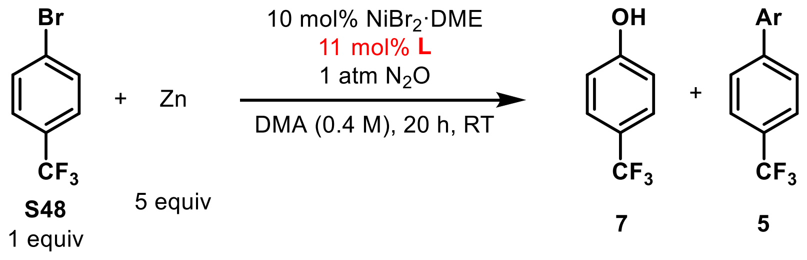

Entry	Ligand	Phenol 7 (%)	Dimer 5 (%)	Conversion S48 (%)
1	bipy **L1**	< 5	20	75
2	dtbbipy **L2**	< 5	25	90
3	phen **L6**	-	50	>95
**4**	**terpy L18**	**10**	**30**	**70**
5	bathocuproine **L10**	-	60	>95
6	2,9-(Bu)$_2$-phen **L12**	-	40	>95

Table S4. Preliminary screening of ligands in the catalytic reactions with 4-CF_3-PhBr. ^{1}H NMR yields are given.

Changing the Ni(II) source revealed that $NiCl_2$ and $Ni(acac)_2$ are not suitable, while $NiCl_2(PPh_3)_2$ gave also 10% yield (Table S5). Increasing the reaction temperature to 60 °C allowed for full conversion, which led to a slight increase of yield (15%).

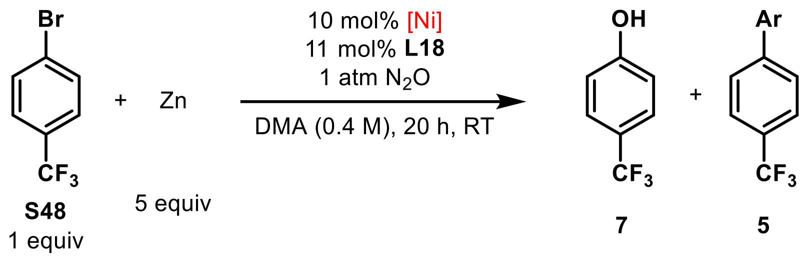

Entry	Ni source	Phenol 7 (%)	Dimer 5 (%)	Conversion S48 (%)
1	$NiBr_2 \cdot DME$	10	30	70
2	$NiBr_2$	10	5	80
3	$NiCl_2 \cdot xH_2O$	traces	N.D	70
4	$NiCl_2(PPh_3)_2$	10	no	70
5	$Ni(acac)_2$	traces	N.D	>95
6	$NiBr_2 \cdot DME$ (at 60 °C)	15	25	>95

Table S5. Screening of Ni sources in the catalytic reactions with 4-CF_3-PhBr. ^{1}H NMR yields are given. N.D.: not determined.

5.1.2. Optimization using aryl iodides

A screening was then performed using aryl iodides as starting materials. We chose electron neutral 4-fluoro-1-iodobenzene (**8**) as starting material, using $NiBr_2 \cdot DME$ as nickel precatalyst ($Ni(COD)_2$ was also tested with similar efficiency), 4 equivalents of zinc powder, in DMA (0.4 M), at room temperature under 1 atmosphere of N_2O (Fig. S27).

General procedure for the optimization of aryl iodides

For the optimization, oven- and heatgun-dried pressure Schlenks with Teflon screw-cap and equipped with a Teflon-coated stir bar were used. These Schlenks were brought into an argon filled glovebox, along with a heatgun-dried vial. Dried DMA was introduced into the vial. nickel precursor, ligand, additives, reducing agent were introduced in the pressure Schlenk. Then, outside the glovebox, using a T-connection, the Schlenk was evacuated and refilled with N_2O. This procedure was repeated three times. Then, under the 1.5 atm of N_2O, the Schlenk was opened and the solvent was added using a syringe *while stirring*. *(Stirring is important to avoid formation of agglomerates.)* As 4-fluoro-phenyl iodide **8** (23 µL, 0.20 mmol, 1.0 equiv) is liquid, it was introduced at this stage using a 25 µl Hamilton syringe. The Schlenk was then

closed, and the pressure of N_2O was adjusted to the expected working pressure (1 – 2 bar). The reaction mixture was kept stirring at 1000 rpm, for 20 h, at the room temperature.
After the reaction, the Schlenk was carefully opened, and the reaction was first diluted using 2 mL of Et_2O. The mixture was quenched first with water (the quenching can be exothermic) followed by HCl (1 M), and extracted 3 times with Et_2O (12 to 15 mL each). The combined organic layers were washed with brine, dried over $MgSO_4$, filtered and concentrated under reduced pressure. The crude was analyzed by ^{1}H and ^{19}F NMR using CH_2Br_2 or 4-fluoro-nitrobenzene as internal standard.

Although suitable in stoichiometric reactivity, and similarly for the arylbromides, bidentate N,N-ligands (**L1-17**) furnished generally homocoupling and protodehalogentation products in high yields, with phenol formation being a minor pathway (< 5%). The goal was therefore to diminish these competitive side reactions to drive the reactivity towards N_2O activation.

Screening tridentate ligands revealed that terpy **L18** (30-35%) and pyridine-bispyrazolyl **L19** (5-20%) allowed catalytic turnover with promising conversion to the phenol product. On the other hand, tetradentate ligands such as quaterpyridine **L20** or **L21** completely inhibited the reactivity. Other tridentate ligand scaffolds (pyridine bisoxazoline **L22-24**, pyridine bis-imine **L25**, recent scaffolds **L26-28** used by Weix and coworkers for reductive couplings, pyridine bis-benzimidazole **L29-30** and pyridine bisquinolines **L31-32**) resulted in either full conversion to Ar-Ar & Ar-H, or almost no reactivity. In reactions using **L20-L32**, only traces of phenol can be sometimes detected.

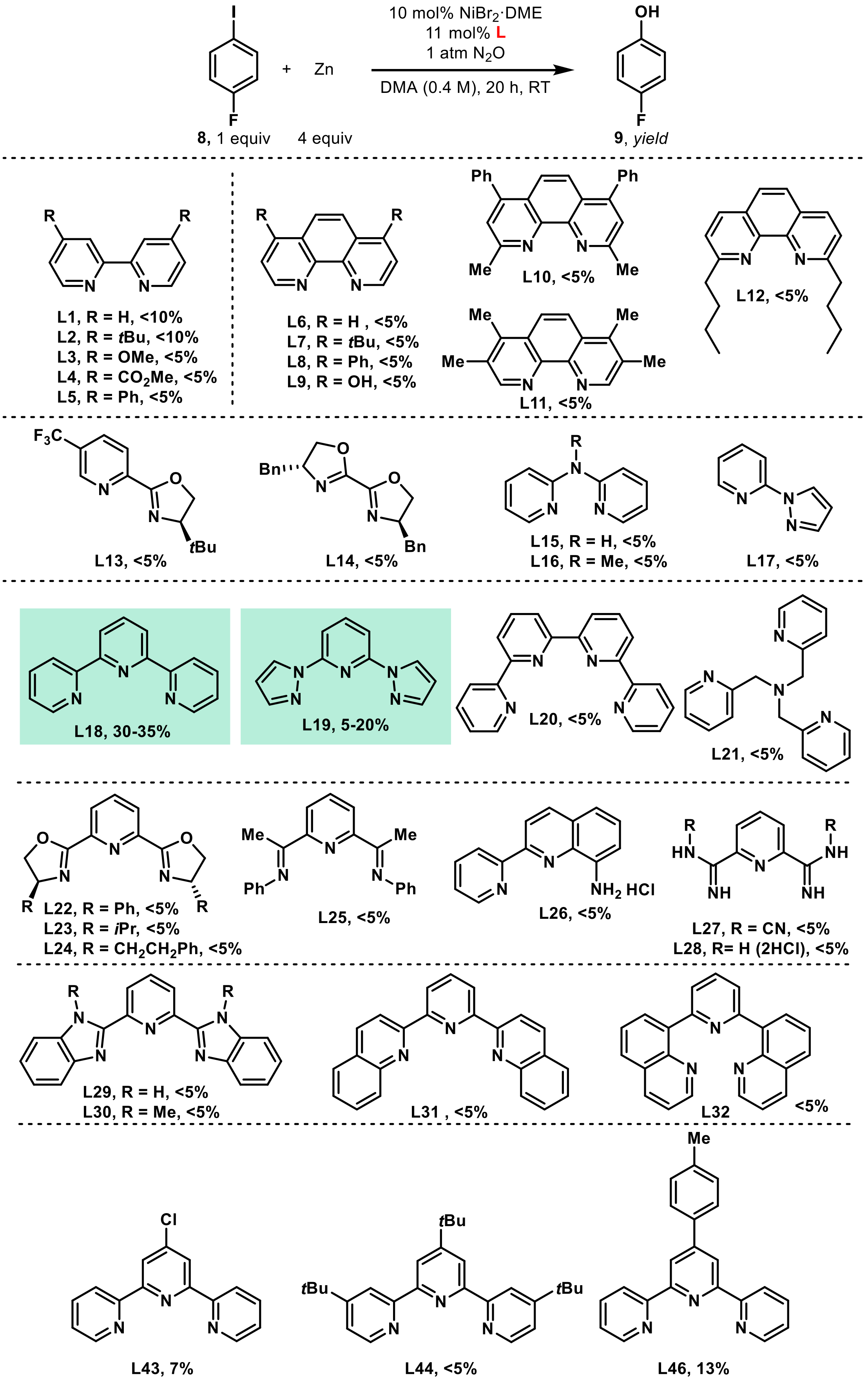

Fig. S27. Screening of ligands in the catalytic reactions with 4-F-PhI. ^{1}H NMR yields are given.

From this first round of ligand screening, we identified terpy as a good candidate for intermediate reactions screening. Commercially available terpy derivatives **L43, L44, L46** were already tested at this stage, but failed to provide phenols catalytically. The results were found to be not very reproducible, and vary between 25-35% when repeating the best results obtained so far with terpy **L18** with 4 equivalents of Zn. Reducing the amount of Zn powder to 2.5 equivalents gave similar yield (25-35%) but did not enhance the reproducibility. We thought that additives could help for achieving better reproducibility.

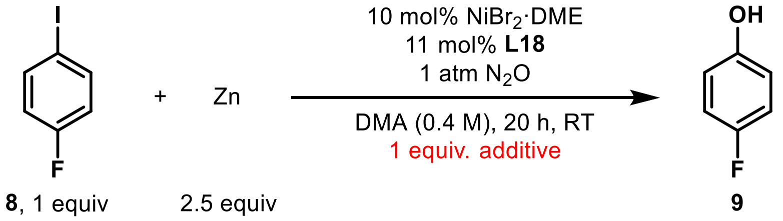

At this stage, extensive screening of classical additives ($ZnCl_2$, $ZnBr_2$, ZnI_2, $Zn(OTf)_2$, $MgCl_2$, $MgBr_2$, LiCl, LiBr, LiI, NaI, NaCl, KI, KF, $TiCp_2Cl_2$, $TiCl_4$, NEt_3.HCl, TMSCl) and some bases (pyridine, NEt_3, K_3PO_4, K_2HPO_4, Na_2CO_3, K_2CO_3) was performed using 2.5 equiv of Zn. No clear trend was found, and repeatedly the reaction conditions were still found not reproducible. Therefore, a quick screening of metal to ligand ratio was performed.

Ligand to metal ratio

The ligand to metal ratio is usually a key parameter in transition metal catalyzed couplings. In our transformation, it also appears to be the case (Table S6). With only 8% of ligand for 10% of metal center, very low amount of phenol is formed, and dimer is the major product (Entry 1). With 11 mol% of terpy, the system affords reasonable amounts of phenol (along with dimer and protodemetallation) (entry 2); however, increasing to 15 mol% of terpy allowed more reproducibility and slightly higher yield (32-37%, >10 experiments, entry 3)). Even higher ligand loading (17.5 mol%) is detrimental, consistent to our observation for aryl bromides, probably due to the formation of inactive $Ni(terpy)_2$. *Considering that terpy may act as a tridentate ligand or bidentate ligand depending of the catalytic step*, we thought that maybe a mixture or terpy and bipy would enhance this interconversion of coordination. Unfortunately, both attempts using 7.5%/7.5% or 10%/5% of terpy/bipy led to low formation of phenol (8%) (Entries 5-6).

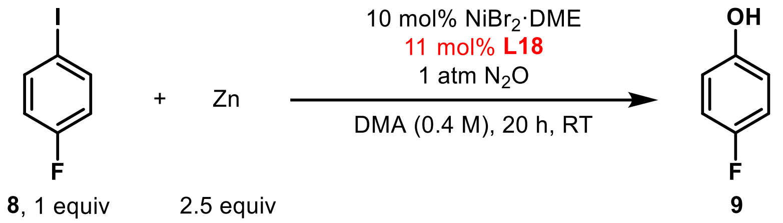

Entry	%terpy L18	Phenol (%)	Conversion	Remarks
1	8	<5	>95%	Mainly dimer
2	11	25-35	>95%	Less robust
**3**	**15**	**32-37**	**>95%**	**Reproducible**
4	17.5	10	75%	Unreactive NiL_n
5	7.5 + 7.5 bipy	8	>95%	50% dimer
6	10 + 5 bipy	8	>95%	30% dimer

Table S6. Screening of Ni:L ratio in the catalytic reactions with 4-F-PhI. ^{1}H NMR yields are given.

With more robust conditions in hand, solvent screening was performed (Table S7). Not surprisingly, highly polar amide solvents were the only suitable solvents. Concentration was also tested with very similar results for 0.2 to 0.5 M, and diminished yield for 0.1 M. Therefore 0.4 M was kept as standard. Bulky amide solvents such as **S49**, **S50**, and **S51** resulted in much lower conversion which is ascribed to a lower polarity, hence difficulties to undergo efficient reduction with heterogeneous zinc particles.

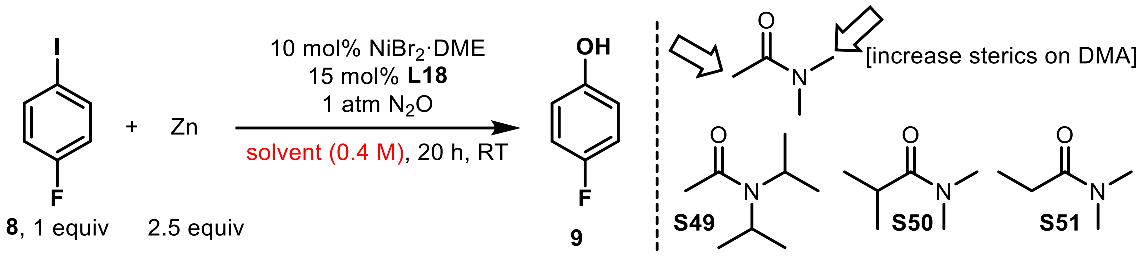

Entry	Solvent	Phenol (%)	Conversion
**1**	**DMA (0.2 – 0.5 M)**	**25-37**	**>95%**
2	DMF	19	>95%
3	NMP	6	>95%
4	DMPU	5	>95%
5	DMSO	7	>95%
6	THF	-	>95%
7	THF/DMA (1:1)	-	>95%
8	MeCN	-	low
9	**S49** (RT)	-	<5%
10	**S49** (90 °C)	-	20%
11	**S50**	2	Low
12	**S51**	5	57

Table S7. Screening of solvents in the catalytic reactions with 4-F-PhI. ^{1}H NMR yields are given.

With the more reproducible reaction conditions in hand (L:Ni ratio), we tested again the screening of additives, with a specific focus on halide sources (Table S8). NaI was found to be a promising additive for our study, with 52% NMR yield.

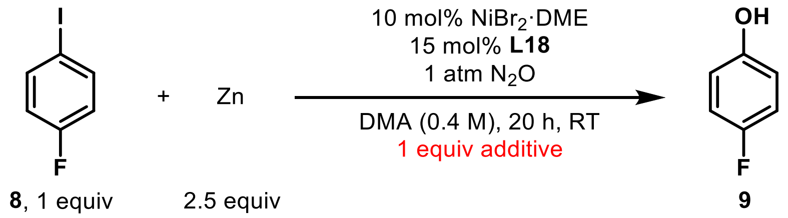

Entry	Additive	Phenol (%)	Dimer (%)	Conv. (%)
1	None	25-37	30-40	> 95
2	CsF	48	31	> 95
3	CsCl	43	31	> 95
4	CsBr	45	39	> 95
5	CsI	45	52	> 95
6	KF	19	80	> 95
7	KCl	35	30	> 95
8	NaCl	12	20	> 95
**9**	**NaI**	**52**	**28**	**> 95**

Table S8. Screening of additives in the catalytic reactions with 4-F-PhI. ^{1}H NMR yields are given.

The use of NaI in at least stoichiometric amount was found to be crucial for enhancing both the yield and reproducibility of the results (Table S9). For the next experiments, 2.0 equivalents of NaI was used as standard. Other iodide sources (NR_4I, R=Me, Et, Pr, Bu, Hex) were tested but found to be inferior to NaI.

Entry	X equiv NaI	Phenol (%)	Dimer (%)
1	0.25	low	n.d.
2	0.50	47-50	20-21%
3	1.0	50-52	28%
4	1.5	50-55	28%
**5**	**2.0**	**52-56**	**26-30%**
6	4.0	42-44	40%

Table S9. Tuning the stoichiometry of NaI in the catalytic reactions with 4-F-PhI. ^{1}H NMR yields are given.

The use of relatively big size particles of Zn (40 – 100 µm) was also very important to get high efficiency in the reaction (Table S10, entries 1-2). Indeed, using smaller particles sizes significantly decreased the formation of phenol due to faster side reactions pathways (especially homocoupling) (Entries 3-4). Interestingly, the reaction tolerates well the presence of 10 mol% of TFA as an additive to activate in situ the Zn powder (Entry 5).

Entry	Zn (µm)	Phenol (%)	Dimer (%)
**1**	**40 - 100**	**52-56**	**26 - 30**
2	40 - 60	55	28
3	< 10	44	49
4	Activated Zn*	< 20	n.d.
5	40 – 100 with 10 mol% TFA	55	23%

Table S10. Checking the effect of Zn particles size in the catalytic reactions with 4-F-PhI. ^{1}H NMR yields are given.

Different pressures of N_2O were also tested and a trend was observed: higher pressure of N_2O was correlated to higher amount of phenol (5-10% increase for 2 bars) (Table S11). Interestingly, repeating entry 1 at 35 °C gave a lower yield of phenol (45%) and greater homocoupling (40%).

Entry	Pressure N_2O	Phenol (%)
1	1 atm	52-56
2	1.5 atm	58
3	1.8 atm	58-60
**4**	**2.0 atm**	**60-62**

Table S11. Tuning the pressure of N_2O in the catalytic reactions with 4-F-PhI. ^{1}H NMR yields are given.

Finally, with this promising set of conditions in hands (60% with terpy **L18**), we decided to reevaluate the tuning of terpy and pyridine-bispyrazolyl scaffolds (Fig. S28). The reevaluation of **L19** afforded an interesting 48% yield. A common feature on ligands to address the competitive homocoupling process is to increase steric bulk around the Ni-center. However, no phenol formation was observed as soon as one or two substituents are added at the *ortho* position of the nitrogen atom on the side heterocycles (**L33-38**). Interestingly, substitution at the 5- and 5’’- positions of the terpy scaffold is tolerated (**L40-42**), with an ethyl substituent giving similar yield as the naked terpy. Again, tuning the electronic on the terpy using

commercially available terpy derivatives mainly resulted in low (**L45** and **L47**) to medium yields (**L44** and **L46**).

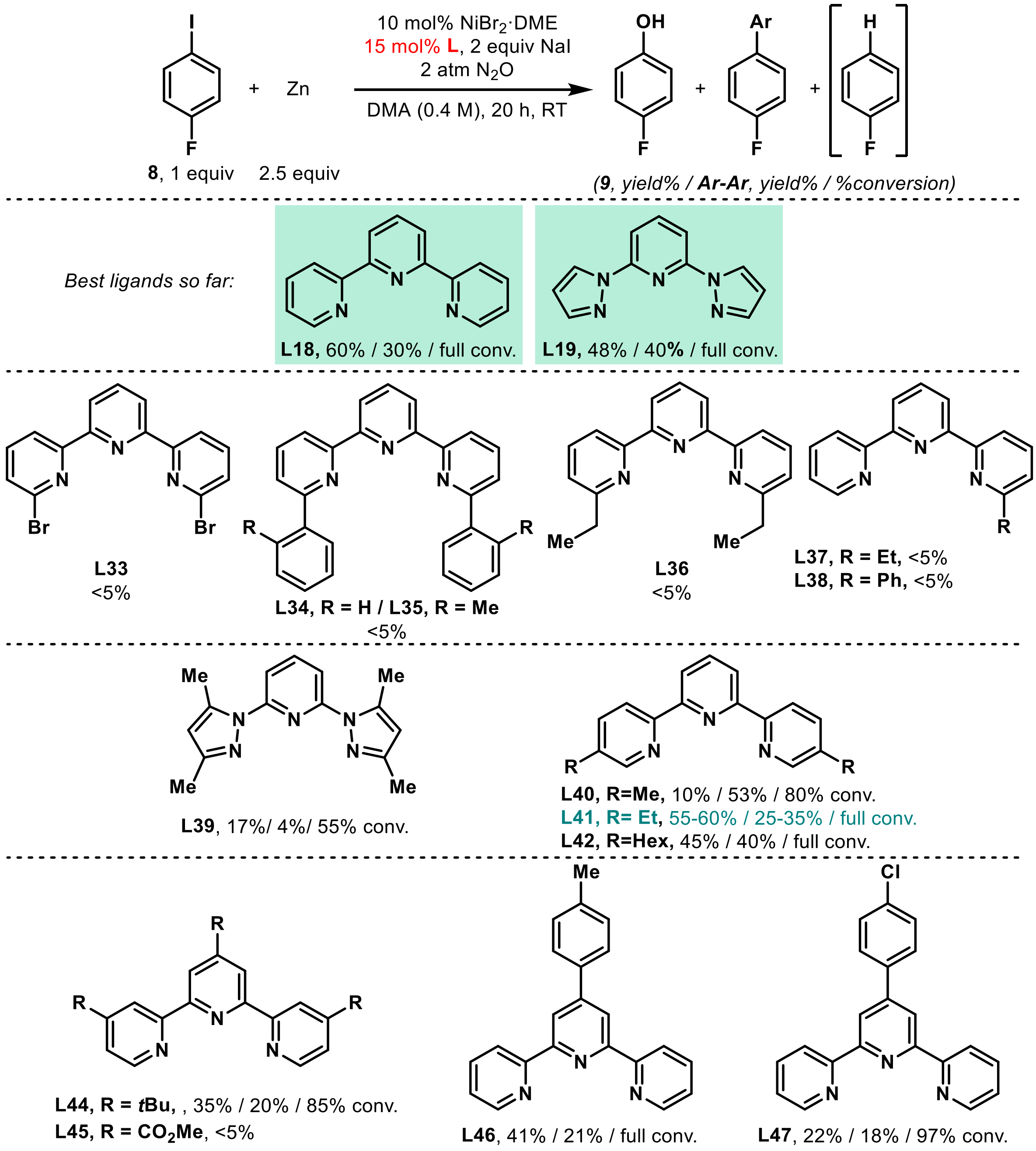

Fig. S28. Screening of tridentate ligands in the catalytic reactions with 4-F-PhI. ^{1}H NMR yields are given.

Finally, we turned our attention to the synthesis of electronically asymmetric tridentate ligands (Fig. S29). First, bidentate **L48** confirmed that a tridentate ligand is required. The introduction of oxazoline instead of one pyridine ring decreased the yield of phenol to 10% (**L49**). Using only one pyrazolyl allowed the identification of bipyridine-pyrazole (**L50**) as an excellent

candidate for the catalytic synthesis of phenols using N_2O and aryl halides. Surprisingly, the homocoupling is dramatically decreased. Other scaffolds, involving protic side arm-, nitrile containing, and more flexible or more rigid ligands resulted in diminished performance (**L51-54**). Fine-tuning the bipyridine-pyrazolyl scaffold did not allow to further improve the yields, but still provided useful information on the important parameters to consider on the pyrazolyl moiety (**L55-61**): 1) steric hindrance close to the Ni center is detrimental; 2) EWG is detrimental; 3) EDG is tolerated and disubstitution is also tolerated as long as it is not close to the Ni center; 4) the pyrazolyl scaffold is superior to the triazene or the thiophene ones.

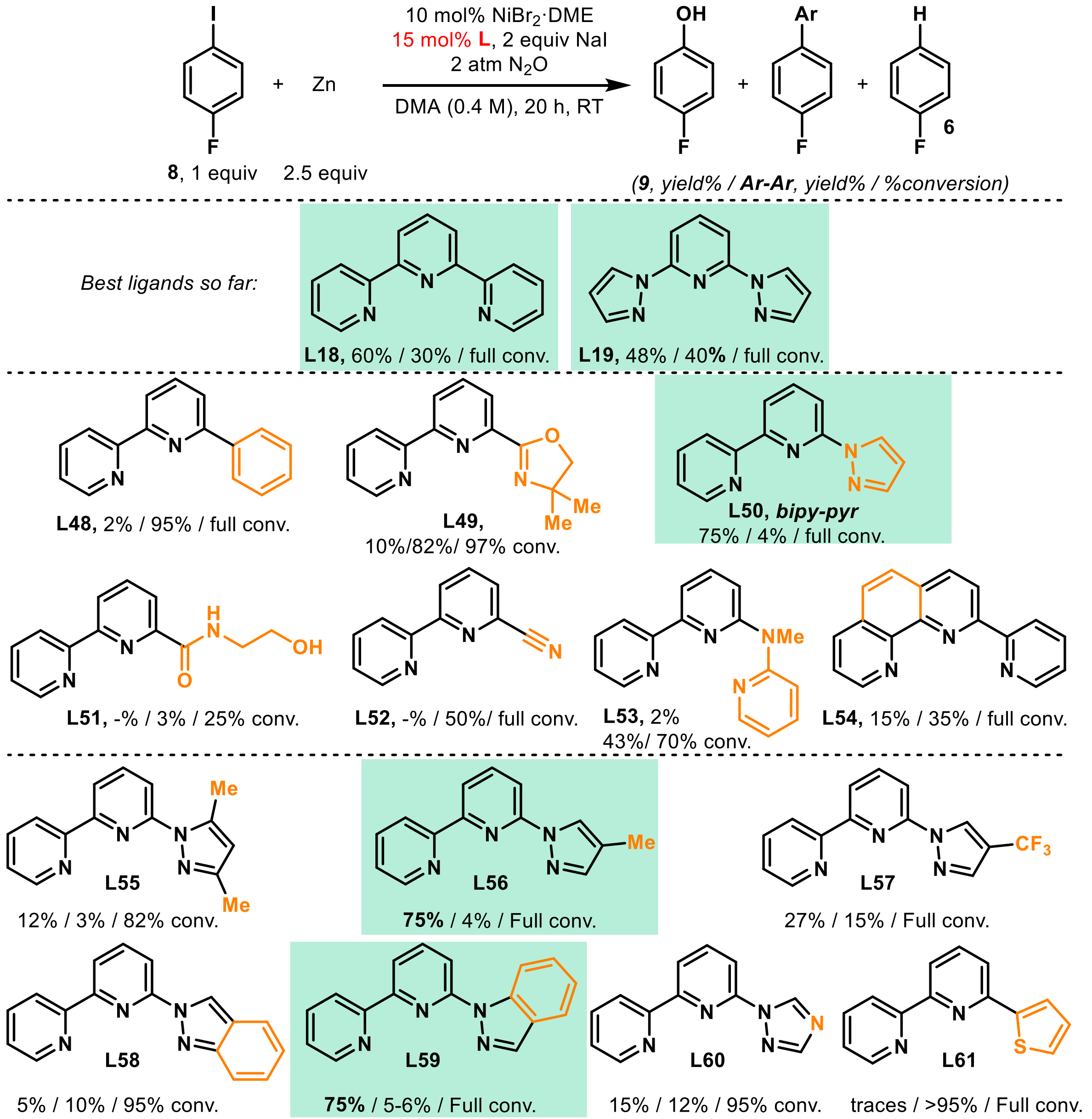

Fig. S29. Screening of electronically asymmetric ligands in the catalytic reactions with 4-F-PhI. ^{1}H NMR yields are given.

It is worth mentioning that no or only traces of phenol formation were observed with any of the following phosphine ligands (monodentate screened with 30 mol%, and bi- and tridentate screened with 15 mol%) (Fig. S30). They have been tested several times at different stages of the optimization, without enabling successful conversion to phenols.

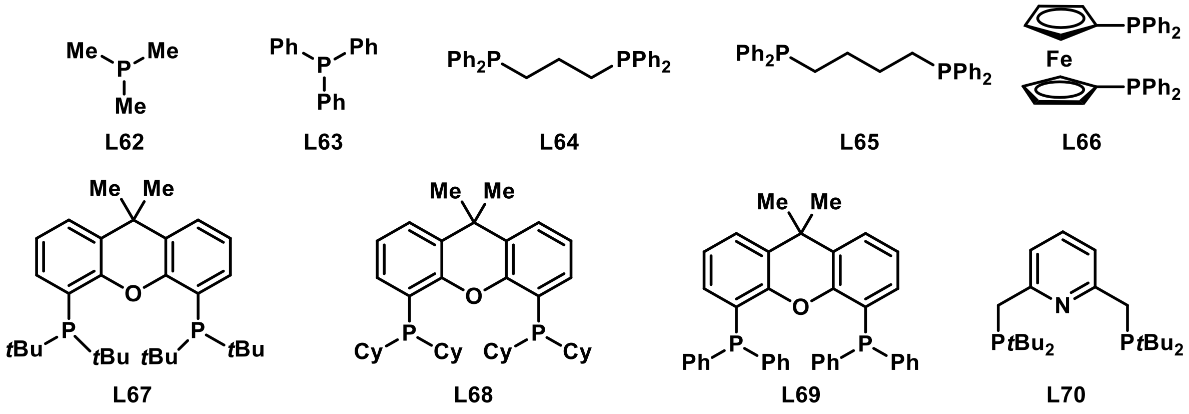

Fig. S30. Set of phosphine ligands tested in the catalytic reactions with 4-F-PhI.

Ligands screening conclusion:

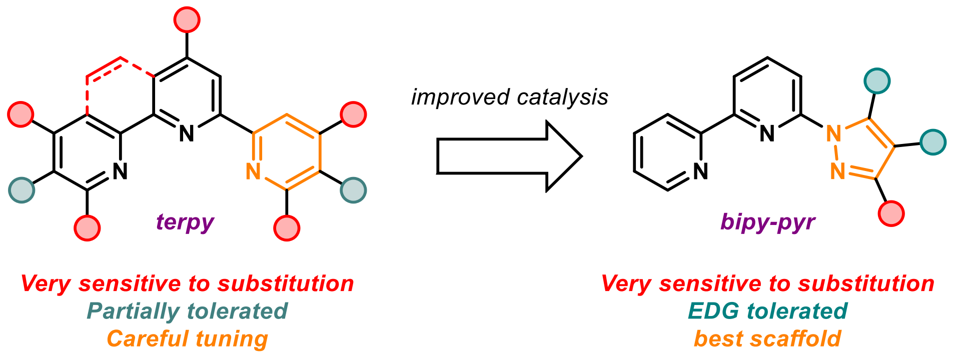

Fig. S31. Overview of key structural parameters of the ligand for efficient catalysis.

The ligand screening revealed that tridendate N,N,N-ligand is of utmost importance to get reactivity. Among them, the terpy-based scaffold is superior to the other tested. However, it is very sensitive to substitution: no substituent should be added at the 6,6´´-positions of the terpy ligand, otherwise a dramatic shut down of the N_2O reactivity is observed. 4,4´,4´´-Positions are also sensitive, both to EWG or EDG substituents. Increasing rigidity by introducing a phenantroline scaffold was also detrimental compared to the more flexible terpy-scaffold, although some reactivity was observed. Finally, 5,5´´-positions can tolerate some alkyl chains, but methyl groups have a very negative impact on the reactivity. Changing one or two pyridine ring(s) to another group also resulted in an on/off reactivity. Interestingly, pyridine bis-

pyrazolyl gave some reactivity, albeit lower than terpy; while bipy-pyrazolyl provided an enhanced yield of the phenol product. Tuning the pyrazolyl moiety did not further improve the yield, but allows a better understanding of the ligand. Therefore, steric hindrance close to the Ni-center and EWG should be avoided.

5.2. Comparison of product distribution with several ligands

The following comparison revealed the dramatic ligand effect on product distribution from bipy scaffold to terpy to bipy-pyr.

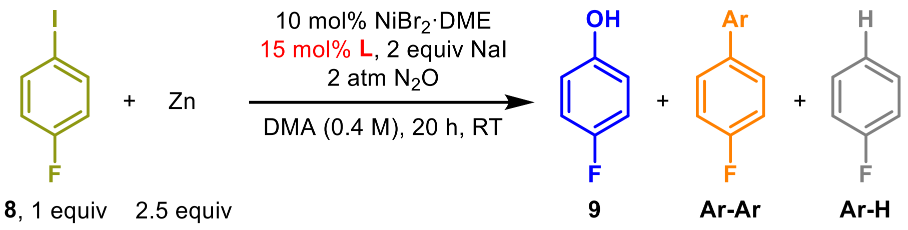

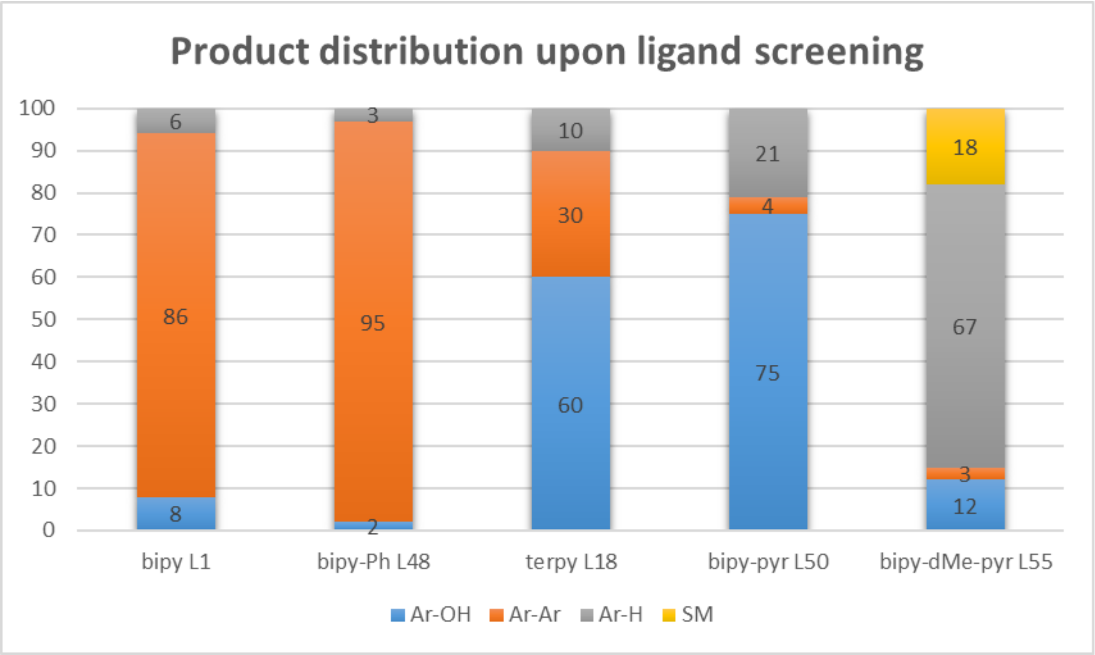

Fig. S32. Comparison of product distribution obtained with 5 ligands, highlighting the importance of specific structural parameters.

5.3. Fine-tuning the conditions

Under these conditions the reevaluation of Ni sources allowed the improvement of the phenol yield when using $NiBr_2$·diglyme (Table S12). Decreasing the catalyst loading to 5 mol% gave slightly diminished yields (Entry 3); while 2.5 mol% of catalyst resulted in incomplete conversion and low phenol formation (Entry 4).

The Zn amount could not be decreased when using terpy (**L18**) as ligand (Entry 6). On the other hand, gratifyingly, only 1.5 equivalents of Zn and NaI are suitable for good and reproducible reactivity using bipy-pyrz (**L50**) (Entry 10). Substoichiometric amounts of Zn results in limited conversions, hence showing the requirement of Zn for turnovers.

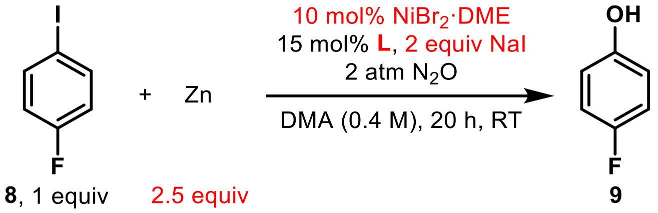

Entry	Change	With L18, yield Phenol (%)	With L50, yield Phenol (%)
1	None	60-62	75-80
2	Using 10 mol% $NiBr_2$·diglyme	70-75	80
3	Using 5 mol% $NiBr_2$·diglyme	65	70-75
4	Using 2.5 mol% $NiBr_2$·diglyme	20%	*Not tested*
5	10 mol% $NiBr_2$·diglyme· with 2.0 equiv Zn	*Not tested*	80-84
6	10 mol% $NiBr_2$·diglyme with 1.5 equiv Zn	59	80-85
7	10 mol% $NiBr_2$·diglyme with 1.2 equiv Zn	*Not tested*	78
8	10 mol% $NiBr_2$·diglyme with 1.0 equiv Zn	*Not tested*	34
9	10 mol% $NiBr_2$·diglyme, **no Zn**	0	0
10	10 mol% $NiBr_2$·diglyme 1.5 equiv NaI, 1.5 equiv Zn	*Not tested*	80-85

Table S12. Tuning the final conditions for the catalytic reactions with 4-F-PhI. ^{1}H NMR yields are given.

Interestingly, round-bottom septum-screw cap vials (Fig. S33) could be used for up to 1.75 bar, using needle connection and safety shield for filling with N_2O, and afforded only slightly

diminished yield (loss of about 10% in general). This is convenient if the practioner wishes to follow the reaction, add chemicals, or for headspace analysis. However, for safety reasons, we still recommend using pressure Schlenks.

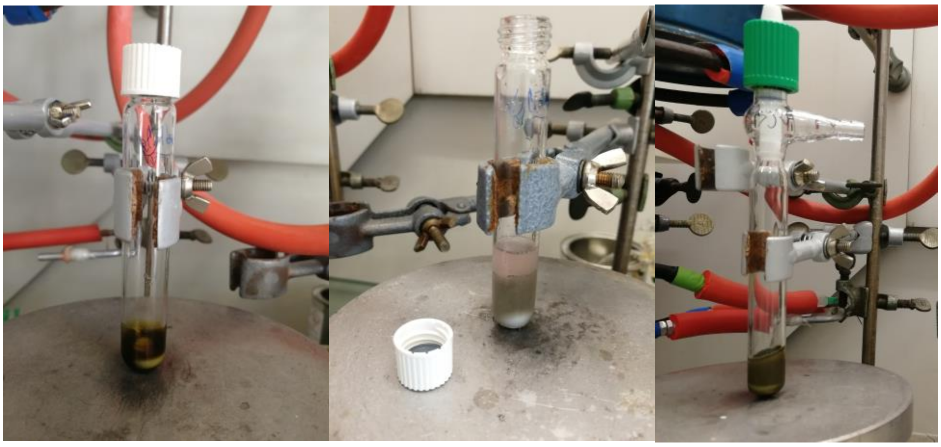

Fig. S33. Use of vial, during the reaction (*left*), after quenching (*middle*). Typical pressure Schlenk used for the catalytic synthesis of phenol using N_2O (*right*)

Optimized conditions

The following optimized reaction conditions will be used for exploring the scope of the catalytic synthesis of phenols using nitrous oxide and aryl halides (Fig. S34).

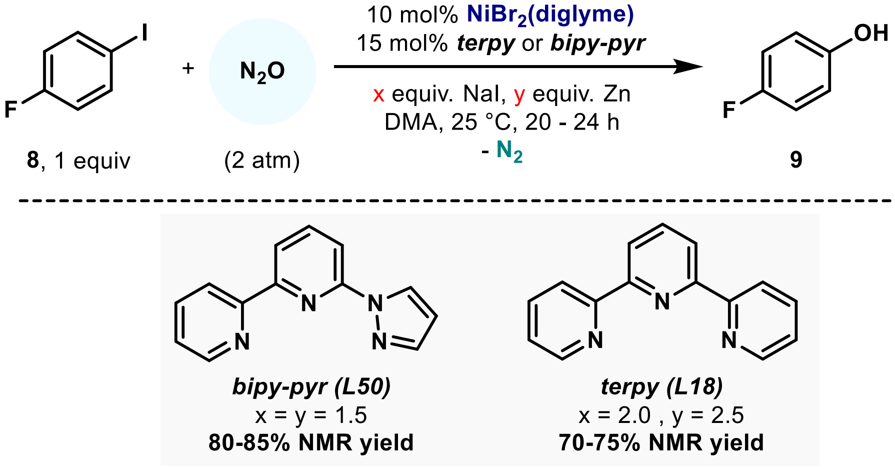

Fig. S34. Optimized conditions for the catalytic synthesis of phenols using N_2O and aryl halides.

In case a high decrease of reactivity is observed, it is important to reconsider:

- That the pressure Schlenk was well-sealed. (Overpressure when opening after the reaction).
- The quality / pressure available in the bottle of N_2O.
- Assessing that good vacuum/ N_2O cycles were performed.
- The purity of the ligand.
- The quality of the DMA (dry and air-free).
- The quality of the starting aryl halides.
- Starting the stirring several seconds before adding the solvent.
- All solids (apart from Zn) dissolved and vigorous stirring.

5.4. Comparison of terpy and bipy-pyr ligands

One clear difference between terpy and bipy-pyr is that the product distribution changed dramatically: homocoupling is almost completely inhibited when using bipy-pyr, resulting in a greater phenol product yield and a reduced arene side product yield. One experimental observation is that the reaction mixture is generally more homogeneous with bipy-pyr compared to when terpy is employed (Fig. S35). It may have an impact of the amount of N_2O in the reaction mixture.

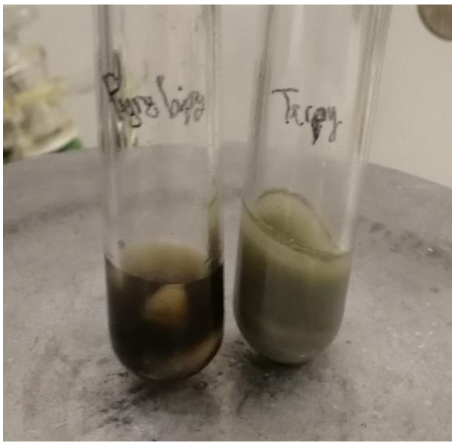

Fig. S35. Higher solubility using bipy-pyr vs terpy.

It is worth mentioning that terpy (**L18**) from various commercial sources were tested and gave slightly diminished yields compared to the initial batch (Sigma) used for the optimization and scope.

Using bipy-pyr (**L50**), the NMR yield during the optimization was found to be reproducible with a 5% margin error with at least 3 different batches. A 2nd column or recrystallization can help securing high purity for catalysis.

5.5. Synthesis, characterization and reactivity of (bipy-pyr)$NiBr_2$ (10)

To investigate how the bipy-pyr ligand coordinates to the nickel center, complexation experiments were conducted.

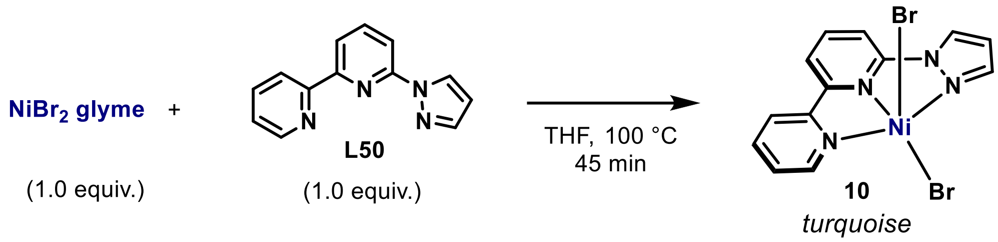

L50 (bipy-pyr, 94 mg, 0.42 mmol, 1 equiv.) and $NiBr_2$·DME (131 mg, 0.420 mmol, 1 equiv.) were introduced into a heatgun-dried Schlenk flask. Tetrahydrofurane (THF, 6 mL) was added and the mixture stirred at 100 °C for 45 min. After letting it cool to room temperature, it was filtrated under argon and washed with THF, diethyl ether, and pentane. A turquoise solid was obtained in 67 % yield (125 mg).

The Ni(II) sample, analyzed *via* ^{1}H-NMR in DMSO-*d*$_6$, appeared to be paramagnetic presenting signals ranging from –2 ppm to 75 ppm (Fig.). This can be attributed to the geometry of the complex, rendering it high-spin, hence paramagnetic with two unpaired electrons. Judging from the number of signals in the paramagnetic region, the sample might contain more than one species in solution, appearing probably due to a dynamic behavior in solution. Multiple species with different NMR activities in equilibrium with each other could be formed, such as monomeric, dimeric, and polymeric species, or the different two species formed via a 1,4-metallotrophic shift in case of a bidentate coordination.[77-78]

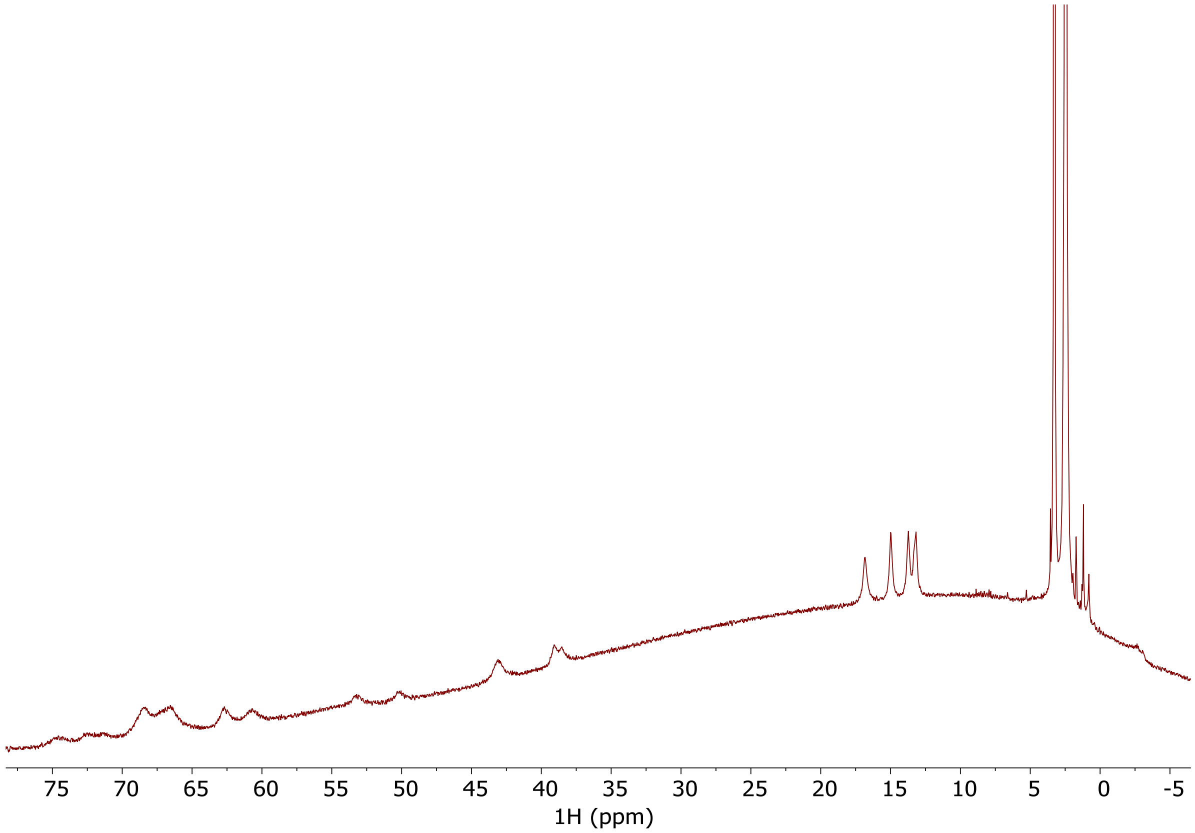

Fig. S36. ^{1}H-NMR spectrum after complexation of **L50** (bipy-pyr) to nickel(II) in DMSO-d_6.

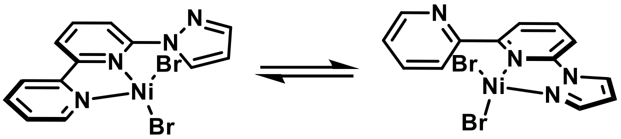

Eq. 1. Possible 1,4-metallotrophic shift in solution

Recrystallization from ethanol afforded yellow crystals which were analyzed *via* X-ray spectroscopy (Fig. S37). Ni(bipy-pyr)Br_2 monomers were revealed in which bipy-pyr (**L50**) coordinates as a tridentate ligand appearing inverted in 20% of the structure (*N.B. The full data is available in section 10*).

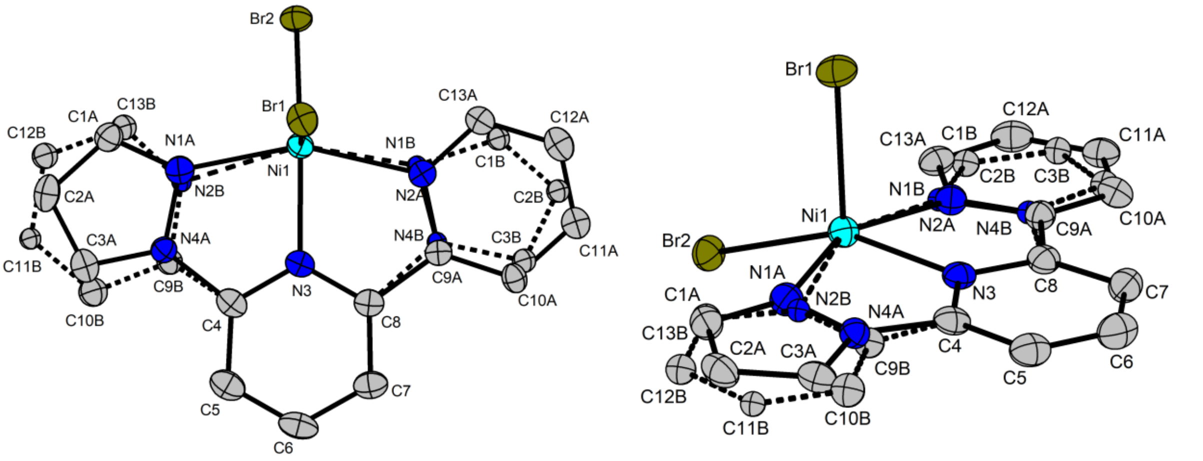

Fig. S37. Molecular structure of **(10)** showing the partial disorder of the *N*,*N*,*N*-(6-pyrazolyl-2,2'-bipyridine) ligand [refined occupancy of the major component 0.763(9)] and the asymmetric binding of the two bromide groups. Left: top view, right: side view. H atoms have been omitted for clarity.

The catalytic activity of the prepared precomplexated Ni(bipy-pyr)Br_2 **(10)** was investigated. These experiments were conducted following general procedure **GP1** employing Ni(bipy-pyr)Br_2 **(10)** (9 mg, 0.02 mmol, 10 mol%) instead of $NiBr_2$·diglyme and bipy-pyr.

The Ni(bipy-pyr)Br_2-catalyzed OAT produced phenol in 68% NMR yield (Table S13, entry 1), thus proving its intrinsic catalytic activity. The lower yield in comparison to the use of nickel(II) precursor $NiBr_2$·diglyme (Table S1, entry 3) might be caused by the lower initial solubility of **(10)**. Moreover, by adopting the optimal ligand-to-metal ratio of 1.5 : 1 by adding 5 mol% of bipy-pyr (2 mg, 0.01 mmol, Table S13, entry 2), the yield of phenol could be improved to 75%.

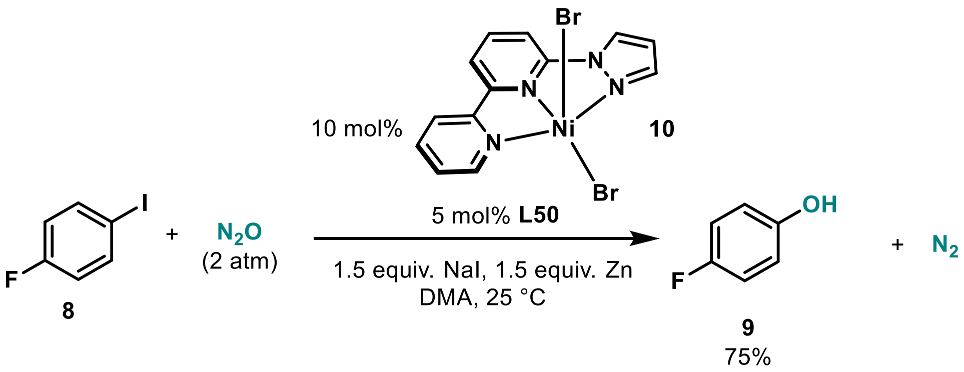

Entry	Ni-source	bipy-pyr	Yield
1	Precomplexated Ni(bipy-pyr)Br_2	0 mol%	68%
2	Precomplexated Ni(bipy-pyr)Br_2	5 mol%	75%
3	$NiBr_2$·diglyme	15 mol%	80%

Table S13. Catalytic activity of Ni(bipy-pyr)Br_2 (**10**). ^{1}H NMR yields are given.

The higher ligand loading might minimize the presence of unligated nickel, which would be prone to decomposition *via* reduction to nickel(0) and aggregation to nickel black.[79]

5.6. Control experiments

- Importantly, the stoichiometric reaction can proceed with other reducing agents than Zn(0) or Mn(0), meaning that the intermediacy of ArM(X) (M=Zn or Mn) is not responsible for the observed reactivity.
- Control experiments consisting of stirring solutions of Ph_2Zn and PhZnBr under N_2O followed by acidic workup did not afford phenol.

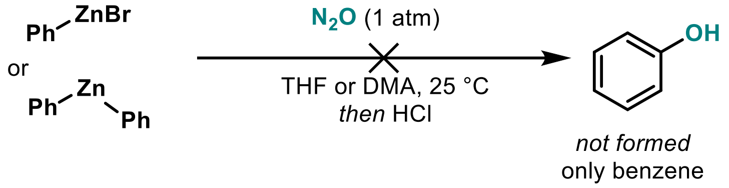

- Also important from the stoichiometric reaction, is that the reaction can proceed in THF. This result rules out the hypothesis of DMA being an O-source.
- Removing the reducing agent from the optimized conditions resulted in low conversion (20%), mainly towards protodemetallation side product. No phenol was observed.
- Removing the nickel catalyst or the ligand from the optimized conditions resulted in no conversion. No phenol was observed. These results point towards a catalytic cycle involving nickel complexes.
- In this regard, the use of other Ni (pre)catalysts of various oxidation states ($NiBr_2$·DME, $NiBr_2$·diglyme, $Ni(COD)_2$, terpyNiBr, terpy$NiBr_2$, terpyNiI_2) was shown to give also good conversions to the phenol product.

5.7. Origin of O-atom

- The analysis of the headspace using GC-TCD was performed for several reactions of the scope (*see section 8*). In all cases, significant amount of N_2 was detected, thus pointing towards the activation of N_2O to give phenol and N_2.
- Repeating the optimized catalytic reaction conditions under argon or air did not provide any phenol. Under argon, full conversion is observed (Ar-Ar and ArH), while under air almost no conversion was detected (*compatibility issue of Ni with O_2 for catalysis*).

These results point towards N_2O being an efficient O-atom source with good compatibility with reductive conditions.

- The addition of 3 equivalents of water under N_2O shut down the phenol formation. Only traces of phenol were detected, while almost full conversion to the protodemetallation side product was observed (very low homocoupling).
- The addition of 3 equivalents of water under argon did not afford any phenol. Full conversion of the starting material was observed, with 17% of dimer, the rest being protodemetallation side product. Our system is therefore not based on the system of Nocera and coworkers (Ni(I)/Ni(III) catalysis with water).[32]
- The addition of molecular sieves (powder) to the optimized reaction conditions still allowed good phenol formation (78% yield compared to 85% without MS), thus indicating that water is not the O-source. Lower phenol yields could be explained by a more difficult reduction in the very heterogeneous reaction mixture.
- When the catalytic model reaction was carried out in ^{18}O-DMF (25% enriched in ^{18}O), 62% isolated yield of ^{16}O-product was obtained and no incorporation of ^{18}O was observed. This experiment rules out a potential activation of the solvent (DMF/DMA) by low valent Ni species, and therefore confirming that N_2O is the O-source in our system (*see section 8 for details*).
- When the catalytic model reaction was carried out using in situ generated ^{18}O-N_2O (23% enriched in ^{18}O), 34% isolated yield of phenolic product was obtained, and 22% of incorporation of ^{18}O was observed. This experiment confirms that N_2O is the O-source in our system (*see section 8 for details*).

6. Scope of the catalytic oxidation of aryl halides using N_2O

General procedure GP1: (using bipy-pyr-L50)

For the scope, oven- and heatgun-dried pressure Schlenks with Teflon screw-cap and equipped with a Teflon-coated stir bar were used. These Schlenks were brought into an argon filled glovebox, along with a heatgun-dried vial. Dried DMA was introduced into the vial. $NiBr_2$·diglyme (7.1 mg, 0.020 mmol, 10 mol%), bipy-pyr **L50** (6.7 mg, 0.030 mmol, 15 mol%), NaI (45 mg, 0.30 mmol, 1.5 equiv), aryl halide (if solid, 0.20 mmol, 1.0 equiv), and zinc (20 mg, 0.30 mmol, 1.5 equiv) were introduced in the pressure Schlenk. Then, outside the glovebox, using a T-connection, the Schlenk was evacuated and refilled with N_2O. This procedure was repeated three times. Then, under 1.5 atm of N_2O, the Schlenk was opened and DMA (0.5 mL) was added using a syringe *while stirring*. *(Stirring is important to avoid formation of agglomerates.)* If the aryl halide was liquid, it was added at that time using the adapted size of Hamilton syringe. The Schlenk was then closed, and the pressure of N_2O was increased to 1.9 – 2.0 atm. The reaction mixture was kept stirring at 1000 rpm, for 20 to 24 h, at RT.

After the reaction, the Schlenk was carefully opened, and the reaction was first diluted using 2 mL of Et_2O or EtOAc depending on the solubility of the expected product. The mixture was quenched first with water (the quenching can be exothermic) followed by HCl (1 M), and extracted 3 times with Et_2O (12 to 15 mL). The combined organic layers were washed with brine, dried over $MgSO_4$, filtered and concentrated under reduced pressure. The crude was purified by column chromatography over silica gel using gradient of solvents.

General procedure 2: (terpy conditions)

The sole modifications for these conditions are highlighted here: $NiBr_2$·diglyme (7.1 mg, 0.020 mmol, 10 mol%), **terpy L18 (7.0 mg, 0.030 mmol, 15 mol%), NaI (60 mg, 0.40 mmol, 2.0 equiv)**, aryl halide (if solid, 0.20 mmol, 1.0 equiv), and **zinc (32.7 mg, 0.500 mmol, 2.5 equiv)** were introduced in the pressure Schlenk.

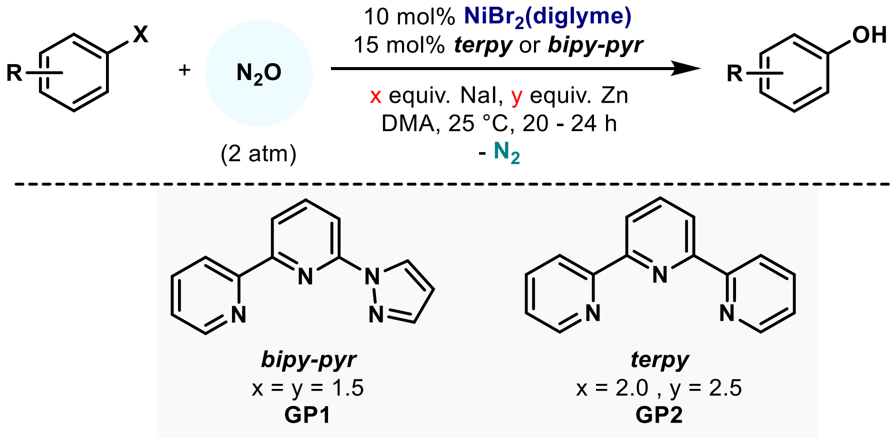

6.1. Scope of aryl iodides

Phenol (**14**)

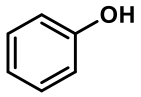

14, 82%, (89%)

Following general procedure **GP1**, using phenyl iodide (22 μL, 0.20 mmol, 1.0 equiv) as starting material, 89% NMR yield of the expected product was observed using CH_2Br_2 as internal standard. Purification was performed using pentane:Et_2O (10:1 to 8:1) as eluent, affording 15.5 mg (0.165 mmol, 82% yield) of phenol (**14**) as a white solid. As a comparison, using **GP2**, 80% NMR yield was observed.

Rf (Pentane:Et_2O = 8:2) 0.3.

^{1}H NMR (400 MHz, $CDCl_3$) δ 7.28 – 7.22 (m, 2H, Ar*H*), 6.94 (m, 1H, Ar*H*), 6.87 – 6.81 (m, 2H, Ar*H*), 4.73 (br s, 1H, O*H*).

^{13}C NMR (101 MHz, $CDCl_3$) δ 155.5, 129.8, 121.0, 115.4.

The values of the NMR spectra are in accordance with reported literature data.[80]

4-Fluorophenol (**9**)

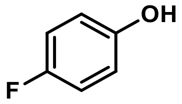

9, 80%

Following general procedure **GP1**, using 4-fluoro-phenyl iodide (23 μL, 0.20 mmol, 1.0 equiv) as starting material, 84% NMR yield was observed, using CH_2Br_2 as internal standard. Purification was performed using pentane:Et_2O (20:1 to 8:1) as eluent, affording 18.0 mg (0.161 mmol, 80% yield) of phenol (**9**) was obtained as a pale yellow solid. As a comparison, using **GP2**, 75% NMR yield was observed.

^{1}H NMR (400 MHz, $CDCl_3$) δ 7.00 – 6.86 (m, 2H, Ar*H*), 6.83 – 6.69 (m, 2H, Ar*H*), 4.77 (br s, 1H, O*H*).

^{13}C NMR (101 MHz, $CDCl_3$) δ 157.4 (d, *J* = 238 Hz), 151.5 (d, *J* = 2.0 Hz), 116.4 (d, *J* = 8.0 Hz), 116.1 (d, *J* = 23.0 Hz).

^{19}F NMR (282 MHz, $CDCl_3$) δ -124.3.

The values of the NMR spectra are in accordance with reported literature data.[80]

4-Chlorophenol (**15**)

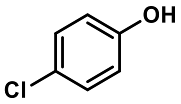

15, 75%

Following general procedure **GP1**, using 1-chloro-4-iodobenzene (47.7 mg, 0.200 mmol, 1.0 equiv) as starting material, 19.3 mg (0.150 mmol, 75% yield) of 4-chlorophenol (**15**) was obtained as a light yellow solid after purification using pentane:Et_2O (19:1 to 10:1) as eluent.

^{1}H NMR (400 MHz, $CDCl_3$) δ 7.23 – 7.14 (m, 2H, Ar*H*), 6.82 – 6.69 (m, 2H, Ar*H*), 4.74 (brs, 1H, O*H*).

^{13}C NMR (101 MHz, $CDCl_3$) δ 154.2, 129.7, 125.9, 116.8.

The values of the NMR spectra are in accordance with reported literature data.[81]

4-Bromophenol (**16**)

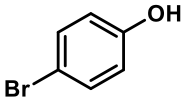

16, 59%

Following general procedure **GP1**, using 1-bromo-4-iodobenzene (56.6 mg, 0.200 mmol, 1.0 equiv) as starting material, 20.4 mg (0.118 mmol, 59% yield) of 4-bromophenol (**16**) was obtained as a light yellow solid after purification using pentane:Et_2O (19:1 to 10:1) as eluent.

^{1}H NMR (300 MHz, $CDCl_3$) δ 7.37 – 7.30 (m, 2H, Ar*H*), 6.77 – 6.67 (m, 2H, Ar*H*), 4.82 (br s, 1H, O*H*).

^{13}C NMR (75 MHz, $CDCl_3$) δ 154.8, 132.6, 117.3, 113.1.

The values of the NMR spectra are in accordance with reported literature data.[80]

3-Fluorophenol (**17**)

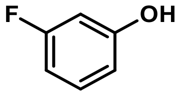

17, 82%

Following general procedure **GP1**, using 1-fluoro-3-iodobenzene (23.5 μL, 0.200 mmol, 1.0 equiv) as starting material, 18.4 mg (0.164 mmol, 82% yield) of 3-fluorophenol (**17**) was obtained as a light yellow oil after purification using pentane:Et_2O (10:1 to 4:1) as eluent.

^{1}H NMR (400 MHz, CDCl$_3$) δ 7.18 (td, *J* = 8.1, 6.6 Hz, 1H, Ar*H*), 6.69 – 6.54 (m, 3H, Ar*H*), 5.00 (br s, 1H, O*H*).
^{13}C NMR (101 MHz, CDCl$_3$) δ 163.8 (d, *J* = 245.5 Hz), 156.9 (d, *J* = 11.1 Hz), 130.6 (d, *J* = 10.0 Hz), 111.3 (d, *J* = 2.8 Hz), 107.9 (d, *J* = 21.5 Hz), 103.4 (d, *J* = 24.5 Hz).
^{19}F NMR (282 MHz, CDCl$_3$) δ -111.8.
The values of the NMR spectra are in accordance with reported literature data.[82]

3-Chlorophenol (**18**)

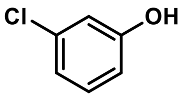

18, 74%

Following general procedure **GP1**, using 1-chloro-3-iodobenzene (24.7 µL, 0.200 mmol, 1.0 equiv) as starting material, 19.0 mg (0.148 mmol, 74% yield) of 3-chlorophenol (**18**) was obtained as a light yellowish oil after purification using pentane:Et$_2$O (10:1 to 4:1) as eluent.
^{1}H NMR (400 MHz, CDCl$_3$) δ 7.16 (t, *J* = 8.1 Hz, 1H, Ar*H*), 6.93 (ddd, *J* = 8.0, 1.9, 0.9 Hz, 1H, Ar*H*), 6.86 (t, *J* = 2.2 Hz, 1H, Ar*H*), 6.72 (ddd, *J* = 8.2, 2.5, 0.9 Hz, 1H, Ar*H*), 4.94 (br s, 1H, O*H*).
^{13}C NMR (101 MHz, CDCl$_3$) δ 156.3, 135.1, 130.6, 121.3, 116.1, 113.9.
The values of the NMR spectra are in accordance with reported literature data.[81]

3-Bromophenol (**19**)

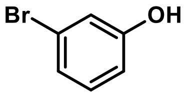

19, 69%

Following general procedure **GP1**, using 1-bromo-3-iodobenzene (25.5 µL, 0.200 mmol, 1.0 equiv) as starting material, 24 mg (0.14 mmol, 69% yield) of 3-bromophenol (**19**) was obtained as a light yellowish oil after purification using pentane:Et$_2$O (15:1 to 10:1) as eluent.
^{1}H NMR (400 MHz, CDCl$_3$) δ 7.13 – 7.05 (m, 2H, Ar*H*), 7.03 – 7.01 (m, 1H, Ar*H*), 6.77 (ddd, *J* = 7.6, 2.5, 1.5 Hz, 1H, Ar*H*), 4.10 (br s, H, O*H*).
^{13}C NMR (101 MHz, CDCl$_3$) δ 156.4, 130.9, 124.1, 122.9, 118.9, 114.4.
The values of the NMR spectra are in accordance with reported literature data.[31]

4-(Trifluoromethyl)phenol (**7**)

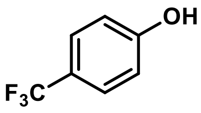

7
75% from ArI
65% from ArBr

Following general procedure **GP1**, using 1-iodo-4-(trifluoromethyl)benzene (29.4 μL, 0.200 mmol, 1.0 equiv) as starting material, 24.3 mg (0.150 mmol, 75% yield) of 4-(trifluoromethyl)phenol (**7**) was obtained as a light yellow solid after purification using pentane:Et_2O (19:1 to 10:1) as eluent.

Following general procedure **GP1**, using 1-bromo-4-(trifluoromethyl)benzene (26 μL, 0.20 mmol, 1.0 equiv) as starting material, 21.1 mg (0.130 mmol, 65% yield) of 4-(trifluoromethyl)phenol (**7**) was obtained as a light yellow solid after purification using pentane:Et_2O (19:1 to 10:1) as eluent.

^{1}H NMR (300 MHz, $CDCl_3$) 7.64 – 7.39 (m, 2H, Ar*H*), 7.02 – 6.80 (m, 2H, Ar*H*), 5.15 (br s, 1H, O*H*).

^{13}C NMR (101 MHz, $CDCl_3$) δ 158.2, 127.3 (q, *J* = 3.7 Hz), 124.5 (d, *J* = 271 Hz). 123.4 (q, *J* = 32.9 Hz), 115.6.

^{19}F NMR (282 MHz, $CDCl_3$) δ -61.6.

The values of the NMR spectra are in accordance with reported literature data.[81]

1-(4-Hydroxyphenyl)ethan-1-one (**20**)

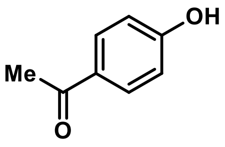

20
74% from ArI
84% from ArBr

Following general procedure **GP1**, using 4-iodo-acetophenone (49.2 mg, 0.200 mmol, 1.0 equiv) as starting material, 20.1 mg (0.148 mmol, 74% yield) of 1-(4-hydroxyphenyl)ethan-1-one (**20**) was obtained as a white solid after purification (2 columns, first one short using pentane:Et_2O (10:1 to 5:1) then 2nd column using DCM to DCM/EtOAc 95:5 as eluent).

Following general procedure **GP1**, using 4-bromo-acetophenone (39.9 mg, 0.200 mmol, 1.0 equiv) as starting material, 22.8 mg (0.167 mmol, 84% yield) of 1-(4-hydroxyphenyl)ethan-1-one (**20**) was obtained as a white solid after purification using pentane:Et_2O (4:1 to 2:1).

¹H NMR (400 MHz, CDCl$_3$) δ 7.96 – 7.87 (m, 2H, Ar*H*), 6.97 – 6.88 (m, 2H, Ar*H*), 2.59 (s, 3H, *Me*).

¹³C NMR (101 MHz, CDCl$_3$) δ 198.4, 161.2, 131.3, 129.9, 115.6, 26.5.

The values of the NMR spectra are in accordance with reported literature data.[81]

N.B.: a very broad, almost in the baseline, signal at 7.22 ppm could be attributed to the O*H.*

Methyl 4-hydroxybenzoate (Paraben, 21)

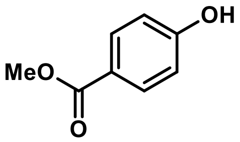

21
68% from ArI
75% from ArBr

Following general procedure **GP2**, using methyl 4-iodobenzoate (52.4 mg, 0.200 mmol, 1.0 equiv) as starting material, 20.7 mg (0.136 mmol, 68% yield) of methyl 4-hydroxybenzoate (paraben, **21**) was obtained as a white solid after purification using pentane:Et$_2$O gradient (from 9:1 to 7:3) as eluent.

Following general procedure **GP1**, using methyl 4-bromobenzoate (43 mg, 0.20 mmol, 1.0 equiv) as starting material, 22.8 mg (0.150 mmol, 75% yield) of methyl 4-hydroxybenzoate (paraben, **21**) was obtained as a white solid after purification using pentane:Et$_2$O gradient (from 9:1 to 7:3) as eluent.

¹H NMR (300 MHz, CDCl$_3$) δ 8.02 – 7.91 (m, 2H, Ar*H*), 6.90 – 6.83 (m, 2H, Ar*H*), 5.83 (br s, 1H, O*H*), 3.90 (s, 3H, O*Me*).

¹³C NMR (75 MHz, CDCl$_3$) δ 167.3, 160.1, 132.1, 122.8, 115.4, 52.2.

The values of the NMR spectra are in accordance with reported literature data.[83]

N.B.: The aryliodide substrate was passed through a column of silica first, using DCM for elution, in order to remove brown impurity. A pure white solid is obtained at the end.

4-Hydroxybenzonitrile (22)

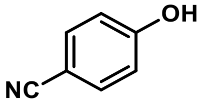

22
67% from ArI
78% from ArBr

Following general procedure **GP1**, using 4-iodo-benzonitrile (45.8 mg, 0.200 mmol, 1.0 equiv) as starting material, 15.9 mg (0.133 mmol, 67% yield) of 4-hydroxybenzonitrile (**22**) was obtained as a white solid after purification using DCM:EtOAc (99:1 to 97:3) as eluent. *No HCl was used during workup, and only water instead.*

Following general procedure **GP1**, using 4-bromo-benzonitrile (36.4 mg, 0.200 mmol, 1.0 equiv) as starting material, 18.6 mg (0.156 mmol, 78% yield) of 4-hydroxybenzonitrile (**22**) was obtained as a white solid after purification using DCM:EtOAc (99:1 to 97:3) as eluent. *No HCl was used during workup, and only water instead.*

^{1}H NMR (400 MHz, $CDCl_3$) δ 7.66 – 7.47 (m, 2H, Ar*H*), 7.02 – 6.83 (m, 2H, Ar*H*), 6.62 (br s, 1H, O*H*).

^{13}C NMR (101 MHz, $CDCl_3$) δ 160.3, 134.5, 119.4, 116.6, 103.2.

The values of the NMR spectra are in accordance with reported literature data.[81]

3-Hydroxybenzonitrile (23)

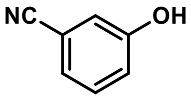

23, 58%

Following general procedure **GP1**, using 3-iodo-benzonitrile (45.8 mg, 0.200 mmol, 1.0 equiv) as starting material, 13.8 mg (0.116 mmol, 58% yield) of 3-hydroxybenzonitrile (**23**) was obtained as a light yellowish oil after purification using pentane:Et_2O (9:1 to 3:1) as eluent. *No HCl was used during the workup, and only water instead.*

^{1}H NMR (400 MHz, $CDCl_3$) δ 7.27 (ddd, *J* = 8.2, 7.5, 0.5 Hz, 1H, Ar*H*), 7.15 (dt, *J* = 7.6, 1.2 Hz, 1H, Ar*H*), 7.10 – 7.03 (m, 2H, Ar*H*), 6.37 (br s, 1H, O*H*).

^{13}C NMR (101 MHz, $CDCl_3$) δ 156.4, 130.7, 124.6, 121.1, 118.9, 118.7, 112.6.

HRMS (ESI$^-$): calcd for $C_7H_4N_1O_1^-$ $[M-H]^-$ 118.02984; found 118.02997.

The values of the NMR spectra are in accordance with reported literature data.[31]

Methyl 3-(4-hydroxyphenyl)propanoate (**24**)

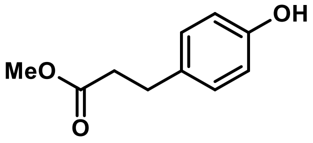

24, 81%

Following general procedure **GP1**, using methyl 3-(4-iodophenyl)propanoate (58 mg, 0.20 mmol, 1.0 equiv) as starting material, 29.1 mg (0.161 mmol, 81% yield) of methyl 3-(4-hydroxyphenyl)propanoate (**24**) was obtained as a light yellow solid after purification using pentane:Et_2O (7:1 to 3:1) as eluent.

Rf (Pentane:Et_2O (6:4)) 0.40.

^{1}H NMR (400 MHz, $CDCl_3$) δ 7.01 – 6.95 (m, 2H, Ar*H*), 6.70 – 6.65 (m, 2H, Ar*H*), 5.15 (br s, 1H, OH), 3.60 (s, 3H, *Me*), 2.81 (t, *J* = 7.7 Hz, 2H, ArCH_2), 2.53 (t, *J* = 7.7 Hz, 2H, ArCH_2CH_2).

^{13}C NMR (101 MHz, $CDCl_3$) δ 173.9, 154.2, 132.6, 129.5, 115.5, 51.8, 36.2, 30.2.

The values of the NMR spectra are in accordance with reported literature data.[84]

4-Pentylphenol (**25**)

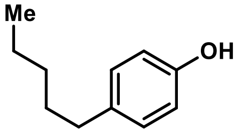

25, 79%

Following general procedure **GP1**, using 1-iodo-4-(pentyl)benzene (39.7 µL, 0.200 mmol, 1.0 equiv) as starting material, 26.0 mg (0.158 mmol, 79% yield) of 4-pentylphenol (**25**) was obtained as a light yellow oil after purification using pentane:Et_2O (95:5 to 9:1) as eluent.

Rf (Pentane:Et_2O (9:1)) 0.25.

^{1}H NMR (300 MHz, $CDCl_3$) δ 7.09 – 7.02 (m, 2H, Ar*H*), 6.79 – 6.73 (m, 2H, Ar*H*), 4.67 (br s, 1H, O*H*), 2.58 – 2.49 (m, 2H, ArCH_2), 1.65 – 1.53 (m, 2H, ArCH_2CH_2), 1.40 – 1.25 (m, 4H, ArCH_2CH_2CH_2CH_2), 0.94 – 0.86 (m, 3H, *Me*).

^{13}C NMR (75 MHz, $CDCl_3$) δ 153.5, 135.4, 129.6, 115.2, 35.2, 31.6, 31.5, 22.7, 14.2.

The values of the NMR spectra are in accordance with reported literature data.[85]

[1,1'-Biphenyl]-4-ol (**26**)

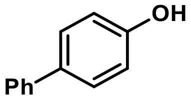

26, 73%

Following general procedure **GP2**, using 1-iodo-4-(phenyl)benzene (56 mg, 0.20 mmol, 1.0 equiv) as starting material, 24.8 mg (0.146 mmol, 73% yield) of [1,1'-Biphenyl]-4-ol (**26**) was obtained as a white solid after purification using hexane:EtOAc (10:1 to 8:2) as eluent.

^{1}**H NMR (400 MHz, CDCl$_3$)** 7.58 – 7.52 (m, 2H, Ar*H*), 7.52 – 7.46 (m, 2H, Ar*H*), 7.45 – 7.38 (m, 2H, Ar*H*), 7.35 – 7.28 (m, 1H, Ar*H*), 6.97 – 6.86 (m, 2H, Ar*H*), 4.73 (br s, 1H, O*H*).
^{13}**C NMR (101 MHz, CDCl$_3$)** δ 155.2, 140.9, 134.2, 128.9, 128.5, 126.9, 115.8.
The values of the NMR spectra are in accordance with reported literature data.[81]

4-Methoxyphenol (Mequinol, 27)

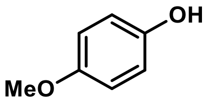

27, 81%

Following general procedure **GP1**, using 4-iodo-anisole (46.8 mg, 0.200 mmol, 1.0 equiv) as starting material, 20.2 mg (0.163 mmol, 81% yield) of 4-methoxyphenol (**27**) was obtained as a white solid after purification using pentane:Et$_2$O (10:1 to 4:1) as eluent.
^{1}**H NMR (400 MHz, CDCl$_3$)** δ 6.82 – 6.74 (m, 4H, Ar*H*), 4.55 (br s, 1H, O*H*), 3.77 (s, 3H, *Me*).
^{13}**C NMR (101 MHz, CDCl$_3$)** δ 153.9, 149.6, 116.2, 115.0, 55.9.
The values of the NMR spectra are in accordance with reported literature data.[81]

4-(Methylthio)phenol (**28**)

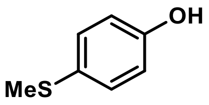

28, 61%

Following general procedure **GP1**, using 4-iodo-thioanisole (50 mg, 0.20 mmol, 1.0 equiv) as starting material, 17.1 mg (0.122 mmol, 61% yield) of 4-(methylthio)phenol (**28**) was obtained as a white solid after purification using pentane:Et$_2$O (9:1 to 4:1) as eluent. *Detection of 6-7% of the corresponding sulfoxide in the crude NMR.*
^{1}**H NMR (300 MHz, CDCl$_3$)** δ 7.26 – 7.19 (m, 2H, Ar*H*), 6.82 – 6.75 (m, 2H, Ar*H*), 4.65 (br s, 1H, O*H*), 2.44 (s, 3H, *Me*).
^{13}**C NMR (75 MHz, CDCl$_3$)** δ 154.2, 130.6, 129.1, 116.2, 18.2.
The values of the NMR spectra are in accordance with reported literature data.[86]

9*H*-Fluoren-2-ol (29)

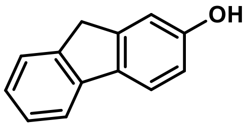

29, 38%

Following general procedure **GP2**, using 2-iodo-9*H*-fluorene (58.4 mg, 0.200 mmol, 1.0 equiv) as starting material, 14 mg (0.077 mmol, 38% yield) of 9*H*-Fluoren-2-ol (**29**) was obtained as a white solid after purification using pentane:Et_2O (10:1 to 8:1) as eluent, followed by a short column using only DCM as eluent to remove colorful impurities.

Rf (DCM) 0.25.

^{1}H NMR (400 MHz, $CDCl_3$) δ 7.68 (d, *J* = 7.5 Hz, 1H, Ar*H*), 7.64 (d, *J* = 8.2 Hz, 1H, Ar*H*), 7.50 (dt, *J* = 7.5, 1.0 Hz, 1H, Ar*H*), 7.34 (td, *J* = 7.5, 1.0 Hz, 1H, Ar*H*), 7.24 (td, *J* = 7.5, 1.2 Hz, 1H, Ar*H*), 7.03 (dd, *J* = 2.5, 1.2 Hz, 1H, Ar*H*), 6.85 (dd, *J* = 8.2, 2.4 Hz, 1H, Ar*H*), 4.71 (br s, 1H, O*H*), 3.85 (s, 2H, CH_2).

^{13}C NMR (101 MHz, $CDCl_3$) δ 155.1, 145.5, 142.7, 141.7, 135.1, 126.9, 125.8, 125.0, 120.8, 119.2, 114.2, 112.3, 37.0.

The values of the NMR spectra are in accordance with reported literature data.[31]

2-Chlorophenol (30)

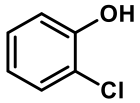

30, 40%, (50%)

Following general procedure **GP1**, using 1-chloro-2-iodobenzene (24.4 µL, 0.200 mmol, 1.0 equiv) as starting material, 10 mg (0.080 mmol, 40% yield) of 2-chlorophenol (**30**) was obtained as a white solid after purification using pentane:Et_2O (1:0 to 8:1) as eluent. This compound is relatively volatile, and the isolated yield (40%) is quite lower than the NMR yield on the crude (50%).

^{1}H NMR (300 MHz, $CDCl_3$) δ 7.32 (dd, *J* = 8.0, 1.6 Hz, 1H, Ar*H*), 7.18 (ddd, *J* = 8.2, 7.4, 1.6 Hz, 1H, Ar*H*), 7.02 (dd, *J* = 8.2, 1.6 Hz, 1H, Ar*H*), 6.87 (ddd, *J* = 8.0, 7.3, 1.6 Hz, 1H, Ar*H*), 5.53 (br s, 1H, O*H*).

^{13}C NMR (75 MHz, $CDCl_3$) δ 151.5, 129.1, 128.6, 121.5, 120.0, 116.4.

The values of the NMR spectra are in accordance with reported literature data.[81]

4-Hydroxy-2,3-dihydro-1*H*-inden-1-one (**31**)

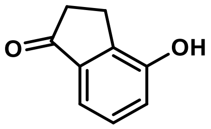

31, 43%

Following general procedure **GP1**, using 4-iodo-2,3-dihydro-1*H*-inden-1-one (51.6 mg, 0.200 mmol, 1.0 equiv) as starting material, 12.8 mg (0.0864 mmol, 43% yield) of 4-hydroxy-2,3-dihydro-1*H*-inden-1-one (**31**) was obtained as a white solid after purification using DCM:EtOAc (1:0 to 97:3) as eluent.

Rf (DCM:EtOAc (98:2)) 0.12.

^{1}H NMR (300 MHz, $CDCl_3$) δ 7.38 (d, *J* = 7.7 Hz, 1H, Ar*H*), 7.29 (t, *J* = 7.6 Hz, 1H, Ar*H*), 7.01 (dd, *J* = 7.7, 1.0 Hz, 1H, Ar*H*), 4.92 (br s, 1H, O*H*), 3.13 – 3.01 (m, 2H $ArCH_2$), 2.75 – 2.70 (m, 2H, $ArCH_2CH_2$).

^{13}C NMR (101 MHz, $CDCl_3$) δ 207.0, 154.6, 141.7, 138.1, 128.1, 119.7, 113.7, 35.8, 22.0.

N.B. the solubility in $CDCl_3$ is low, so 2 drops of DMSO-d_6 were added to record the ^{13}C spectrum.

The values of the ^{1}H NMR spectrum are in accordance with reported literature data.[87]

HRMS (ESI$^-$): calcd for $C_9H_7O_2^-$ [M-H]$^-$ 147.04515; found 147.04525.

Guaiacol (32)

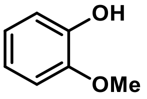

32, 79%

Following general procedure **GP1**, using 2-iodoanisole (26.1 µL, 0.200 mmol, 1.0 equiv) as starting material, 19.6 mg (0.158 mmol, 79% yield) of guaiacol (**32**) was obtained as a white solid after purification using pentane:Et_2O (10:1 to 8:1) as eluent.

Rf (Pentane:Et_2O (7:3)) 0.45.

^{1}H NMR (400 MHz, $CDCl_3$) δ 6.96 – 6.91 (m, 1H, Ar*H*), 6.91 – 6.84 (m, 3H, Ar*H*), 5.61 (br s, 1H, O*H*), 3.89 (s, 3H, *Me*).

^{13}C NMR (101 MHz, $CDCl_3$) δ 146.7, 145.8, 121.6, 120.3, 114.7, 110.8, 56.0.

The values of the NMR spectra are in accordance with reported literature data.[88]

4-(((Triisopropylsilyl)oxy)methyl)phenol (33)

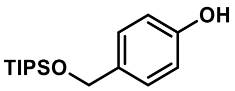

33, 55%

Following general procedure **GP1**, using ((4-iodobenzyl)oxy)triisopropylsilane (78.1 mg, 0.200 mmol, 1.0 equiv) as starting material, 31 mg (0.11 mmol, 55% yield) of 4-(((triisopropylsilyl)oxy)methyl)phenol (**33**) was obtained as a colorless sticky oil after purification using pentane:Et_2O (10:1 to 8:1) as eluent.

Rf (Pentane:Et_2O (7:3)) 0.40.

^{1}H NMR (400 MHz, $CDCl_3$) δ 7.24 – 7.18 (m, 2H, Ar*H*), 6.83 – 6.75 (m, 2H, Ar*H*), 4.76 (s, 2H, ArC*H$_2$*), 4.72 (br s, 1H, O*H*), 1.11 – 1.04 (m, 21H, *TIPS*).

^{13}C NMR (101 MHz, $CDCl_3$) δ 154.5, 134.2, 127.5, 115.1, 64.9, 18.2, 12.2.

HRMS (ESI$^+$): calcd for $C_{16}H_{28}O_2Si_1Na_1^+$ $[M+Na]^+$ 303.17508; found 303.17538.

The values of the NMR spectra are in accordance with reported literature data.[89]

Diethyl (4-hydroxybenzyl)phosphonate (34)

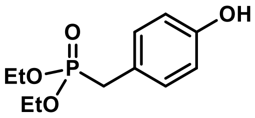

34, 41%, (50%)

Following general procedure **GP1**, using diethyl (4-iodobenzyl)phosphonate (70.8 mg, 0.200 mmol, 1.0 equiv) as starting material, NMR yield indicated 50% from the crude, and 20 mg (0.082 mmol, 41% yield) of diethyl (4-hydroxybenzyl)phosphonate (**34**) was obtained as a solid after tedious purification using DCM:MeOH (1:0 to 95:5) as eluent.

^{1}H NMR (400 MHz, $CDCl_3$) δ 7.03 (dq, *J* = 7.9, 3.0 Hz, 2H, Ar*H*), 6.65 – 6.60 (m, 2H, Ar*H*), 4.03 (dqd, *J* = 8.3, 7.0, 3.8 Hz, 4H, OC*H$_2$*CH$_3$), 3.06 (d, *J* = 20.9 Hz, 2H, ArC*H$_2$*), 1.26 (t, *J* = 7.1 Hz, 6H, OCH$_2$C*H$_3$*).

^{13}C NMR (101 MHz, $CDCl_3$) δ 156.2 (d, *J* = 3.6 Hz), 130.8 (d, *J* = 6.5 Hz), 121.3 (d, *J* = 9.4 Hz), 116.2 (d, *J* = 3.0 Hz), 62.6 (d, *J* = 7.0 Hz), 32.7 (d, *J* = 139.8 Hz), 16.5 (d, *J* = 5.9 Hz).

^{31}P NMR (162 MHz, $CDCl_3$) δ 27.54.

The values of the NMR spectra are in accordance with reported literature data.[90]

tert-Butyl 5-hydroxy-1H-indole-1-carboxylate (**35**)

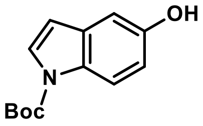

35, 74%

Following general procedure **GP1**, using *tert*-butyl 5-iodo-1*H*-indole-1-carboxylate (68.6 mg, 0.200 mmol, 1.0 equiv) as starting material, 34.7 mg (0.149 mmol, 74% yield) of *tert*-butyl 5-hydroxy-1*H*-indole-1-carboxylate (**35**) was obtained as a sticky light yellow foam after purification using hexane:EtOAc (10:1 to 8:1) as eluent.

Rf (Hexane:EtOAc (8:2)) 0.40.

^{1}H NMR (400 MHz, $CDCl_3$) δ 7.98 (br d, *J* = 8.7 Hz, 1H, Ar*H*), 7.57 (br d, *J* = 3.6 Hz, 1H, Ar*H*), 6.99 (d, *J* = 2.5 Hz, 1H, Ar*H*), 6.85 (dd, *J* = 8.8, 2.5 Hz, 1H, Ar*H*), 6.45 (dd, *J* = 3.7, 0.8 Hz, 1H, Ar*H*), 4.84 (br s, 1H, O*H*), 1.67 (s, 9H, *tBu*).

^{13}C NMR (101 MHz, $CDCl_3$) δ 151.6, 149.9, 131.8, 130.2, 126.9, 116.0, 113.1, 107.1, 106.2, 83.8, 28.3.

The values of the ^{1}H NMR spectrum are in accordance with reported literature data.[91]

6-Hydroxy-quinoline (**36**)

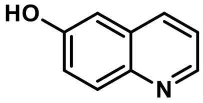

36, 38%

Following general procedure **GP1**, using 6-iodo-quinoline (51 mg, 0.20 mmol, 1.0 equiv) as starting material, 11 mg (0.076 mmol, 38% yield) of 6-hydroxy-quinoline (**36**) was obtained as a white solid after purification using hexane:EtOAc (2:1 to 1:1) as eluent.

Rf (Hexane:EtOAc (1:1)) 0.20

^{1}H NMR (400 MHz, DMSO-d_6) δ 9.99 (s, 1H, O*H*), 8.65 (dd, *J* = 4.2, 1.7 Hz, 1H, Ar*H*), 8.13 (dd, *J* = 8.4, 1.7 Hz, 1H, Ar*H*), 7.86 (d, *J* = 9.1 Hz, 1H, Ar*H*), 7.39 (dd, *J* = 8.3, 4.2 Hz, 1H, Ar*H*), 7.31 (dd, *J* = 9.1, 2.7 Hz, 1H, Ar*H*), 7.14 (d, *J* = 2.7 Hz, 1H, Ar*H*).

^{13}C NMR (101 MHz, DMSO-d_6) δ 155.4, 147.1, 143.0, 134.1, 130.4, 129.3, 121.9, 121.4, 108.3.

The values of the NMR spectra are in accordance with reported literature data.[92]

9-Phenyl-9*H*-carbazol-3-ol (37)

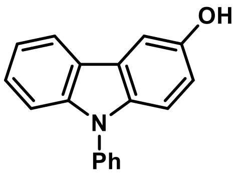

37, 51%

Following general procedure **GP1**, using 3-bromo-9-phenyl-9*H*-carbazole (62.2 mg, 0.200 mmol, 1.0 equiv) as starting material, 26.4 mg (0.102 mmol, 51% yield) of 9-phenyl-9*H*-carbazol-3-ol (**37**) was obtained as a colorless oil after purification using hexanes:EtOAc (5:1) as eluent.

Rf (Hexanes:EtOAc (5:1)) 0.20.

^{1}H NMR (300 MHz, CDCl$_3$) δ 8.02 (dt, *J* = 7.8, 1.0 Hz, 1H, Ar*H*), 7.63 – 7.49 (m, 5H, Ar*H*), 7.48 – 7.33 (m, 3H, Ar*H*), 7.30 – 7.15 (m, 2H, Ar*H*), 6.94 (dd, *J* = 8.8, 2.5 Hz, 1H, Ar*H*), 4.84 (s, 1H, O*H*).

^{13}C NMR (75 MHz, CDCl$_3$) δ 149.8, 141.6, 138.0, 136.1, 130.0, 127.4, 127.1, 126.2, 124.2, 123.1, 120.5, 119.7, 115.0, 110.6, 110.0, 105.9.

The values of the NMR spectra are in accordance with reported literature data.[93]

Dibenzo[b,d]thiophen-2-ol (38)

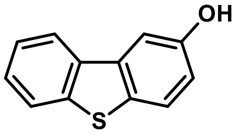

38, 37%

Following general procedure **GP1**, using 2-bromodibenzo[b,d]thiophene (52.4 mg, 0.200 mmol, 1.0 equiv) as starting material, 14.8 mg (0.074 mmol, 37% yield) of dibenzo[b,d]thiophen-2-ol (**38**) was obtained as a white solid after purification using hexanes:EtOAc (5:1) as eluent.

Rf (Hexanes:EtOAc (5:1)) 0.20.

^{1}H NMR (300 MHz, DMSO-d_6) δ 9.63 (s, 1H, O*H*), 8.28 – 8.15 (m, 1H, ArH), 7.99 – 7.88 (m, 1H, ArH), 7.78 (d, *J* = 8.6 Hz, 1H, ArH), 7.66 (d, *J* = 2.4 Hz, 1H, ArH), 7.53 – 7.40 (m, 2H, ArH), 7.02 (dd, *J* = 8.6, 2.4 Hz, 1H, ArH).

^{13}C NMR (75 MHz, DMSO-d_6) δ 155.4, 139.6, 136.3, 134.9, 128.5, 126.8, 124.3, 123.6, 123.0, 121.9, 116.6, 107.4.

The values of the NMR spectra are in accordance with reported literature data. [94]

4-(Diphenylamino)phenyl pivalate (**39**)

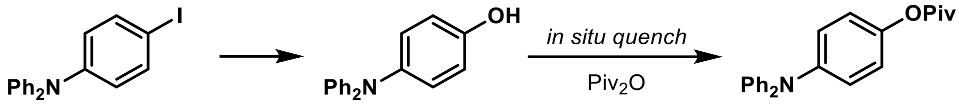

S52 **S53** *unstable towards oxidation* **39**, 68%

Following general procedure **GP1**, using 4-iodo-triphenylamine (74.2 mg, 0.200 mmol, 1.0 equiv) as starting material, the reaction was stirred for 22 h under N_2O atmosphere. Then, the pressure Schlenk was then carefully open, placed under argon, and pivalic anhydride (122 μL, 0.600 mmol, 3.0 equiv) was added to the crude reaction mixture and stirred for additional 2 h 30 min at 50 °C. After that time, 10 mL of water and drops of $NaHCO_3$ were added and the crude was extracted 3 times using 12 mL of Et_2O. Then, the combined organic layers were washed using saturated $NaHCO_3$, dried over $MgSO_4$, filtered and concentrated under reduced pressure. Column chromatography over silica gel using full pentane to pentane:Et_2O (20:1) as eluent delivered 47.0 mg (0.136 mmol, 68% yield) of 4-(diphenylamino)phenyl pivalate (**39**) as obtained as a colorless oil.

Rf (Pentane:Et_2O (15:1) 0.45.

^{1}H NMR (400 MHz, $CDCl_3$) δ 7.29 – 7.20 (m, 4H, Ar*H*), 7.12 – 7.07 (m, 6H, Ar*H*), 7.03 – 6.93 (m, 4H, Ar*H*), 1.37 (s, 9H, *tBu*).

^{13}C NMR (101 MHz, $CDCl_3$) δ 177.4, 147.9, 146.5, 145.4, 129.4, 125.3, 124.1, 122.8, 122.3, 39.2, 27.3.

HRMS (ESI$^+$): calcd for $C_{23}H_{24}N_1O_2^+$ $[M+H]^+$ 346.180154; found 346.18022.

Ethyl 2-(4-hydroxyphenoxy)-2-methylpropanoate (40)

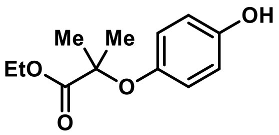

40, 78%

Following general procedure **GP1**, using ethyl 2-(4-iodophenoxy)-2-methylpropanoate (66.8 mg, 0.200 mmol, 1.0 equiv) as starting material, 35.0 mg (0.156 mmol, 78% yield) of ethyl 2-(4-hydroxyphenoxy)-2-methylpropanoate (**40**) was obtained as a off-white solid after purification using pentane:Et_2O (8:1 to 2:1) as eluent.

Rf (Pentane:Et_2O (7:3)) 0.20.

^{1}H NMR (300 MHz, CDCl$_3$) δ 6.84 – 6.75 (m, 2H, Ar*H*), 6.73 – 6.65 (m, 2H, Ar*H*), 4.75 (br s, 1H, O*H*), 4.24 (q, *J* = 7.1 Hz, 2H, C*H*$_2$CH$_3$), 1.53 (s, 6H, *Me*), 1.28 (t, *J* = 7.1 Hz, 3H, CH$_2$C*H*$_3$).

^{13}C NMR (75 MHz, CDCl$_3$) δ 174.6, 151.3, 149.1, 122.0, 115.8, 79.9, 61.5, 25.4, 14.2.

HRMS (ESI$^-$): calcd for $C_{12}H_{15}O_4^-$ [M-H]$^-$ 223.09758; found 223.09760.

The values of the NMR spectra are in accordance with reported literature data.[95]

4-(4,4,5,5-Tetramethyl-1,3,2-dioxaborolan-2-yl)phenol (41)

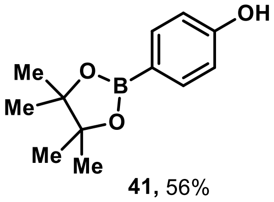

41, 56%

Following general procedure **GP1**, using 4-(4,4,5,5-tetramethyl-1,3,2-dioxaborolan-2-yl)phenyliodide (66 mg, 0.20 mmol, 1.0 equiv) as starting material, 24.7 mg (0.112 mmol, 56% yield) of 4-(4,4,5,5-tetramethyl-1,3,2-dioxaborolan-2-yl)phenol (**41**) was obtained as a white solid after purification using pentane:Et$_2$O (10:1 to 8:1) as eluent.

Rf (Hexanes:EtOAc (3:1)) 0.32.

^{1}H NMR (400 MHz, CDCl$_3$) δ 7.75 – 7.67 (m, 2H, Ar*H*), 6.87 – 6.78 (m, 2H, Ar*H*), 4.96 (br s, 1H, O*H*), 1.33 (s, 12H, *Me*).

^{13}C NMR (101 MHz, CDCl$_3$) δ 158.4, 136.9, 114.9, 83.8, 25.0.

^{11}B NMR (128 MHz, CDCl$_3$) δ 30.8.

The values of the NMR spectra are in accordance with reported literature data.[96]

N.B.: the carbon attached to the boron atom is not seen.

6.2. Scope of aryl bromides

5-Hydroxyisobenzofuran-1(3*H*)-one (42)

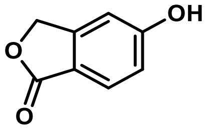

42, 58%

Following general procedure **GP1**, using 5-bromphthalide (42.6 mg, 0.200 mmol, 1.0 equiv) as starting material, 17.5 mg (0.117 mmol, 58% yield) of 5-hydroxyisobenzofuran-1(3*H*)-one (**42**) was obtained as a white solid after purification using hexanes:EtOAc (9:1 to 5:1) as eluent.

Rf (Hexanes:EtOAc (6:4) 0.30.

^{1}H NMR (400 MHz, DMSO-*d*$_6$) δ 10.64 (s, 1H, O*H*), 7.70 – 7.61 (m, 1H, Ar*H*), 6.96 – 6.92 (m, 2H, Ar*H*), 5.27 (s, 2H, C*H*$_2$).

^{13}C NMR (101 MHz, DMSO-*d*$_6$) δ 170.4, 163.1, 150.3, 126.7, 117.1, 115.6, 108.4, 69.0.

The values of the NMR spectra are in accordance with reported literature data.[97]

HRMS (ESI$^-$): calcd for $C_8H_5O_3^-$ [M-H]$^-$ 149.02442; found 149.02432.

Methyl 6-hydroxy-2-naphthoate (43)

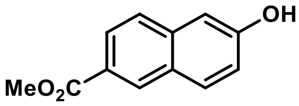

43, 65%

Following general procedure **GP1**, using methyl-6-bromo-2-naphthoate (53 mg, 0.20 mmol, 1.0 equiv) as starting material, 26.2 mg (0.130 mmol, 65% yield) of methyl-6-hydroxy-2-naphthoate (**43**) was obtained as a light brownish – off white solid after purification using DCM:EtOAc (99:1 to 97:3) as eluent followed by trituration in minimal amount of pentane/DCM.

Rf (DCM:EtOAc (98:2)) 0.22.

^{1}H NMR (300 MHz, CDCl$_3$) δ 8.59 – 8.44 (m, 1H, Ar*H*), 8.01 (dd, *J* = 8.6, 1.7 Hz, 1H, Ar*H*), 7.86 (dd, *J* = 8.5, 1.1 Hz, 1H, Ar*H*), 7.70 (d, *J* = 8.7 Hz, 1H, Ar*H*), 7.22 – 7.13 (m, 2H, Ar*H*), 5.44 (br s, 1H, O*H*), 3.97 (s, 3H, *Me*).

^{13}C NMR (75 MHz, CDCl$_3$) δ 167.6, 155.7, 137.3, 131.6, 131.2, 128.0, 126.7, 126.2, 125.4, 118.8, 109.7, 52.3.

N.B.: The solubility in CDCl$_3$ is not very good.

The values of the NMR spectra are in accordance with reported literature data.[98]

Ethyl (E)-3-(4-hydroxyphenyl)acrylate (44)

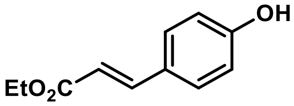

44, 78%

Following general procedure **GP1**, using ethyl (E)-3-(4-bromophenyl)acrylate (37.5 μL) as starting material, 30.0 mg (0.156 mmol, 78% yield) of ethyl (E)-3-(4-hydroxyphenyl)acrylate (**44**) was obtained as a white solid after purification using pentane:Et_2O (6:1 to 3:1) as eluent. No Z isomer was detected.

Rf (Pentane:Et_2O (7:3)) 0.2

^{1}H NMR (400 MHz, $CDCl_3$) δ 7.63 (d, *J* = 16.0 Hz, 1H, ArC*H*=CH), 7.47 – 7.39 (m, 2H, Ar*H*), 6.93 – 6.79 (m, 2H, Ar*H*), 6.30 (d, *J* = 15.9 Hz, 1H, ArCH=C*H*), 5.55 (br s, 1H, O*H*), 4.26 (q, *J* = 7.1 Hz, 2H, OCH_2CH_3), 1.34 (t, *J* = 7.1 Hz, 3H, OCH_2CH_3).

^{13}C NMR (101 MHz, $CDCl_3$) δ 167.8, 157.8, 144.6, 130.1, 127.4, 116.0, 115.8, 60.7, 14.5.

The values of the NMR spectra are in accordance with reported literature data.[99]

HRMS (ESI$^-$): calcd for $C_{11}H_{11}O_3^-$ [M-H]$^-$ 191.07137; found 191.07148.

4-(Methylsulfonyl)phenol (45)

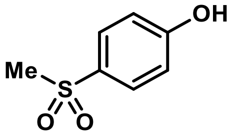

45, 82%

Following general procedure **GP1**, using 1-bromo-4-(methylsulfonyl)benzene (47 mg, 0.20 mmol, 1.0 equiv) as starting material, 28.2 mg (0.164 mmol, 82% yield) of 4-(methylsulfonyl)phenol (**45**) was obtained as a white solid after purification using hexanes:EtOAc (10:1 to 4:1) as eluent.

Rf (Hexanes:EtOAc (6:4)) 0.5

^{1}H NMR (400 MHz, $CDCl_3$) δ 7.80 – 7.74 (m, 2H, Ar*H*), 7.17 (s, 1H, O*H*), 7.01 – 6.95 (m, 2H, Ar*H*), 3.06 (s, 3H, *Me*).

^{13}C NMR (101 MHz, $CDCl_3$) δ 161.3, 131.3, 129.8, 116.4, 45.1.

The values of the NMR spectra are in accordance with reported literature data.[100]

6.3. Scope of complex molecules

(S)-4-Chloro-3-(4-((tetrahydrofuran-3-yl)oxy)benzyl)phenol (46)

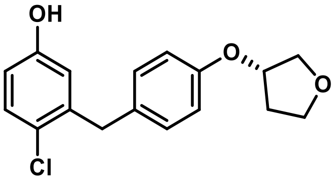

46, 68%

Following general procedure **GP1**, using (S)-3-(4-(2-chloro-5-iodobenzyl)phenoxy)tetrahydrofuran (82.9 mg, 0.200 mmol, 1.0 equiv) as starting material, 41.3 mg (0.136 mmol, 68% yield) of (S)-4-chloro-3-(4-((tetrahydrofuran-3-yl)oxy)benzyl)phenol (**46**) was obtained as a white solid after purification using DCM:EtOAc (1:0 to 95:5) as eluent, followed by a second column using pentane:EtOAc (10:1) as eluent.

Rf (DCM:EtOAc (98:2)) 0.15.

^{1}H NMR (400 MHz, $CDCl_3$) δ 7.21 (d, *J* = 8.6 Hz, 1H, ArH), 7.14 – 7.08 (m, 2H, ArH), 6.84 – 6.74 (m, 2H, ArH), 6.63 (dd, *J* = 8.6, 3.0 Hz, 1H, ArH), 6.54 (d, *J* = 3.0 Hz, 1H, ArH), 5.05 (br s, 1H, O*H*), 4.88 (ddt, *J* = 5.3, 4.2, 2.6 Hz, 1H, OC*H*), 4.01 – 3.92 (m, 5H, ArCH_2 + CH_2OCH_2), 3.89 (ddd, *J* = 8.4, 7.6, 4.8 Hz, 1H, CH_2OCH_2), 2.24 – 2.09 (m, 2H, OCH$_2$CH_2).

^{13}C NMR (101 MHz, $CDCl_3$) δ 156.1, 154.5, 140.5, 131.8, 130.4, 130.3, 125.5, 117.7, 115.6, 114.8, 77.4, 73.2, 67.3, 38.4, 33.1.

The values of the NMR spectra are in accordance with reported literature data.[101]

HRMS (ESI$^+$): calcd for $C_{17}H_{17}Cl_1O_3Na^+$ $[M+Na]^+$ 327.07584; found 327.07578.

4-Allyl-2-methoxyphenyl 4-hydroxybenzoate (47)

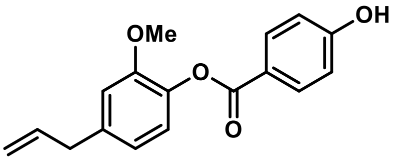

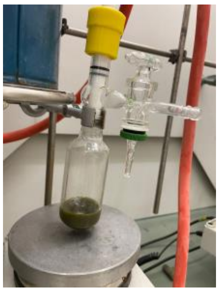

47
0.2 mmol scale: **84%**
5.0 mmol scale: 66%

Following general procedure **GP1**, using 4-allyl-2-methoxyphenyl 4-bromobenzoate (69.4 mg, 0.200 mmol, 1.0 equiv) as starting material, 47.6 mg (0.167 mmol, 84% yield) of 4-allyl-2-methoxyphenyl 4-hydroxybenzoate (**47**) was obtained as a white solid after purification using hexanes:EtOAc (10:1 to 8:2) as eluent.

Following general procedure **GP1** on *5 mmol scale in 100 mL Schlenk tube with 2.5 atm N_2O during 3 days*, using 4-allyl-2-methoxyphenyl 4-bromobenzoate (1.73 g, 5.00 mmol, 1.0 equiv) as starting material, 937 mg (3.30 mmol, 66% yield) of 4-allyl-2-methoxyphenyl 4-hydroxybenzoate (**47**) was obtained as a white solid after purification using hexanes:EtOAc (8:1 to 4:1) as eluent.

M.p.: 166.8 – 168.8 °C.

Rf (Hexanes:EtOAc (8:2)) 0.25.

^{1}H NMR (400 MHz, $CDCl_3$) δ 8.13 – 8.08 (m, 2H, ArH), 7.05 (dd, *J* = 7.7, 0.5 Hz, 1H, ArH), 6.89 – 6.85 (m, 2H, ArH), 6.81 (dd, *J* = 9.0, 1.3 Hz, 2H, ArH), 5.98 (ddt, *J* = 16.8, 10.0, 6.7 Hz, 1H, C=C*H*), 5.64 (br s, 1H, O*H*), 5.15 – 5.08 (m, 2H, C=CH_2), 3.80 (s, 3H, O*Me*), 3.40 (d, *J* = 6.7 Hz, 2H, ArCH_2).

^{13}C NMR (101 MHz, $CDCl_3$) δ 165.0, 160.4, 151.3, 139.2, 138.3, 137.3, 132.9, 122.9, 122.0, 120.9, 116.3, 115.5, 113.0, 56.0, 40.3.

HRMS (ESI$^+$): calcd for $C_{17}H_{16}O_4Na^+$ $[M+Na]^+$ 307.09408; found 307.09429.

***tert*-Butyl 4-(4-hydroxybenzyl)-3-oxopiperazine-1-carboxylate (48)**

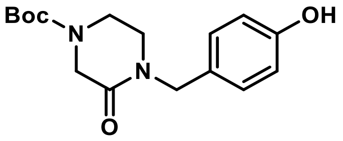

48, 54%

Following general procedure **GP1**, using *tert*-butyl 4-(4-iodobenzyl)-3-oxopiperazine-1-carboxylate (83.3 mg, 0.200 mmol, 1.0 equiv) as starting material, 33.0 mg (0.108 mmol, 54% yield) of *tert*-butyl 4-(4-hydroxybenzyl)-3-oxopiperazine-1-carboxylate (**48**) was obtained as a white solid after purification using DCM:EtOAc:MeOH (80:20:0.5) as eluent.

M.p.: 150.1 – 154.7 °C.

Rf (DCM:EtOAc (8:2)) 0.15.

^{1}H NMR (400 MHz, DMSO-*d_6*) δ 9.36 (s, 1H, O*H*), 7.05 (d, *J* = 8.5 Hz, 2H, Ar*H*), 6.71 (d, *J* = 8.5 Hz, 2H, Ar*H*), 4.40 (s, 2H, ArCH_2), 3.95 (s, 2H, NCH_2C(O)N), 3.49 (t, *J* = 5.5 Hz, 2H, BocNCH_2CH_2N), 3.19 (dd, *J* = 6.2, 4.8 Hz, 2H, BocNC$H_2$$CH_2$N), 1.40 (s, 9H, *tBu*).

^{13}C NMR (101 MHz, DMSO-*d_6*) δ 164.7 (br s), 156.7, 153.2, 129.2, 126.9, 115.3, 79.6, 48.3, 47.6-46.7 (br s), 45.8, 44.9, 27.9.

N.B.: some signals are broadened due to the existence of possible rotamers.

HRMS (ESI$^+$): calcd for $C_{16}H_{22}N_2O_4Na^+$ $[M+Na]^+$ 329.14718; found 329.14710.

tert-Butyl 3-((4-(4-hydroxyphenyl)-4-oxobutanoyl)oxy)azetidine-1-carboxylate (49)

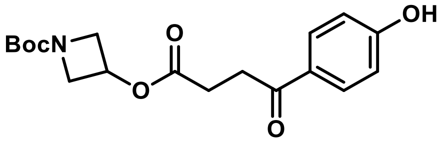

49, 51%

Following general procedure **GP1**, using *tert*-butyl 3-((4-(4-bromophenyl)-4-oxobutanoyl)oxy)azetidine-1-carboxylate (82.5 mg, 0.200 mmol, 1.0 equiv) as starting material, 35.6 mg (0.102 mmol, 51% yield) of *tert*-butyl 3-((4-(4-hydroxyphenyl)-4-oxobutanoyl)oxy)azetidine-1-carboxylate (**49**) was obtained as a white solid after purification using hexanes:EtOAc (7:3 to 6:4) as eluent.

M.p. 164.1 – 168.7 °C.

Rf (Hexanes:EtOAc (7:3)) 0.15.

^{1}H NMR (400 MHz, CDCl$_3$) δ 7.90 – 7.85 (m, 2H, ArH), 6.90 – 6.85 (m, 2H, ArH), 5.15 (tt, *J* = 6.8, 4.3 Hz, 1H, OC*H*), 4.24 (ddd, *J* = 10.2, 6.8, 1.1 Hz, 2H, NC*H$_2$*), 3.93 (ddd, *J* = 10.0, 4.3, 1.1 Hz, 2H, NC*H$_2$*), 3.26 (dd, *J* = 6.9, 6.1 Hz, 2H, ArC(O)C*H$_2$*), 2.76 (dd, *J* = 6.9, 6.0 Hz, 2H, OC(O)C*H$_2$*), 1.45 (s, 9H, *tBu*).

^{13}C NMR (101 MHz, CDCl$_3$) δ 196.5, 172.9, 161.0, 156.4, 130.8, 129.4, 115.6, 80.5, 63.7, 56.3, 33.0, 28.5, 28.3.

HRMS (ESI$^-$): calcd for $C_{18}H_{22}N_1O_6^-$ [M-H]$^-$ 348.15426; found 348.14511.

1-(4-Hydroxybenzoyl)pyrrolidin-2-one (50)

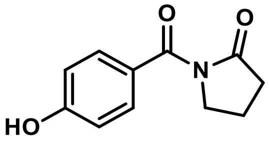

50, 51%

Following general procedure **GP1**, using 1-(4-bromobenzoyl)pyrrolidin-2-one (53.6 mg, 0.200 mmol, 1.0 equiv) as starting material, 20.9 mg (0.102 mmol, 51% yield) of 1-(4-hydroxybenzoyl)pyrrolidin-2-one (**50**) was obtained as a white solid after purification using DCM:EtOAc (99:1 to 98:2) as eluent.

R$_f$ (DCM:EtOAc (98:2) 0.15.

^{1}H NMR (400 MHz, CDCl$_3$) δ 7.59 – 7.54 (m, 2H, Ar*H*), 6.76 – 6.72 (m, 2H, Ar*H*), 3.95 (t, *J* = 7.0 Hz, 2H, NC*H$_2$*), 2.64 (dd, *J* = 8.3, 7.7 Hz, 2H, (O)NCC*H$_2$*), 2.15 (p, *J* = 7.5 Hz, 2H, NCH$_2$C*H$_2$*).

^{13}C NMR (101 MHz, CDCl$_3$) δ 175.4, 170.2, 159.8, 132.1, 126.1, 115.03, 47.2, 33.7, 17.9.
The values of the NMR spectra are in accordance with reported literature data.[102]
This is an intermediate in the synthesis of Aniracetam (stimulant and mental enhancer), as demonstrated in a patent using diazomethane as methylating agent.[103]

1-(4-Hydroxyphenyl)-4-(3-oxo-8-azabicyclo[3.2.1]octan-8-yl)butane-1,4-dione (51)

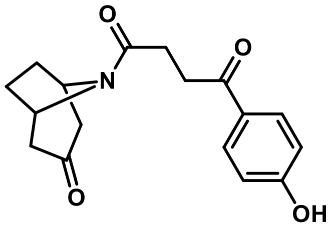

51, 61%

Following general procedure **GP1**, using 1-(4-bromophenyl)-4-(3-oxo-8-azabicyclo[3.2.1]octan-8-yl)butane-1,4-dione (72.8 mg, 0.200 mmol, 1.0 equiv) as starting material, 36.6 mg (0.121 mmol, 61% yield) of 1-(4-hydroxyphenyl)-4-(3-oxo-8-azabicyclo[3.2.1]octan-8-yl)butane-1,4-dione (**51**) was obtained as a white solid after purification using hexanes:EtOAc (1:2 to 1:5) as eluent.
M.p.: 144.8 – 147.6 °C.
Rf (Hexanes:EtOAc (1:3)) 0.15.
^{1}H NMR (400 MHz, CDCl$_3$) δ 7.67 – 7.63 (m, 2H, Ar*H*), 6.80 – 6.75 (m, 2H, Ar*H*), 4.95 (br s, 1H, NC*H*), 4.70 (br s, 1H, NC*H*), 3.44 (ddd, *J* = 18.0, 8.1, 5.3 Hz, 1H, ArC(O)C*H$_2$*), 3.21 (dt, *J* = 18.2, 5.7 Hz, 1H, ArC(O)C*H$_2$*), 3.00 (dd, *J* = 15.3, 4.5 Hz, 1H, C*H$_2$*C(O)C*H$_2$*), 2.96 – 2.80 (m, 2H, NCHC*H$_2$*C*H$_2$*), 2.76 (dd, *J* = 16.0, 4.8 Hz, 1H, C*H$_2$*C(O)C*H$_2$*), 2.51 (d, *J* = 16.0 Hz, 1H, C*H$_2$*C(O)C*H$_2$*), 2.43 (d, *J* = 16.0 Hz, 1H, C*H$_2$*C(O)C*H$_2$*), 2.26 (dt, *J* = 13.0, 7.0 Hz, 1H, NC(O)C*H$_2$*), 2.13 (dd, *J* = 12.8, 6.6 Hz, 1H, NC(O)C*H$_2$*), 1.88 (td, *J* = 12.7, 11.3, 4.2 Hz, 1H, NCHC*H$_2$*C*H$_2$*), 1.75 (td, *J* = 11.4, 9.7, 4.8 Hz, 1H, NCHC*H$_2$*C*H$_2$*).
^{13}C NMR (101 MHz, CDCl$_3$) δ 207.3, 196.9, 169.9, 161.7, 130.7, 128.9, 115.8, 54.3, 51.8, 49.6, 49.1, 33.2, 30.0, 28.0, 27.5.
HRMS (ESI$^-$): calcd for $C_{17}H_{18}N_1O_4^-$ [M–H]$^-$ 300.12413; found 300.12391.

4-Bromobenzyl 4-hydroxybenzoate (52)

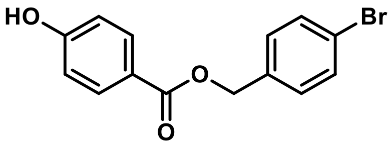

52, 78%

Following general procedure **GP1**, using 4-bromobenzyl 4-bromobenzoate (74 mg, 0.20 mmol, 1.0 equiv) as starting material, 48.3 mg (0.157 mmol, 78% yield, 97% purity with presence of 3% homocoupling) of 4-bromobenzyl 4-hydroxybenzoate (**52**) was obtained as a white solid after purification using DCM:EtOAc (1:0 to 95:5) as eluent, followed by a second column using pentane:EtOAc (10:1) as eluent.

M.p. 145.8 – 150.7 °C.

Rf (Hexane:EtOAc (8:2)) 0.35.

^{1}H NMR (400 MHz, CDCl$_3$) δ 8.00 – 7.95 (m, 2H, Ar*H*), 7.53 – 7.48 (m, 2H, Ar*H*), 7.33 – 7.29 (m, 2H, Ar*H*), 6.90 – 6.82 (m, 2H, Ar*H*), 5.51 (br s, 1H, O*H*), 5.28 (s, 2H, ArC*H$_2$*).

^{13}C NMR (101 MHz, CDCl$_3$) δ 166.3, 160.1, 135.3, 132.2, 131.9, 130.0, 122.6, 122.4, 115.4, 65.9.

HRMS (ESI$^-$): calcd for $C_{14}H_{10}O_3Br_1^-$ [M-H]$^-$ 304.98189; found 304.98222.

4-((2S,3R)-1-(4-Fluorophenyl)-3-((S)-3-(4-fluorophenyl)-3-hydroxypropyl)-4-oxoazetidin-2-yl)phenyl 4-hydroxybenzoate (53)

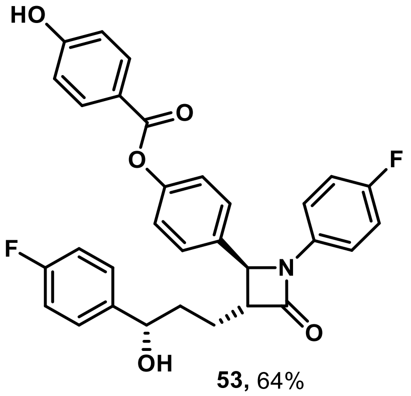

53, 64%

Following general procedure **GP1**, using 4-((2S,3R)-1-(4-fluorophenyl)-3-((S)-3-(4-fluorophenyl)-3-hydroxypropyl)-4-oxoazetidin-2-yl)phenyl 4-bromobenzoate (44.4 mg, 75.0 μmol, 1.0 equiv) as starting material, NiBr$_2$·diglyme (2.6 mg, 7.5 μmol, 0.10 equiv), bipy-pyr ligand **L50** (2.5 mg, 11 μmol, 0.15 equiv), NaI (16.8 mg, 113 μmol, 1.5 equiv), Zn (7.4 mg, 113 μmol, 1.5 equiv), and 0.4 mL of DMA (0.19 M). Following the work-up as depicted in **GP1**, 25.5 mg (48 μmol, 64% yield) of 4-((2S,3R)-1-(4-fluorophenyl)-3-((S)-3-(4-

fluorophenyl)-3-hydroxypropyl)-4-oxoazetidin-2-yl)phenyl 4-hydroxybenzoate (**53**) was obtained as a white solid after purification by preparative TLC using DCM:MeOH (97:3) as eluent.

M.p. 149.7 – 154.3 °C.

Rf (DCM:MeOH (97:3)) 0.30.

^{1}H NMR (400 MHz, $CDCl_3$) δ 8.13 – 8.05 (m, 2H, Ar*H*), 7.40 – 7.34 (m, 2H, Ar*H*), 7.34 – 7.27 (m, 2H, Ar*H*), 7.27 – 7.19 (m, 4H, Ar*H*), 7.06 – 6.99 (m, 2H, Ar*H*), 6.98 – 6.90 (m, 4H, Ar*H*), 4.77 – 4.71 (m, 1H, ArC*H*OH), 4.66 (d, *J* = 2.4 Hz, 1H, ArC*H*N), 3.15 – 3.08 (m, 1H NC(O)C*H*), 2.08 – 1.86 (m, 4H, CH_2CH_2).

^{13}C NMR (101 MHz, $CDCl_3$) δ 167.6, 164.8, 160.7, 157.1 (d, *J* = 192.7 Hz), 151.3, 140.0, 135.0, 132.8, 132.2, 127.6 (d, *J* = 8.2 Hz), 127.1, 122.9, 121.8, 118.6 (d, *J* = 7.9 Hz), 116.1 (d, *J* = 22.8 Hz), 115.6, 115.6 (d, *J* = 21.1 Hz), 73.3, 61.1, 60.5, 36.7, 25.2.

^{19}F NMR (282 MHz, $CDCl_3$) δ -114.7, -117.7.

N.B.: one carbon is not resolved due to C-F coupling.

HRMS (ESI$^-$): calcd for $C_{31}H_{24}F_2N_1O_5^-$ [M-H]$^-$ 528.16280; found 528.16303.

1-((3S,4R)-3-((Benzo[d][1,3]dioxol-5-yloxy)methyl)-4-(4-fluorophenyl)piperidin-1-yl)-4-(4-hydroxyphenyl)butane-1,4-dione (54)

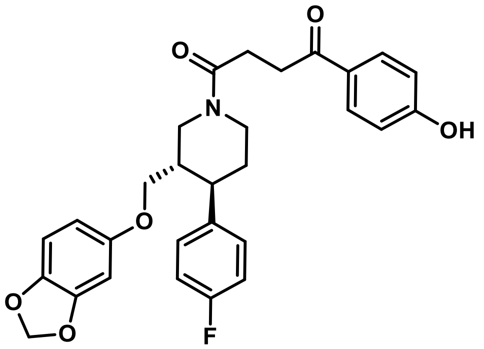

54, 41%

Following general procedure **GP1**, using 1-((3S,4R)-3-((benzo[d][1,3]dioxol-5-yloxy)methyl)-4-(4-fluorophenyl)piperidin-1-yl)-4-(4-bromophenyl)butane-1,4-dione (114 mg, 0.200 mmol, 1.0 equiv) as starting material, 41.5 mg (0.082 mmol, 41% yield) of 1-((3S,4R)-3-((benzo[d][1,3]dioxol-5-yloxy)methyl)-4-(4-fluorophenyl)piperidin-1-yl)-4-(4-hydroxyphenyl)butane-1,4-dione (**54**) was obtained as a white solid after purification using hexanes:EtOAc (2:1 to 2:3) as eluent.

M.p. 168.2 – 172.8 °C.

Rf (Hexanes:EtOAc (2:3)) 0.2.

HRMS (ESI$^-$): calcd for $C_{29}H_{27}F_1N_1O_6^-$ [M-H]$^-$ 504.18279; found 504.18302.

NMR: For this compound, two rotamers exist due to the presence of the amide bond. Efforts to analyse the rotamers were performed by the NMR department of our institute, using VT-NMR and 2D-experiments, ensuring good characterization and confirming a 1:0.9 ratio between the two rotamers (**54a** and **54b**). A summary can be found in the two tables S14-15 below.

LEF-LA-723

$CDCl_3$; 233 K; VT 223-328 K; 8 mg; av500as

Rotamer 1

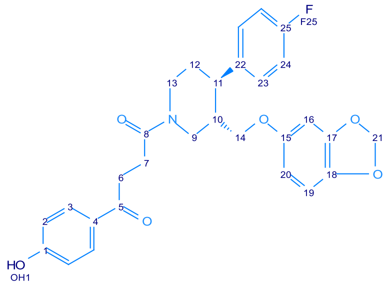

Rotamer Ratio ~1:0.9

Atom	δ (ppm)	J	HSQC	HMQC	COSY	ROESY/NOESY	H,F-HOESY
C1	161.74			2, 3			
OH1	9.65	br s				2	
C2	115.77		2				
H2	6.826	m (AA'XX')	2	1, 4	3	OH1, 3	
C3	130.61		3				
H3	7.656	m (AA'XX')	3	1, 5	2	2, 6	
C4	128.20			2			
C5	197.21			3, 6, 7			
C6	32.90		6	7			
H6	3.311	m (overlapped)	6	5, 7, 8	7	3	
C7	26.86		7	6			
H7	2.943	m (overlapped)	7	5, 6, 8	6	9a	
C8	171.29			6, 7			
C9	49.03		9a, 9b	14a, 14b			
H9a	4.416	dm 13.6(9b)	9		9b, 10, 13a	7, 9b, 10, 14a	
H9b	3.214	d 13.6(9a), d 11.7(10)	9		9a, 10	9a, 13b, 14b	
C10	42.39		10				
H10	2.188	m	10		9a, 9b, 11, 14a, 14b	9a, 12b, 14a, 14b, 23	
C11	43.77		11	23			
H11	2.792	m (overlapped)	11	22, 23	10, 12b	12a, 14b, 23	
C12	33.31		12a, 12b				
H12a	1.920	m (overlapped)	12		12b, 13b	11, 12b, 13a	
H12b	1.746	d 13.4(12a), t 13.0(11, 13b), d 4.6(13a)	12		11, 12a, 13a, 13b	10, 12a, 13a, 23	
C13	42.95		13a, 13b				
H13a	4.762	dm 13.0(13b)	13		9a, 12b, 13b	12a, 12b, 13b	
H13b	2.779	m (overlapped)	13		12a, 12b, 13a	9b, 13a	
C14	67.93		14a, 14b				
H14a	3.616	m (overlapped)	14	9	10, 14b	9a, 10, 16, 20, 23	
H14b	3.477	d 9.4(14a), d 7.1(10)	14	9	10, 14a	9b, 10, 11, 16, 20, 23	
C15	153.62			16, 19, 20			
C16	97.69		16	19, 20			
H16	6.402	d 2.5(20)	16	15, 17, 18, 20	20	14a, 14b	
C17	148.05			16, 19, 21			
C18	141.52			16, 19, 20, 21			
C19	107.89		19				
H19	6.664	d 8.5(20)	19	15, 16, 17, 18	20		
C20	104.68		20	16			
H20	6.149	d 8.5(19), d 2.5(16)	20	15, 16, 18	16, 19	14a, 14b	
C21	101.28		21				
H21	5.925	s	21	17, 18			
C22	138.16	d 3.0(F25)		11, 24			
C23	128.77	d 8.6(F25)	23	11			
H23	7.161	dm (AA'XX'Y; 5.3(F25))	23	11, 24, 25	24	10, 11, 12b, 14a, 14b	
C24	115.77	d 20.8(F25)	24	23			
H24	7.011	dm (AA'XX'Y; 8.5(F25))	24	22, 25	23		F25
C25	161.52	d 245.0(F25)		23, 24			
F25	-115.62	s					24

Table S14. NMR data of rotamer **54a**.

LEF-LA-723

$CDCl_3$; 233 K; VT 223-328 K; 8 mg; av500as

Rotamer 2

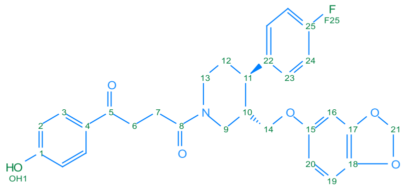

Atom	δ (ppm)	J	HSQC	HMQC	COSY	ROESY/NOESY	H,F-HOESY
C1	161.74			2, 3			
OH1	9.65	br s				2	
C2	115.70		2				
H2	6.802	m (AA'XX')	2	1, 4	3	OH1, 3	
C3	130.56		3				
H3	7.597	m (AA'XX')	3	1, 5	2	2, 6	
C4	128.13			2			
C5	197.23			3, 6, 7			
C6	32.97		6	7			
H6	3.289	m (overlapped)	6	5, 7, 8	7	3	
C7	26.95		7	6			
H7	2.911	m (overlapped)	7	5, 6, 8	6	13a, 13b	
C8	171.24			6, 7			
C9	45.54		9a, 9b	14b			
H9a	4.890	dm 13.3(9b)	9		9b, 10, 13a	9b, 10, 14a	
H9b	2.833	d 13.3(9a), d 11.7(10)	9		9a, 10	9a, 13b, 14b	
C10	41.83		10	14b			
H10	2.025	m (overlapped)	10		9a, 9b, 11, 14a, 14b	9a, 14a, 14b, 23	
C11	43.29		11	23			
H11	2.927	m (overlapped)	11	22, 23	10, 12b	12a, 14b, 23	
C12	33.93		12a, 12b				
H12a	1.997	m (overlapped)	12		12b, 13b	11, 12b, 13a, 13b, 23	
H12b	1.837	q 13.5(11, 12a, 13b), d 4.7(13a)	12		11, 12a, 13a, 13b	12a, 13a, 23	
C13	46.28		13a, 13b				
H13a	4.232	dm 13.4(13b)	13		9a, 12b, 13b	7, 12a, 12b, 13b	
H13b	3.287	m (overlapped)	13		12a, 12b, 13a	7, 9b, 12a, 13a	
C14	67.64		14a, 14b				
H14a	3.621	m (overlapped)	14		10, 14b	9a, 10, 16, 20, 23	
H14b	3.433	d 9.4(14a), d 5.4(10)	14	9, 10	10, 14a	9b, 10, 11, 16, 20, 23	
C15	153.88			16, 19, 20			
C16	97.79		16	19, 20			
H16	6.401	d 2.5(20)	16	15, 17, 18, 20	20	14a, 14b	
C17	147.95			16, 19, 21			
C18	141.35			16, 19, 20, 21			
C19	107.80		19				
H19	6.638	d 8.5(20)	19	15, 16, 17, 18	20		
C20	104.64		20	16			
H20	6.127	d 8.5(19), d 2.5(16)	20	15, 16, 18	16, 19	14a, 14b	
C21	101.19		21				
H21	5.906	s	21	17, 18			
C22	138.18	d 3.0(F25)		11, 24			
C23	128.85	d 8.7(F25)	23	11			
H23	7.155	dm (AA'XX'Y; 5.3(F25))	23	11, 24, 25	24	10, 11, 12a, 12b, 14a, 14b	
C24	115.69	d 20.2(F25)	24	23			
H24	6.994	dm (AA'XX'Y; 8.5(F25))	24	22, 25	23		F25
C25	161.47	d 245.0(F25)		23, 24			
F25	-115.74	s					24

Table S15. NMR data of rotamer **54b**.

6.4. Current limitations of the catalytic synthesis of phenols using N_2O and aryl halides

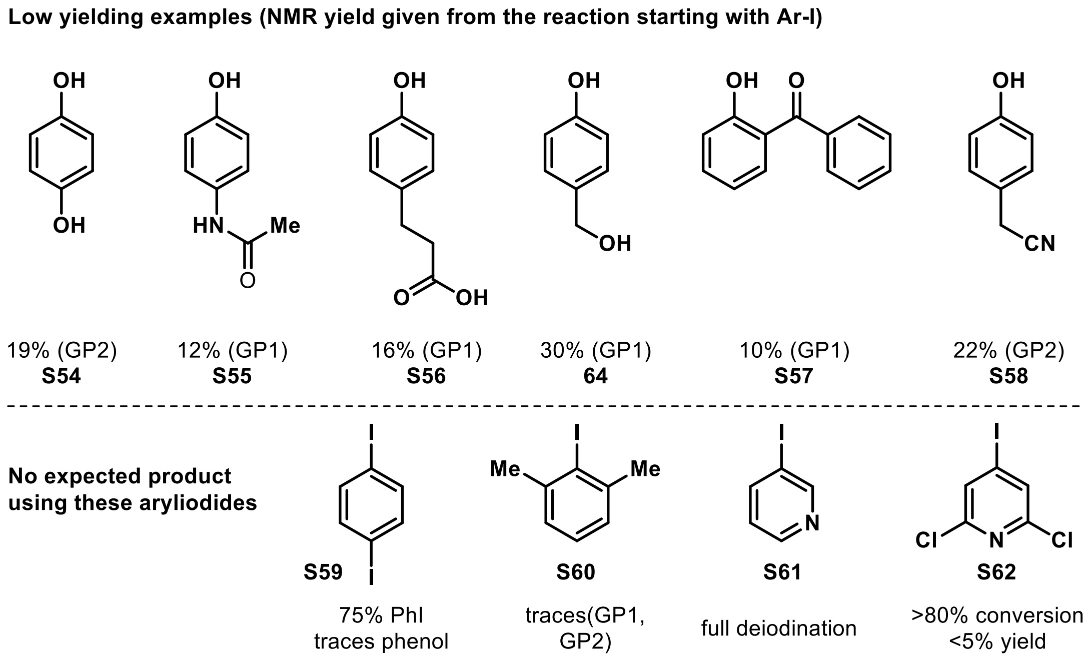

Fig. S38. Current limitations for (het)aryl iodides.

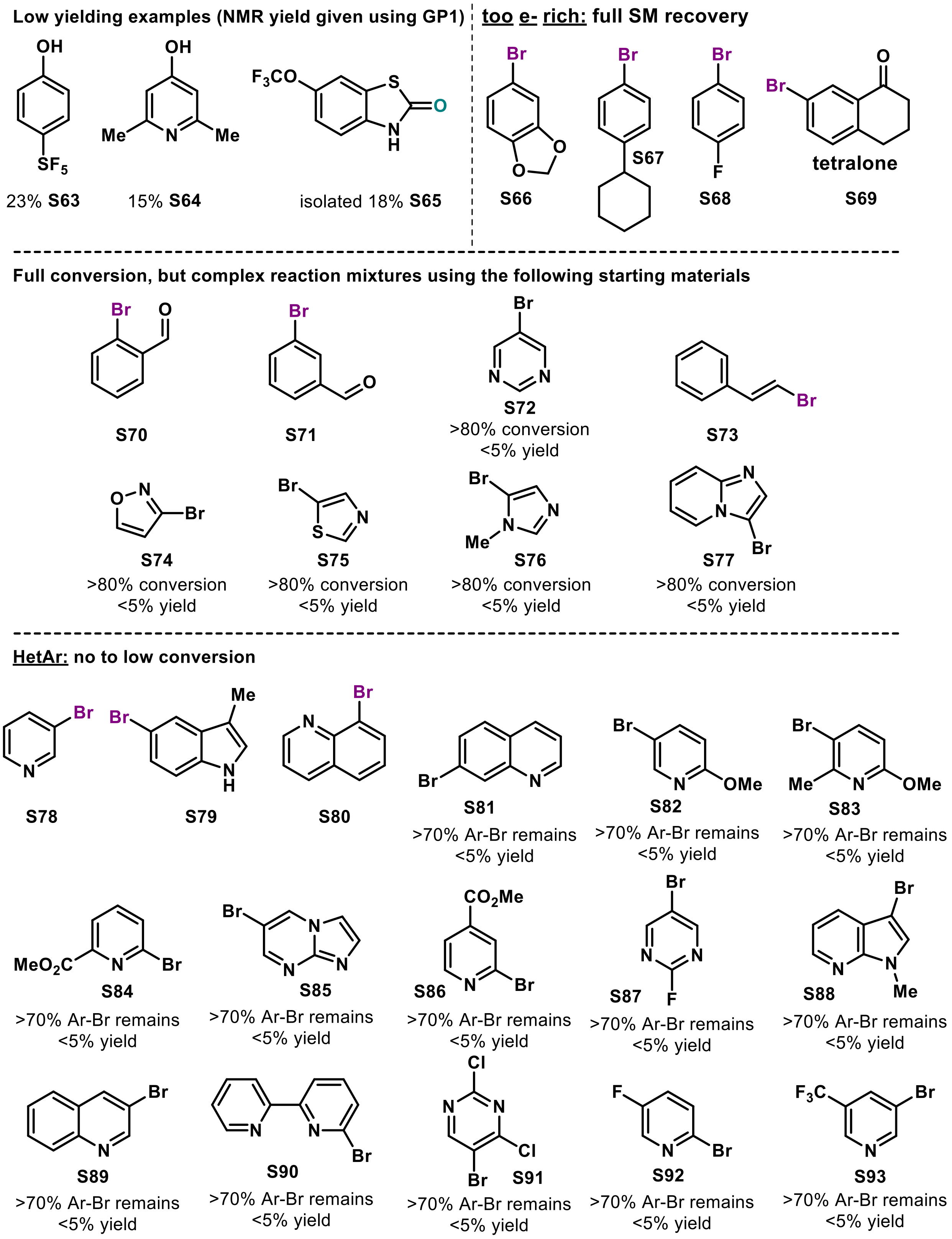

Fig. S39. Current limitations for (het)aryl and vinyl bromides.

7. Applications

7.1. Synthesis of metaxalone intermediate

3,5-Dimethylphenol (56)

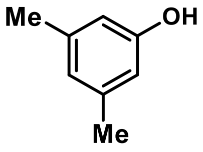

56, 63%

Following general procedure **GP2**, using 1-iodo-3,5-dimethylbenzene (29 μL) as starting material, 15.5 mg (0.127 mmol, 63% yield) of 3,5-dimethylphenol (**56**) was obtained as a light yellow solid with a smokey smell after purification using pentane:Et_2O (10:1 to 8:1) as eluent.
Rf (Pentane:Et_2O = 8:1) 0.3.
^{1}H NMR (400 MHz, $CDCl_3$) δ 6.59 (dt, *J* = 1.5, 0.7 Hz, 1H, Ar*H*), 6.47 (dt, *J* = 1.5, 0.7 Hz, 2H, Ar*H*), 4.58 (s, 1H, O*H*), 2.27 (d, *J* = 0.8 Hz, 6H, *Me*).
^{13}C NMR (101 MHz, $CDCl_3$) δ 155.5, 139.7, 122.7, 113.2, 21.4.
The values of the NMR spectra are in accordance with reported literature data.[80]

2-((3,5-Dimethylphenoxy)methyl)oxirane (58)

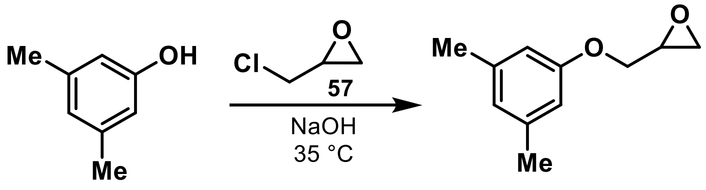

56 **58**, 96%

Following a reported procedure,[104] in a 5 mL flask at room temperature (23-25 °C), 35.0 mg of phenol **56** (0.287 mmol, 1.0 equiv) was suspended in 0.5 mL of water. Then aqueous sodium hydroxide [made from 11.5 mg NaOH (0.287 mmol, 1 equiv) in 0.5 mL of water] was added and the reaction system became clear. After stirring for 30 minutes, 67 μL of epichlorohydrin (**57**, 0.860 mmol, 3 equiv) was added and the reaction was stirred at 35 °C overnight (16 hours total). The reaction mixture was cooled to room temperature and transferred to a separatory funnel, diluted with water (5 mL) and extracted with DCM (3 x 10 mL). The organic layers were combined, dried over $MgSO_4$, filtered and concentrated. The crude NMR indicated a 2:1 ratio of the epoxide and the chloro-alcohol intermediate. Therefore, a new aqueous solution of sodium hydroxide [made from 11.5 mg NaOH (0.287 mmol, 1 equiv) in 0.5 mL of water] was

added. The reaction system was stirred for 18 additional hours at room temperature (25 °C). The reaction mixture was transferred to a separatory funnel, diluted with water (5 mL) and extracted with DCM (3 x 10 mL). The organic layers were combined, dried over $MgSO_4$, filtered and concentrated to give a very clean crude product. For characterization purpose, a fast column chromatography over silica gel using 10:1 to 8:1 pentane:Et_2O as eluent gradient afforded the pure epoxide product **58** in 96% (49.1 mg, 0.275 mmol).

Rf (Pentane:Et_2O = 8:2) 0.40.

^{1}H NMR (400 MHz, $CDCl_3$) δ 6.62 (m, 1H, Ar*H*), 6.56 (m, 2H, Ar*H*), 4.17 (dd, *J* = 11.0, 3.3 Hz, 1H, ArOC*H*$_2$), 3.95 (dd, *J* = 11.0, 5.5 Hz, 1H, ArOC*H*$_2$), 3.34 (dddd, *J* = 5.8, 4.1, 3.3, 2.6 Hz, 1H ArOCH$_2$C*H*), 2.90 (dd, *J* = 5.0, 4.1 Hz, 1H, ArOCH$_2$CHC*H*$_2$), 2.75 (dd, *J* = 5.0, 2.7 Hz, 1H, ArOCH$_2$CHC*H*$_2$), 2.29 (m, 6H, *Me*).

^{13}C NMR (101 MHz, $CDCl_3$) δ 158.7, 139.4, 123.1, 112.5, 68.7, 50.3, 44.9, 21.6.

The values of the NMR spectra are in accordance with reported literature data.[42]

5-((3,5-Dimethylphenoxy)methyl)oxazolidin-2-one (*not performed*)

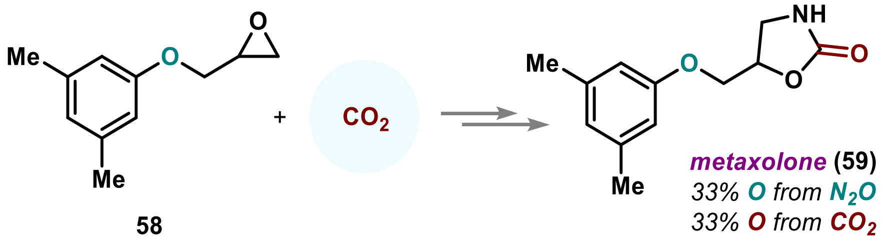

As reported,[42] this 2-step procedure involves the ring-opening of epoxide **58** by sodium azide followed by Staudinger ligation/carbon dioxide activation to build the final oxazolidin-2-one **59**.

7.2. Synthesis of phenol precursors towards bazedoxifene

1-(4-Hydroxyphenyl)propan-1-one (**63**)

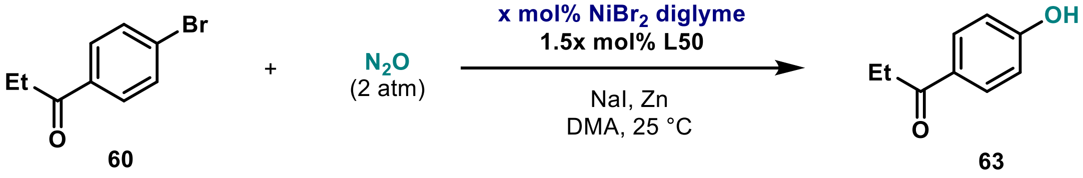

Scale-up

at 10 mol% **[Ni]**, isolated yields given
0.2 mmol, 20 h, 2 bar, **87%**
0.4 mmol, 20 h, 2 bar, **85%**
1.0 mmol, 36 h, 2.5 bar, **78%**

Catalyst loading

**[Ni]**, NMR yields given
10 mol%, 87%
2.5 mol%, 51%
1 mol%, 42%

As reported in the scope section, following general procedure **GP1**, using 1-(4-iodophenyl)propan-1-one (42.6 mg, 0.200 mmol, 1.0 equiv) as starting material, 26.1 mg (0.174 mmol, 87% yield) of 1-(4-hydroxyphenyl)propan-1-one (**63**) was obtained as a white solid after purification using DCM:EtOAc (95:5 to 90:10) as eluent.

^{1}H NMR (400 MHz, $CDCl_3$) δ 7.94 – 7.90 (m, 2H, Ar*H*), 6.91 – 6.86 (m, 2H, Ar*H*), 5.54 (br s, 1H, O*H*), 2.96 (q, *J* = 7.3 Hz, 2H, CH_2), 1.22 (t, *J* = 7.3 Hz, 3H, *Me*).

^{13}C NMR (101 MHz, $CDCl_3$) δ 199.8, 159.9, 130.7, 130.4, 115.4, 31.6, 8.6.

The values of the NMR spectra are in accordance with reported literature data.[105]

Scale-up reactions:

A scale up at 0.4 mmol following accordingly adapted **GP1** furnished the product in 85% yield. Finally, still in the same small Schlenk (12 mL total volume), a scale up to 1 mmol was performed following accordingly adapted **GP1**, using 10 mol% $NiBr_2$·diglyme, 15 mol% **L50**, 1.5 equivalent of zinc, 1.5 equivalent of NaI, 1-(4-iodophenyl)propan-1-one (0.21 g, *1.0 mmol*, 1.0 equiv) as starting material, in *DMA (2.0 mL, 0.5 M*) and stirred for *36 h* under *2.5 bar* of N_2O, afforded 117 mg of product **63** (0.779 mmol, 78%).

Lower catalyst loading reactions:

Following general procedure **GP1**, using *2.5 mol%* $NiBr_2$·diglyme and *3.75 mol%* **L50**, 1-(4-iodophenyl)propan-1-one (42.6 mg, 0.200 mmol, 1.0 equiv) as starting material, 51% NMR yield was obtained (determined by ^{1}H NMR using 1,3,5-trimethoxybenzene as internal standard).

Following general procedure **GP1**, using *1 mol%* $NiBr_2$·diglyme and *1.5 mol%* **L50**, 1-(4-iodophenyl)propan-1-one (42.6 mg, 0.200 mmol, 1.0 equiv) as starting material, 42% NMR yield was obtained (determined by ^{1}H NMR using 1,3,5-trimethoxybenzene as internal standard).

Phenol (**14**)

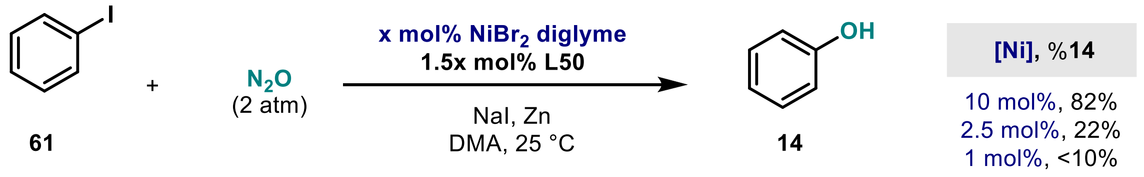

As reported in the scope section, following general procedure **GP1**, using phenyl iodide (22 μL, 0.20 mmol, 1.0 equiv) as starting material, 89% NMR yield of the expected product was observed using CH_2Br_2 as internal standard. Purification was performed using pentane:Et_2O

(10:1 to 8:1) as eluent, affording 15.5 mg (0.165 mmol, 82% yield) of phenol (**14**) as a white solid.

Rf (Pentane:Et_2O = 8:2) 0.3.

^{1}H NMR (400 MHz, $CDCl_3$) δ 7.28 – 7.22 (m, 2H, Ar*H*), 6.94 (m, 1H, Ar*H*), 6.87 – 6.81 (m, 2H, Ar*H*), 4.73 (br s, 1H, O*H*).

^{13}C NMR (101 MHz, $CDCl_3$) δ 155.5, 129.8, 121.0, 115.4.

The values of the NMR spectra are in accordance with reported literature data.[80]

<u>Lower catalyst loading reactions:</u>

Following general procedure **GP1**, using *2.5 mol%* $NiBr_2$·diglyme and *3.75 mol%* **L50**, phenyl iodide (22 µL, 0.20 mmol, 1.0 equiv) as starting material, 22% NMR yield was obtained (determined by ^{1}H NMR using 1,3,5-trimethoxybenzene as internal standard).

Following general procedure **GP1**, using *1 mol%* $NiBr_2$·diglyme and *1.5 mol%* **L50**, phenyl iodide (22 µL, 0.20 mmol, 1.0 equiv) as starting material, only trace amount product could be detected (less than 10%).

4-(Hydroxymethyl)phenol (64)

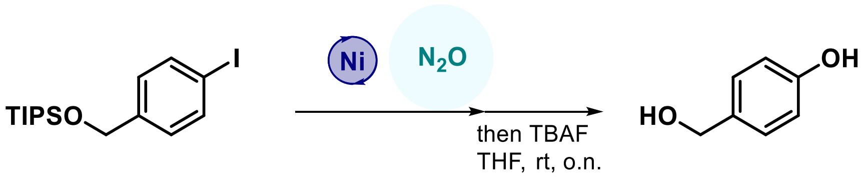

62, 53% **64**, 53%

Following general procedure **GP1** adapted for a reaction at 0.4 mmol scale, using ((4-iodobenzyl)oxy)triisopropylsilane (**62**) (156 mg, 0.400 mmol, 1.0 equiv) as starting material, the work-up as performed as for described in GP1, thus affording a crude yellow oil.

The crude mixture containing 4-(((triisopropylsilyl)oxy)methyl)phenol (**33**) was then dissolved in 0.8 mL of THF in a dry vial equipped with a Teflon coated stir bar, under argon. The solution was cooled to 0 °C and 1.2 mL of TBAF (1 M in THF, 1.2 mmol, 3 equiv) was added dropwise by syringe. The resulting mixture was allow to warm up to room temperature and stirred for 24 h. The reaction was quenched by diluting with Et_2O (2 mL) and adding 1 mL of NH_4Cl. Then the mixture was poured into a separatory funnel containing 10 mL of saturated aqueous $NaHCO_3$, and it was extracted 4 times with Et_2O (15 mL). The combined organic layers were then washed with brine, dried over $MgSO_4$, filtered and concentrated under reduce pressure. The crude material was then purified by column chromatography over silica gel using

hexanes:EtOAc (2:1 to 1:1) as eluent, affording 26.4 mg (0.213 mmol, 53% over 2 steps) of pure 4-(hydroxymethyl)phenol (**64**) as white crystalline solid.

Rf (Hexanes:EtOAc = 4:6) 0.4.

^{1}H NMR (400 MHz, DMSO-*d$_6$*) δ 9.21 (s, 1H, ArO*H*), 7.12 – 7.07 (m, 2H, Ar*H*), 6.72 – 6.67 (m, 2H, Ar*H*), 4.93 (t, *J* = 5.7 Hz, 1H, CH_2O*H*), 4.35 (d, *J* = 5.7 Hz, 2H, C*H_2*OH).

^{13}C NMR (101 MHz, DMSO-*d$_6$*) δ 156.2, 132.8, 128.0, 114.8, 62.8.

The values of the NMR spectra are in accordance with reported literature data.[101]

1-(4-Methoxyphenyl)propan-1-one (65)

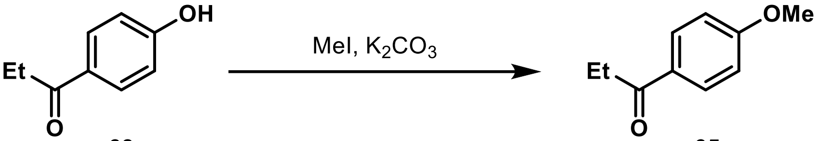

63 65

A reaction tube was charged with 1-(4-hydroxyphenyl)propan-1-one (15.0 mg, 0.1 mmol), K_2CO_3 (41.4 mg, 0.3 mmol), acetone (1.0 mL), and MeI (70.5 mg, 5.0 mmol) under air. The reaction mixture was refluxed for 5 h and cooled to room temperature. The reaction mixture was filtered and the filtrate was concentrated under reduced pressure to afford the crude product. The product was purified by preparative TLC using hexanes and EtOAc (10:1) as an eluent, affording 15.1 mg of the final product **65** (92% yield).

^{1}H NMR (300 MHz, $CDCl_3$) δ 8.00 – 7.88 (m, 2H, Ar*H*), 6.96 – 6.84 (m, 2H, Ar*H*), 3.85 (s, 3H, O*Me*), 2.94 (q, *J* = 7.3 Hz, 2H, C*H_2*CH_3), 1.20 (t, *J* = 7.3 Hz, 3H, CH_2C*H_3*).

^{13}C NMR (75 MHz, $CDCl_3$) δ 199. 6, 163.4, 130.3, 130.2, 113.8, 55.5, 31.5, 8.5.

The values of the NMR spectra are in accordance with reported literature data.[106]

Di-*tert*-butyl 1-(4-methoxyphenyl)hydrazine-1,2-dicarboxylate (66)

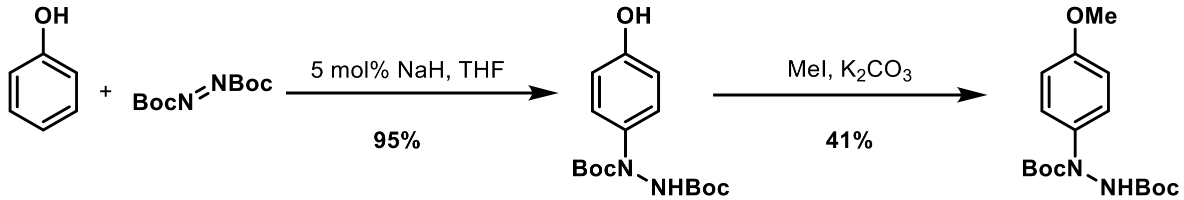

S94 66

Following a reported procedure,[43] a solution of 0.500 g of phenol (5.31 mmol) and 8.0 mg of NaH (0.27 mmol) in 10 mL of THF in a 50 mL flask was cooled with an ice bath (acetone and dry ice). A portion of azodicarboxylate ester was added (5.57 mmol) while stirring at room temperature and kept stirring for 3 hours. The solvent was evaporated and the reaction was

diluted with CH_2Cl_2. The organic layer was washed with $NaHCO_3$ and NaCl, and dried over Na_2SO_4. The product was purified by a silica gel column using hexanes and EtOAc (5:1) as an eluent, affording 1.6344 g of the expected product **S94** (95% yield).

A reaction tube was charged with di-*tert*-butyl 1-(4-hydroxyphenyl)hydrazine-1,2-dicarboxylate (**S94**, 32.4 mg, 0.10 mmol), K_2CO_3 (41.4 mg, 0.30 mmol), acetone (1.0 mL), and MeI (70.5 mg, 5.0 mmol) under air. The reaction mixture was refluxed for 5 h and cooled to room temperature. The reaction mixture was filtered and the filtrate was concentrated under reduced pressure to afford the crude product. The product was purified by preparative TLC using hexanes and EtOAc (10:1) as an eluent, affording 13.9 mg of the final product **66** (41% yield).

^{1}H NMR (600 MHz, $CDCl_3$) δ 7.31 (br s, 2H, Ar*H*), 6.87 – 6.81 (m, 2H, Ar*H*), 6.78 (s, 1H, N*H*), 3.79 (s, 3H, O*Me*), 1.63 – 1.32 (m, 18H, *Boc*).

^{13}C NMR (151 MHz, $CDCl_3$) δ 157.6, 155.4, 154.0, 135.4, 126.0, 113.7, 82.0, 81.4, 55.4, 28.2, 28.2.

The values of the NMR spectra are in accordance with reported literature data.[107]

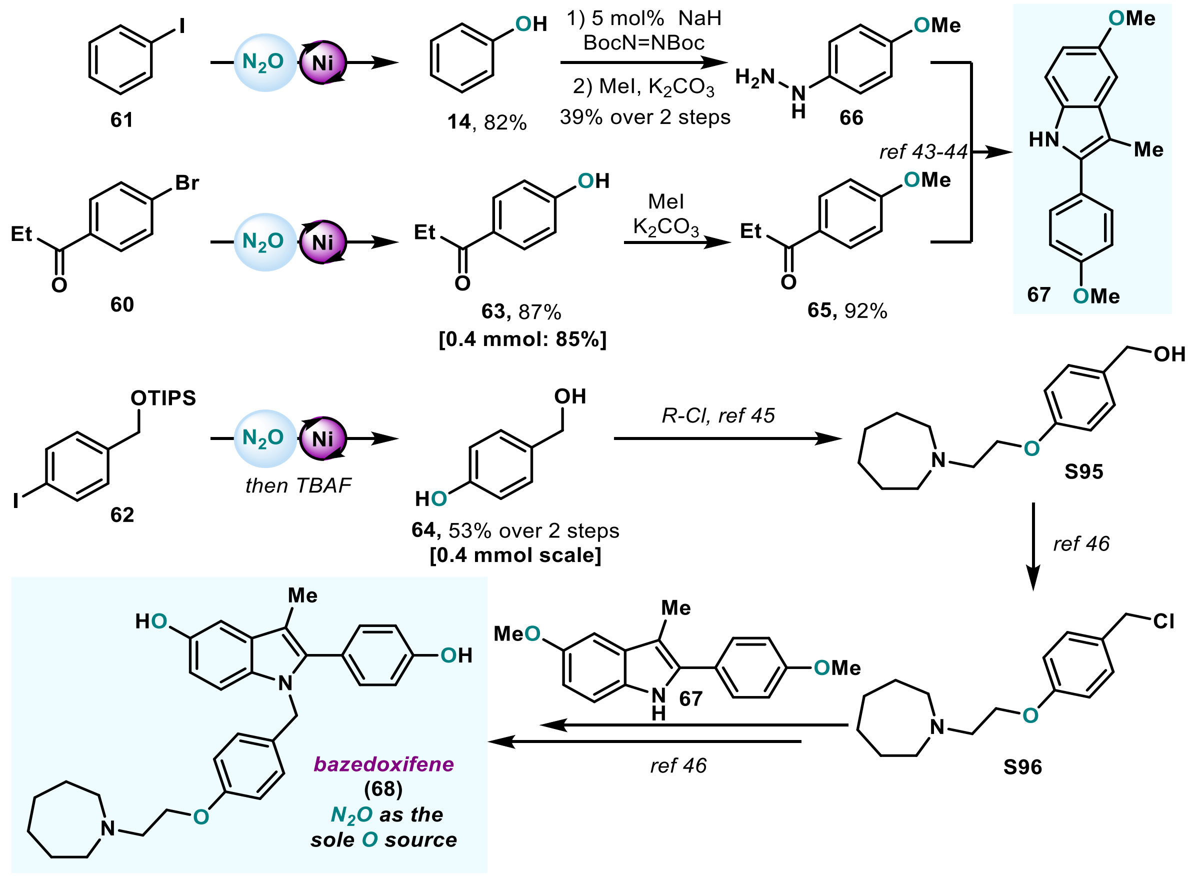

Fig. S40. Use of Ni/N_2O to access phenolic building blocks and reported procedures for the synthesis of bazedoxifene.

8. Labelling experiment

8.1. Synthesis of ^{18}O-dimethylformamide

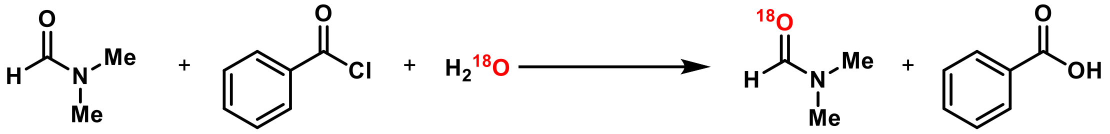

DMF   S97   50% ^{18}O labeled water   25%-^{18}O labeled DMF   S98

Following a slightly modified reported procedure,[108] a culture tube equipped with a magnetic stirrer was evacuated and backfilled with argon (three times). Subsequently, dimethylformamide (25.0 mmol, 1 equiv.) and benzoyl chloride (25.0 mmol, 1 equiv.) was added using a syringe. The mixture was cooled to 0 °C in an ice bath for about 10 min. 0.463 g (25.0 mmol, 1 equiv.) of ^{18}O-labelled water (*50% mole of ^{18}O isotope*) was then introduced slowly while stirring. In about 5 min after addition of isotope labelled water the contents were solidified due to the precipitation of benzoic acid. The reaction mixture was diluted with acetone, then Na_2CO_3 (2.0 g) was added and keeping stirring for 1 hour. Upon filtration, the organic layer was concentrated under reduced pressure (*water bath at 40 °C*). The residual liquid was distilled at reduced pressure to obtain around 1.5 mL of ^{18}O-dimethylformamide (*25% mole of ^{18}O isotope, which was determined by MS, Fig S41*).

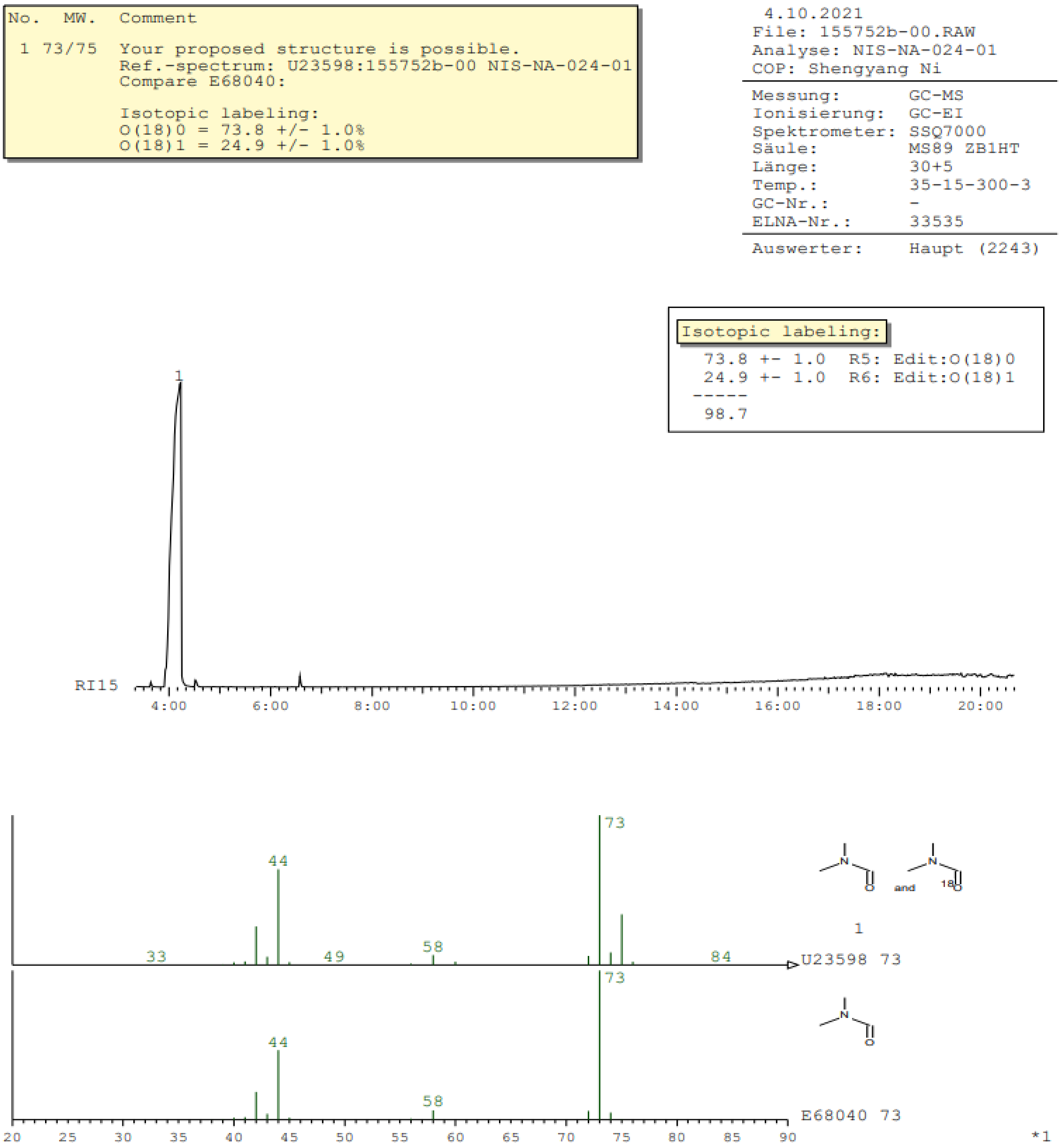

Fig. S41. GC-MS analysis of the 25% enriched ^{18}O-dimethylformamide.

8.2. Reaction using ^{18}O-dimethylformamide as solvent

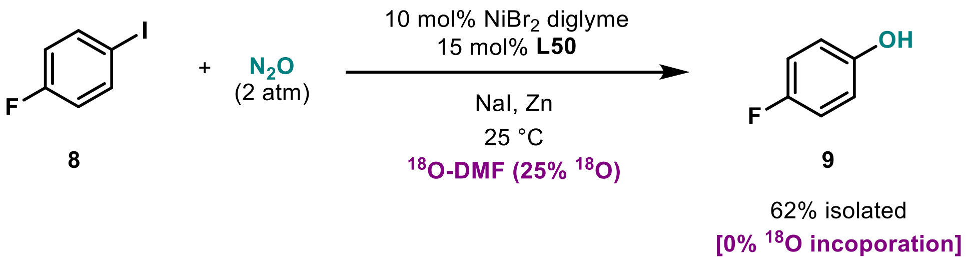

Following general procedure **GP1**, *using* ^{18}*O-dimethylformamid (25% mole of* ^{18}*O isotope) as the solvent* and using 4-fluoro-phenyl iodide (23 µL, 0.20 mmol, 1.0 equiv) as starting material.

Purification via column chromatography over silica gel was performed using pentane:EtOAc (10:1) as eluent, affording 13.9 mg (0.124 mmol, 62% yield) of 4-fluorophenol (**9**) as a pale yellow solid. ***According to the MS analysis (Fig. S42), no incorporation of*** ^{18}***O in 14 was obtained.***

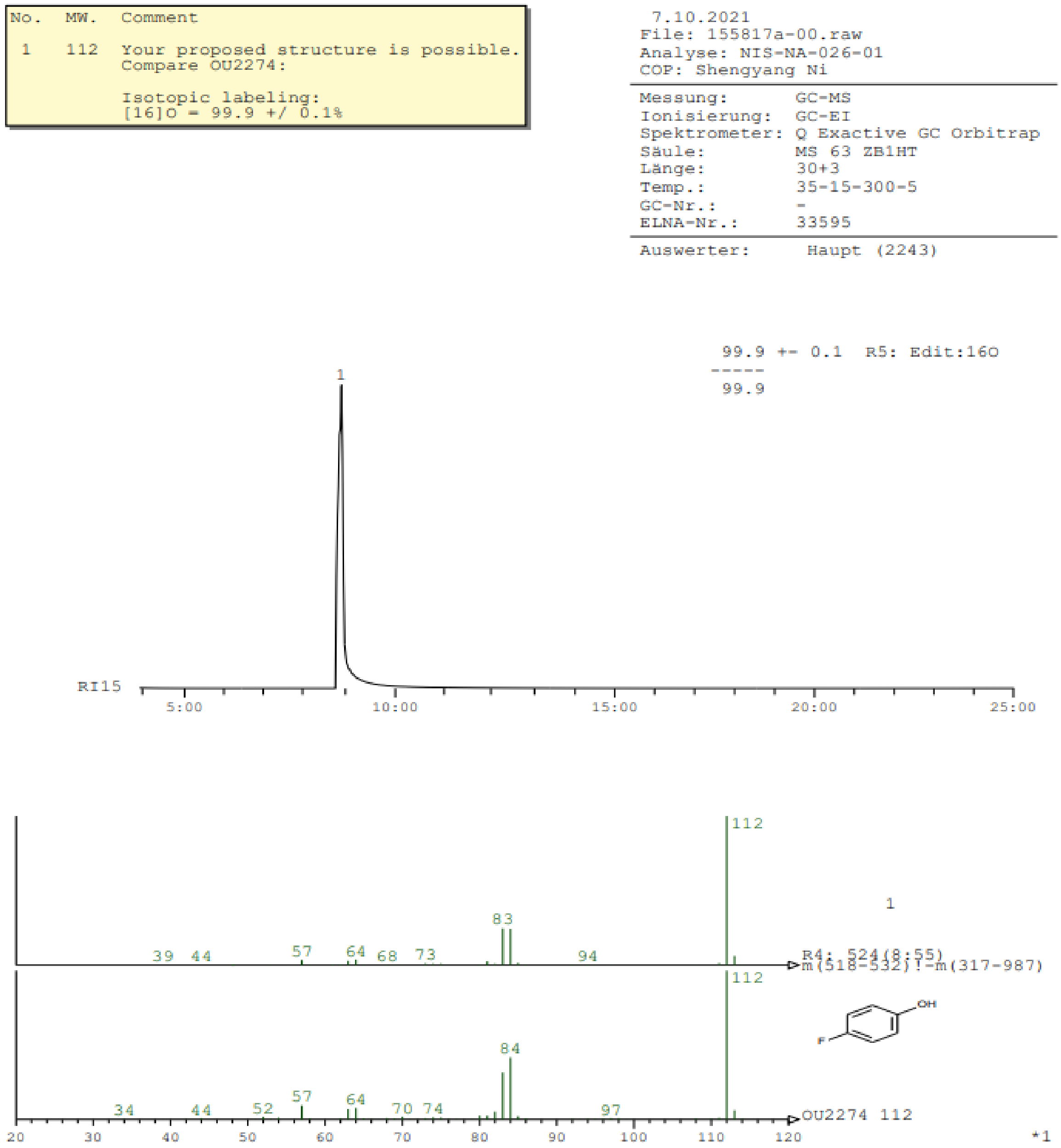

Fig. S42. GC-MS analysis of the isolated phenolic product using 25% enriched ^{18}O-dimethylformamide as solvent.

8.3. Reaction using in situ formed ^{18}O-N_2O

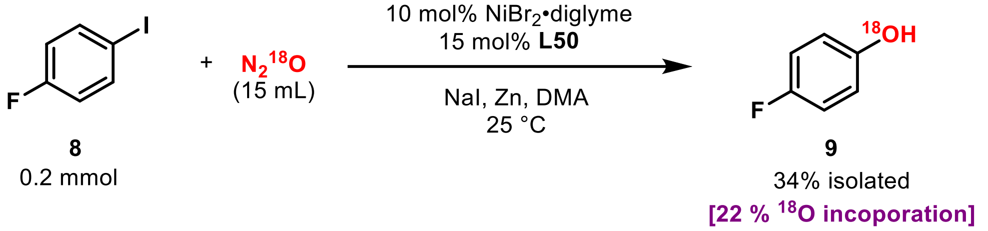

A reaction tube (3 mL) equipped with a Teflon-coated stir bar was used. $HONH_2 \cdot HCl$ (100 mg) was introduced into the reaction tube. After that, the tube was closed with a septum screw cap and a syringe (20 mL) with a needle pierced through the septum. Then, the reaction tube was evacuated and refilled with argon (3 times). After that, H_2O (0.5 mL) was added using a syringe while stirring. After 2 min, $NaNO_2$ (60.0 mg, 45% ^{18}O-labelled) dissolved in 0.6 mL H_2O was added dropwise using syringe. Around 15 mL $N_2{}^{18}O$ gas could be collected by the syringe, which was used directly for the next step.

An oven- and heatgun-dried reaction tube equipped with a Teflon-coated stir bar was used. The reaction tube was brought into an argon-filled glovebox, along with a heatgun-dried vial. Dried DMA was introduced into the vial. $NiBr_2 \cdot$diglyme (7.1 mg, 0.020 mmol, 10 mol%), bipy-pyr **L50** (6.7 mg, 0.030 mmol, 15 mol%), NaI (45 mg, 0.30 mmol, 1.5 equiv), and zinc (20 mg, 0.30 mmol, 1.5 equiv) were introduced into the reaction tube, and a septum screw-cap employed to close it. Once outside the glovebox, the reaction tube was evacuated and removed from the vacuum line. The septum screw cap of the reaction tube was then pierced with the syringe containing the *^{18}O-N_2O*, and 15 ml gas were introduced into the reaction tube. Then, the DMA (0.5 mL) was added using a syringe, *and* 1-fluoro-4-iodobenzene was subsequently added using a Hamilton syringe. After that, the reaction mixture was kept stirring at 1000 rpm, for 20 h, at RT.

After that time, the reaction was first diluted using 2 mL of Et_2O. The mixture was then quenched with HCl (1 M) and extracted 3 times with Et_2O. The combined organic layers were washed with brine, dried over $MgSO_4$, filtered and concentrated under reduced pressure. The crude was purified by preparative TLC affording 7.6 mg of the final product (34% yield). ***According to the MS analysis (Fig. S43), 22% of ^{18}O in 9 was obtained.***

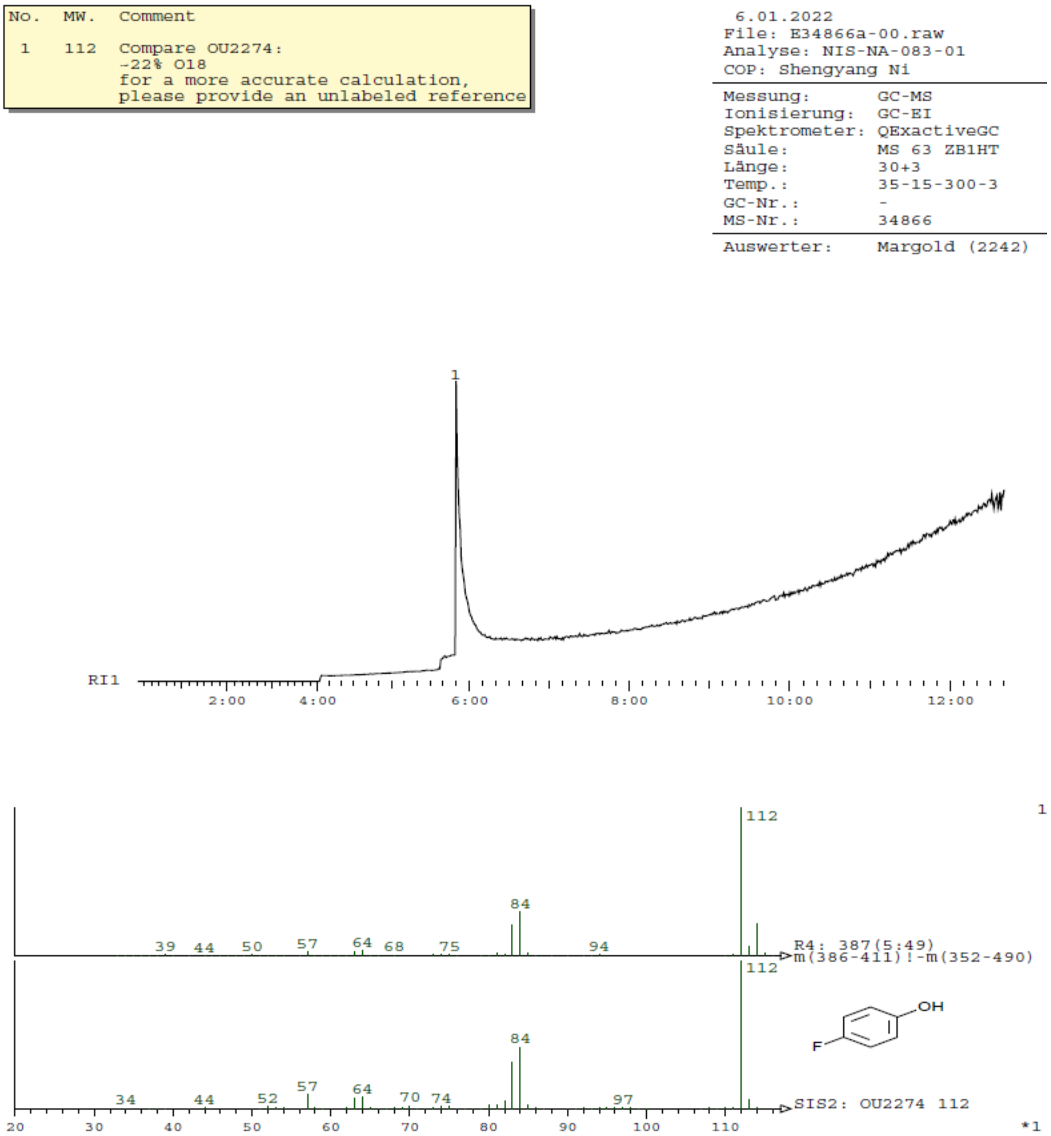

Fig. S43. GC-MS analysis of the isolated phenolic product using 23% enriched ^{18}O-N_2O.

Mechanism of N_2O formation

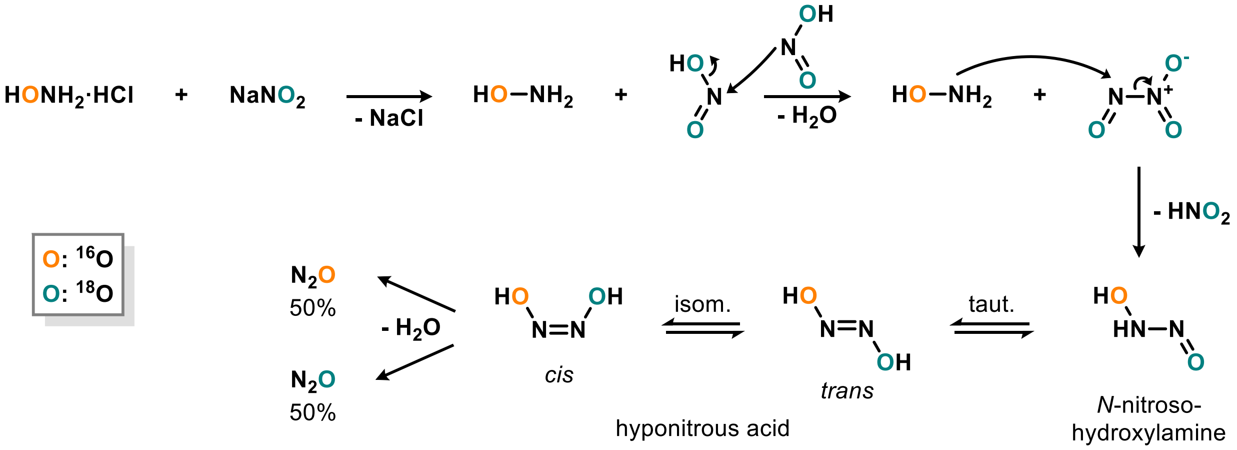

Figure S44: Mechanism of the formation of N_2O from hydroxylamine hydrochloride and labelled sodium nitrite.

The mechanism of the formation of N_2O from hydroxylamine hydrochloride and sodium nitrite is reported in the literature as depicted in Figure S.[109] Initially, nitrous acid is formed from the salt metathesis of the hydrochloride and the sodium nitrite. The anhydric form of nitrous acid N_2O_3 acts then as the nitrosation agent reacting with hydroxylamine. The thus formed *N*-nitrosohydroxylamine tautomerizes to hyponitrous acid, of which its *cis*-isomer decomposes to N_2O upon loss of a water molecule. Importantly, in the hypothetical reaction of unlabelled hydroxylamine hydrochloride with 100% ^{18}O-labelled sodium nitrite, N_2O would be formed from a symmetric intermediate *cis*-hyponitrous acid, containing both a labelled ^{18}O-atom and a non-labelled ^{16}O-atom. Since the dehydration could occur at both sites, the resulting N_2O gas would be ^{18}O-labelled only at 50 %. In the present experiment the employed sodium nitrite was 45% ^{18}O-labelled, therefore the thus formed N_2O gas was expected to be 23% ^{18}O-labelled.

9. Headspace analysis

General procedure of the reactions for the GC measurement of the headspace

To support the hypothesis that N_2O is the source of oxygen in the phenol products, we performed analysis of the headspace after the reaction of several substrates. The detection of significant amounts of N_2 would support the N_2O activation. Such analyses are easily achieved by gas chromatography using a thermal conductivity detector (GC-TCD), typically used for gas analysis. To facilitate the GC measurement, the reaction was conducted in a vial equipped with a screw septum cap, and the pressure reduced to 1.5 bar. This vial was found to be suitable, and provided only slightly diminished yield for the optimized substrates (*see section 5.2*) using 1.75 bar. The use of 1.5 bar is expected to provide good but lower yields compared to the reaction in pressure Schlenks.

Following the procedure detailed below, the obtained results are summarized in table S16.

Entry		Yield phenol[a]		Ar, O_2[c]	N_2O	N_2
		In vial	In Schlenk[b]			
**1**	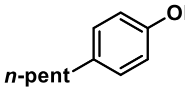 **25**	46%	79%	1%	82%	**17%**
**2**	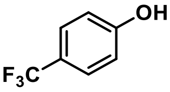 **7**	61%	75%	1%	60%	**40%**
**3**	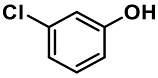 **18**	60%	74%	1%	62%	**38%**
**4**	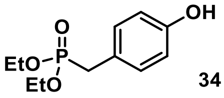 **34**	29%	50%	1%	63%	**36%**
**5**	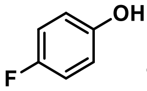 **9**	68%	80%	2%	55%	**43%**
**6**	**Control N_2O + DMA + Zn**	-	-	5%	90%	**4%**
**7**	**Control N_2O + DMA**	-	-	3%	96	**1%**
**8**	**Control N_2O**	-	-	<1%	98%	**2%**
**9**	**Control air**	-	-	21%	0%	**79%**

Table S16. GC analysis of the headspace of the N_2O activation reaction for various substrates. [a] NMR yield. [b] The phenol yield for the reaction in the optimized conditions using a Schlenk flask is given for comparison. [c] Since their signals overlapped in the GC measurement, their percentages are given as a sum. *N.B.: The measurements are qualitative and not quantitative.*

Procedure

Following **GP1** but using a normal vial sealed with screw cap having a rubber-septum, the N_2O

atmosphere was set to 1.5 bar. The mixture was stirred at room temperature for 20 h, and then the headspace analyzed via GC-TCD. Water (2 mL), Et_2O (2 mL), and aqueous HCl (2 M, 2 mL) were added thereafter to quench the reaction, stirring for 10 min. The mixture was extracted with Et_2O (3 × 10 mL), the combined organic layers washed with brine (10 mL), dried over Na_2SO_4, filtrated, and concentrated under reduced pressure (34 °C, 650 mbar). The resulting phenol was quantified via ^1H NMR using 4-fluoronitrobenzene or dibromomethane as an internal standard.

Discussion

In all measurements of the scope reactions, N_2 gas could be detected in the headspace after reaction, while argon and oxygen were only found as less than 2 vol% combined. Several control experiments were undertaken. First, Zn+DMA+N_2O gave only minor production of N_2, thus confirming the good compatibility of Zn and N_2O. Only DMA+N_2O and control experiment using a vial filled with N_2O confirmed the excellent quality of our bottle by discarding the presence of large amount of N_2, as well as the absence of oxygen. Another control experiment using a vial filled with atmospheric air highlights the abscence of possible leaks during the reaction, that would have obviously been revealed by high amount of oxygen in the headspaces. The lower yields achieved are consistent with the reduced N_2O pressure.

All the chromatograms obtained after GC-TCD measurement are reported below (Fig. S45 to S53).

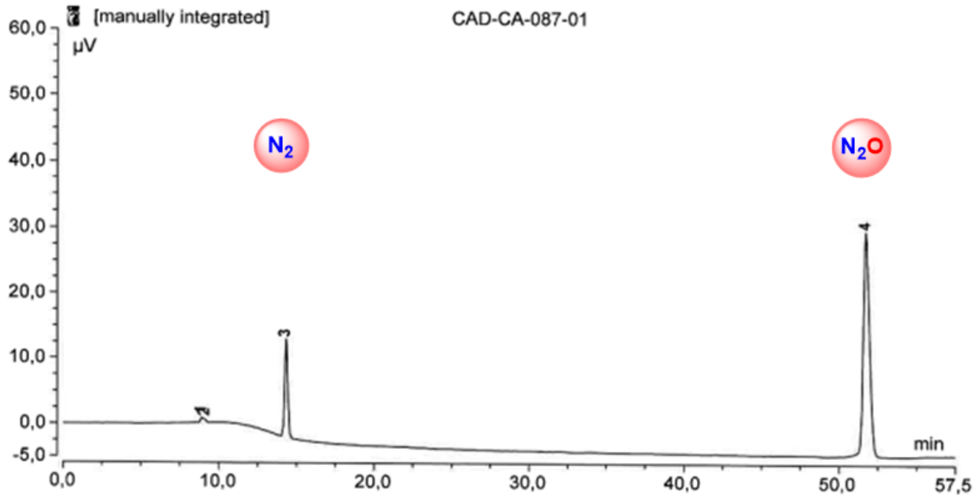

Sample:	CAD-CA-087-01	Instrument:	GC_311
Sequenz:	8140 CAD-CA VD	Measured:	23.03.21 12:28
Sequenz date:	23.03.21	Processing M.:	CAD-CA
		Report-File:	087-01

Zuordnung siehe PAY-PA-642-01 20/7477 und LEF-LA-442-01 20/7680

No.	Ret.Time min	area-% %	Peak Name
1	8,92	0,79	Argon (keine Basislinientrennung)
2	9,12	0,38	O2 (keine Basislinientrennung)
3	14,33	17,38	N2
4	51,72	81,45	N2O

Fig. S45. GC-TCD analysis of the headspace during 4-pentylphenol synthesis (entry 1, Table S16).

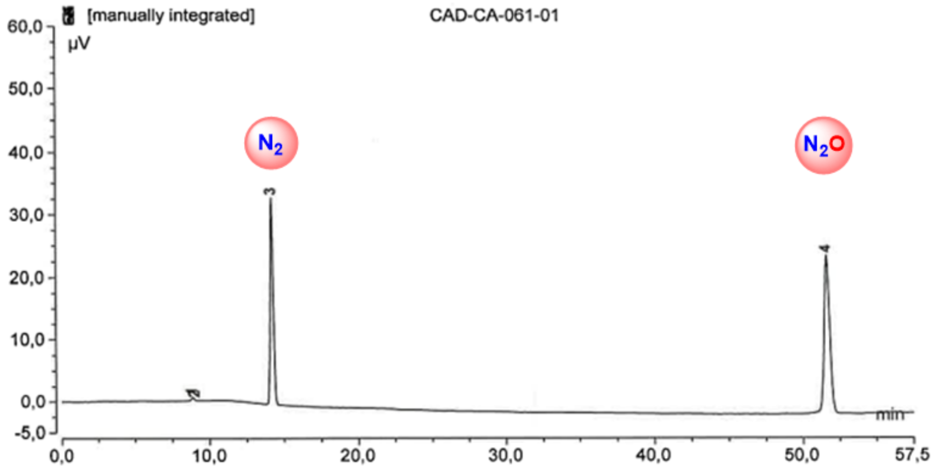

Sample:	CAD-CA-061-01	Instrument:	GC_311
Sequenz:	8101 CAD-CA PH	Measured:	09.03.21 14:23
Sequenz date:	09.03.21	Processing M.:	CAD-CA
		Report-File:	056-01

Zuordnung siehe PAY-PA-642-01 20/7477 und LEF-LA-442-01 20/7680
Retentionszeitverschiebung aufgund von Gerätewechsel

No.	Ret.Time min	area-% %	Peak Name
1	8,88	0,52	Argon
2	9,04	0,05	O2
3	14,18	39,78	N2
4	51,61	59,65	N2O

Fig. S46. GC-TCD analysis of the headspace during 4-trifluoromethylphenol synthesis (entry 2, Table S16).

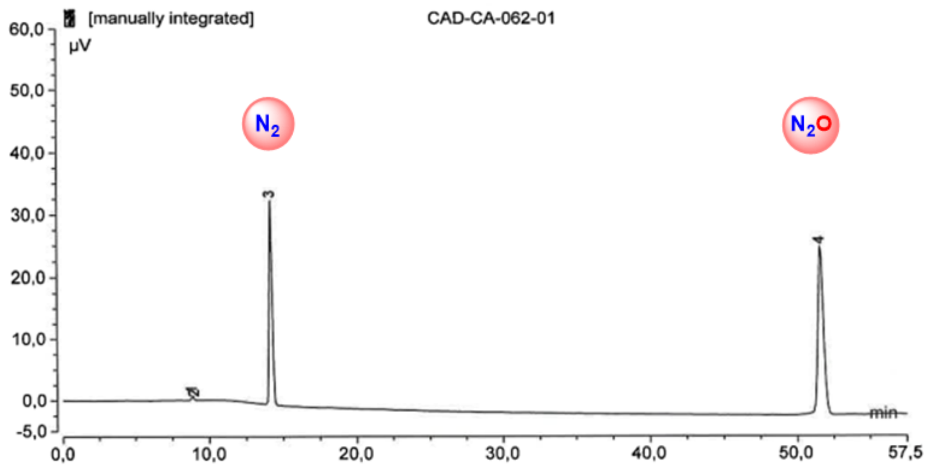

Sample:	CAD-CA-062-01	Instrument:	GC_311
Sequenz:	8102 CAD-CA PH	Measured:	10.03.21 10:25
Sequenz date:	09.03.21	Processing M.:	CAD-CA
		Report-File:	056-01

Zuordnung siehe PAY-PA-642-01 20/7477 und LEF-LA-442-01 20/7680
Retentionszeitverschiebung aufgund von Gerätewechsel

No.	Ret.Time min	area-% %	Peak Name
1	8,87	0,62	Argon
2	9,03	0,03	O2
3	14,17	37,78	N2
4	51,60	61,56	N2O

Fig. S47. GC-TCD analysis of the headspace during 3-chlorophenol synthesis (entry 3, Table S16).

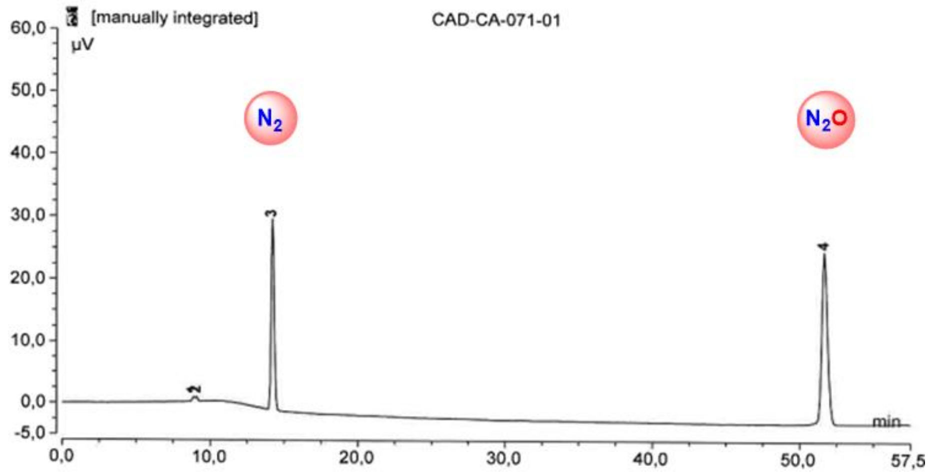

Sample:	CAD-CA-071-01	Instrument:	GC_311
Sequenz:	8120 CAD-CA VD	Measured:	16.03.21 13:44
Sequenz date:	16.03.21	Processing M.:	CAD-CA
		Report-File:	071-01

Zuordnung siehe PAY-PA-642-01 20/7477 und LEF-LA-442-01 20/7680
Retentionszeitverschiebung aufgund von Gerätewechsel

No.	Ret.Time min	area-% %	Peak Name
1	8,88	0,61	Argon
2	9,07	0,65	O2
3	14,21	35,53	N2
4	51,65	63,21	N2O

Fig. S48. GC-TCD analysis of the headspace during diethyl (4-hydroxybenzyl)phosphonate synthesis (entry 4, Table S16).

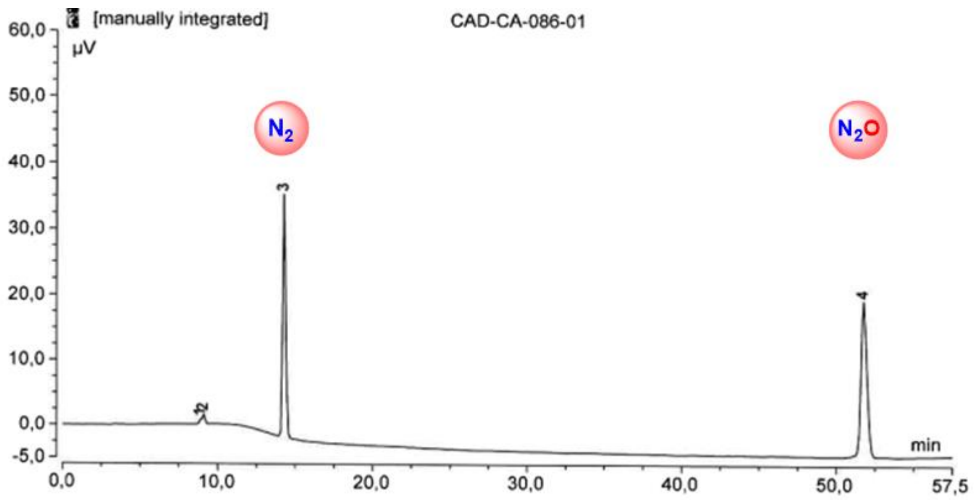

Sample:	**CAD-CA-086-01**	Instrument:	**GC_311**
Sequenz:	**8139 CAD-CA VD**	Measured:	**23.03.21 09:54**
Sequenz date:	**23.03.21**	Processing M.:	**CAD-CA**
		Report-File:	**086-01**

Zuordnung siehe PAY-PA-642-01 20/7477 und LEF-LA-442-01 20/7680

No.	Ret.Time min	area-% %	Peak Name
1	8,92	0,59	Argon (keine Basislinientrennung)
2	9,11	1,38	O2 (keine Basislinientrennung)
3	14,26	43,28	N2
4	51,75	54,74	N2O

Fig. S49. GC-TCD analysis of the headspace during 4-fluorophenol synthesis (entry 5, Table S16).

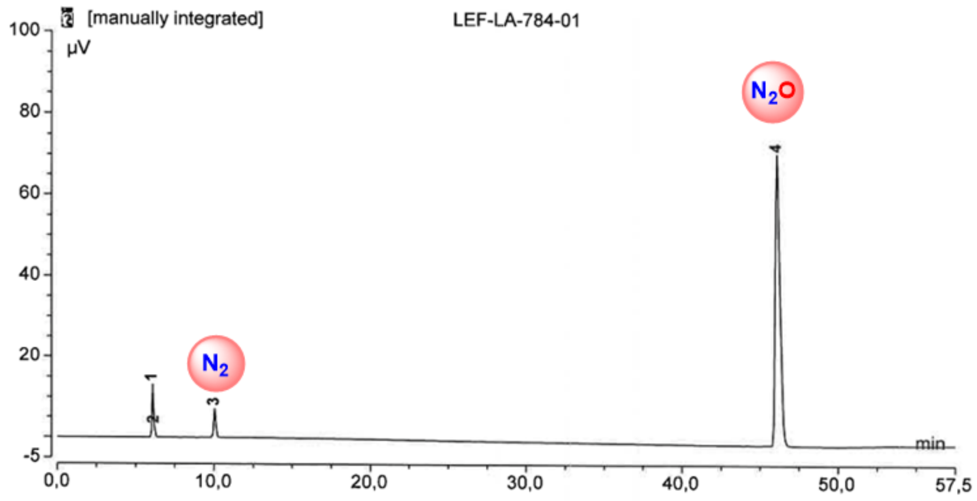

Sample:	**LEF-LA-784-01**	Instrument:	**GC_113**
Sequenz:	**8334 LEF-LA VD**	Measured:	**16.07.21 11:25**
Sequenz date:	**16.07.21**	Processing M.:	**LEF-LA**
		Report-File:	**784-01**

Zuordnung siehe PAY-PA-642-01 20/7477 und LEF-LA-442-01 20/7680

No.	Ret.Time min	area-% %	Peak Name
1	6,07	4,85	Argon (keine Basislinientrennung)
2	6,19	0,74	O2 (keine Basislinientrennung)
3	10,05	4,21	N2
4	46,04	90,19	N2O

Fig. S50. GC-TCD analysis of the headspace of a vial containing Zn+DMA+N_2O stirred overnight (entry 6, Table S16).

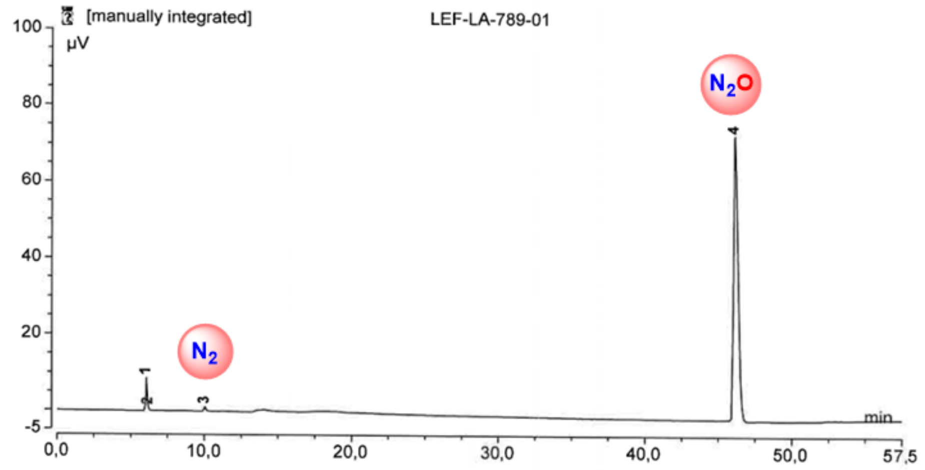

Sample:	**LEF-LA-789-01**	Instrument:	**GC_113**
Sequenz:	**8338 LEF-LA VD**	Measured:	**20.07.21 09:38**
Sequenz date:	**19.07.21**	Processing M.:	**LEF-LA**
		Report-File:	**789-01**

Zuordnung siehe PAY-PA-642-01 20/7477 und LEF-LA-442-01 20/7680

No.	Ret.Time min	area-% %	Peak Name
1	6,06	3,18	Argon (keine Basislinientrennung)
2	6,18	0,16	O2 (keine Basislinientrennung)
3	10,04	0,72	N2
4	46,10	95,94	N2O

Fig. S51. GC-TCD analysis of the headspace of a vial containing Zn+DMA+N_2O stirred overnight (entry 7, Table S16).

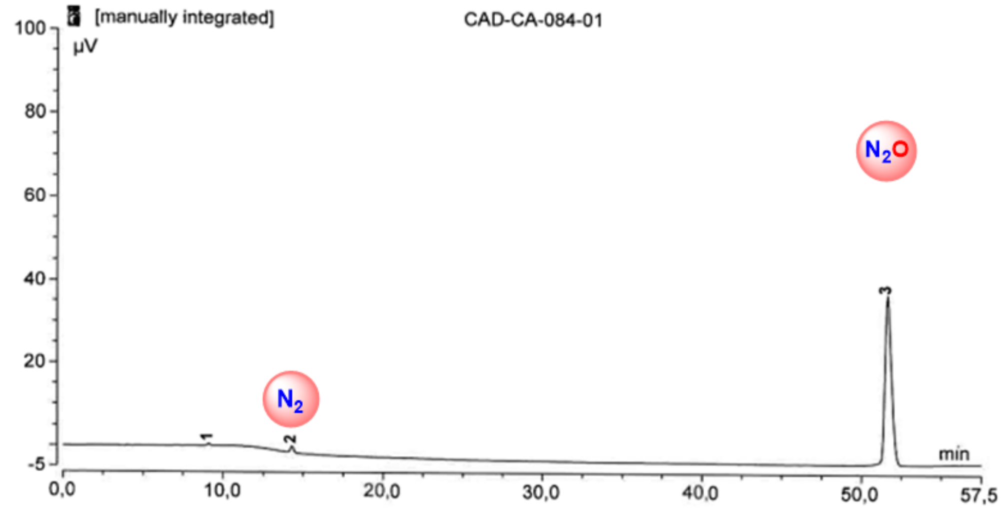

Sample:	**CAD-CA-084-01**	Instrument:	**GC_311**
Sequenz:	**8134 CAD-CA VD**	Measured:	**22.03.21 13:01**
Sequenz date:	**22.03.21**	Processing M.:	**CAD-CA**
		Report-File:	**084-01**

N2O
Zuordnung siehe PAY-PA-642-01 20/7477 und LEF-LA-442-01 20/7680

No.	Ret.Time min	area-% %	Peak Name
1	9,09	0,45	O2
2	14,29	1,84	N2
3	51,62	97,71	N2O

Fig. S52. GC-TCD analysis of the headspace of a vial containing N_2O (entry 8, Table S16).

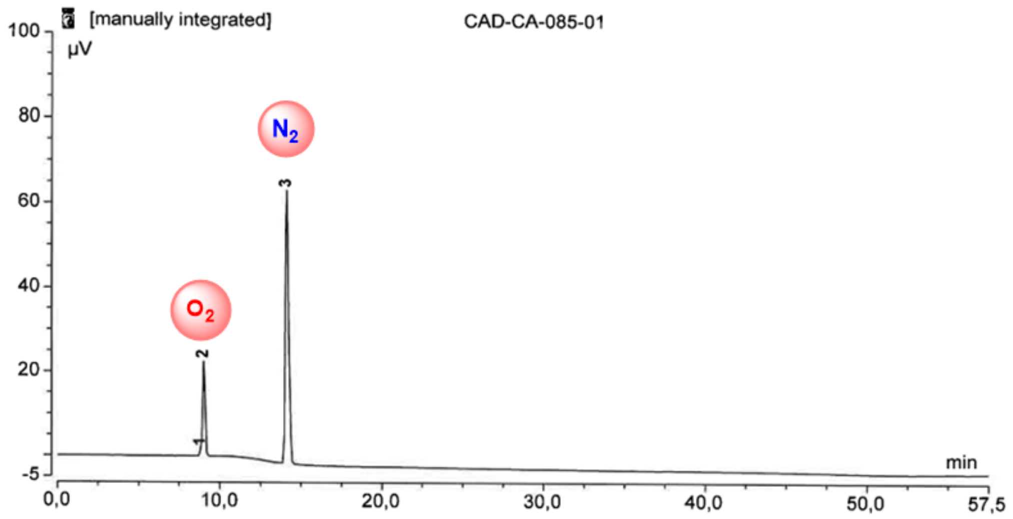

Sample:	CAD-CA-085-01	Instrument:	GC_311
Sequenz:	8135 CAD-CA VD	Measured:	22.03.21 11:42
Sequenz date:	22.03.21	Processing M.:	CAD-CA
		Report-File:	085-01

Raumluft
Zuordnung siehe PAY-PA-642-01 20/7477 und LEF-LA-442-01 20/7680

No.	Ret.Time min	area-% %	Peak Name
1	8,83	0,58	Argon (keine Basislinientrennung)
2	9,01	20,22	O2 (keine Basislinientrennung)
3	14,10	79,19	N2

Fig. S53. GC-TCD analysis of the headspace of a vial containing air (entry 9, Table S16).

10. X-ray crystal structure analysis of 10

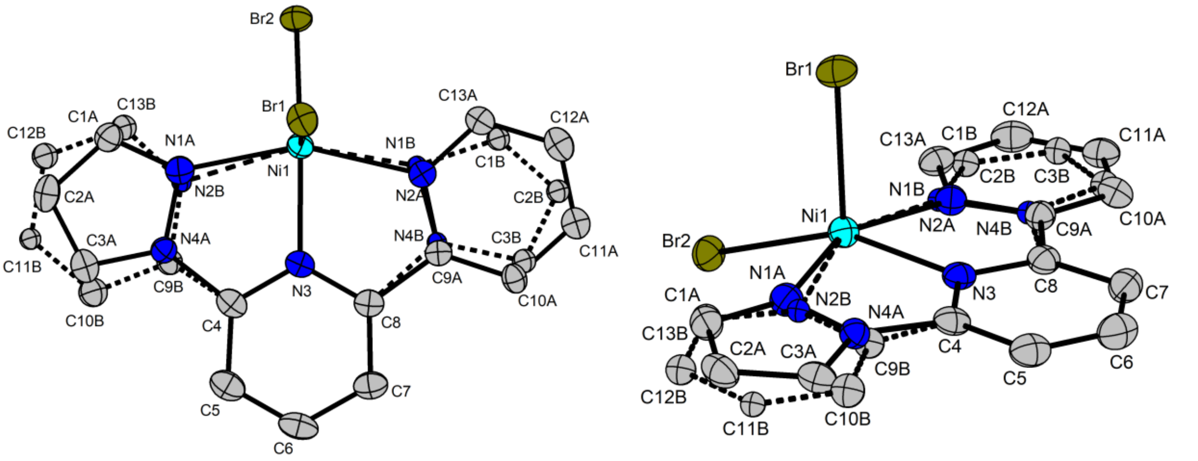

Molecular structure of **10** showing the partial disorder of the *N*,*N*,*N*-(6-pyrazolyl-2,2'-bipyridine) ligand [refined occupancy of the major component 0.763(9)] and the asymmetric binding of the two bromide groups. Left: top view, right: side view. H atoms have been omitted for clarity.

X-ray Crystal Structure Analysis of complex 10: C_{13} H_{10} Br_2 N_4 Ni, M_r = 440.78 g mol^{-1}, yellow prism, crystal size 0.03 x 0.07 x 0.10 mm^3, monoclinic, P 2_1/c [14], a = 11.0108(6) Å, b = 8.0128(7) Å, c = 15.9255(9) Å, β = 95.582(5) °, V = 1398.40(16) Å^3, T = 100(2) K, Z = 4, D_{calc} = 2.094 g·cm^3, λ = 0.71073 Å, μ(*Mo-K*$_\alpha$) = 7.095 mm^{-1}, Gaussian absorption correction (T_{min} = 0.54650, T_{max} = 0.84694), Bruker AXS Enraf-Nonius KappaCCD diffractometer with a FR591 rotating Mo-anode X-ray source, 2.848 < θ < 33.159°, 19801 measured reflections, 5333 independent reflections, 4137 reflections with $I > 2\sigma(I)$, R_{int} = 0.0575.

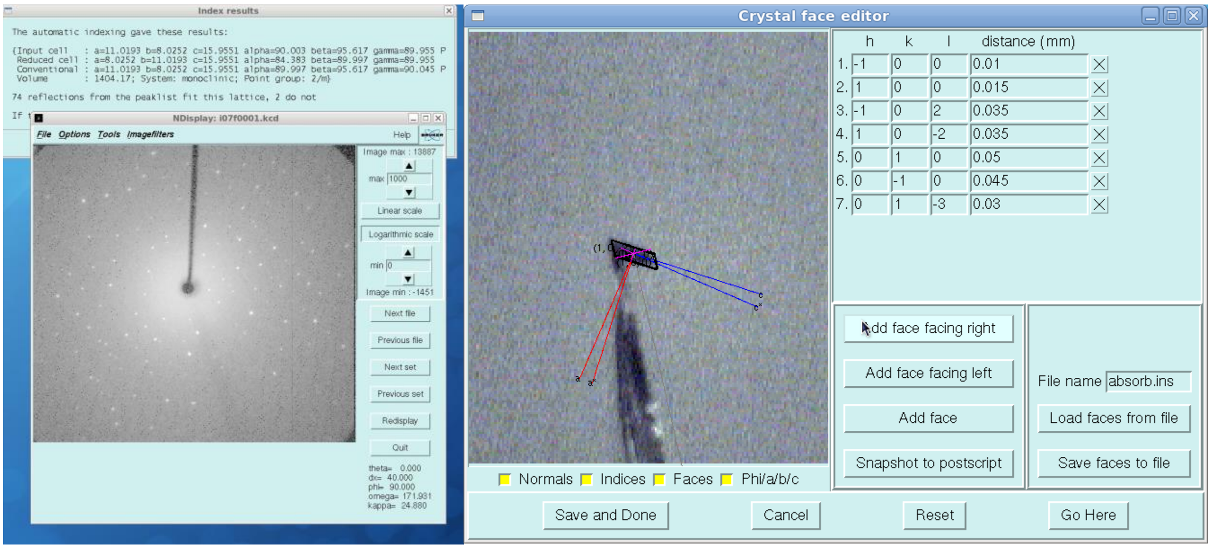

INTENSITY STATISTICS FOR DATASET

Resolution	#Data	#Theory	%Complete	Redundancy	Mean I	Mean I/s	Rmerge	Rsigma
Inf - 2.69	86	92	93.5	6.87	129.36	38.05	0.0547	0.0221
2.69 - 1.81	199	199	100.0	5.47	90.77	31.69	0.0500	0.0256
1.81 - 1.42	290	291	99.7	4.96	56.94	27.57	0.0464	0.0285
1.42 - 1.24	280	280	100.0	4.78	35.71	23.73	0.0430	0.0322
1.24 - 1.12	283	283	100.0	4.51	34.66	22.02	0.0410	0.0337
1.12 - 1.04	294	294	100.0	4.43	25.40	19.32	0.0440	0.0380
1.04 - 0.97	317	318	99.7	4.16	21.74	17.87	0.0491	0.0414
0.97 - 0.93	228	228	100.0	4.06	15.95	15.05	0.0559	0.0477
0.93 - 0.88	342	342	100.0	3.76	12.29	12.77	0.0632	0.0558
0.88 - 0.85	246	246	100.0	3.67	10.93	11.55	0.0690	0.0626
0.85 - 0.82	304	304	100.0	3.41	9.64	10.32	0.0750	0.0705
0.82 - 0.79	317	317	100.0	3.40	7.68	9.08	0.0968	0.0858
0.79 - 0.77	263	263	100.0	3.21	7.72	8.39	0.0914	0.0907
0.77 - 0.75	263	263	100.0	2.98	6.10	6.88	0.1147	0.1127
0.75 - 0.73	308	308	100.0	2.94	5.74	6.31	0.1324	0.1243
0.73 - 0.71	345	345	100.0	2.88	5.18	5.70	0.1379	0.1386
0.71 - 0.70	182	182	100.0	2.71	4.01	4.75	0.1653	0.1772
0.70 - 0.68	399	399	100.0	2.59	3.75	4.22	0.1841	0.1969
0.68 - 0.67	225	226	99.6	2.55	2.38	2.98	0.2509	0.3030
0.67 - 0.66	231	233	99.1	2.54	2.75	3.10	0.2755	0.2837
0.66 - 0.65	256	259	98.8	2.42	2.68	2.81	0.2477	0.3092
0.75 - 0.65	1946	1952	99.7	2.67	3.92	4.40	0.1751	0.1893
Inf - 0.65	5658	5672	99.8	3.61	18.92	12.55	0.0570	0.0486

The structure was solved by *SHELXT* and refined by full-matrix least-squares (*SHELXL*) against F^2 to R_1 = 0.0423 [$I > 2\sigma(I)$], wR_2 = 0.0884, 225 parameters, 30 restraints. Residual electron density: highest peak 0.88 eÅ^{-3}, 0.70 Å from Br1; deepest hole -1.25 eÅ^{-3}, 0.75 Å from Br2. The *N*,*N*,*N*-(6-pyrazolyl-2,2'-bipyridine) ligand is partially disordered over two positions with a refined occupancy of 0.763(9) for the major component. Because of the close proximity to minor component atoms, C1A, C9A, N1A, N2A and N4A of the major component were restrained to have isotropic atomic displacement parameters with an effective standard deviation of 0.001 and the atomic displacement parameters of the atoms N1B and N4B in the minor component pyrazol ring were contrained to be equal. **CCDC 2114695**.

Crystal data and structure refinement.

Identification code	**13717**
Empirical formula	C_{13} H_{10} Br_2 N_4 Ni
Color	yellow
Formula weight	440.78 g·mol^{-1}
Temperature	100(2) K
Wavelength	0.71073 Å
Crystal system	monoclinic

Space group	P 21/c, (no. 14)	
Unit cell dimensions	a = 11.0108(6) Å	α= 90°.
	b = 8.0128(7) Å	β= 95.582(5)°.
	c = 15.9255(9) Å	γ = 90°.
Volume	1398.40(16) Å^3	
Z	4	
Density (calculated)	2.094 Mg·m^{-3}	
Absorption coefficient	7.095 mm^{-1}	
F(000)	856 e	
Crystal size	0.10 x 0.07 x 0.03 mm^3	
θ range for data collection	2.848 to 33.159°.	
Index ranges	$-16 \le h \le 16$, $-12\le k \le 12$, $-22\le l \le 24$	
Reflections collected	19801	
Independent reflections	5333 [R_{int} = 0.0575]	
Reflections with I>2σ(I)	4137	
Completeness to θ = 25.242°	99.9 %	
Absorption correction	Gaussian	
Max. and min. transmission	0.84694 and 0.54650	
Refinement method	Full-matrix least-squares on F^2	
Data / restraints / parameters	5333 / 30 / 225	
Goodness-of-fit on F^2	1.109	
Final R indices [I>2σ(I)]	R_1 = 0.0423	wR^2 = 0.0814
R indices (all data)	R_1 = 0.0639	wR^2 = 0.0884
Extinction coefficient	0	
Largest diff. peak and hole	0.881 and -1.247 e·Å^{-3}	

Atomic coordinates and equivalent isotropic displacement parameters (Å²).

U_{eq} is defined as one third of the trace of the orthogonalized U_{ij} tensor.

	x	y	z	U_{eq}
C(4)	0.8297(3)	0.4166(4)	0.4779(2)	0.022(1)
C(5)	0.8504(3)	0.3744(4)	0.3960(2)	0.028(1)
C(6)	0.7729(3)	0.4433(4)	0.3318(2)	0.031(1)
C(7)	0.6772(3)	0.5482(4)	0.3493(2)	0.026(1)
C(8)	0.6629(3)	0.5825(4)	0.4331(2)	0.021(1)
Br(1)	0.8389(1)	0.8384(1)	0.6240(1)	0.027(1)
Br(2)	0.6498(1)	0.5316(1)	0.7514(1)	0.025(1)
N(3)	0.7405(2)	0.5192(3)	0.4952(2)	0.020(1)
Ni(1)	0.7208(1)	0.5797(1)	0.6147(1)	0.020(1)
C(1A)	0.9448(5)	0.3383(8)	0.6865(4)	0.023(1)
C(2A)	1.0288(4)	0.2370(5)	0.6500(3)	0.025(1)
C(3A)	0.9979(4)	0.2513(6)	0.5646(4)	0.023(1)
C(9A)	0.5660(9)	0.6830(12)	0.4613(5)	0.019(2)
C(10A)	0.4773(6)	0.7586(8)	0.4112(5)	0.020(1)
C(11A)	0.3867(4)	0.8480(5)	0.4478(3)	0.021(1)
C(12A)	0.3898(4)	0.8592(5)	0.5341(3)	0.023(1)
C(13A)	0.4836(5)	0.7791(6)	0.5838(4)	0.020(1)
N(1A)	0.8669(6)	0.4144(8)	0.6302(4)	0.023(2)
N(2A)	0.5669(8)	0.6935(11)	0.5475(5)	0.020(2)
N(4A)	0.9017(5)	0.3590(7)	0.5542(4)	0.019(1)
C(1B)	0.4621(17)	0.787(2)	0.5609(11)	0.018(4)
C(2B)	0.4006(13)	0.8407(18)	0.4858(11)	0.020(3)
C(3B)	0.461(2)	0.774(3)	0.4255(14)	0.017(5)
C(9B)	0.891(2)	0.347(3)	0.5420(14)	0.020(5)
C(10B)	0.9859(18)	0.237(3)	0.5372(12)	0.026(4)
C(11B)	1.0482(12)	0.1799(17)	0.6112(9)	0.021(3)
C(12B)	1.0156(14)	0.2349(19)	0.6885(11)	0.024(3)
C(13B)	0.9212(17)	0.352(2)	0.6920(12)	0.020(4)
N(1B)	0.572(2)	0.689(3)	0.5570(14)	0.013(4)
N(2B)	0.8608(17)	0.399(2)	0.6189(11)	0.012(3)
N(4B)	0.566(2)	0.686(3)	0.4721(13)	0.013(4)

Bond lengths [Å] and angles [°].

C(4)-C(9B)	1.29(2)	C(4)-N(3)	1.330(4)
C(4)-C(5)	1.387(4)	C(4)-N(4A)	1.461(6)
C(5)-C(6)	1.382(5)	C(5)-H(5)	0.9500
C(6)-C(7)	1.398(5)	C(6)-H(6)	0.9500
C(7)-C(8)	1.386(4)	C(7)-H(7)	0.9500
C(8)-N(3)	1.343(4)	C(8)-C(9A)	1.442(10)
C(8)-N(4B)	1.53(2)	Br(1)-Ni(1)	2.4434(5)
Br(2)-Ni(1)	2.4149(5)	N(3)-Ni(1)	1.995(3)
Ni(1)-N(1B)	2.00(2)	Ni(1)-N(1A)	2.080(7)
Ni(1)-N(2B)	2.111(18)	Ni(1)-N(2A)	2.120(8)
C(1A)-N(1A)	1.327(8)	C(1A)-C(2A)	1.399(8)
C(1A)-H(1A)	0.9500	C(2A)-C(3A)	1.374(8)
C(2A)-H(2A)	0.9500	C(3A)-N(4A)	1.364(7)
C(3A)-H(3A)	0.9500	C(9A)-C(10A)	1.344(11)
C(9A)-N(2A)	1.375(10)	C(10A)-C(11A)	1.400(8)
C(10A)-H(10A)	0.9500	C(11A)-C(12A)	1.374(6)
C(11A)-H(11A)	0.9500	C(12A)-C(13A)	1.395(7)
C(12A)-H(12A)	0.9500	C(13A)-N(2A)	1.322(10)
C(13A)-H(13A)	0.9500	N(1A)-N(4A)	1.378(8)
C(1B)-C(2B)	1.38(2)	C(1B)-N(1B)	1.45(3)
C(1B)-H(1B)	0.9500	C(2B)-C(3B)	1.33(2)
C(2B)-H(2B)	0.9500	C(3B)-N(4B)	1.49(3)
C(3B)-H(3B)	0.9500	C(9B)-N(2B)	1.36(3)
C(9B)-C(10B)	1.38(3)	C(10B)-C(11B)	1.38(2)
C(10B)-H(10B)	0.9500	C(11B)-C(12B)	1.39(2)
C(11B)-H(11B)	0.9500	C(12B)-C(13B)	1.40(2)
C(12B)-H(12B)	0.9500	C(13B)-N(2B)	1.34(3)
C(13B)-H(13B)	0.9500	N(1B)-N(4B)	1.35(3)
C(9B)-C(4)-N(3)	116.1(11)	C(9B)-C(4)-C(5)	121.1(11)
N(3)-C(4)-C(5)	122.6(3)	N(3)-C(4)-N(4A)	112.0(3)
C(5)-C(4)-N(4A)	125.4(4)	C(6)-C(5)-C(4)	116.9(3)
C(6)-C(5)-H(5)	121.6	C(4)-C(5)-H(5)	121.6
C(5)-C(6)-C(7)	121.0(3)	C(5)-C(6)-H(6)	119.5
C(7)-C(6)-H(6)	119.5	C(8)-C(7)-C(6)	118.1(3)
C(8)-C(7)-H(7)	121.0	C(6)-C(7)-H(7)	121.0

N(3)-C(8)-C(7)	120.7(3)	N(3)-C(8)-C(9A)	114.7(4)
C(7)-C(8)-C(9A)	124.5(4)	N(3)-C(8)-N(4B)	108.9(8)
C(7)-C(8)-N(4B)	130.3(8)	C(4)-N(3)-C(8)	120.6(3)
C(4)-N(3)-Ni(1)	120.0(2)	C(8)-N(3)-Ni(1)	119.4(2)
N(3)-Ni(1)-N(1B)	80.1(7)	N(3)-Ni(1)-N(1A)	78.72(18)
N(3)-Ni(1)-N(2B)	73.6(5)	N(1B)-Ni(1)-N(2B)	151.4(8)
N(3)-Ni(1)-N(2A)	77.3(2)	N(1A)-Ni(1)-N(2A)	154.8(3)
N(3)-Ni(1)-Br(2)	153.55(7)	N(1B)-Ni(1)-Br(2)	99.3(7)
N(1A)-Ni(1)-Br(2)	96.04(18)	N(2B)-Ni(1)-Br(2)	99.3(5)
N(2A)-Ni(1)-Br(2)	102.1(2)	N(3)-Ni(1)-Br(1)	99.09(7)
N(1B)-Ni(1)-Br(1)	93.5(7)	N(1A)-Ni(1)-Br(1)	97.63(18)
N(2B)-Ni(1)-Br(1)	101.4(5)	N(2A)-Ni(1)-Br(1)	93.5(2)
Br(2)-Ni(1)-Br(1)	107.319(18)	N(1A)-C(1A)-C(2A)	113.3(6)
N(1A)-C(1A)-H(1A)	123.4	C(2A)-C(1A)-H(1A)	123.3
C(3A)-C(2A)-C(1A)	104.7(4)	C(3A)-C(2A)-H(2A)	127.7
C(1A)-C(2A)-H(2A)	127.7	N(4A)-C(3A)-C(2A)	106.7(5)
N(4A)-C(3A)-H(3A)	126.7	C(2A)-C(3A)-H(3A)	126.7
C(10A)-C(9A)-N(2A)	120.0(8)	C(10A)-C(9A)-C(8)	125.7(6)
N(2A)-C(9A)-C(8)	114.3(7)	C(9A)-C(10A)-C(11A)	119.3(7)
C(9A)-C(10A)-H(10A)	120.3	C(11A)-C(10A)-H(10A)	120.3
C(12A)-C(11A)-C(10A)	120.0(5)	C(12A)-C(11A)-H(11A)	120.0
C(10A)-C(11A)-H(11A)	120.0	C(11A)-C(12A)-C(13A)	118.8(4)
C(11A)-C(12A)-H(12A)	120.6	C(13A)-C(12A)-H(12A)	120.6
N(2A)-C(13A)-C(12A)	119.8(6)	N(2A)-C(13A)-H(13A)	120.1
C(12A)-C(13A)-H(13A)	120.1	C(1A)-N(1A)-N(4A)	103.2(6)
C(1A)-N(1A)-Ni(1)	144.5(5)	N(4A)-N(1A)-Ni(1)	112.2(4)
C(13A)-N(2A)-C(9A)	122.0(8)	C(13A)-N(2A)-Ni(1)	123.9(5)
C(9A)-N(2A)-Ni(1)	113.9(6)	C(3A)-N(4A)-N(1A)	112.1(5)
C(3A)-N(4A)-C(4)	130.8(5)	N(1A)-N(4A)-C(4)	117.0(5)
C(2B)-C(1B)-N(1B)	118.2(17)	C(2B)-C(1B)-H(1B)	120.9
N(1B)-C(1B)-H(1B)	120.9	C(3B)-C(2B)-C(1B)	105.2(17)
C(3B)-C(2B)-H(2B)	127.4	C(1B)-C(2B)-H(2B)	127.4
C(2B)-C(3B)-N(4B)	104.3(19)	C(2B)-C(3B)-H(3B)	127.8
N(4B)-C(3B)-H(3B)	127.8	C(4)-C(9B)-N(2B)	115.2(19)
C(4)-C(9B)-C(10B)	124.9(19)	N(2B)-C(9B)-C(10B)	119.7(19)
C(9B)-C(10B)-C(11B)	118.9(18)	C(9B)-C(10B)-H(10B)	120.6
C(11B)-C(10B)-H(10B)	120.6	C(10B)-C(11B)-C(12B)	120.0(14)
C(10B)-C(11B)-H(11B)	120.0	C(12B)-C(11B)-H(11B)	120.0

C(11B)-C(12B)-C(13B)	120.3(15)	C(11B)-C(12B)-H(12B)	119.9
C(13B)-C(12B)-H(12B)	119.9	N(2B)-C(13B)-C(12B)	117.5(17)
N(2B)-C(13B)-H(13B)	121.3	C(12B)-C(13B)-H(13B)	121.3
N(4B)-N(1B)-C(1B)	95.2(19)	N(4B)-N(1B)-Ni(1)	114.4(18)
C(1B)-N(1B)-Ni(1)	150.2(17)	C(13B)-N(2B)-C(9B)	123.5(19)
C(13B)-N(2B)-Ni(1)	121.5(14)	C(9B)-N(2B)-Ni(1)	114.8(14)
N(1B)-N(4B)-C(3B)	117(2)	N(1B)-N(4B)-C(8)	116.6(18)
C(3B)-N(4B)-C(8)	126.4(15)		

Anisotropic displacement parameters (Å^2).

The anisotropic displacement factor exponent takes the form:

$-2\pi^2[h^2a^{*2}U_{11} + ... + 2 h k a^* b^* U_{12}]$.

	U_{11}	U_{22}	U_{33}	U_{23}	U_{13}	U_{12}
C(4)	0.022(1)	0.018(1)	0.026(1)	0.001(1)	0.009(1)	-0.004(1)
C(5)	0.030(2)	0.025(2)	0.032(2)	-0.003(1)	0.012(1)	-0.005(1)
C(6)	0.038(2)	0.031(2)	0.027(2)	-0.006(1)	0.015(1)	-0.007(1)
C(7)	0.028(2)	0.028(2)	0.022(1)	-0.001(1)	0.005(1)	-0.007(1)
C(8)	0.022(1)	0.020(1)	0.022(1)	0.001(1)	0.005(1)	-0.005(1)
Br(1)	0.022(1)	0.024(1)	0.036(1)	-0.003(1)	0.002(1)	-0.005(1)
Br(2)	0.026(1)	0.028(1)	0.020(1)	0.000(1)	0.006(1)	-0.004(1)
N(3)	0.022(1)	0.017(1)	0.022(1)	0.000(1)	0.006(1)	-0.004(1)
Ni(1)	0.020(1)	0.020(1)	0.019(1)	0.000(1)	0.004(1)	-0.001(1)
C(1A)	0.023(2)	0.023(2)	0.023(2)	0.002(1)	0.002(1)	0.000(1)
C(2A)	0.022(2)	0.023(2)	0.031(3)	0.007(2)	-0.001(2)	0.001(2)
C(3A)	0.021(2)	0.020(2)	0.030(3)	0.002(2)	0.003(2)	-0.001(2)
C(9A)	0.020(2)	0.019(2)	0.019(2)	0.000(1)	0.002(1)	-0.002(1)
C(10A)	0.018(2)	0.018(2)	0.026(3)	0.002(2)	0.006(2)	0.001(2)
C(11A)	0.020(2)	0.018(2)	0.026(2)	0.000(2)	0.002(2)	-0.003(1)
C(12A)	0.018(2)	0.020(2)	0.033(2)	-0.002(2)	0.005(2)	-0.003(1)
C(13A)	0.018(2)	0.020(2)	0.023(2)	-0.002(2)	0.003(2)	-0.004(2)
N(1A)	0.023(2)	0.023(2)	0.023(2)	0.000(1)	0.003(1)	-0.001(1)
N(2A)	0.021(2)	0.020(2)	0.021(2)	0.001(1)	0.002(1)	-0.002(1)
N(4A)	0.019(2)	0.019(1)	0.019(2)	0.001(1)	0.002(1)	-0.001(1)

Hydrogen coordinates and isotropic displacement parameters (Å^2).

	x	y	z	U_{eq}
H(5)	0.9149	0.3016	0.3846	0.034
H(6)	0.7848	0.4189	0.2748	0.038
H(7)	0.6235	0.5946	0.3051	0.031
H(1A)	0.9436	0.3512	0.7458	0.028
H(2A)	1.0930	0.1727	0.6781	0.030
H(3A)	1.0362	0.1967	0.5213	0.028
H(10A)	0.4761	0.7515	0.3516	0.024
H(11A)	0.3231	0.9009	0.4130	0.026
H(12A)	0.3291	0.9203	0.5594	0.028
H(13A)	0.4876	0.7861	0.6435	0.024
H(1B)	0.4328	0.8130	0.6135	0.022
H(2B)	0.3305	0.9103	0.4789	0.024
H(3B)	0.4411	0.7815	0.3662	0.021
H(10B)	1.0084	0.2014	0.4840	0.031
H(11B)	1.1134	0.1029	0.6090	0.025
H(12B)	1.0573	0.1933	0.7392	0.029
H(13B)	0.9010	0.3954	0.7444	0.024

11. Spectra of new compounds

^{1}H NMR (400 MHz, DMSO-*d₆*) of compound **L50.**

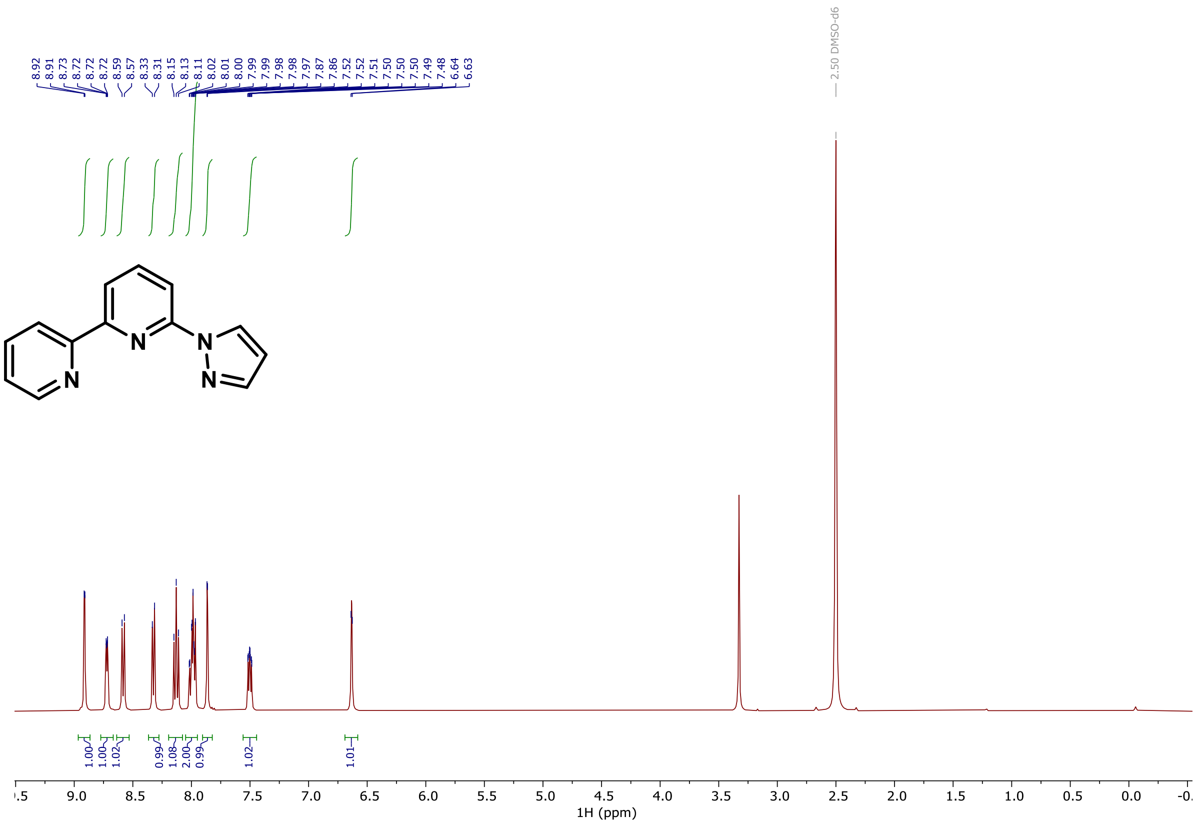

^{13}C NMR (101 MHz, DMSO-*d₆*) of compound **L50.**

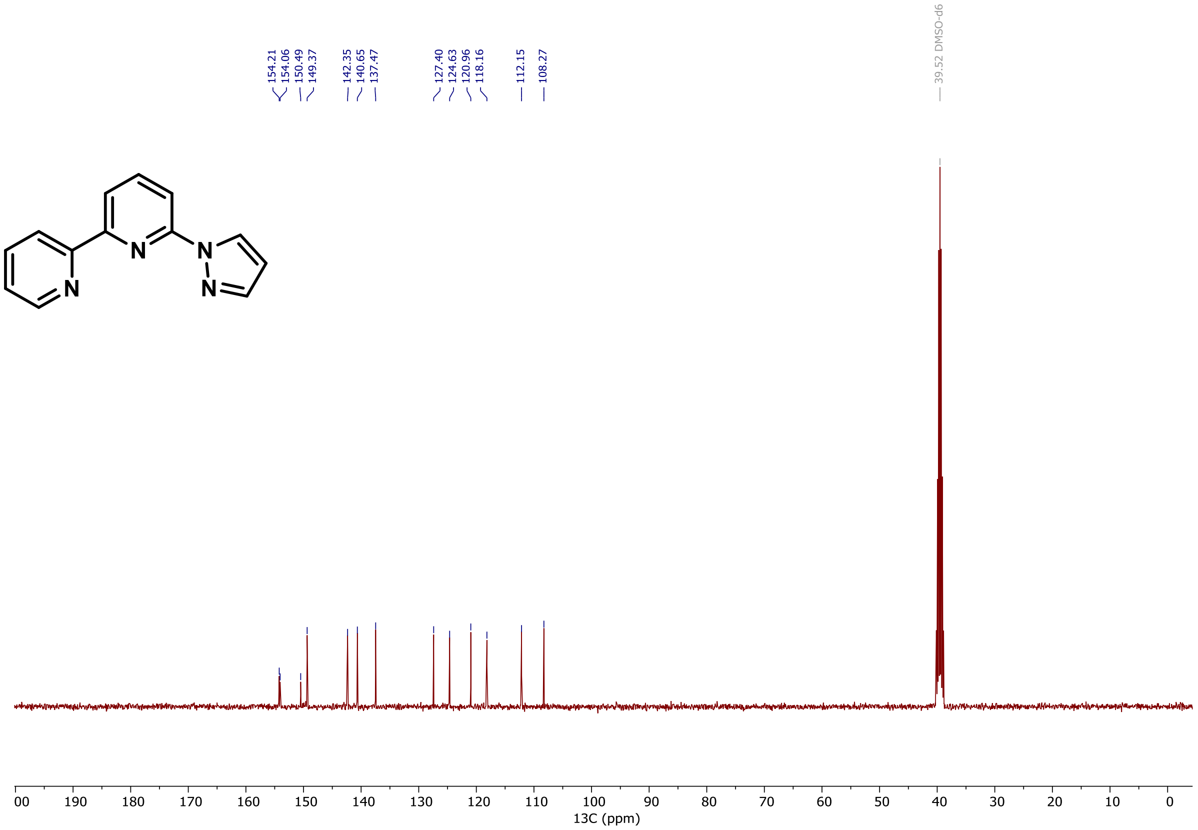

^{1}H NMR (400 MHz, $CDCl_3$) of compound **L36.**

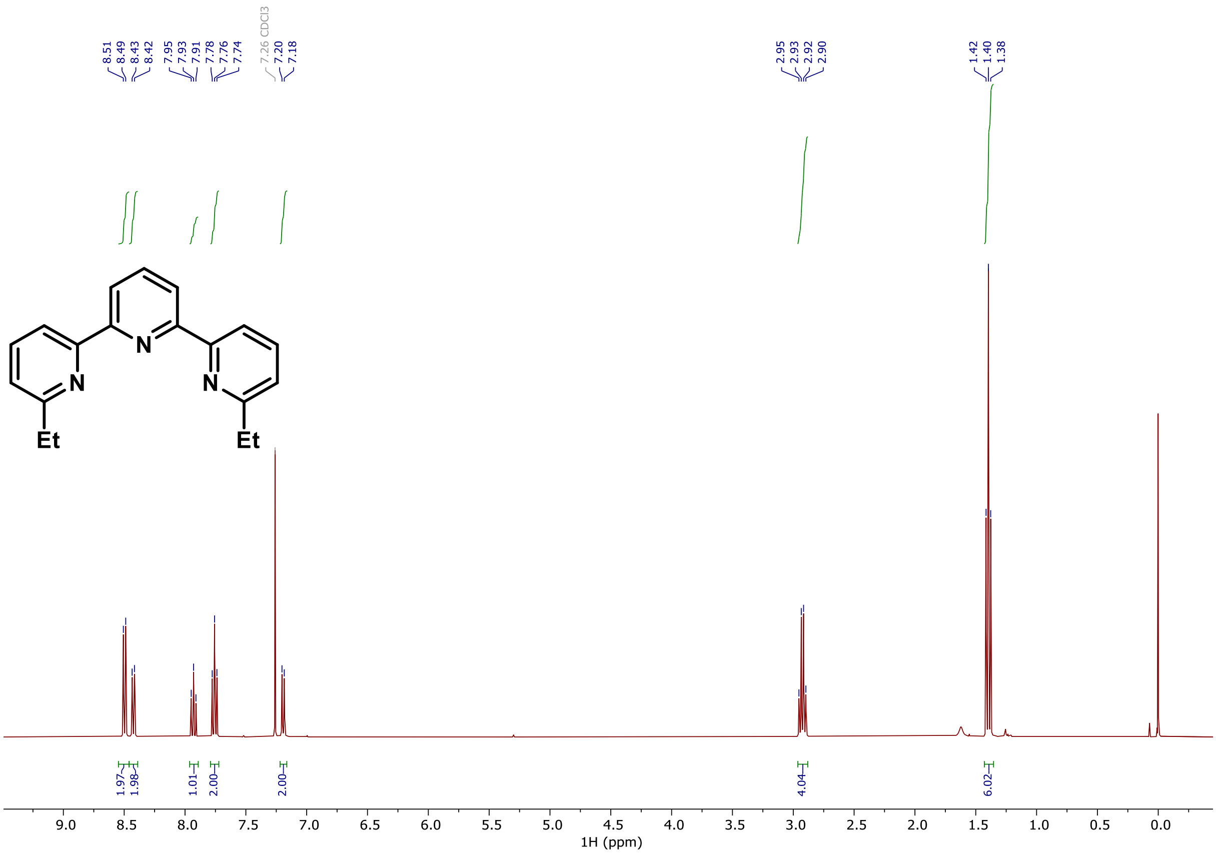

^{13}C NMR (101 MHz, $CDCl_3$) of compound **L36.**

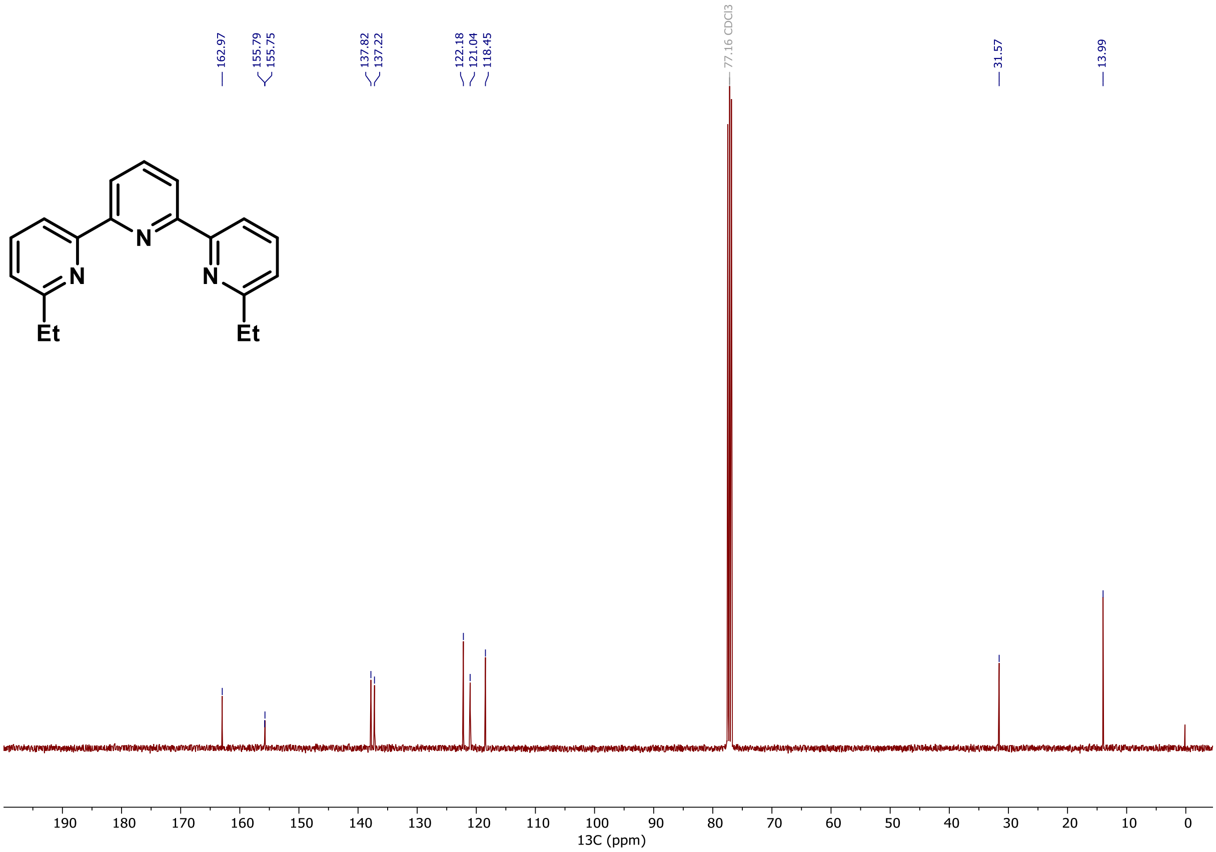

^{1}H NMR (400 MHz, $CDCl_3$) of compound **L37.**

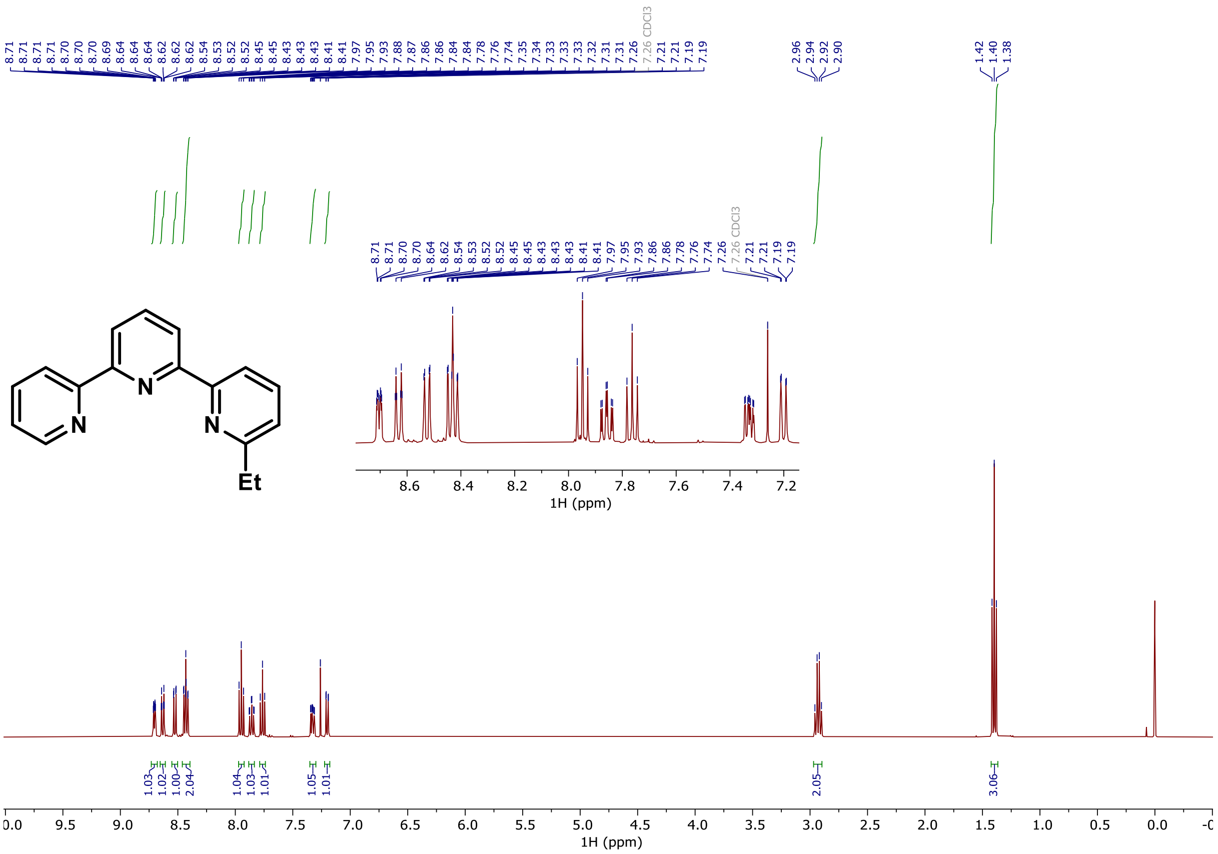

^{13}C NMR (101 MHz, $CDCl_3$) of compound **L37.**

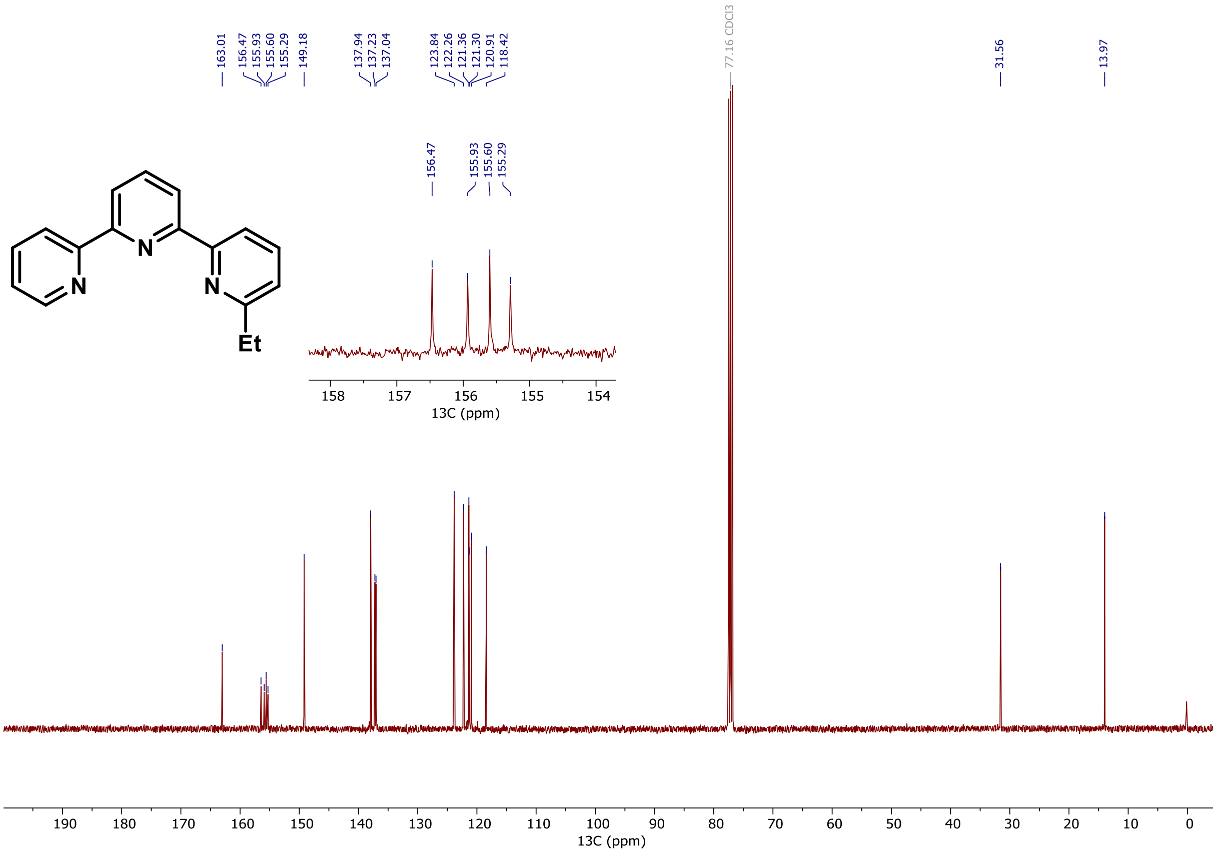

^{1}H NMR (400 MHz, CDCl$_3$) of compound **L42.**

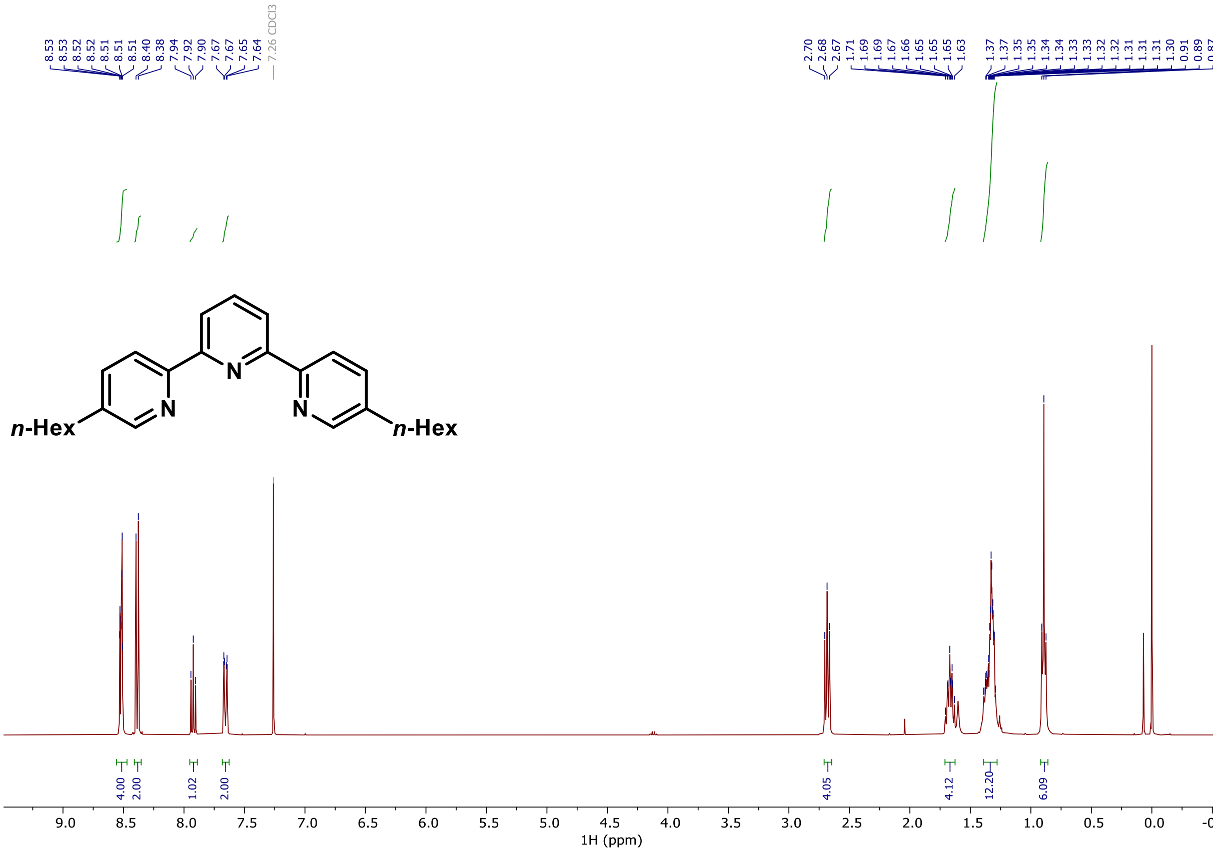

^{13}C NMR (101 MHz, CDCl$_3$) of compound **L42.**

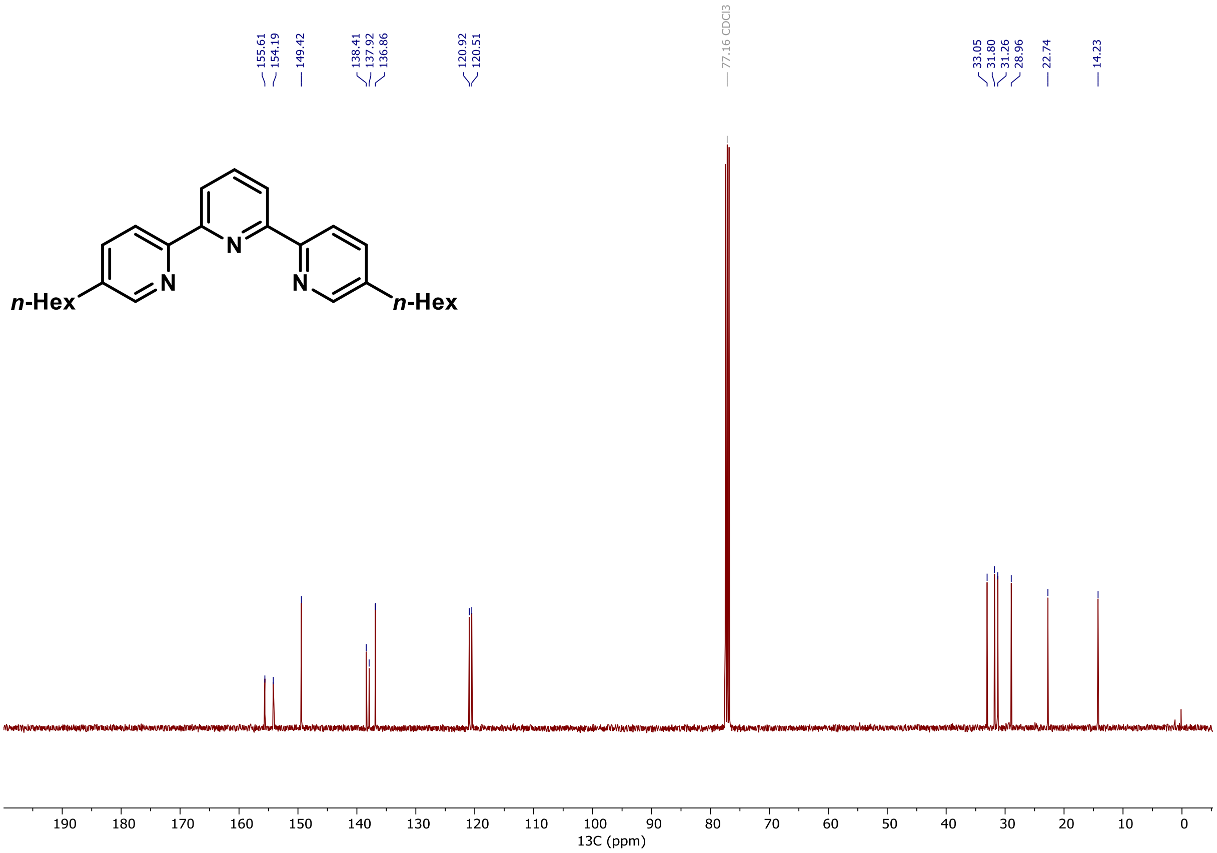

^{1}H NMR (400 MHz, CDCl$_3$) of compound **L49.**

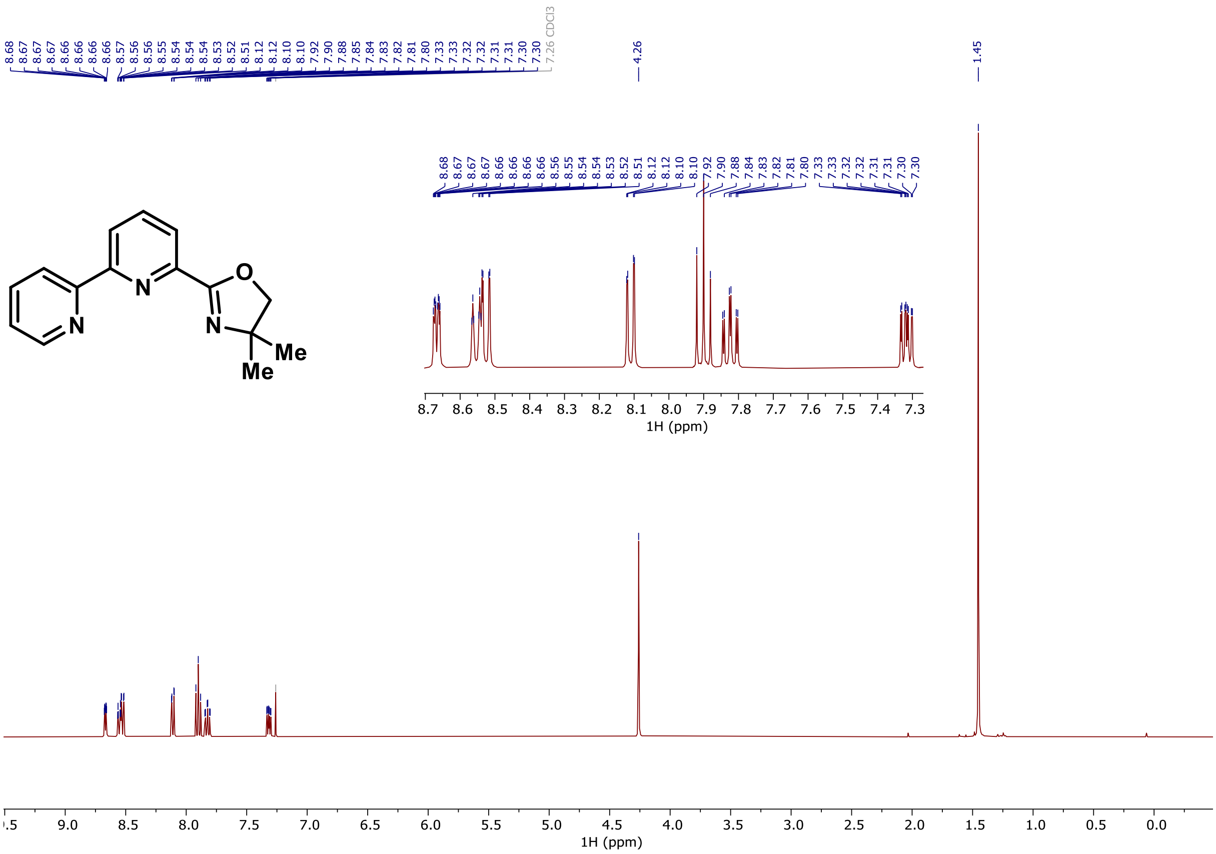

^{13}C NMR (101 MHz, CDCl$_3$) of compound **L49.**

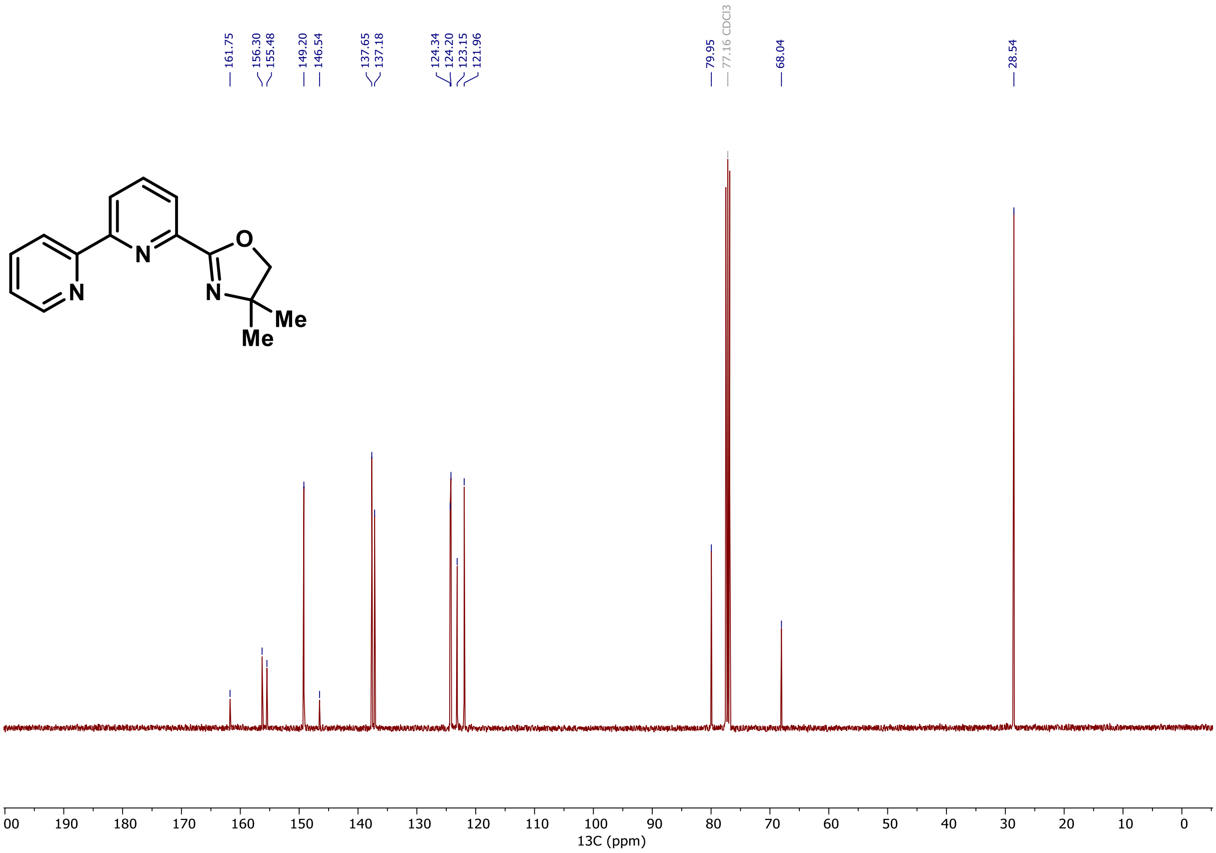

^{1}H NMR (400 MHz, CDCl$_3$) of compound **L51.**

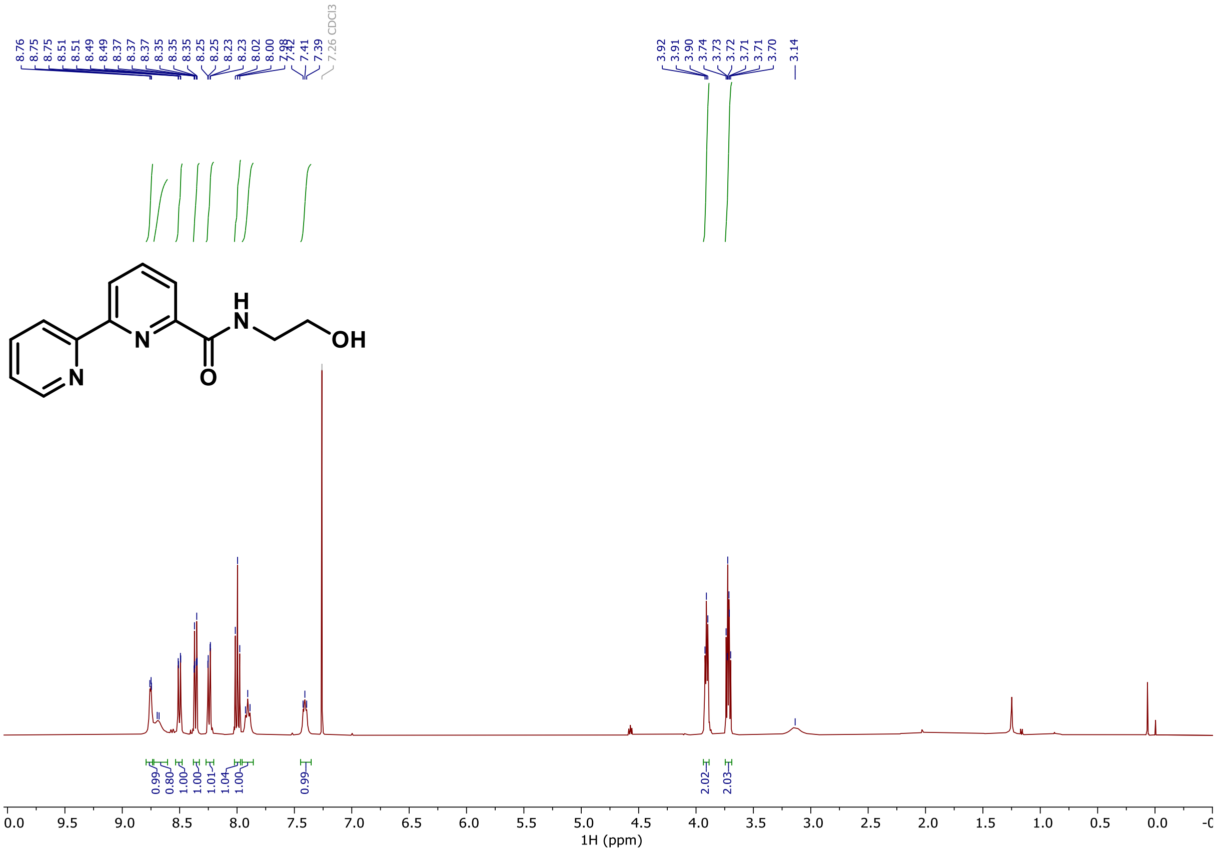

^{13}C NMR (101 MHz, CDCl$_3$) of compound **L51.**

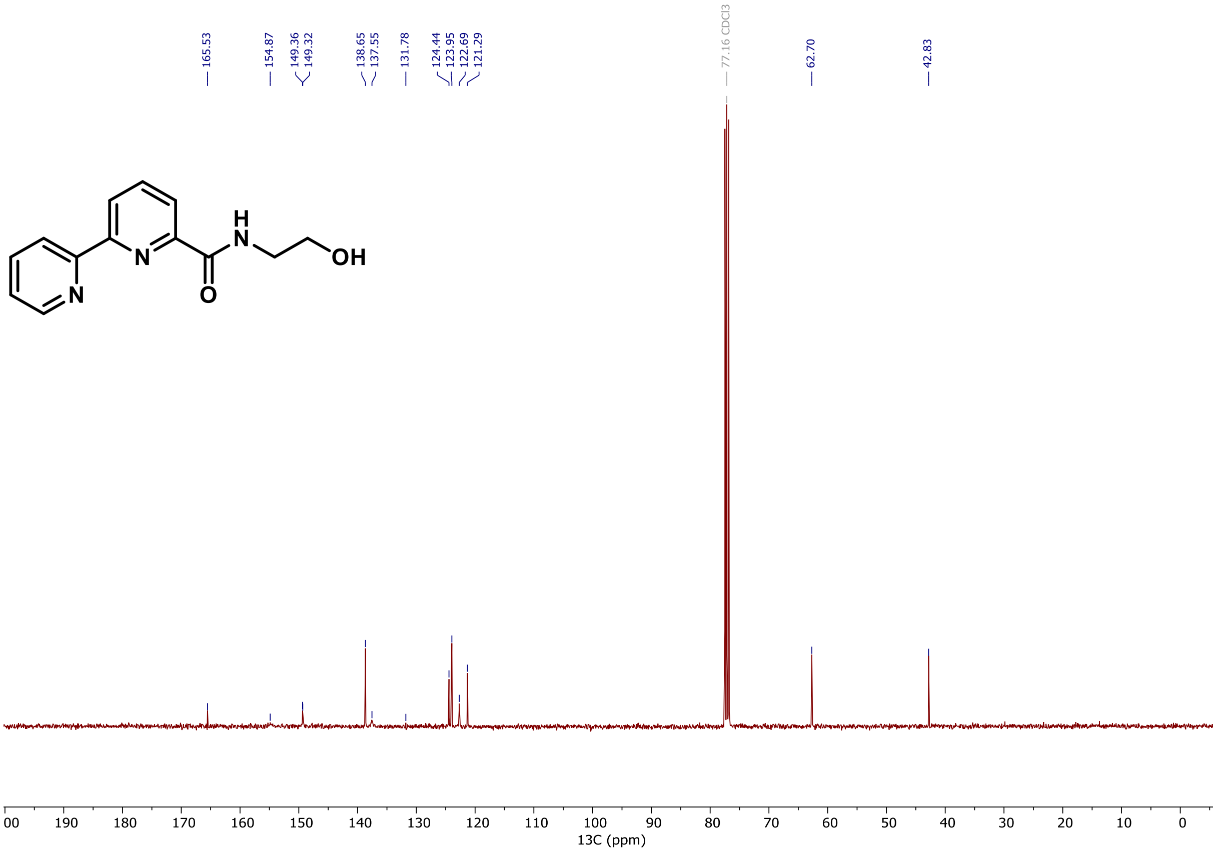

^{1}H NMR (400 MHz, $CDCl_3$) of compound **L57.**

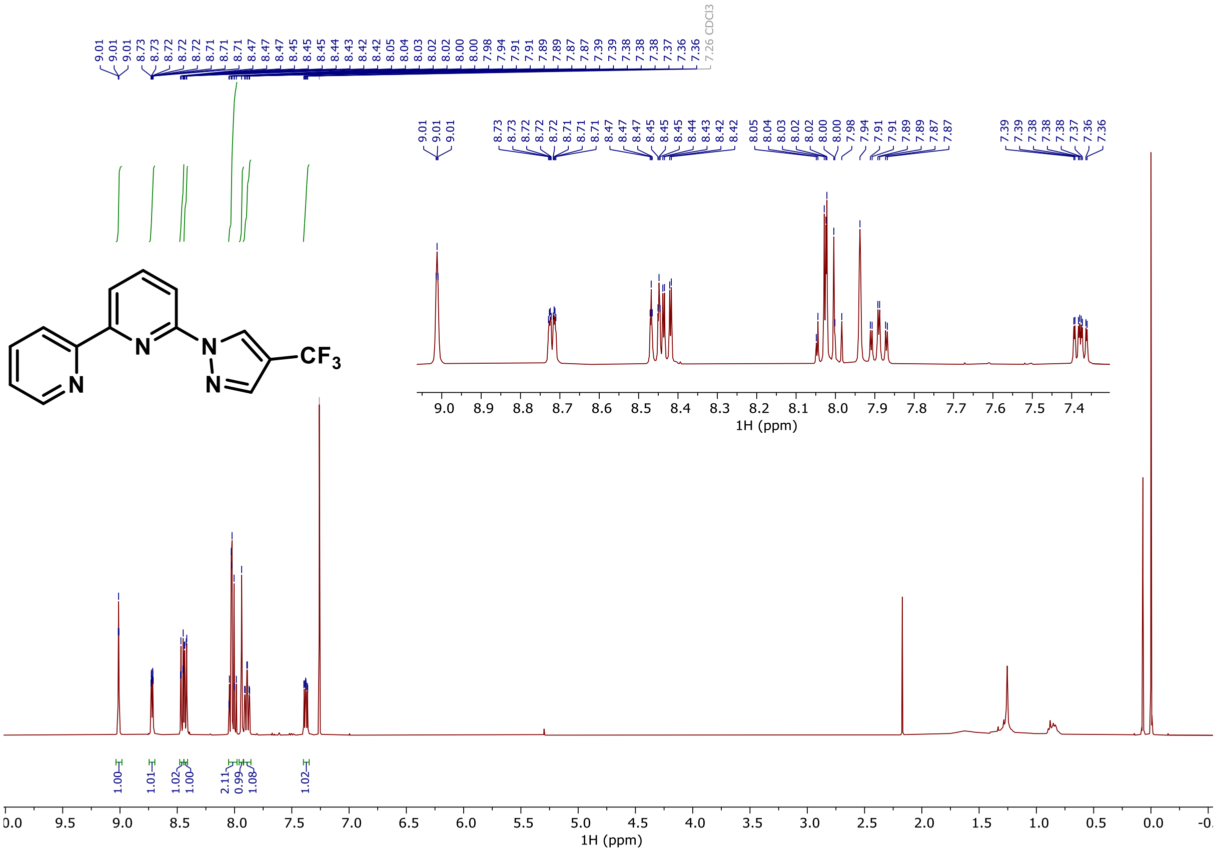

^{13}C NMR (101 MHz, $CDCl_3$) of compound **L57.**

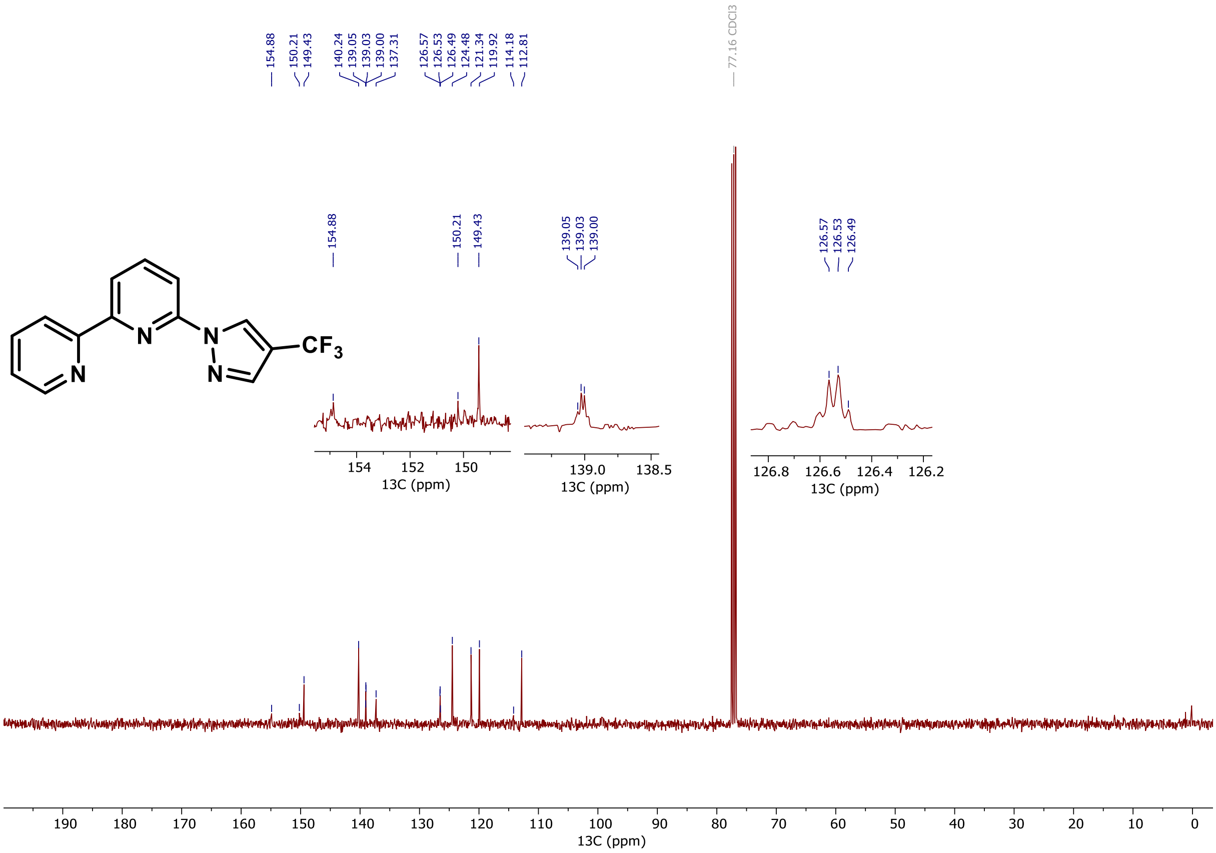

^{1}H NMR (600 MHz, $CDCl_3$) of compound **L58.**

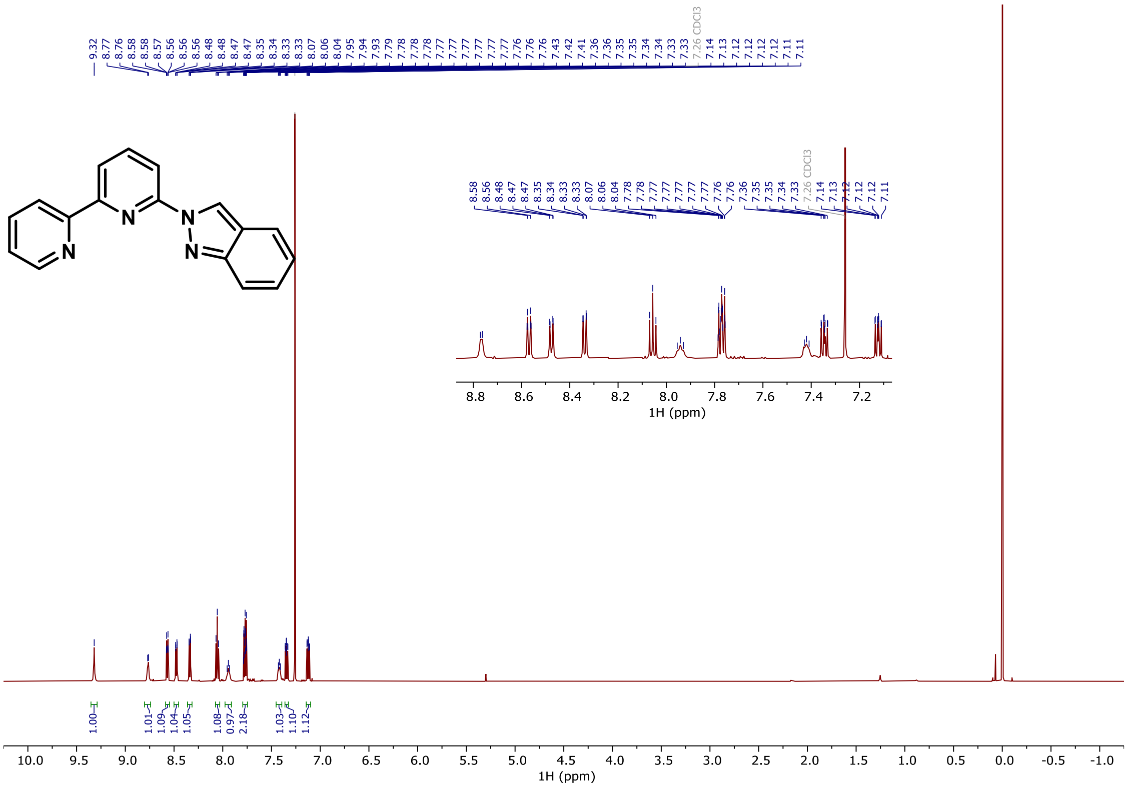

^{13}C NMR (151 MHz, $CDCl_3$) of compound **L58.**

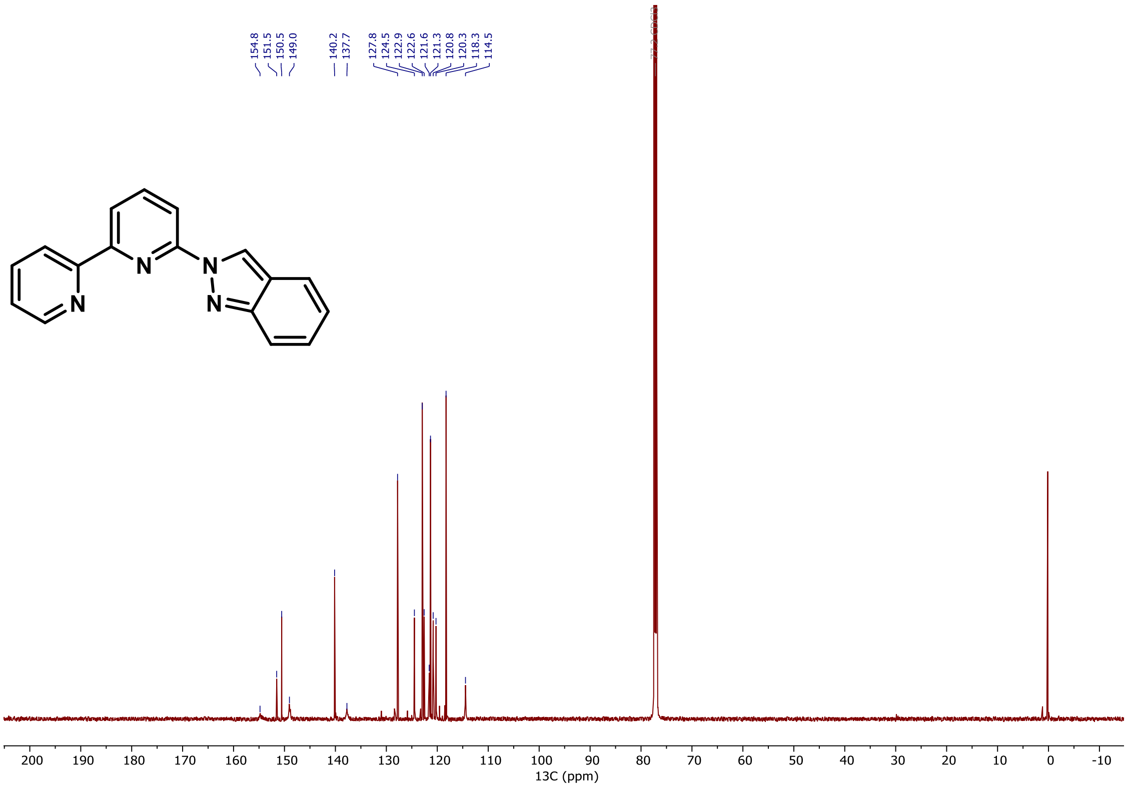

¹H NOESY ($CDCl_3$) of compound L58.

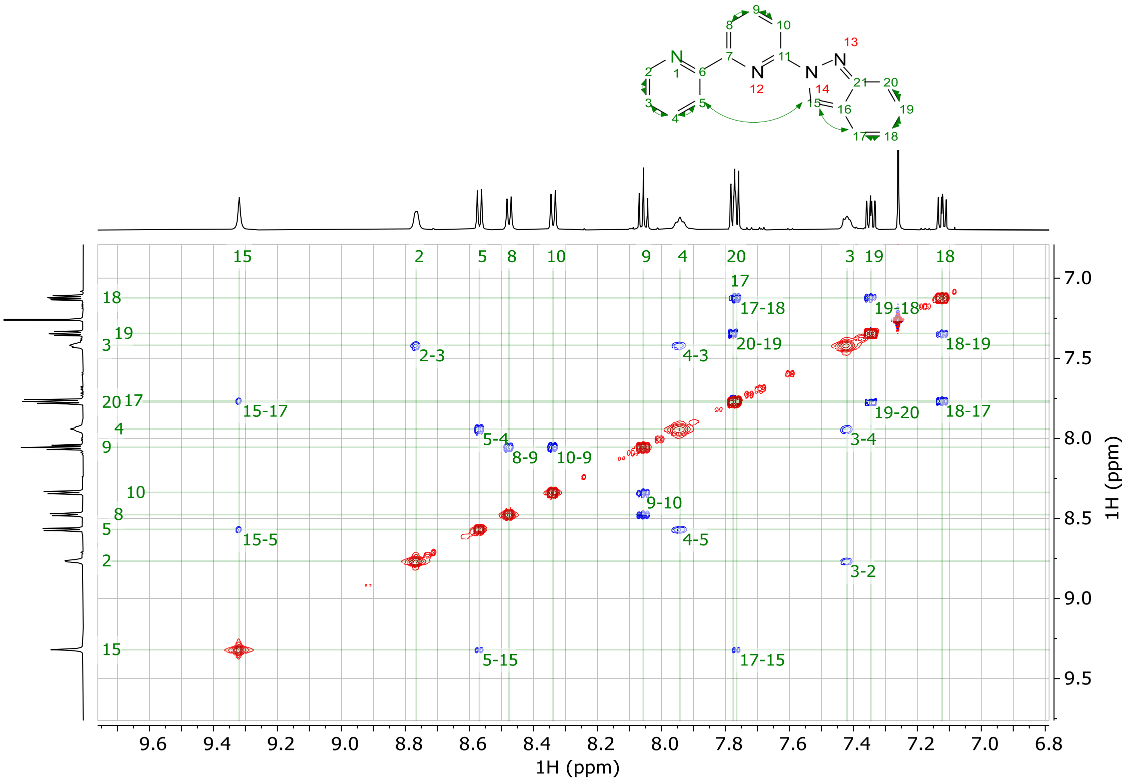

^{1}H NMR (600 MHz, CDCl3) of compound **L59.**

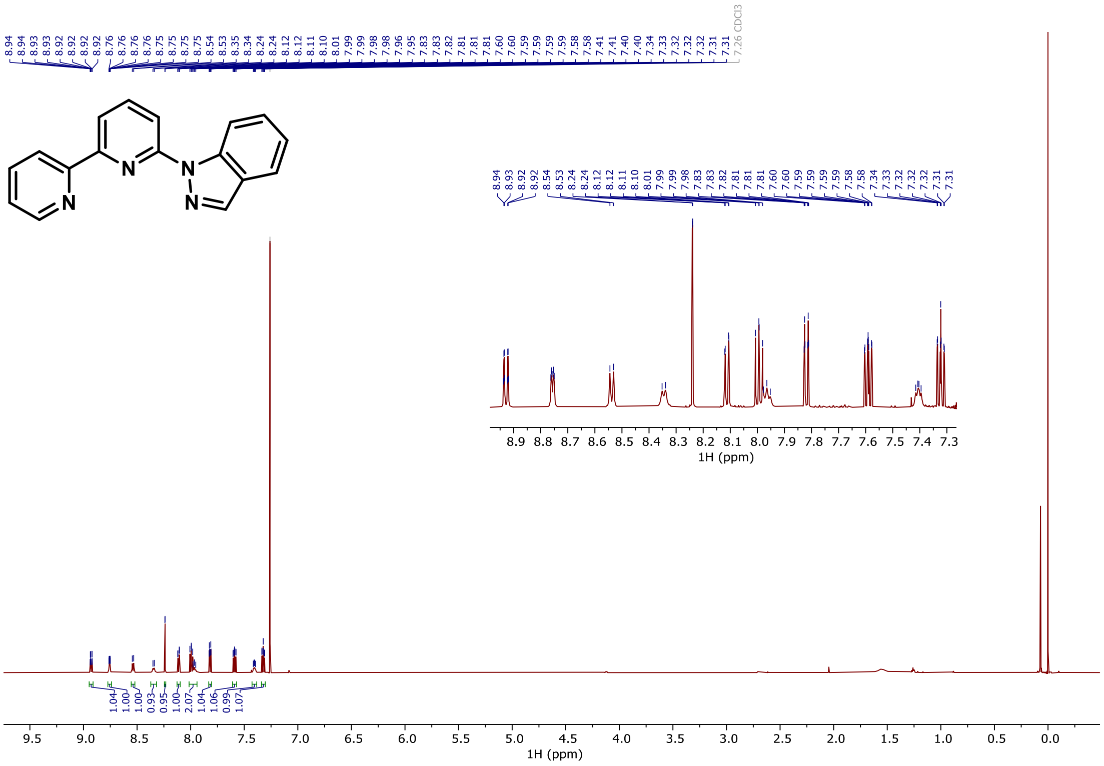

^{13}C NMR (151 MHz, CDCl3) of compound **L59.**

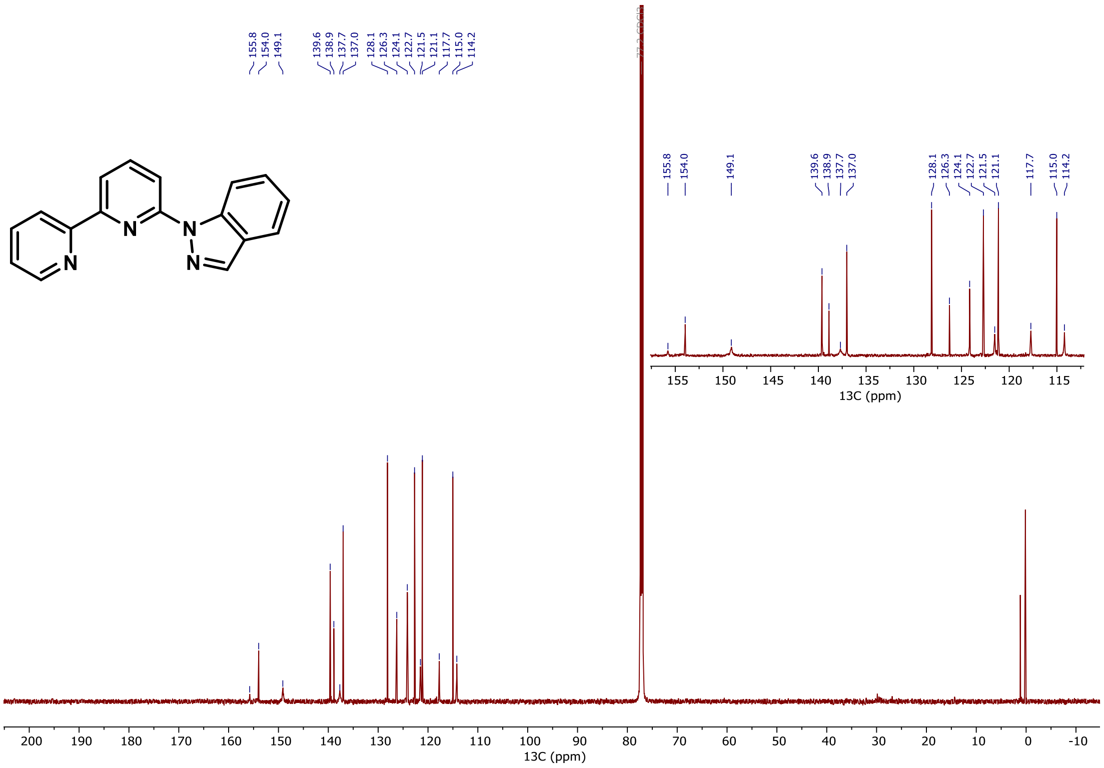

^{1}H NOESY ($CDCl_3$) of compound **L59.**

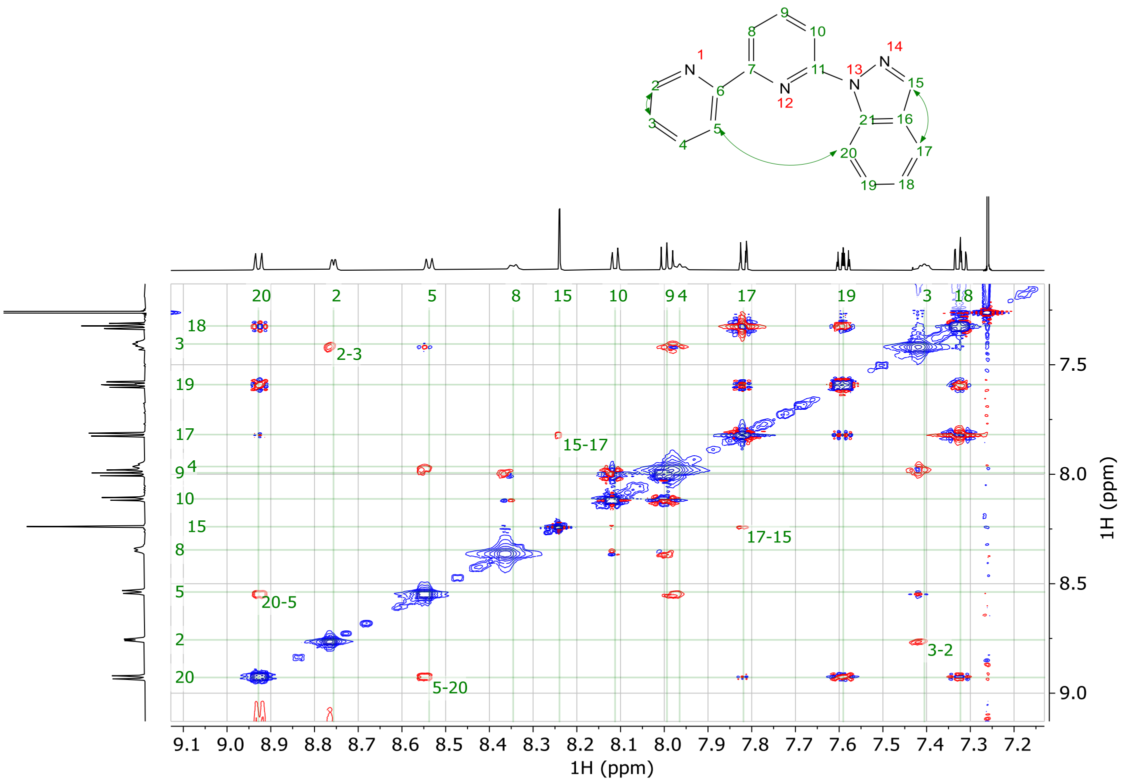

^{1}H NMR (400 MHz, CDCl$_3$) of compound **S17.**

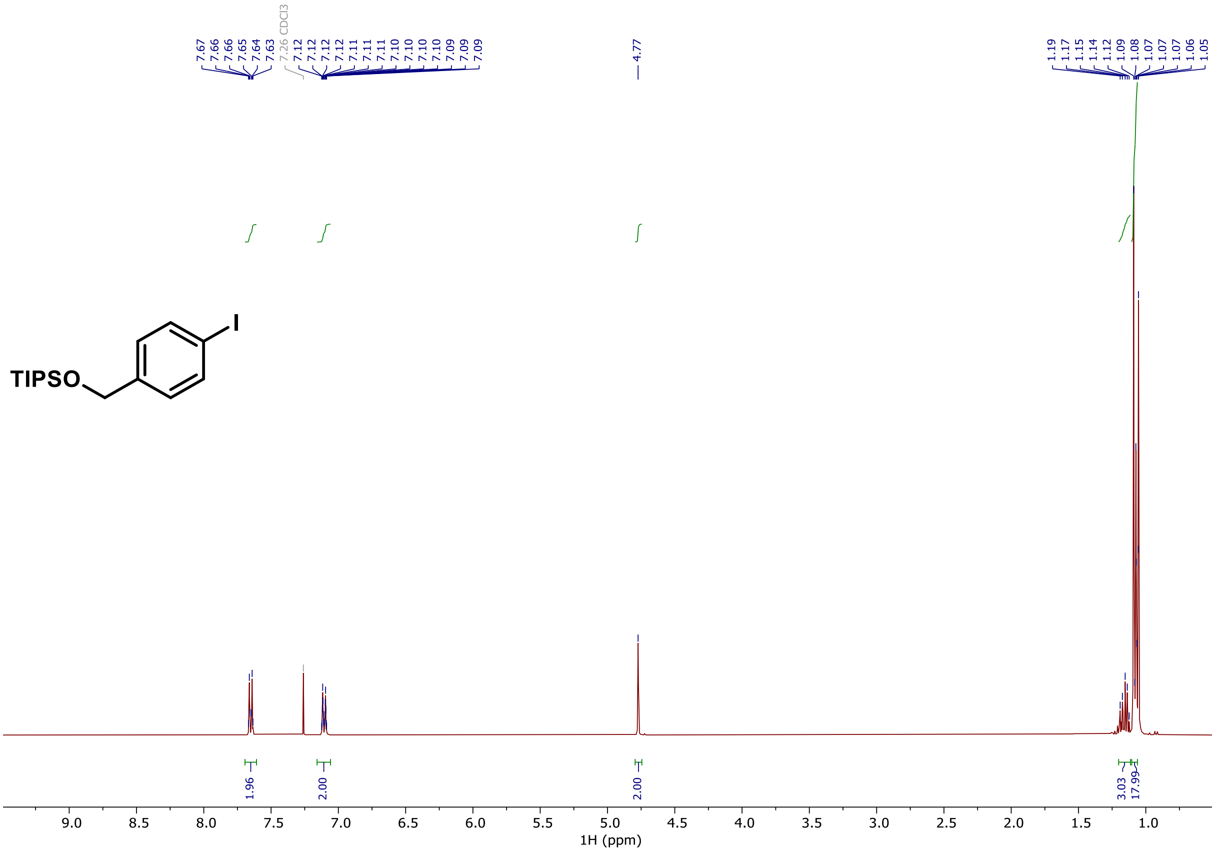

^{13}C NMR (101 MHz, CDCl$_3$) of compound **S17.**

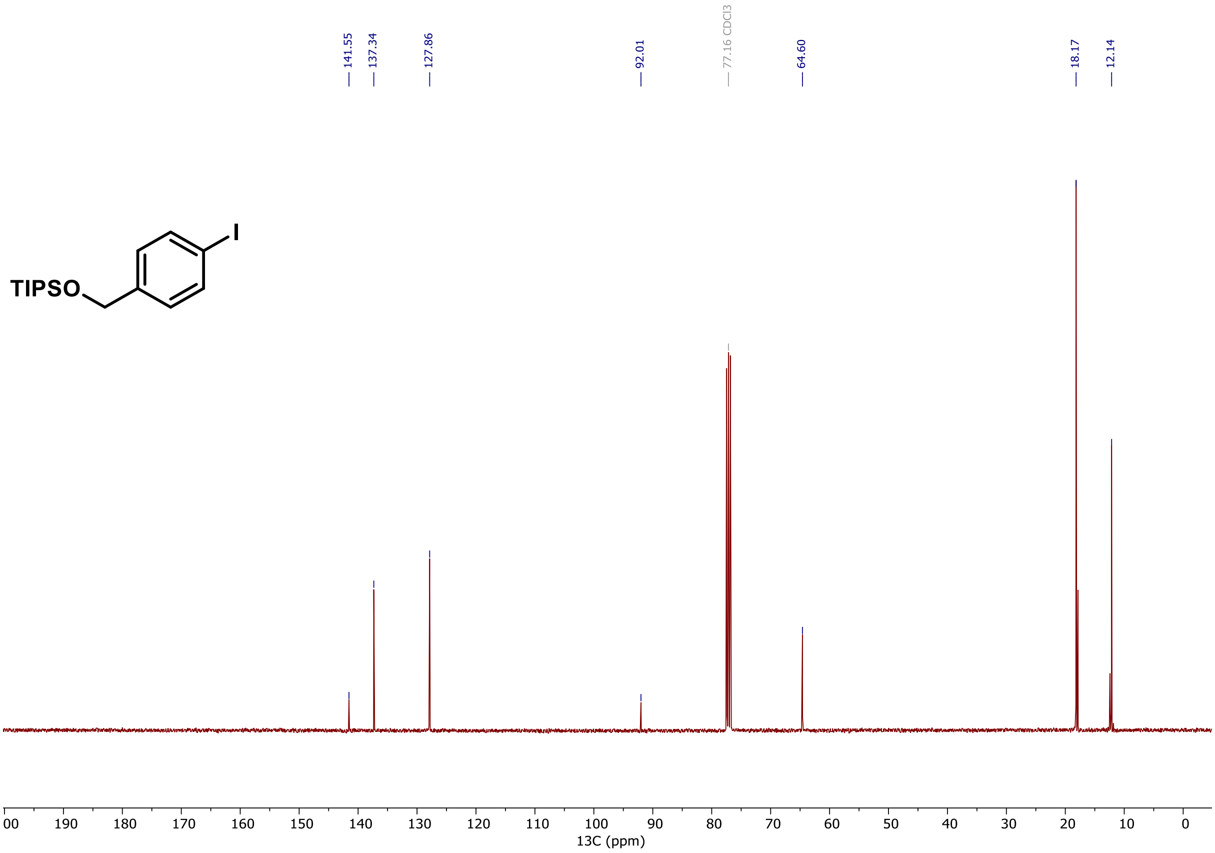

^{1}H NMR (400 MHz, CDCl₃) of compound S25.

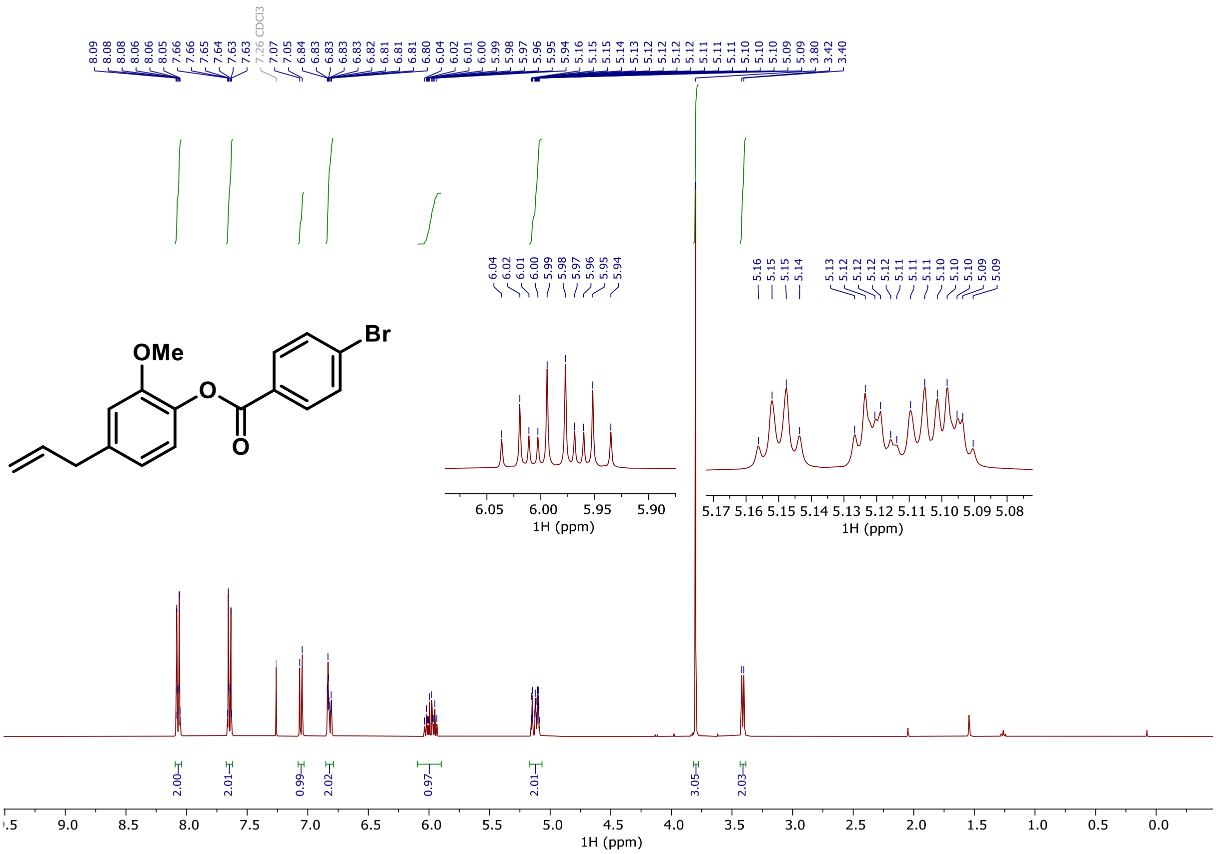

^{13}C NMR (101 MHz, CDCl₃) of compound S25.

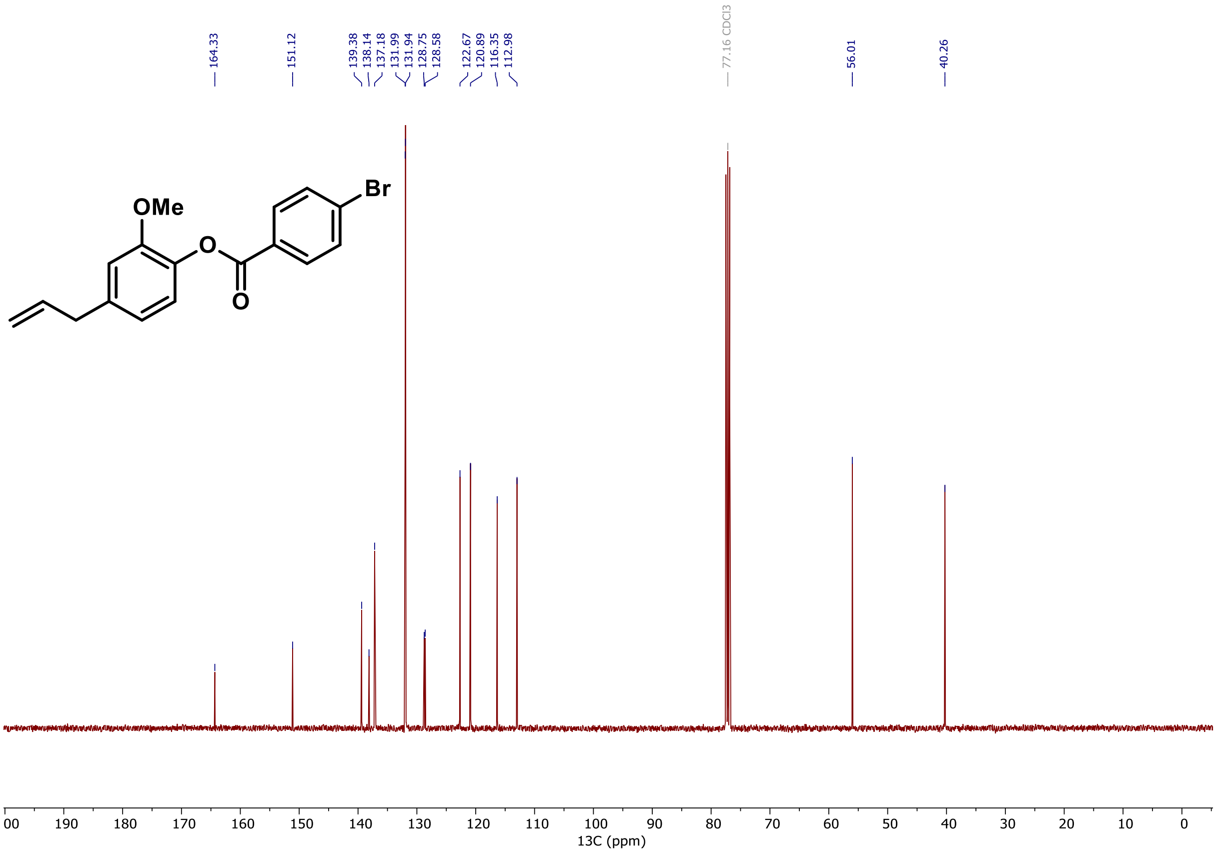

^{1}H NMR (400 MHz, CDCl$_3$) of compound **S32.**

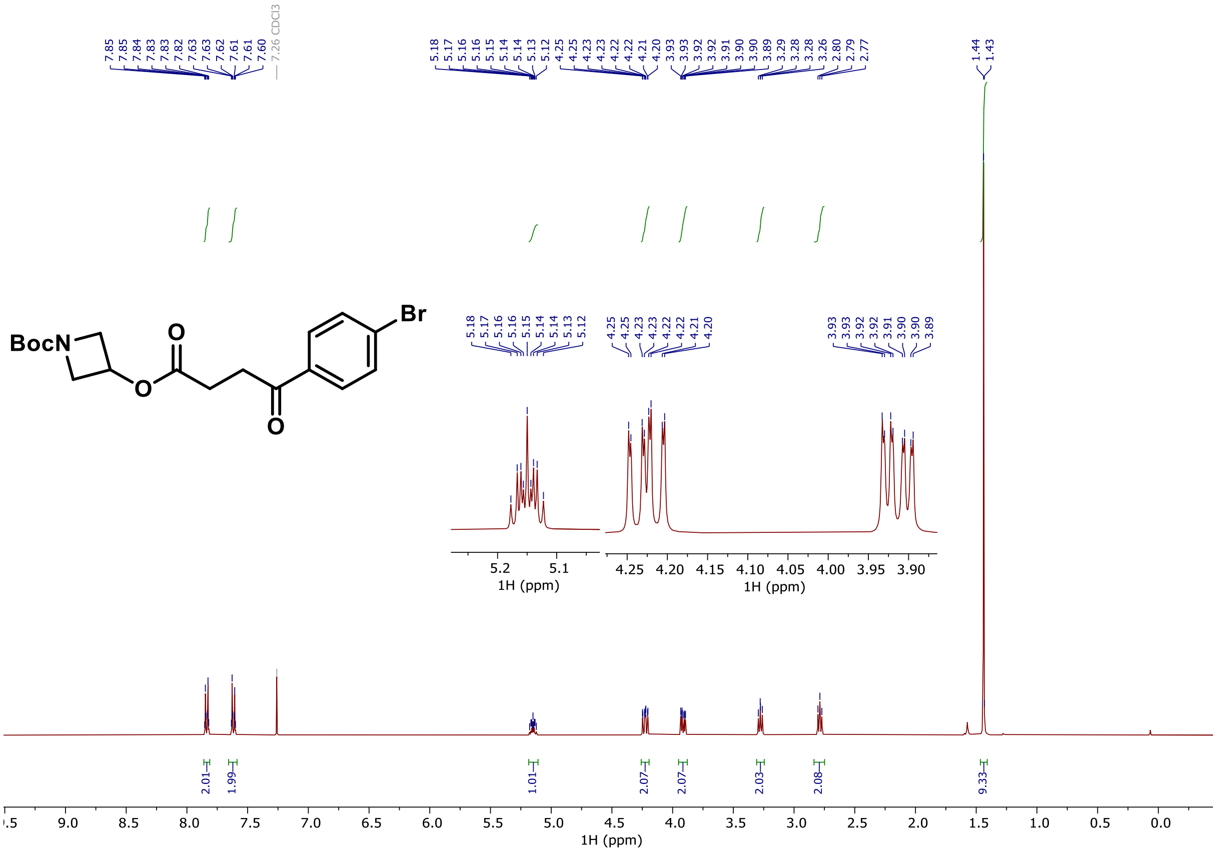

^{13}C NMR (101 MHz, CDCl$_3$) of compound **S32.**

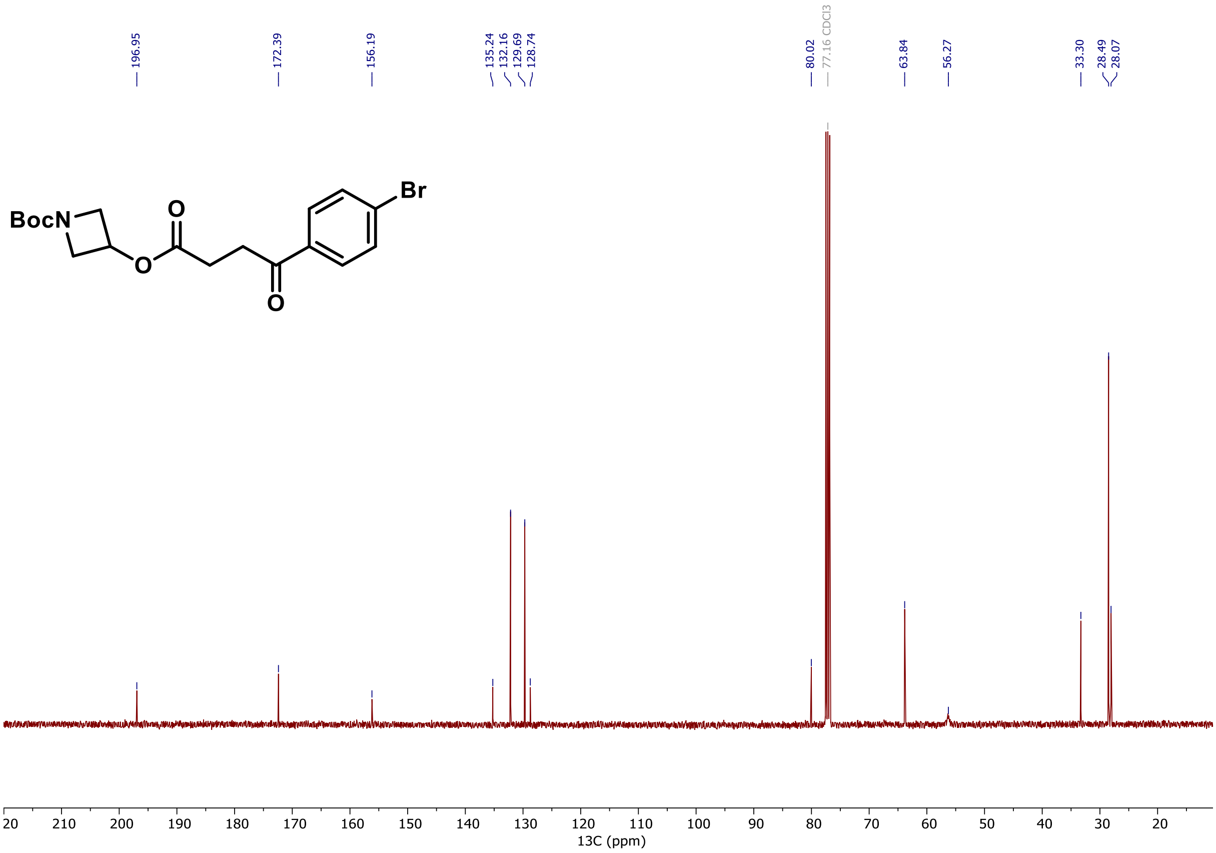

^{1}H NMR (300 MHz, CDCl3) of compound S34.

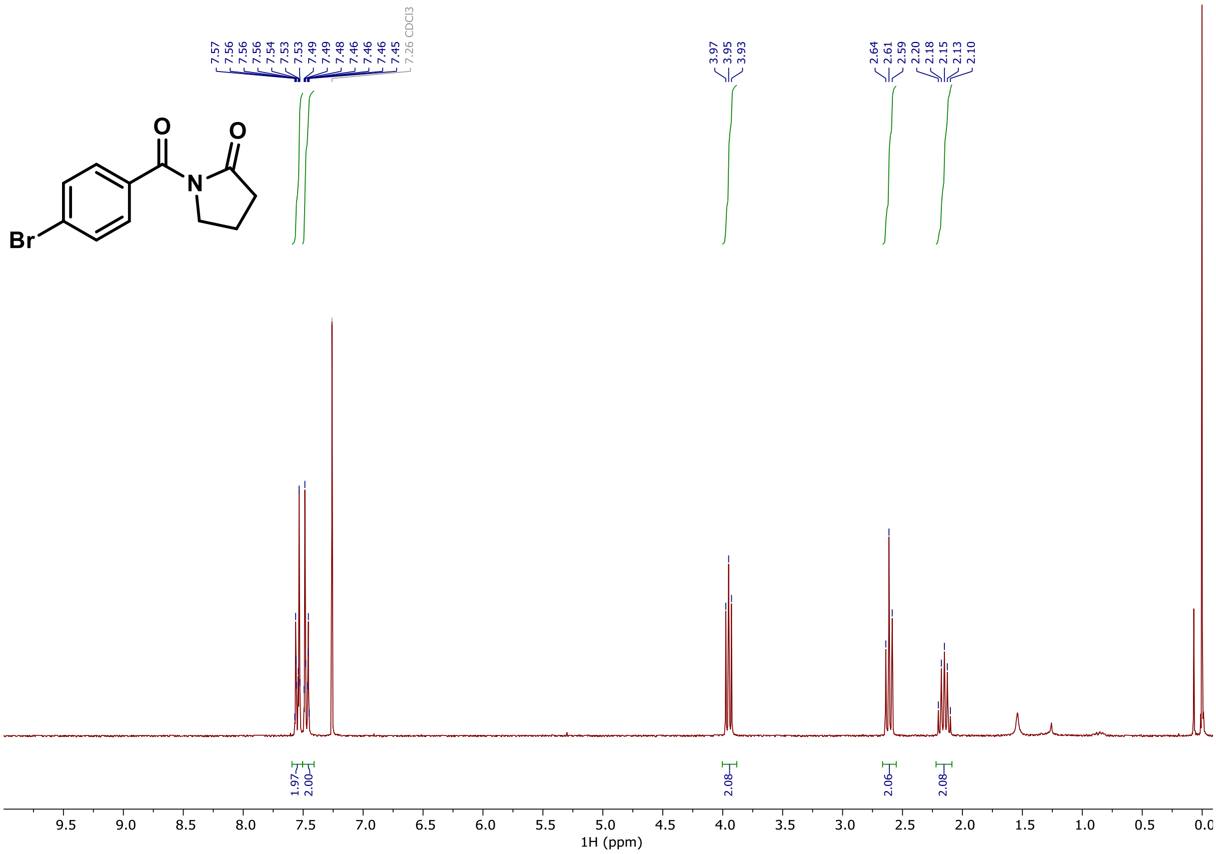

^{13}C NMR (75 MHz, CDCl3) of compound S34.

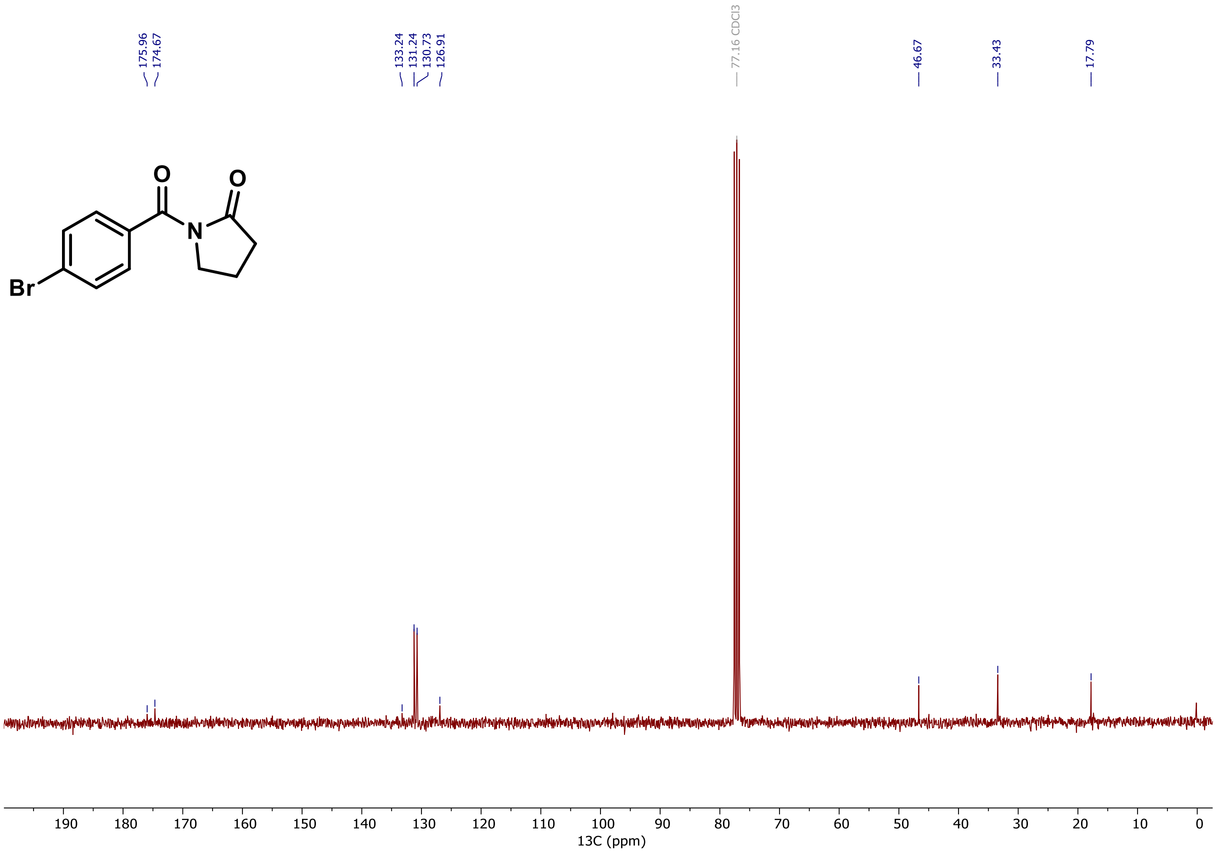

^{1}H NMR (400 MHz, CDCl3) of compound **S36.**

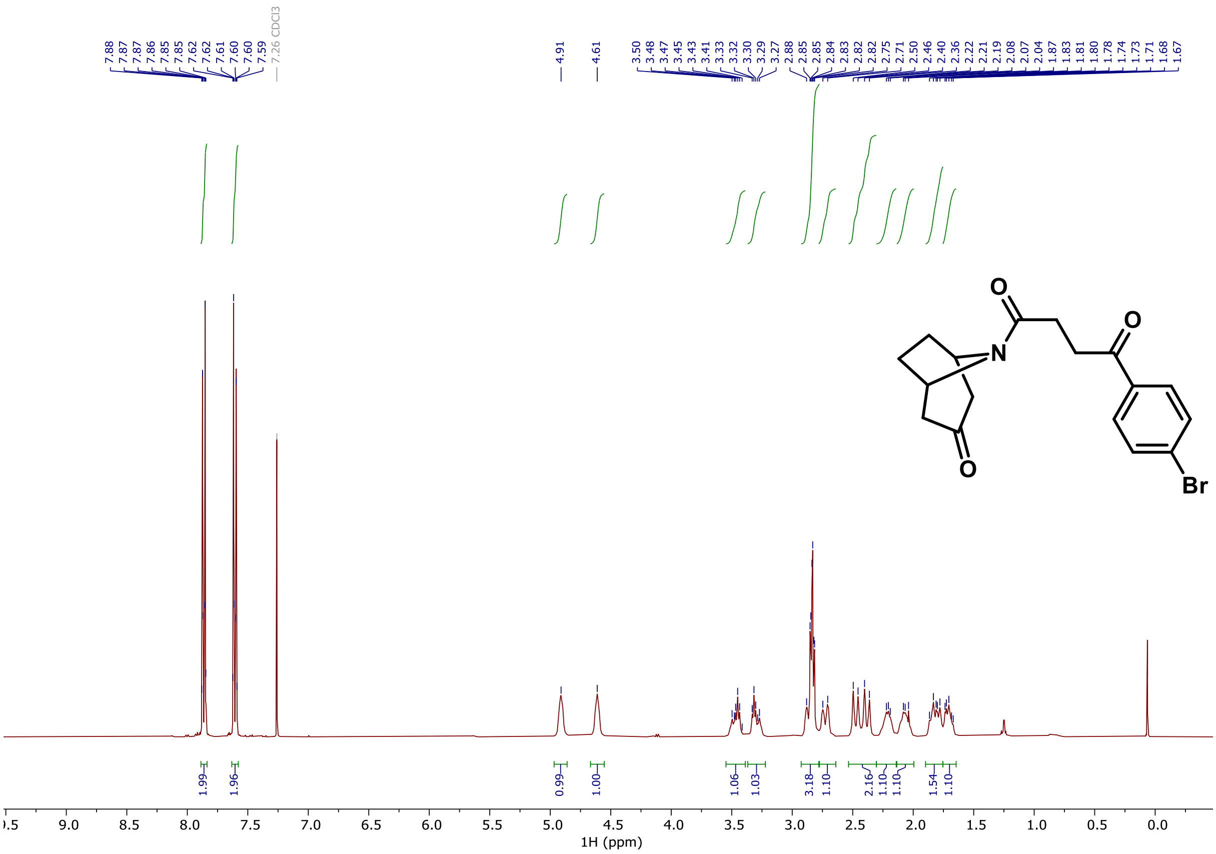

^{13}C NMR (101 MHz, CDCl3) of compound **S36.**

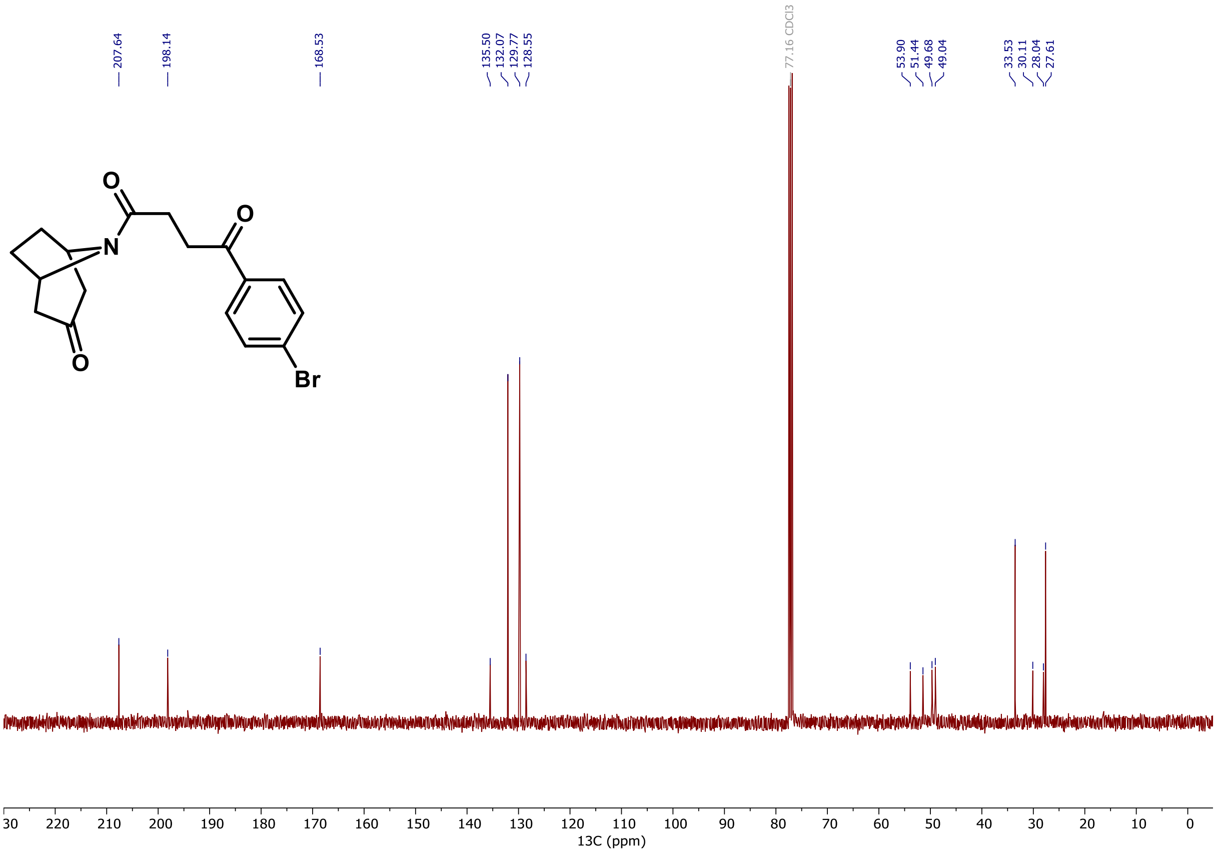

^{1}H NMR (300 MHz, CDCl₃) of compound **S38.**

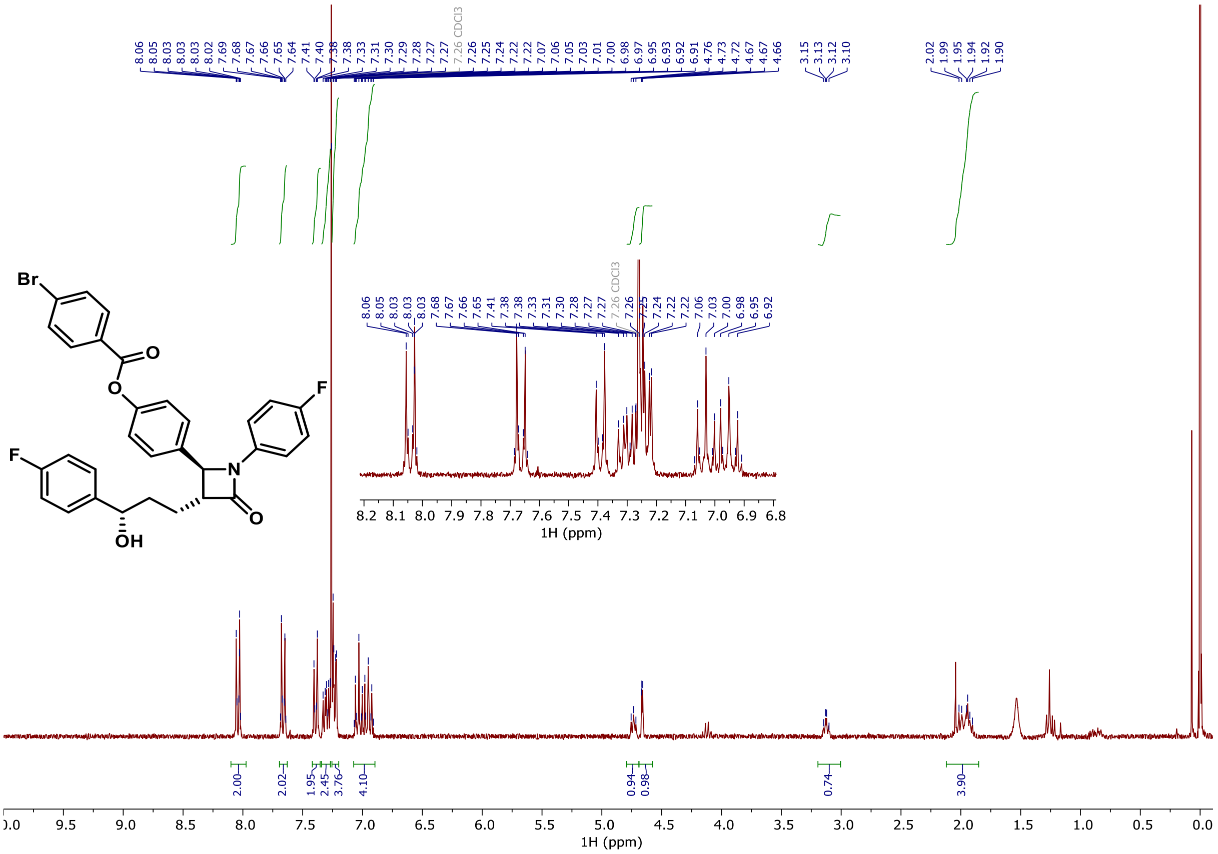

^{1}H NMR (400 MHz, CDCl3) of compound S40.

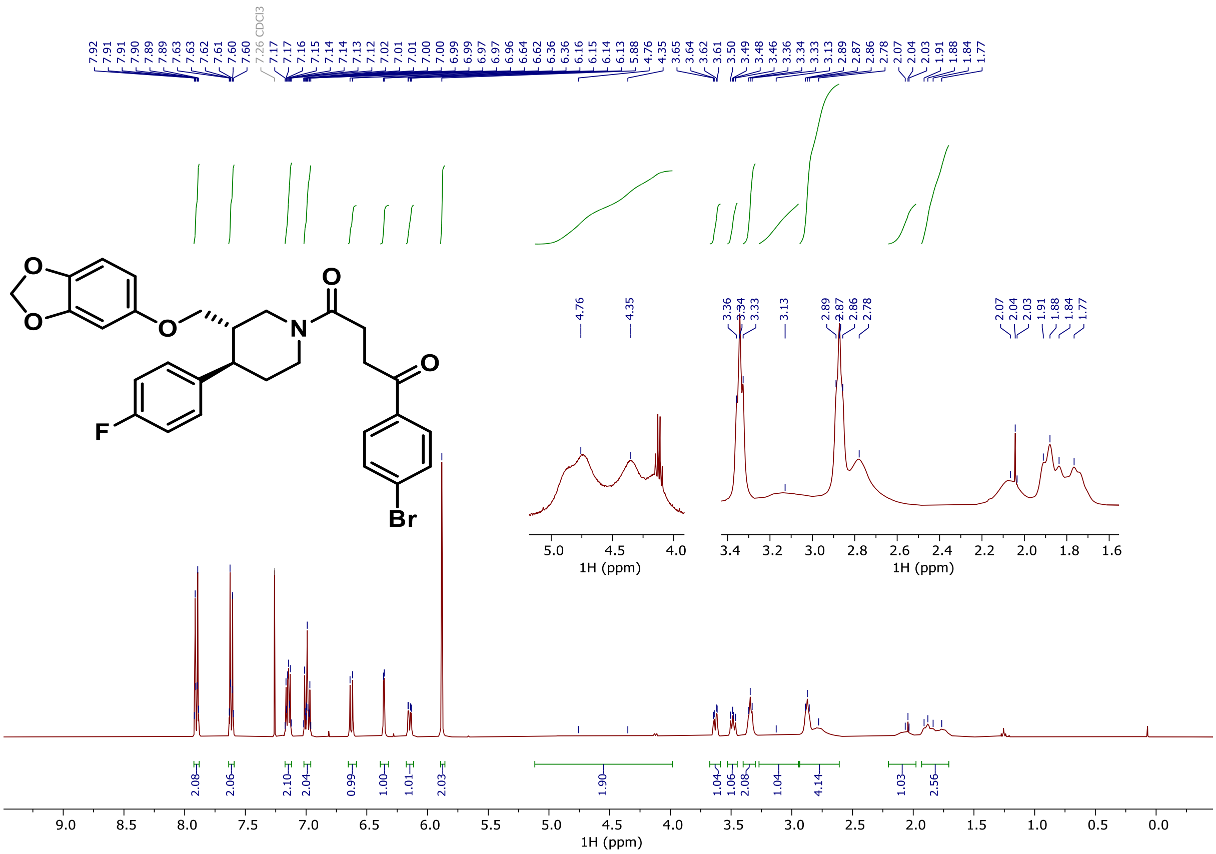

^{13}C NMR (101 MHz, CDCl3) of compound S40.

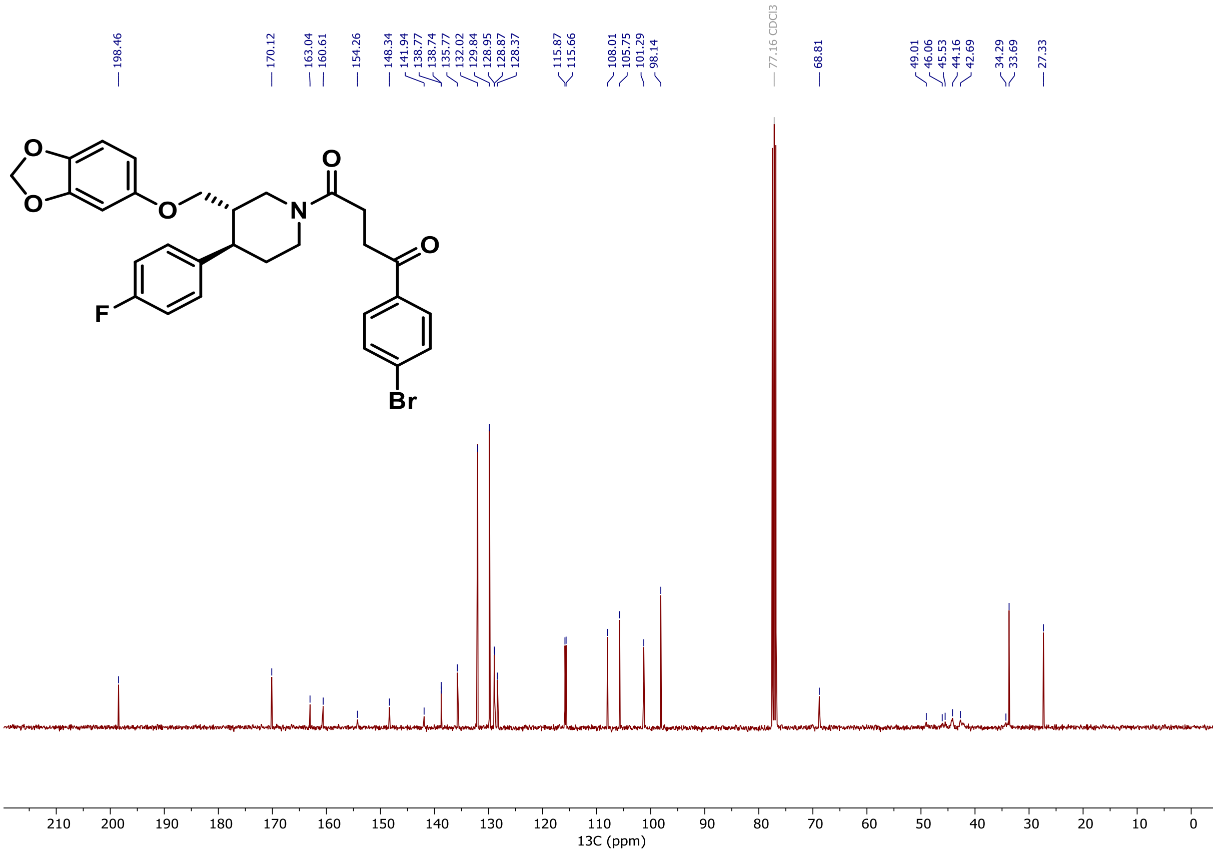

^{1}H NMR (600 MHz, THF-*d_8*) of compound **S47.** [(*t*Bu-terpy)NiPh]I.

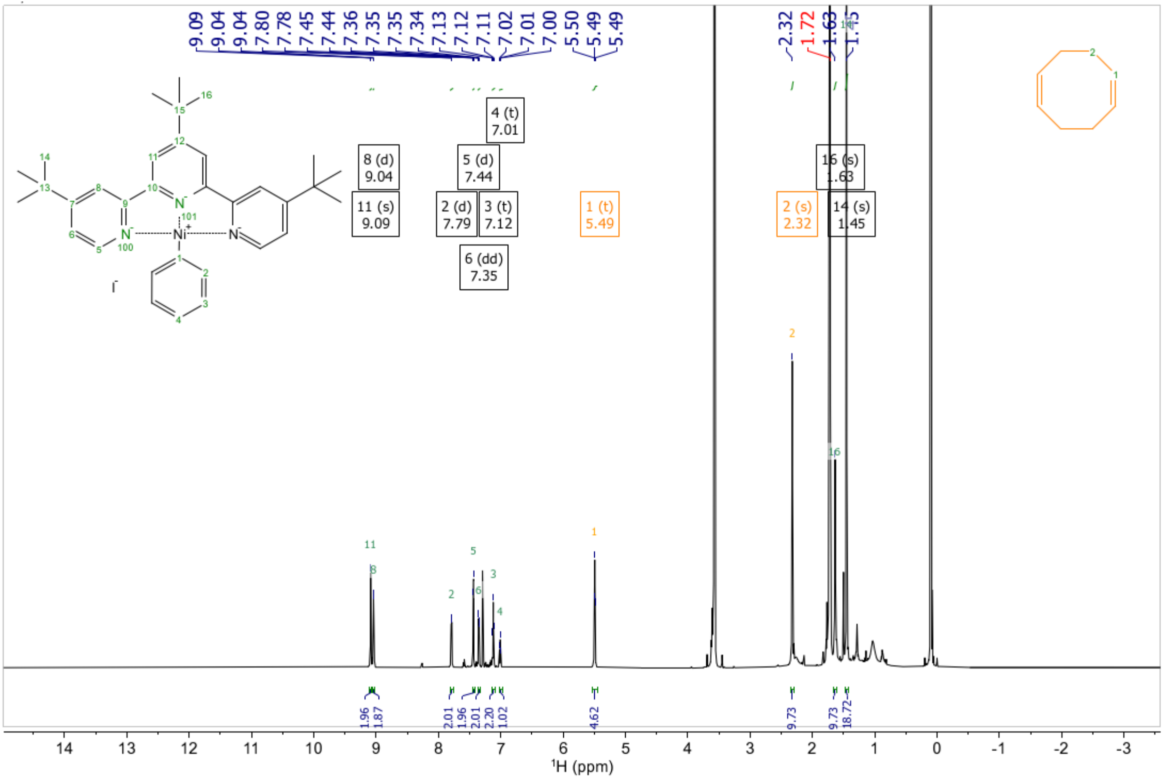

^{13}C NMR (151 MHz, THF-*d_8*) of compound **S47.**

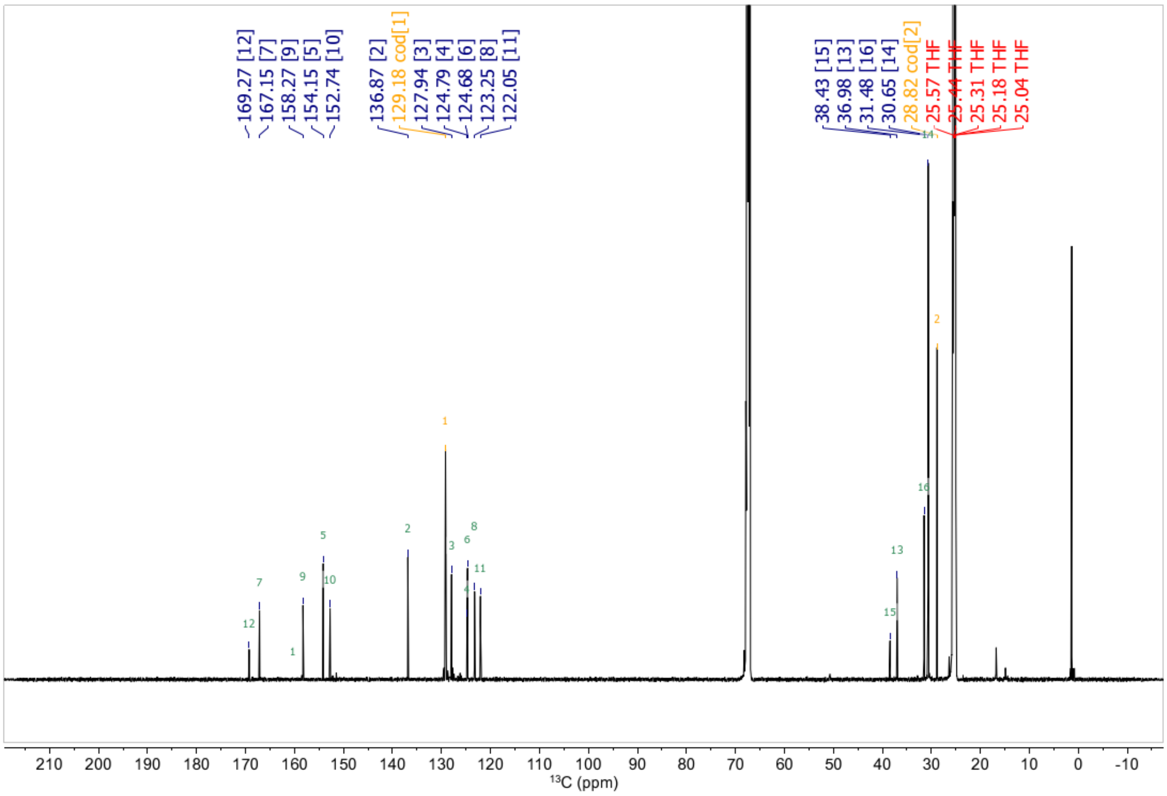

^{1}H-^{13}C HSQC (THF-*d_8*) of compound S47.

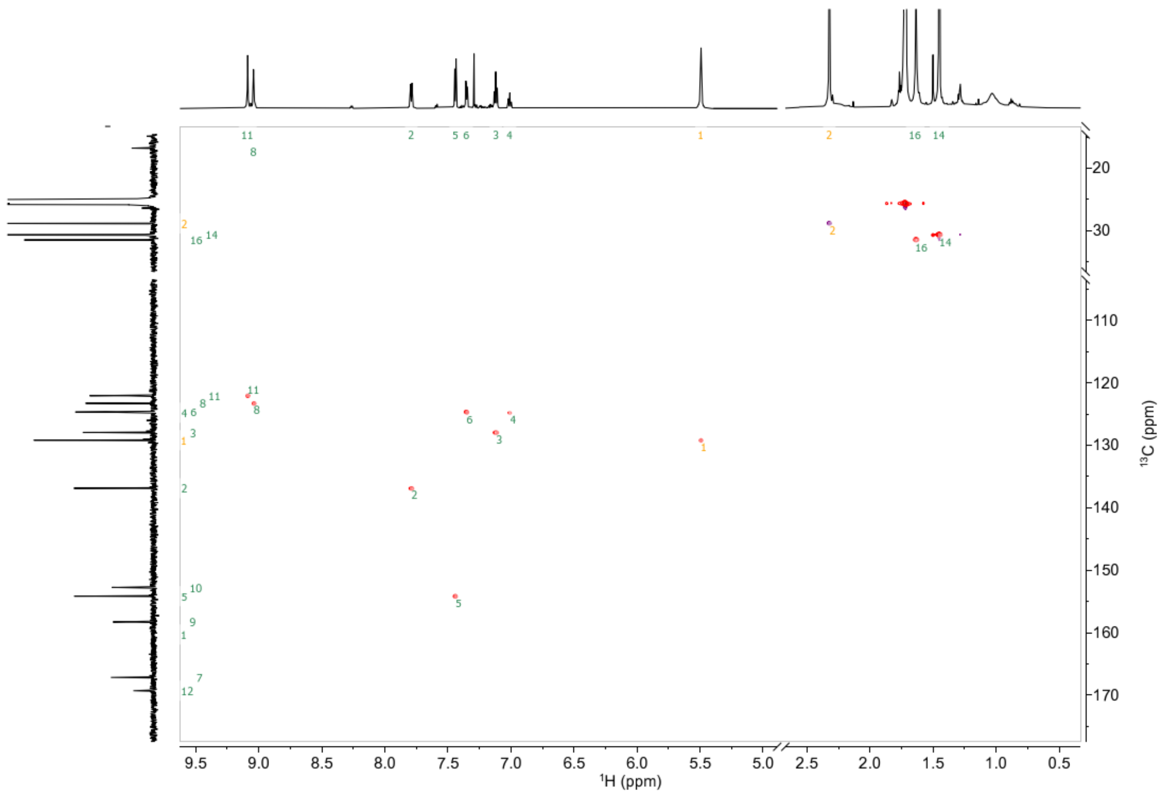

^{1}H-^{13}C HMBC (THF-*d_8*) of compound S47.

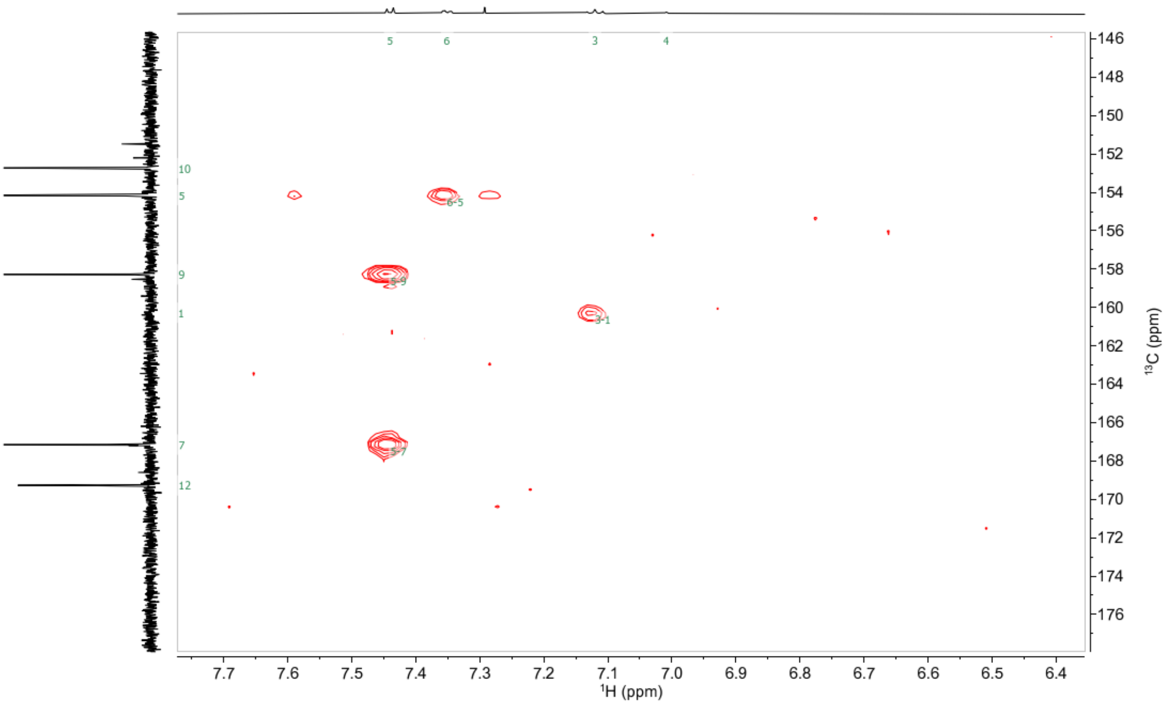

^{1}H-^{1}H COSY (THF-d_8) of compound **S47.**

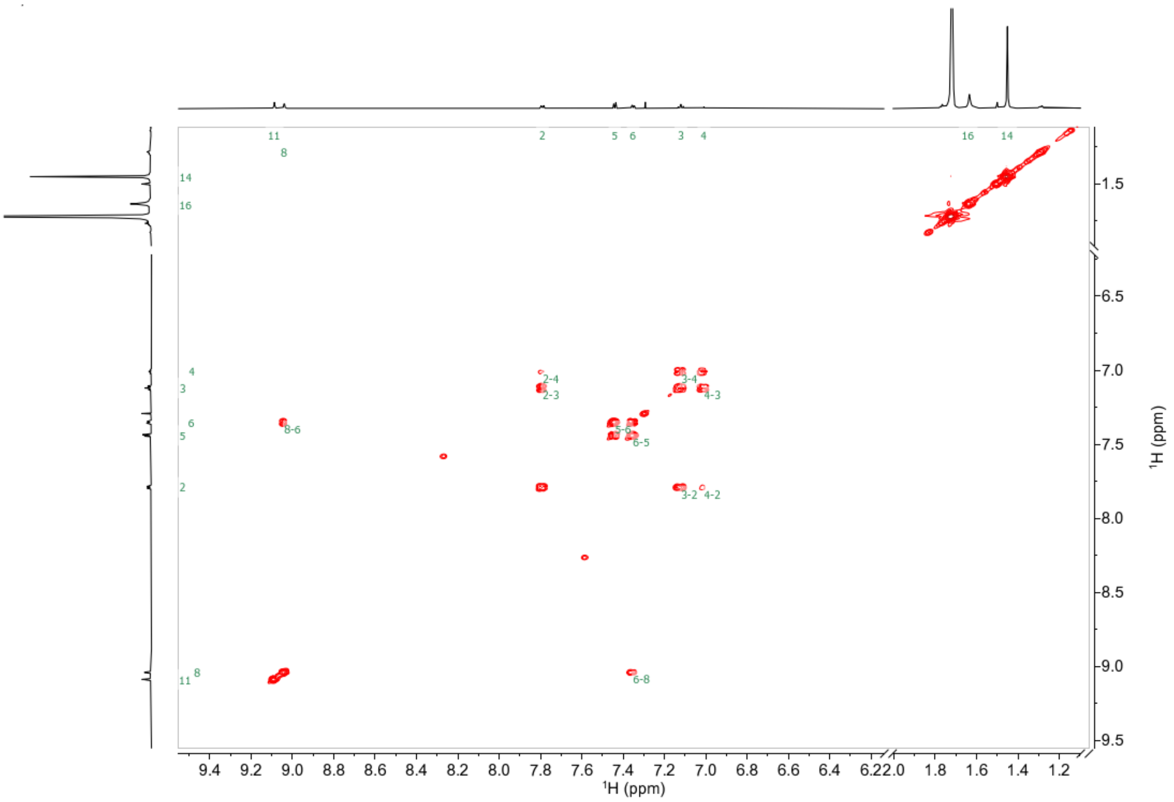

^{1}H-^{1}H NOESY (THF-d_8) of compound **S47.**

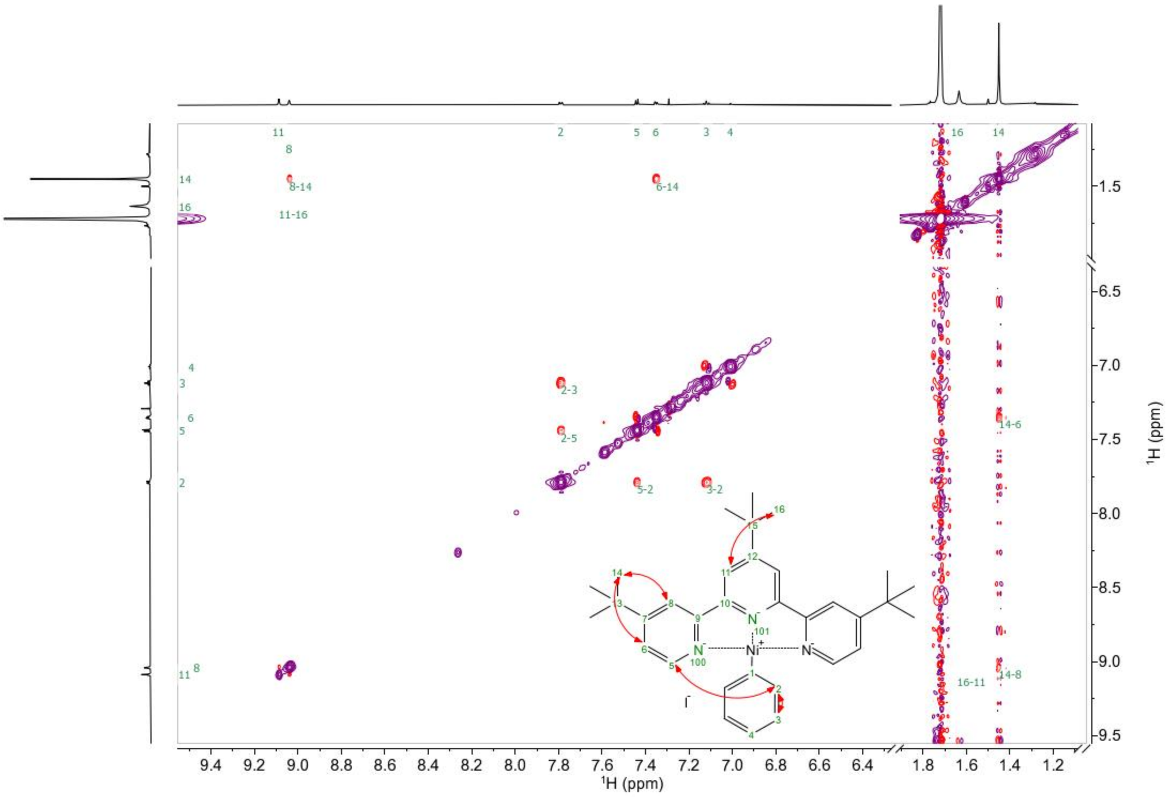

^{1}H-^{1}H ROESY (THF-*d_8*) of compound **S47.**

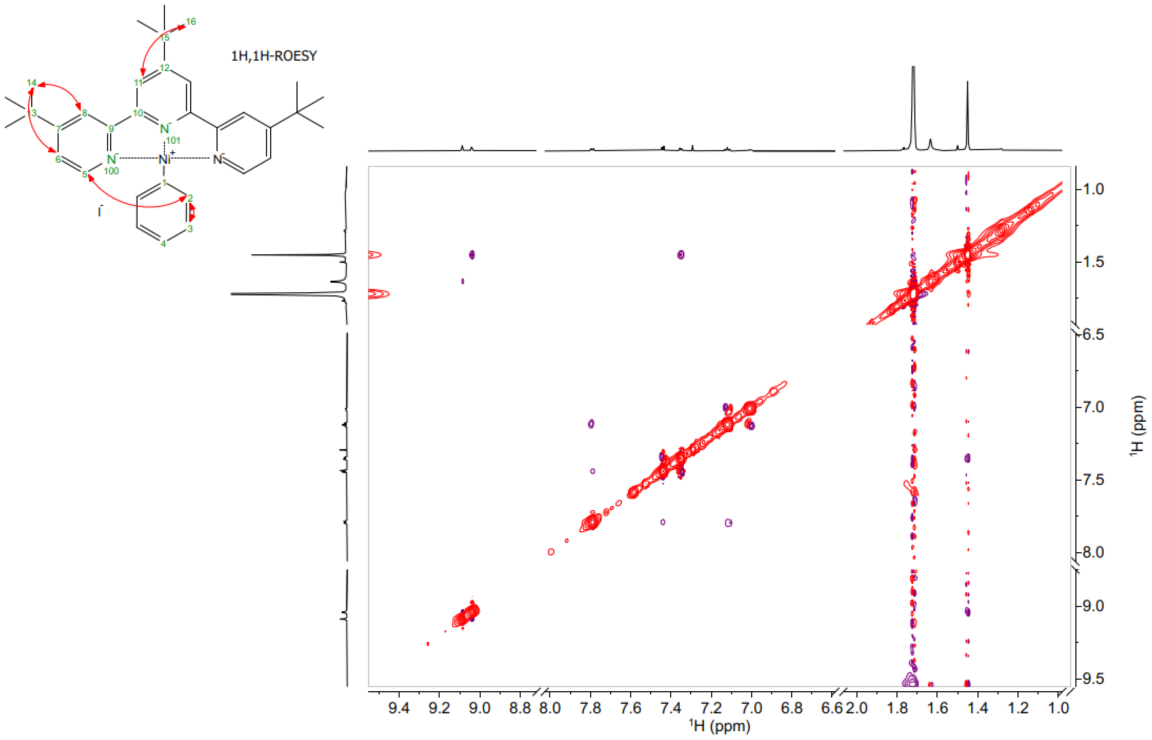

^{1}H-^{15}N HMBC (THF-*d_8*) of compound **S47.**

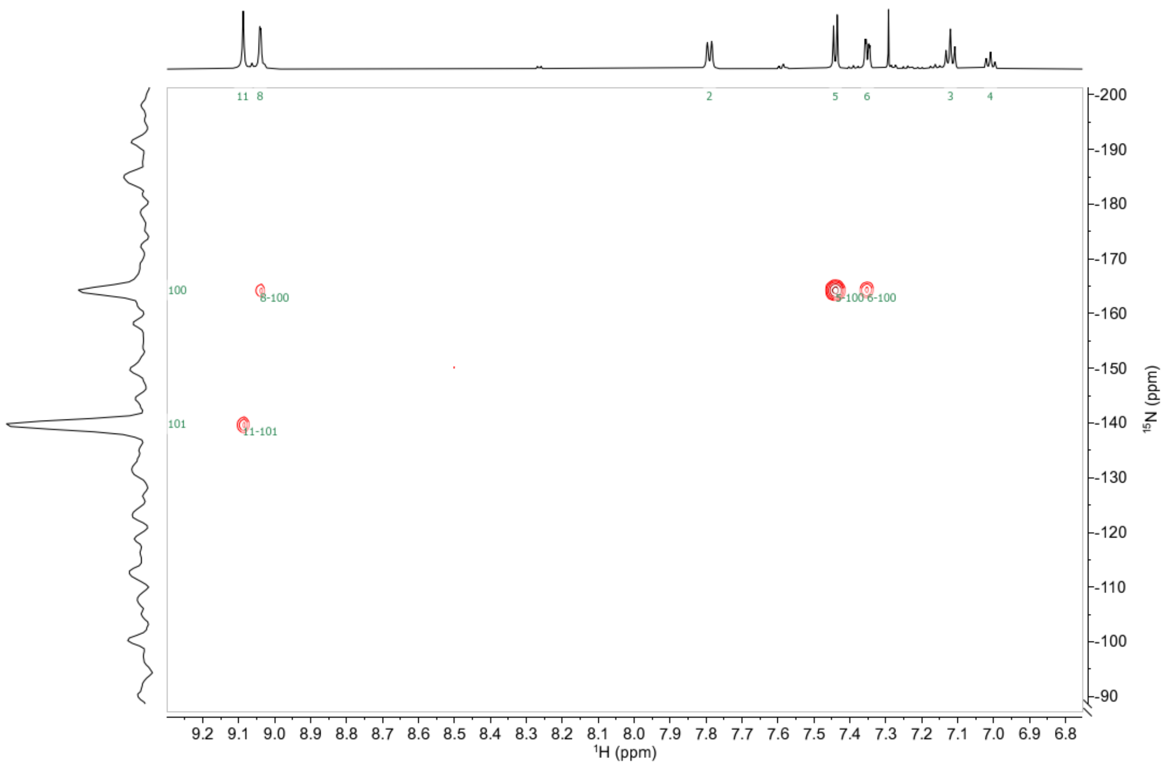

^{1}H NMR (400 MHz, CDCl$_3$) of compound **14.**

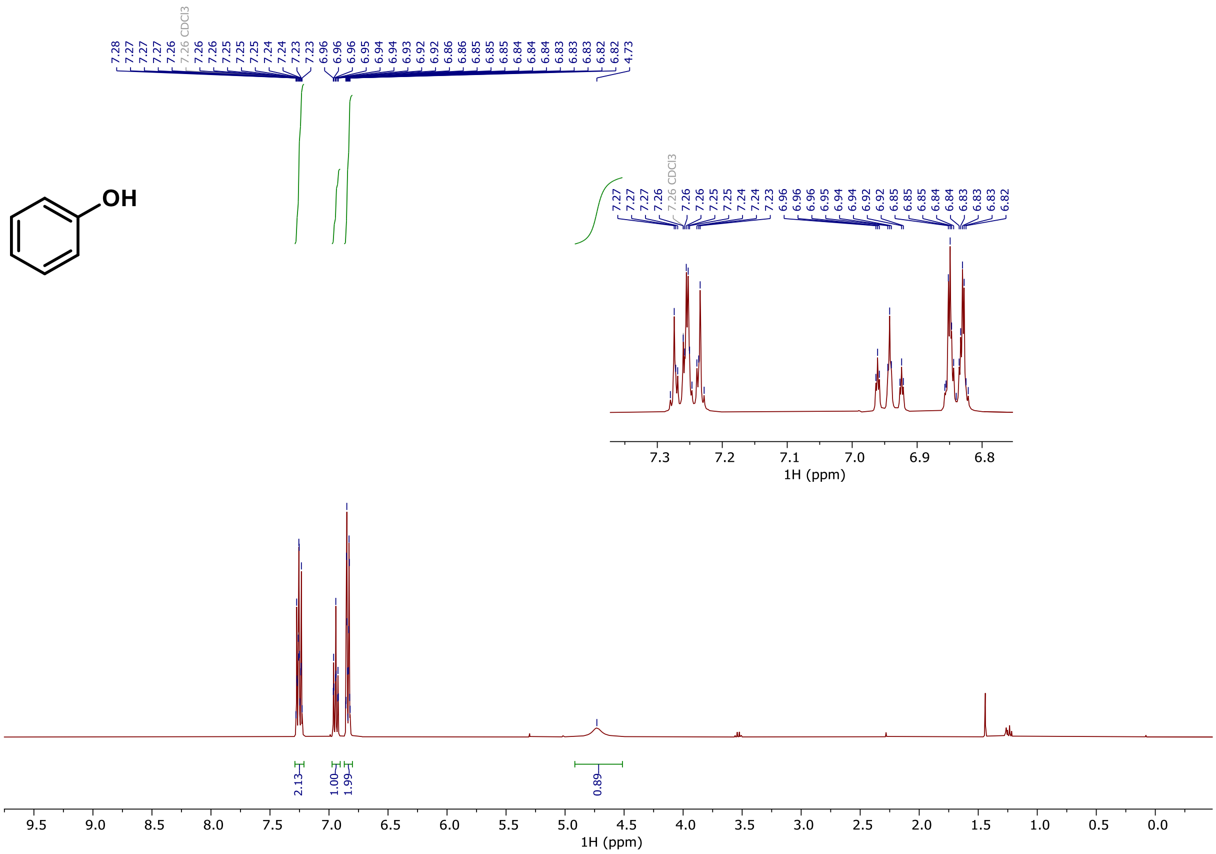

^{13}C NMR (101 MHz, CDCl$_3$) of compound **14.**

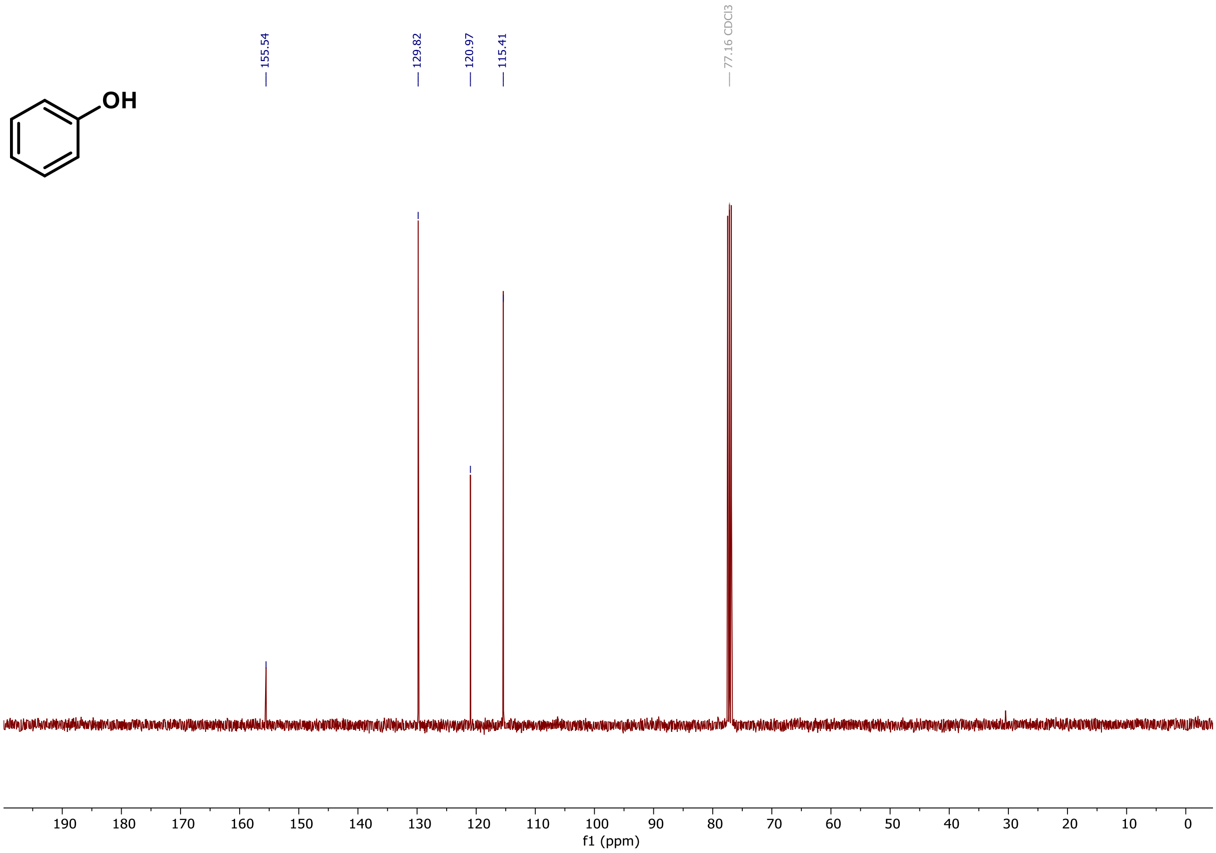

^{1}H NMR (400 MHz, CDCl₃) of compound **9.**

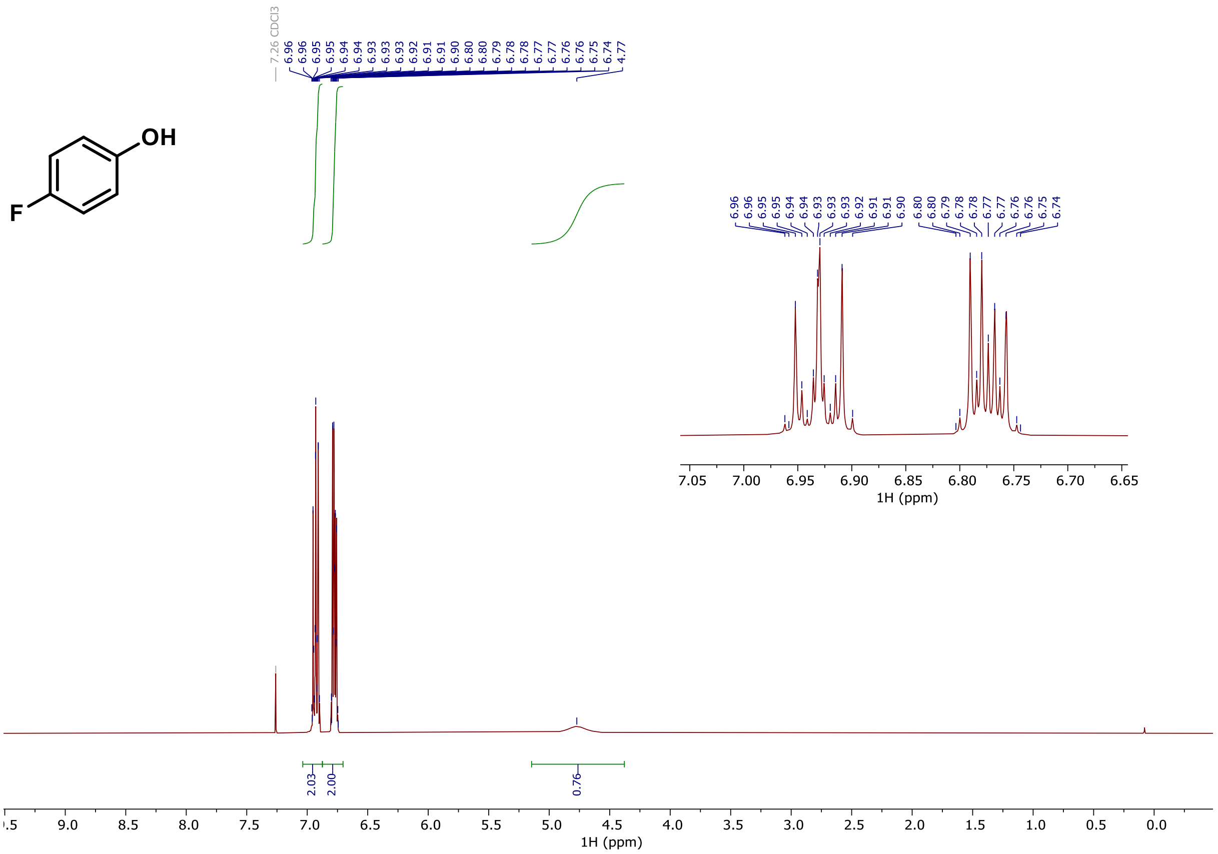

^{13}C NMR (101 MHz, CDCl₃) of compound **9.**

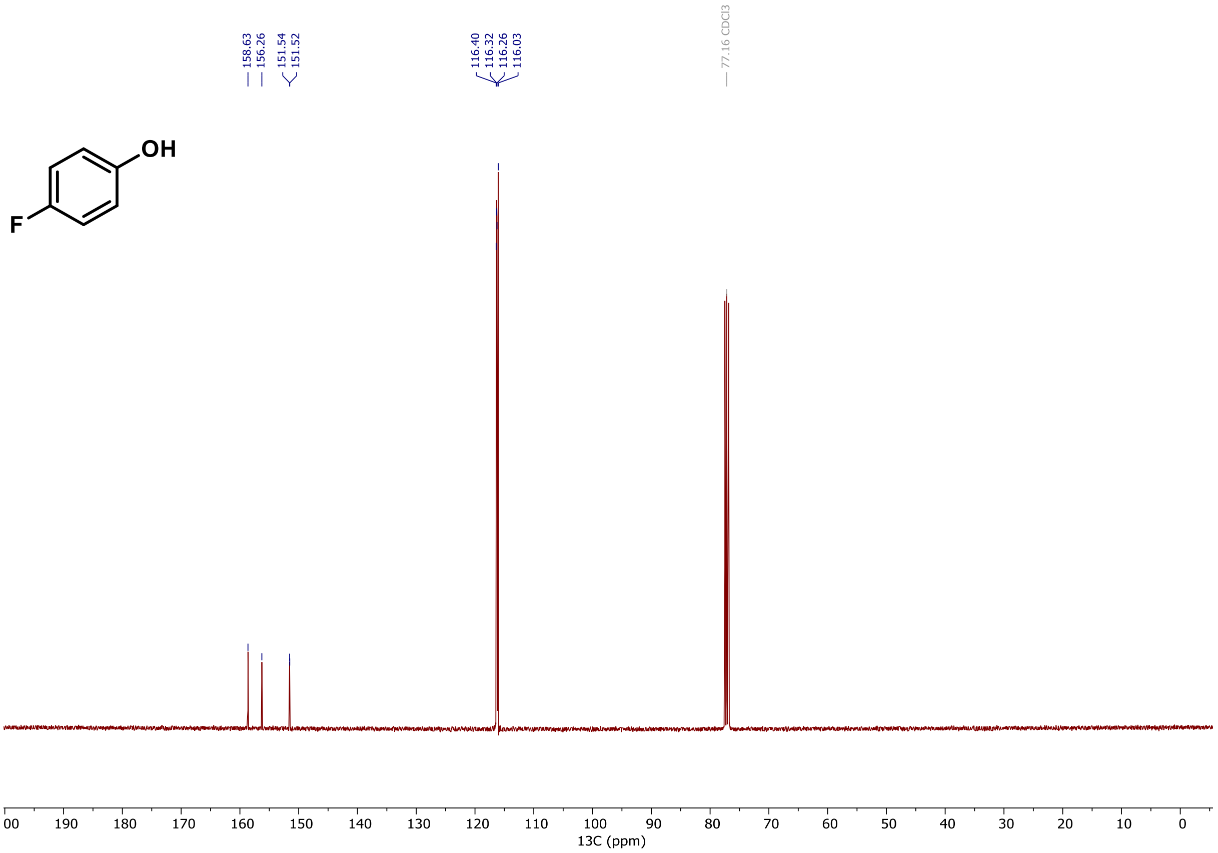

^{1}H NMR (400 MHz, $CDCl_3$) of compound **15.**

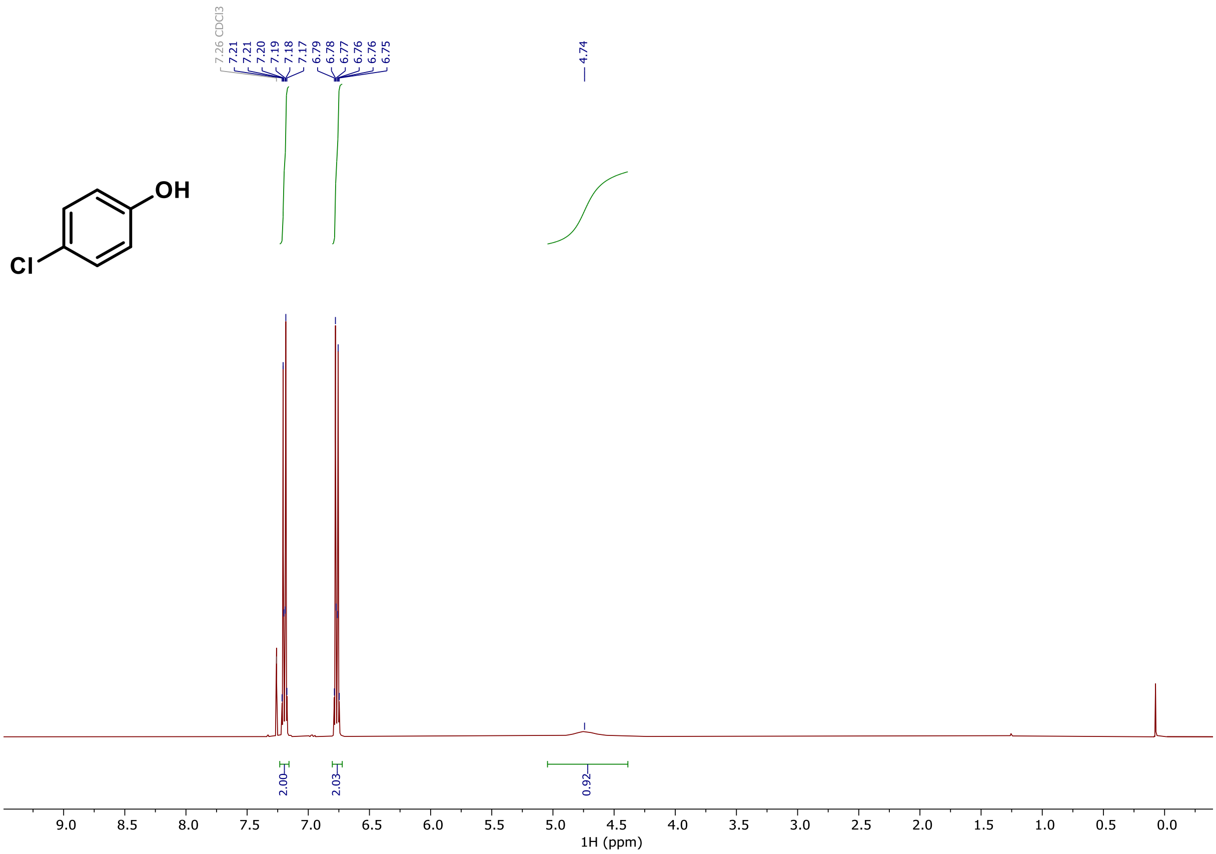

^{13}C NMR (101 MHz, $CDCl_3$) of compound **15.**

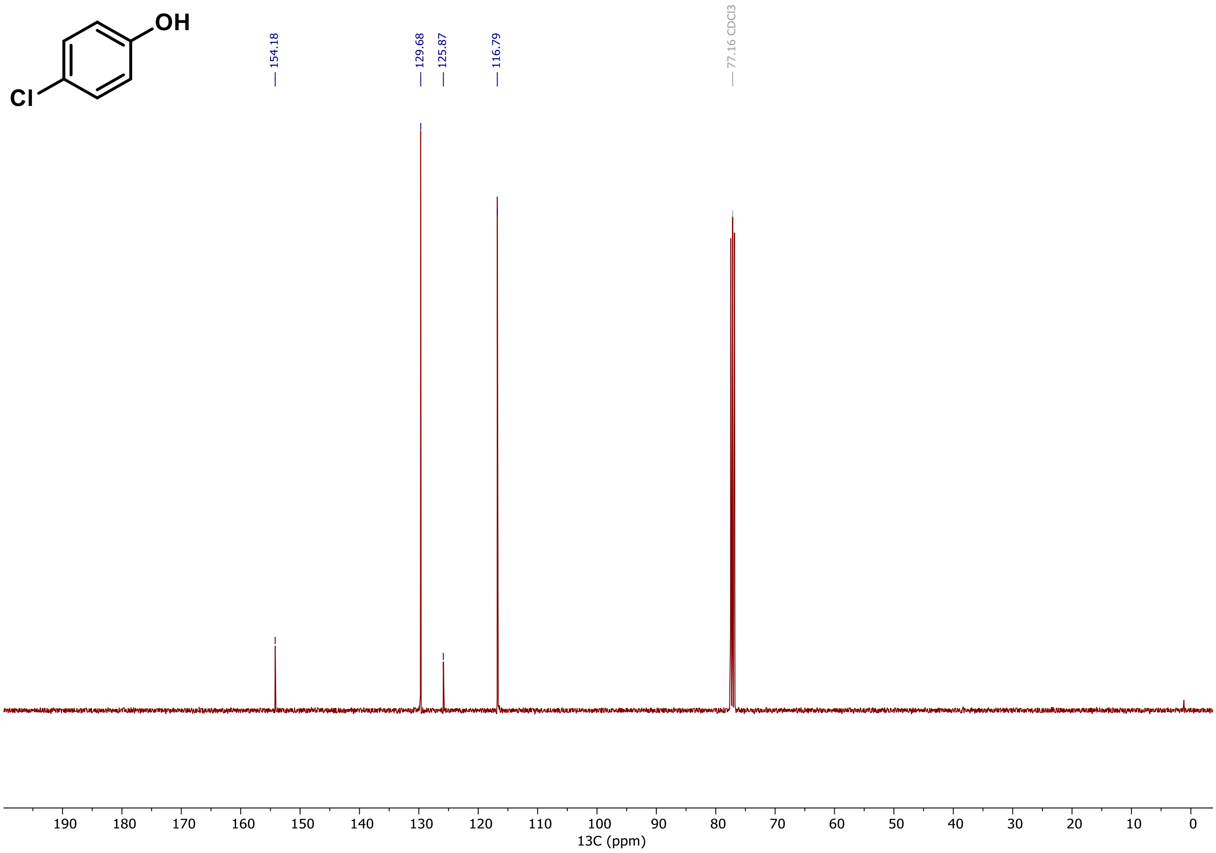

^{1}H NMR (300 MHz, $CDCl_3$) of compound **16.**

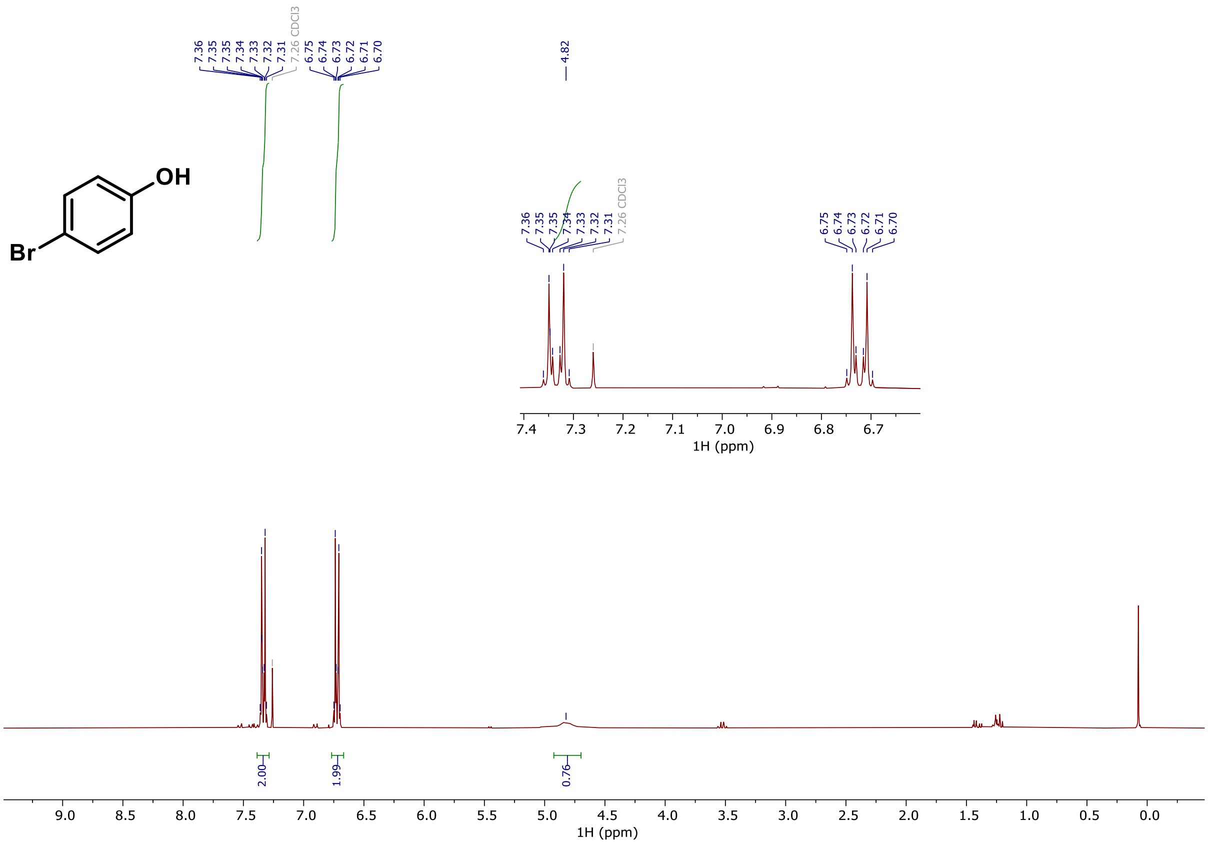

^{13}C NMR (75 MHz, $CDCl_3$) of compound **16.**

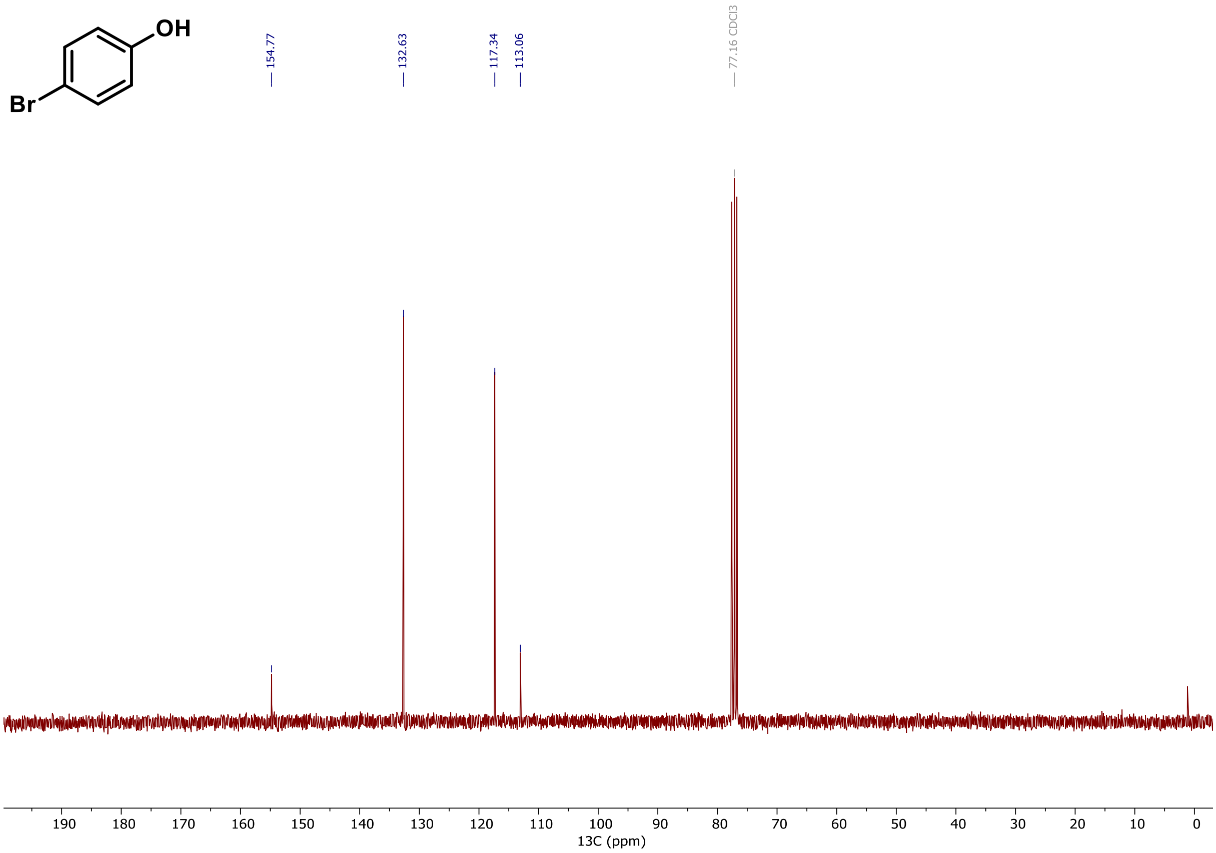

^{1}H NMR (400 MHz, CDCl$_3$) of compound **17.**

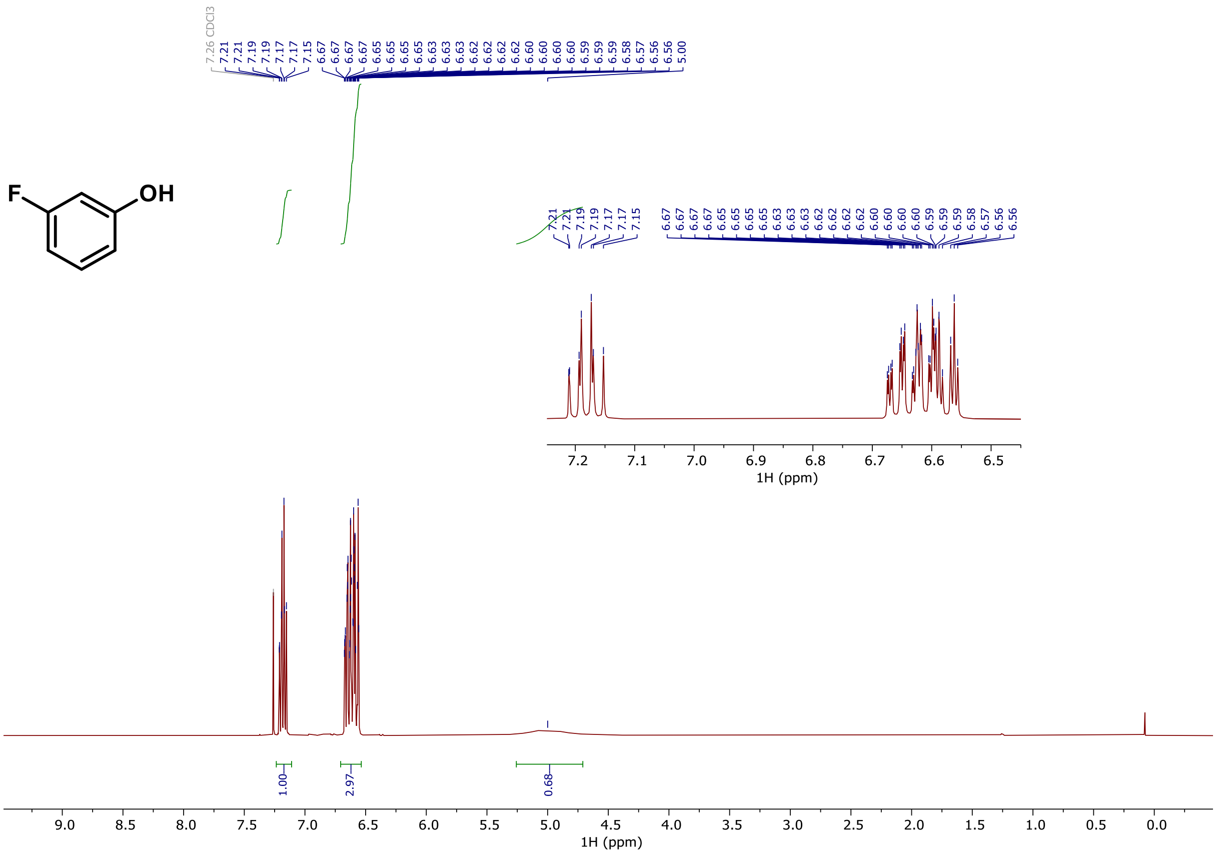

^{13}C NMR (101 MHz, CDCl$_3$) of compound **17.**

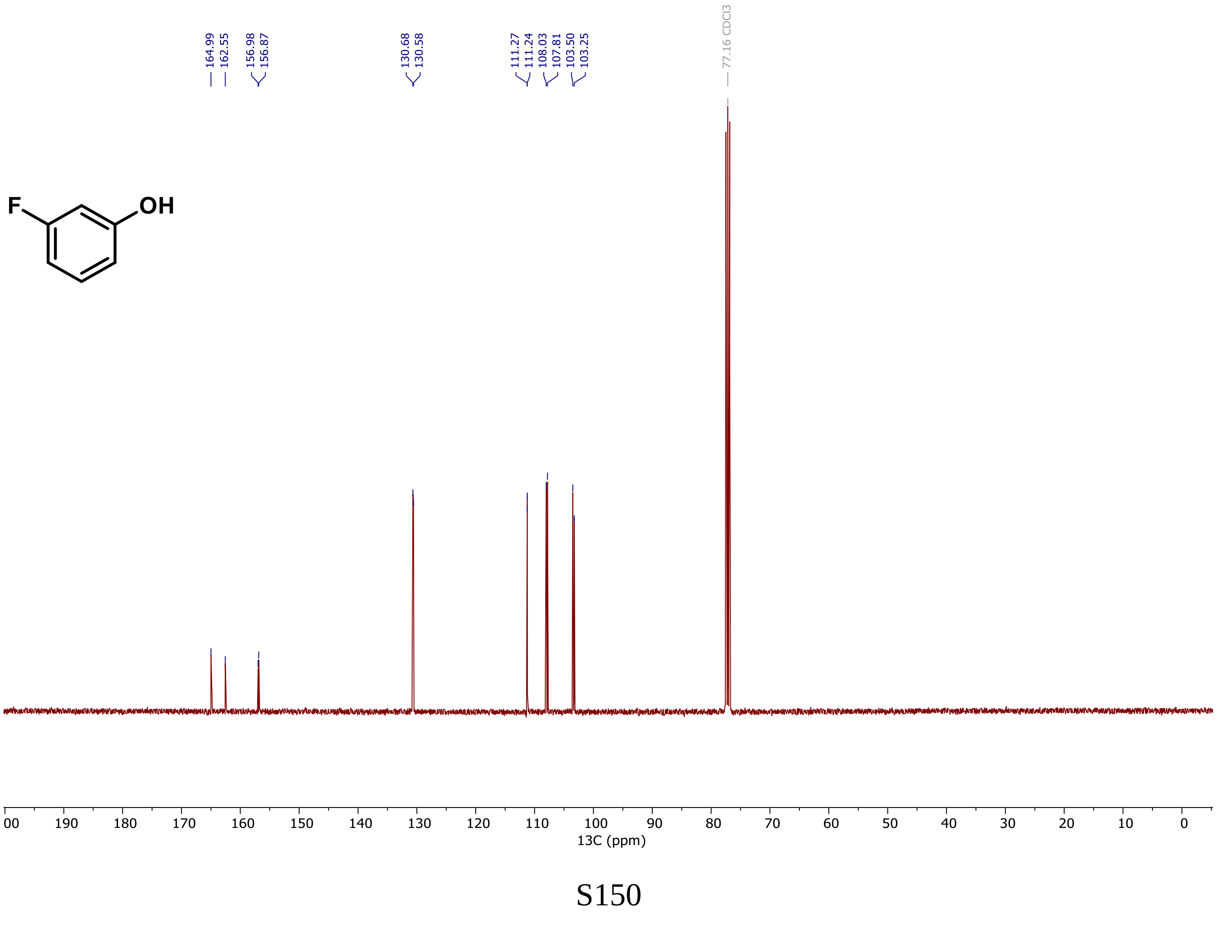

^{1}H NMR (400 MHz, CDCl$_3$) of compound **18.**

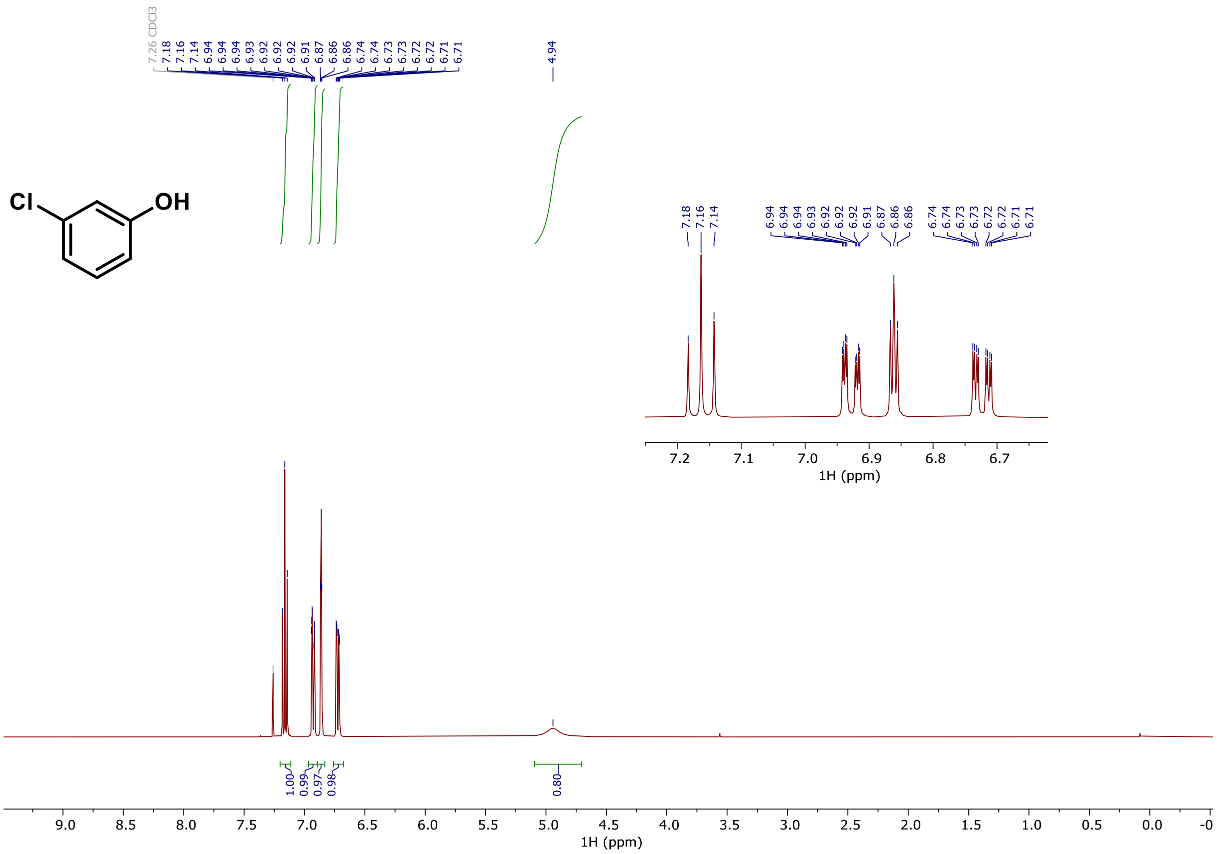

^{13}C NMR (101 MHz, CDCl$_3$) of compound **18.**

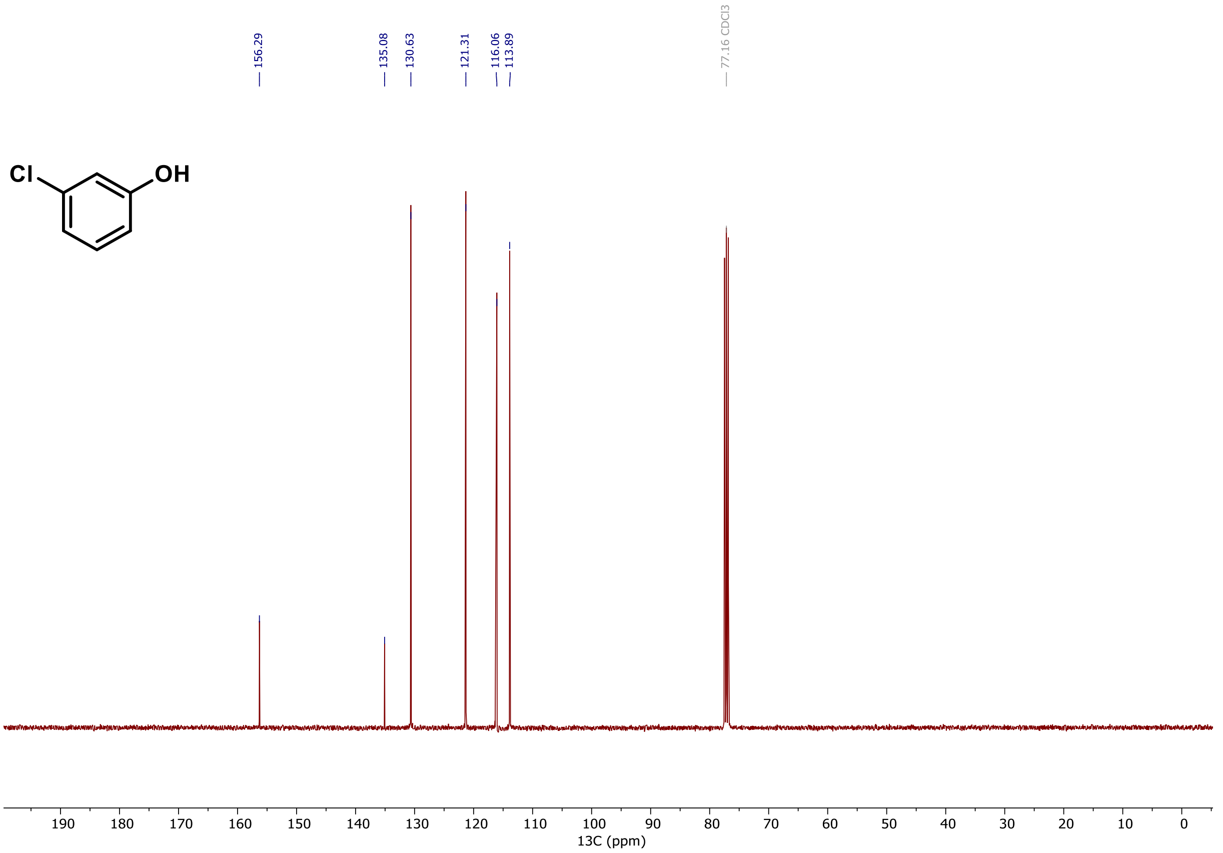

^{1}H NMR (400 MHz, $CDCl_3$) of compound **19.**

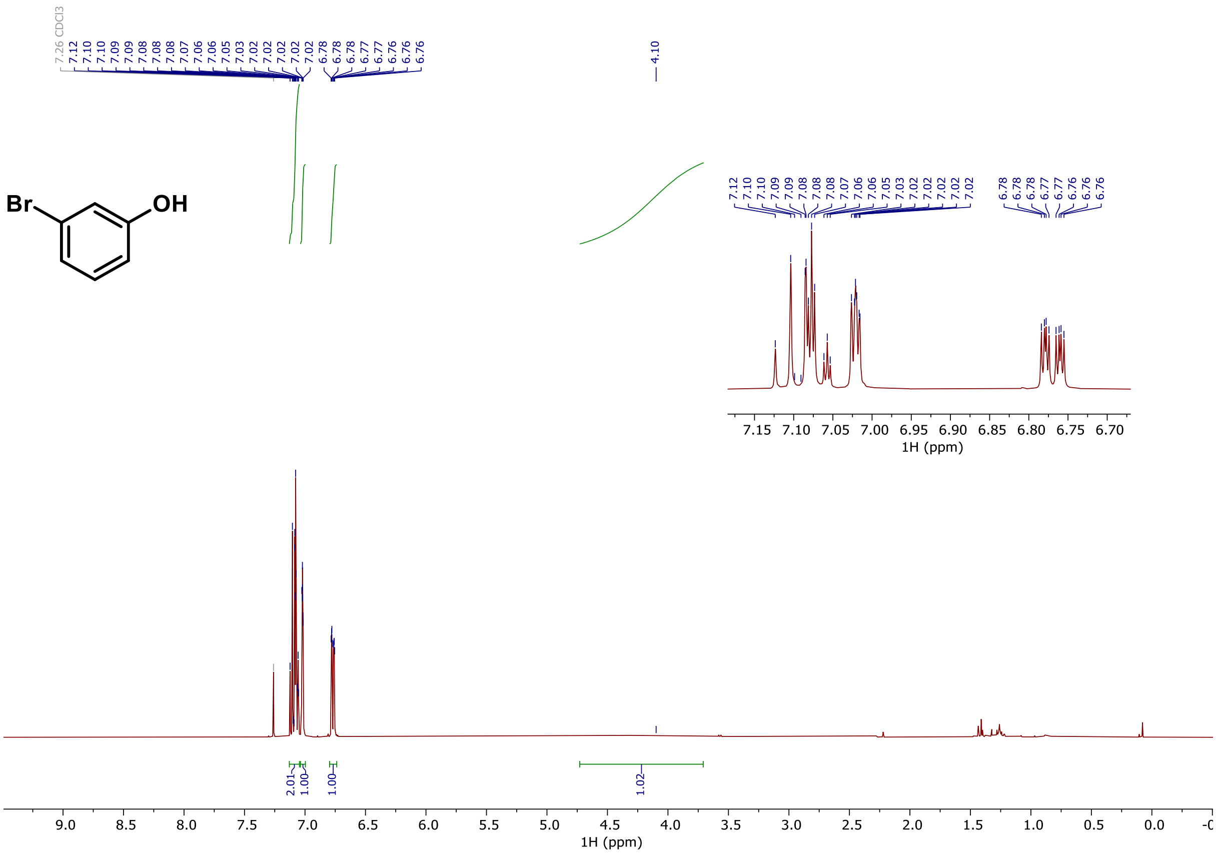

^{13}C NMR (101 MHz, $CDCl_3$) of compound **19.**

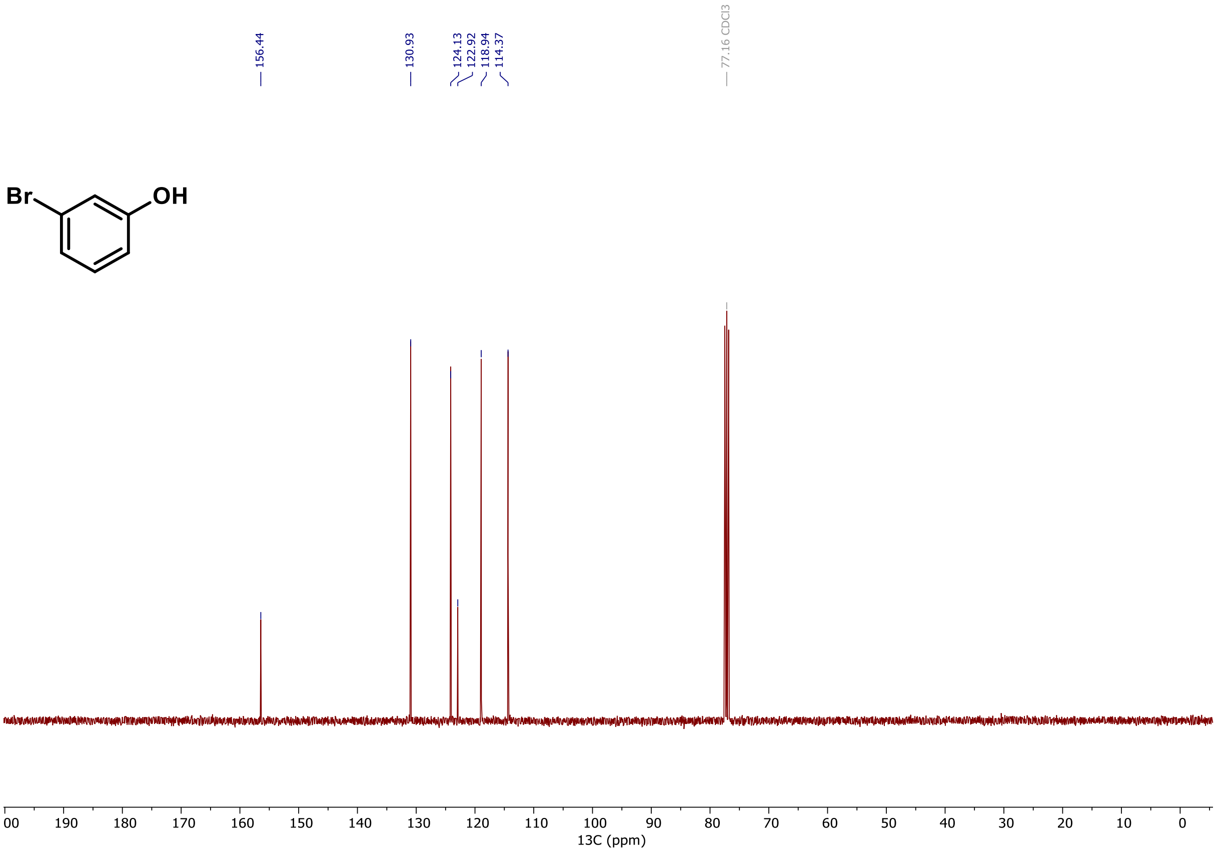

^{1}H NMR (300 MHz, $CDCl_3$) of compound **7.**

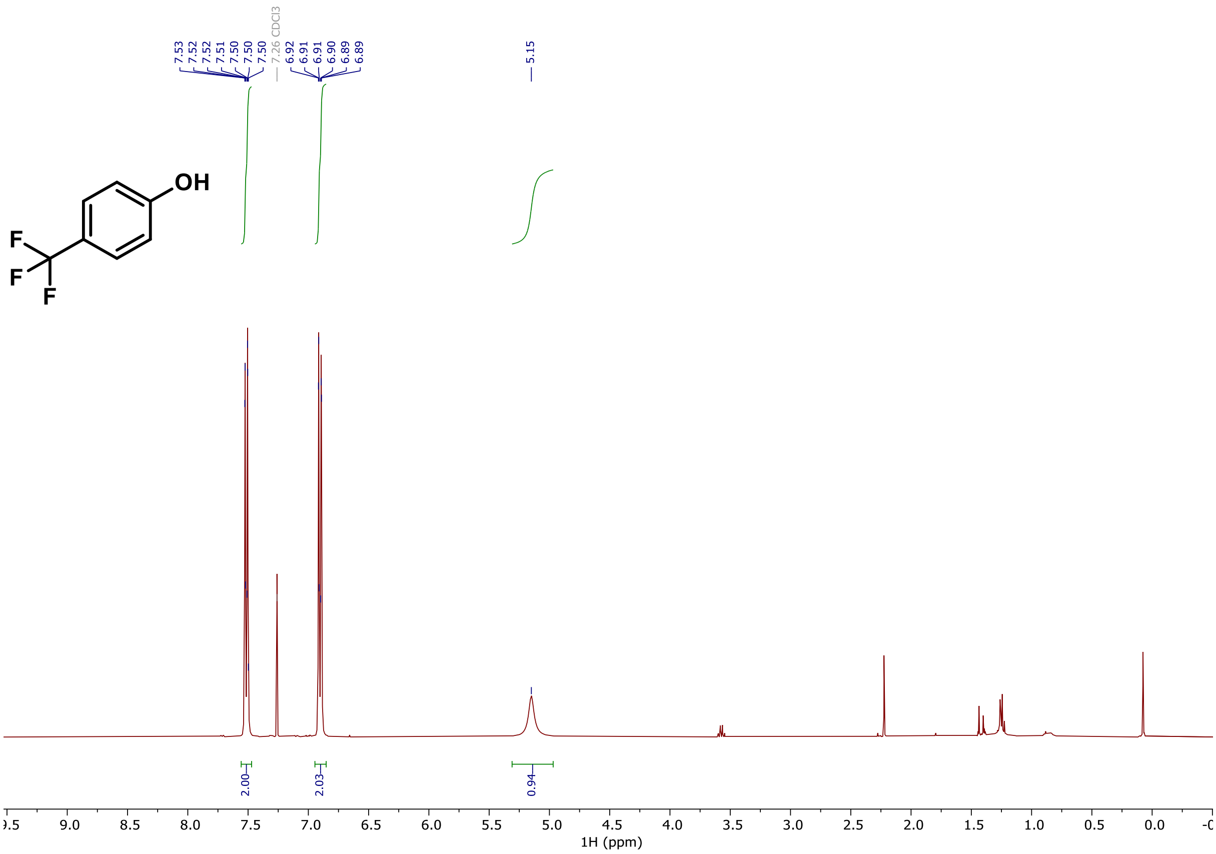

^{13}C NMR (101 MHz, $CDCl_3$) of compound **7.**

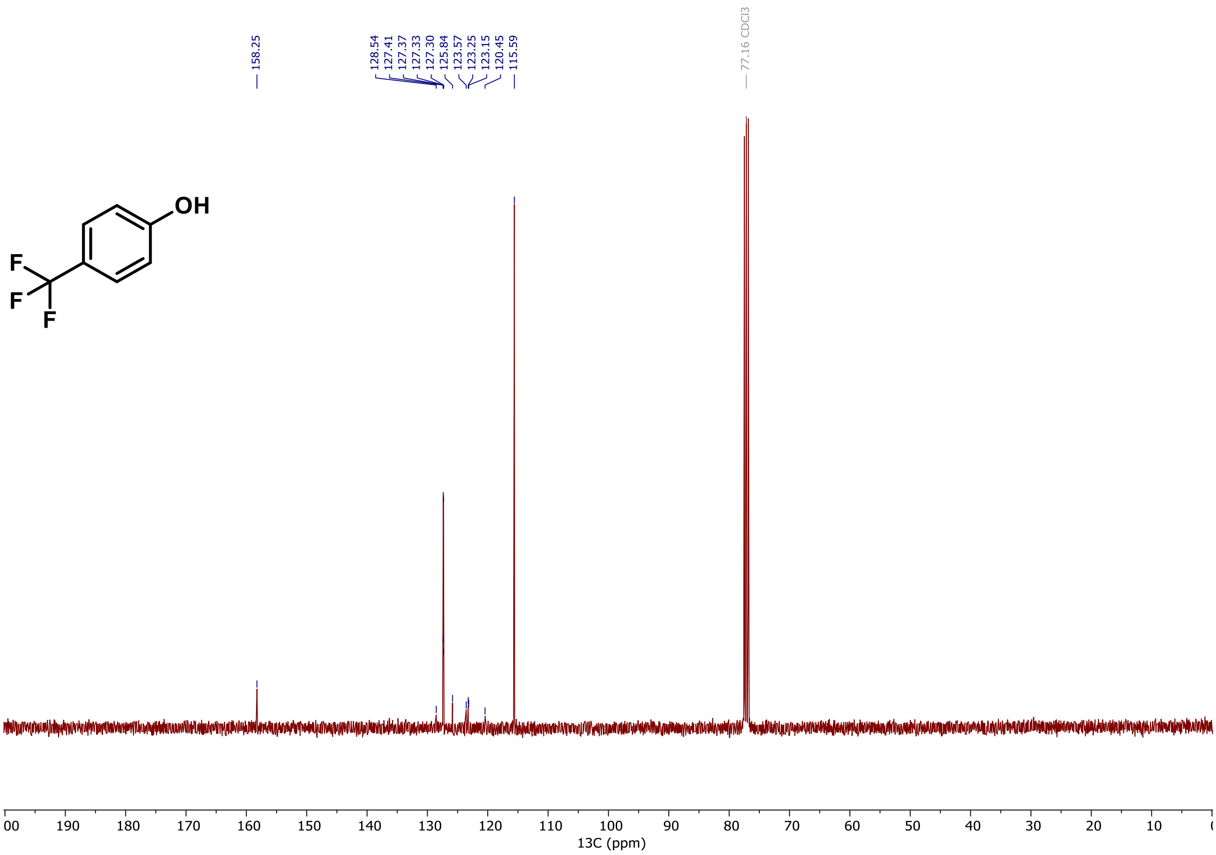

^{1}H NMR (400 MHz, $CDCl_3$) of compound **20.**

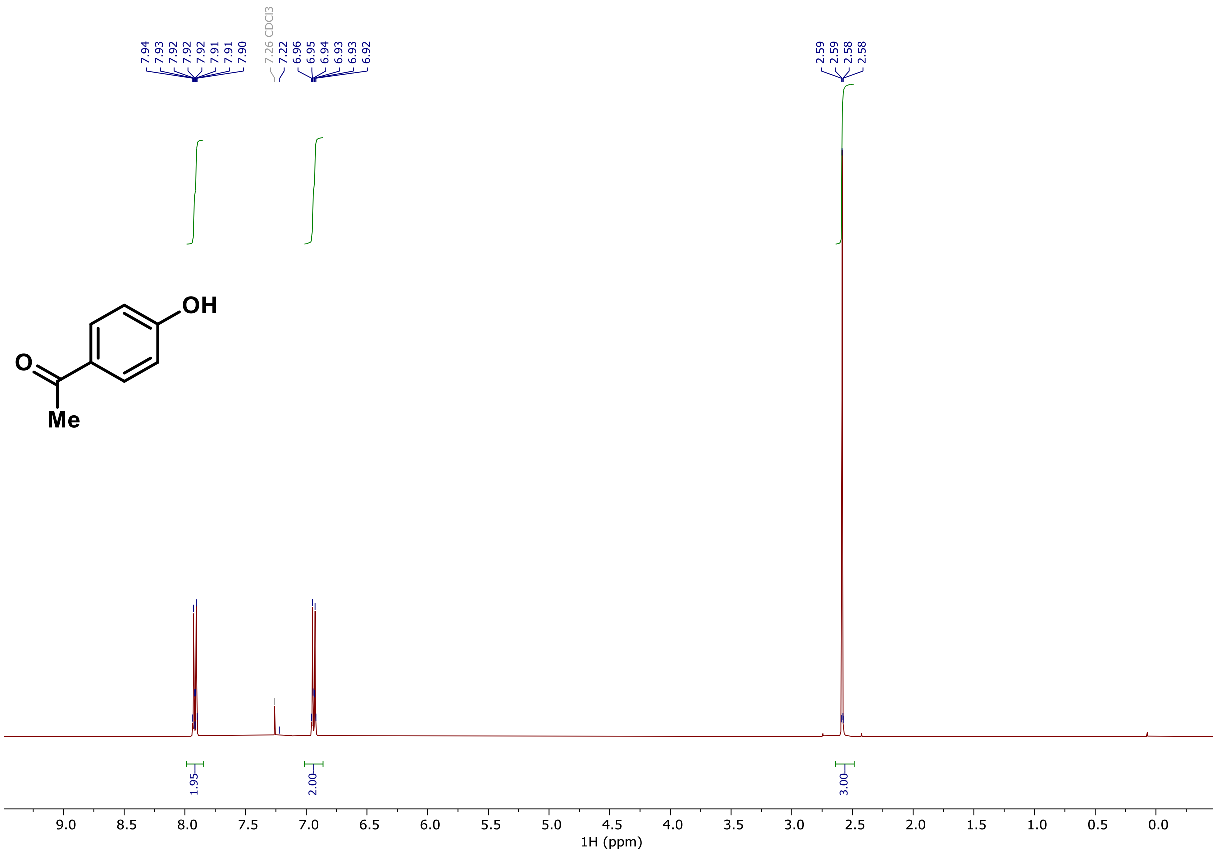

^{13}C NMR (101 MHz, $CDCl_3$) of compound **20.**

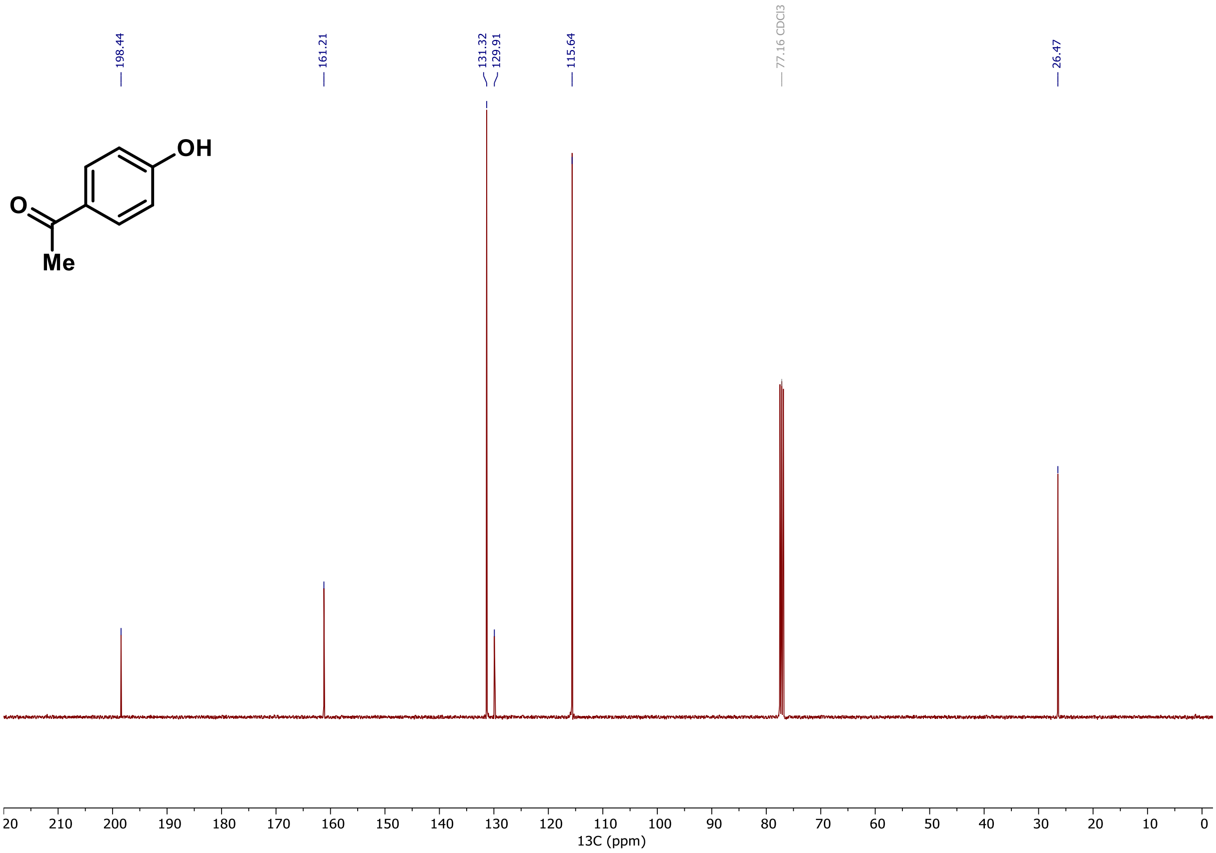

^{1}H NMR (300 MHz, CDCl$_3$) of compound **21.**

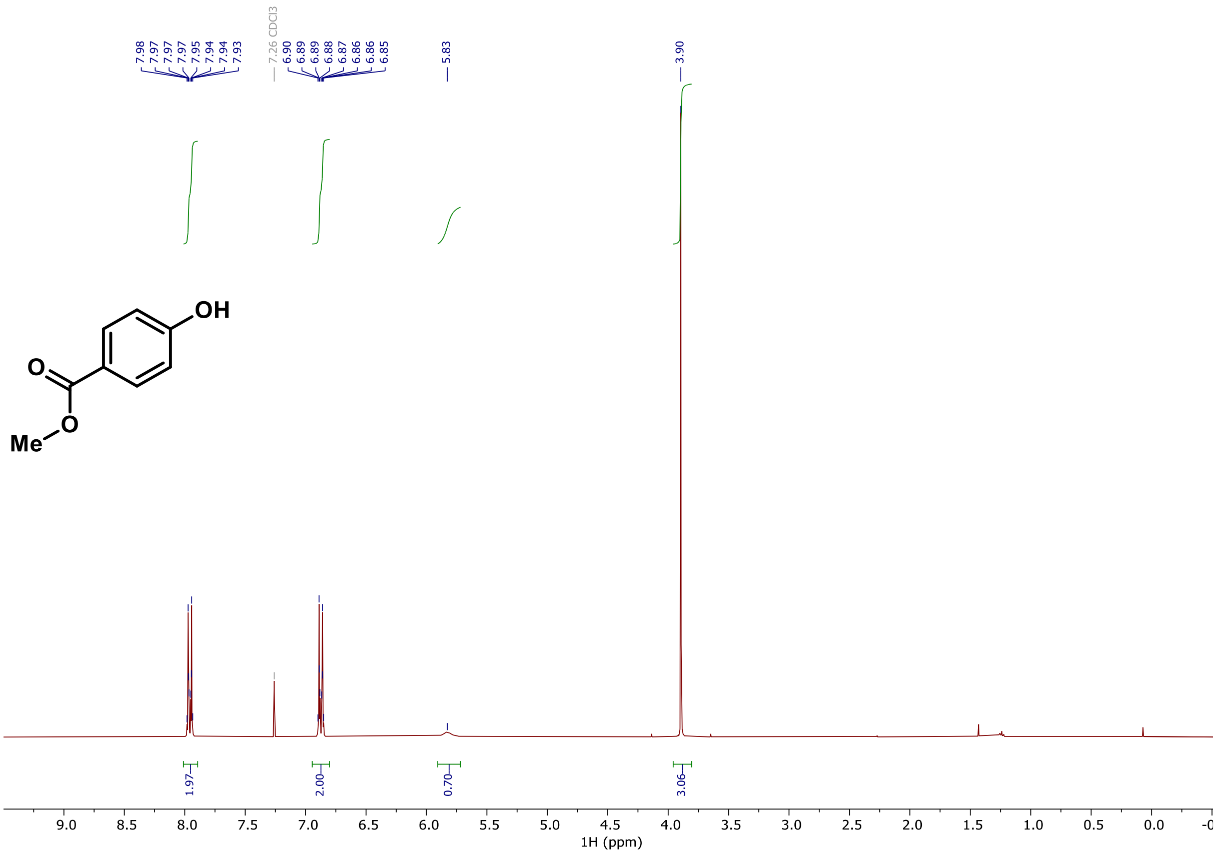

^{13}C NMR (75 MHz, CDCl$_3$) of compound **21.**

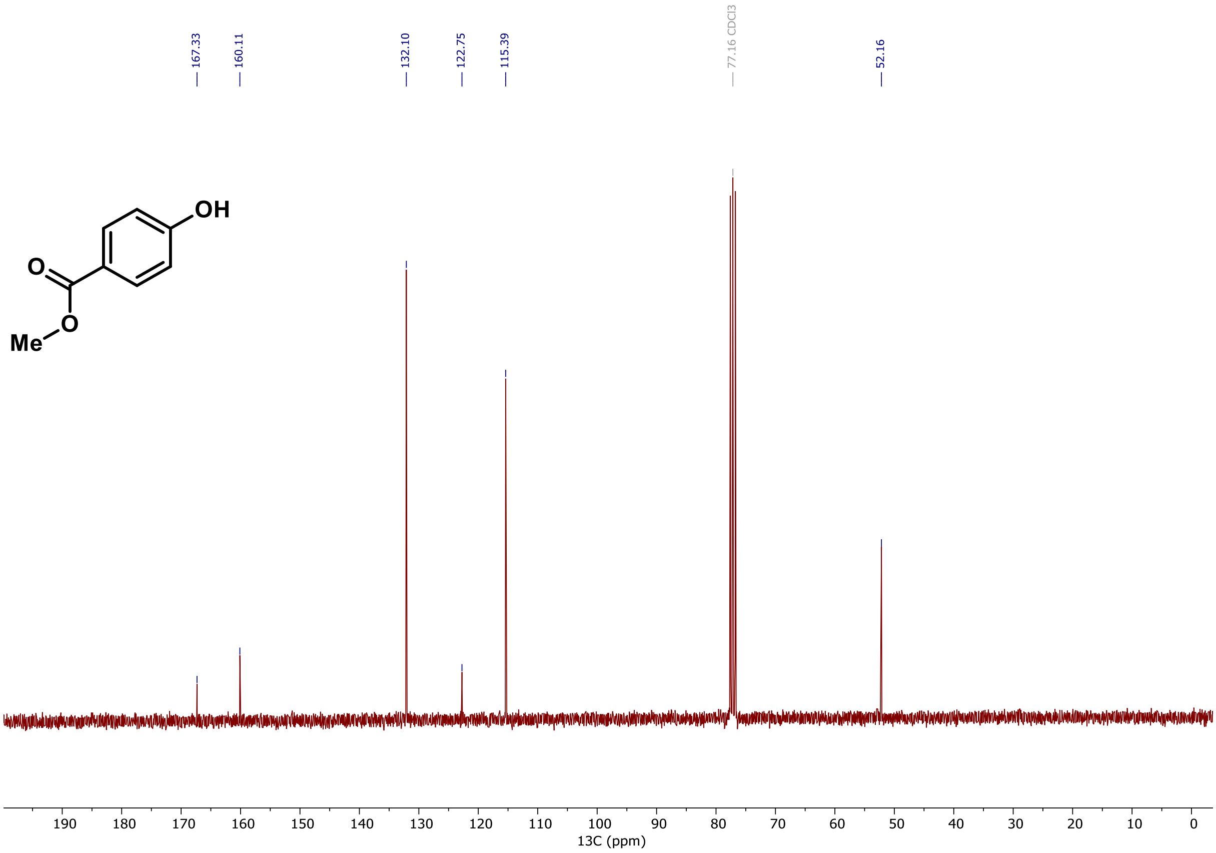

^{1}H NMR (400 MHz, $CDCl_3$) of compound **22.**

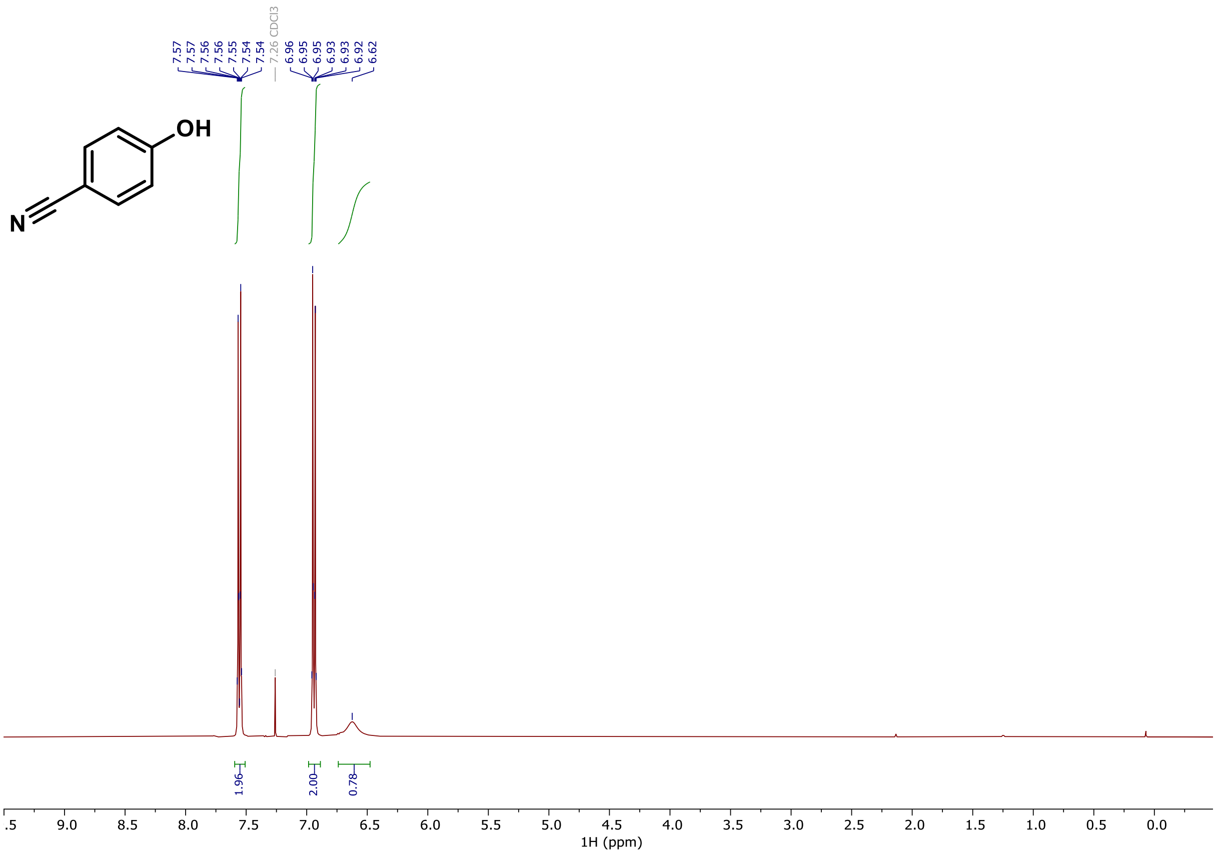

^{13}C NMR (101 MHz, $CDCl_3$) of compound **22.**

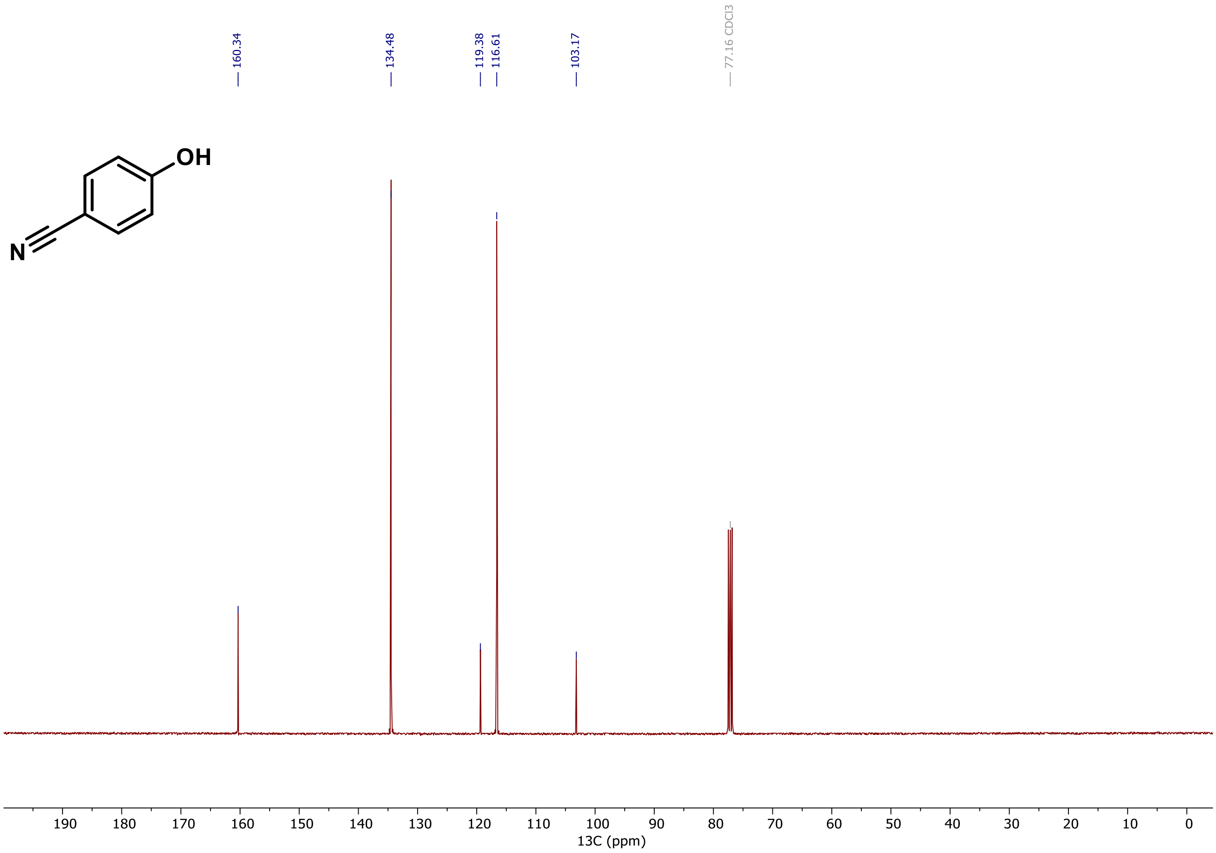

^{1}H NMR (400 MHz, CDCl$_3$) of compound **23.**

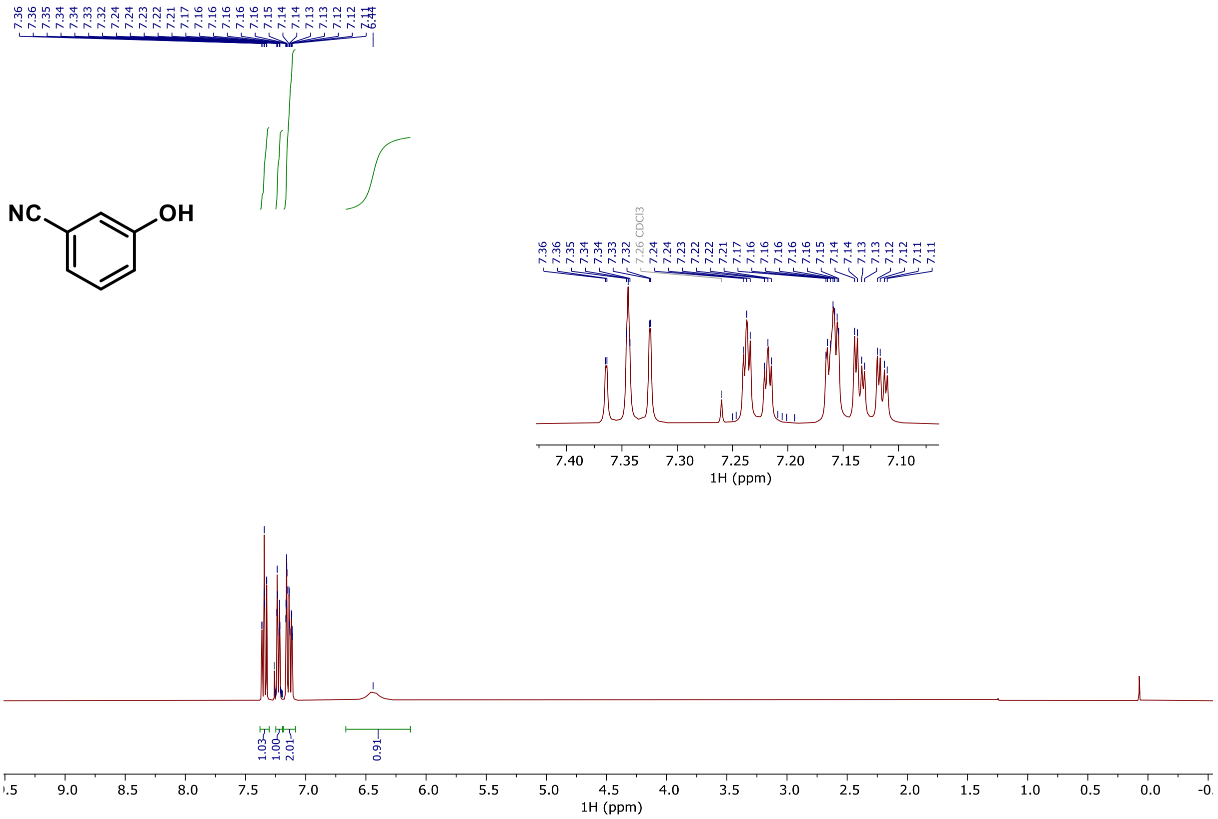

^{13}C NMR (101 MHz, CDCl$_3$) of compound **23.**

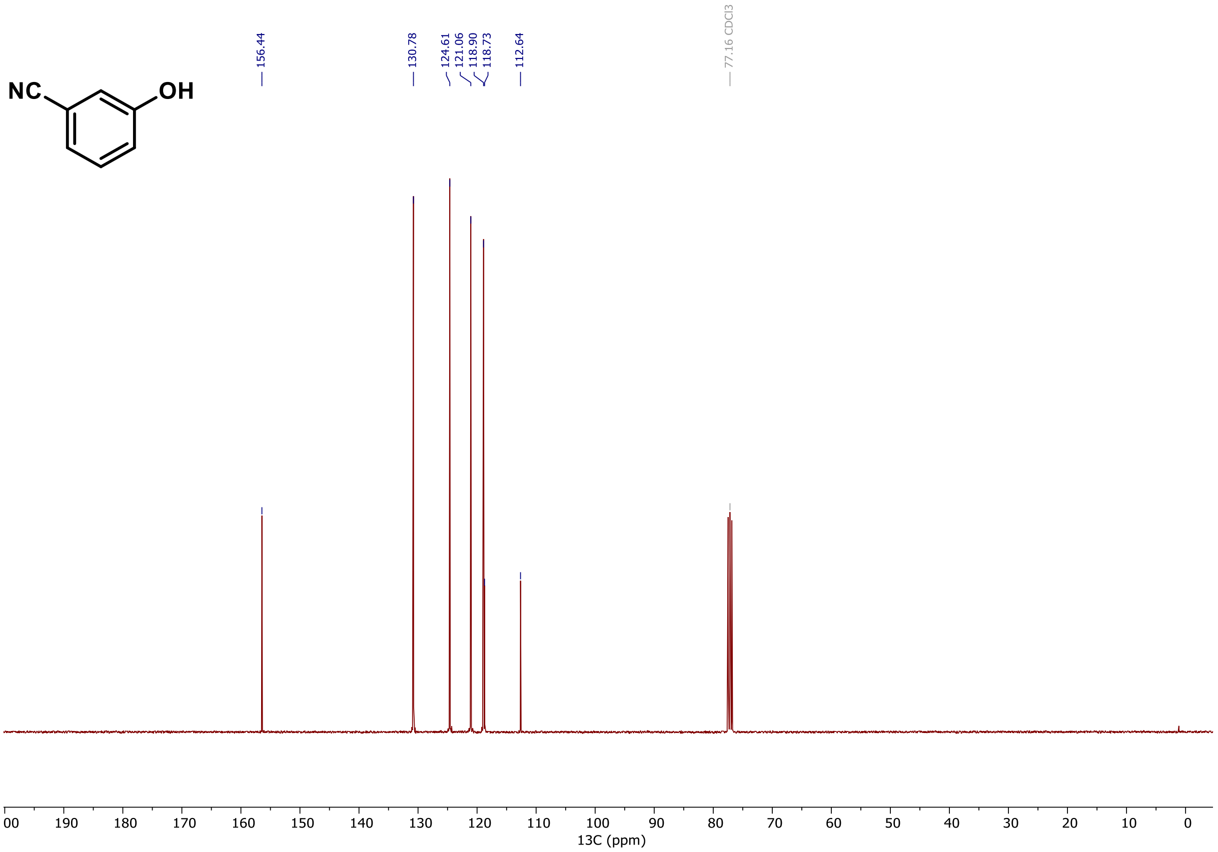

^{1}H NMR (400 MHz, CDCl$_3$) of compound **24.**

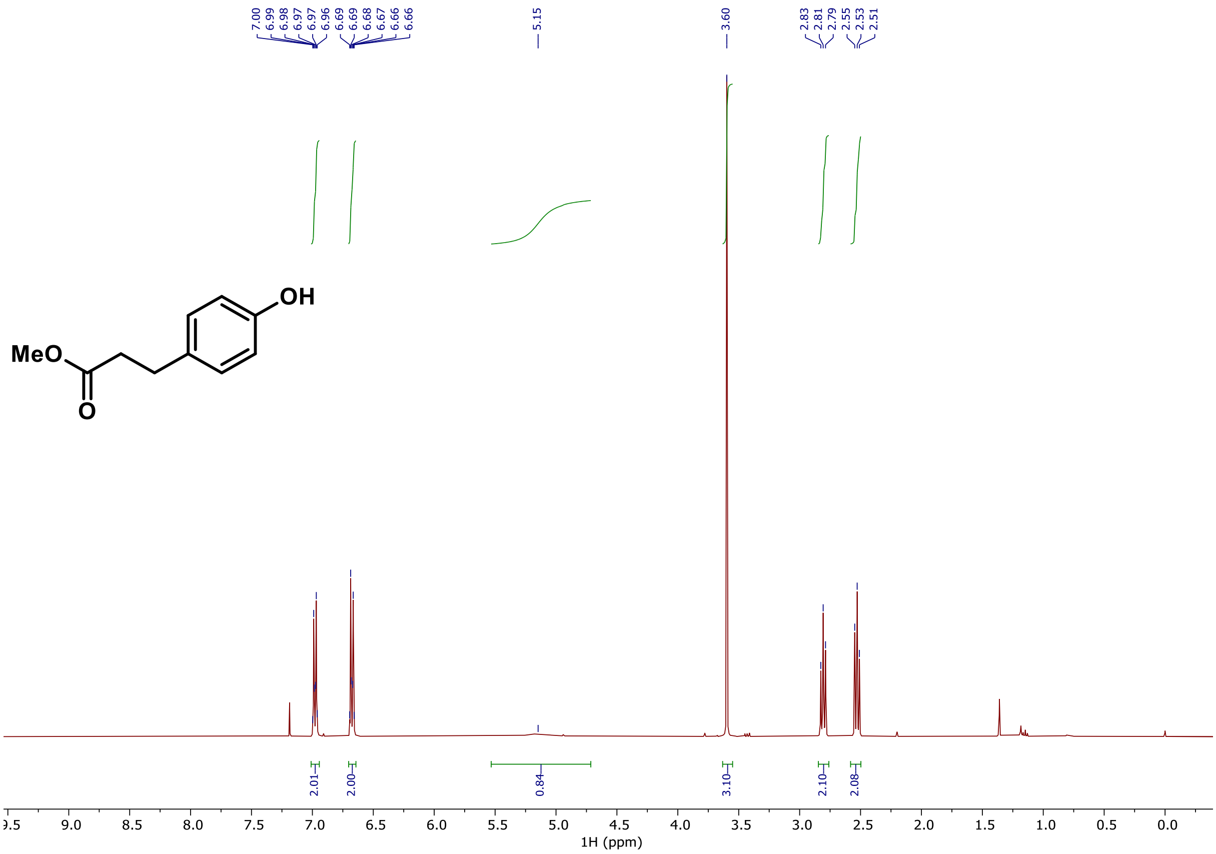

^{13}C NMR (101 MHz, CDCl$_3$) of compound **24.**

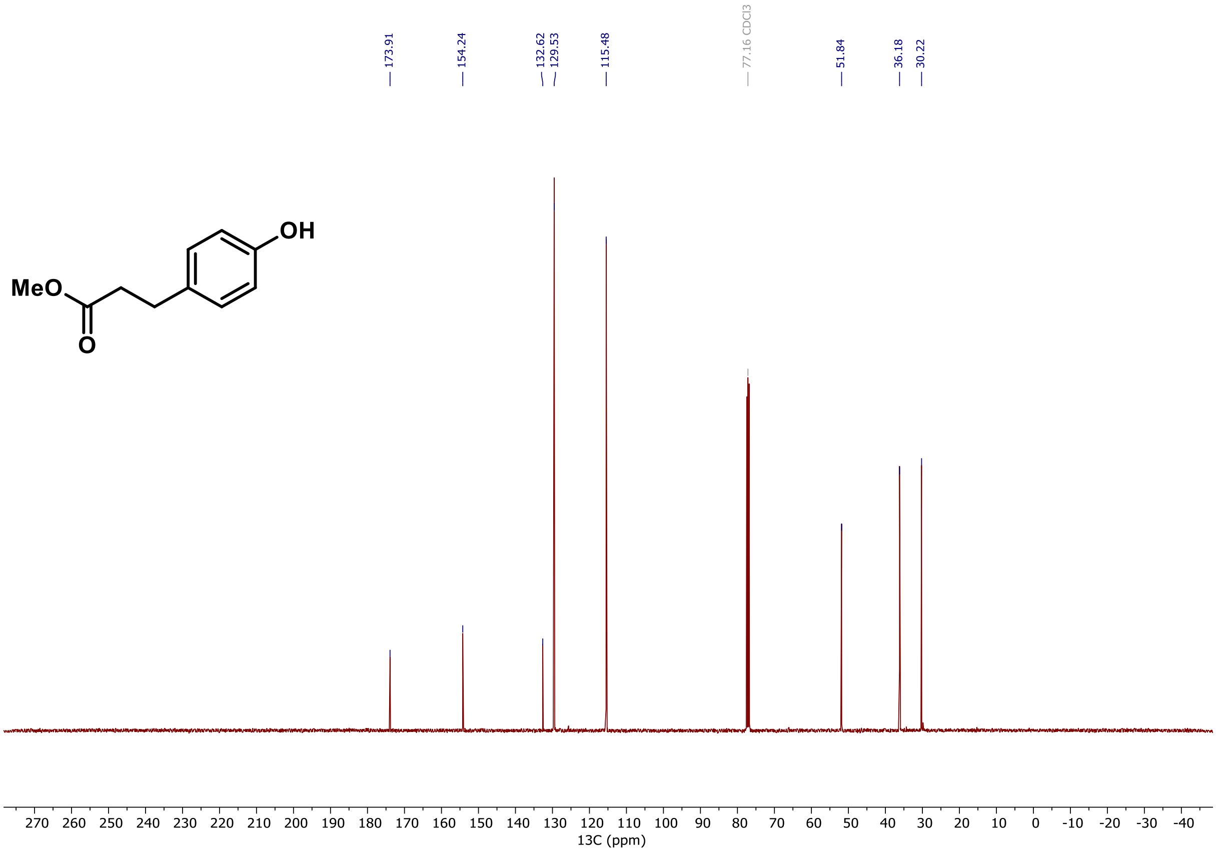

^{1}H NMR (300 MHz, CDCl$_3$) of compound **25.**

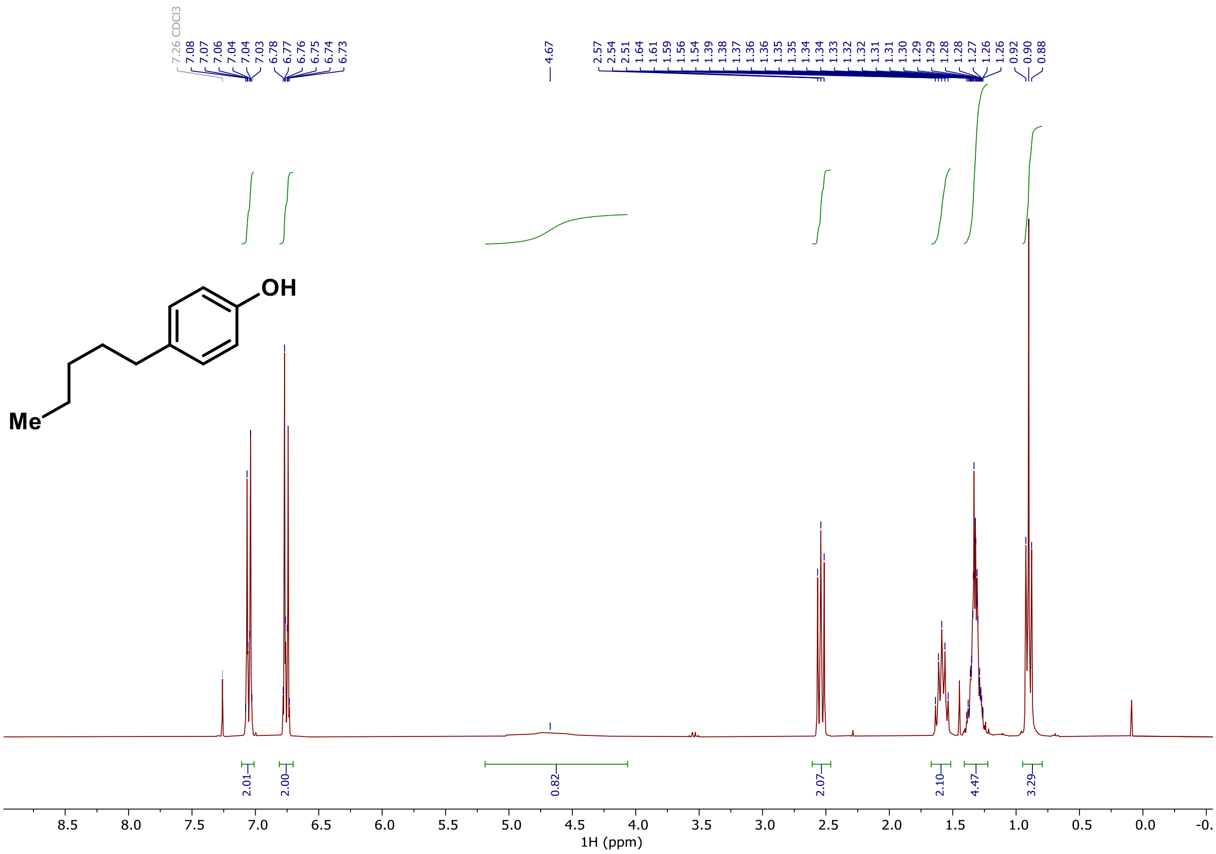

^{13}C NMR (75 MHz, CDCl$_3$) of compound **25.**

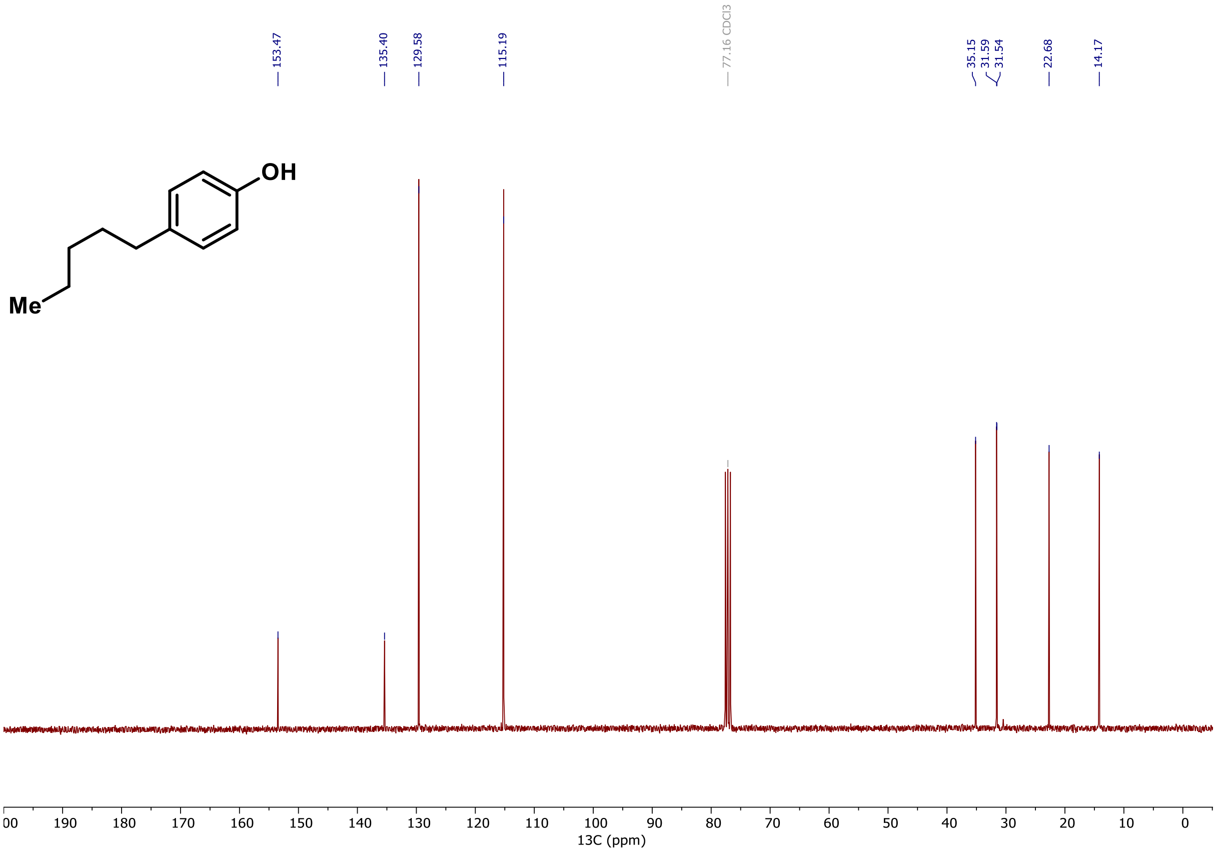

^{1}H NMR (400 MHz, $CDCl_3$) of compound **26.**

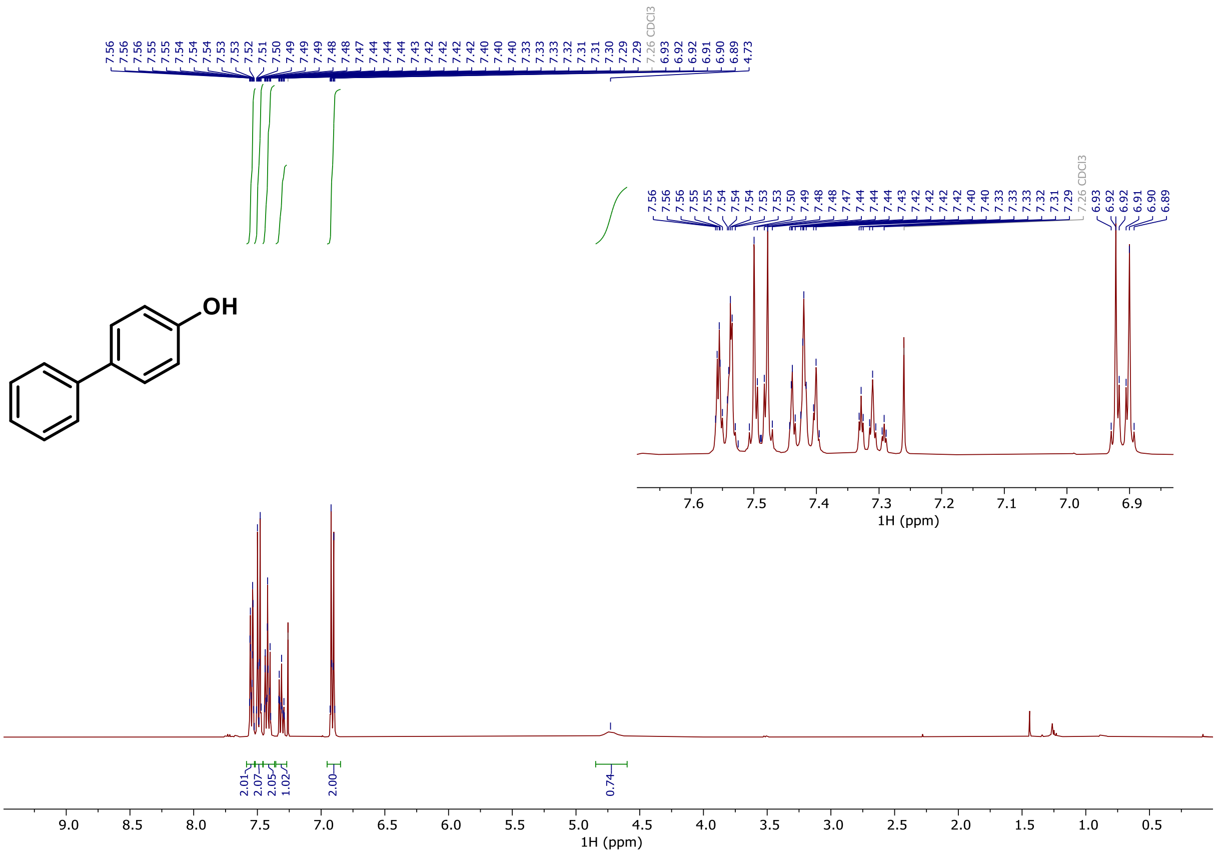

^{13}C NMR (101 MHz, $CDCl_3$) of compound **26.**

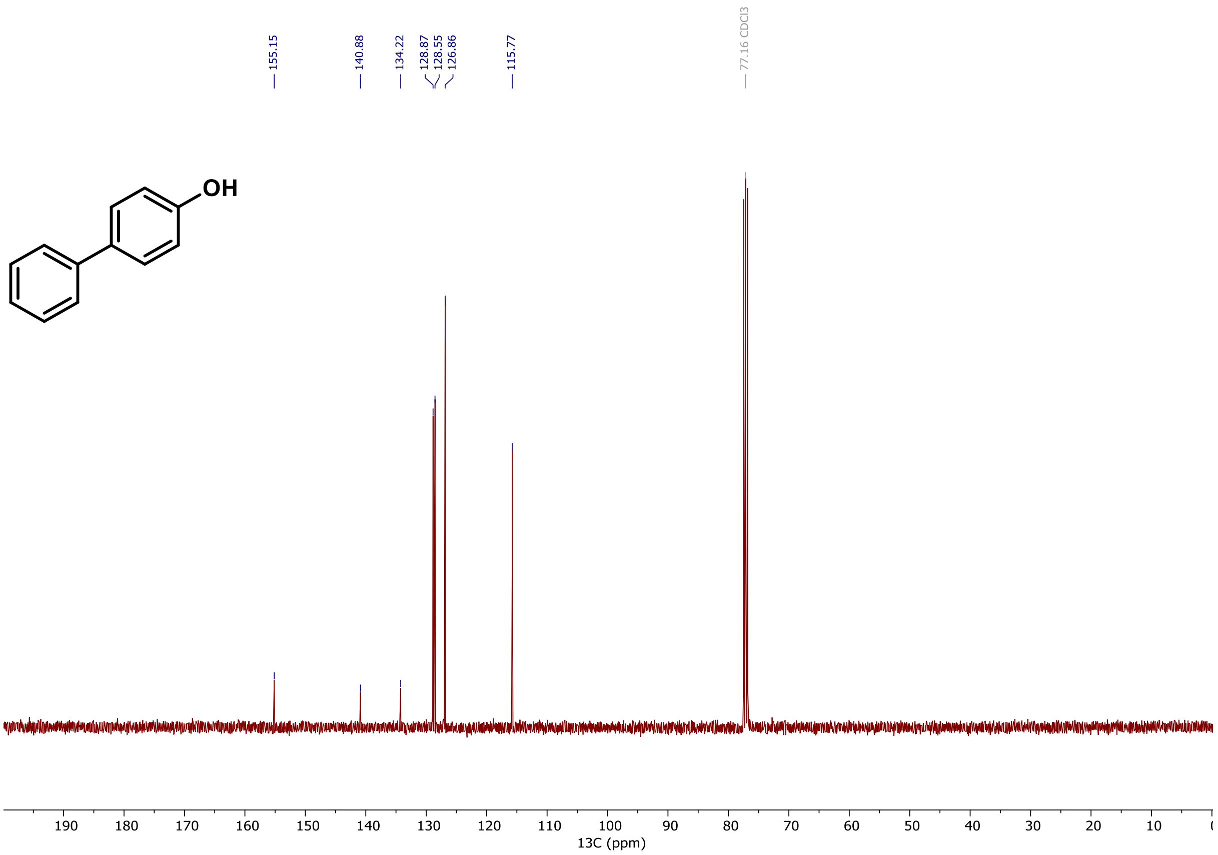

^{1}H NMR (400 MHz, $CDCl_3$) of compound 27.

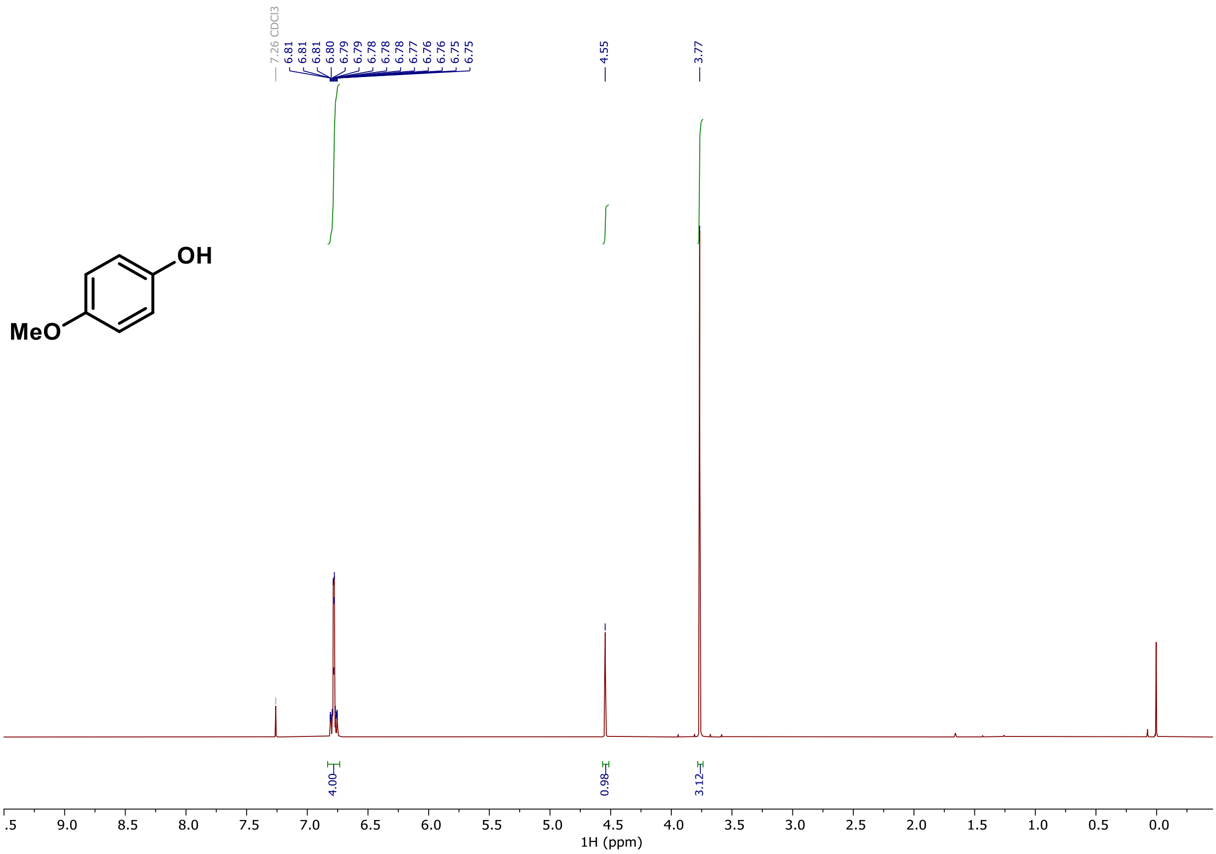

^{13}C NMR (101 MHz, $CDCl_3$) of compound 27.

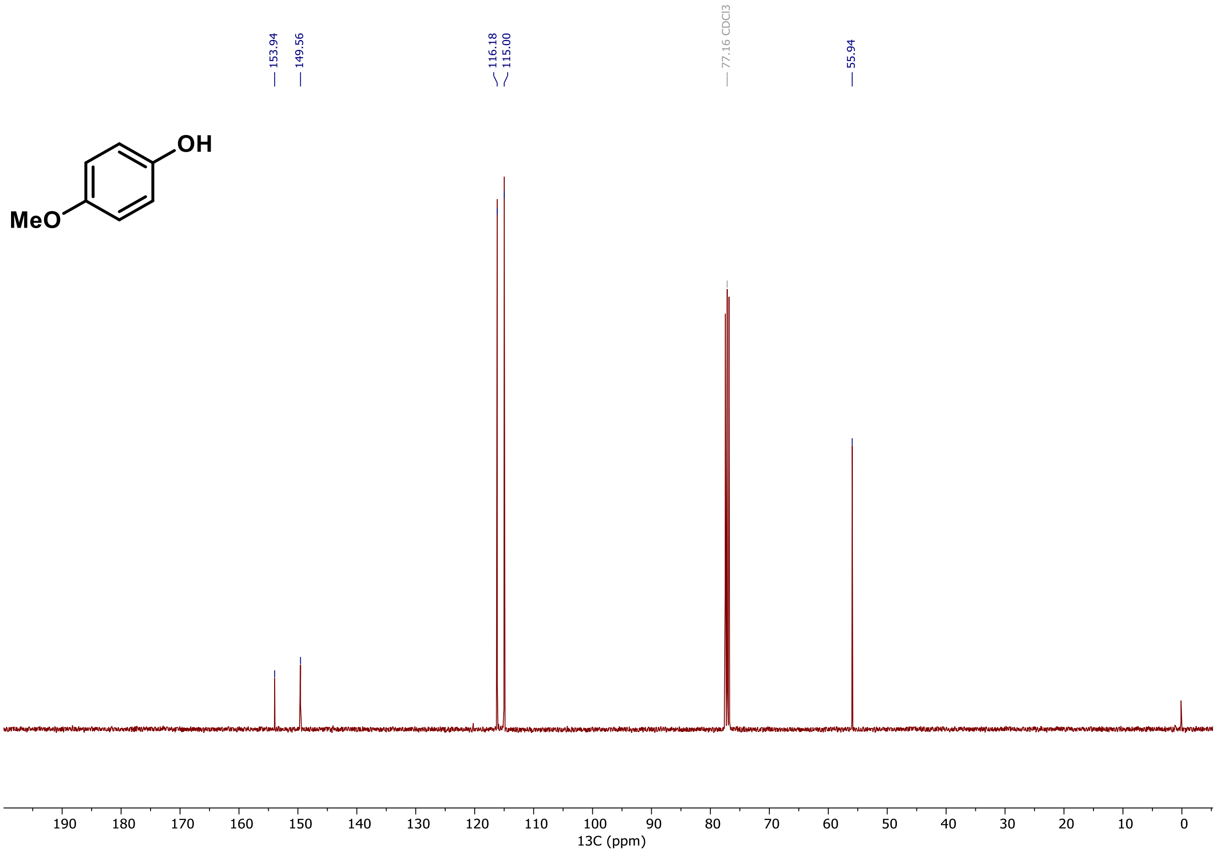

^{1}H NMR (300 MHz, $CDCl_3$) of compound 28.

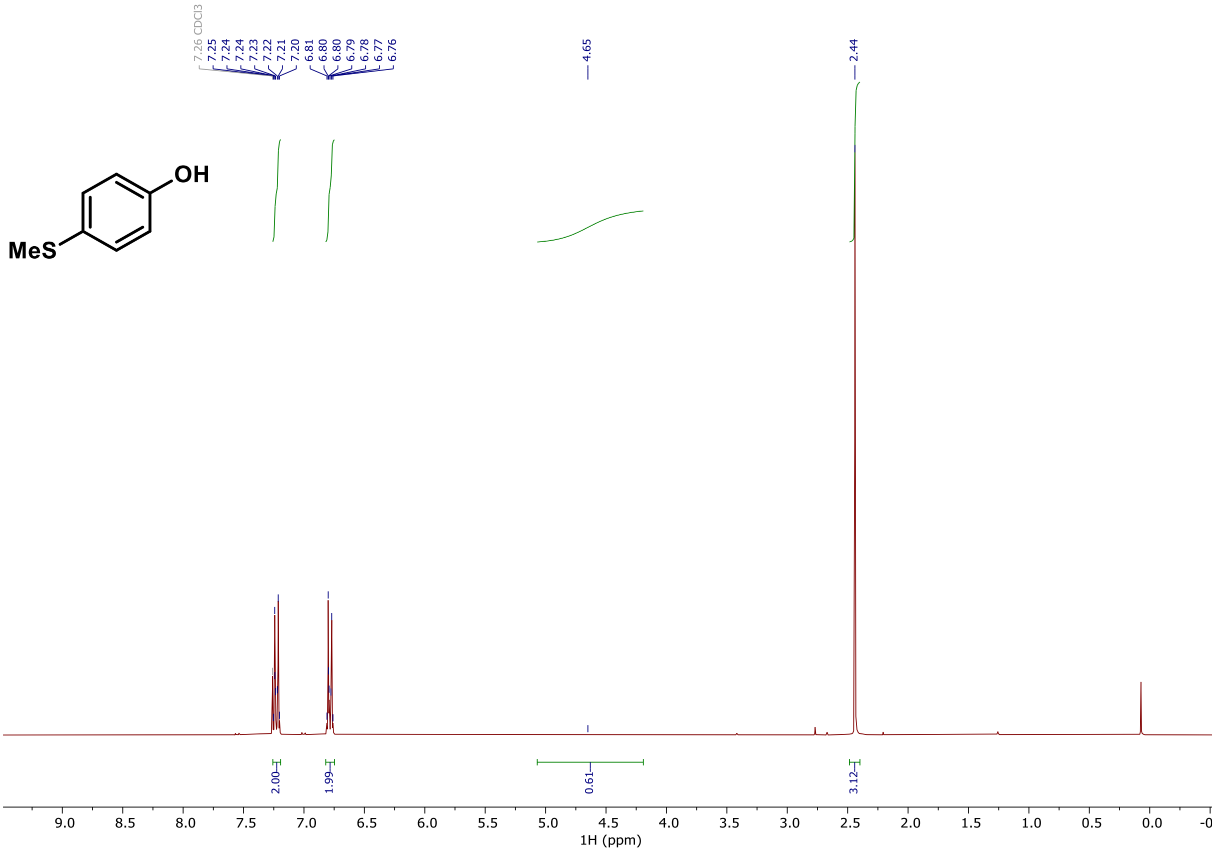

^{13}C NMR (75 MHz, $CDCl_3$) of compound 28.

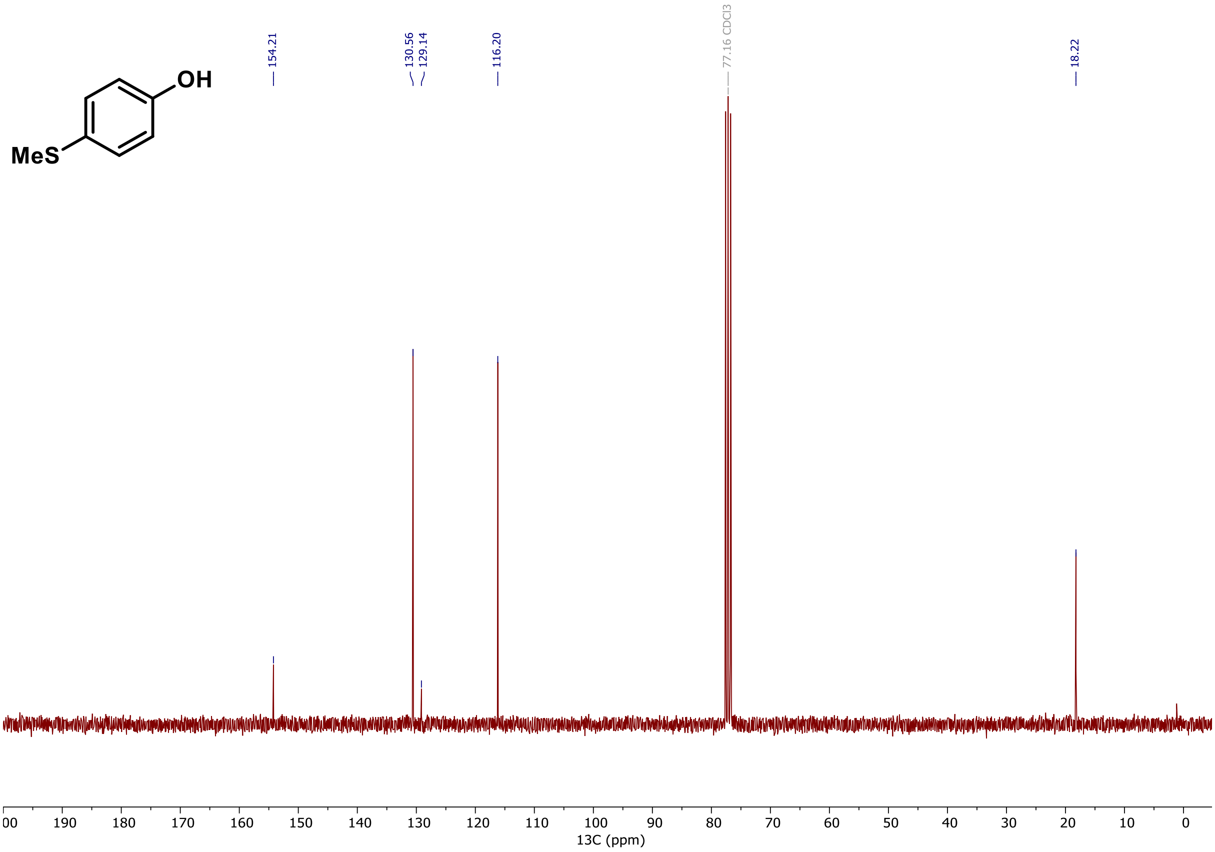

^{1}H NMR (400 MHz, CDCl₃) of compound **29.**

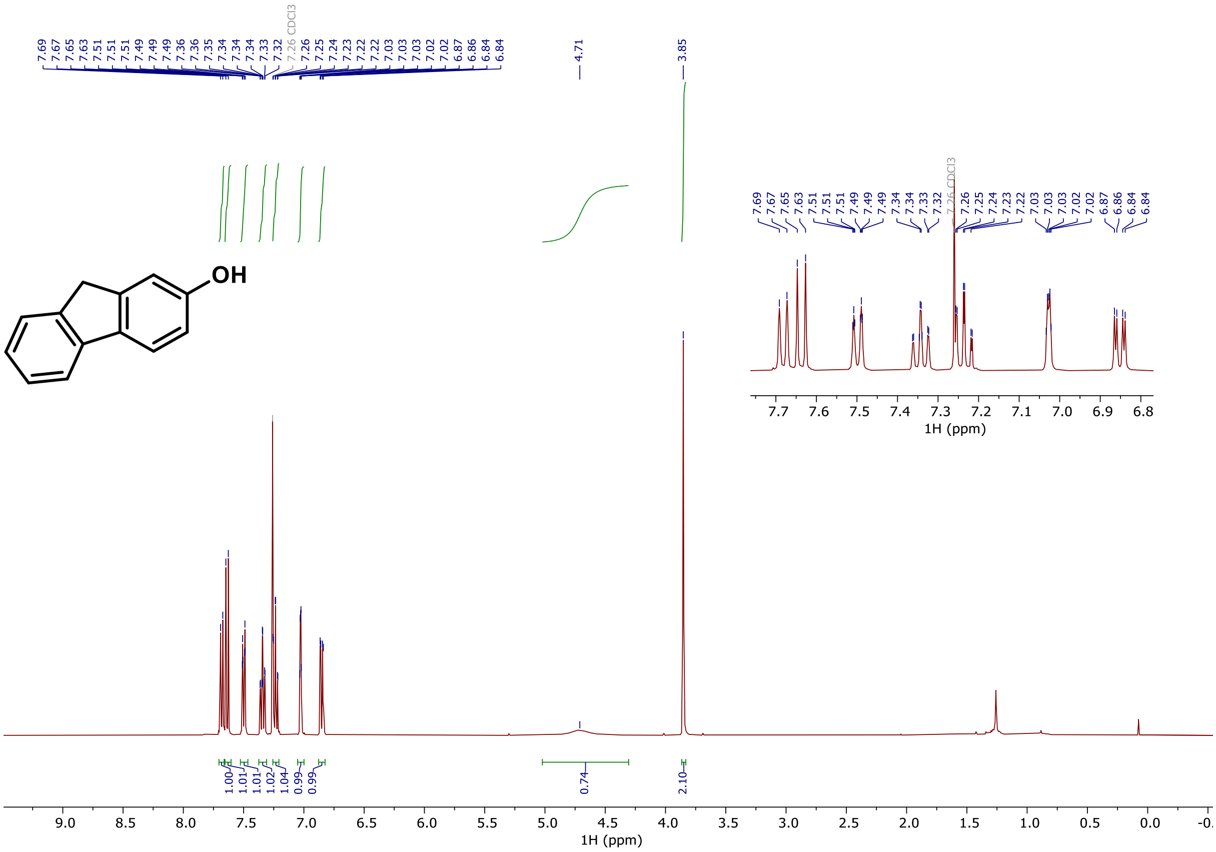

^{13}C NMR (101 MHz, CDCl₃) of compound **29.**

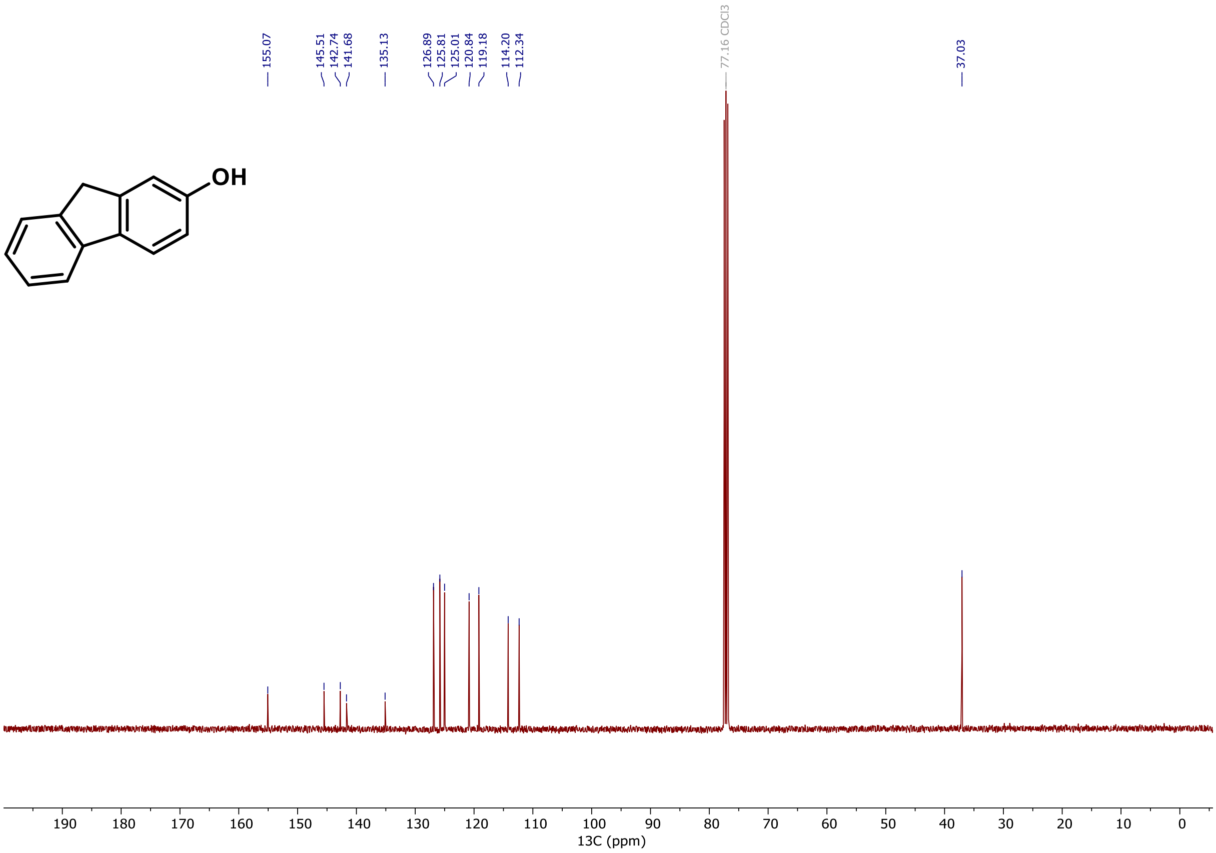

^{1}H NMR (300 MHz, CDCl$_3$) of compound **30.**

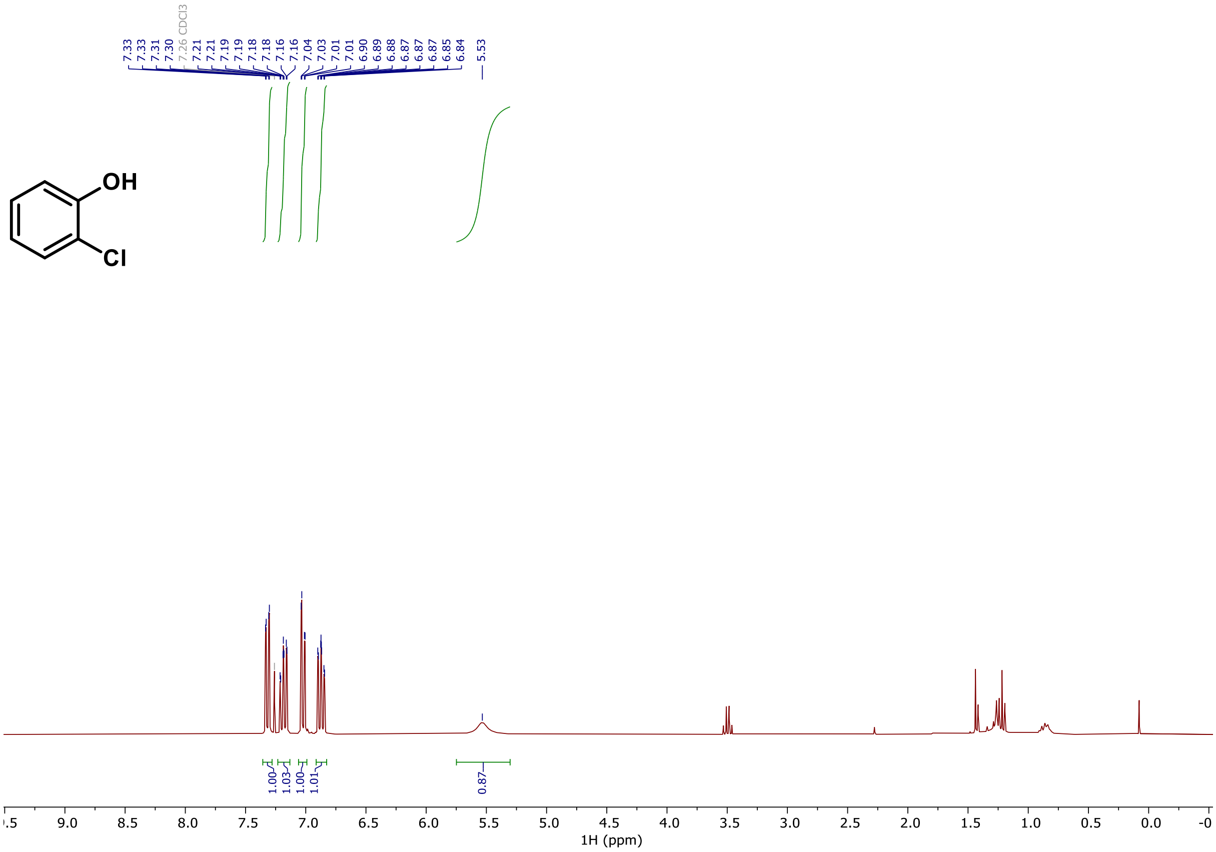

^{13}C NMR (75 MHz, CDCl$_3$) of compound **30.**

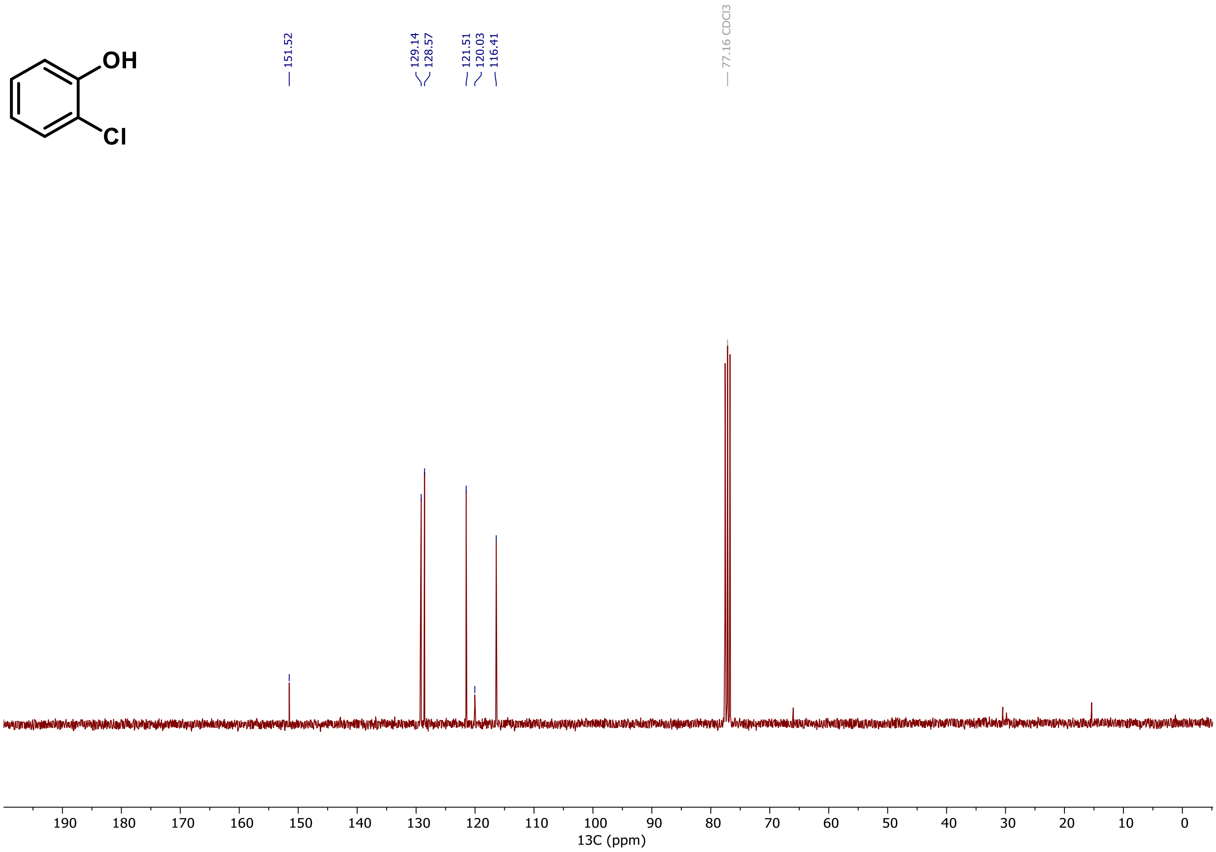

^{1}**H NMR (300 MHz, CDCl$_3$)** of compound **31.**

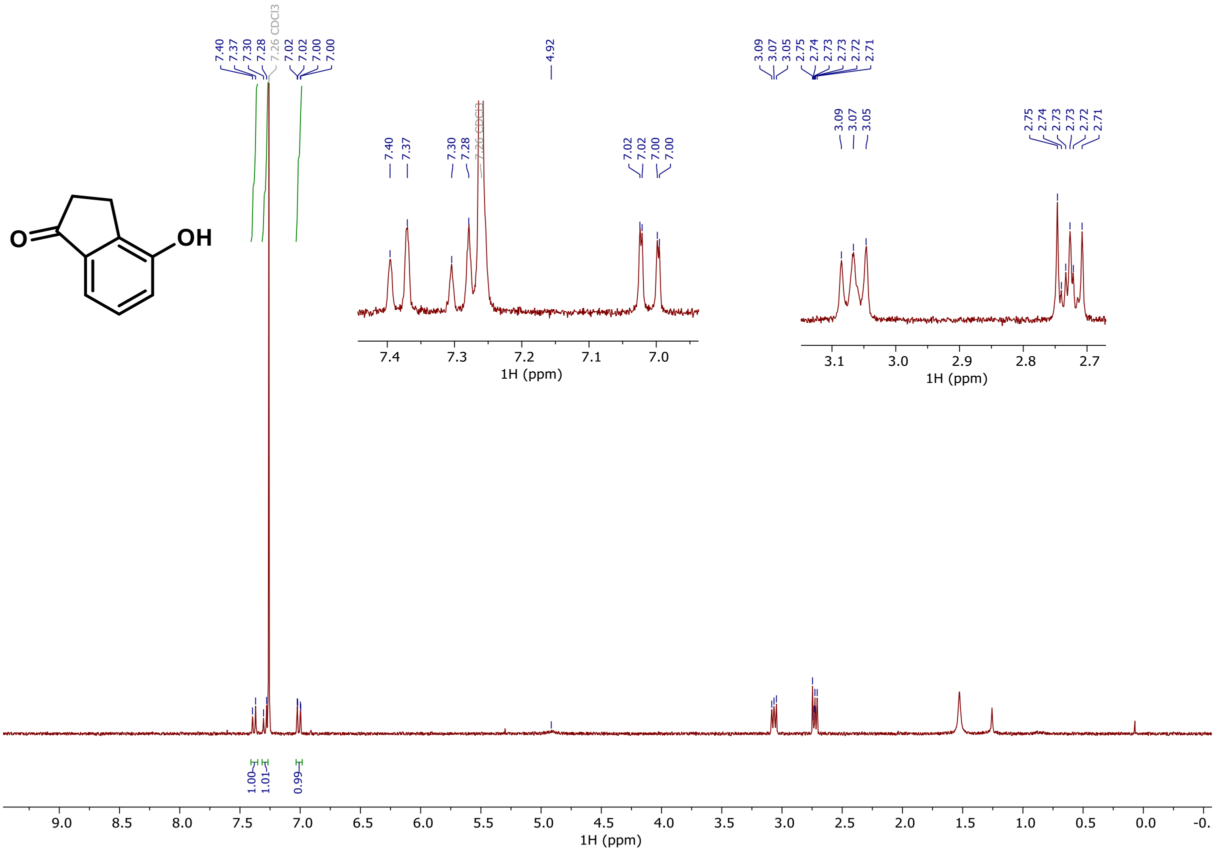

^{13}**C NMR (101 MHz, CDCl$_3$ with 2 drops of DMSO-*d$_6$*)** of compound **31.**

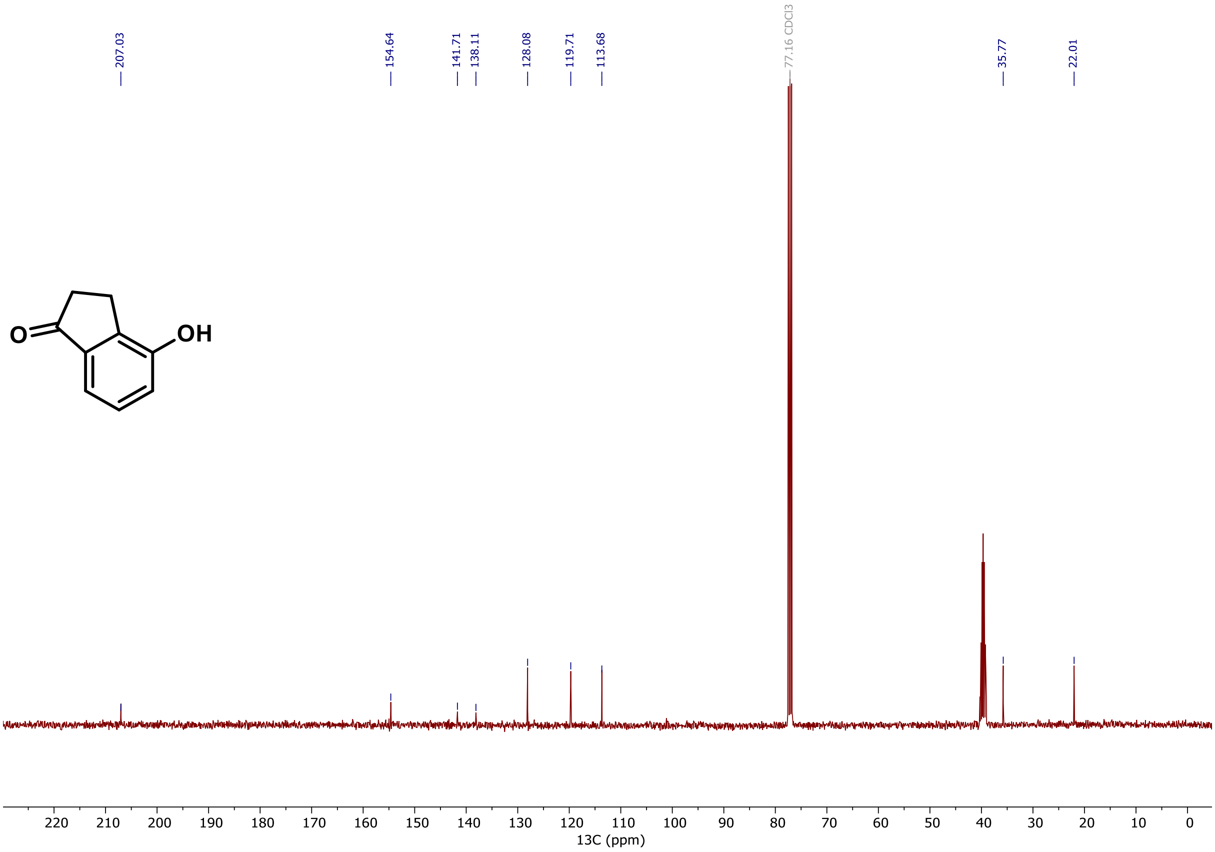

^{1}H NMR (400 MHz, $CDCl_3$) of compound **32.**

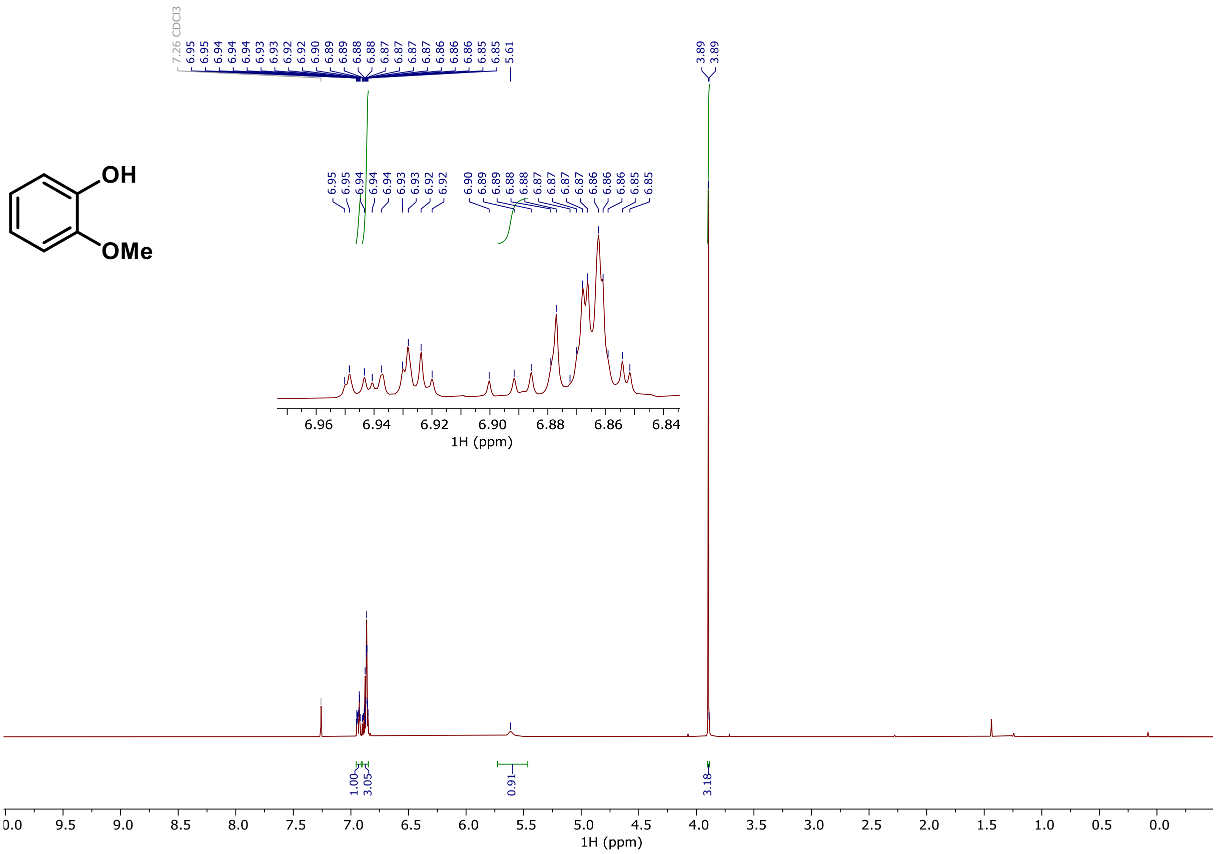

^{13}C NMR (101 MHz, $CDCl_3$) of compound **32.**

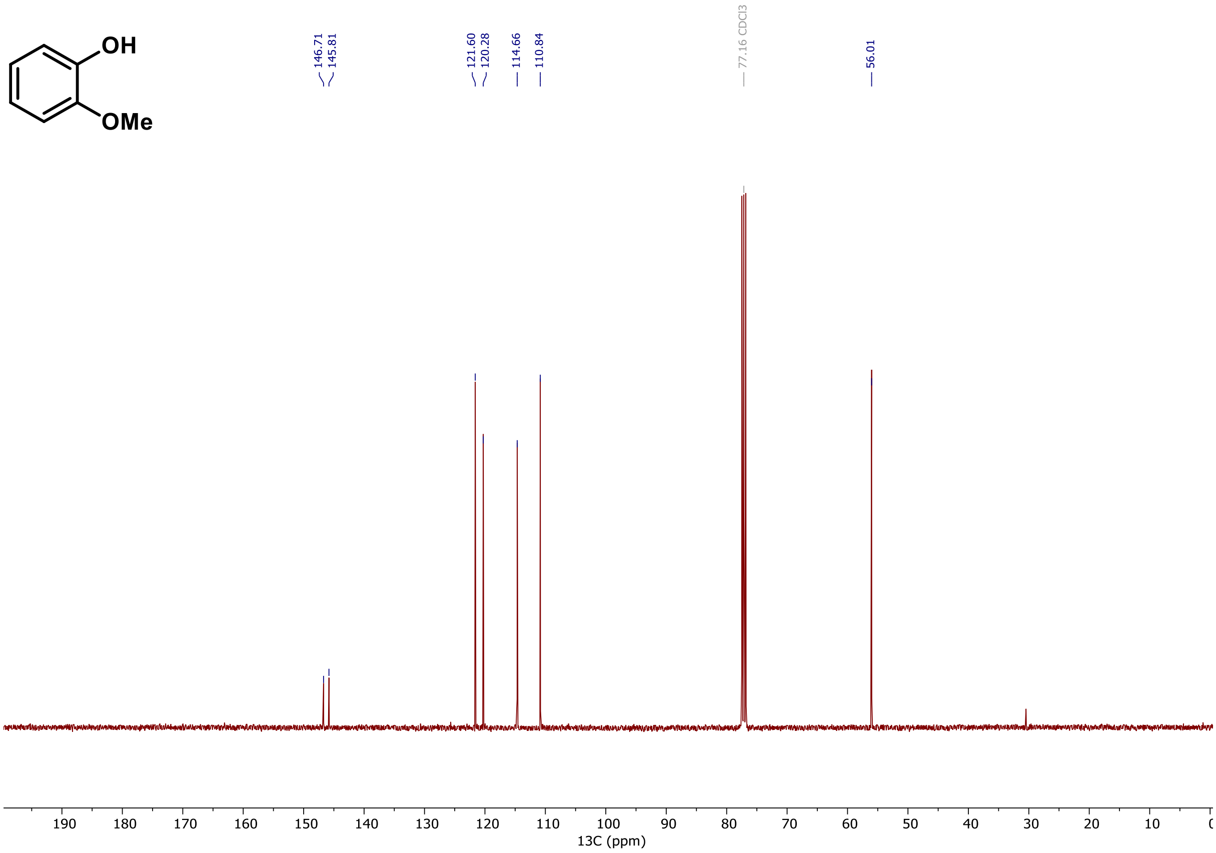

^{1}H NMR (400 MHz, $CDCl_3$) of compound **33.**

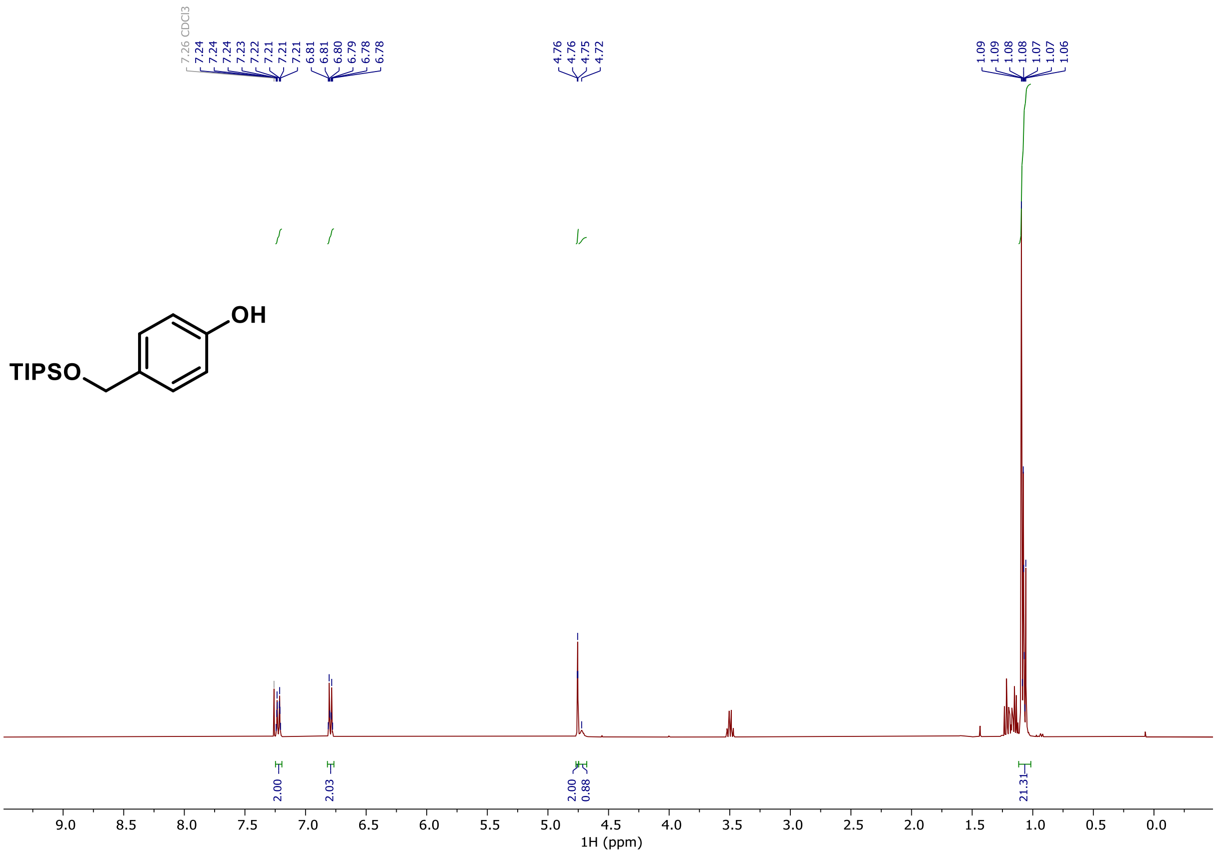

^{13}C NMR (101 MHz, $CDCl_3$) of compound **33.**

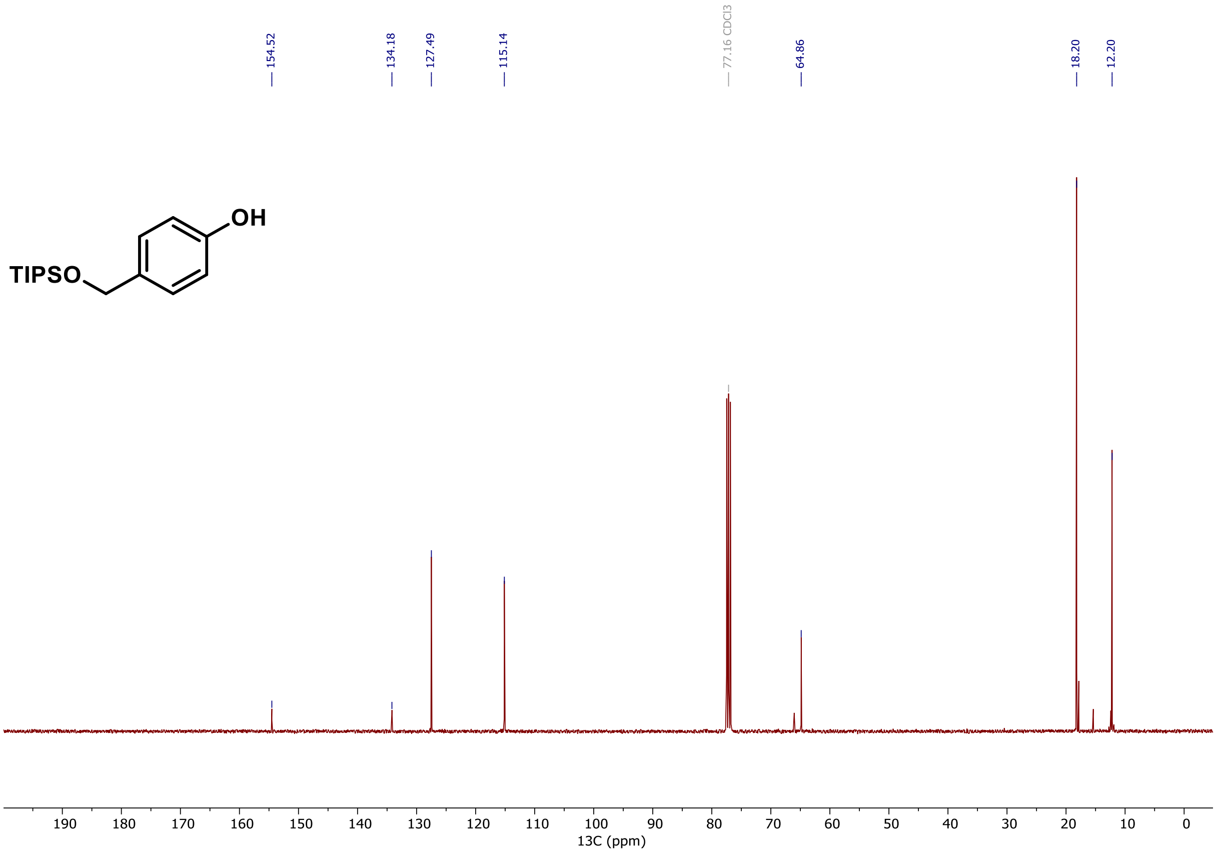

^{1}H NMR (400 MHz, $CDCl_3$) of compound **34.**

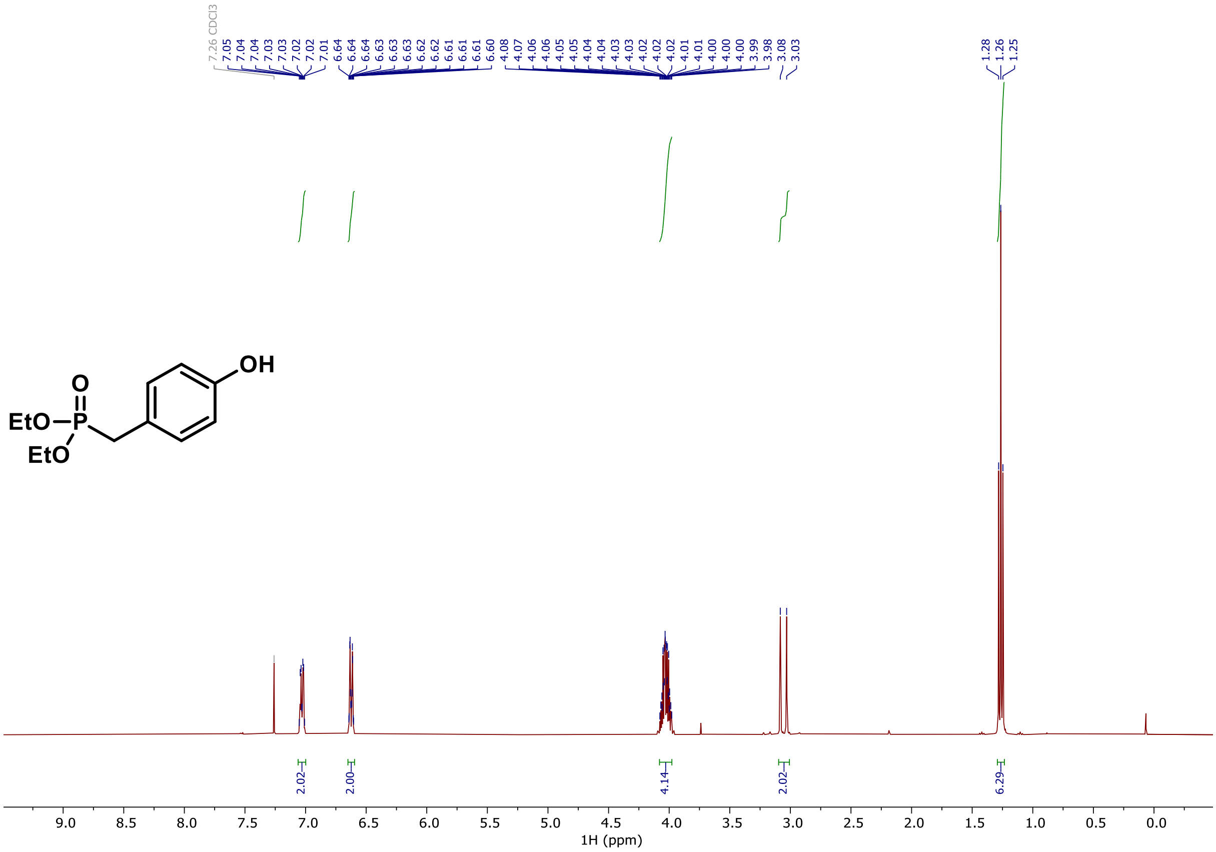

^{13}C NMR (101 MHz, $CDCl_3$) of compound **34.**

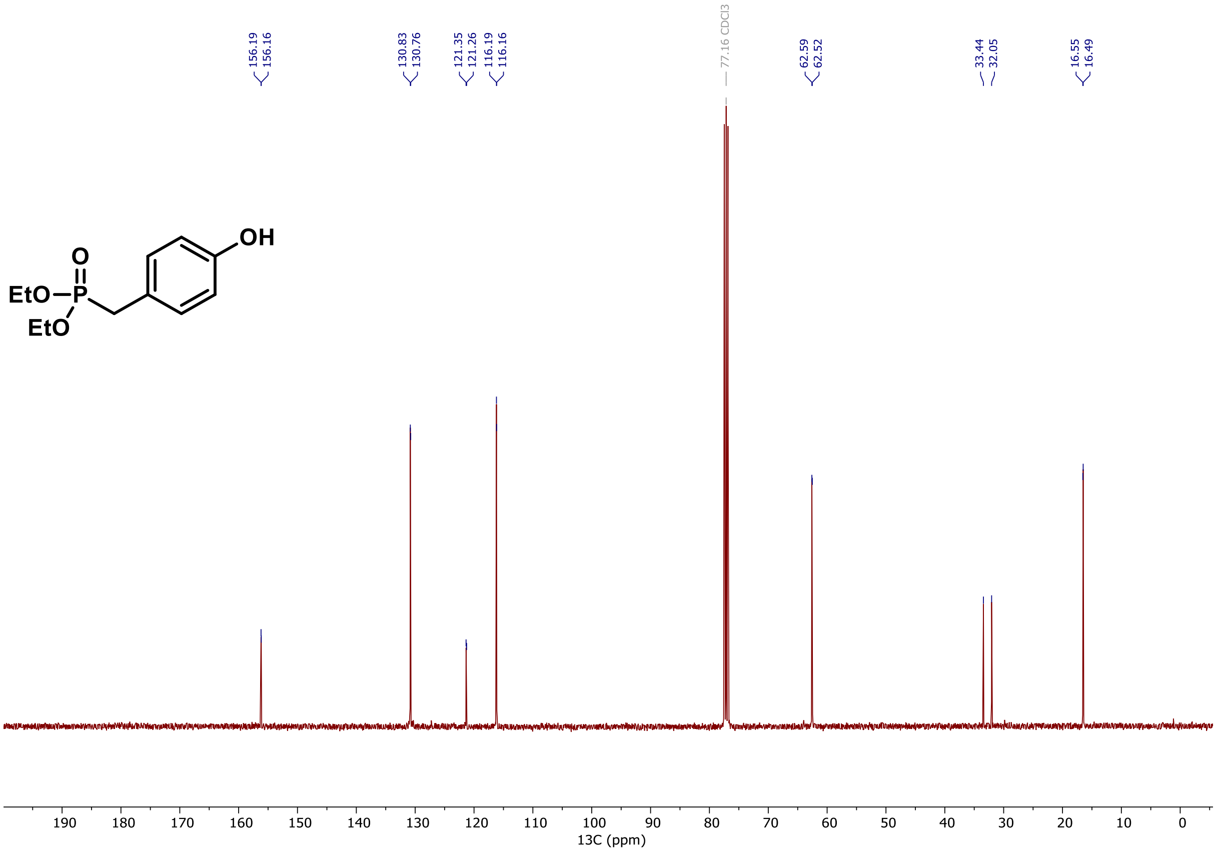

^{1}H NMR (400 MHz, $CDCl_3$) of compound **35.**

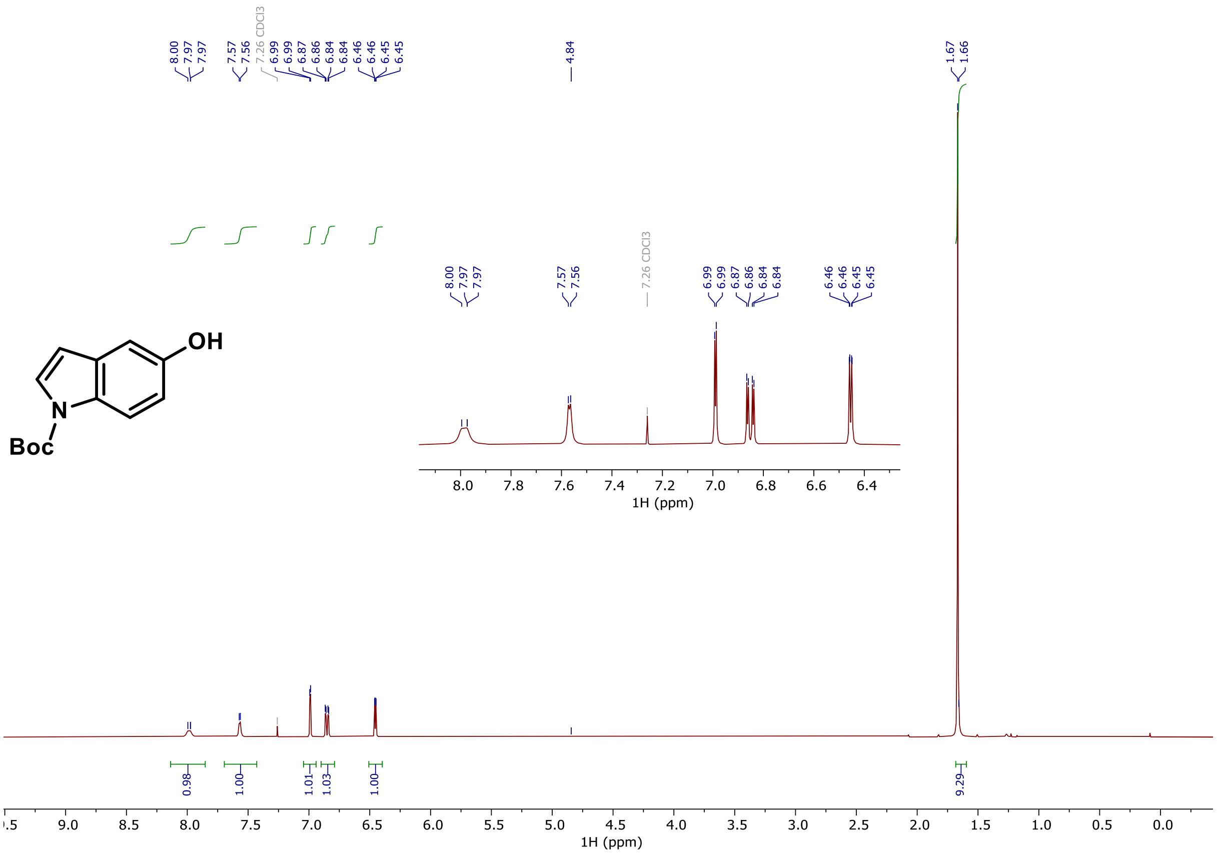

^{13}C NMR (101 MHz, $CDCl_3$) of compound **35.**

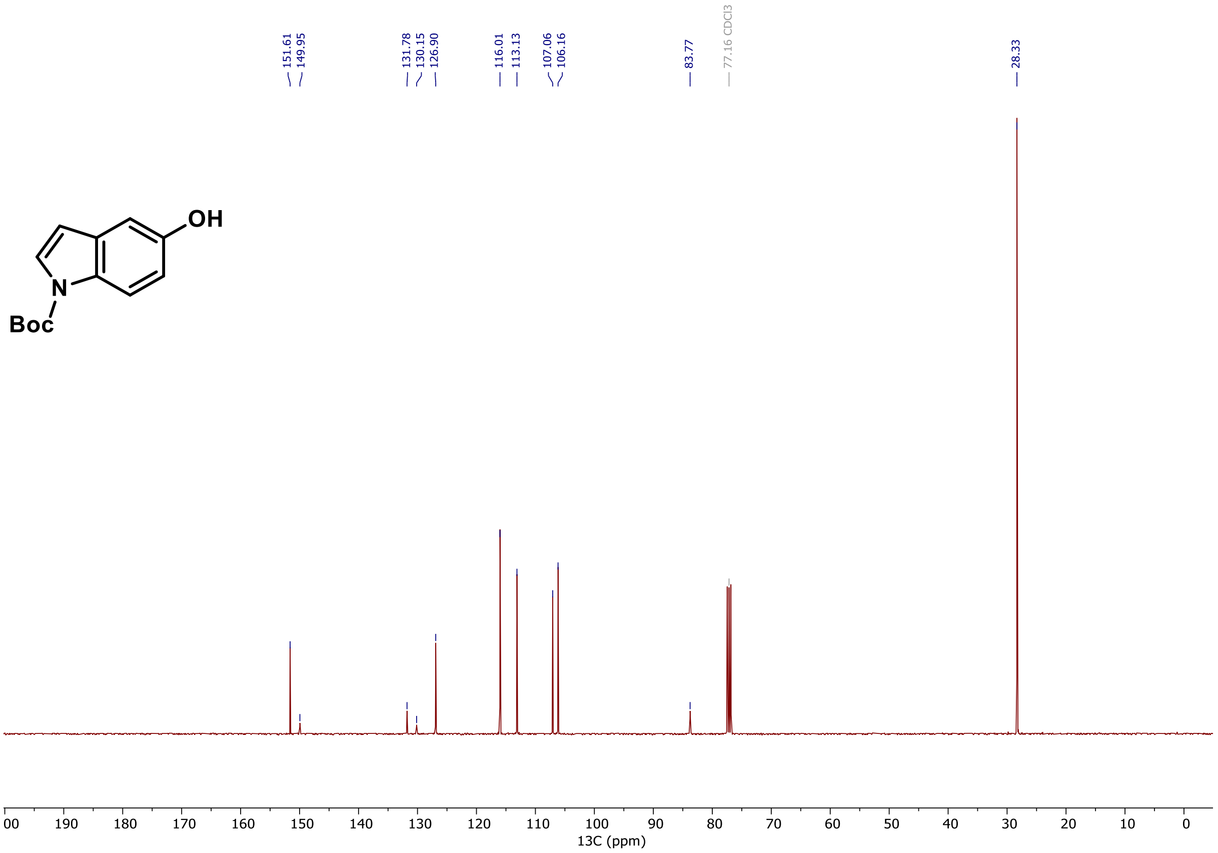

^{1}H NMR (400 MHz, DMSO-d_6) of compound **36.**

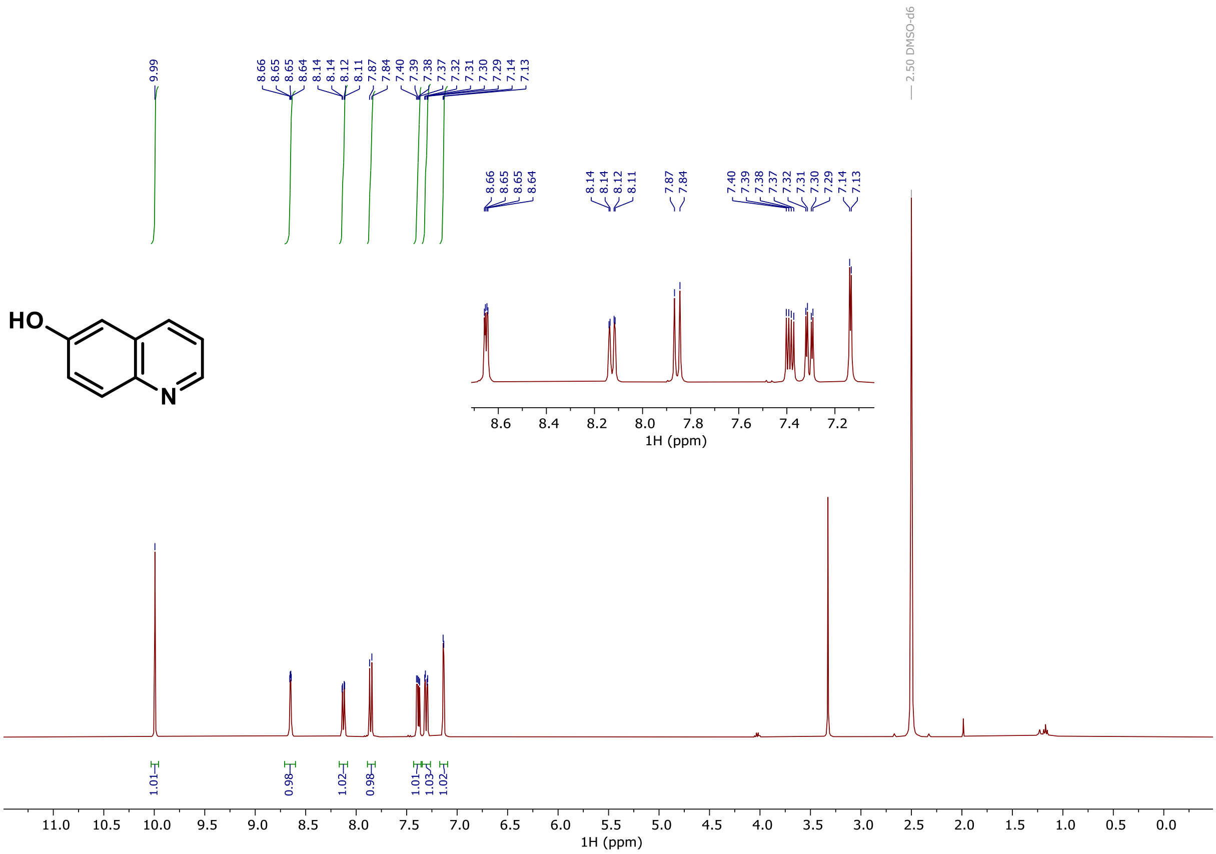

^{13}C NMR (101 MHz, DMSO-d_6) of compound **36.**

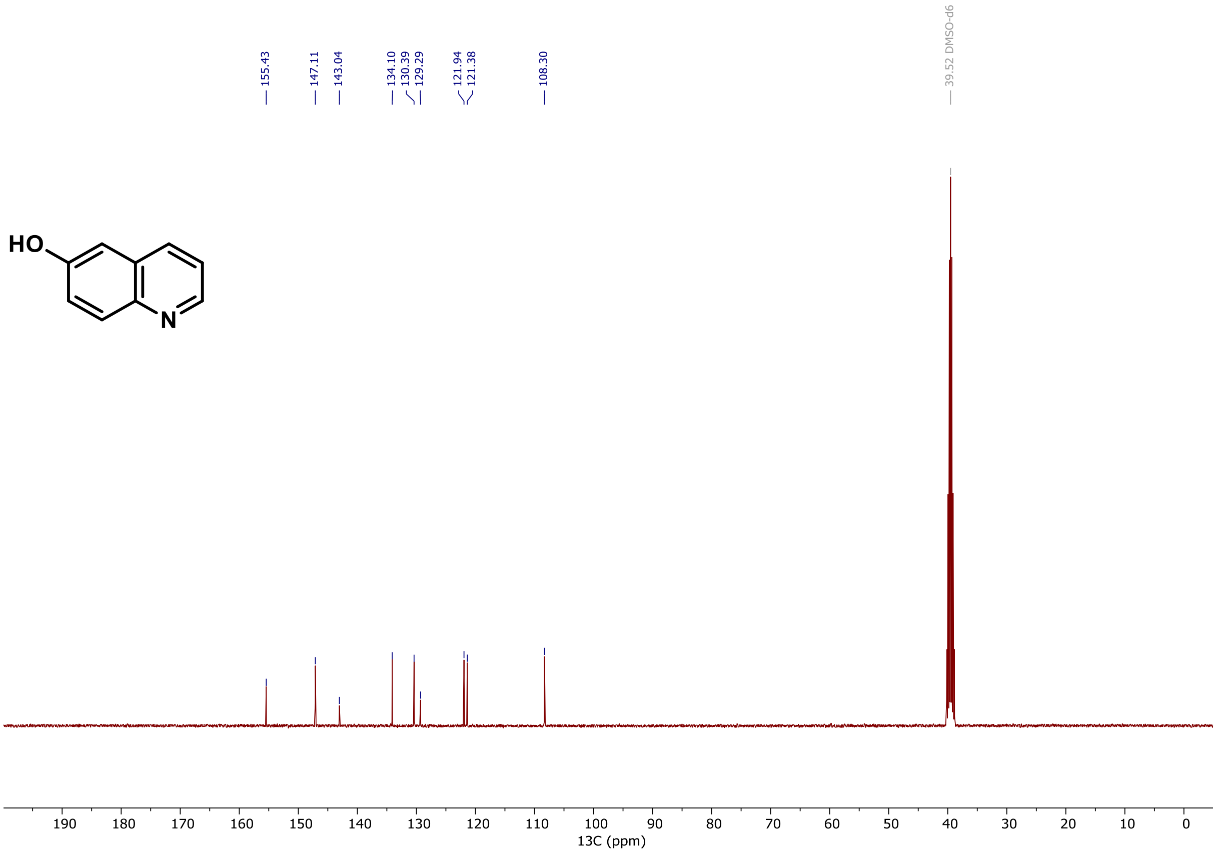

^{1}H NMR (300 MHz, $CDCl_3$) of compound **37.**

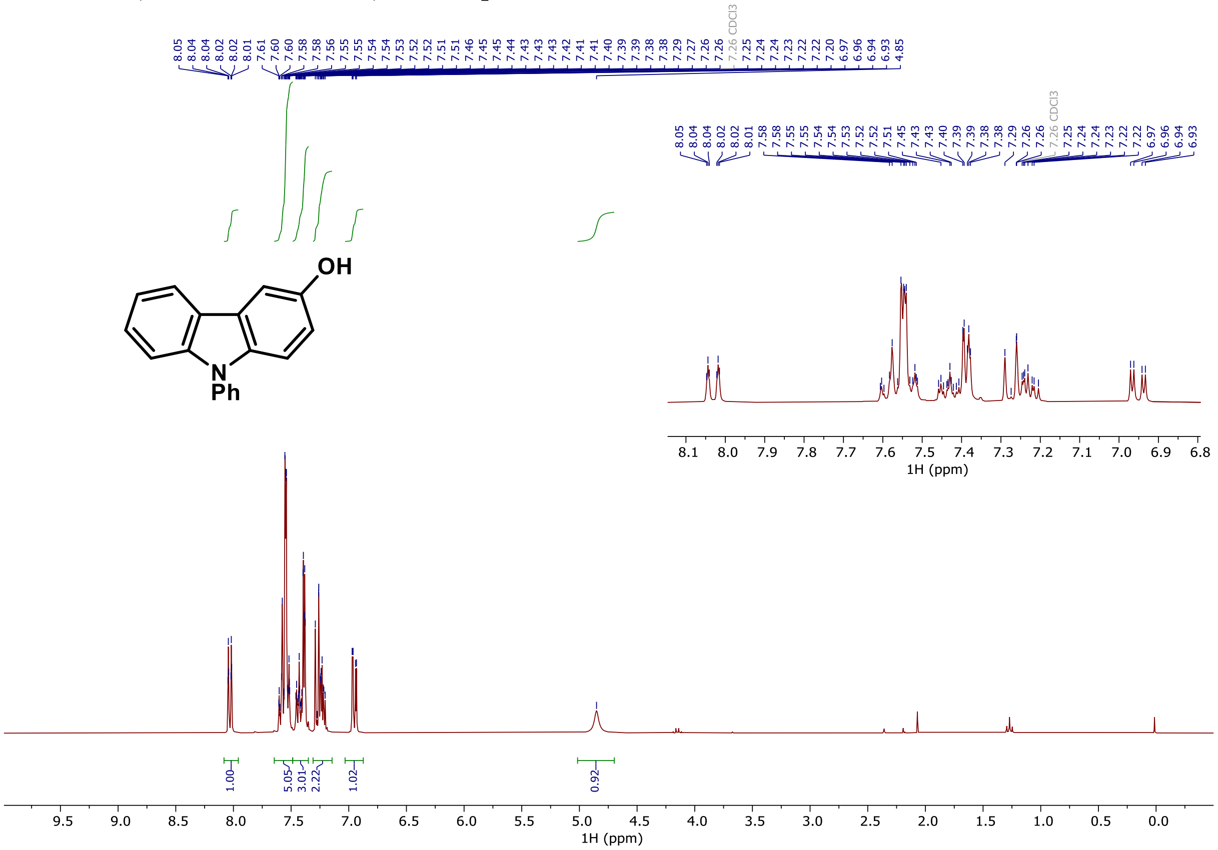

^{13}C NMR (75 MHz, $CDCl_3$) of compound **37.**

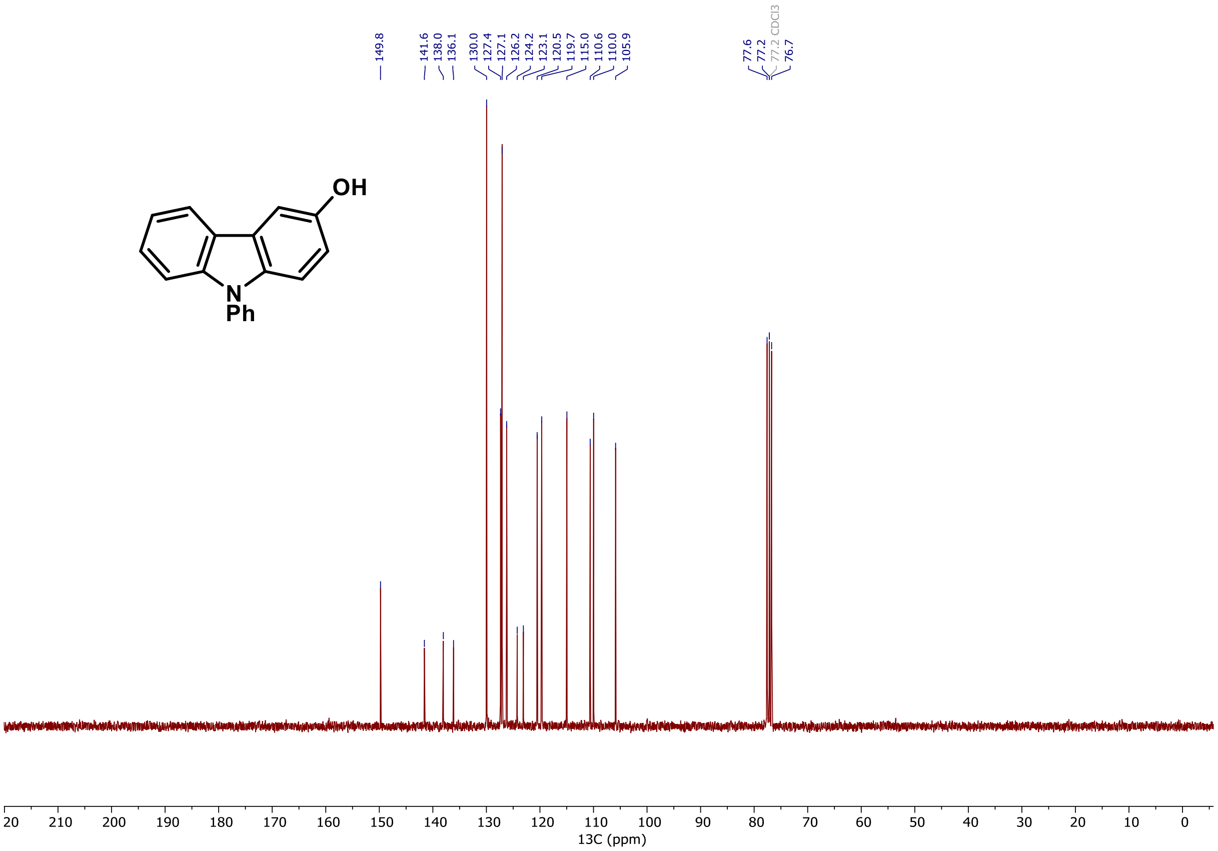

^{1}H NMR (300 MHz, DMSO-d_6) of compound **38.**

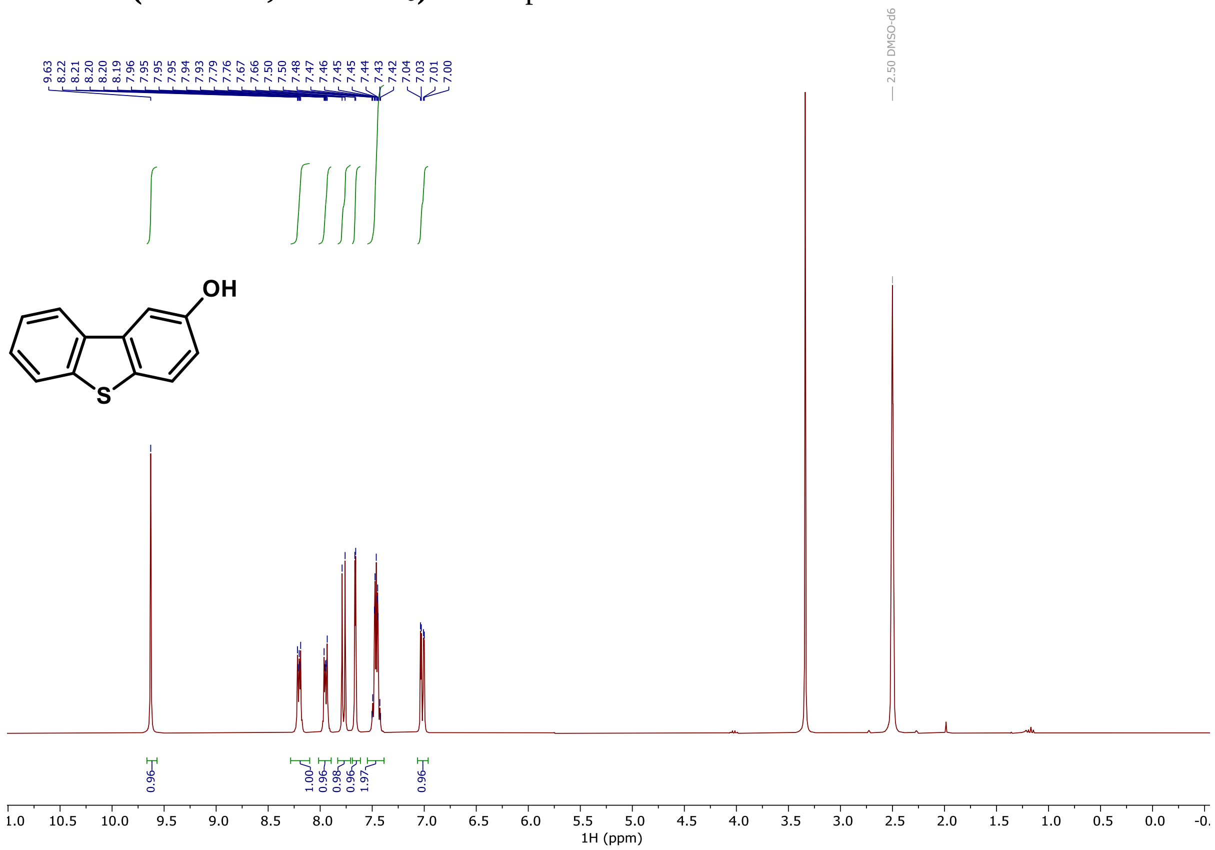

^{13}C NMR (75 MHz, DMSO-d_6) of compound **38.**

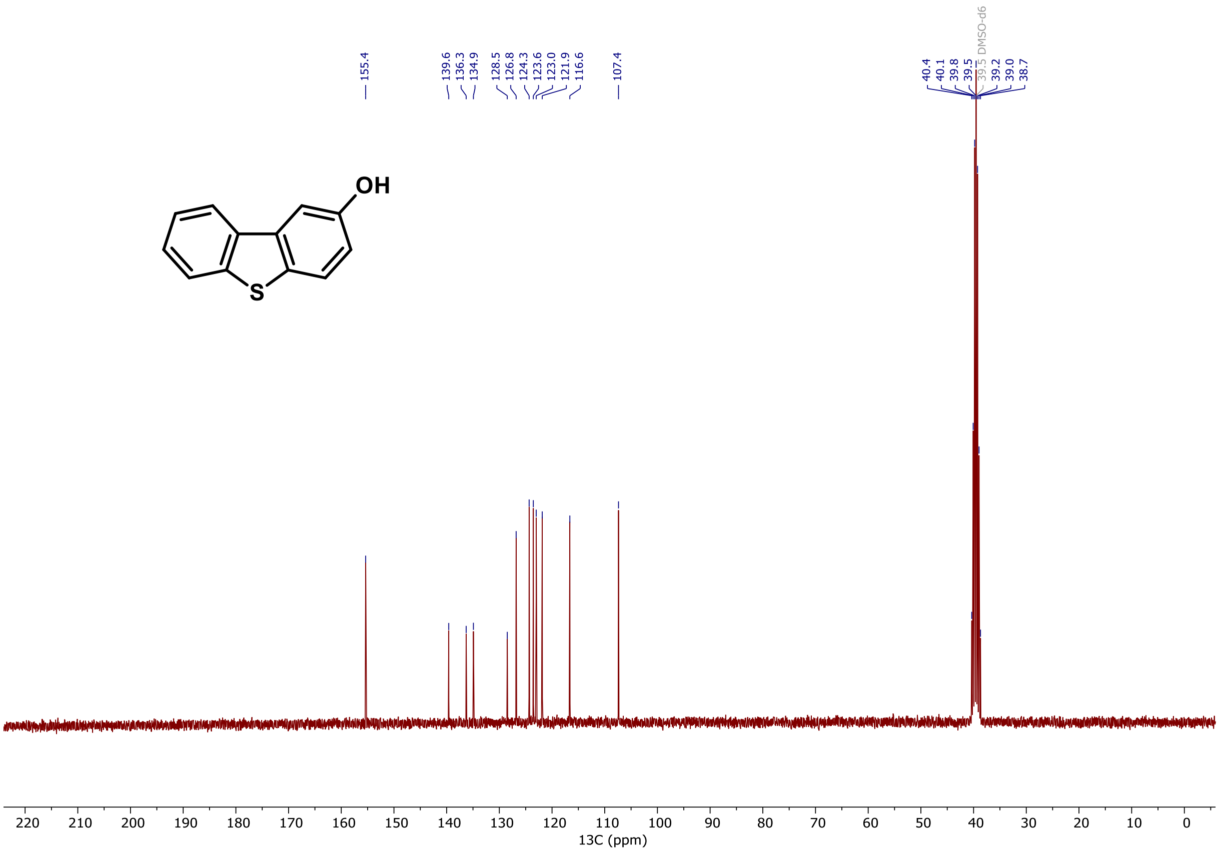

^{1}H NMR (400 MHz, CDCl$_3$) of compound **39.**

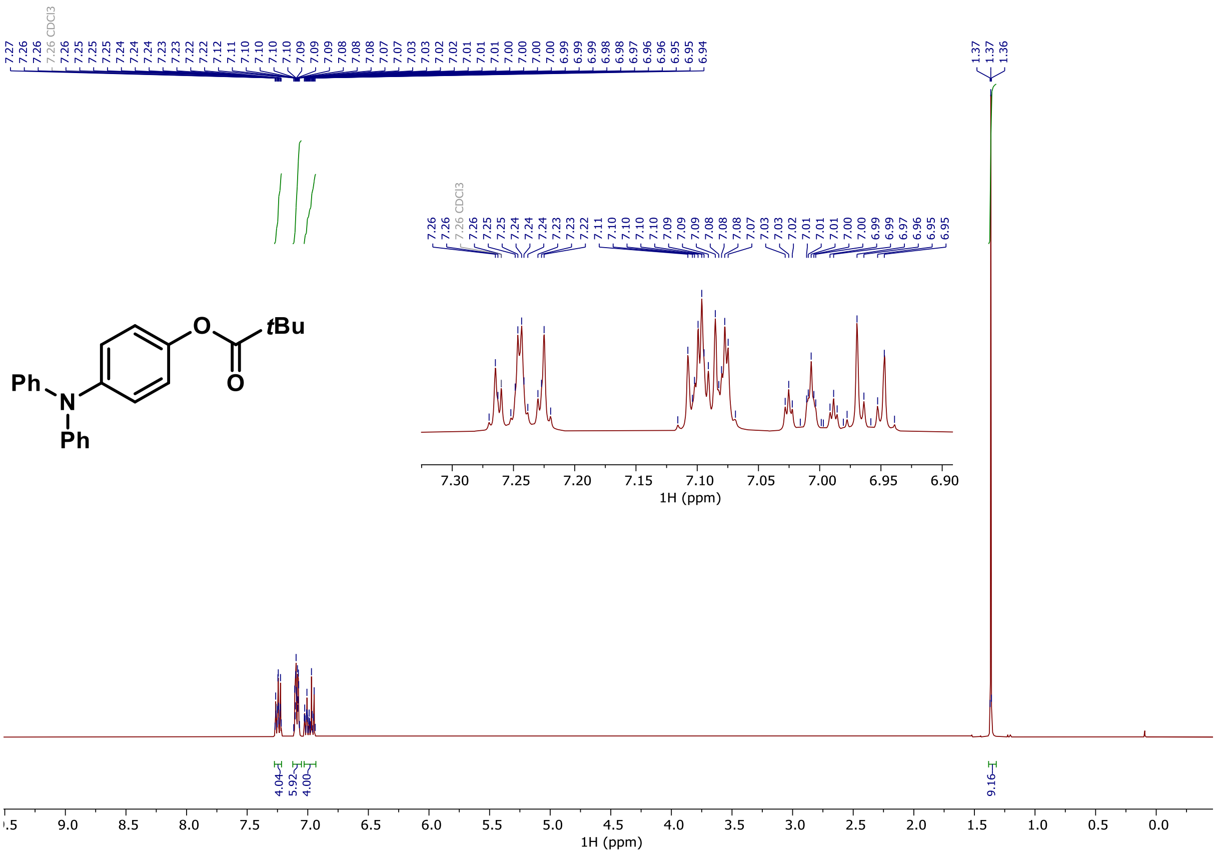

^{13}C NMR (101 MHz, CDCl$_3$) of compound **39.**

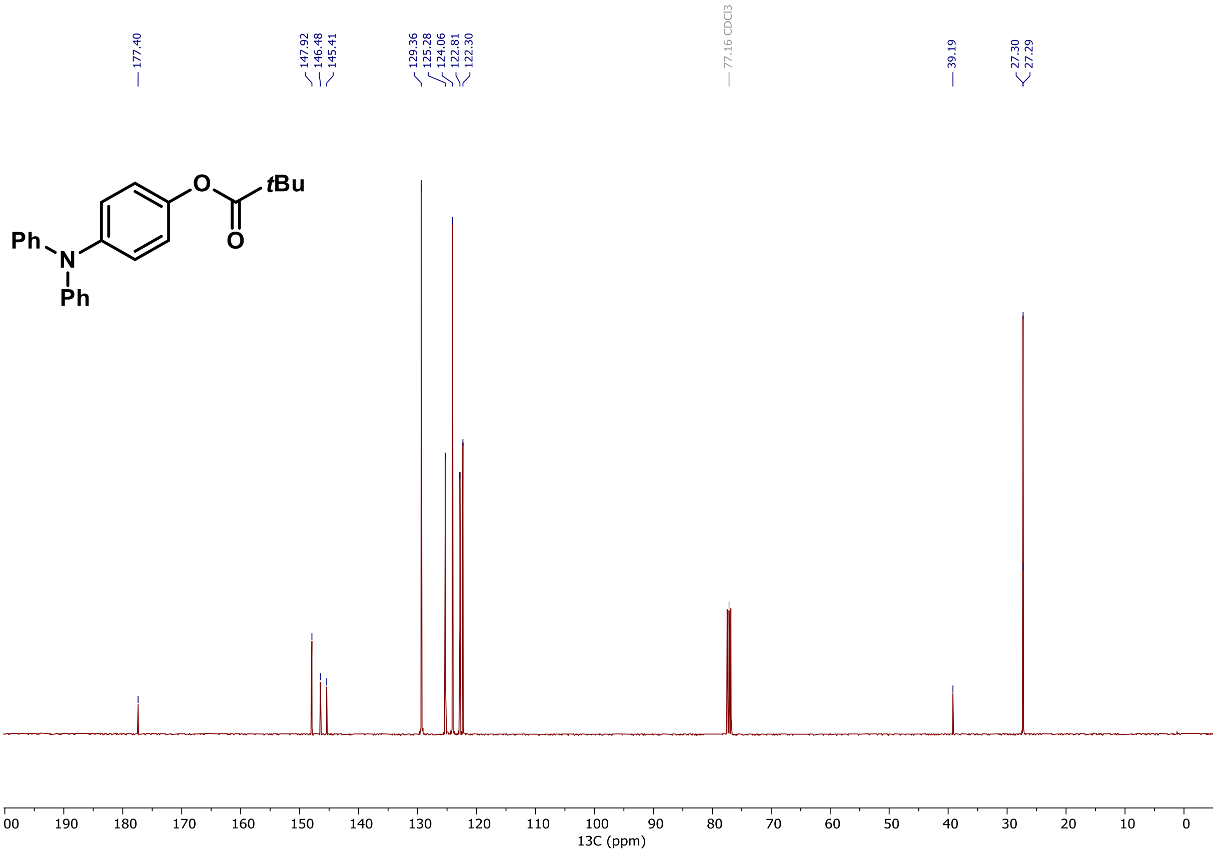

^{1}H NMR (300 MHz, $CDCl_3$) of compound **40.**

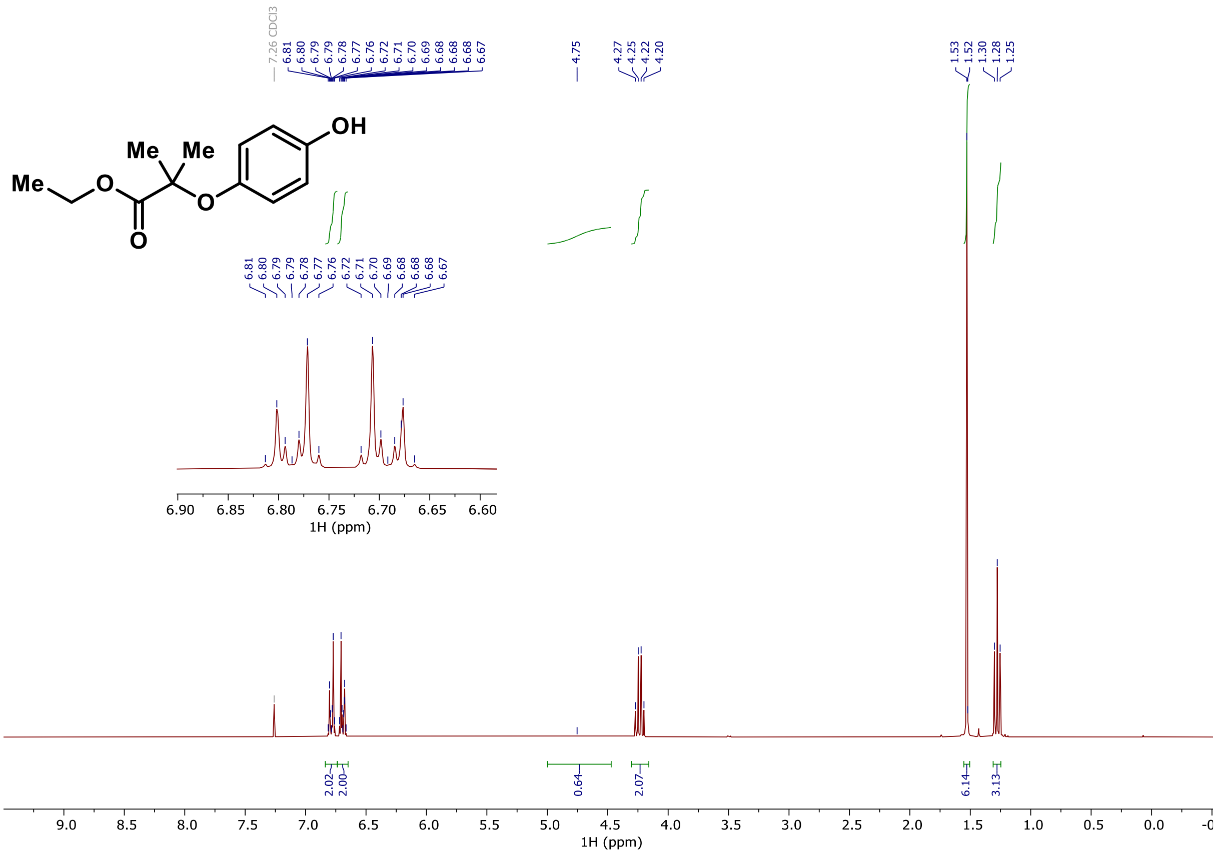

^{13}C NMR (75 MHz, $CDCl_3$) of compound **40.**

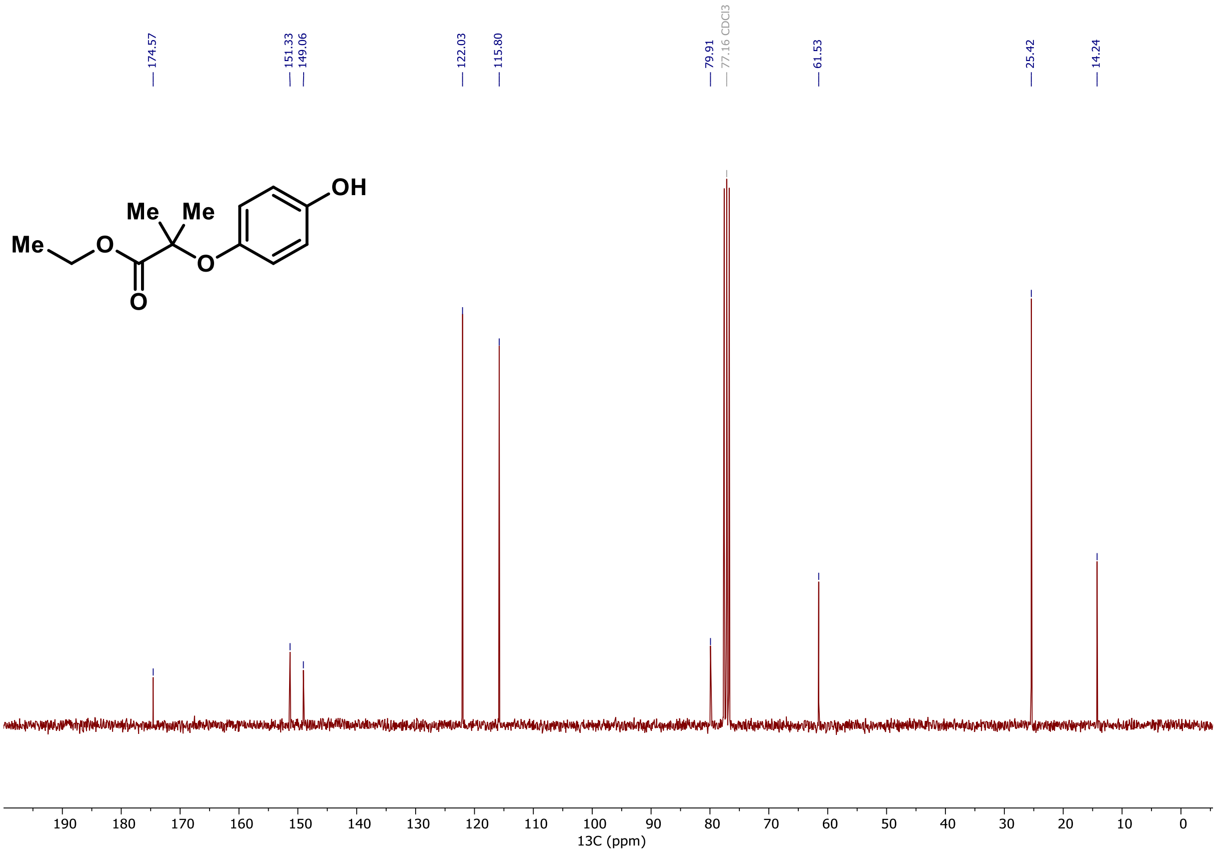

^{1}H NMR (400 MHz, CDCl$_3$) of compound **41.**

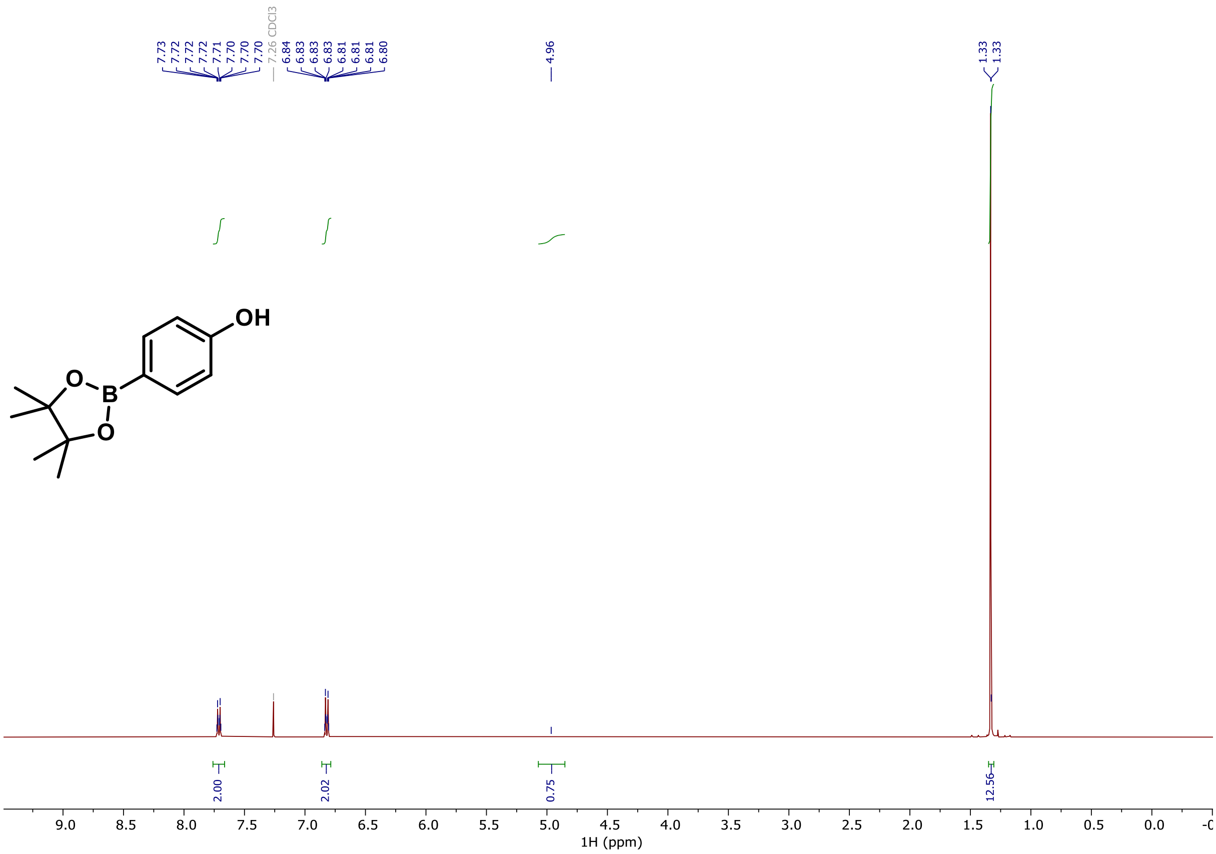

^{13}C NMR (101 MHz, CDCl$_3$) of compound **41.**

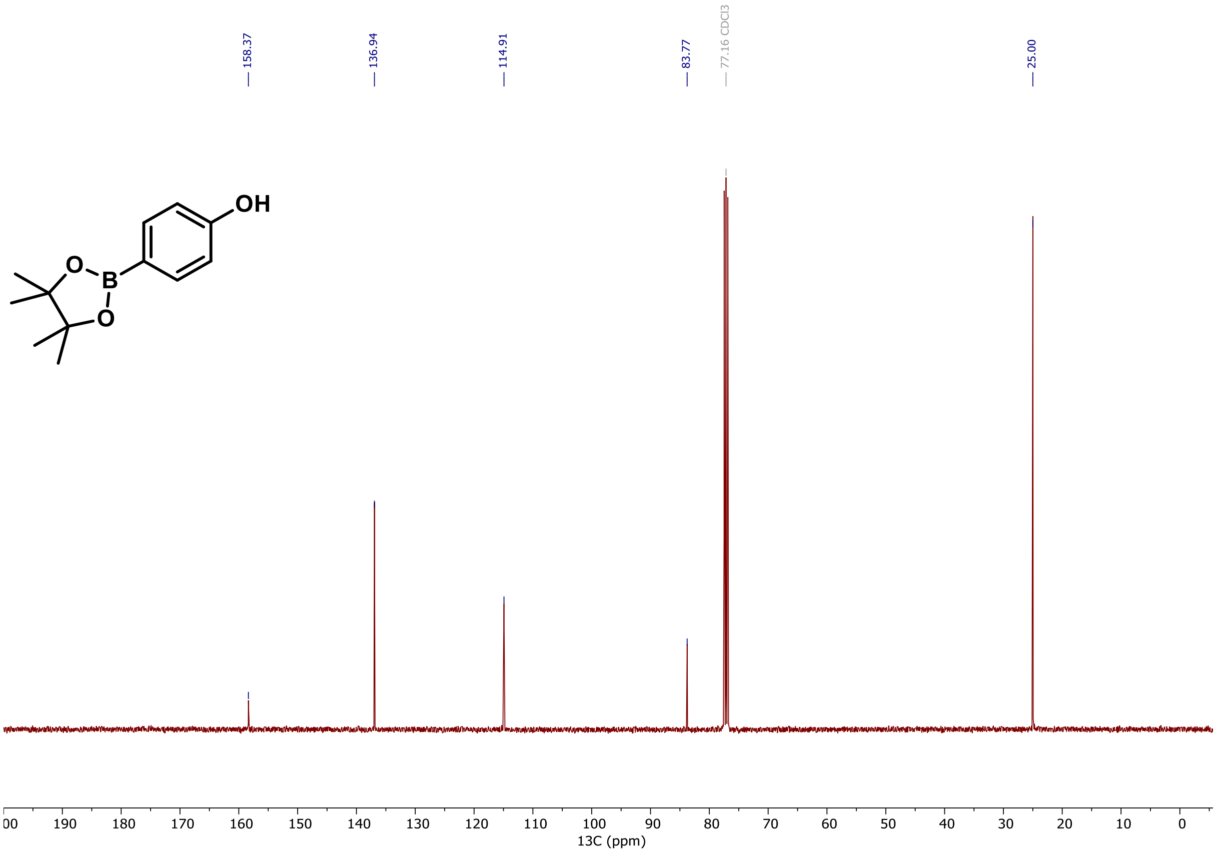

^{1}H NMR (400 MHz, DMSO-*d_6*) of compound **42.**

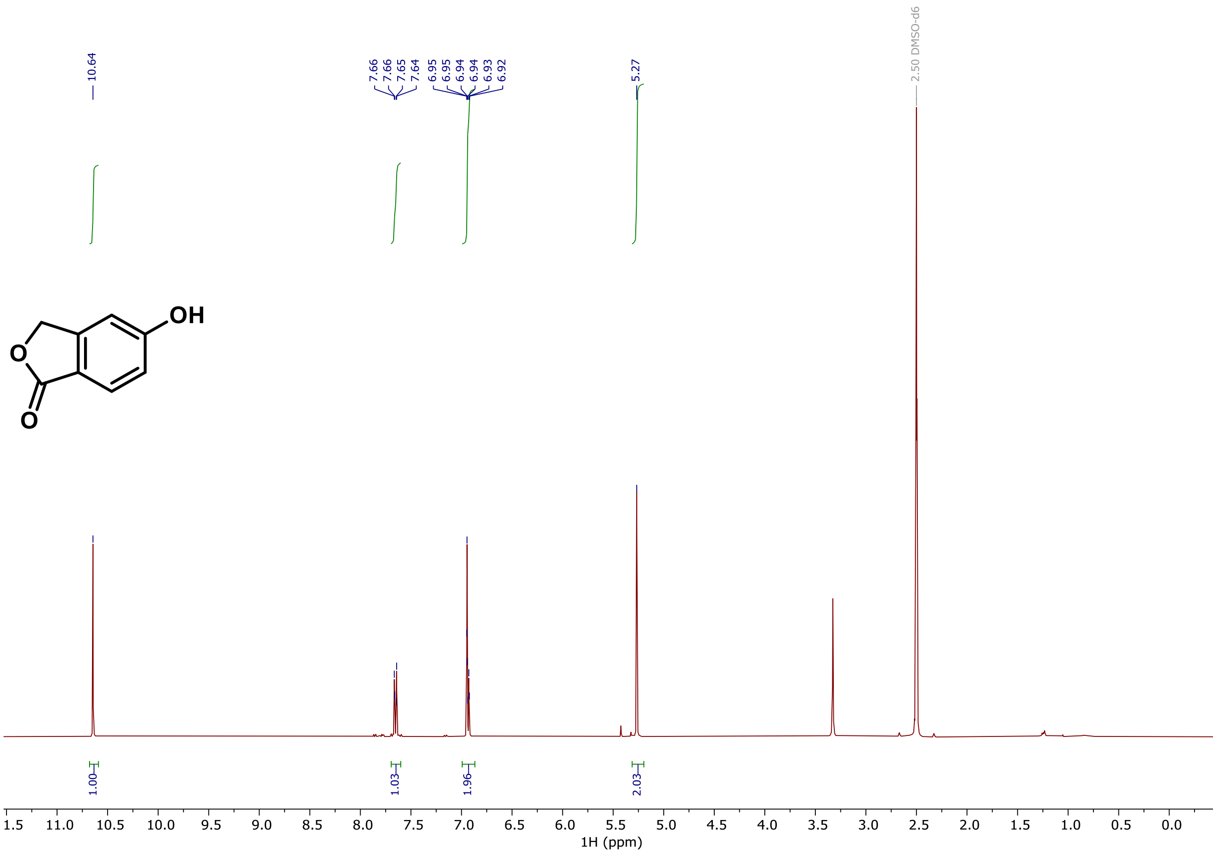

^{13}C NMR (101 MHz, DMSO-*d_6*) of compound **42.**

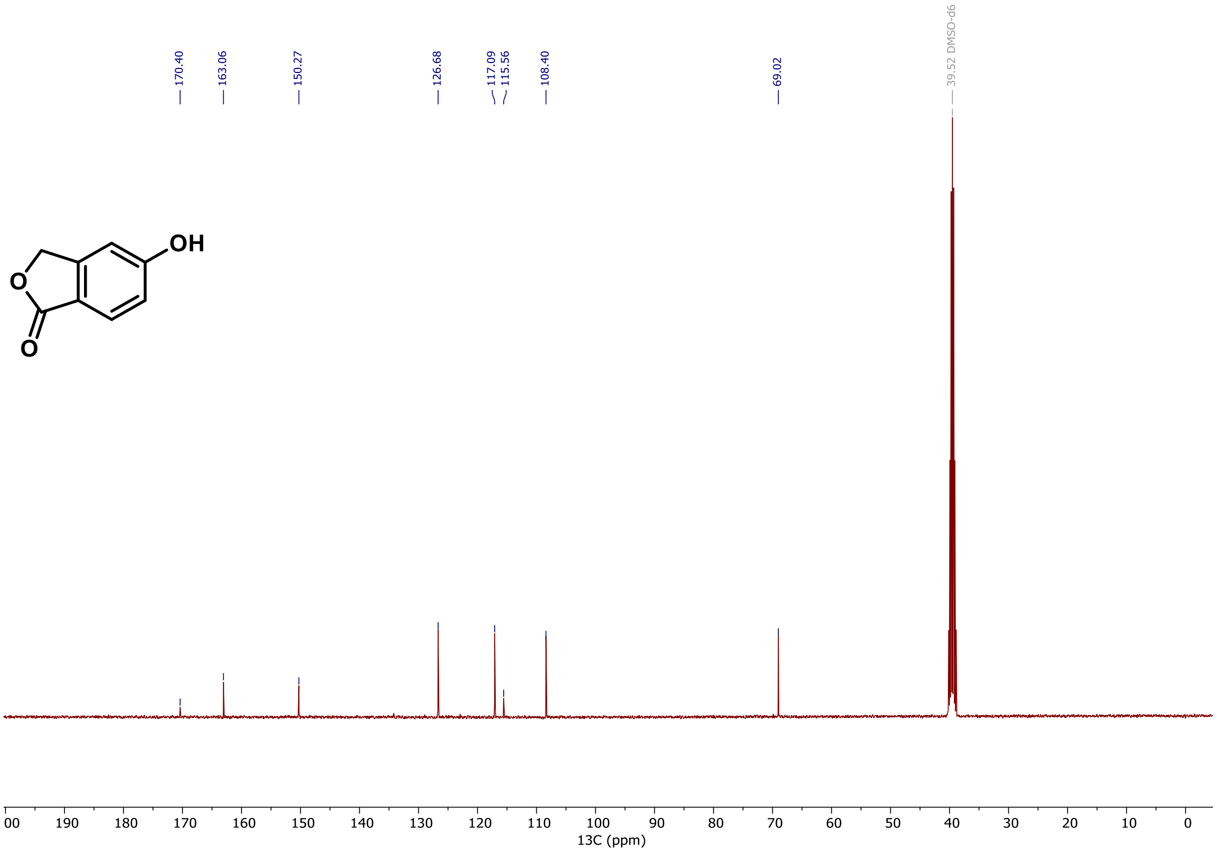

^{1}H NMR (300 MHz, $CDCl_3$) of compound **43.**

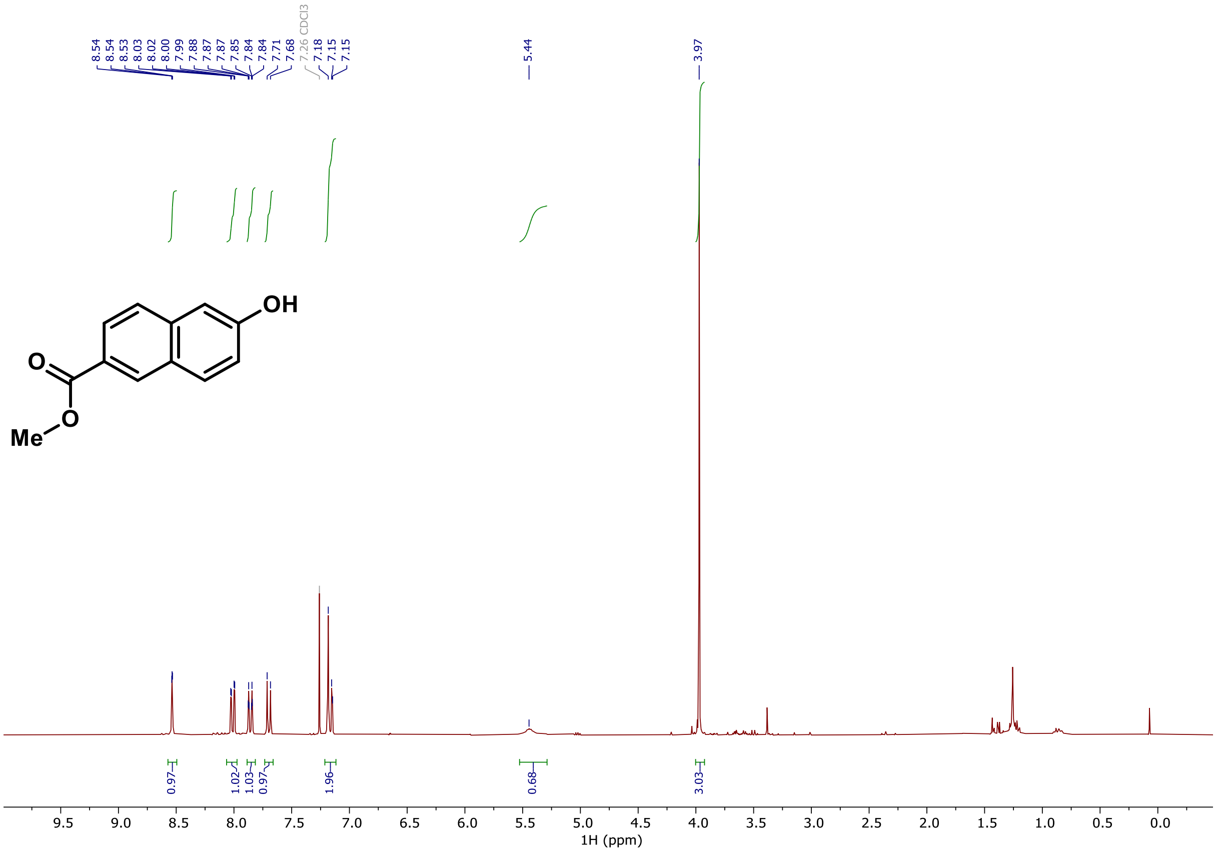

^{13}C NMR (75 MHz, $CDCl_3$) of compound **43.**

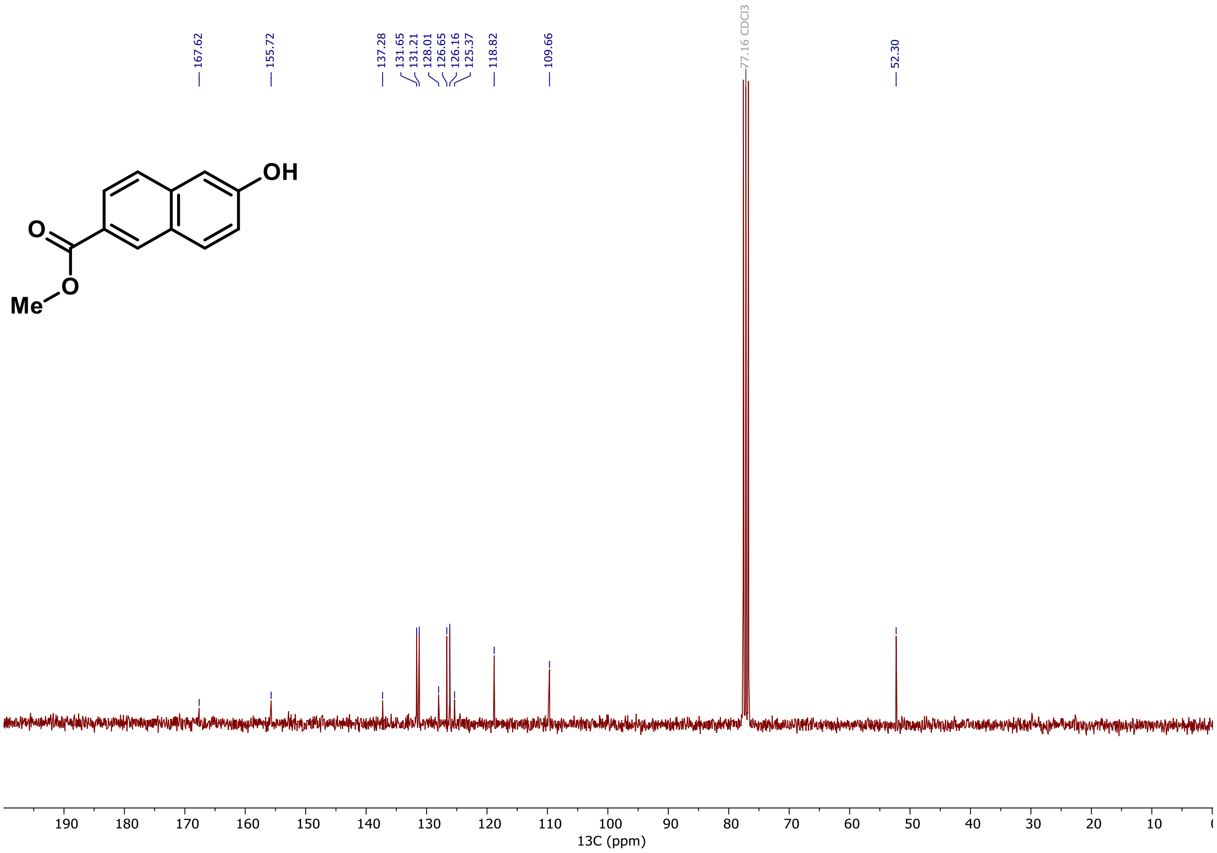

^{1}H NMR (400 MHz, $CDCl_3$) of compound **44.**

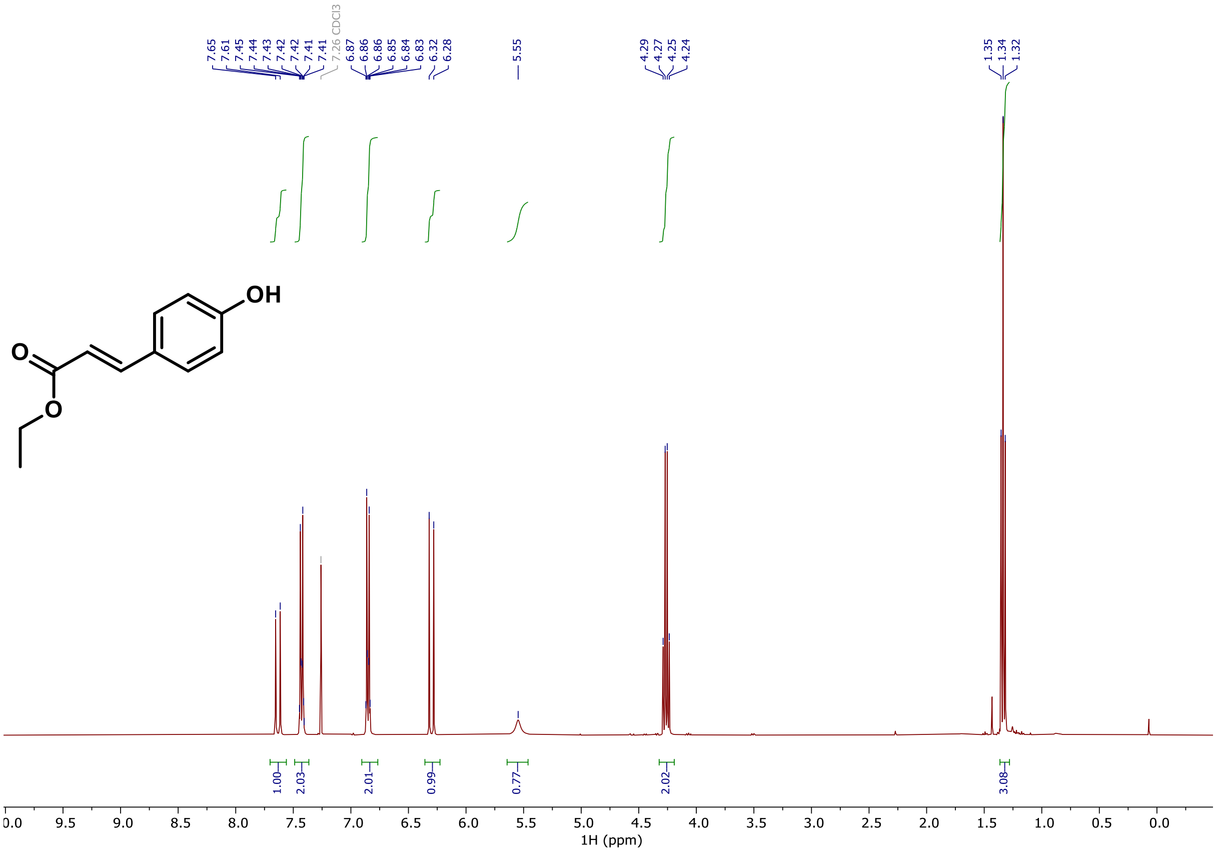

^{13}C NMR (101 MHz, $CDCl_3$) of compound **44.**

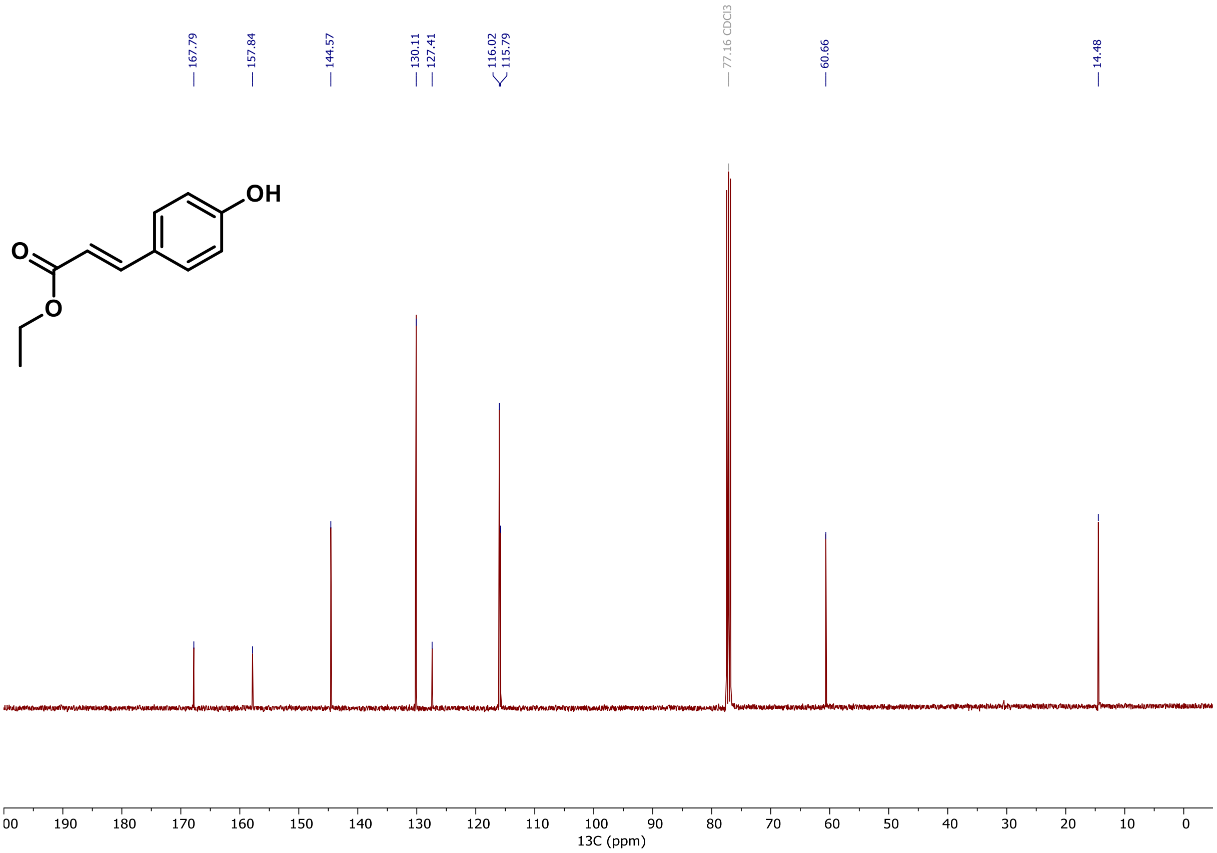

^{1}H NMR (400 MHz, CDCl$_3$) of compound **45.**

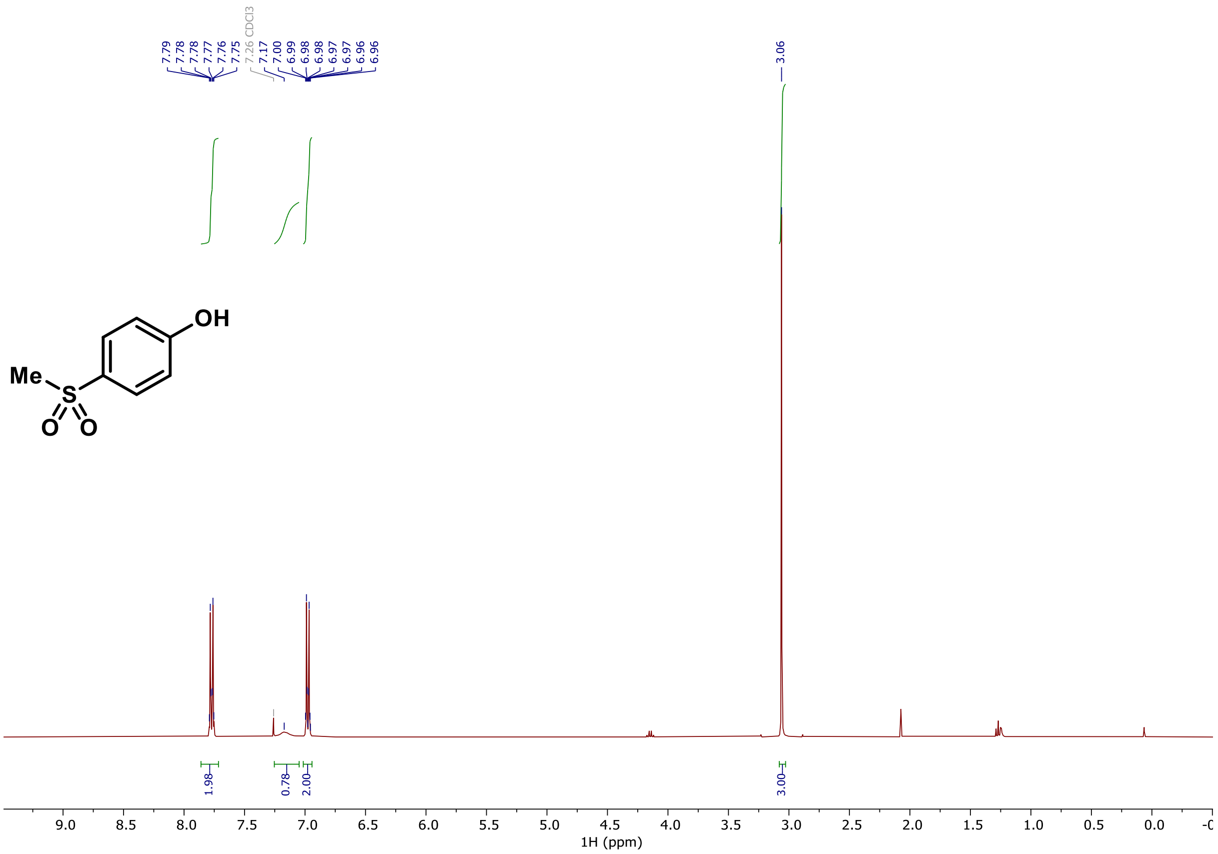

^{13}C NMR (101 MHz, CDCl$_3$) of compound **45.**

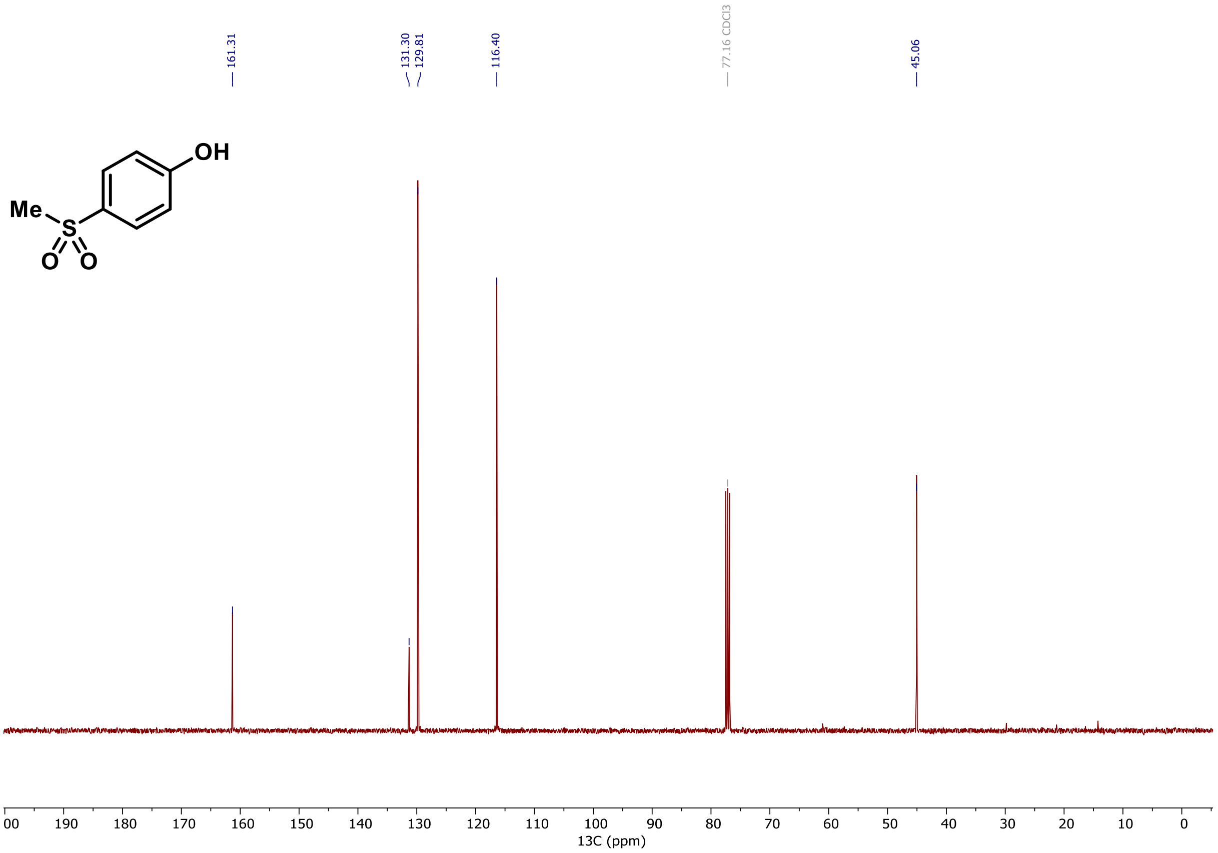

^{1}H NMR (400 MHz, CDCl3) of compound 46.

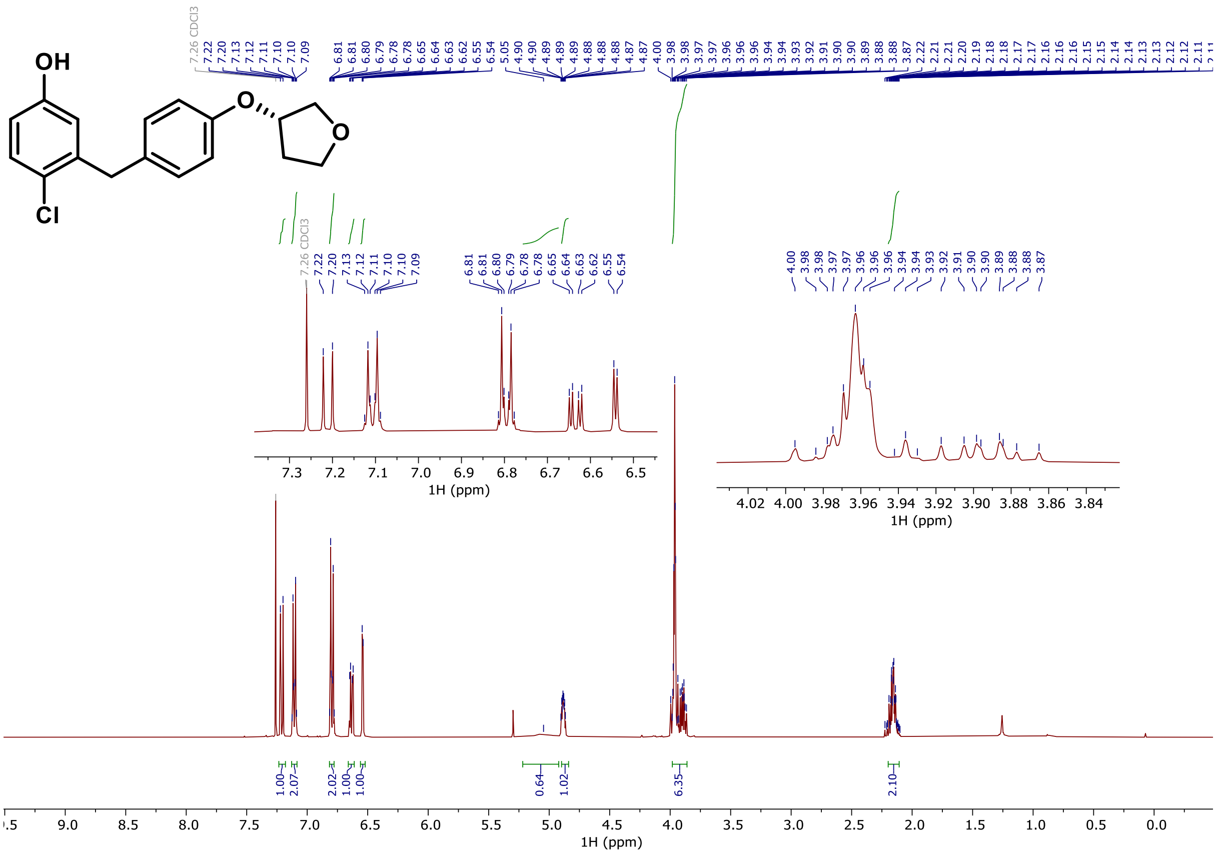

^{13}C NMR (101 MHz, CDCl3) of compound 46.

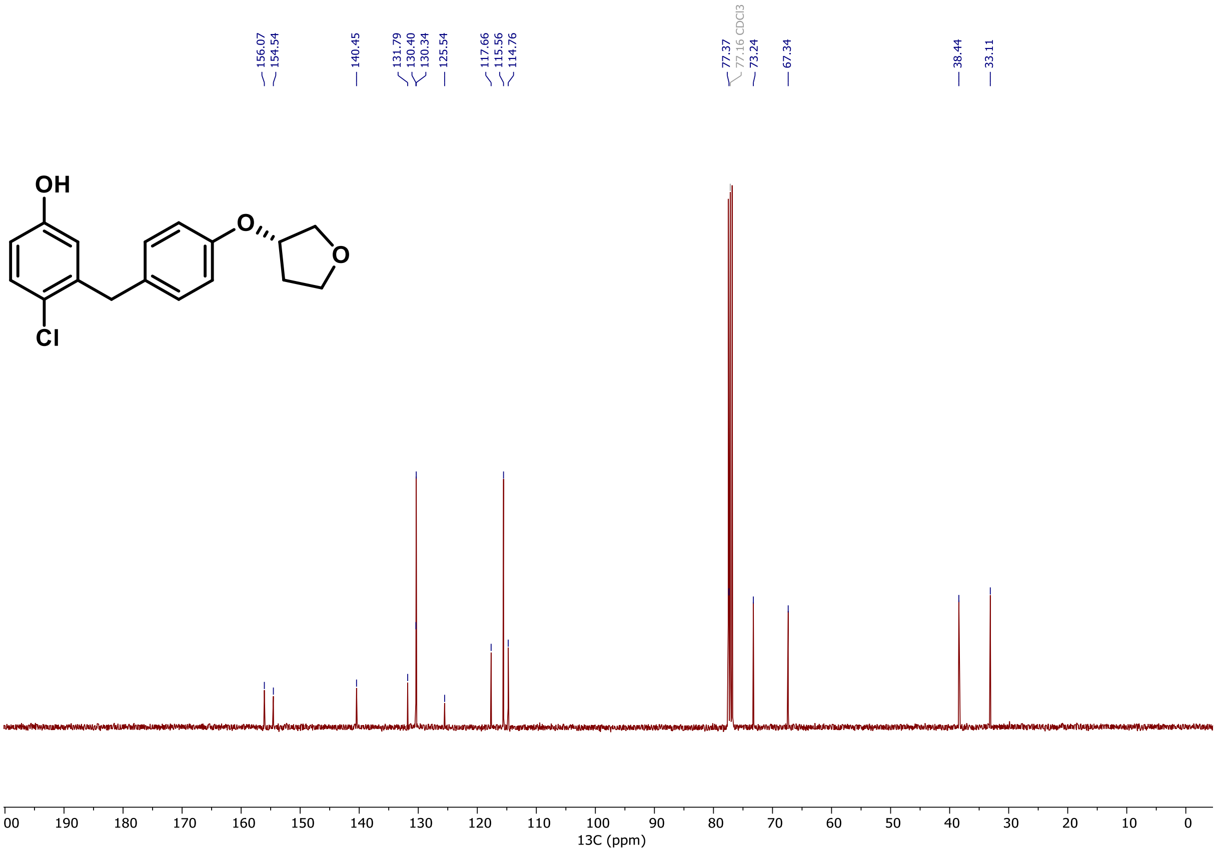

^{1}H NMR (400 MHz, CDCl$_3$) of compound **47.**

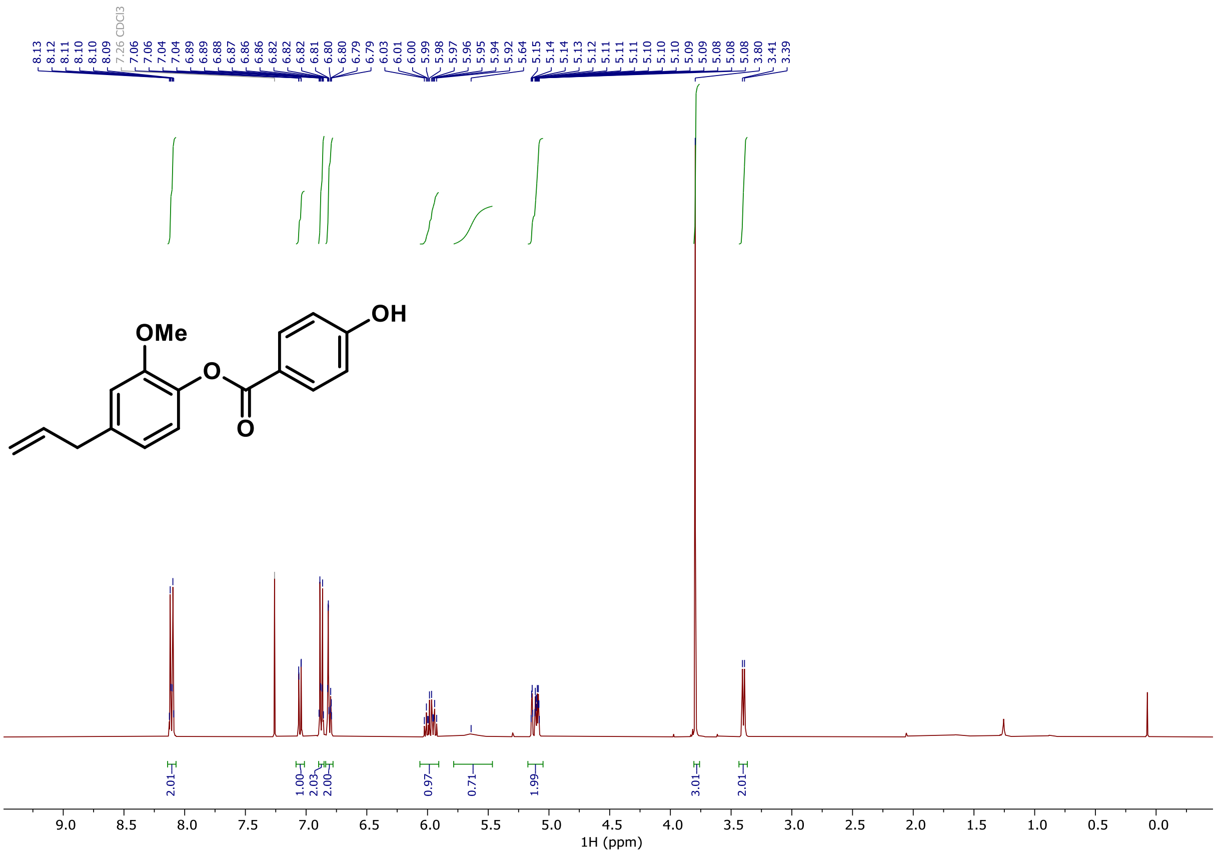

^{13}C NMR (101 MHz, CDCl$_3$) of compound **47.**

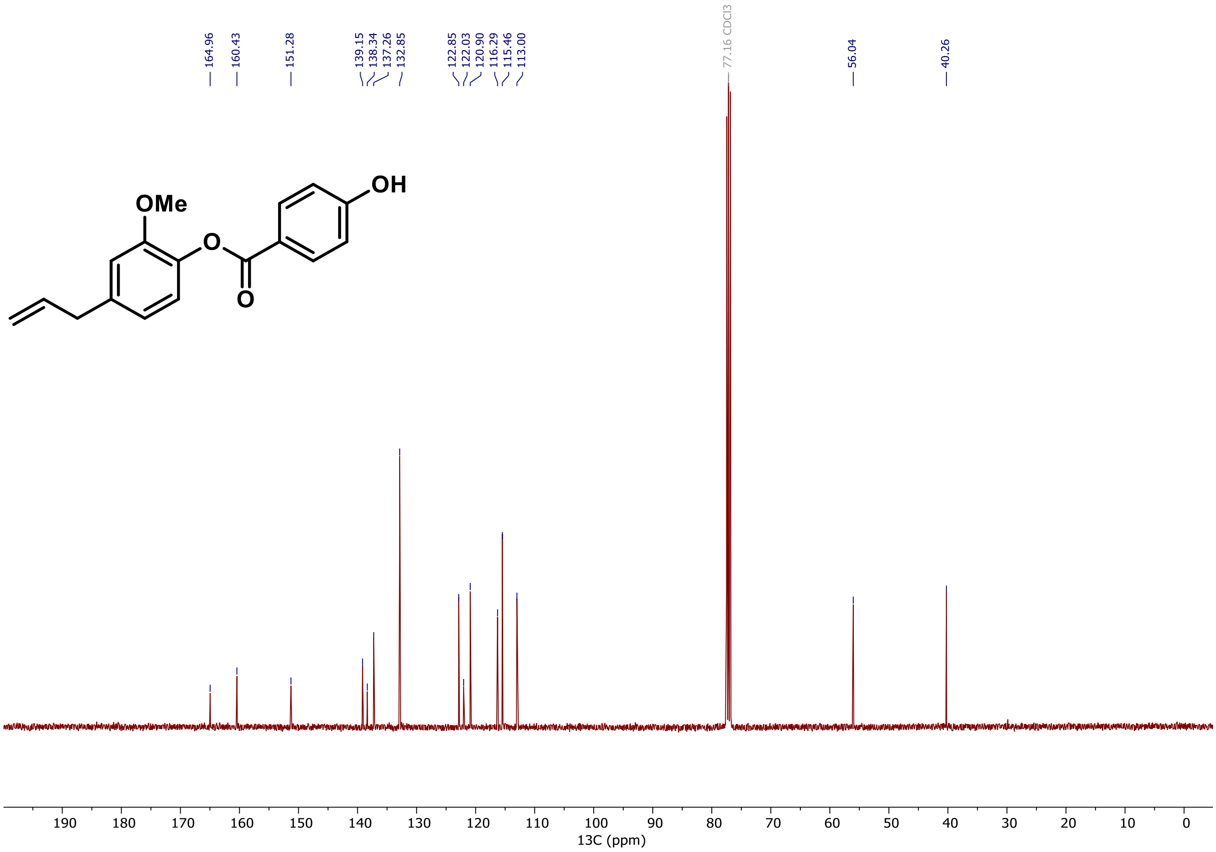

^{1}H NMR (400 MHz, DMSO-d_6) of compound **48.**

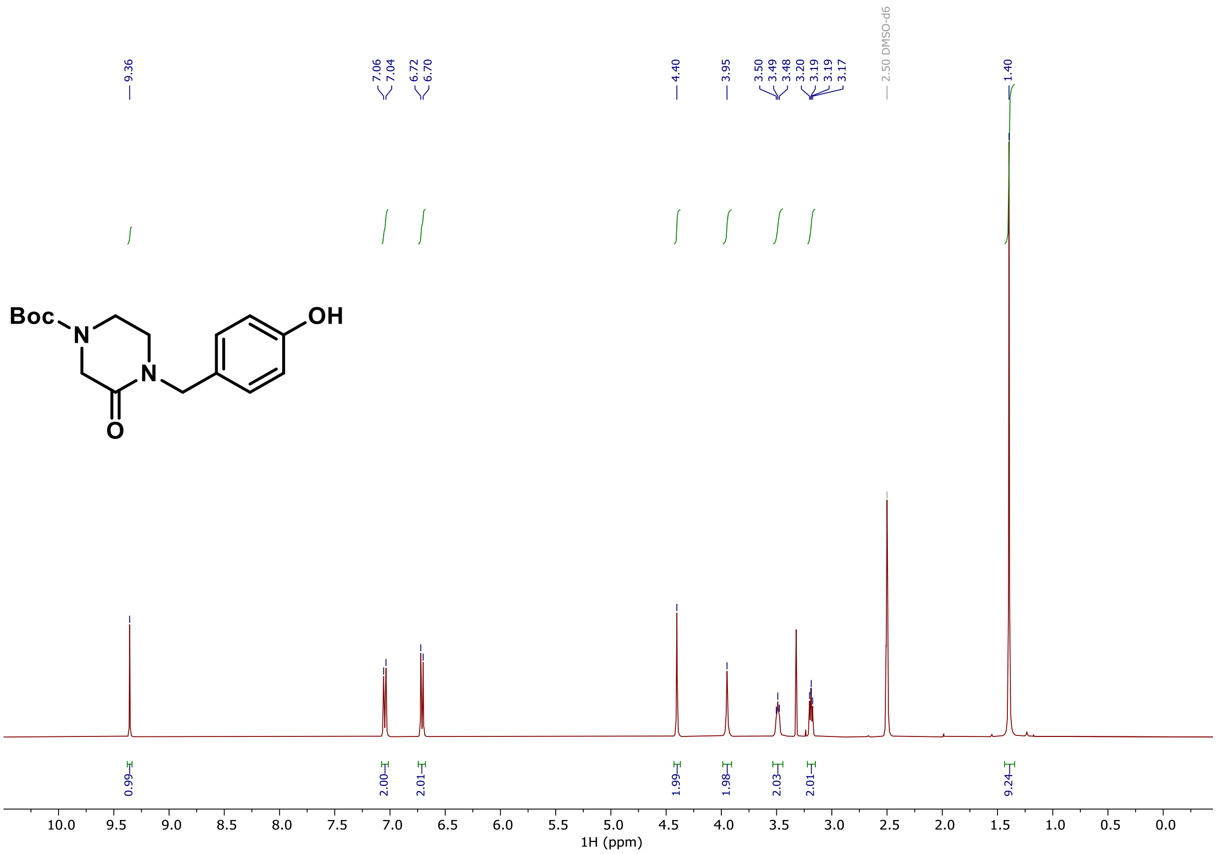

^{13}C NMR (101 MHz, DMSO-d_6) of compound **48.**

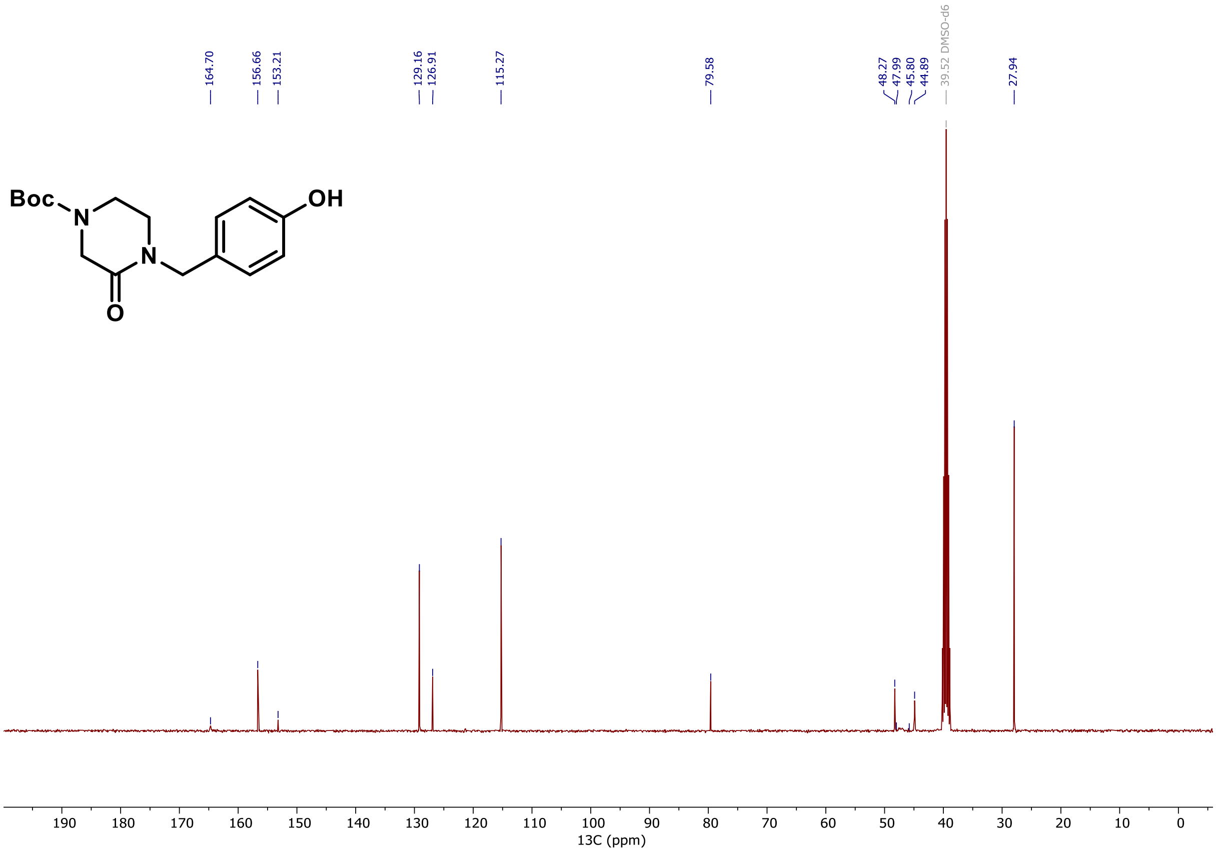

^{1}H NMR (400 MHz, $CDCl_3$) of compound **49.**

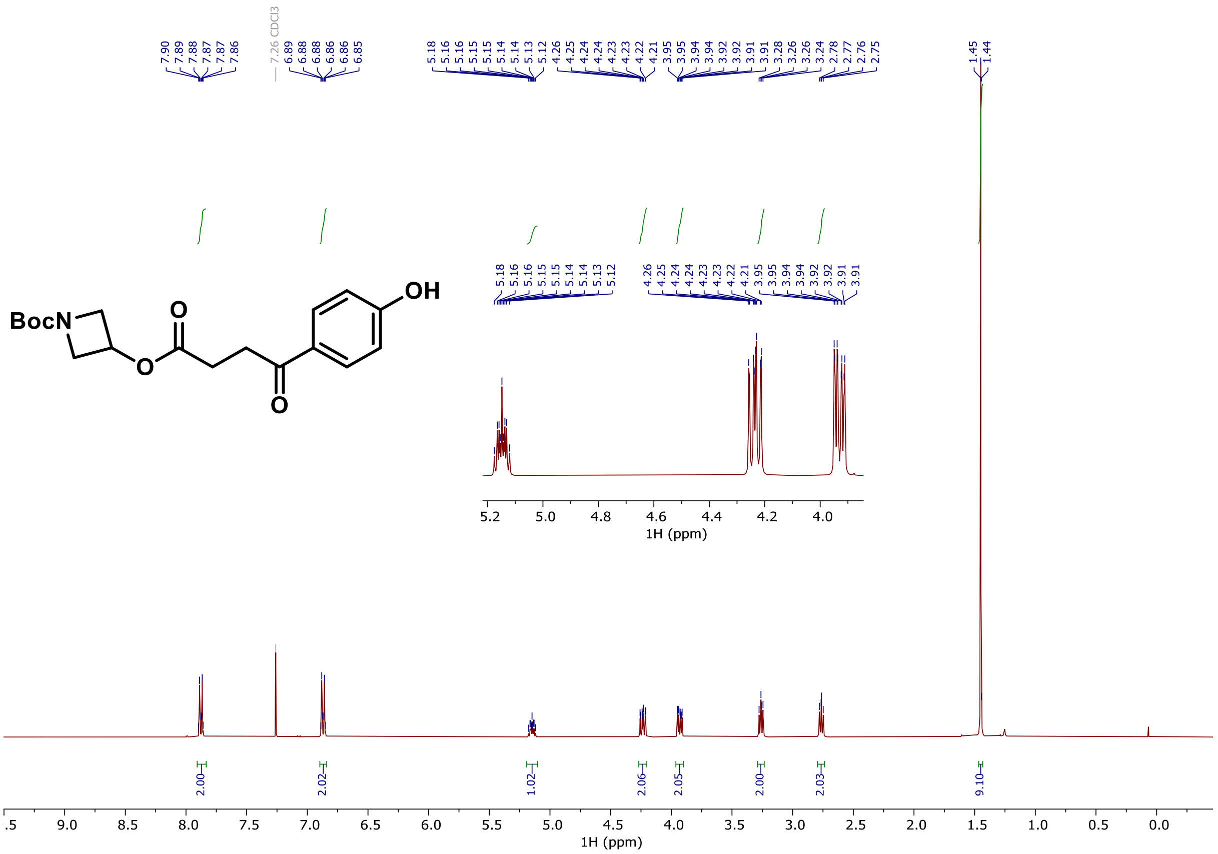

^{13}C NMR (101 MHz, $CDCl_3$) of compound **49.**

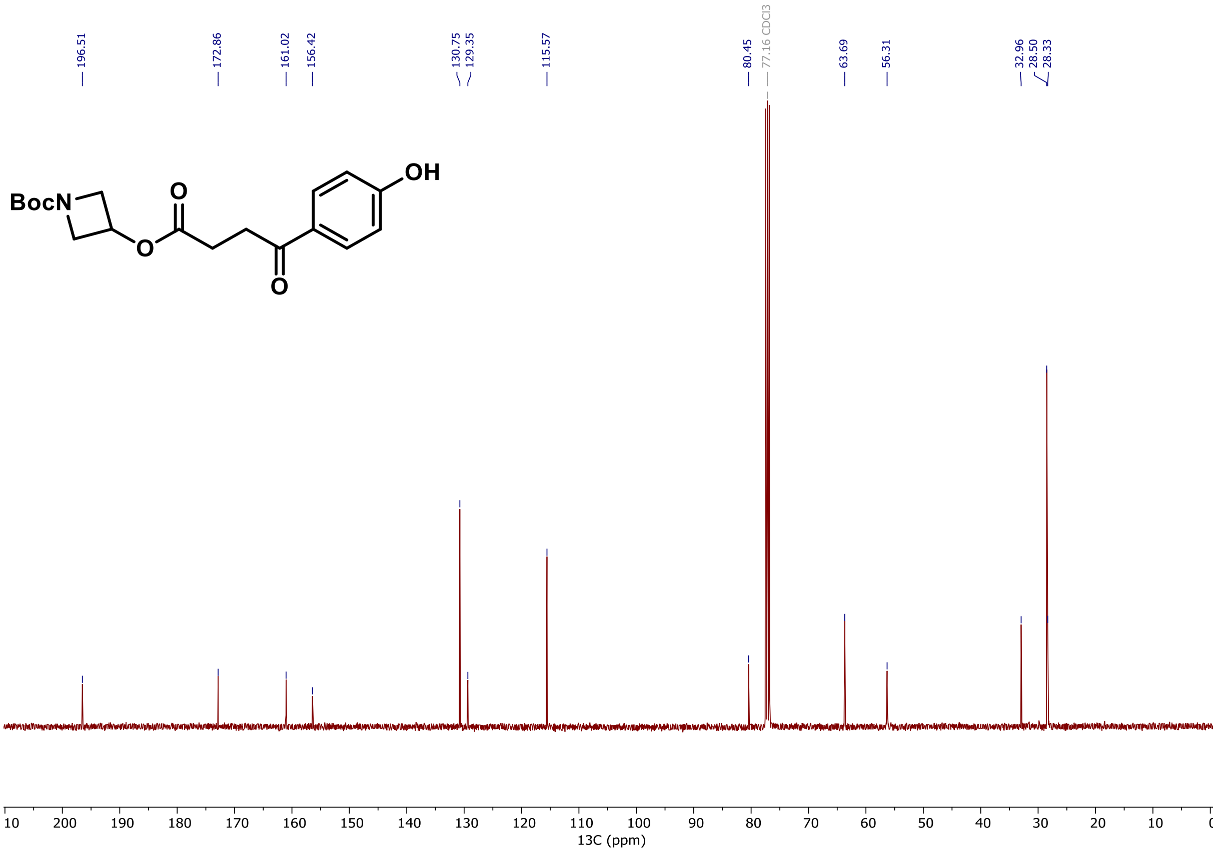

^{1}H NMR (400 MHz, CDCl$_3$) of compound **50.**

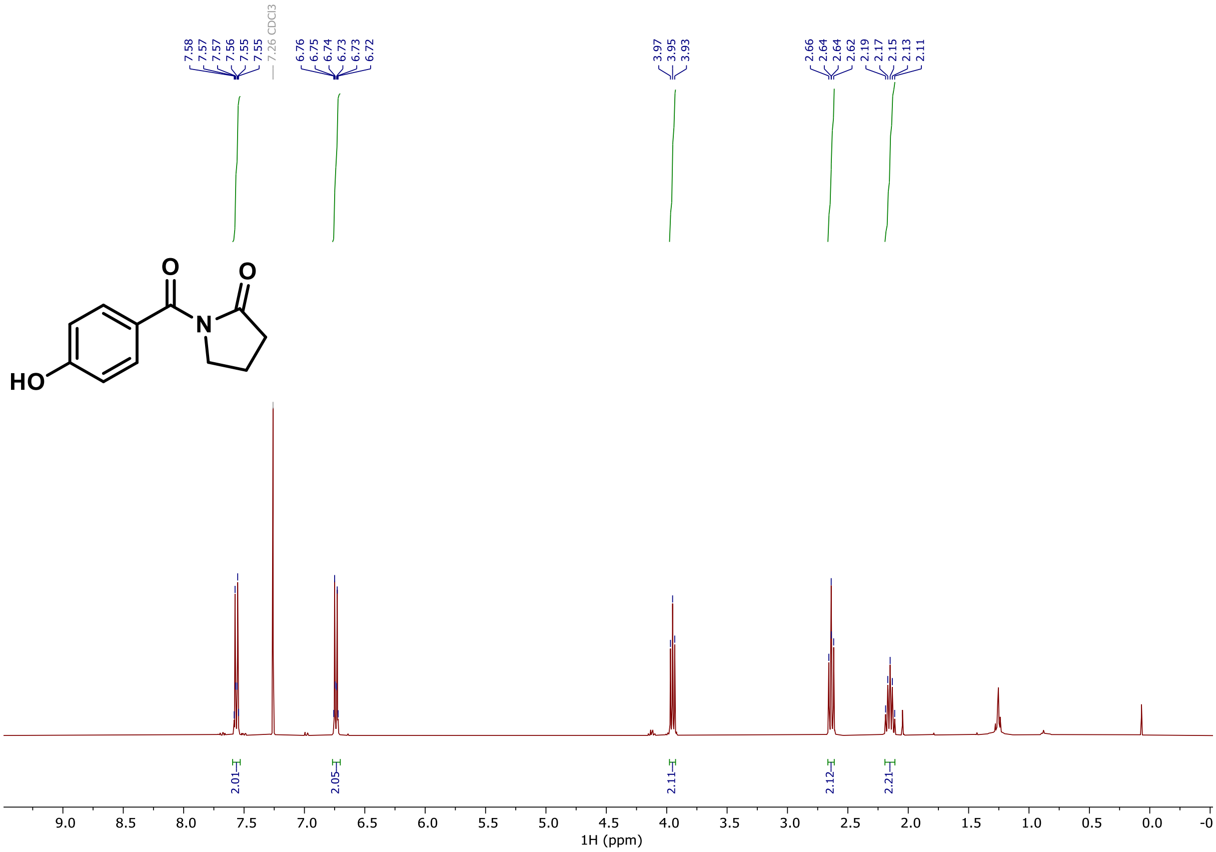

^{13}C NMR (101 MHz, CDCl$_3$) of compound **50.**

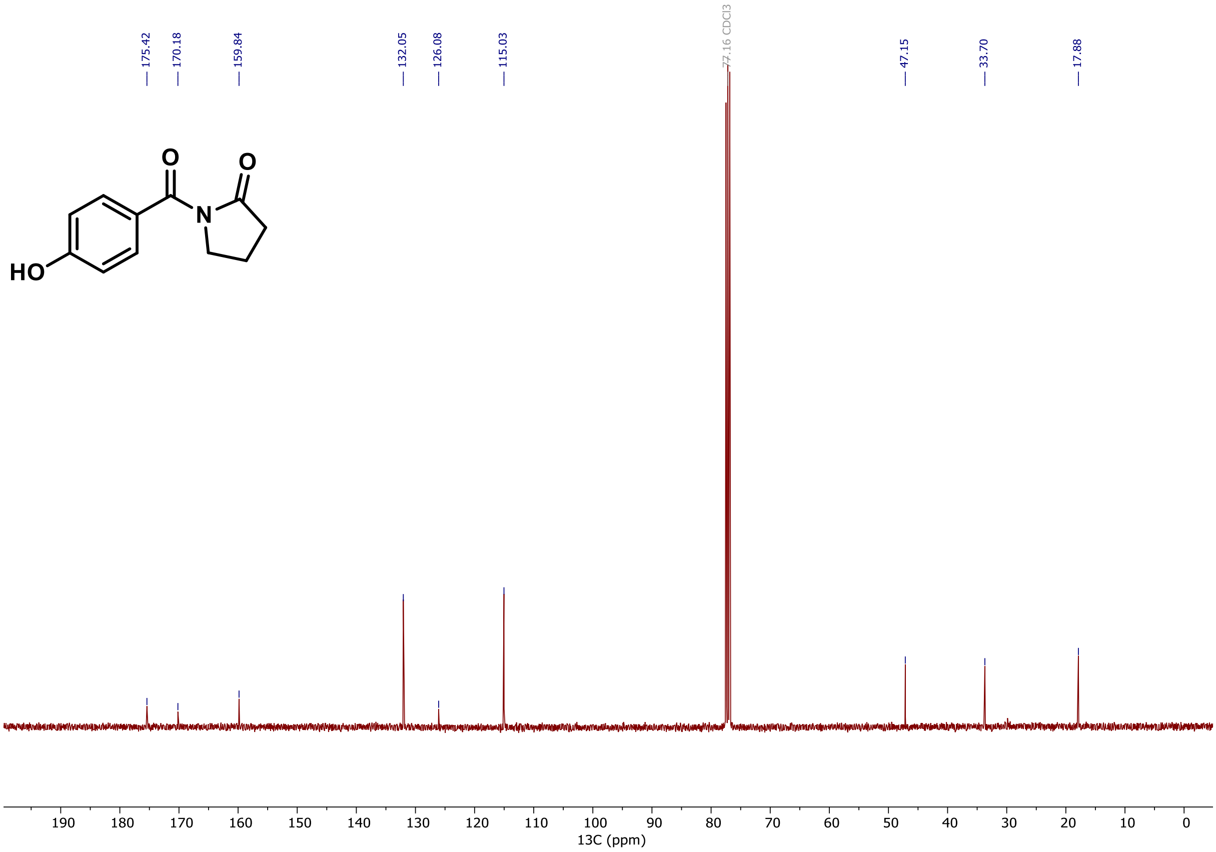

^{1}H NMR (400 MHz, CDCl₃) of compound **51.**

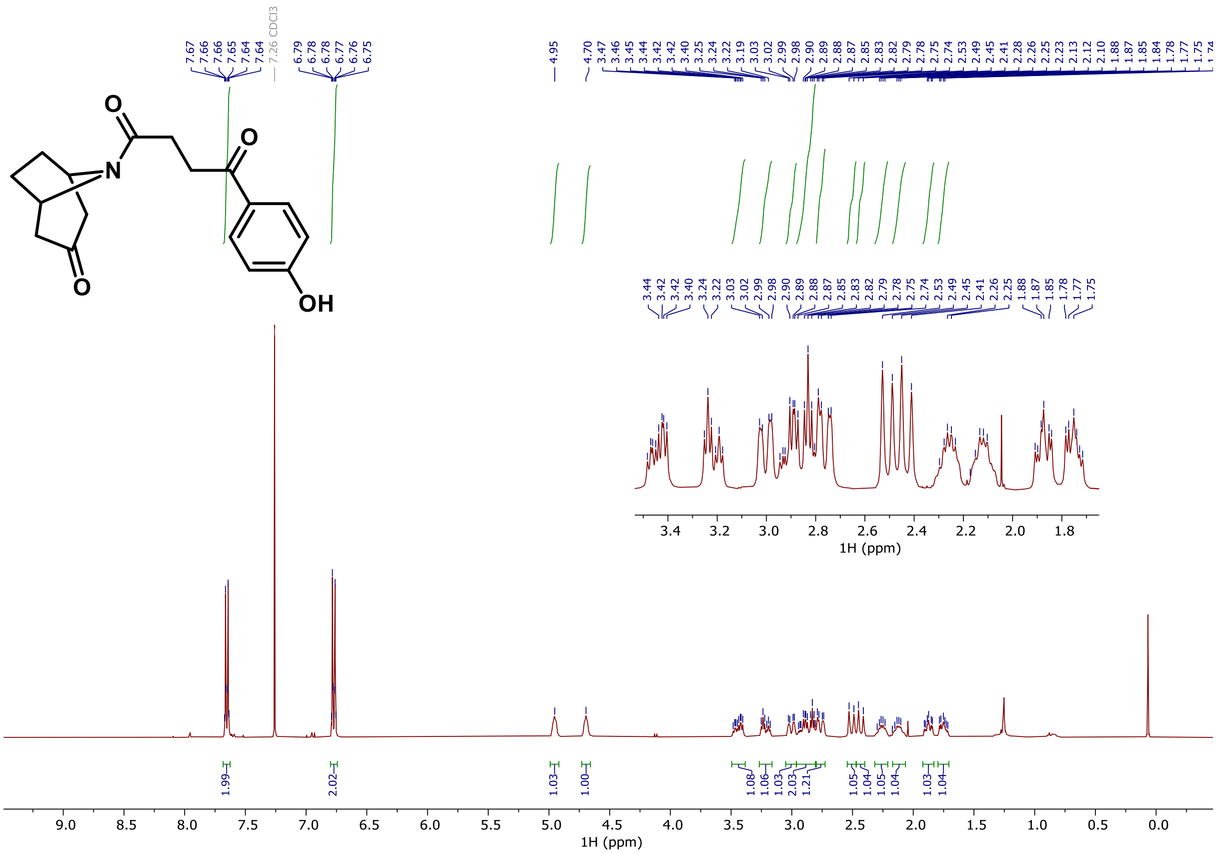

^{13}C NMR (101 MHz, CDCl₃) of compound **51.**

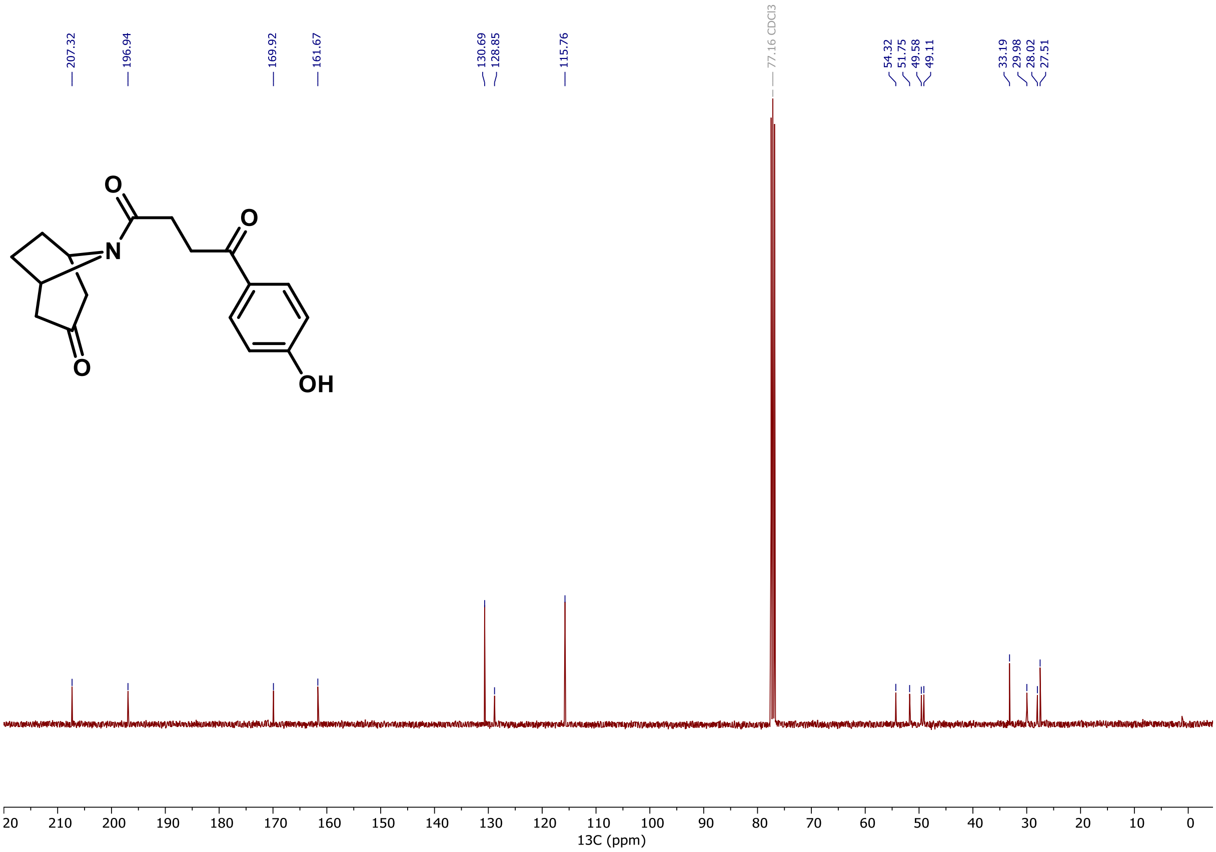

^{1}H NMR (400 MHz, $CDCl_3$) of compound 52.

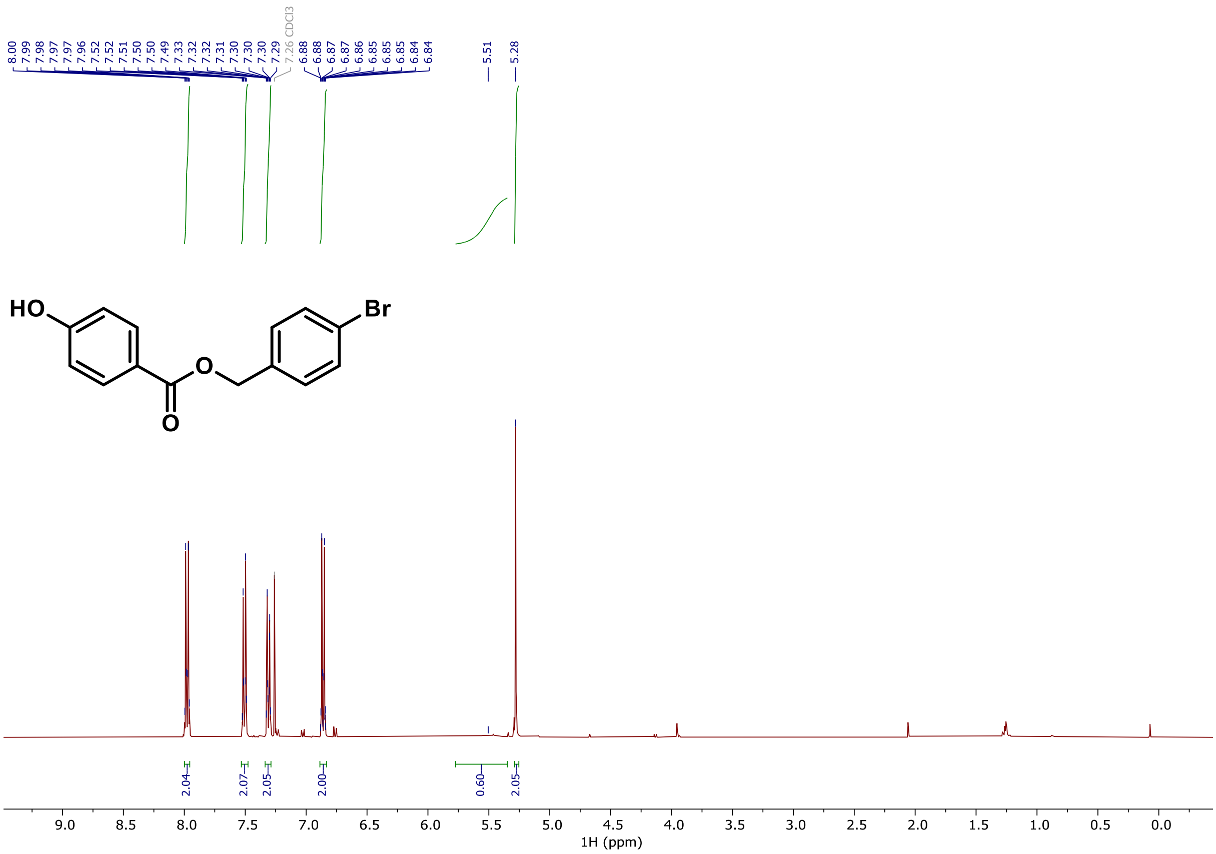

^{13}C NMR (101 MHz, $CDCl_3$) of compound 52.

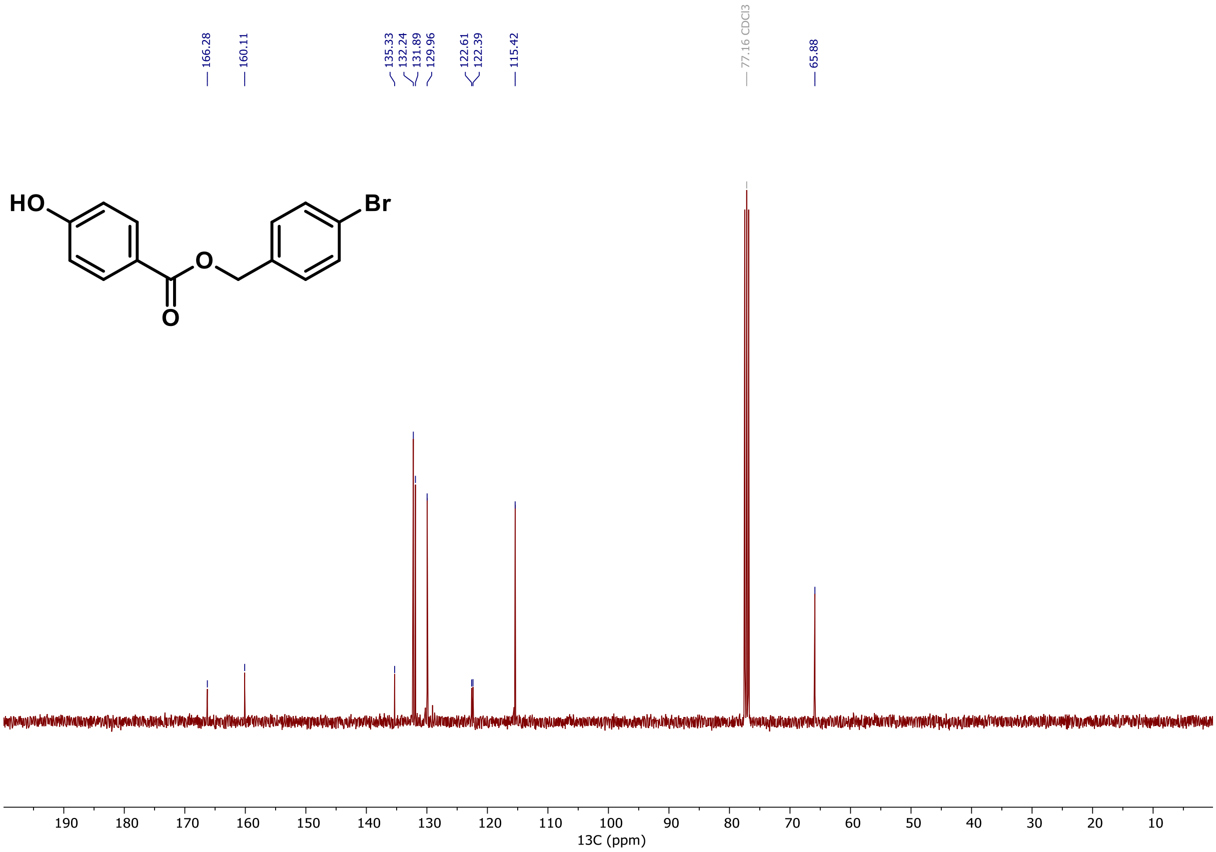

^{1}H NMR (400 MHz, CDCl$_3$) of compound **53.**

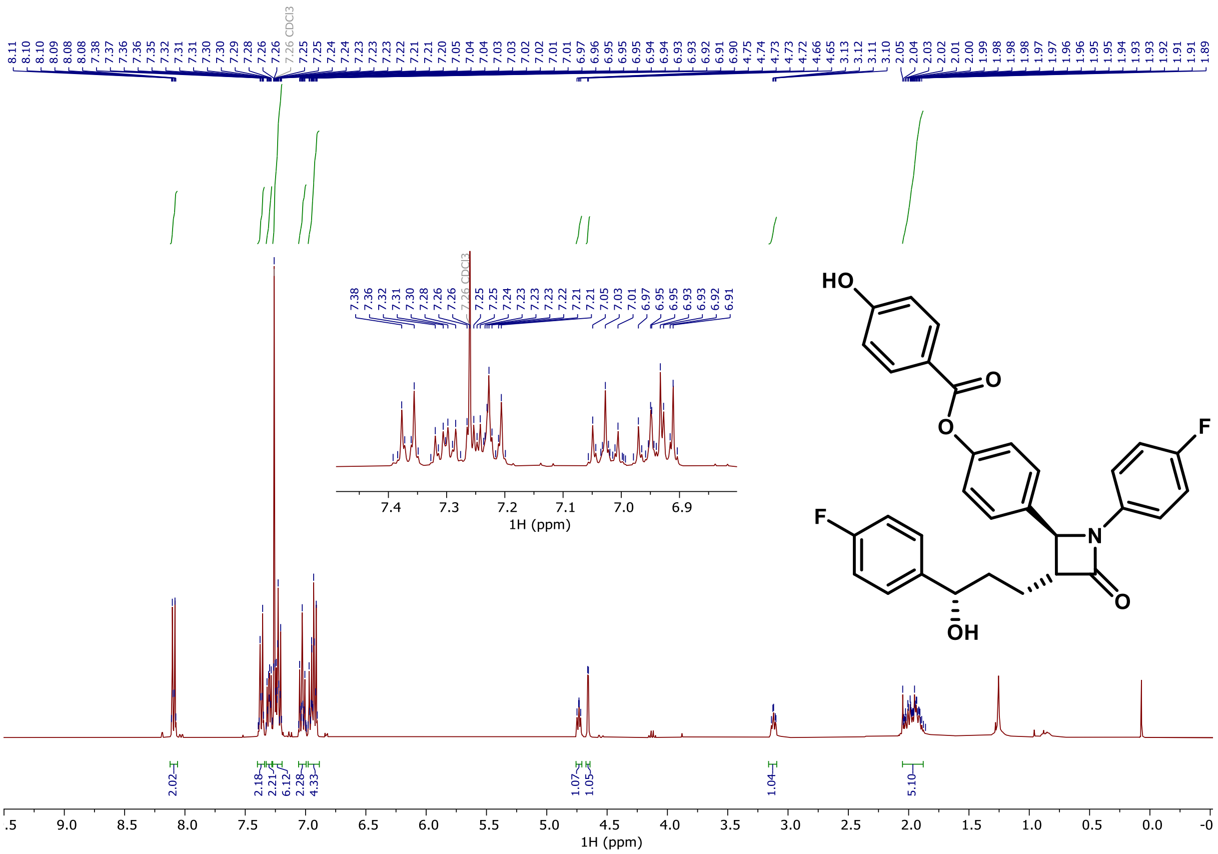

^{13}C NMR (101 MHz, CDCl$_3$) of compound **53.**

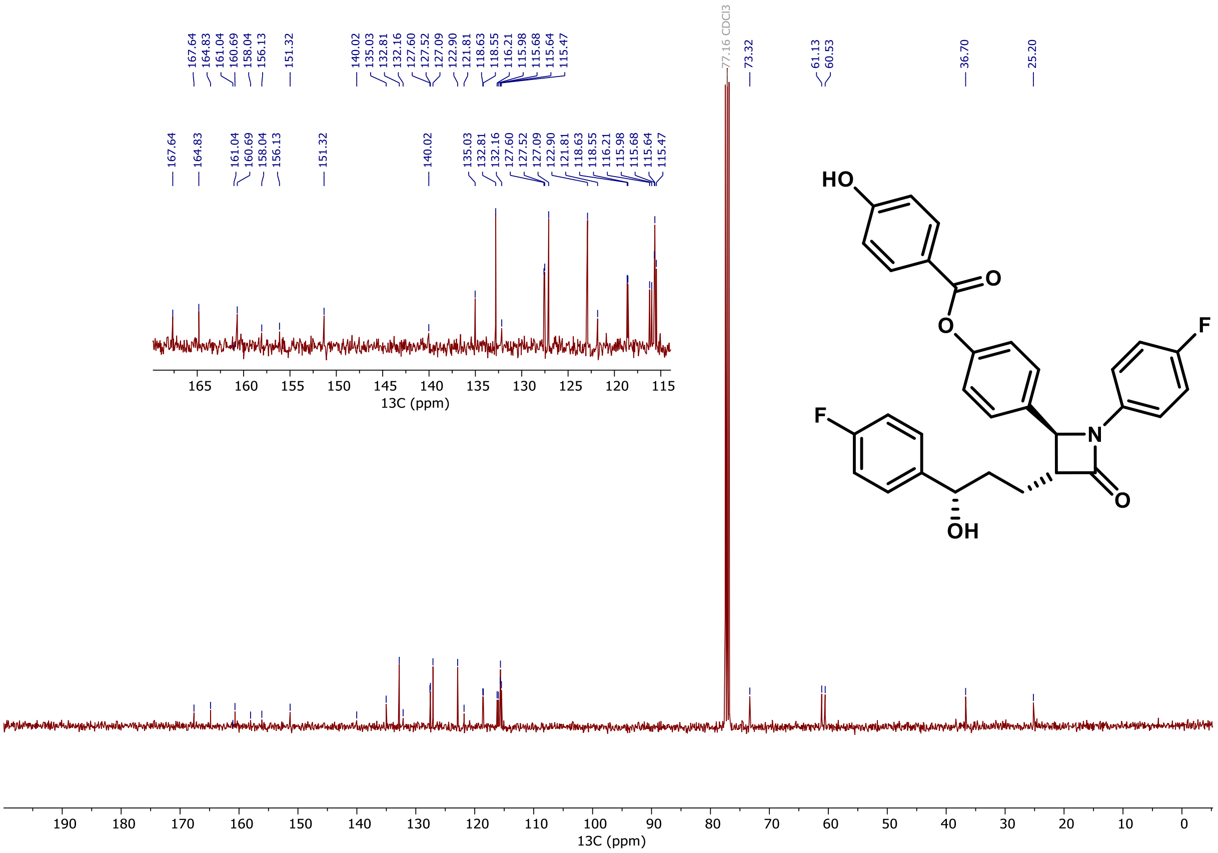

^{19}F NMR (282 MHz, $CDCl_3$) of compound **53.**

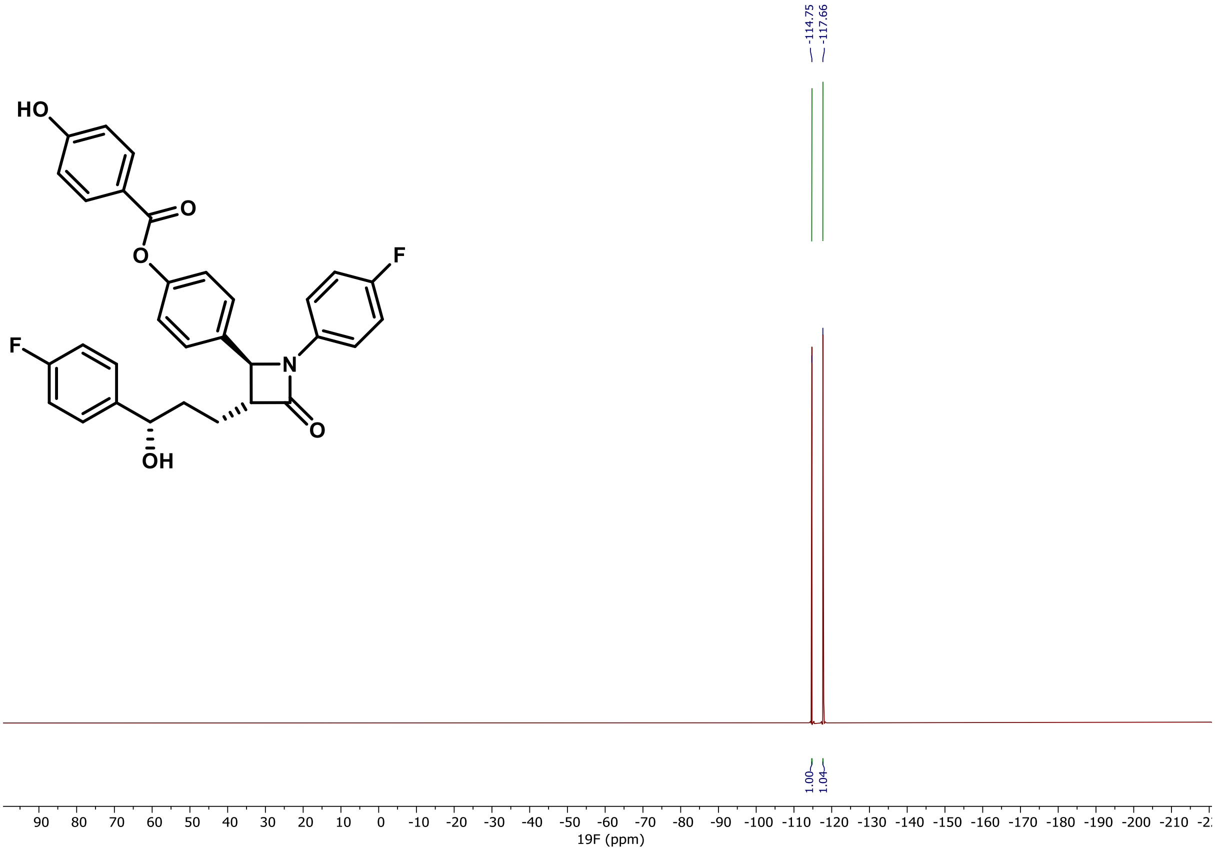

^{1}H NMR (500 MHz, $CDCl_3$, at 233 K) of compound **54.**

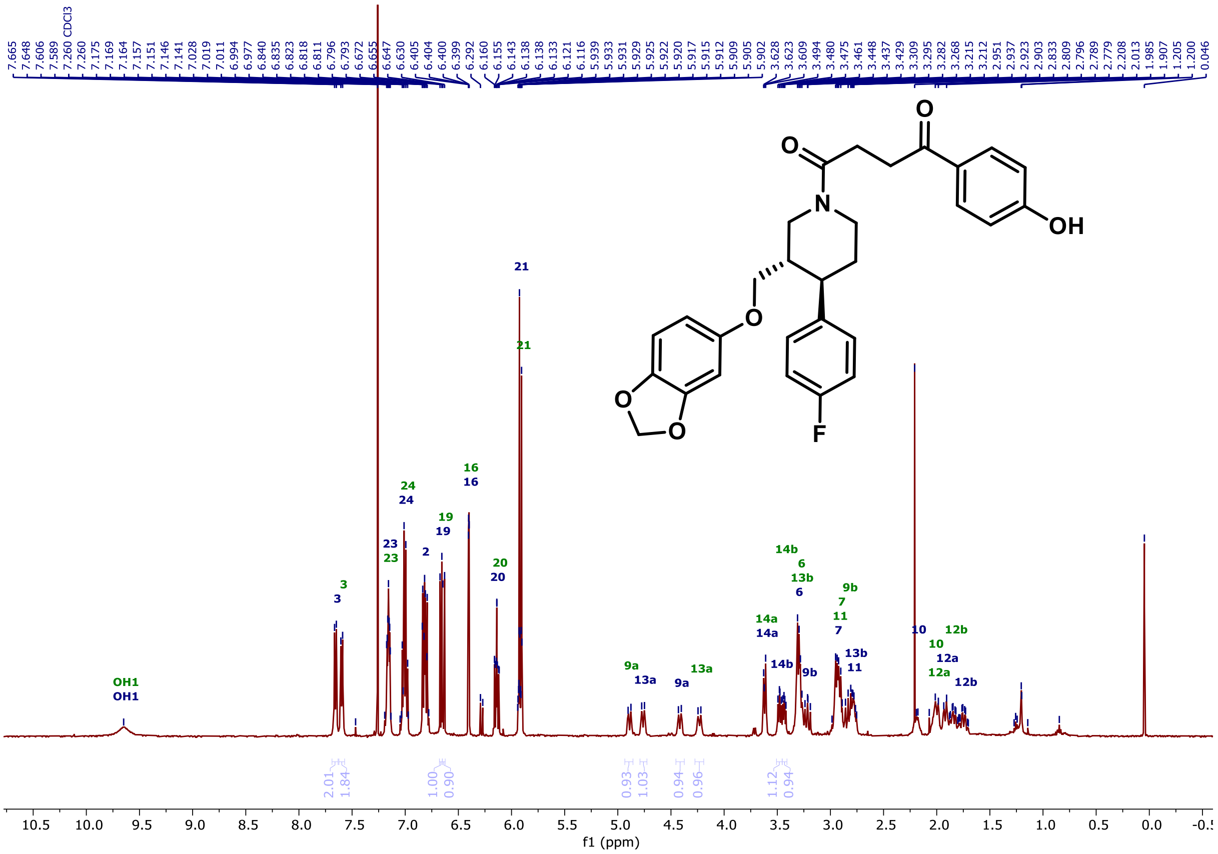

^{13}C NMR (126 MHz, $CDCl_3$) of compound **54.**

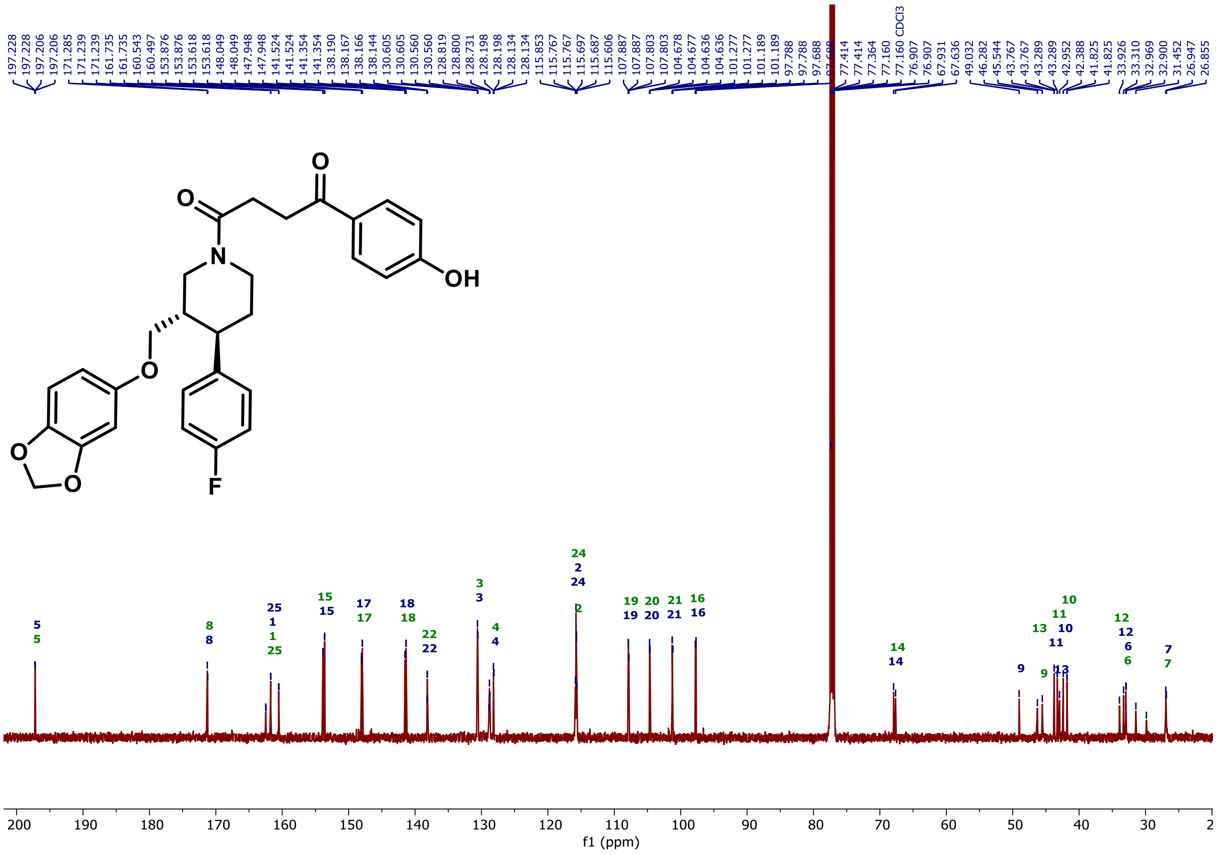

^{19}F NMR (470 MHz, CDCl$_3$, at 233 K) of compound **54.**

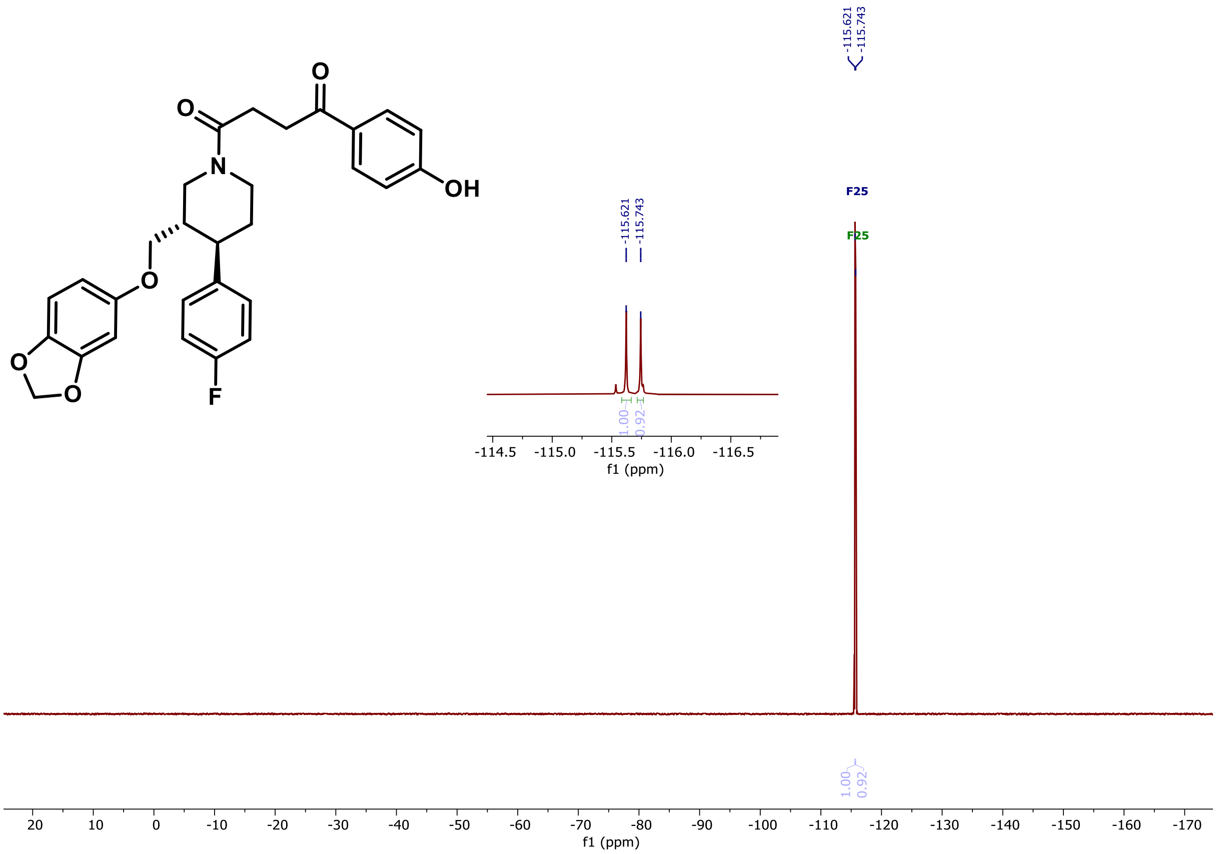

^{1}H ROESY NMR (500 MHz, CDCl$_3$, at 233 K) of compound **54.**

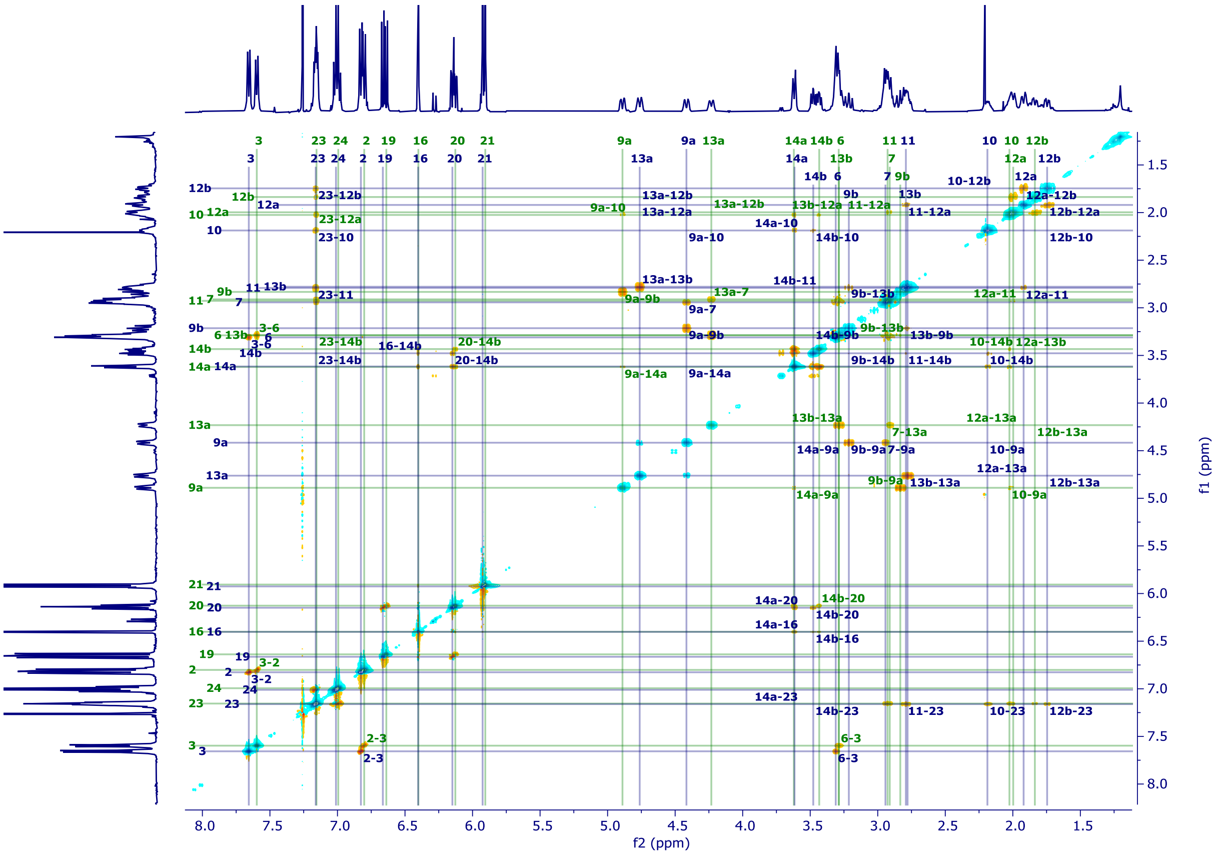

^{1}H NMR (400 MHz, $CDCl_3$) of compound **56.**

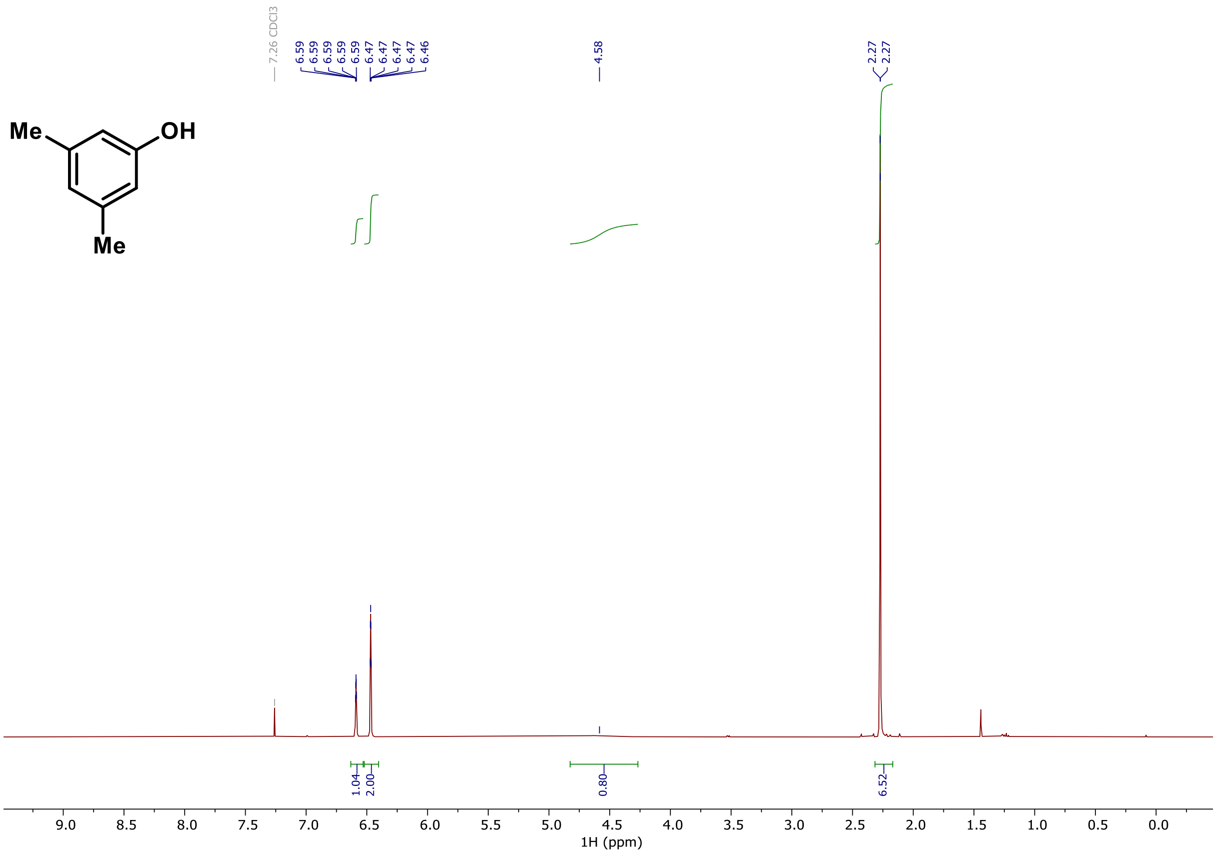

^{13}C NMR (101 MHz, $CDCl_3$) of compound **56.**

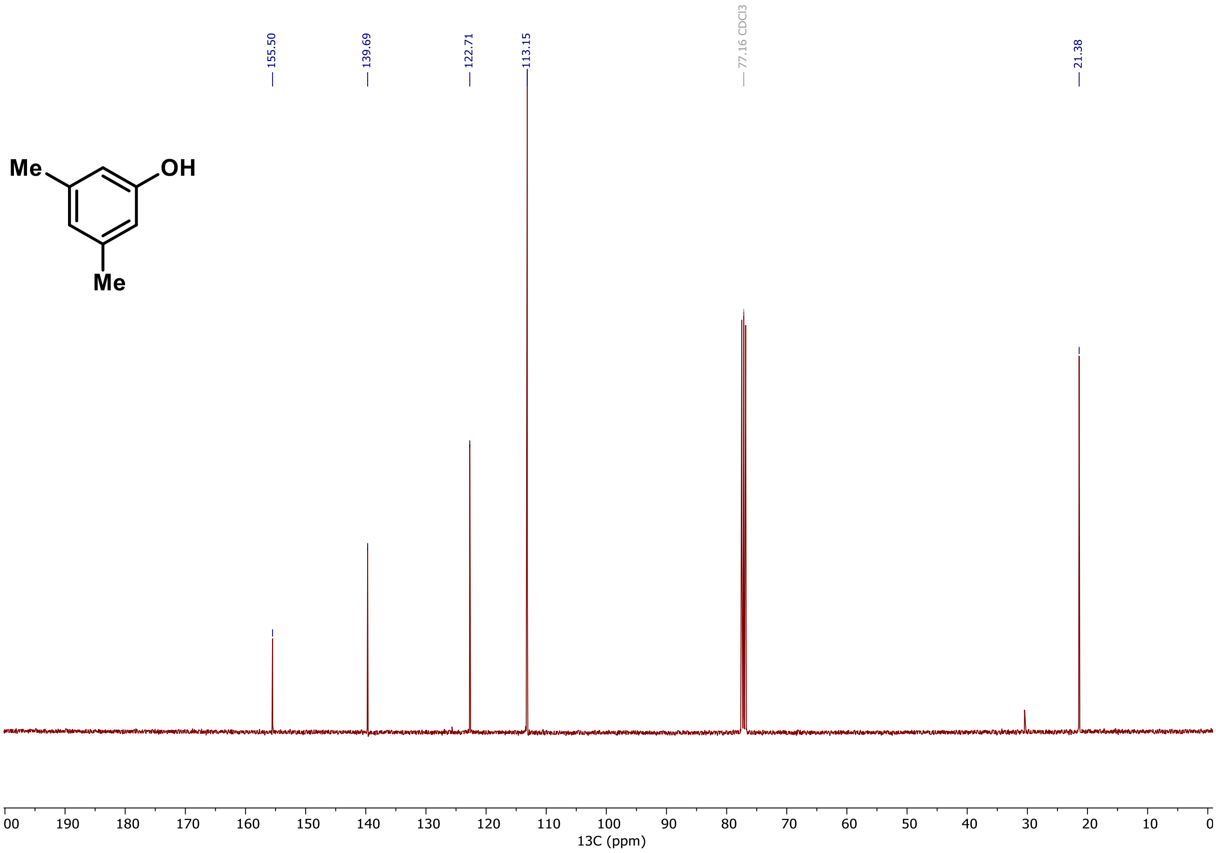

^{1}H NMR (400 MHz, $CDCl_3$) of compound **63.**

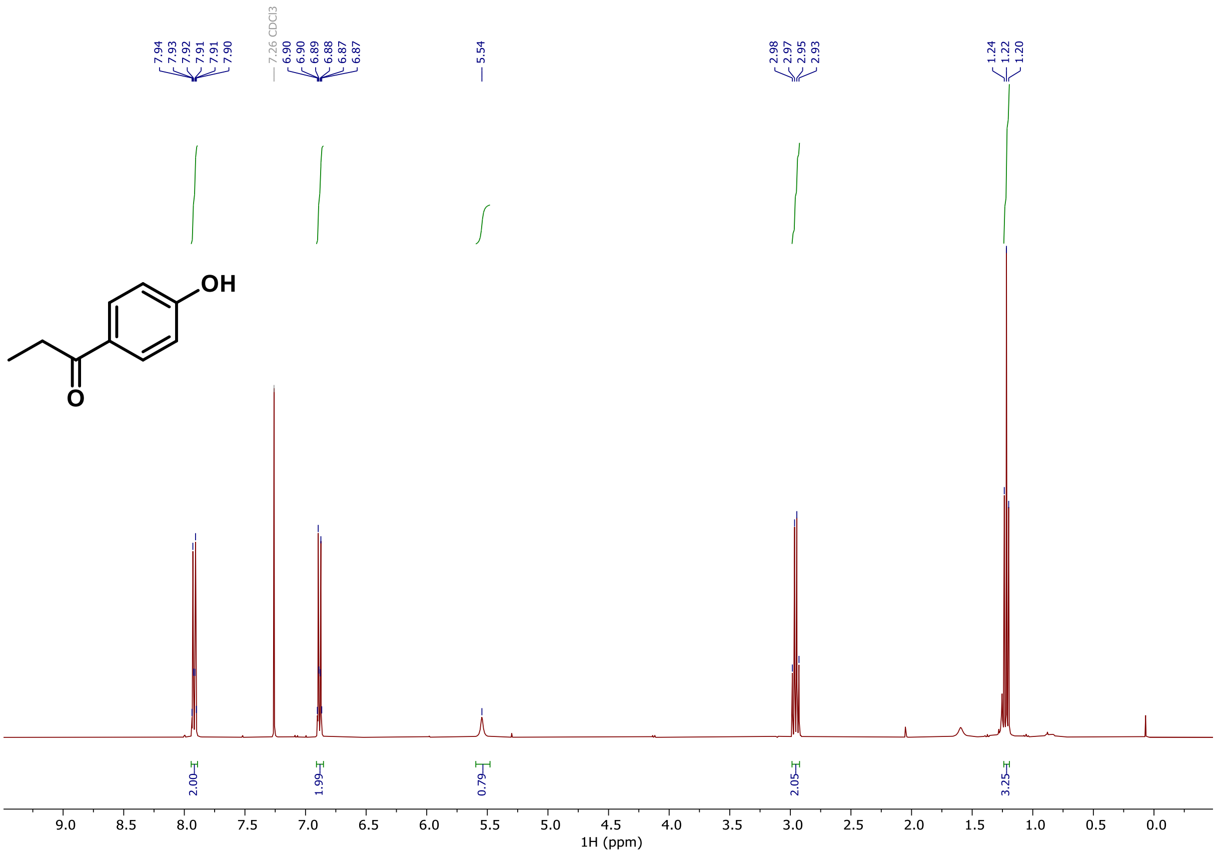

^{13}C NMR (101 MHz, $CDCl_3$) of compound **63.**

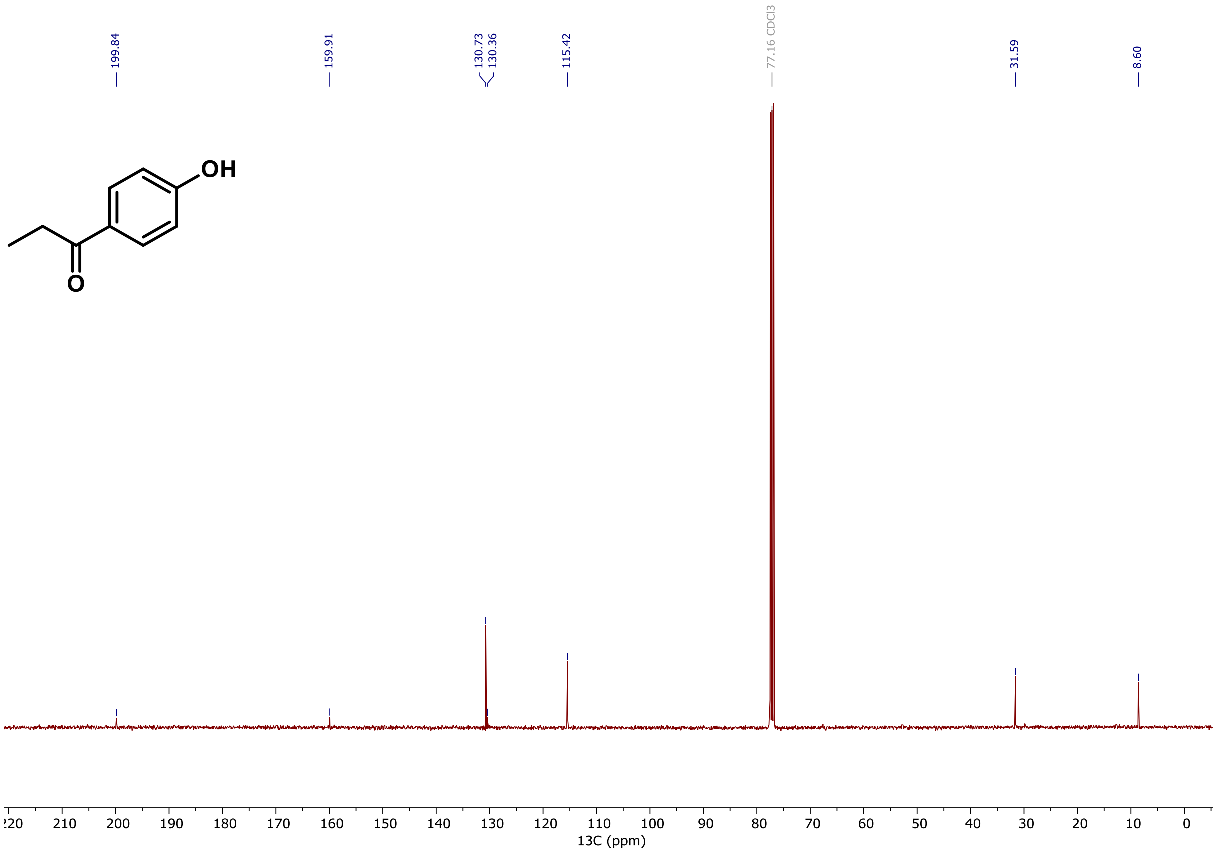

^{1}H NMR (400 MHz, DMSO-d_6) of compound **64.**

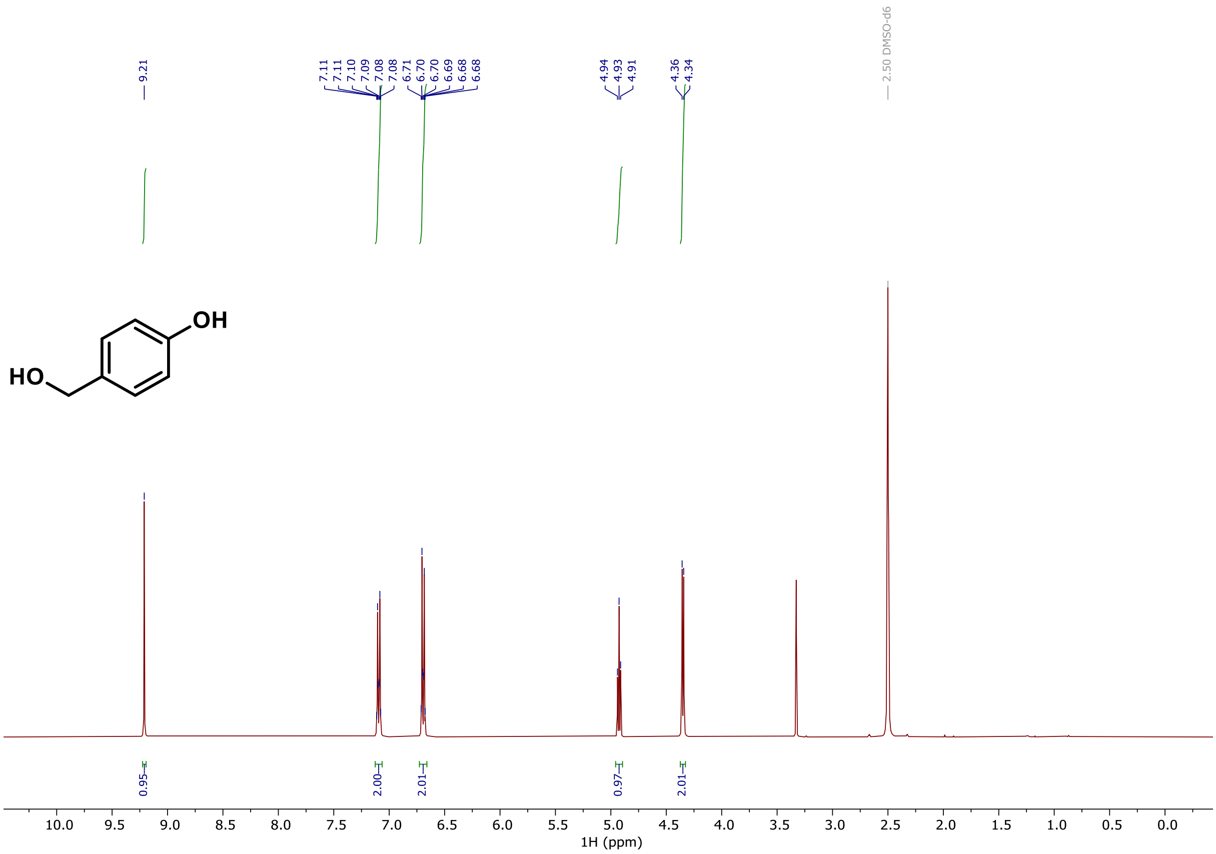

^{13}C NMR (101 MHz, DMSO-d_6) of compound **64.**

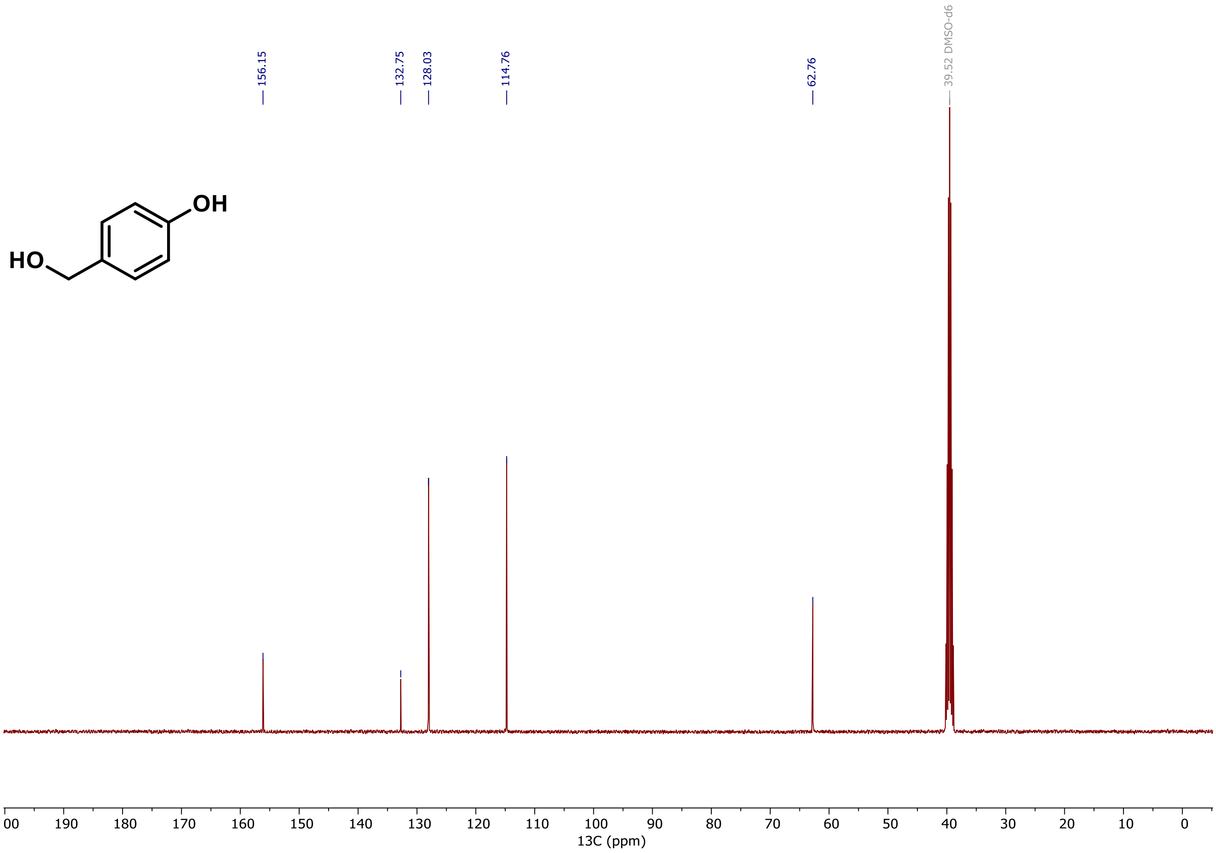

12. References

1. UNFCCC (2008) Kyoto Protocol Reference Manual on Accounting of Emissions and Assigned Amount. https://unfccc.int/resource/docs/publications/08_unfccc_kp_ref_manual.pdf.
2. Fawzy, S., Osman, A. I., Doran, J. and Rooney, D. W. Strategies for mitigation of climate change: a review. *Environ. Chem. Lett.* **18**, 2069–2094 (2020).
3. Crutzen, P. J. The influence of nitrogen oxides on the atmospheric ozone content. *Q. J. R. Meteorol. Soc.* **96**, 320 (1970).
4. Ravishankara, A. R., Daniel, J. S. and Portmann, R. W. Nitrous Oxide (N_2O): The Dominant Ozone-Depleting Substance Emitted in the 21st Century. *Science* **326**, 123–125 (2009).
5. Davidson, E. A. Representative concentration pathways and mitigation scenarios for nitrous oxide. *Environ. Res. Lett.* **7**, 024005 (2012).
6. Tian, H. *et al*. A comprehensive quantification of global nitrous oxide sources and sinks. *Nature* **586**, 248–256 (2020).
7. Parmon, V. N., Panov G. I., Uriarte, A. and Noskov, A. S. Nitrous oxide in oxidation chemistry and catalysis: application and production. *Catal. Today* **100**, 115–131 (2005).
8. Teles, J. H., Roessler, B., Pinkos, R., Genger, T. and Preiss, T. Method for producing a ketone. US Patent US7449606, Nov 11 (2008).
9. Tolman, W. B. Binding and Activation of N_2O at Transition-Metal Centers: Recent Mechanistic Insights. *Angew. Chem. Int. Ed.* **49**, 1018–1024 (2010).
10. Severin, K. Synthetic chemistry with nitrous oxide. *Chem. Soc. Rev.* **44**, 6375–6386 (2015).
11. Groves, J. T. and Roman, J. S. Nitrous Oxide Activation by a Ruthenium Porphyrin. *J. Am. Chem. Soc.* **117**, 5594-5595 (1995).
12. Aresta, M., Dibenedetto, A. and Angelini, A. Catalysis for the Valorization of Exhaust Carbon: from CO_2 to Chemicals, Materials, and Fuels. Technological Use of CO_2. *Chem. Rev.* **114**, 1709–1742 (2014).
13. Sun, L., Wang, Y., Guan, N. and Li, L. Methane Activation and Utilization: Current Status and Future Challenges. *Energy Technol.* **8**, 1900826 (2020).
14. Corona, T. and Company, A. Nitrous oxide activation by a cobalt(ii) complex for aldehyde oxidation under mild conditions. *Dalton Trans*. **37**, 14530-14533 (2016).
15. Gianetti, T. L. *et al*. Nitrous Oxide as a Hydrogen Acceptor for the Dehydrogenative Coupling of Alcohols. *Angew. Chem. Int. Ed.* **55**, 1854–1858 (2015).
16. Yonke, B. L., Reeds, J. P., Zavalij, P. Y. and Sita, L. R. Catalytic Degenerate and Nondegenerate Oxygen Atom Transfers Employing N_2O and CO_2 and a MII/MIV Cycle Mediated by Group 6 MIV Terminal Oxo Complexes. *Angew. Chem. Int. Ed.* **50**, 12342–12346 (2011).
17. Vaughan, G. A., Rupert, P. B. and Hillhouse, G. L. Selective O-atom transfer from nitrous oxide to hydride and aryl ligands of bis(pentamethylcyclopentadienyl)hafnium derivatives. *J. Am. Chem. Soc.* **109**, 5538–5539 (1987).
18. Figg, T. M., Cundari, T. R. and Gunnoe, T. B. Non-redox Oxy-Insertion via Organometallic Baeyer–Villiger Transformations: A Computational Hammett Study of Platinum(II) Complexes. *Organometallics* **30**, 3779–3785 (2011).
19. Figg, T. M., Webb, J. R., Cundari, T. R. and Gunnoe, T. B. Carbon–Oxygen Bond Formation via Organometallic Baeyer–Villiger Transformations: A Computational Study on the Impact of Metal Identity. *J. Am. Chem. Soc.* **134**, 2332–2339 (2012).

20. Cheng, M.-J. *et al.* The para-substituent effect and pH-dependence of the organometallic Baeyer–Villiger oxidation of rhenium–carbon bonds. *Dalton Trans.* **41**, 3758–3763 (2012).
21. Mei, J. *et al.* Oxygen Atom Insertion into Iron(II) Phenyl and Methyl Bonds: A Key Step for Catalytic Hydrocarbon Functionalization. *Organometallics* **33**, 5597–5605 (2014).
22. Bischof, S. M. *et al.* Functionalization of Rhenium Aryl Bonds by O-Atom Transfer. *Organometallics* **30**, 2079–2082 (2011).
23. Sugimoto, H. *et al.* Oxygen Atom Insertion into the Osmium–Carbon Bond via an Organometallic Oxido–Osmium(V) Intermediate. *Organometallics* **40**, 102–106 (2021).
24. Liu, Y., Liu, S. and Xiao, Y. Transition-metal-catalyzed synthesis of phenols and aryl thiols. *Beilstein J. Org. Chem.* **13**, 589–611 (2017).
25. Anderson, K. W., Ikawa, T., Tundel, R. E. and Buchwald, S. L. The Selective Reaction of Aryl Halides with KOH: Synthesis of Phenols, Aromatic Ethers, and Benzofurans. *J. Am. Chem. Soc.* **128**, 10694–10695 (2006).
26. Sambiagio, C., Marsden, S. P., Blacker, A. J. and McGowan, P. C. Copper catalysed Ullmann type chemistry: from mechanistic aspects to modern development. *Chem. Soc. Rev.* **43**, 3525–3550 (2014).
27. Tlili, A., Xia., N., Monnier, F. and Taillefer, M. A Very Simple Copper-Catalyzed Synthesis of Phenols Employing Hydroxide Salts. *Angew. Chem. Int. Ed.* **48**, 8725–8728 (2009).
28. Zhao, D. *et al.* Synthesis of Phenol, Aromatic Ether, and Benzofuran Derivatives by Copper-Catalyzed Hydroxylation of Aryl Halides. *Angew. Chem. Int. Ed.* **48**, 8729–8732 (2009).
29. Fier, P. S. and Maloney, K. M. Synthesis of Complex Phenols Enabled by a Rationally Designed Hydroxide Surrogate. *Angew. Chem. Int. Ed*. **56**, 4478-4482 (2017).
30. Terrett, J. A., Cuthbertson, J. D., Shurtleff, V. W. and MacMillan, D. W. C. Switching on elusive organometallic mechanisms with photoredox catalysis. *Nature* **524**, 330–334 (2015).
31. Yang, L. *et al.* Synthesis of Phenols: Organophotoredox/Nickel Dual Catalytic Hydroxylation of Aryl Halides with Water. *Angew. Chem. Int. Ed.* **57**, 1968–1972 (2018).
32. Sun, R., Qin, Y. and Nocera, D. G. General Paradigm in Photoredox Nickel-Catalyzed Cross-Coupling Allows for Light-Free Access to Reactivity. *Angew. Chem. Int. Ed.* **59**, 9527–9533 (2020).
33. Li, Z. *et al.* A tautomeric ligand enables directed C–H hydroxylation with molecular oxygen. *Science* **372**, 1452–1457 (2021).
34. Koo, K., Hillhouse, G. L. and Rheingold, A. L. Oxygen-Atom Transfer from Nitrous Oxide to an Organonickel(II) Phosphine Complex. Syntheses and Reactions of New Nickel(II) Aryloxides and the Crystal Structure of [cyclic] (Me2PCH2CH2PMe2)Ni(O-o-C6H4CMe2CH2). *Organometallics* **14**, 456–460 (1995).
35. Matsunaga, P. T., Hillhouse, G. L. and Rheingold, A. L. Oxygen-atom transfer from nitrous oxide to a nickel metallacycle. Synthesis, structure, and reactions of [cyclic] (2,2'-bipyridine)Ni(OCH2CH2CH2CH2). *J. Am. Chem. Soc.* **115**, 2075–2077 (1993).
36. Lin, C.-Y. and Power, P. P. Complexes of Ni(i): a “rare” oxidation state of growing importance. *Chem. Soc. Rev.* **46**, 5347–5399 (2017).
37. Klein, A., Kaiser, A., Sarkar, B., Wanner, M. and Fiedler, J. The Electrochemical Behaviour of Organonickel Complexes: Mono-, Di- and Trivalent Nickel. *Eur. J. Inorg. Chem.* **2007**, 965–976 (2007).

38. Hamacher, C., Hurkes, N., Kaiser, A., Klein, A. and Schüren, A. Electrochemistry and Spectroscopy of Organometallic Terpyridine Nickel Complexes. *Inorg. Chem.* **48**, 9947–9951 (2009).
39. Figg, T. M. and Cundari, T. R. Mechanistic Study of Oxy Insertion into Nickel-Carbon Bonds with Nitrous Oxide. *Organometallics* **31**, 4998–5004 (2012).
40. Liang, Y. *et al.* Electrochemically induced nickel catalysis for oxygenation reactions with water. *Nat. Catal.* **4**, 116–123 (2021).
41. Walsh, S. *et al.* INHIBITORS OF THE RENAL OUTER MEDULLARY POTASSIUM CHANNEL. WO2013039802 (A1) (Merck Sharp & Dohme, 2013).
42. Del Vecchio, A. *et al.* Audisio, Carbon isotope labeling of carbamates by late-stage [^{11}C], [^{13}C] and [^{14}C]carbon dioxide incorporation. *Chem. Commun.* **56**, 11677–11680 (2020).
43. Solyev, P. N., Sherman, D. K., Novikov, R. A., Levina, E. A. and Kochetkov, S. N. Hydrazo Coupling: the Efficient Transition-Metal-Free C–H Functionalization of 8-Hydroxyquinoline and Phenol through Base Catalysis. *Green Chem.* **21**, 6381–6389 (2019).
44. Kelly, P. M. *et al.* Synthesis, antiproliferative and pro-apoptotic activityof 2-phenylindoles. *Bioorg. Med. Chem*. **24**, 4075-4099 (2016).
45. Wang, C., Sun, H., Fang, Y. and Huang, Y. General and Efficient Synthesis of Indoles through Triazene-Directed C–H Annulation. *Angew. Chem. Int. Ed.* **52**, 5795–5798 (2013).
46. Hwang, S.G. *et al.* Methods of Preparing Bazedoxifene. KR201895239 (A) (M F C Co., Ltd., 2018).

Supplementary References

47. Reijerse, E. J., Lendzian, F., Isaacson, R. and Lubitz, W. A A tunable general purpose Q-band resonator for CW and pulse EPR/ENDOR experiments with large sample access and optical excitation. *J. Magn. Reson.* **214**, 237–243 (2012).
48. Hyde, J. S., Pasenkiewicz-Gierula, M., Jesmanowicz, A. and Antholine, W. E. Pseudo field modulation in EPR spectroscopy. *Appl. Magn. Reson.* **1**, 483, (1990).
49. Bogdanović, B., Kröner, M. and Wilke, G. Olefin-Komplexe des Nickel(0). *Liebigs Ann. Chem.* **699**, 1–23 (1966).
50. Guo, L., Tu, H.-Y., Zhu, S. and Chu, L. Selective, Intermolecular Alkylarylation of Alkenes via Photoredox/Nickel Dual Catalysis. *Org. Lett.* **21**, 4771–4776 (2019).
51. Beckmann, U., Hägele, G. and Frank, W. Square-Planar 2-Toluenido(triphenylphosphane)nickel(II) Complexes Containing Bidentate N,O Ligands: An Example of Planar Chirality. *Eur. J. Inorg. Chem.* **2010**, 1670–1678 (2010).
52. Hamacher, C., Hurkes, N., Kaiser, A. and Klein, A. Back-bonding in Organonickel Complexes with Terpyridine Ligands – A Structural Approach. *Z. Anorg. Allg. Chem.* **633**, 2711–2718 (2007).
53. Ciszewski, J. T. *et al.* Redox Trends in Terpyridine Nickel Complexes. *Inorg. Chem.* **50**, 8630–8635 (2011).
54. Anderson, T. J., Jones, G. D. and Vicic, D. A. Evidence for a NiI Active Species in the Catalytic Cross-Coupling of Alkyl Electrophiles. *J. Am. Chem. Soc.* **126**, 8100–8101 (2004).
55. Delcamp, J. H., Rodrigues, R. R., Peddapuram, A. and Cheema, H. A. DYES, DYE-SENSITIZED SOLAR CELLS, AND METHODS OF MAKING AND USING THE SAME. WO2019/23436 (A1) (U. Mississippi, 2019).

56. Teo, Y.-C., Yong, F.-F. and Sim, S. Ligand-free Cu2O-catalyzed cross coupling of nitrogen heterocycles with iodopyridines. *Tetrahedron* **69**, 7279–7284 (2013).
57. Li, H., Zhong, Y.-L., Chen, C.-Y., Ferraro, A. E. and Wang, D. A Concise and Atom-Economical Suzuki–Miyaura Coupling Reaction Using Unactivated Trialkyl- and Triarylboranes with Aryl Halides. *Org. Lett.* **17**, 3616−3619 (2015).
58. Erkkilä, A. and Pihko, P. M. Mild Organocatalytic α-Methylenation of Aldehydes. *J. Org. Chem.* **71**, 2538−2541 (2006).
59. Sasaki, I., Daran, J. C. and Balavoine, G. G. A. An Effective Route to Polysubstituted Symmetric Terpyridines. *Synthesis* **1999**, 815−820 (1999).
60. Peng, J., Docherty, J. H., Dominey, A. P. and Thomas, S. P. Cobalt-catalysed Markovnikov selective hydroboration of vinylarenes. *Chem. Commun.* **53**, 4726−4729 (2017).
61. Arendt, K. M. and Doyle, A. G. Dialkyl Ether Formation by Nickel-Catalyzed Cross-Coupling of Acetals and Aryl Iodides. *Angew. Chem. Int. Ed.* **54**, 9876−9880 (2015).
62. Skibinski, D., Jain, S., Singh, M. and O'Hagan, D. Homogenous Suspension of Immunopotentiating Compounds and Uses Thereof. US9408907, (B2) (GlaxoSmithKline Biologicals SA, 2016).
63. Rosen, B. M., Huang, C. and Percec, V. Sequential Ni-Catalyzed Borylation and Cross-Coupling of Aryl Halides via in Situ Prepared Neopentylglycolborane. *Org. Lett.* **10**, 2597–2600 (2008).
64. Nguyen, N.-H., Apriletti, J. W., Baxter, J. D. and Scanlan, T. S. Hammett Analysis of Selective Thyroid Hormone Receptor Modulators Reveals Structural and Electronic Requirements for Hormone Antagonists. *J. Am. Chem. Soc.* **127**, 4599–4608 (2005).
65. Hong, M. C. *et. al.* Synthesis and evaluation of stilbene derivatives as a potential imaging agent of amyloid plaques. *Bioorg. Med. Chem.* **18**, 7724–7730 (2010).
66. Shao. X. and Malcolmson, S. J. Catalytic Enantio- and Diastereoselective Cyclopropanation of 2-Azadienes for the Synthesis of Aminocyclopropanes Bearing Quaternary Carbon Stereogenic Centers. *Org. Lett.* **21**, 7380–7385 (2019).
67. Bratt, E. and Granberg, K. Heterocyclic sulfonamide derivatives as inhibitors of factor xa. WO2007008144 (A1) (Astrazeneca, 2007).
68. Tan, H. *et al*. N-Heterocyclic Carbene Catalyzed Ester Synthesis from Organic Halides through Incorporation of Oxygen Atoms from Air. *Angew. Chem. Int. Ed.* **60**, 2140–2144 (2021).
69. Liu, Y., Solari, E., Scopelliti, R., Fadai Tirani, F. and Severin, K. Lewis Acid-Mediated One-Electron Reduction of Nitrous Oxide. *Chem. Eur. J.* **24**, 18809–18815 (2018).
70. Hsu, C.-W., Rathnayaka, S. C., Islam, S. M., MacMillan, S. N. and Mankad, N. P. N_2O Reductase Activity of a [Cu_4S] Cluster in the 4CuI Redox State Modulated by Hydrogen Bond Donors and Proton Relays in the Secondary Coordination Sphere. *Angew. Chem. Int. Ed.* **59**, 627–631 (2020).
71. Charbonneau, D. J. *et al.* Development of an Improved System for the Carboxylation of Aryl Halides through Mechanistic Studies. *ACS Catal.* **9**, 3228−3241 (2019).
72. Kjellberg, M. *et al.* Photocatalytic deoxygenation of N–O bonds with rhenium complexes: from the reduction of nitrous oxide to pyridine N-oxides. *Chem. Sci. ASAP*. DOI: *10.1039/d1sc01974k* (2021).
73. Smith III, M. R., Matsunaga, P. T. and Andersen, R. A. Preparation of monomeric (Me5C5)2VO and (Me5C5)2Ti(O)(L) and their decomposition to (Me5C5)4M4(.mu.-O)6. *J. Am. Chem. Soc.* **115**, 7049−7050 (1993).
74. Huang, L., Ackerman, L. K. G., Kang, K., Parsons, A. M. and Weix, D. J. LiCl-Accelerated Multimetallic Cross-Coupling of Aryl Chlorides with Aryl Triflates. *J. Am. Chem. Soc.* **141**, 10978–10983 (2019).

75. Stoll, S. and Schweiger, A. EasySpin, a comprehensive software package for spectral simulation and analysis in EPR. *J. Magn. Reson.* **178**, 42–55 (2006).
76. Deeba, R., Molton, F., Chardon-Noblat, S. and Costentin, C. Effective Homogeneous Catalysis of Electrochemical Reduction of Nitrous Oxide to Dinitrogen at Rhenium Carbonyl Catalysts. *ACS Catal.* **11**, 6099–6103 (2021).
77. Gelling, A., Orrell, K. G., Osborne, A. G. and Šik, V. Rhenium(I) tricarbonyl halide complexes of pyrazolyl-bipyridine ligands. Part 1. Nuclear magnetic resonance studies of solution fluxionality. *J. Chem. Soc., Dalton Trans.* 3545–3552 (1994).
78. Gelling, A. *et al.* Trimethylplatinum(iv) halide complexes of pyrazolylbipyridyl ligands. crystal structure of [PtIMe3{6-(3,5-DIMETHYLPYRAZOL-1-YL)-2,2′-BIPYRIDINE}]. *Polyhedron* **15**, 3203–3210 (1996).
79. Kawamata, Y. *et al.* Electrochemically Driven, Ni-Catalyzed Aryl Amination: Scope, Mechanism, and Applications. *J. Am. Chem. Soc.* **141**, 6392–6402 (2019).
80. Weng, W.-Z., Liang, H. and Zhang, B. Visible-Light-Mediated Aerobic Oxidation of Organoboron Compounds Using in Situ Generated Hydrogen Peroxide. *Org. Lett.* **20**, 4979–4983 (2018).
81. Jiang, M., Yang, H. and Fu, H. Visible-Light Photoredox Borylation of Aryl Halides and Subsequent Aerobic Oxidative Hydroxylation. *Org. Lett.* **18**, 5248–5251 (2016).
82. Zhang, Y. *et al.* Pyrenediones as versatile photocatalysts for oxygenation reactions with *in situ* generation of hydrogen peroxide under visible light. *Green Chem.,* **22**, 22–27 (2020).
83. Song, Z.-Q. and Wang, D.-H. Palladium-Catalyzed Hydroxylation of Aryl Halides with Boric Acid. *Org. Lett.* **22**, 8470–8474 (2020).
84. Tung, C. L., Wong, C. T. T., Fung, E. Y. M. and Li, X. Traceless and Chemoselective Amine Bioconjugation via Phthalimidine Formation in Native Protein Modification. *Org. Lett.* **18**, 2600–2603 (2016).
85. Kumar, I., Sharma, R., Kumar, R., Kumar, R. and Sharma, U. C70 Fullerene-Catalyzed Metal-Free Photocatalytic ipso-Hydroxylation of Aryl Boronic Acids: Synthesis of Phenols. *Adv. Synth. Catal.* **360**, 2013–2019 (2018).
86. Zhu, C., Wang, R. and Falck, J. R. Mild and Rapid Hydroxylation of Aryl/Heteroaryl Boronic Acids and Boronate Esters with N-Oxides. *Org. Lett.* **14**, 3494–3497 (2012).
87. Zhang, Y. *et al.* Bridged Ring Compounds as Hepatitis C Virus (HCV) Inhibitors and Pharmaceutical Applications Thereof. WO/2014/19344 (A1) (Sunshine Lake Pharma Co., Ltd., 2014)
88. Mottweiler, J., Rinesch, T., Besson, C., Buendia. J. and Bolm, C. Iron-catalysed oxidative cleavage of lignin and β-O-4 lignin model compounds with peroxides in DMSO. *Green Chem.* **17**, 5001–5008 (2015).
89. Yan, H., Oha, J.-S. and Song, C. E. A mild and efficient method for the selective deprotection of silyl ethers using KF in the presence of tetraethylene glycol. *Org. Biomol. Chem.* **9**, 8119–8121 (2011).
90. Ma, X. *et al.* Alcohol-based Michaelis–Arbuzov reaction: an efficient and environmentally-benign method for C–P(O) bond formation. *Green Chem.* **20**, 3408–3413 (2018).
91. Heller, S. T., Schultz, E. E. and Sarpong, R. Chemoselective N-Acylation of Indoles and Oxazolidinones with Carbonylazoles. *Angew. Chem. Int. Ed.* **51**, 8304–8308 (2012).
92. Yu, C.-W., Chen, G. S., Huang, C.-W. and Chern, J.-W. Efficient Microwave-Assisted Pd-Catalyzed Hydroxylation of Aryl Chlorides in the Presence of Carbonate. *Org. Lett.* **14**, 3688–3691 (2012).

93. Yu, H., Liu, C., Dai, X., Wang, J., Qiu J. Cyclometalated Ir(III) complexes-catalyzed aerobic hydroxylation of arylboronic acids induced by visible-light. *Tetrahedron* **73**, 3031–3035 (2017).
94. Cheung, C. W. and Buchwald, S. L. Palladium-Catalyzed Hydroxylation of Aryl and Heteroaryl Halides Enabled by the Use of a Palladacycle Precatalyst. *J. Org. Chem.* **53**, 5351–5358 (2014).
95. Giampietro, L. *et al.* Novel Phenyldiazenyl Fibrate Analogues as PPAR α/γ/δ Pan-Agonists for the Amelioration of Metabolic Syndrome. *ACS Med. Chem. Lett.* **10**, 545–551 (2019).
96. Mihai, M. T., Williams, B. D. and Phipps, R. J. Para-Selective C–H Borylation of Common Arene Building Blocks Enabled by Ion-Pairing with a Bulky Countercation. *J. Am. Chem. Soc.* **141**, 15477–15482 (2019).
97. Padwa, A., Bullock, W. H., Norman, B. H. and Perumattam, J. (Nitroaryl)sulfinyl-substituted allenes. Novel and convenient propargyl alcohol synthons in 4 + 2 cycloaddition chemistry. *J. Org. Chem.* **56**, 4252–4259 (1991).
98. Kuo, H.-M., I, C,-H., Sheu, H.-S. and Lai, C. K. Symmetric mesogenic twins derived from salicylaldimines. *Tetrahedron* **69**, 4226–4235 (2013).
99. Ma, X., Farndon, J. J., Young, T. A., Fey, N. and Bower, J. F. A Simple and Broadly Applicable C−N Bond Forming Dearomatization Protocol Enabled by Bifunctional Amino Reagents. *Angew. Chem. Int. Ed.* **56**, 14531–14535 (2017).
100. Fier, P. S. and Maloney, K. M. Direct Conversion of Haloarenes to Phenols under Mild, Transition Metal-Free Conditions. *Org. Lett.* **18**, 2244−2247 (2016).
101. Cai, Y.-M. *et al.* Photoinduced Hydroxylation of Organic Halides under Mild Conditions. *Org. Lett.* **21**, 8479–8484 (2019).
102. Akula, M., Blevins, D., Kabalka, G. and Osborne, D. Synthesis of N-[4-(2'-[18f]Fluoroalkoxybenzoyl)]- and N-(3-[123i]Iodo-4-methoxybenzoyl)pyrrolidin-2-ones As Potential Brain Imaging Agents. *Heterocycles* **97**, 1226–1236 (2018).
103. Kyburz, E. and Aschwanden, W. 1-(P-Methoxy p-hydroxy, and p-benzyloxy benzoyl)-2-pyrrolidinones. US4369139A (Hoffmann-La Roche Inc, 1983).
104. Shuyan, Z., Hongfeng, D. and Qinggang, T. Preparation Method of Beta-Carboxyl Phosphate. CN107011377 (A) (Nantong Shu Chuang Pharmaceutical Technology Co., Ltd.; Nantong Tongteng Pharmaceutical Co., Ltd, 2017).
105. Zu, W., Day, C., Wei, L., Jia, X. and Xu, L. Dual aminoquinolate diarylboron and nickel catalysed metallaphotoredox platform for carbon–oxygen bond construction. *Chem. Commun.* **56**, 8273–8276 (2020).
106. Liu, W., Wu, Q., Wang, M., Huang, Y. and Hu P. Iron-Catalyzed C–C Single-Bond Cleavage of Alcohols *Org. Lett.* **23**, 8413–8418 (2021).
107. Tuck, J. R., Tombari, R. J., Yardeny, N. and Olson D. E. A Modular Approach to Arylazo-1,2,3-triazole Photoswitches. *Org. Lett.* **23**, 4305–4310 (2021).
108. Koganty, R. R. and Digenis, G. A. A convenient preparation of 18O-dimethylformamide. *J. Labelled Compds.* **10**, 419–422 (1974).
109. Bonner, F. T., Kada, J. and Phelan K. G. Symmetry of the intermediate in the hydroxylamine-nitrous acid reaction *Inorg. Chem.* **22**, 1389–1391 (1983).
